# Familial co-occurrence of congenital heart defects follows distinct patterns

**DOI:** 10.1093/eurheartj/ehx314

**Published:** 2017-07-02

**Authors:** Sabrina G Ellesøe, Christopher T Workman, Patrice Bouvagnet, Christopher A Loffredo, Kim L McBride, Robert B Hinton, Klaartje van Engelen, Emma C Gertsen, Barbara J M Mulder, Alex V Postma, Robert H Anderson, Vibeke E Hjortdal, Søren Brunak, Lars A Larsen

**Affiliations:** 1 Programme for Disease Systems Biology, Novo Nordisk Foundation Center for Protein Research, University of Copenhagen, Blegdamsvej 3, DK-2200 Copenhagen, Denmark; 2 Department of Biotechnology and Biomedicine, Technical University of Denmark, Lyngby, Denmark; 3 Laboratoire Cardiogénétique, Hospices Civils de Lyon, Groupe Hospitalier Est, 59 boulevard Pinel, CBPE, 69677, Bron, France; 4 Department of Oncology, Georgetown University Medical Center, 3970 Reservoir Road, Washington, DC 20057-1472, USA; 5 Center for Cardiovascular Research, Nationwide Children's Hospital, and Department of Pediatrics, Ohio State University, 700 Children's Drive Columbus, OH 43205, Columbus, OH, USA; 6 Division of Cardiology, The Heart Institute, Cincinnati Children's Hospital Medical Center, 3333 Burnet Ave, MLC 2003, Cincinnati, OH, 45229, USA; 7 Department of Clinical Genetics, Academic Medical Centre, Meibergdreef 15, Amsterdam 1105 AZ, The Netherlands; 8 Department of Clinical Genetics, VU University, De Boelelaan 1117, NL-1081 HV Amsterdam, The Netherlands; 9 Department of Cardiology, Academic Medical Centre, Meibergdreef 9, 1105 AZ Amsterdam, the Netherlands; 10 Department of Anatomy, Embryology & Physiology, Academic Medical Centre, Meibergdreef 15, Amsterdam 1105 AZ, The Netherlands; 11 Institute of Genetic Medicine, Newcastle University, Central Pkwy, Newcastle upon Tyne NE1 3BZ, UK; 12 Department of Cardiothoracic Surgery, Aarhus University Hospital, Skejby, Palle Juul-Jensens Boulevard 99, 8200 Aarhus N, Denmark; 13 Wilhelm Johannsen Centre for Functional Genome Research, Department of Cellular and Molecular Medicine, University of Copenhagen, Blegdamsvej 3, DK-2200 Copenhagen, Denmark

**Keywords:** Congenital heart defects, Congenital heart disease, Polygenic inheritance, Familial occurrence, Concordance, Discordance

## Abstract

**Aims:**

Congenital heart defects (CHD) affect almost 1% of all live born children and the number of adults with CHD is increasing. In families where CHD has occurred previously, estimates of recurrence risk, and the type of recurring malformation are important for counselling and clinical decision-making, but the recurrence patterns in families are poorly understood. We aimed to determine recurrence patterns, by investigating the co-occurrences of CHD in 1163 families with known malformations, comprising 3080 individuals with clinically confirmed diagnosis.

**Methods and results:**

We calculated rates of concordance and discordance for 41 specific types of malformations, observing a high variability in the rates of concordance and discordance. By calculating odds ratios for each of 1640 pairs of discordant lesions observed between affected family members, we were able to identify 178 pairs of malformations that co-occurred significantly more or less often than expected in families. The data show that distinct groups of cardiac malformations co-occur in families, suggesting influence from underlying developmental mechanisms. Analysis of human and mouse susceptibility genes showed that they were shared in 19% and 20% of pairs of co-occurring discordant malformations, respectively, but none of malformations that rarely co-occur, suggesting that a significant proportion of co-occurring lesions in families is caused by overlapping susceptibility genes.

**Conclusion:**

Familial CHD follow specific patterns of recurrence, suggesting a strong influence from genetically regulated developmental mechanisms. Co-occurrence of malformations in families is caused by shared susceptibility genes.

## Introduction

Congenital heart defects (CHD) affect up to 8 of 1000 newborns, making heart defects the most common congenital malformations.^1^^,^^2^ The defects comprise an assortment of structural malformations, ranging from insignificant defects to complex life-threatening malformations, which require highly specialized medical care. The disorder is genetically heterogeneous and can be caused by both single nucleotide variants within genes as well as genomic copy number variants which may affect several genes. The defects may present as isolated malformations or in combination with other birth defects.^3^ Most patients survive into reproductive age, increasing the need for accurate counselling regarding the risk for recurrence.^4^ The overall risk of recurrence has been estimated to be from 3 to 9%,^5–8^ with variation depending on the malformation in question, indicating that some malformations are more heritable than others.^6^^,^^9–13^

Because of the varying severity of malformations, an important factor to consider when counselling families is the ability to predict the type of lesion which might recur. Several studies have investigated the patterns of recurrence of similar, or concordant, as opposed to different, or discordant, types of malformation.^5^^,^^10^^,^^14–19^ It has previously been suggested that familial occurrence of certain malformations might be determined by cardiac developmental biology, and that this is reflected in specific patterns of familial co-occurrence of malformations. Conflicting results, however, have left this matter unresolved.^15^^,^^18^^,^^20^ A possible reason for the divergent results may be that many previous studies have been based on small numbers of affected pairs or that part of the malformations have not been confirmed by diagnostic methods or inspection of medical records.^14^^,^^16^^,^^18^^,^^19^

In this study, therefore, we sought to determine if familial co-occurrence of lesions follows specific patterns by analysing their co-occurrence in 1163 families, in which 3080 family members had a clinically confirmed diagnosis. We calculated concordance and discordance rates for 41 different types of lesion, and analysed the patterns of discordant pairs within families in order to investigate if specific constellations of malformations occur in families. We hypothesized that shared susceptibility genes could mechanistically explain the observed patterns of co-occurrence in families, testing our hypothesis using data from mouse models and patients.

## Methods

### Study population

**Figure 1:**
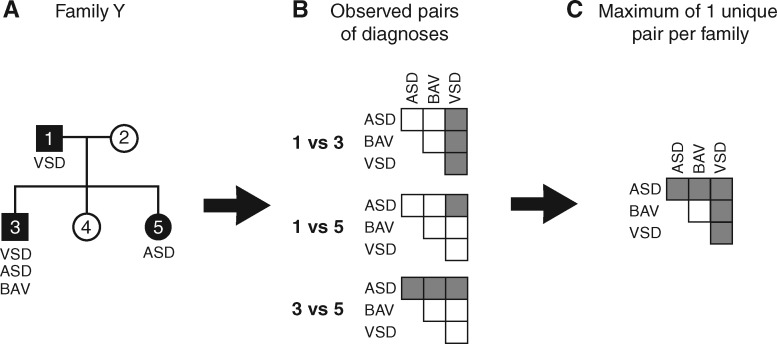
The number of unique pairs of diagnoses per family was tailed. The total number of times a pair was observed between families was used in calculating the odds ratio for different pairs of diagnoses. (*A*) Example of a family with three affected individuals. (*B*) Possible pairs of diagnoses observed between individuals. (*C*) A maximum of one unique pair was included per family. See Supplementary material online, *Table S2* for abbreviations.

Data are based on 637 previously unpublished families and 526 families from 187 peer reviewed papers (see Supplementary material online, *Table S1*). Only clinically confirmed malformations were included in our analyses. We excluded 197 individuals from our analyses due to unverified malformations. (additional information in Supplemental material online). Patients were assigned International Pediatric Congenital Cardiac Codes (IPCCCs, www.ipccc.net) (see Supplementary material online, *Table S2*, *Figure **1*A).

### Data analysis

Odds ratios (OR) were calculated as in Agresti *et al.*^21^ We counted the number of unique pairs of diagnoses in a family as opposed to the number of observed phenotype-pairs, as this number expands multiplicatively in families with many affected individuals, ensuring that concordant and discordant events were only recorded between affected family members (*Figure **1**B* and *C*).

Grouping of the malformations was performed using hierarchical clustering analysis according to Ward’s hierarchical agglomerative clustering method.^22^ Significant differences in gender ratio were determined using χ^2^ testing. Difference between rate of first-degree relatedness (RFR) was calculated using a two-sided Mann–Whitney *U* test. Significance of overlapping susceptibility genes was calculated using Fisher’s exact test and adjusted for multiple testing. Additional information regarding data analysis, including a comparison of published and unpublished data and analysis for negative selection bias, is provided in Supplementary methods.

## Results

We included in the study a total of 1163 families with 3080 individuals with verified CHD, of whom 1591 were male. Pedigrees are shown in Supplementary material online, *Figures S2* and *S3*. The average number of individuals within the families was 8.8, with the average number of individuals with verified diagnoses per family being 2.65 (±1.45). We observed 159 families (13.7%) with more than three affected individuals, of which only 9 families had more than 10 affected individuals. A total of 86 families (7.4%) were observed with affected individuals in three or more generations.

**Figure 2:**
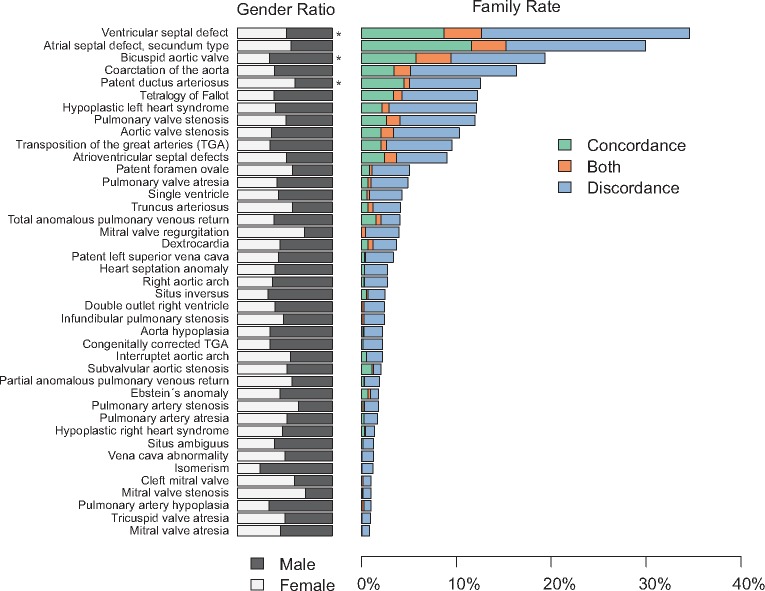
Gender ratio, concordance and discordance in 1163 congenital heart defects families. A family was considered to be concordant (green bar) if all affected individuals shared the same diagnosis. A family was considered to be discordant (blue bar) if all affected individuals had different diagnoses. If a family had family members that shared some of the diagnoses it was considered as both concordant and discordant (orange bar). *Statistical significant differences.

We observed possible autosomal recessive, autosomal dominant and X-linked inheritance patterns among the families, but for a large fraction of the families several patterns of inheritance are possible, due to the small family sizes. As deduced from apparent obligate carriers, we noticed reduced penetrance in 371 families (32%), suggesting that this is a common phenomenon in familial congenital heart disease. The most common malformations in the families were atrial septal defects, followed by ventricular septal defects, bicuspid aortic valve, aortic coarctation and persistent patency of the arterial duct, these lesions affecting 685, 587, 387, 259 and 252 individuals, respectively. We observed a significant male predominance for bicuspid aortic valve and female predominance for atrial septal defects and patency of the arterial duct (*Figure **2*).

### Co-occurrence of pairs of malformations in families

Family rates of concordance and discordance were calculated for each malformation in the dataset (*Figure **2*). For most malformations, the frequency of discordance was higher than the frequency of concordance, suggesting that a high degree of discordance is a common phenomenon in familial congenital cardiac disease. We observed a few families with more than seven affected individuals, with these families displaying a high degree of concordance, possibly caused by a single mutation with high penetrance.

**Figure 3:**
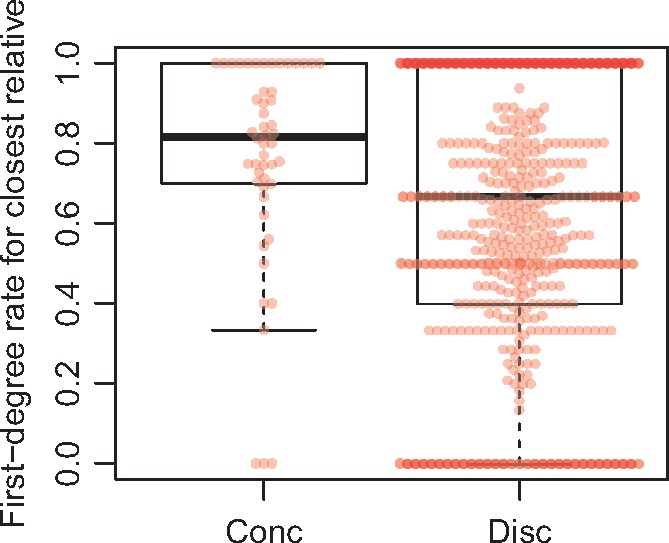
Comparison of familial relatedness with concordance and discordance. Relatedness is shown as a rate of first-degree relatedness, of which first-degree relatives have a rate of 1. The rate decreases for more distant relatives.

To compare rates of concordance and discordance with relatedness in the families, we calculated the RFR (see Supplementary methods). The average RFR was significantly larger for concordant compared with discordant pairs (0.81 vs. 0.68, respectively, *P* = 0.011) (*Figure **3*), suggesting that concordance and relatedness is correlated.

Odds ratios were calculated for 1640 pairs of diagnoses observed in the data, with 178 pairs passing the confidence interval test (CI did not span 1). Odds ratios for the most common (>25 observations in the data) discordant pairs with increased relative risk are shown in *Table 1*. The full list of all 178 pairs is available in Supplementary material online, *Table S4*.

We also found pairs of diagnoses with ORs <1, suggesting decreased relative risk (*Table 1*). For example, the previous occurrence of an atrial septal defect in a family suggests a low likelihood of the occurrence of either tetralogy of Fallot (OR = 0.26) or hypoplastic left heart syndrome (OR = 0.35) in other family members.

**Table 1:** Odds ratios for pairs of malformations

Diagnosis A	Diagnosis B	OR	CI	# Pairs	# Families
CHD with OR > 1^a^					
ASD	EbA	6.1	2.3–16	42	15
AVS	BAV	7.4	4.8–11	165	59
AVSD	PLSVC	6.6	3.2–13.4	30	13
AVSD	SV	9.9	1.9–7.8	27	12
AVSD	CMV^b^	123.7	15.8–969	46	11
BAV	CoA	3	2.1–4.3	165	59
DORV	VSD	3.4	1.3–8.7	32	12
Dxc	PVS	6.2	3.2–12	39	17
Dxc	TAPVR	12	5.4–25	29	11
Dxc	HSA	23	10–54	25	11
IAA	VSD	6.7	2.5–18	41	17
RAA	TOF	3.6	1.7–7.9	29	10
TA	VSD	4.2	2.2–8.1	76	29
TGA	Dxc	6.1	3.0–12	31	13
TGA	TAPVR	3.7	1.8–7.3	30	12
TGA	SV	5.4	2.7–11	27	12
TGA	PVA	3.1	1.5–6.2	27	11
CHD with OR < 1^c^					
ASD	BAV	0.21	0.14–0.34	77	22
ASD	HLHS	0.35	0.21–0.57	52	19
ASD	TOF	0.26	0.15–0.45	45	16
AVS	VSD	0.21	0.12–0.38	32	13
AVS	PDA	0.35	0.15–0.81	17	6
AVS	TOF	0.17	0.05–0.53	6	3
AVS	TGA	0.22	0.07–0.71	6	3
AVSD	BAV	0.28	0.13–0.62	15	7
BAV	VSD	0.23	0.15–0.34	65	28
BAV	PDA	0.34	0.18–0.62	34	12
BAV	PVS	0.17	0.07–0.39	15	6
BAV	TOF	0.08	0.25–0.25	7	3
BAV	TGA	0.11	0.03–0.34	8	3
CoA	PVS	0.36	0.19–0.71	26	10
CoA	TOF	0.10	0.03–0.31	6	3
HLHS	TGA	0.33	0.13–0.82	13	5
HLHS	TOF	0.09	0.02–0.37	4	2

See Supplementary material, *Table S2* for abbreviations.

# Pairs, the total number of pairs; # Families, the number of families; CI, confidence interval; OR, odds ratio.

a Odds ratios for pairs occurring more than 25 times between individuals.

b We are aware that the correct anatomical term in this setting is ‘cleft left AV valve’. Some CMV diagnoses may be AVSD.

c Odds ratios for pairs of diagnoses observed less often than expected from the total number of occurrences in the data. The shown pairs are observed more than 100 times without the other (A without B and B without A, respectively).

### Analysis of group-wise co-occurrence of discordant malformations

**Figure 4:**
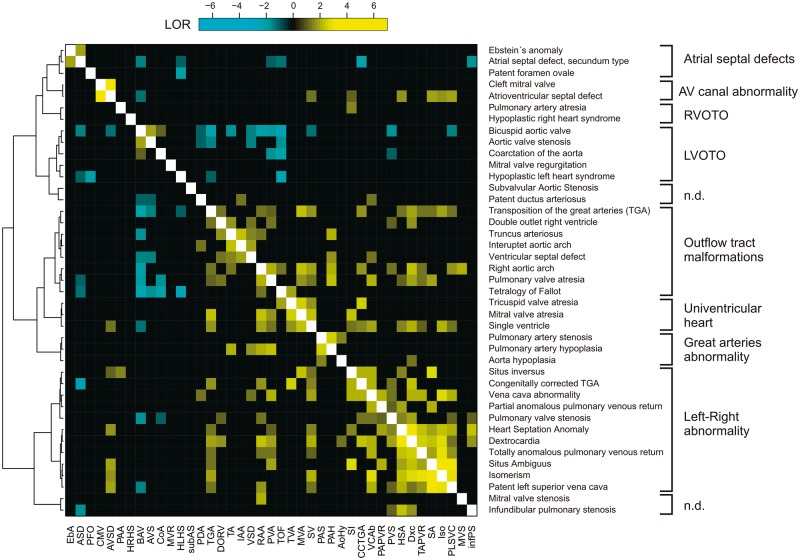
Patterns of co-occurrence of phenotypes in congenital heart defects families. The log-odds ratio (LOR) for 1640 pairs of malformations is displayed as a heatmap. Only pairs occurring ≥10 times in the dataset were included. To enhance readability only LOR values ≤ −1 and ≥1 are shown. The data were grouped according to similarities in LOR using hierarchical cluster analysis. An anatomical term for each group is suggested on the right. LVOTO, left ventricular outflow tract obstruction; RVOTO, right ventricular outflow tract obstruction; n.d., not determined. International Pediatric Congenital Cardiac Code diagnoses are indicated in full and abbreviated.

We analysed the data for specific patterns of familial co-occurrence of malformations using an unbiased, data-driven approach. Hierarchical clustering analysis was used to group ORs calculated for the 1640 pairs of diagnoses (*Figure **4*). This analysis revealed 10 major groups with similar patterns of co-occurrence, confirming that specific groups of phenotypes co-occur within families. Our data also suggest that certain malformations co-occur in more than one group, for example discordant ventriculo-arterial connections and atrioventricular septal defect. We compared our chosen groups of lesions with the taxonomy proposed by Houyel *et al.*^23^ (see Supplementary material online, *Figure S10*), revealing some differences. Outflow tract malformations, for example, are split in three groups in our data, compared with one large group in their taxonomy.

### Sharing of susceptibility genes in co-occurring malformations

**Figure 5:**
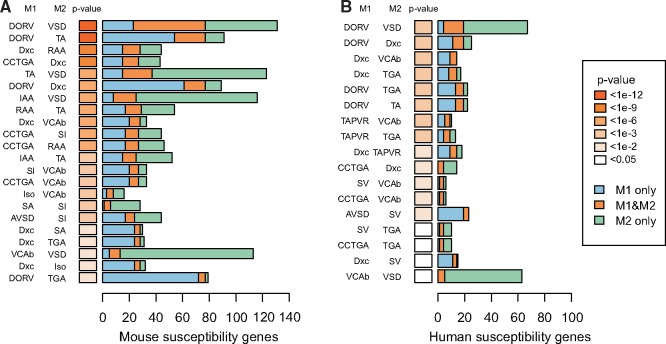
Overlap of deduced susceptibility genes between discordant phenotypes. Mouse (*A*) and human (*B*) susceptibility genes were identified (numbers shown in bar chart) and malformations were analysed in pairs (M1 and M2, indicated on the y-axis).

To test whether the observed patterns of co-occurrence of discordant malformations could be explained by shared susceptibility genes, we identified sets of genes which cause cardiac malformations in mice, corresponding to the cardiac lesions we observed within families (see Supplementary material online, *Table S5*). The overlap between such deduced susceptibility genes was calculated for each discordant pairs of 41 malformations, testing significance of the overlap using Fisher’s exact test, with correction for multiple testing (see Supplementary material online, *Figure S11*). We found that 110 pairs have a log-odds ratio (LOR) ≥ 1 (marked yellow in *Figure **4*), suggesting that they often co-occur in families, while 27 pairs have a LOR ≤ −1 (marked cyan in *Figure **4*), suggesting that they rarely co-occur in families. We observed significant gene-overlap between 22 pairs (20.0%) out of the 110 discordant pairs with LOR ≥ 1 (*P* = 1.69e-06, Fishers exact test) (*Figure **5*A). None of the 27 pairs of malformations with LOR ≤ −1 displayed significant gene overlap. We observed similar results when using a smaller set of human susceptibility genes (19.1% overlap, *P* = 0.00017) (*Figure **5*B).

## Discussion

We have investigated the co-occurrence of cardiac malformations presenting in families with congenital cardiac disease. The dataset differs significantly from population-based cohorts and registry-based studies.^1^^,^^7^^,^^24^ It is not possible, therefore, to calculate recurrence risk from our dataset. Our dataset, nonetheless, does permit high-confidence calculation of co-occurrence ORs for a wide range of lesions. Thus, we believe that the data could be helpful in the counselling of families in whom congenital cardiac disease has previously been observed.

We observed a majority of small families, a low abundance of large and multigenerational families, and a high frequency of reduced penetrance. We only included verified diagnoses, thus the number of affected individuals per family is likely to be an underestimate and many factors could potentially influence family size. Nevertheless, our data contradict a monogenic mode of inheritance, favouring a polygenic model. The abundance of families of small size is in agreement with previous observations of higher recurrence risk in first degree relatives when compared with second and third degree relatives.^14^^,^^19^

Our data suggest that families are predominantly discordant for cardiac malformations in agreement with previous reports.^5^^,^^10^^,^^16^ Between one and two-fifth of families with occurrence of ventricular and atrial septal defects, aortic coarctation, patency of the arterial duct, and tetralogy of Fallot, nevertheless, display a high degree of exact concordance. Exact concordance in a proportion of familial incidence of tetralogy of Fallot was reported recently.^14^^,^^19^ Examples of predominantly concordant families segregating a single mutation in *NKX2-5*, *GATA4* or *ACTC1* have also been published previously.^25–28^

Our data suggest correlation between concordance and relatedness, but the large range of RFR in discordant pairs suggests that this relationship is far from simple. Similar correlation was observed in a recent study of familial tetralogy.^14^ An explanation for these observations may be that the number of different genetic modifiers of the different lesions increases with decreasing relatedness.

Specific patterns of co-occurrence of malformations were even more pronounced when analysed by hierarchical cluster analysis, which suggest that recurrence of defects follows specific patterns. In this regard, our data support the notion that co-occurrence of defects in families is determined by perturbations of specific developmental mechanisms.^15^^,^^20^ This hypothesis has yet to be proven, but recent evidence from genetic studies of unrelated patients suggests that the lesions are caused by disturbances of the molecular networks which control cardiac development.^29–32^ We find it likely that a similar mechanism is responsible for the group-wise co-occurrence of malformations we observe in families. This suggests that genetic defects inherited in families perturb a signalling network or developmental mechanism responsible for formation of the particular cardiac anatomical structures affected in the given patients.

Our data contradict the findings by Oyen *et al*.,^18^ who did not find any distinct patterns of co-occurrence. Their study, however, was performed on unvalidated registry data, which we recently reported to have a false discovery rate of over one-third.^33^ Thus, false positive familial occurrences in the registries could have confounded the analyses in that particular study. In addition, Oyen *et al.* classified their malformations according to the system proposed by Botto *et al*.^34^ Their data were combined into 13 large groups of malformations before analysis, which may have concealed some patterns of co-occurrences.

Clinical classification of congenital cardiac disease is challenging, and several systems have been proposed. Most recently, Houyel *et al.*^23^ proposed a classification system based on IPCCC. This system is based on anatomy, echocardiography and criteria for clinical management, but not as yet on presumed developmental mechanisms. The group-wise co-occurrence of malformations we observe in families is partly in agreement with this taxonomy, but we also observe differences. We propose that some of our observed differences may indeed reflect an influence from underlying genetic or developmental mechanisms.

We found a high frequency of discordance for atrioventricular septal defect, which may suggest that familial exact concordance of this lesion is lower than previously reported.^5^^,^^35^ However, it is possible that some of the diagnoses of clefting of the mitral valve may, in reality, represent deficient atrioventricular septation. The hierarchical clustering analysis suggested that familial occurrence follows two distinct patterns. In the first pattern, atrioventricular septal defects, including clefting of the mitral valve, seem to be an isolated entity, while a second pattern includes those with abnormalities in left–right patterning, or the so-called ‘heterotaxy’. Our data, therefore, may suggest that two distinctly different developmental mechanisms can lead to deficient atrioventricular septation, and that it is very rare for both of these mechanisms to occur in one family. This is of clinical importance, since surgical correction of deficient atrioventricular septation when combined with abnormalities in left–right patterning is usually more complicated than treatment of isolated defects.

Clustering analysis grouped transposition of the great arteries together with other malformations involving the outflow tract. This showed a strong association between transposition and abnormalities in left–right patterning, suggesting a significant contribution to the factors producing the discordant ventriculo-arterial connections from the developmental mechanism which also control asymmetry, an association reported previously by several groups.^36–39^

Double outlet right ventricle, closely related anatomically to discordant ventriculo-arterial connections when associated with a subpulmonary interventricular communication, presented with a different pattern of co-occurrence for that seen for the discordant ventriculo-arterial connections. In our hierarchical clustering, double outlet was grouped together with the discordant ventriculo-arterial connections as outflow tract malformations, but the association between double outlet and lateralization abnormalities was weak compared with the association noted for the overall combination of discordant ventriculo-arterial connections and concordant atrioventricular connections. This suggests that parts of the molecular mechanisms responsible for the overall groups of double outlet as opposed to discordant ventriculo-arterial connections may be different, or that our data were insufficient to detect the different phenotypes making up the overall group of double outlet right ventricle. In this regard, nonetheless, Peyvandi *et al.*^19^ did note differences in the pattern of risk of recurrence between patients having transposition as opposed to double outlet.

An aortic valve with two leaflets is found in up to 2% of the general population. Such individuals account for up to half of those with stenotic aortic valves, and may also carry a risk of bacterial endocarditis and aortic dissection.^40^^,^^41^ A better understanding of the aetiology of the bifoliate valve, therefore, is important. In our analysis, we grouped the bicuspid valve with other malformations involving the left ventricular outflow tract, finding that the bicuspid valve displays a high OR with both aortic coarctation (OR = 3) and aortic valvar stenosis (OR = 7.4). In keeping with previous reports,^12^^,^^40^ this suggests a common developmental mechanism. We also observed negative associations with 11 different malformations, suggesting that familial occurrence of the bifoliate valve is almost exclusively related to malformations involving the left ventricular outflow tract.

We used data from mouse models and human disease genes to investigate if familial co-occurrence of malformations could be explained by the sharing of susceptibility genes. Translation of clinical diagnoses into cardiac phenotypes observed in mice is not always straightforward and an unknown part of the phenotypes reported in the MGI database could well be misclassified. Both gene sets, nonetheless, showed that one-fifth of pairs of malformations have a significant overlap of susceptibility genes. Since it is likely that many genes associated with cardiac malformations in mice and humans remain to be found, our findings could well be underestimated.

Similar phenotypic pleiotrophy has been observed in previous reports of families segregating a known mutation with large effect, supporting that a single gene can be associated with more than one type of congenital cardiac malformation in a family.^25^^,^^42^^,^^43^

We have shown, therefore, that depending on the malformation, co-occurrence of CHD in families is highly variable. In general, discordance can be expected twice as often as concordance. Familial co-occurrence of discordant heart defects, however, follows distinct patterns, which suggests an underlying developmental mechanism, such as the sharing of susceptibility genes.

## Supplementary material

Supplementary material is available at *European Heart Journal* online.

## Supplementary Material

Supplementary Data
