# Supplementary material for: Familial co-occurrence of congenital heart defects follows distinct patterns
Source: Eur Heart J. 2017 Jul 2;39(12):1015–22. doi: 10.1093/eurheartj/ehx314 (PMC6018923; doi:10.1093/eurheartj/ehx314)
Supplement: Supplementary Data [file ehx314_supplemental_material_ellesoe_et_al._r4.pdf]

SUPPLEMENTAL MATERIAL

Familial co-occurrence of congenital heart defects follows distinct patterns

Sabrina G. Ellesøe; Christopher T. Workman; Patrice Bouvagnet; Christopher A. Loffredo; Kim L. McBride; Robert B. Hinton; Klaartje van Engelen; Emma C. Gertsen; Barbara J.M. Mulder; Alex V. Postma; Robert H. Anderson; Vibeke E. Hjortdal; Søren Brunak; Lars A. Larsen

The supplemental material contains:

	Page
Supplemental Methods	2-6
Initial comparison of the published and unpublished data sets	6
Comparison of sib-pairs and parent-offspring pairs	6-7
Data analysis	7-9
Exclusion of families with known monogenic cause	9-10
Analysis of negative selection in families	10
Supplemental Table 1	11-24
Supplemental Table 2	25-27
Supplemental Table 3	28-40
Supplemental Table 4	41-44
Supplemental Table 5	45-46
Supplemental Table 6	47
Supplemental Table 7	48
Supplemental Figure 1	49
Supplemental Figure 2	50-209
Supplemental Figure 3	210-342
Supplemental Figure 4	343
Supplemental Figure 5	344
Supplemental Figure 6	345
Supplemental Figure 7	346
Supplemental Figure 8	347
Supplemental Figure 9	348
Supplemental Figure 10	349
Supplemental Figure 11	350
Supplemental Figure 12	351
Supplemental Figure 13	352
Supplemental references	353-358

Supplemental Methods

Most of the diagnoses in our dataset are based on retrospective studies of database records, patient files and published articles. This design allowed us to collect data from a large number of CHD families and we applied stringent quality criteria to the data we included (see Methods). But a weakness of this approach is that we rely on skills of the consulting cardiologist and therefore we cannot exclude that diagnostic errors could have occurred and minor abnormalities such as a bicuspid aortic valve might not be have been noted in the written report. However, the vast majority of diagnoses were established by pediatric cardiologists from tertiary centers, and thus we believe that the error rate is low.

In many cases it was not possible to distinguish between muscular and membranous VSDs because such detailed information was unavailable and we decided to pool all VSDs into one group.

We included previously unpublished CHD families collected by one of the authors. These families were labeled “Unpublished”. We also included families published in peer-reviewed journals and cited in key literature from 1953-2013. These were labeled “Published”. A list of the papers included from the literature is shown in Supplemental Table 1. Families were included if they fulfilled the following inclusion criteria: 1) The CHD had been confirmed by, echocardiography, heart catheterization, surgery or autopsy; 2) two or more affected individuals from the same family had a verified CHD; and 3) a pedigree was available, or construction of a pedigree was possible from information given in the paper or by the collaborator.

The two datasets include 1,163 families, comprising 10,278 individuals, of whom 3,080 had a clinically confirmed diagnosis. A total of 197 individuals were not included in the analysis because their diagnoses were unverified and, in most cases, also impossible to place in one of the 59 International Pediatric Congenital Cardiac Codes. However, we find it likely that many of these

individuals may have CHD. We have included a table showing the number of excluded individuals per family (Supplemental Table 3).

Ellesoe families (n=201)

In Denmark, all citizens have a central population registry number (cpr) number assigned at birth or immigration. The cpr-number serves as a unique identifier in the registries and enables cross-linking of information reported by doctors in hospitals. The Civil Registration System holds information on first, second and in some instances third degree relatives that are organized by cpr-number and therefore discoverable. A copy of the patient file was manually reviewed and the diagnosis was confirmed in two or more related individuals, before the family was included in the project. The patients were not screened by array CGH or tested for 22q11 deletions.

McBride families (n=59)

Pediatric patients with CHD were identified via a search of the patient databases of the Division of Cardiology, Dept. of Pediatrics at Baylor College of Medicine, or by referral. The families of the probands were contacted and enrolled after obtaining informed consent. The diagnoses were documented by echocardiography or cardiac catheterization. The patients were not screened by array CGH or tested for 22q11 deletions. Patients with Turner syndrome were excluded from the study.

Postma families (n=54)

Probands who had at least one relative with CHD were referred to the clinical genetics department by the cardiologist or pediatric cardiologist. The families of the probands were contacted and

informed consent was obtained. The diagnoses were documented by echocardiography or cardiac catheterization or by retrospective review of the patient files. All families were seen by clinical geneticists, none of the families included in the study presented with syndromic features. Probands in family 247, 761 and XXE were screened for 22q11 deletion, all with negative result.

Hinton families (n=21)

Hinton (RBH) families were originally published in 2007 (187, Table S1). Thus these families are included in the group of "Published data" and shown in figure S3. However, for the current work, additional information was provided by RBH. All the probands were identified through electronic medical records maintained in the Division of Cardiology at Cincinnati Children's Hospital Medical Center. The first-degree relatives were screened and if any of these were affected their first-degree relatives were screened etc. The diagnoses were validated retrospectively by review of the patient file and the echocardiography. The patients were not screened by array CGH or tested for 22q11 deletions. Patients with Turner syndrome or genetic syndromes were excluded from the study.

Bouvagnet families (n=262)

The LYS_Z, LYS_S and LYS_M families (n=210) were identified through a French National Registry established in 2004. Pediatric cardiologists, adult cardiologists, geneticists, fetopathologists, surgeons and other physicians were asked to identify such families and to inform patients and families of the register. Phenotyping was performed retrospectively by review of the patient file of all probands and their affected relatives.

The LYS_AKBA-LYS_ZS&S families were collected by writing systematically to pediatric cardiologist of the world (mainly Japan, USA, USA, Germany and North Africa). The diagnoses

were verified by the cardiologist sharing the pedigree(s) by echocardiography, cardiac catheterization, autopsy or surgery.

Probands from the following families were screened with array CGH; LYS_M006 (normal), LYS_M147 (normal), LYS_M165 (duplication of exon1 in FAM123), LYS_M169 (del5q35.1), LYS_M192 (normal), LYS_M220 (normal), LYS_M232 (normal), LYS_MC013 (normal), LYS_MC019 (normal), LYS_S020 (normal), LYS_M061 (dupl 2q37.3). Probands in the following families were tested negative for 22q11 del; LYS_M025, LYS_M070, LYS_M097, LYS_M100, LYS_M100, LYS_M112, LYS_M118, LYS_M147, LYS_M181, LYS_M185, LYS_M235, LYS_M238, LYS_MC082, LYS_S071, LYS_S198, LYS_Z107. A 22q11 duplication was identified in families LYS_M074 (III.3) and LYS_M089 (II.2 and II.3).

Loffredo families (n=61)

Three pediatric cardiology centers collaborated on this project: the University of Maryland and Johns Hopkins University in Baltimore, and the University of Rochester, New York. At each center, pediatric cardiologists identified newly diagnosed infants with HLHS, COA, or TGA; eligible subjects were live-born and diagnosed in the first year of life during 1990–1993. Parents were approached and invited to participate along with their children and as many relatives (aunts, uncles, cousins, and grandparents) as could be recruited. After providing signed informed consent, the parents attended an interview and were asked about occurrences of congenital heart defects among their children and relatives, and they provided details for the construction of complete family pedigrees. Consent for medical record review was obtained from all participating adults, and chart reviews confirmed reported cardiac anomalies in 2nd and 3rd degree relatives. All consenting 1st degree relatives of probands (siblings and parents) were individually examined by clinicians. In addition, all consenting 1st degree relatives of any parent with congenital heart defects (aunts,

uncles, and grandparents of the proband) were examined at the study centers. Each participant received a clinical examination followed by an echocardiogram including two-dimensional and color flow/Doppler imaging, performed by experienced technicians under direct physician supervision. The patients were not screened by array CGH or tested for 22q11 deletions.

Initial comparison of the published and unpublished data sets

Before merging of the published and unpublished data sets, we investigated whether major differences existed between these by plotting the rates of discordant and concordant pairs (different and identical diagnoses, respectively) observed in the *Unpublished* and *Published* data. We calculated the R squared value as a measure of deviation from the mean. Likewise, we also compared the number of affected individuals per family versus the family size (total number of individuals in the family) to address any major differences between the two data sets.

The family sizes and occurrence of malformations was homogeneous between the published and unpublished datasets (Supplemental Figure 4 and 5) and we found it acceptable to merge the two datasets into one. Below we present the results of analyses of the merged data.

Comparison of sib-pairs and parent-offspring pairs

Peyvandi and colleagues analyzed heart malformations in 258 affected relatives to probands presenting with a conotruncal defect (50). They counted the number of simple and complex malformations in parents to the probands and compared these numbers to the distribution of simple and complex malformations in siblings to the probands. Their data showed that parents often presented with simple malformations compared to probands, while siblings presented tended to present with more complex malformations, suggesting a negative selection bias.

To test for a possible effect from a negative selection bias on distribution of odd-ratios in our families, we assigned a severity score between 1 and 3 (1=low severity, 2=intermediate severity, 3=high severity) to each of the 59 malformations observed in the families (severity score is listed in Supplemental Table 2). Next, we analyzed the cumulative distribution of log-odds ratios for the observed combinations of severities in our dataset. We did not observe any difference in the distribution of log-odds ratios when we compared sibling-pairs with parent-offspring pairs (Supplemental figure 6). To test if negative selection might influence concordance or discordance, we counted the number of concordant and discordant pairs in sib-pairs and parent-offspring pairs. We did not observe any difference in overall distribution of concordant and discordant pairs between the two groups (Supplementary table 7). Based on these analyses we conclude that neither the log-odds ratios of discordant malformations nor the distribution of concordance or discordance is significantly influenced by negative selection.

Data analysis

Significant differences in gender ratio were determined using a chi-square test for the proportion of affected males versus affected females for each phenotype relative to the overall numbers of affected males and females in the study (2074 males, 1820 females). A significance level of 0.05 was applied after adjustment for multiple hypothesis testing (Holm method).

The OR between phenotypes A and B depended upon the number of families where A was observed but not B, $\mathrm{n}(\mathrm{A},\overline{\mathrm{B}})$, where B was observed and not A, $\mathrm{n}(\overline{\mathrm{A}},\mathrm{B})$, and the number of families where neither A nor B were observed, $\mathrm{n}(\overline{\mathrm{A}},\overline{\mathrm{B}})$. Based on these family counts, the OR was:

$\mathrm{OR}(\mathrm{A},\mathrm{B}) = \mathrm{n}(\mathrm{A},\mathrm{B})\mathrm{n}(\overline{\mathrm{A}},\overline{\mathrm{B}})/\mathrm{n}(\mathrm{A},\overline{\mathrm{B}})\mathrm{n}(\overline{\mathrm{A}},\mathrm{B})$.

To compare concordance and discordance rate with relatedness in the families, we observed the family relationship between the closest affected family members. More specifically, for each effected individual we identified the closest concordant family member in the pedigree, if present, and assessed their relationship as first degree or not as measured by the kinship coefficient, $\phi \leq 0.25$ (related to the fraction of alleles that are identical by descent). This process was repeated for each combination of discordant phenotype pairs observed. The rate of first-degree relatedness (RFR) was then calculated for concordant and discordant phenotype pairs. A RFR close to 1 in these instances indicated that this concordance or discordance presented predominantly between first-degree relatives. To investigate if there was an overall bias for the RFR in concordance to be higher (more likely first-degree) than the RFR in discordance, we applied a two-sided Mann–Whitney U test. The result of this test indicated that the RFR of concordance was indeed higher than RFR of discordance ($p = 0.011$).

To investigate the similarity of diagnoses, we performed a hierarchical clustering analysis. Eight different clustering methods were applied, in order to investigate whether any groups were consistent across clustering methods. The clustering method compares the malformations according to their similarities in co-occurrences and split them into groups based on these similarities. The inverse ORs (1/OR) was used as a distance measure. After clustering, ORs with confidence intervals (CIs) spanning 1 were omitted. We found several clusters to be highly conserved across the different methods, suggesting with high confidence some of the co-occurring groups (Supplemental Figure 6). However, some diagnoses shifted position between groups depending on what clustering method was applied, indicating that the co-occurence between these specific diagnoses could be interpreted differently. This could indicate that these malformations were likely to associate with several groups, or that the amount of data was insufficient to result in robust groupings. We did not investigate these differences in detail, but decided to base the grouping on

Ward's hierarchical agglomerative clustering method, where distances were estimated by OR^{-1/2}.

Due to a high degree of genetic, functional and anatomical conservation, the mouse is generally accepted as a valid model for human cardiac development. In order to test whether the observed groups were caused by overlap of genes, we searched the Mouse Genome Informatics database (http://www.informatics.jax.org) for genes related to the IPCCCs observed within the 1,163 families. For each of 42 types of human clinical diagnoses, corresponding cardiac phenotype terms in mice were identified using the Mammalian Phenotype Ontology in MGI. These phenotype terms were used to search the MGI database for deduced susceptibility genes, corresponding to the 42 human cardiac phenotypes (Supplemental Table 5). For example; the human clinical phenotype “Pulmonary Artery Atresia” (PAA, IPCCC code 09.10.21) corresponds to the Mammalian phenotype term “pulmonary artery hypoplasia” (MP:0010460) in MGI. According to MGI, four genes are known to cause this phenotype, when mutated. Human susceptibility genes were identified from the literature (Supplemental Table 6).

The overlap of genes between each pair of phenotypes was assessed by a two-sided Fisher’s exact test (significance level 0.05) after correction for multiple testing by the Benjamini-Hochberg method. For this gene set enrichment test, the union of all genes assigned to phenotypes was used as the sample space.

Statistical software R version 3.1.2 was used for all analyses. R packages *pedigree and kinship2* were used for pedigree construction.

Exclusion of families with known monogenic cause.

We analyzed if exclusion of families with a known monogenic cause would change the overall conclusions. By assessing our own data from the 637 unpublished families, we found that 49

families (7.6%) had a known monogenic cause. Out of 526 published families, 34 families (6.5%) have a known monogenic cause. We excluded these families from the dataset, and reanalyzed the data. We did observe minor differences in the results, but the rates of concordance and discordance, and the grouping of phenotypes, did not change significantly (please see Supplemental figures 9 and 10). Manual inspection of the 49 unpublished families did reveal families with a high degree of concordance, but we also observed many families without such concordance. We did not observe any general tendency towards higher concordance in families with monogenic disease (data not shown).

Analysis of negative selection in families

Peyvandi et al. grouped malformations identified in CHD families as “simple” or “Complex” and calculated the numbers in parent-offspring pairs and sibling pairs (50). They found that complex malformations were found in 20% of parent-offspring pairs, compared to 58% of sibling pairs and suggested that an explanation for this difference could reflect survival bias and reproductive fitness. To compare our data with the data presented by Peyvandi et al., we calculated the number of pairs with different combinations of severities, and compared the distributions in parent-offspring pairs and sibling pairs (Supplemental Table 7). We observed approximately two times higher frequency of pairs which included the most severe malformations (severity 3) among siblings compared to parent-offspring pairs. Thus our data support the observations reported previously by Pervanti et al.

Supplemental Table 1. References on published families. Overview of the 187 papers published in peer-reviewed journals reporting familial CHD and where diagnoses had been confirmed in at least two related individuals by echocardiography, heart catheterization, surgery or autopsy.

1	Abushaban L, Uthaman B, Kumar AR, Selvan J	Familial truncus arteriosus: a possible autosomal-recessive trait	*Pediatr Cardiol*	2003;24(1):64–6
2	Adams HD	Fallot's tetralogy in twins	*Cardiovasc Dis Bull Texas Hear Inst*	1974;1(2):85–86
3	Alonso S, Pierpont ME, Radtke W, et al	Heterotaxia Syndrome and Autosomal Dominant Inheritance	*Am J Med Genet*	1995;56:12–15
4	Amati F, Mari A, Mingarelli R, et al	Two Pedigrees of Autosomal Dominant Atrioventricular Canal Defect (AVCD): Exclusion From the Critical Region on	*Am J Med Genet*	1995;57:483–488
5	Arnold GL, Bixler D, Girod D	Probable autosomal recessive inheritance of polysplenia, situs inversus and cardiac defects in an Amish family	*Am J Med Genet*	1983;16(1):35–42
6	Ashida K, Itoh A, Naruko T, et al	Familial Scimitar Syndrome : Three-Dimensional Visualization of Anomalous Pulmonary Vein in Young Sisters	*Circulation*	2001;103(25):e126–e127
7	Balaji S, Dennis NR, Keeton BR	Familial Ebstein's anomaly: a report of six cases in two generations associated with mild skeletal abnormalities	*Br Heart J*	1991;66(March):26–29
8	Baron P, Gutgesell H, Hawkins E, McNamara D	Infradiaphragmatic total anomalous pulmonary venous connection in siblings	*Am Heart J*	1982;104(5):1107–1109
9	Beekman RH, Robinow M	Coarctation of the aorta inherited as an autosomal dominant trait	*Am J Cardiol*	1985;56:818–819
10	Benson DW, Sharkey A, Fatkin D, et al	Reduced penetrance, variable expressivity and genetic heterogeneity of familial atrial septal defects	*Circulation*	1998;97:2043–2048
11	Bizarro RO, Callahan JA, Feldt RH, Kurland LT, Gordon H, Brandenburg RO	Familial Atrial Septal Defect with Prolonged Atrioventricular Conduction: A Syndrome Showing the Autosomal Dominant Pattern of Inheritance	*Circulation*	1970;41(4):677–683

12	Bleyl S, Ruttenberg HD, Carey JC, Ward K	Familial total anomalous pulmonary venous return: A large Utah-Idaho Family	*Am J Med Genet*	1994;52:462–466
13	Bonnet D, Fermont L, Kachaner J, Sidi D	Tricuspid atresia and conotruncal malformations in five families Clinical governance and genetic medicine	*J Med Genet*	1999;36:349–350
14	Boon AR, Farmer MB, Roberts DF	A Family Study of Fallot's Tetralogy	*J Med Genet*	1972;9:179–192
15	Brekke VG	Clinical Reports. Congenital aortic atresia and hypoplasia of the aortic orifice	*Am Heart J*	1953;45(6):925–930
16	Brenner JI, Berg KA, Schneider DS, Clark EB, Boughman JA	Cardiac malformations in relatives of infants with hypoplastic left-heart syndrome	*Am J Dis Child*	1989;143(12):1492–4
17	Brunson SC, Nudel D, Gootman N, Aftalion B	Letters to the Editor. Truncus arteriosus in a family	*Am Heart J*	1978;96(3):419–420
18	Buch J, Wennevold A, Efsen F, Andersen GE	Interrupted aortic arch in two siblings	*Acta Paediatr Scand*	1980;69:783–785
19	Burman D	Case Report. Familial patent ductus arteriosus	*Br Heart J*	1961;23:603–604
20	Burnell R, Stern L	Five Instances of Persistent Ductus Arteriosus in One Sibship	*Clin Pediatr (Phila)*	1971;10:541–542
21	Campbell M, Polani P	The aetiology of coarctation of the aorta	*Lancet*	1961;(March):463–468
22	Casey B, Cuneo BF, Vitali C, et al	Autosomal dominant transmission of familial laterality defects	*Am J Med Genet*	1996;61(4):325–8
23	Cassidy SC, Allen HD	Tetralogy of Fallot in Triplet Siblings	*Am J Cardiol*	1991;67:1442–1444
24	Cesko I, Hajd J, Toth T, Marton T	Ivemark syndrome with asplenia in siblings	*J Pediatr*	1997;130(5):822–824
25	Cesko I, Hajdú J, Marton T, Tarnai L, Papp Z	Polysplenia and Situs inversus in siblings	*Fetal Diagn Ther*	2001;16:1–3
26	Chapelle A de la, Herva R, Koivisto M, Aula P	A Deletion in Chromosome 22 Can Cause DiGeorge Syndrome	*Hum Genet*	1981;57:253–256
27	Chen S-C, Monteleone PL	Familial splenic anomaly syndrome	*J Pediatr*	1977;91(1):160–161
28	Chen Y, Mao J, Sun Y, et al	A novel mutation of GATA4 in a familial atrial septal defect	*Clin Chim Acta*	2010;411(21-22):1741–5

29	Clementi M, Notari L, Borghi A, Tenconi R	Familial Congenital Bicuspid Aortic Valve : A Disorder of Uncertain Inheritance	*Am J Med Genet*	1996;62:336–338
30	Cousineau A, Lauer R, Pierpont M, et al	Linkage analysis of autosomal dominant atrioventricular canal defects: exclusion of chromosome 21	*Hum Genet*	1994;93:103–108
31	Cripe L, Andelfinger G, Martin LJ, Shooner K, Benson DW	Bicuspid Aortic Valve Is Heritable	*J Am Coll Cardiol*	2004;44(1):138–143
32	Cymbron T, Anjos R, Cabral R, Macedo C, Pereira Duarte C, Mota-Vieira L	Epidemiological characterization of congenital heart disease in São Miguel Island, Azores, Portugal	*Community Genet*	2006;9(2):107–12
33	Czeizel A, Mészáros M	Two family studies of children with ventricular septal defect	*Eur J Pediatr*	1981;136:81–85
34	Davachi F, McLean RH, Moller JH, Edwards JE	Hypoplasia of the right ventricle and tricuspid valve in siblings	*J Pediatr*	1967;71(6):869–874
35	Davidson H	A large family with patent ductus arteriosus and unusual face	*J Med Genet*	1992;30:503–505
36	Davison BC	Concordance and discordance of congenital heart disease in 20 families	*J Med Genet*	1967;4(4):245–250
37	De Meeus A, Sarda P, Tenconi R, Ferrière M, Bouvagnet P	Blastogenesis dominant 1: a sequence with midline anomalies and heterotaxy	*Am J Med Genet*	1997;68(4):405–8
38	Debrus S, Berger G, de Meeus A, et al	Familial non-syndromic conotruncal defects are not associated with a 22qll microdeletion	*Hum Genet*	1996;97:138–144
39	Delatycki MB, Sheffield LJ	Letter to the Editor Familial Heterotaxia : What Is the Inheritance in This Family?	*Am J Med Genet*	1997;69:429–430
40	Dennis N, Warren J	Risks to the offspring of patients with some common congenital heart defects	*J Med Genet*	1981;18(3):8–16
41	Der Kaloustian VM, Ratl H, Malouf J, et al	Tetralogy of Fallot With Pulmonary Atresia in Siblings	*Am J Med Genet*	1985;21:119–122
42	Devriendt K, Casaer A, Cauter A Van, et al	Asplenia syndrome and isolated total anomalous pulmonary venous connection in siblings	*Eur J Pediatr*	1994;153:712–714

43	DiChiara JA, Pieroni DR, Gingell RL, Bannerman RM, Vlad P	Familial pulmonary atresia. Its occurrence with a ventricular septal defect	*Am J Dis Child*	1980;134(2):506–508
44	Digilio MC, Casey B, Toscano A, et al	Complete transposition of the great arteries: Patterns of congenital heart disease in familial precurrence	*Circulation104*	2001;104:2809–2814
45	Digilio MC, Giannotti A, Marino B, Obregon MG, Dallapiccola B	Discrete membranous subaortic stenosis in siblings	*Eur J Pediatr*	1993;152(7):622
46	Digilio MC, Marino B, Canepa SA, Borzaga U, Giannotti A, Dallapiccola B	Congenital Heart Defect in Sibs With Discordant Karyotypes	*Am J Med Genet*	1998;80(February):169–172
47	Digilio MC, Marino B, Cicini MP, Gianotti A, Formigari R, Dallapiccola B	Risk of congenital heart defects in relatives of patients with atrioventricular canal	*Am J Dis Child*	1993;147(12):1295–1297
48	Digilio MC, Marino B, Giannotti A, Dallapiccola B	Familial atrioventricular septal defect: possible genetic mechanism	*Heart*	1994;72(3):301–301
49	Digilio MC, Marino B, Giannotti A, Toscano A, Dallapiccola B	Recurrence risk figures for isolated tetralogy of Fallot after screening for 22q11 microdeletion	*J Med Genet*	1997;34:188–190
50	Digilio MC, Marino B, Gianotti A	Familial Recurrence of Transposition of the Great Arteries and intact ventricular septum	*Am J Med Genet*	1997;73(March):93–94
51	DiGiovanna EL	Family cluster of atrial septal defect	*J Am Osteopath Assoc*	1999;99(12):620–625
52	Disegni E, Pierpont MEM, Bass JL, Kaplinsky E	Two-Dimensional Echocardiography in Detection of Endocardial Cushion Defect in Families	*Am J Cardiol*	1985;55(13):1649–1652
53	Donegan CC, Moore MM, Wiley TM, Hernandez FA, Green JR, Schiebler GL	Familial Ebstein's anomaly of the tricuspid valve	*Am Heart J*	1968;75(3):375–379
54	Ehlers KH, Engle MA	Familial congenital heart disease: I Genetic and environmental factors	*Circulation*	1966;34:503–516
55	Eldadah ZA, Hamosh	Familial Tetralogy of Fallot caused by	*Hum Mol Genet*	2001;10(2):163–169

	A, Biery NJ, et al	mutation in the jagged1 gene		
56	Elliott DA, Kirk EP, Yeoh T, et al	Cardiac Homeobox Gene NKX2-5 Mutations and Congenital Heart Disease	*J Am Coll Cardiol*	2003;41(11):4–8
57	Emanuel R, Brien KO, Somerville J, Jefferson K, Hegde M	Association of secundum atrial septal defect with abnormalities of atrioventricular conduction or left axis deviation Genetic study of 10 families	*Br Heart J*	1975;37:1085–1092
58	Emanuel R, Somerville J, Inns A, Withers R	Evidence of congenital heart disease in the offspring of parents with atrioventricular defects	*Br Heart J*	1983;49:144–147
59	Eronen M, Kajantie E, Boldt T, Pitkänen O, Aittomäki K	Right atrial isomerism in four siblings	*Pediatr Cardiol*	2004;25(2):141–4
60	Fatimi SH, Ahmad U, Javed MA, Shamim S, Ahmad R	Familial membranous subaortic stenosis: Review of familial inheritance patterns an a case report	*J Thorac Cardiovasc Surg*	2006;132(December):1484–1487
61	Ferrero GB, Gebbia M, Pilia G, et al	A submicroscopic deletion in Xq26 associated with familial situs ambiguus	*Am J Hum Genet*	1997;61(2):395–401
62	Fryns J, Van den Berghe H	Clouding, Corneal Stenosis, Subvalvular Aortic Hypoplasia, Midfacial Syndrome, Growth Retardation--a New Syndrome?	*Eur J Pediatr*	1979;131:179–183
63	Furhmann W	Congenital Heart Disease in Sibships Ascertained by two Affected Siblings	*Humangenetik*	1968;6:1–12
64	Gale A, Cartmill T, Bernstein L	Familial subaortic membranous stenosis	*Aust an New Zeal J Med*	1974;4:576–581
65	Gale AN, McKusick VA, Hutchins GM, Gott VL	Familial congenital bicuspid aortic valve	*Chest*	1977;72(5):868–870
66	Gelb BD, Zhang J, Sommer RJ, Wasserman JM, Reitman MJ, Willner JP	Familial Patent Ductus Arteriosus and Bicuspid Aortic Valve With Hand Anomalies : A Novel Heart-Hand Syndrome	*Am J Med Genet*	1999;87:175–179
67	Gelernter-Yaniv L, Lorber A	The familial form of atrial septal defect	*Acta Paediatr*	2007;96(5):726–30
68	Gerboni S, Sabatino G, Mingarelli R,	Coarctation of the aorta, interrupted aortic arch and hypoplastic left heart	*J Med Genet*	1993;30:328–329

	Dallapiccola B	syndrome in three generations		
69	Giustra FX, Tosti, Vincent G	True cor biloculare in identical twins	*Am Heart J*	1938;17:249–250
70	Glancy DL, Wegmann M, Dhurandhar RW	Aortic dissection and patent ductus arteriosus in three generations	*Am J Cardiol*	2001;87:813–815
71	Gleason MM	Concordant total anomalous pulmonary venous connection in dizygotic twins	*Am Heart J*	1989;118(6):1338–1340
72	Glick BN, Roberts WC	Congenitally Bicuspid Aortic Valve in Multiple Family Members	*Am J Cardiol*	1994;73:400–404
73	Gobel JW, Pierpont MEM, Moller JH, Singh A, Edwards JE	Familial interruption of the aortic arch	*Pediatr Cardiol*	1993;14:110–115
74	Godden D, Sandhu P, Kerr F	Stenosed bicuspid aortic valves in twins	*Eur Heart J*	1987;8(3):316–318
75	Goodyear JE	Persistent truncus arteriosus in two siblings	*Heart*	1961;23:194–197
76	Grant JW	Congenital malformations of the tricuspid valve in siblings	*Pediatr Cardiol*	1996;17:327–329
77	Grobman W, Pergament E	Isolated hypoplastic left heart syndrome in three siblings	*Obstet Gynecol*	1996;88(4):673–675
78	Gueron M, Hirsch M, Stern J, Cohen W, Levy M	Familial Ebstein's Anomaly with Emphasis on the Surgical Treatment	*Am J Cardiol*	1966;18(July):105–111
79	Gunal N, Gül S, Kahramnyol Ö	Familial atrial septal defect with prolonged atrioventricular conduction	*Acta Paediatr Jpn Overseas Ed*	1997;39(February):634–636
80	Hinton RB Jr, Martin LJ, Tabangin ME et al.	Hypoplastic left heart syndrome is heritable.	*J Am Coll Cardiol.*	2007; 50(16):1590-5
81	Hirayama-Yamada K, Kamisago M, Akimoto K, et al	Phenotypes with GATA4 or NKX2.5 mutations in familial atrial septal defect	*Am J Med Genet A*	2005;135(1):47–52
82	Houyel L, Khoshnood B, Anderson RH, et al	Population-based evaluation of a suggested anatomic and clinical classification of congenital heart defects based on the International Paediatric and Congenital Cardiac Code	*Orphanet J Rare Dis*	2011;6(1):64

83	Huntington K, Hunter AGW, Chan K	A Prospective Study to Assess the Frequency of Familial Clustering of Congenital Bicuspid Aortic Valve	*JACC*	1997;30(7):1809–1812
84	Hurwitz RC, Caskey T	Ivemark syndrome in siblings	*Clin Genet*	1982;22:7–11
85	Joyce JC, O'Toole SP	Congenital heart disease. Report of an unusually high incidence in one family	*Br Med J*	1954;1(4873):1241–1242
86	Katcher AL	Familial Asplenia, Other Malformations, and Sudden Death	*Pediatrics*	1980;65(3):633–635
87	Kawashima H, Ohno I, Ueno Y, Nakaya S, Kato E, Taniguchi N	Syndrome of microtia and aortic arch anomalies resembling isotretinoin embryopathy	*J Pediatr*	1987;111(5):738–740
88	Keller HI, Cheitlin MD	The occurrence of mild coarctation of the aorta (pseudocoarctation) and coarctation in one family	*Am Heart J*	1965;70(1):115–118
89	Khau Van Kien P, Wolf J-E, Mathieu F, et al	Familial thoracic aortic aneurysm/dissection with patent ductus arteriosus: genetic arguments for a particular pathophysiological entity	*Eur J Hum Genet*	2004;12(October 2003):173–180
90	Klinge T, Baekgaard Laursen H	Familial pulmonary stenosis with underdeveloped or normal right ventricle	*Br Heart J*	1975;37:60–64
91	Kodo K, Nishizawa T, Furutani M, et al	GATA6 mutations cause human cardiac outflow tract defects by disrupting semaphorin-plexin signaling	*PNAS*	2009;106(33):13933–13938
92	Kumar A, Victorica BE, Gessner IH, Alexander JA	Tricuspid atresia an annular hypoplasia: Report of a familial occurrence	*Pediatr Cardiol*	1994;15:201–203
93	Kumar A, Williams CA, Victorica BE	Familial atrioventricular septal defect: possible genetic mechanisms	*Br Heart J*	1994;71:79–81
94	Kwiatkowska J, Wierzba J, Aleszewicz-Baranowska J, Ereciński J	Genetic background of congenital conotruncal heart defects--a study of 45 families	*Kardiol Pol*	2007;65(1):32–37; discussion 38–39
95	Lang MJ, Aughton DJ, Riggs TW, Milad MP, Biesecker LG	Dizygotic twins concordant for truncus arteriosus	*Clin Genet*	1991;39(1):75–9
96	Lo KS, Loventhal JP, Walton jr JA	Familial Ebstein's anomaly	*Cardiology*	1979;64:246–255

97	Luca A De, Sarkozy A, Consoli F, et al	Familial transposition of the great arteries caused by multiple mutations in laterality genes	*Heart*	2010;96:673–678
98	Lynch H, Grissom R, Magnuson C, Krush A	Patent ductus arteriosus. Study of two families	*JAMA*	1965;194:135–138
99	Lynch HT, Bachenberg K, Harris RE, Becker W	Hereditary atrial septal defect. Update of a large kindred	*Am J Dis Child*	1978;132(6):600–4
100	Lynch HT, Tips RL, Krush AJ	Tetralogy of Fallot in two siblings	*Am J Dis Child*	1966;11:304–307
101	Maron BJ, Borer JS, Lau SH, Damato AN, Scott LP, Epstein SE	Association of secundum atrial septal defect and atrioventricular nodal dysfunction A genetically transmitted syndrome	*Br Heart J*	1978;40(December 1977):1293–1299
102	Martin R, Banner N, Radley-Smith R	Familial persistent ductus arteriosus	*Arch Dis Child*	1986;61:906–907
103	Mathias RS, Lacro R V, Jones KL	X-linked laterality sequence: situs inversus, complex cardiac defects, splenic defects	*Am J Med Genet*	1987;28(1):111–6
104	McBride KL, Zender GA, Fitzgerald-Butt SM, et al	Linkage analysis of left ventricular outflow tract malformations (aortic valve stenosis , coarctation of the aorta, and hypoplastic left heart syndrome)	*Eur J Hum Genet*	2009;17(6):811–819
105	McDonald K, Maurer B	Familial aortic valve disease: evidence for a genetic influence?	*Eur Heart J*	1989;10(7):676–677
106	McIntosh N, Chitayat D, Bardanis M, Fouron J-C	Ebstein Anomaly : Report of a Familial Occurrence and Prenatal Diagnosis	*Am J Med Genet*	1992;42:307–309
107	McKusick VA	Association of congenital bicuspid aortic valve and Erdheim's cystic medial necrosis	*Lancet*	1972;6(1):1026–1027
108	Medd WE, Neufeld HN, Weidman WH, Edwards JE	Isolated hypoplasia of the right ventricle and tricuspid valve in siblings	*Br Heart J*	1961;23:25–31
109	Mégarbané A, Stephan E, Kassab R, et al	Autosomal Dominant Secundum Atrial Septel Defect With Various Cardiac and Noncardiac Defects : A New	*Am J Med Genet*	1999;83:193–200

		Midline Disorder		
110	Menahem S	Familial aggregation of defects of the left-sided structures of the heart	*Int J Cardiol*	1990;29:239–240
111	Mikkilä SP, Janas M, Karikoski R, Tarkkila T, Simola KO	X-linked laterality sequence in a family with carrier manifestations	*Am J Med Genet*	1994;49(4):435–8
112	Miller ME, Smith DW	Conotruncal malformation complex: examples of possible monogenic inheritance	*Pediatrics*	1979;63(6):890–893
113	Milner S, Levin SE, Marchand PE, Hitchcock F	Total anomalous pulmonary venous drainage in sibs	*Arch Dis Child*	1977;52(12):984
114	Monte SM De, Hutchins GM	Brief Clinical Report : Sisters With Polysplenia	*Am J Med Genet*	1985;21:171–173
115	Morelli SH, Young L, Reid B, Ruttenberg H, Bamshad MJ	Clinical Analysis of Families With Heart, Midline and Laterality Defects	*Am J Med Genet*	2001;101(November 2000):388–392
116	Moss AJ	Coarctation of the aorta in siblings	*J Pediatr*	1955;46(6):707–709
117	Mu T, McAdams R, Bush D	A Case of Hypoplastic Left Heart Syndrome and Bicuspid Aortic Valve in Monochorionic Twins	*Pediatr Cardiol*	2005;26:884–885
118	Nakada T, Yonesaka S	Interruption of aortic arch type A in two siblings	*Acta Paediatr Jpn Overseas Ed*	1996;38(1):63–65
119	Neufeld HN, Ongley PA, Swan H, Burgert EO, Edwards JE	Biventricular origin of the pulmonary trunk with subaortic stenosis above the ventricular septal defect	*Am Heart J*	1961;February:189+198
120	Nicolae MI, Summers KM, Radford DJ	Familial muscular ventricular septal defects and aneurysms of the muscular interventricular septum	*Cardiol Young*	2007;17(5):523–7
121	Niikawa N, Kohsaka S, Mizumoto M, Hamada I, Kajii T	Familial clustering of situs inversus totalis, and asplenia and polysplenia syndromes	*Am J Med Genet*	1983;16(1):43–7
122	O'Noullain S, Hall JG, Stamm SJ	Autosomal dominant inheritance of endocardial cushion defect	*Birth defects*	1977:143–147
123	Okubo A, Miyoshi O, Baba K, et al	A novel GATA4 mutation completely segregated with atrial septal defect in a large Japanese family	*J Med Genet*	2004;41(7):e97–e97

124	Onat A, Onat T, Domaniç N	Discrete subaortic stenosis as part of a short stature syndrome	*Hum Genet*	1984;65:331–335
125	Pankau R, Funda J, Wessel A	Interrupted Aortic Arch Type B1 in a Brother and Sister : Suggestion of a Recessive Gene	*Am J Med Genet*	1990;36:175–177
126	Paz JE, Castilla EE	Familial Total Anomalous Pulmonary Venous Return	*J Med Genet*	1971;8(June 1968):312–315
127	Petsas AA, Anastassiades LC, Constantinou EC, Antonopoulos AG	Familial discrete subaortic stenosis	*Clin Cardiol*	1998;21:63–65
128	Pierard LA, Henrard L, Demoulin J-C	Persistent atrial standstill in familial Ebstein's anomaly	*Br Heart J*	1985;53(November 1984):594–597
129	Polani P, Campbell M	Factors in the causation of persisten ductus arteriosus	*Ann Hum Genet*	1959;60(24):343–357
130	Raatikka M, Rapola J, Tuuteri L, Louhimo I, Savilahti E	Familial third and fourth pharyngeal pouch syndrome with truncus arteriosus: DiGeorge Syndrome	*Pediatrics*	1981;67(2):173–175
131	Raisher BD, Dowton SB, James W	Father and Two Children With Total Anomalous Pulmonary Venous Connection	*Am J Med Genet*	1991;40:105–106
132	Record RG, Mckeown T	Observations relating to the aetiology of patent ductus arteriosus	*Br Heart J*	1953;15:376–387
133	Rein AJ	Genetics of Conotruncal Malformations : Further Evidence of Autosomal Recessive Inheritance	*Am J Med Genet*	1994;50:302–303
134	Richardson ME, Menahem S, Wilkinson JL	Familial fixed subaortic stenosis	*Int J Cardiol*	1991;30(November):351–353
135	Rogers JC, Begleiter ML, Harris DJ	Patent ductus arteriosus in four generations of a family	*J Med Genet*	1992;29:758
136	Rohn RD, Leffeli MS, Leadem P, Johnson D, Rubio T, Emanuel BS	Familial third-fourth pharyngeal pouch syndrome with apparent autosomal dominant transmission	*J Pediatr*	1984;105(1):47–51
137	Rosenmann A, Arad I, Simcha A, Schaap T	Familial Ebstein ' s anomaly	*J Med Genet*	1976;13(6):532–535
138	Ruggieri M, Abbate M, Parano E, Distefano A,	Scimitar vein anomaly with multiple cardiac malformations, craniofacial,	*Am J Med Genet A*	2003;116A(2):170–5

	Guarnera S, Pavone L	and central nervous system abnormalities in a brother and sister: familial scimitar anomaly or new syndrome?		
139	Ruttenberg HD, Neufeld HN, Lucas R V, et al	Syndrome of congenital cardiac disease with Asplenia. Distinction from other forms of congenital cyanotic cardiac disease	*Am J Cardiol*	1964;13:387–406
140	Sackner M, Robinson M, Jamison W, Lewis D	Isolated right ventricular hypoplasia with atrial septal defect of patent foramen ovale	*Circulation*	1961;24:1388–1402
141	Schönfeld E, Frischman B	Syndrome of spleen agenesis, defects of the heart and vessels and situs inversus. Report of a case suggesting heredity as an etiological factor	*Helv Paediatr Acta*	1958;13:636–640
142	Schunkert H, Bröckel U, Kromer EP, Elsner D, Jacob HJ, Riegger GA	A Large Pedigree With Valvuloseptal Defects	*Am J Cardiol*	1997;1(80):968–970
143	Sehested J	Coarctation of the aorta in monozygotic twins	*Br Heart J*	1982;47:619–620
144	Seides SF, Shemin RJ, Morrow AG	Congenital cardiac abnormalities in monozygotic twins Report and review of the literature	*Br Heart J*	1979;42:742–745
145	Shapiro SR, Ruckman RN, Kapur S, et al	Single ventricle with truncus arteriosus in siblings	*Am Heart J*	1981;3(1):456–459
146	Shokeir MH	Hypoplastic left heart syndrome: an autosomal recessive disorder	*Clin Genet*	1971;2(1):7–14
147	Shokeir MH	Hypoplastic left heart. Evidence for possible autosomal recessive inheritance	*Birth Defects Orig Artic Ser*	1974;10(4):228–30
148	Silver W, Steier M, Chandra N	Asplenia Syndrome with Congenital Heart Disease and Tetralogy of Fallot in Siblings	*Am J Cardiol*	1972;30(4):91–94
149	Simon AB, Zloto AE, Perry BL, Sigmann JM	Familial Aspects of Coarctation of the Aorta	*Chest*	1974;66(6):887–889
150	Simpson J, Zellweger H	Familial Occurrence of Ivemark Syndrome with Splenic Hypoplasia	*J Med Genet*	1973;10(1963):102–103

		and Asplenia in Sibs		
151	Skelton R, Coles JC	Familial homogeneity of congenital malformations of the heart: Report of atrial septal defect occurring in two sisters	*Can Med Assoc J*	1958;79(11):910–912
152	Slavotinek A, Clayton-smith J, Super M	Familial Patent Ductus Arteriosus : A Further Case of CHAR Syndrome	*Am J Med Genet*	1997;71:229–232
153	Sletten LJ, Pierpont MEM	Familial Occurrence of Patent Ductus Arteriosus	*Am J Med Genet*	1995;57:27–30
154	Soltan HC, Li MD	Hereditary dextrocardia associated with other congenital heart defects: report of a pedigree	*Clin Genet*	1974;5(1):51–8
155	Solymar L, Sabel KG, Zetterqvist P	Total anomalous pulmonary venous connection in siblings. Report on three families	*Acta Paediatr Scand*	1987;76(1):124–7
156	Taylor R, Pollock B	Clinical Reports. Coarctation of the aorta in three members of a family	*Am Heart J*	1953;45(3):470–475
157	Thammineni K, Lohr J, Trefz M, Sivanandam S	Familial recurrence of congenital heart diseases	*J Perinatol*	2011;31(March):742–743
158	Toriello H V, Kokx N, Higgins J V, Hofman R, Waterman DF	Sibs with the polyasplenia developmental field defect	*Am J Med Genet Suppl*	1986;2:31–6
159	Udwadia A, Khambadkone S, Bharucha B, Lokhandwala Y, Irani S	Familial congenital valvar pulmonary stenosis: autosomal dominant inheritance	*Pediatr Cardiol*	1996;17:407–409
160	Urbach J, Glaser J, Balkin J, et al	Familial membranous subaortic stenosis	*Cardiology*	1985;72:214–217
161	Uyan C, Yazici M, Uyan AP, Akdemir R	Ebstein's anomaly in siblings : an original observation	*Int J Cardiovasc Imaging*	2002;18:435–438
162	Van de Meerakker JB a, van Engelen K, Mathijssen IB, et al	A novel autosomal dominant condition consisting of congenital heart defects and low atrial rhythm maps to chromosome 9q	*Eur J Hum Genet*	2011;19(7):820–6
163	Vergara P, Digilio MC, Limongelli G, et al	Familial Recurrence of Anomalous Origin of Right Pulmonary Artery From the Aorta	*Am J Med Genet*	2006;140A:794–796

164	Vinh LT, Van Duc T, Aicardi J, Theiffrey S	Retour veineux pulmonaire anormal total infradiaphragmatique	*Arch Franç Ped*	1968;25:1141–1149
165	Wang J, Xin Y-F, Liu X-Y, Liu Z-M, Wang X-Z, Yang Y-Q	A novel NKX2-5 mutation in familial ventricular septal defect	*Int J Mol Med*	2011;27:369–375
166	Wang X, Wang J, Zhao P, et al	Familial congenital heart disease: data collection and preliminary analysis	*Cardiol Young*	2013;23(3):394–9
167	Wei J, Chang Y-C, Ko G-C, Shieh S-M	Familial patent ductus arteriosus	*Am J Cardiol*	1984;54:235–236
168	Weigel TJ, Driscoll DJ, Michels V V	Occurrence of Congenital Heart Defects in Siblings of Patients with Univentricular Heart and Tricuspid Atresia	*Am J Cardiol*	1989;64:768–771
169	Weil MH, Allenstein BJ	Familial occurrence of defects of the interatrial septum	*Circulation*	1959;20:782–783
170	Weinstein A	Congenital heart disease in successive generations. Interatrial septal defect in a 63-year-old and her 31-year-old son	*J Chronic Dis*	1958;8(6):669–677
171	Williamson EM	A Family Study of Atrial Septal Defect	*J Med Genet*	1969;6:255–265
172	Wilmshurst P, Panikkar J, Pearson M, Nightingale S	Relation Between Inheritance of Cyanotic Congenital Heart Disease and Persistent Foramen Ovale	*AJC*	2009;104(1):148–149
173	Wilmshurst PT, Pearson MJ, Nightingale S, Walsh KP, Morrison WL	Inheritance of persistent foramen ovale and atrial septal defects and the relation to familial migraine with aura	*Heart*	2004;90(11):1315–20
174	Wilson D, Cross I, Goodship J, et al	DiGeorge syndrome with isolated aortic coarctation and isolated ventricular septal defect in three sibs with a 22ql 1 deletion of maternal origin	*Br Heart J*	1991;66:308–313
175	Wilson D, Goodship J, Burn J, Cross I, Scambler P	Deletions within chromosome 22q11 in familial congenital heart disease	*Lancet*	1992;340:573–575
176	Wilson L, Curtis A, Korenberg J, et al	A Large , Dominant Pedigree of Atrioventricular Septal Defect (AVSD): Exclusion from the Down Syndrome Critical Region on	*Am J Hum Genet*	1993;53:1262–1268

		Chromosome 21		
177	Woods CG, Sheffield LJ	Further family with autosomal dominant patent ductus arteriosus	*J Med Genet*	1994;31:659
178	Wulfsberg EA, Zintz EJ, Moore JW	The inheritance of conotruncal malformations: a review and report of two siblings with tetralogy of Fallot with pulmonary atresia	*Clin Genet*	1991;40(1):12–6
179	Yang Y-Q, Li L, Wang J, et al	A novel GATA4 loss-of-function mutation associated with congenital ventricular septal defect	*Pediatr Cardiol*	2012;33(4):539–46
180	Yang Y-Q, Wang J, Liu X-Y, et al	Novel GATA4 mutations in patients with congenital ventricular septal defects	*Med Sci Monit*	2012;18(6):344–350
181	Yao JK, Thompson MW, Trusler GA, Trimble AS	Familial Atrial Septal Defect of the Primum Type: A Report of Four Cases in One Sibship Type	*Can Med Assoc J*	1968;98:218–219
182	Zellers TM, Driscoll DJ, Michels V V	Prevalence of significant congenital heart defects in children of parents with Fallot's Tetralogy	*Am J Cardiol*	1990;65:523–526
183	Zhang W, Li X, Shen A, Jiao W, Guan X, Li Z	GATA4 mutations in 486 Chinese patients with congenital heart disease	*Eur J Med Genet*	2008;51(6):527–35
184	Zheng G-F, Wei D, Zhao H, Zhou N, Yang Y-Q, Liu X-Y	A novel GATA6 mutation associated with congenital ventricular septal defect	*Int J Mol Med*	2012;29:1065–1071
185	Zlotogora J, Elian E	Asplenia and polysplenia syndromes with abnormalities of lateralisation in a sibship	*J Med Genet*	1981;18(4):301–302
186	Zlotogora J, Schimmel MS, Glaser Y	Familial situs inversus and congenital heart defects	*Am J Med Genet*	1987;26(1):181–4
187	Zuckerman HS, Zuckerman GH, Mammen RE, Wassermil M	Atrial septal defect. Familial occurrence in four generations of one family	*Am J Cardiol*	1962;9(4):515–520

Supplemental Table 2. Diagnostic terms, abbreviations, severity and total number of cases

Malformation	IPCC code	Abbreviation	Severity	No. of individuals (No. of families)
Abnormal Aortic Branch*	09.30.00	AbAB	2	12 (9)
Abnormal Coronary Artery	09.46.03	AbCA	2	1 (1)
Abnormal Mitral Valve	06.02.11	AbMV	1	12 (8)
Abnormal Pulmonary Valve	09.05.29	AbPV	1	2 (2)
Abnormal Tricuspid Valve	06.01.11	AbTV	1	8 (8)
Abnormal Veins	04.05.00	AbV	2	4 (3)
Aorta Hypoplasia	09.16.03	AoHy	1	29 (26)
Aortic Valve Atresia	09.15.06	AVA	2	8 (7)
Aortic Valve Regurgitation	09.15.07	AVR	1	10 (7)
Aortic Valve Stenosis	09.15.01	AVS	1	169 (120)
Aortopulmonary Window	09.04.01	APWin	2	2 (2)
ASD, sinus venosus type	05.05.00	ASDsv	2	9 (7)
Atrial Septal Defect, secundum type	05.04.02	ASD	1	685 (348)
Atrioventricular Septal Defect	06.06.09	AVSD	3	128 (84)
Atrioventricular Valve malformation	06.05.01	AVmal	1	5 (4)
Bicuspid Aortic Valve	09.15.22	BAV	2	387 (225)
Cleft Mitral Valve (anterior)	06.02.36	CMV	1	45 (25)
Coarctation of Aorta	09.29.01	CoA	2	259 (190)
Common Arterial Trunk (Truncus Arteriosus)	09.01.01	TA	3	69 (48)
Congenital Aortic Aneurysm	07.09.30	CAA	1	1 (1)
Congenitally Corrected Transposition of The Great Arteries	01.01.03	CCTGA	3	29 (26)
Dextrocardia	02.01.02	Dxc	1	58 (43)

Double Aortic Arch	09.28.09	DAA	2	2 (1)
Double Inlet Right Ventricle	01.04.03	DIRV	3	1 (1)
Double Outlet Right Ventricle	01.01.04	DORV	2	33 (28)
Ebstein's Anomaly	06.01.34	EbA	2	38 (21)
Heart Septation Anomaly†	05.06.01	HSA	2	38 (32)
Hypoplastic Left Heart Syndrome	01.01.09	HLHS	1	179 (141)
Hypoplastic Right Heart Syndrome	07.02.10	HRHS	1	21 (16)
Infundibular Pulmonary Stenosis	07.17.05	infPS	1	31 (28)
Interrupted Aortic Arch	09.29.31	IAA	2	34 (26)
Isomerism	01.03.08	Iso	3	17 (14)
Mitral Valve Atresia	06.02.02	MVA	3	11 (10)
Mitral Valve Regurgitation	06.02.25	MVR	1	54 (46)
Mitral Valve Stenosis	06.02.07	MVS	1	14 (12)
Partial Anomalous Pulmonary Venous Return	04.07.01	PAPVR	2	26 (22)
Patent Ductus Arteriosus	09.27.21	PDA	1	252 (146)
Patent Foramen Ovale	05.03.01	PFO	1	100 (59)
Patent Left Superior Vena Cava	04.01.31	PLSVC	1	46 (39)
Pulmonary Artery Atresia	09.10.21	PAA	2	23 (20)
Pulmonary Artery Hypoplasia	09.10.11	PAH	1	15 (12)
Pulmonary Artery Stenosis	09.10.01	PAS	1	31 (21)
Pulmonary Valve Atresia	09.05.11	PVA	1	70 (57)
Pulmonary Valve Stenosis	09.05.04	PVS	1	208 (139)
Right Aortic Arch	09.28.15	RAA	1	38 (32)
Single Ventricle	01.01.22	SV	3	60 (50)
Situs Ambiguus	01.01.71	SA	3	18 (15)

Situs Inversus	03.01.03	SI	3	37 (29)
Subvalvular Aortic Stenosis	07.09.00	subAS	2	44 (24)
Supravalvular Aortic Stenosis	09.16.18	supAS	2	17 (7)
Tetralogy of Fallot	01.01.01	TOF	2	207 (142)
Total Anomalous Pulmonary Venous Return	04.08.05	TAPVR	2	80 (47)
Transposition of The Great Arteries‡	01.01.02	TGA	3	148 (111)
Tricuspid Valve Atresia	06.01.01	TVA	3	12 (11)
Tricuspid Valve Regurgitation	06.01.25	TVR	1	3 (3)
Tricuspid Valve Stenosis	06.01.07	TVS	1	2 (2)
Vascular Ring	09.31.00	Vring	2	3 (3)
Vena Cava Abnormality§	04.05.16	VCAb	2	16 (15)
Ventricular Septal Defect‖	07.15.05	VSD	2	587 (402)

* Includes sinus valsalva aneurysm, abnormal left subclavian artery. † Includes common atrium, cor biloculare, cor triloculare. ‡ The combination of concordant atrioventricular and discordant ventriculo-arterial connections. § Includes patent left inferior vena cava, vena azygos continuation of superior vena cava, interrupted inferior vena cava. ‖ Includes both muscular and membranous types. # A severity score between 1 and 3 was assigned to each malformation (1=low severity, 2=intermediate severity, 3=high severity).

Supplemental Table 3. Non-validated individuals per family. The number of individuals with unverified CHD diagnose per family in the unpublished dataset is shown. These individuals were excluded from our data analysis. The median number of non-validated individuals per family is 2.

Family name	PMID/reference	Family size	No. of affected individuals	No. of non-validated individuals
McBride_156_a	19142209	4	2	0
McBride_1568	19142209	6	2	0
McBride_1574	19142209	5	2	0
McBride_1646	19142209	7	2	0
McBride_1685	19142209	4	2	0
McBride_1705	19142209	4	2	0
McBride_90	19142209	5	2	0
McBride_219	19142209	5	2	0
McBride_234	19142209	7	2	1
McBride_238	19142209	6	2	0
McBride_242	19142209	7	2	0
McBride_258	19142209	5	2	0
McBride_974	19142209	8	3	0
McBride_80	19142209	16	6	0
McBride_648	19142209	18	5	0
McBride_268	19142209	8	2	0
McBride_304	19142209	5	2	0
McBride_321	19142209	5	2	0
McBride_324	19142209	8	3	0
McBride_329	19142209	8	2	0
McBride_341	19142209	11	4	0
McBride_344	19142209	6	2	1
McBride_471	19142209	5	2	0
McBride_523	19142209	7	3	0
McBride_546	19142209	4	2	0
McBride_55007	19142209	10	3	0
McBride_564	19142209	6	3	0
McBride_614	19142209	6	2	0
McBride_630	19142209	6	2	0
McBride_1022	19142209	7	3	0
McBride_1154	19142209	5	2	0
McBride_1206	19142209	4	2	0
McBride_1254	19142209	12	2	0
McBride_1298	19142209	12	2	0
McBride_977	19142209	4	2	0
McBride_134	19142209	7	2	0
McBride_137	19142209	10	2	0

McBride_1374	19142209	4	2	0
McBride_1426	19142209	7	2	0
McBride_1541	19142209	8	2	0
Gale_1977	913155	4	2	0
Glick_II	8109558	9	2	0
Glick_III	8109558	5	2	1
Glick_IV	8109558	7	2	3
Glick_V	8109558	15	2	0
Glick_VI	8109558	9	2	1
Schonfeld_1958	13640457	7	2	0
Seides_1979	575295	11	2	0
Kodo_2009	19666519	6	3	0
Wang_1	23021226	5	2	0
Wang_2	23021226	5	2	0
Wang_3	23021226	5	2	0
Wang_4	23021226	5	2	0
Wang_5	23021226	5	2	0
Wang_6	23021226	5	2	0
Wang_7	23021226	5	2	0
Wang_8	23021226	5	2	0
Wang_9	23021226	5	2	0
Wang_10	23021226	5	2	0
Wang_11	23021226	5	2	0
Wang_12	23021226	5	2	0
Wang_13	23021226	5	2	0
Wang_15	23021226	4	2	1
Wang_16	23021226	4	2	0
Wang_17	23021226	4	2	0
Wang_19	23021226	4	2	0
Wang_20	23021226	4	2	1
Wang_21	23021226	4	2	0
Yang_1	22648249	5	2	0
Yang_2	22648249	7	3	0
Yang_3	22648249	6	2	0
Zheng_2012	22407241	10	5	0
Yang_2012_2	22101736	17	7	2
Zhang_2008	18672102	5	2	1
Wang_2011	21165553	9	5	0
Cymbron_2006	16612061	7	4	0
Nicolae_2007	17637070	23	5	6
Czeizel_1	7215392	5	2	0
Czeizel_2	7215392	5	2	0

Czeizel_3	7215392	4	2	0
Czeizel_4	7215392	4	2	0
Czeizel_5	7215392	4	2	0
Czeizel_6	7215392	4	2	0
Czeizel_7	7215392	4	2	0
Czeizel_8	7215392	4	2	0
Czeizel_9	7215392	4	2	0
Czeizel_10	7215392	4	2	0
Solymar_1	3564987	8	3	0
Solymar_2	3564987	7	2	0
Solymar_3	3564987	6	2	0
Gleason_1989	2686385	4	2	0
Baron_1982	7137007	4	2	0
Ferrero_1997	9311745	4	2	0
Milner_1	564667	6	2	0
Ruggieri_2003	12494437	7	2	0
Ashida_2001	11425786	4	2	0
Bleyl_A	7747759	22	3	5
Bleyl_B	7747759	21	3	0
Bleyl_C	7747759	20	3	0
Paz_1971	5097138	9	3	0
Devriendt_1994	7813525	6	2	0
Raisher_1991	1887837	6	3	0
Vergara_2006_B	16523515	6	2	0
Dennis_8	7253006	6	3	0
Dennis_16	7253006	5	2	0
Dennis_29	7253006	5	2	0
Dennis_32	7253006	6	2	0
Dennis_70	7253006	6	2	0
Dennis_105	7253006	5	2	1
Dennis_185	7253006	4	2	0
Dennis_215	7253006	4	2	0
Dennis_240	7253006	5	2	0
Dennis_254	7253006	4	2	0
Dennis_261	7253006	5	2	0
Dennis_276	7253006	4	2	0
Dennis_284	7253006	4	2	0
Dennis_286	7253006	5	2	0
Dennis_426	7253006	5	2	0
Weigel_1	2801528	4	2	0
Weigel_2	2801528	4	2	0
Weigel_3	2801528	4	2	0

Weigel_4	2801528	4	2	0
Weigel_5	2801528	4	2	0
Weigel_6	2801528	4	2	0
Weigel_7	2801528	5	3	0
Shapiro_1981	6455913	7	3	0
Bonnet_A	10227411	7	2	0
Bonnet_B	10227411	8	2	0
Bonnet_C	10227411	8	2	0
Bonnet_D	10227411	6	2	0
Bonnet_E	10227411	4	2	0
Davachi_1967	6070007	4	2	0
Medd_1960	13768823	4	2	0
Sackner_1961	14495868	7	2	1
Pierard_1985	4005080	5	2	0
McIntosh_1992	1536167	4	2	0
Kumar_1994_b	7991439	5	3	0
Grant_1996	8660450	4	2	0
Balaji_1991	1854572	11	6	0
Gueron_1966	5938901	9	2	0
Rosenmann_1	1018315	6	3	1
Donegan_1968	5638476	16	2	0
Lo_1979	476731	4	2	0
Uyan_2002	12537411	4	2	0
Disegni_1	4003317	12	3	0
Disegni_2	4003317	8	2	1
Disegni_3	4003317	13	4	2
Disegni_4	4003317	38	3	0
Yao_1968	20329145	12	4	0
Gelernter-Yaniv_1	17462063	5	2	1
Gelernter-Yaniv_2	17462063	6	3	0
Gelernter-Yaniv_3	17462063	6	3	0
Gelernter-Yaniv_4	17462063	4	2	0
Gelernter-Yaniv_5	17462063	4	2	0
Gelernter-Yaniv_6	17462063	8	2	2
Schunkert_1997	9382022	19	7	4
Amati_1	7677156	16	6	0
Amati_2	7677156	28	7	0
Emanuel_1	6824534	5	2	0
Emanuel_2	6824534	5	2	0
Emanuel_3	6824534	5	2	0
Emanuel_4	6824534	5	2	0
Digilio_1993_1	8249947	16	6	0

Digilio_1993_2	8249947	5	2	0
Digilio_1993_3	8249947	7	2	0
Digilio_1993_4	8249947	4	2	1
Digilio_1993_5	8249947	28	6	0
Cousineau_1994	8112730	52	12	0
Wilson_1993	8250042	51	9	2
ONoullain_1977	884239	23	5	7
Digilio_1998_1	9805136	4	2	0
Digilio_1998_2	9805136	4	2	0
Digilio_1998_3	9805136	5	2	0
Digilio_1994	7946792	9	4	0
Kumar_1994_a	8297702	14	3	0
Weinstein_1958	13598778	6	2	0
Weil_1959	Circulation: Proceedings of the 32nd Scientific Session pg 782	7	4	0
Zuckerman_1962	American Jounal of Cardiology; 1962;9;4;515-520	24	7	1
Skelton_1958	13608370	4	2	0
Williamson_15	5345096	8	2	0
Williamson_16	5345096	6	2	0
Williamson_17	5345096	10	6	1
Williamson_34	5345096	5	2	0
Williamson_47	5345096	8	2	0
Williamson_54	5345096	7	2	0
Williamson_71	5345096	5	2	0
Williamson_83	5345096	15	3	0
Williamson_87	5345096	6	2	0
Williamson_88	5345096	7	2	0
Williamson_111	5345096	10	2	0
Williamson_117	5345096	7	2	0
Williamson_121	5345096	8	2	0
Maron_1978	718771	9	4	4
Megarbane_1999	10096596	25	13	1
Gunal_1997	9363669	6	2	3
Bizarro_1970	5437412	39	15	1
Wilmshurst_2004_1	15486131	53	17	3
Wilmshurst_2004_2	15486131	8	4	0
Wilmshurst_2004_3	15486131	13	6	0
Wilmshurst_2004_4	15486131	9	4	0
Wilmshurst_2004_5	15486131	7	3	0
Wilmshurst_2004_6	15486131	8	2	0
Wilmshurst_2009	15486131	22	9	0
Chen_2010_1	20659440	8	4	0

Benson_1998_A	9610535	32	11	1
Benson_1998_B	9610535	27	10	0
Benson_1998_C	9610535	27	11	2
Emanuel_1975_A	1191421	7	2	2
Emanuel_1975_B	1191421	6	3	0
Emanuel_1975_C	1191421	11	3	0
Hirayama-Yamada_1	15810002	15	7	0
Hirayama-Yamada_2	15810002	6	3	0
Hirayama-Yamada_3	15810002	6	4	0
Hirayama-Yamada_4	15810002	14	2	0
Hirayama-Yamada_5	15810002	13	6	1
DiGiovanna_1999	10641493	36	11	0
Elliott_1024	12798584	8	3	0
Elliott_AF1	12798584	10	4	0
Chen_2010_2	20659440	20	8	0
Ehlers_1	5922714	7	2	1
Ehlers_2	5922714	7	3	0
Ehlers_3	5922714	8	2	1
Ehlers_4	5922714	6	2	0
Ehlers_5	5922714	8	2	0
Ehlers_6	5922714	16	2	1
Ehlers_7	5922714	15	2	0
Ehlers_8	5922714	12	2	0
Ehlers_9	5922714	8	2	0
Ehlers_10	5922714	7	2	0
Ehlers_11	5922714	7	2	0
Ehlers_12	5922714	7	2	0
Ehlers_13	5922714	5	2	0
Ehlers_14	5922714	6	2	0
Ehlers_15	5922714	8	3	0
Ehlers_17	5922714	10	3	0
Ehlers_18	5922714	7	2	0
Ehlers_19	5922714	19	2	0
Ehlers_20	5922714	12	2	0
Ehlers_21	5922714	7	2	0
Ehlers_22	5922714	21	2	1
Ehlers_23	5922714	10	2	0
Ehlers_24	5922714	22	2	0
Ehlers_25	5922714	19	2	0
Ehlers_26	5922714	15	2	0
Ehlers_27	5922714	32	2	0
Ehlers_29	5922714	14	2	0

Ehlers_30	5922714	9	2	0
Ehlers_31	5922714	8	2	0
Ehlers_32	5922714	16	2	1
Miller_1	450526	10	2	3
Miller_2	450526	5	3	0
Wulfsberg_1991	1884513	5	2	0
Wilson_1992_1	1355155	6	2	0
Wilson_1992_2	1355155	7	3	0
Wilson_1992_4	1355155	8	4	0
Wilson_1992_5	1355155	7	3	0
Eldadah_2001	11152664	25	12	3
Zellers_1990_1	2305694	5	2	0
Zellers_1990_2	2305694	5	2	0
Adams_1974	15215987	4	2	0
Boon_1972_1	5065286	7	2	3
Boon_1972_2	5065286	26	3	0
Digilio_1997_1_a	9132487	9	2	0
Digilio_1997_1_b	9132487	9	2	0
Digilio_1997_1_c	9132487	7	2	0
Digilio_1997_1_d	9132487	6	2	0
Digilio_1997_1_e	9132487	4	2	1
Digilio_1997_2_a	9132487	8	2	0
Digilio_1997_2_b	9132487	5	2	1
Digilio_1997_2_c	9132487	13	2	0
Debrus_1996_A	8566942	7	2	1
Debrus_1996_C	8566942	6	2	0
Debrus_1996_D	8566942	4	2	0
Debrus_1996_E	8566942	9	3	0
Debrus_1996_F	8566942	7	2	0
Debrus_1996_G	8566942	5	2	0
Debrus_1996_H	8566942	18	5	2
Debrus_1996_I	8566942	15	2	1
Debrus_1996_J	8566942	5	3	0
Debrus_1996_K	8566942	5	2	0
Debrus_1996_L	8566942	13	2	1
Debrus_1996_M	8566942	18	5	0
Debrus_1996_N	8566942	9	2	0
Debrus_1996_O	8566942	6	2	0
Debrus_1996_P	8566942	16	4	0
Der_Kaloustian_1985	4003436	6	2	0
Cassidy_1991	2042580	6	3	0
Silver_1972	5035578	5	2	0

de_la_Chapelle_1981	7250965	25	4	0
Rohn_1984	6737148	4	2	1
Wilson_1991	1747284	6	3	0
Raatikka_1981	7243440	6	3	0
Fuhrmann_1	5699890	4	2	0
Fuhrmann_2	5699890	4	2	0
Fuhrmann_3	5699890	4	2	0
Fuhrmann_5	5699890	4	2	0
Fuhrmann_6	5699890	4	2	0
Fuhrmann_7	5699890	4	2	0
Fuhrmann_9	5699890	4	2	0
Fuhrmann_11	5699890	4	2	0
Fuhrmann_12	5699890	4	2	0
Fuhrmann_13	5699890	4	2	0
Fuhrmann_17	5699890	4	2	0
Fuhrmann_20	5699890	4	2	0
Fuhrmann_21	5699890	4	2	0
Fuhrmann_22	5699890	4	2	1
Fuhrmann_25	5699890	4	2	0
Fuhrmann_27	5699890	4	2	0
Fuhrmann_28	5699890	4	2	0
Fuhrmann_31	5699890	4	2	0
Fuhrmann_33	5699890	4	2	0
Fuhrmann_34	5699890	4	2	0
Fuhrmann_35	5699890	4	2	0
Fuhrmann_36	5699890	4	2	0
Fuhrmann_41	5699890	4	2	0
Fuhrmann_42	5699890	4	2	1
Fuhrmann_44	5699890	4	2	0
Fuhrmann_45	5699890	4	2	0
Fuhrmann_46	5699890	4	2	0
Fuhrmann_47	5699890	4	2	0
Fuhrmann_49	5699890	4	2	0
Fuhrmann_52	5699890	4	2	0
Fuhrmann_53	5699890	4	2	0
Fuhrmann_58	5699890	4	2	0
Fuhrmann_59	5699890	4	2	0
Fuhrmann_61	5699890	4	2	0
Fuhrmann_63	5699890	4	2	0
Fuhrmann_64	5699890	4	2	0
Fuhrmann_72	5699890	4	2	0
Fuhrmann_74	5699890	4	2	0

Fuhrmann_77	5699890	4	2	0
Fuhrmann_78	5699890	4	2	0
Fuhrmann_a	5699890	4	2	0
Fuhrmann_b	5699890	4	2	0
Lynch_1966	5904472	5	2	0
Thammineni_2011_1	22037157	9	2	0
Thammineni_2011_2	22037157	4	2	0
De_Luca_1	19933292	18	2	0
De_Luca_2	19933292	12	2	0
De_Luca_3	19933292	13	2	0
De_Luca_4	19933292	16	2	0
De_Luca_5	19933292	12	2	0
De_Luca_6	19933292	19	2	0
De_Luca_7	19933292	5	2	0
Zlotogora_1980	7277426	4	2	0
Eronen_2004	14648004	6	4	0
Delatycki_1997	9098496	11	3	0
Morelli_2001_1	11471163	21	3	0
Morelli_2001_2	11471163	23	4	0
Morelli_2001_3	11471163	26	5	0
Soltan_1974	4839027	24	3	5
Toriello_1986	3146297	4	2	0
Mathias_1987	3674105	46	5	8
de_Meeus_1997	9021011	26	3	0
Arnold_1983	6638068	43	4	1
Rogers_1992	1433244	10	4	0
Slavotinek_1997	9217229	13	4	3
Lynch_1965_A	5897316	11	3	2
Lynch_1965_B	5897316	12	3	1
Burman_1961	13689120	7	3	1
Record_2	13093871	7	2	0
Record_3	13093871	4	2	0
Record_7	13093871	7	2	0
Sletten_1995	7645594	37	6	1
Ekstrom_S6	Acta Chirurgica Scandinavica[Suppl]1952;169:1-197	5	2	0
Ekstrom_KLB_9	Acta Chirurgica Scandinavica[Suppl]1952;169:1-198	4	2	0
Ekstrom_KLB_30	Acta Chirurgica Scandinavica[Suppl]1952;169:1-199	4	2	0
Ekstrom_KLB_52	Acta Chirurgica	5	3	0

	Scandinavica[Suppl]1952;169:1-200			
Ekstrom_S146	Acta Chirurgica Scandinavica[Suppl]1952;169:1-201	4	2	0
Niikowa_1983	6638069	10	3	0
Mikkila_1994	8160739	12	2	0
Chen_1977_a	874654	4	2	0
Chen_1977_b	874654	8	2	0
De_la_Monte_1985	4003441	7	2	0
Katcher_1980	7360556	4	2	2
Cesko_1999	11125242	6	2	0
Cesko_1997	9152295	4	2	0
Simpson_1973	4774542	5	2	0
Ruttenberg_1964	14128648	5	2	1
Alonso_2	7747776	11	2	0
Alonso_3	7747776	12	2	0
Alonso_4	7747776	14	4	0
Alonso_6	7747776	21	4	0
Casey_1996	8834043	25	4	0
Gerboni_1993	8487284	8	3	2
Shokeir_1971_A	5111754	4	2	2
Shokeir_1971_B	5111754	5	2	0
Shokeir_1971_C	5111754	6	2	0
Shokeir_1971_D	5111754	7	2	0
Shokeir_1971_E	5111754	9	2	1
Brenner_1	2589285	8	2	0
Brenner_2	2589285	8	2	0
Brenner_3	2589285	9	3	0
Brenner_4	2589285	5	2	0
Mu_2005	16235019	4	2	0
Menaheim_1990_1	2269543	5	3	1
Menaheim_1990_2	2269543	6	2	1
Fatimi_2006	17140990	4	2	0
Digilio_1992	8354327	4	2	0
Abdallah_1994	7991438	11	3	1
Richardson_1991	1829065	8	2	0
Onat_1984	6537946	8	3	2
Fryns_1979	573203	8	2	0
Urbach_1	4053117	7	3	0
Urbach_2	4053117	6	2	0
Urbach_3	4053117	13	2	0
Gale_1974	4533948	4	2	0

Goodyear_1960	13707075	5	2	1
Brunson_1978	685815	13	5	0
Abushaban_2003	12574981	40	6	0
Kwiatkowska_2006	17295158	8	3	0
Nakada_1996	8992863	6	2	0
Buch_1980	7211363	4	2	0
Kawashima_1987	3478461	5	3	0
Pankau_1990	2368805	7	2	0
Neufeld_1960	13728505	4	2	0
Gobel_1993	8469627	9	3	0
Cripe_a	15234422	6	3	0
Cripe_b	15234422	11	2	0
Cripe_c	15234422	5	2	0
Cripe_d	15234422	5	2	0
Cripe_e	15234422	6	3	0
Cripe_f	15234422	11	2	0
Cripe_g	15234422	12	3	0
Cripe_h	15234422	10	2	0
Cripe_i	15234422	10	2	0
Godden_1987	3582390	4	2	0
Clementi_1996	8723060	9	4	0
Huntington_1	9385911	6	2	0
Huntington_2	9385911	11	2	0
Huntington_3	9385911	6	2	0
Huntington_4	9385911	8	4	0
Huntington_10	9385911	15	2	0
Huntington_9	9385911	7	3	0
Huntington_23	9385911	6	2	0
Huntington_26	9385911	12	3	0
Huntington_27	9385911	10	2	1
Huntington_28	9385911	9	2	0
Brekke_1952	13050604	5	2	0
Moss_1955	14368483	5	2	0
Taylor_1952	13030400	4	2	1
Campbell_1961	13690260	4	2	0
Beekman_1985	4061317	11	4	0
Sehested	7200795	4	2	0
Keller_1965	14314175	6	2	0
Khau_van_Kien_2004	14722581	36	5	6
Joyce_1954	13160445	11	2	2
Davidson_1992	8326495	44	9	3
Glancy_2001	11249915	7	3	1

Martin	3767421	11	6	2
Gelb_1999	10533032	12	4	3
Wei_1984	6741821	8	4	0
Grobman_1996	8841248	7	3	0
Simon_1974_1	4426202	9	3	0
Simon_1974_2	4426202	5	2	0
Simon_1974_3	4426202	4	2	0
Simon_1974_4	4426202	6	2	0
Simon_1974_5	4426202	8	3	1
Lang_1991	1997221	4	2	0
McDonald_1989	2767077	5	3	0
Digilio_1997_a	9375931	15	2	0
Digilio_2001_1	11733399	8	2	0
Digilio_2001_3	11733399	17	3	0
Digilio_2001_4	11733399	6	2	0
Digilio_2001_5	11733399	6	3	1
Digilio_2001_6	11733399	12	2	0
Digilio_2001_7	11733399	4	2	0
Digilio_2001_8	11733399	4	2	0
Digilio_2001_9	11733399	4	2	0
Digilio_2001_10	11733399	8	2	0
Digilio_2001_11	11733399	5	2	0
Zlotogora_1987	3812559	9	4	0
Van_de_Meerakker_2011	21386876	25	5	0
McKusick_1972	4112361	5	2	0
Woods_1994	7815430	17	8	0
Davison_120	6082900	4	2	0
Davison_209	6082900	4	2	0
Davison_205	6082900	4	2	2
Davison_13	6082900	4	2	0
Davison_69	6082900	4	2	0
Davison_108	6082900	4	2	0
Davison_134	6082900	4	2	0
Davison_4	6082900	4	2	0
DiChiara_1980	7377161	7	2	0
Udwadia_1996	8781095	8	4	0
Klinge_1975	1111560	33	3	0
Friedberg_1974	4834778	26	4	0
Schwartz_1993	8357105	10	2	1
Burnell_1971	5095166	15	5	0
Polani_G79	13736684	4	2	0
Polani_G65	13736684	4	2	0

Polani_56	13736684	4	2	0
Polani_67	13736684	4	2	0
Hurwitz_1982	7172476	4	2	0
Zetterqvist_1960	13788275	14	4	9
Lynch_1978	148839	31	10	10
Okubo_2004	15235040	17	6	5
Kaufman_1972	Birth Defects: Original article series(1972):8;5 p 88	16	3	0
Petsas_1998	9474469	14	4	0
Vinh_1968	Arc Franc Ped 25:1141-9	4	2	0
Shokeir_1974_1	4470892	5	2	2
Shokeir_1974_2	4470892	5	2	0
Shokeir_1974_3	4470892	6	2	0
Shokeir_1974_4	4470892	7	2	0
Shokeir_1974_5	4470892	9	2	1
Hinton_1	17936159	13	2	3
Hinton_2	17936159	8	2	5
Hinton_3	17936159	9	2	0
Hinton_4	17936159	8	2	0
Hinton_5	17936159	17	2	2
Hinton_6	17936159	13	4	1
Hinton_7	17936159	6	2	1
Hinton_8	17936159	8	2	0
Hinton_9	17936159	5	2	1
Hinton_10	17936159	9	2	1
Hinton_11	17936159	7	2	0
Hinton_12	17936159	11	2	1
Hinton_13	17936159	4	2	0
Hinton_14	17936159	8	2	0
Hinton_15	17936159	6	2	0
Hinton_16	17936159	7	2	0
Hinton_17	17936159	10	2	0
Hinton_18	17936159	7	3	0
Hinton_19	17936159	9	2	1
Hinton_20	17936159	10	3	0
Hinton_21	17936159	13	4	4

Supplemental Table 4. Complete list of Odds Ratios. The list includes 178 pairs of diagnoses where the confidence interval (CI) for the Odds Ratios (OR) did not include one. Number of individuals indicates the number of pairs on individual level, meaning that a family with four affected can have the pair four times. In order to deflate this phenomenon we used the number of families with each pair of diagnoses (families). The last column shows how many individuals with diagnosis A without B and the opposite (B not A). Diagnoses with OR>1: Pairs of diagnoses with an OR of more than one indicating an increased relative risk. Diagnoses with OR<1: Pairs of diagnoses with an OR of less than one indicating a decreased relative risk. A not B (B not A) shows that these diagnoses occur very often in the data, but they still co-occur very rare, indicating that having one excludes having the other. Abbreviations are listed in Supplemental Table 2.

Diagnosis A	Diagnosis B	OR	CI	Number of pairs (families)	A not B (B not A)
Diagnoses with OR>1					
AbAB	TOF	5.89	[1.56-22.20]	11 (4)	5 (138)
AbAB	AVA	23.92	[2.58-222.05]	4 (1)	8 (6)
AbAB	AbV	72.00	[5.92-876.43]	3 (1)	8 (2)
AbPV	DORV	42.00	[2.56-689.30]	5 (1)	1 (27)
AbPV	RAA	36.45	[2.23-596.28]	2 (1)	1 (31)
AbTV	AVR	27.36	[2.90-257.92]	2 (1)	7 (6)
AbTV	HRHS	10.86	[1.26-93.80]	2 (1)	7 (15)
AbTV	TVA	16.36	[1.84-145.55]	2 (1)	7 (10)
AbV	HSA	18.21	[1.61-206.20]	3 (1)	2 (31)
AbV	Dxc	13.31	[1.18-149.71]	2 (1)	2 (42)
AbV	PAA	30.03	[2.61-345.51]	2 (1)	2 (19)
AbV	RAA	18.21	[1.61-206.20]	2 (1)	2 (31)
AbV	SA	40.93	[3.51-477.95]	2 (1)	2 (14)
AbV	VCAb	40.93	[3.51-477.95]	2 (1)	2 (14)
AoHy	Dxc	3.58	[1.03-12.41]	8 (3)	23 (40)
AoHy	PAS	4.90	[1.08-22.24]	4 (2)	24 (19)
APWin	PAH	104.55	[6.14-1780.00]	2 (1)	1 (11)
APWin	PFO	19.02	[1.18-307.89]	2 (1)	1 (58)
APWin	TA	23.70	[1.46-384.81]	2 (1)	1 (47)
ASD	EbA	6.07	[2.34-15.79]	42 (15)	333 (6)
ASDsv	PAPVR	29.92	[5.16-173.38]	4 (2)	4 (19)
ASDsv	EbA	9.47	[1.09-82.31]	2 (1)	6 (20)
AVA	PDA	5.31	[1.18-23.98]	6 (3)	4 (143)
AVA	MVS	17.35	[1.93-156.37]	2 (1)	6 (11)
AVmal	Dxc	27.27	[3.75-198.41]	5 (2)	2 (41)
AVmal	AVS	8.82	[1.23-63.21]	4 (2)	2 (118)
AVmal	CCTGA	47.29	[6.39-349.88]	4 (2)	2 (24)
AVmal	SV	47.32	[4.22-531.18]	4 (2)	1 (47)
AVmal	HSA	12.13	[1.23-119.91]	2 (1)	3 (31)
AVmal	RAA	12.13	[1.23-119.91]	2 (1)	3 (31)
AVmal	VCAb	27.26	[2.67-278.46]	2 (1)	3 (14)
AVR	BAV	5.64	[1.25-25.39]	11 (4)	3 (221)
AVR	MVA	21.24	[2.32-194.85]	2 (1)	6 (9)
AVR	PAA	9.97	[1.14-86.92]	2 (1)	6 (19)
AVS	BAV	7.36	[4.84-11.20]	165 (59)	47 (152)
AVS	supAS	6.66	[1.47-30.12]	9 (3)	117 (4)
AVSD	PLSVC	6.57	[3.22-13.42]	30 (13)	89 (23)
AVSD	SV	3.90	[1.95-7.77]	27 (12)	90 (35)
AVSD	CMV*	123.7	[15.80-969.5]	46 (11)	94 (1)

AVSD	HSA	5.12	[2.05-12.78]	14 (7)	89 (16)
AVSD	SI	3.24	[1.27-8.27]	14 (6)	96 (20)
AVSD	SA	7.06	[2.46-20.25]	12 (6)	99 (9)
AVSD	Iso	5.42	[1.34-22.02]	6 (3)	97 (6)
BAV	CoA	3.01	[2.10-4.32]	165 (59)	146 (111)
CCTGA	TGA	5.37	[2.34-12.36]	18 (9)	17 (102)
CCTGA	PVS	3.42	[1.38-8.47]	15 (7)	16 (129)
CCTGA	Dxc	9.66	[3.64-25.63]	14 (6)	19 (36)
CCTGA	SI	8.09	[2.60-25.20]	8 (4)	22 (25)
CCTGA	RAA	5.40	[1.53-19.10]	6 (3)	22 (28)
CCTGA	SV	4.72	[1.33-16.74]	6 (3)	17 (41)
CCTGA	TVA	12.27	[2.47-61.00]	4 (2)	23 (8)
CCTGA	VCAb	8.15	[1.73-38.52]	4 (2)	23 (12)
DORV	VSD	3.39	[1.33-8.69]	32 (12)	7 (381)
DORV	TGA	3.98	[1.62-9.76]	17 (7)	18 (101)
DORV	PVS	2.78	[1.08-7.17]	18 (6)	17 (128)
DORV	TA	5.52	[2.00-15.22]	10 (5)	23 (43)
DORV	Dxc	5.02	[1.66-15.23]	8 (4)	23 (38)
DORV	PVA	3.40	[1.14-10.16]	8 (4)	24 (53)
Dxc	PVS	6.22	[3.23-11.97]	39 (17)	23 (119)
Dxc	TGA	6.05	[2.98-12.29]	31 (13)	24 (92)
Dxc	TAPVR	11.71	[5.42-25.31]	29 (11)	30 (34)
Dxc	HSA	23.22	[10.07-53.57]	25 (11)	29 (18)
Dxc	SV	8.78	[3.89-19.81]	21 (9)	30 (37)
Dxc	PVA	6.91	[2.98-16.00]	18 (8)	29 (43)
Dxc	RAA	9.82	[3.94-24.45]	16 (7)	34 (23)
Dxc	Iso	16.24	[5.19-50.79]	14 (5)	38 (9)
Dxc	SA	10.34	[3.15-33.93]	9 (4)	39 (11)
Dxc	infPS	5.02	[1.66-15.23]	8 (4)	38 (23)
Dxc	PLSVC	4.02	[1.34-11.99]	8 (4)	35 (31)
Dxc	VCAb	7.76	[2.08-28.91]	6 (3)	39 (11)
Dxc	TVR	13.31	[1.18-149.71]	2 (1)	42 (2)
HSA	PVS	4.78	[2.14-10.66]	24 (10)	17 (124)
HSA	TGA	3.71	[1.61-8.54]	20 (8)	22 (101)
HSA	TAPVR	10.96	[4.35-27.62]	20 (7)	20 (35)
HSA	PLSVC	7.98	[2.84-22.41]	14 (5)	23 (30)
HSA	PVA	3.26	[1.10-9.69]	9 (4)	26 (51)
HSA	infPS	9.26	[2.94-29.16]	8 (4)	24 (20)
HSA	SV	4.32	[1.44-13.02]	8 (4)	24 (42)
HSA	PAPVR	6.05	[1.70-21.61]	6 (3)	29 (19)
HSA	SI	4.74	[1.35-16.63]	6 (3)	28 (25)
HSA	Iso	10.80	[2.19-53.35]	8 (2)	26 (8)
HSA	SA	6.43	[1.38-30.05]	4 (2)	29 (12)
HSA	TVR	18.21	[1.61-206.20]	2 (1)	31 (2)
infPS	PVS	2.85	[1.18-6.91]	16 (7)	19 (130)
Iso	TGA	7.18	[2.24-23.02]	14 (5)	7 (104)
Iso	TAPVR	18.97	[5.15-69.96]	12 (4)	6 (39)
Iso	PLSVC	16.91	[4.85-58.97]	10 (4)	8 (33)
Iso	SA	31.04	[7.50-128.44]	6 (3)	10 (11)
Iso	SV	8.18	[2.14-31.24]	6 (3)	9 (45)
Iso	VCAb	14.56	[2.96-71.68]	4 (2)	12 (13)
IAA	VSD	6.75	[2.47-18.43]	41 (17)	5 (381)
IAA	PDA	3.44	[1.46-8.11]	19 (8)	17 (137)
IAA	TA	9.85	[3.92-24.74]	15 (7)	19 (41)
IAA	Vring	22.70	[1.99-258.62]	2 (1)	25 (2)
MVA	TGA	9.98	[2.46-40.49]	11 (4)	4 (105)
MVA	SV	9.85	[1.86-52.15]	6 (2)	5 (45)
MVA	PVA	5.82	[1.18-28.66]	5 (2)	7 (54)

MVA	RAA	9.36	[1.91-45.95]	5 (2)	8 (30)
MVA	SI	10.43	[2.11-51.43]	4 (2)	8 (27)
MVA	TVA	12.70	[1.47-109.88]	2 (1)	9 (10)
MVR	TVR	12.39	[1.10-139.17]	2 (1)	45 (2)
MVS	RAA	7.47	[1.57-35.60]	4 (2)	10 (30)
MVS	TVS	104.55	[6.14-1780.00]	2 (1)	11 (1)
PAH	VSD	5.14	[1.36-19.50]	21 (8)	3 (393)
PAH	PVA	10.36	[3.02-35.49]	10 (4)	8 (53)
PAH	TA	8.19	[2.15-31.30]	10 (3)	9 (45)
PAH	RAA	7.47	[1.57-35.60]	6 (2)	10 (30)
PAH	PAS	11.92	[2.44-58.11]	4 (2)	10 (19)
PAH	supAS	17.35	[1.93-156.37]	2 (1)	11 (6)
PAPVR	PVS	2.84	[1.09-7.39]	13 (6)	16 (133)
PAPVR	SA	8.68	[1.84-41.00]	4 (2)	20 (13)
PAPVR	VCAb	8.68	[1.84-41.00]	4 (2)	20 (13)
PAS	supAS	89.33	[18.55-430.13]	15 (4)	17 (3)
PDA	VCAb	4.00	[1.32-12.11]	10 (5)	140 (9)
PLSVC	PVS	2.74	[1.26-5.98]	21 (9)	26 (126)
PLSVC	TGA	3.62	[1.65-7.95]	21 (9)	26 (98)
PLSVC	TAPVR	5.72	[2.25-14.55]	16 (6)	30 (38)
PLSVC	SA	21.80	[6.76-70.32]	10 (5)	32 (8)
PLSVC	SV	3.93	[1.46-10.58]	10 (5)	32 (43)
PLSVC	RAA	5.50	[1.81-16.72]	8 (4)	32 (25)
PLSVC	VCAb	7.51	[1.56-36.12]	4 (2)	33 (9)
PVA	TGA	3.07	[1.52-6.20]	27 (11)	39 (93)
PVA	RAA	7.86	[3.18-19.43]	18 (7)	46 (21)
PVA	TAPVR	3.73	[1.59-8.74]	16 (7)	50 (40)
PVA	SV	3.57	[1.44-8.86]	14 (6)	46 (39)
PVA	VCAb	7.51	[2.32-24.38]	9 (4)	53 (11)
PVA	SA	6.32	[1.69-23.67]	8 (3)	52 (10)
PVS	RAA	3.73	[1.72-8.10]	22 (10)	128 (21)
PVS	VCAb	3.39	[1.03-11.17]	8 (4)	133 (9)
PAA	SI	4.59	[1.02-20.79]	4 (2)	18 (27)
PAA	TVR	30.03	[2.61-345.51]	2 (1)	19 (2)
RAA	TOF	3.64	[1.68-7.89]	29 (10)	21 (131)
RAA	TGA	3.51	[1.53-8.05]	20 (8)	23 (102)
RAA	SV	6.94	[2.68-17.95]	13 (6)	23 (41)
RAA	TA	3.75	[1.26-11.19]	10 (4)	27 (43)
RAA	TAPVR	3.84	[1.29-11.48]	8 (4)	27 (42)
SA	TGA	3.54	[1.11-11.30]	10 (4)	11 (107)
SA	SI	16.34	[4.87-54.84]	9 (4)	11 (25)
SA	TAPVR	6.27	[1.71-23.03]	7 (3)	12 (44)
SI	SV	3.78	[1.27-11.32]	9 (4)	25 (46)
SI	VCAb	8.17	[1.72-38.79]	4 (2)	25 (11)
SV	TGA	5.44	[2.65-11.16]	27 (12)	26 (87)
SV	TAPVR	2.97	[1.12-7.89]	10 (5)	44 (41)
SV	VCAb	7.35	[1.96-27.64]	6 (3)	45 (10)
SV	TVA	5.88	[1.22-28.44]	4 (2)	47 (8)
TA	VSD	4.21	[2.20-8.05]	76 (29)	14 (368)
TAPVR	TGA	3.67	[1.84-7.31]	30 (12)	34 (98)
TAPVR	VCAb	7.90	[2.10-29.77]	6 (3)	42 (10)
TGA	Vring	19.28	[1.73-214.41]	4 (2)	109 (1)
TOF	TVA	6.17	[1.86-20.50]	10 (5)	137 (6)
VCAb	VSD	3.47	[1.16-10.43]	20 (9)	5 (392)
Diagnoses with OR<1					
ASD	BAV	0.21	[0.14-0.34]	77 (22)	321 (198)
ASD	HLHS	0.35	[0.21-0.57]	52 (19)	325 (118)
ASD	TOF	0.26	[0.15-0.45]	45 (16)	332 (126)

ASD	PVA	0.33	[0.14-0.79]	19 (6)	333 (42)
ASD	infPS	0.19	[0.05-0.82]	19 (2)	344 (24)
ASD	CCTGA	0.11	[0.02-0.82]	2 (1)	343 (21)
AVS	VSD	0.21	[0.12-0.38]	32 (13)	104 (386)
AVS	PDA	0.35	[0.15-0.81]	17 (6)	112 (138)
AVS	TOF	0.17	[0.05-0.53]	6 (3)	117 (139)
AVS	TGA	0.22	[0.07-0.71]	6 (3)	117 (108)
AVSD	BAV	0.28	[0.13-0.62]	15 (7)	96 (216)
BAV	VSD	0.23	[0.15-0.34]	65 (28)	192 (369)
BAV	PDA	0.34	[0.18-0.62]	34 (12)	213 (134)
BAV	PVS	0.17	[0.07-0.39]	15 (6)	218 (132)
BAV	TOF	0.08	[0.03-0.25]	7 (3)	222 (139)
BAV	TGA	0.11	[0.03-0.34]	8 (3)	221 (107)
BAV	PVA	0.15	[0.04-0.61]	4 (2)	222 (54)
BAV	SV	0.26	[0.08-0.83]	7 (3)	222 (47)
BAV	TA	0.17	[0.04-0.72]	5 (2)	223 (46)
BAV	PLSVC	0.22	[0.05-0.91]	4 (2)	223 (37)
BAV	RAA	0.13	[0.02-0.96]	2 (1)	224 (31)
CoA	PVS	0.36	[0.19-0.71]	26 (10)	180 (129)
CoA	TOF	0.10	[0.03-0.31]	6 (3)	187 (139)
CoA	PVA	0.18	[0.04-0.73]	4 (2)	188 (55)
HLHS	TGA	0.33	[0.13-0.82]	13 (5)	134 (104)
HLHS	TOF	0.09	[0.02-0.37]	4 (2)	139 (140)
HLHS	PFO	0.12	[0.02-0.86]	2 (1)	140 (58)

* We are aware that the correct anatomical term in this setting is “cleft left AV valve”. Some CMV diagnoses may be AVSD

Supplemental Table 5. List of deduced susceptibility genes. Genes associated with specific cardiac phenotypes were identified using the Mouse Genome Informatics database (http://www.informatics.jax.org). Human orthologous to the mouse genes are listed.

**Phenotype** *	**Genes (HGNC symbol)**
VCAb	CITED2 ZIC3 DNAH11 LEFTY2 LEFTY1 DNAH5 DYNC2H1 IFT74 CFC1 CFC1B PLXND1 CEP290 DRC1
SI	DNAH11 MGAT1 ZIC3 KIF3A INVS PKD2 FOXJ1 CFC1 CFC1B RFX3 CITED2 PITX2 DAND5 DPCD DNAH5 GDF1 TGIF1 MGRN1 LEFTY1 BICC1 DLL1 MBD4 IFT122 CCDC40 NME7 PKD1L1 MKS1
Dxc	PITX2 FGF10 ZIC3 ACVR2B FOXJ1 CFC1 CFC1B RFX3 PCSK6 CITED2 INVS GDF1 MGRN1 RPGRIP1L LEFTY2 DNAH5 PCSK5 DNAH11 PKD2 MEGF8 MKS1 PKD1L1 DNAI1 BICC1 B9D1 CCDC39 ARMC4 DRC1
TAPVR	NTF3 NTRK3 TGFB3 HMOX2 SEMA3D
PLSVC	PITX2 PIKFYVE
Iso	CITED2 NODAL INVS LEFTY2 LEFTY1 CCDC40 DNAH11 CEP290
AVSD	PITX2 PKD1 RXRA CITED2 GATA4 ZFPM2 TGFB2 CYR61 GDF1 GJA5 LEFTY2 PDS5B LEFTY1 DNAH5 MEGF8 SRSF10 GPC3 IFT172 DYNC2H1 CFC1 CFC1B CC2D2A PLXND1 PCSK5
SA	ZIC3 PKD2 INVS TGIF1 NODAL BICC1
PAS	RXRA CITED2 HEY2 LOX
PAH	TGFB2 GJA5 KIF7 EPAS1
CCTGA	EDNRA NODAL HSPG2 ACVR2B DVL2 FOXJ1 ROR2 CFC1 CFC1B NRP1 ZIC3 INVS GDF1 KAT6A LEFTY2 LEFTY1 PITX2 DNAH5 TGFBR3 LUZP1 SMAD7 SEC24B PNN MKS1 DRC1 PATZ1 RERE
infPS	NTF3 ZFPM2 MYH10 HHEX GJA1 TRIP11
PAPVR	NTF3 NTRK3 TGFB3 HMOX2 SEMA3D
PVA	CC2D2A CXCL16 FUZ KIF15
TGA	BICC1 MEGF8 MMP21 SMAD6 DNAH11 PCSK5 CEP290
RAA	CITED2 ZIC3 ALDH1A2 PITX2 JUN CHRD NRP1 CFC1 CFC1B VEGFA PLXND1 TBX1 VANGL2 GBX2 MGRN1 LEFTY1 LTBP1 HOXA1 PCSK5 MEGF8 MKL2 MKS1 INVS DNAH5 ARMC4 FUZ NPRL3 DNAH11 RERE
SV	NKX2-5 MYCN SMYD1 GATA4 PCSK6 SALL4 KAT6A FLNA
PVS	NTF3 NTRK3 HEY2 ADAM19 PHC1 HBEGF FGFRL1 ACKR3 GATA4
DORV	EDNRA PITX2 PKD1 NF1 CRKL RXRA FGFR2 TFAP2A CITED2 ZIC3 TLL1 DVL2 GATA4 TGFB2 SEMA3C EDN1 ECE1 PAX3 CFC1 CFC1B FGF19 PTPN11 PSEN1 PCSK6 FOXP1 TBX1 HHEX JMJD6 ZFPM2 VANGL2 MKL2 INVS GBX2 FBLN1 GJA5 FLNA SSR1 MGRN1 JARID2 LEFTY2 LEFTY1 DNAH5 PCSK5 MYH10 LUZP1 AIP DVL3 PDS5A GPC3 BAZ1B PNN PIFO PTK7 DAAM1 DNAI1 BICC1 SOX11 IFT74 CC2D2A SUFU TAB1 LTBP1 SMAD6 PLXND1 SMARCA4 NPRL3 HSPB11 TMEM67 ARMC4 CEP290 MEGF8 CNTRL CCDC39 ROBO1 KIF7 NEK8 RERE
TVA	HEY2 ZFPM2 LEFTY2 LEFTY1 CC2D2A MMP21 SMAD6
AbTV	NTRK3 RXRA IDUA ADAM19 NFATC1 HBEGF ADAM17 IGF2R COL18A1
HRHS	PITX2 HAND2 NEK8 TMEM67 CNTRL
MVA	RXRA
AoHy	HOXA3 FOXJ1 INVS HECTD1
MVS	RXRA GJA5
PFO	HEY2 GJA1 NKX2-5 CYR61 GATA4
BAV	PLCE1 NOS3 NKX2-5 HOXA1 GATA5
CoA	ATP2A2 BAZ1B CHD7

AVS	ADAM19 HOXA3 PHC1 PLCE1 HBEGF INVS NKX2-5 WRN MUS81 ACKR3 SOS1
ASD	NTF3 NTRK3 NOS3 HEY2 PDS5B DNAH5 FBLN1 RYR1 SOS1
PDA	EDNRA PTGER4 FOXC1 PTGS2 HPGD PCSK5 GPC3 SLCO2A1 CACNA1C SMARCA4 TFAP2B
PAA	TGFB2 GJA5 KIF7 EPAS1
MVR	IDUA HEY2 ARSB
TOF	EDNRA NTF3 CRKL FGFR2 CITED2 ZFPM2 MYH10 HEY2 ADAM19 EDN1 ECE1 PHC1 FGF19 VEGFA KAT6A GBX2 ACKR3 HOXA1 FBLN1 TRIP11 HECTD1 SUFU TAB1 SMAD6 SMARCA4 NPRL3 HSPB11 KIF7
IAA	FOXC2 CRKL TFAP2A FOXC1 CITED2 BMPR2 TGFB2 SEMA3C ECE1 VEGFA TBX1 ZIC3 GBX2 FLNA LTBP1 HOXA1 DNAH5 CHD7 MKL2 RIPPLY3 EYA1 TAB1 NPRL3 TMEM67 KIF7
VSD	FOXC2 PKD1 PKD2 ADAM17 NF1 NTRK3 CRKL CITED2 FOXC1 ZIC3 ATE1 TLL1 HEY2 PAX3 ALDH1A2 ACVR2B DVL2 PITX2 JUN GATA4 ZFPM2 SIRT1 EP300 SOX4 EDN1 ECE1 CYR61 PHC1 CFC1 CFC1B CXCR4 FGF19 TBX1 VEGFA PTPN11 PSEN1 SOX11 PCSK6 DAND5 FOXP1 HHEX JMJD6 VANGL2 CHD7 NOS3 FKBP1A TBX5 GDF1 KAT6A ATP2A2 FBLN1 GJA5 FLNA MKL2 SALL4 MGRN1 JARID2 ACKR3 PDS5B LEFTY1 NCOR2 HOXA1 DNAH5 PCSK5 MYH10 LUZP1 AIP IFT88 HEG1 MEGF8 SMAD7 NFATC1 FES PDS5A SRSF10 GPC3 BICC1 NIPBL PNN PDGFRA RNF4 PTK7 GBE1 RIPPLY3 DAAM1 NXN SOS1 SMARCA4 CYP51A1 TCTN2 DOCK1 B9D1 CC2D2A MMP21 CXCL16 TAB1 DNAH11 NPRL3 HSPB11 ARMC4 KIF15 CNTRL CEP290 ARID1A KIF7 ROBO1 YWHAE LIN28A
TA	EDNRA NF1 NTF3 RXRA TFAP2A CITED2 ATE1 PAX3 ALDH1A2 DVL2 JUN CHRD SEMA3C HOXA3 ECE1 NRP1 VEGFA PLXND1 SOX11 PCSK6 FOXP1 TBX1 FLNA SSR1 MKL2 LTBP1 SOX4 PCSK5 IFT88 DVL3 PARVA FUZ PNN NXN RDH10 CC2D2A TAB1

* Abbreviations are listed in Supplemental Table 2.

Supplemental Table 6. List of human CHD disease genes. Gene lists were generated from reference 1-49 (see supplemental references).

Phenotype *	Genes (HGNC symbol)
ASD	ACTC1 ADNP BCOR BRAF CDK13 CFC1 CHD4 CHD7 CITED2 CREBBP CYR61 DHCR7 DYRK1A EHMT1 FOXC1 GATA4 GATA6 JAG1 KDM6A KIAA0196 KMT2D KRAS MYH6 MYH7 NKX2 NODAL NOTCH2 NSD1 PACS1 PITX2 PTPN11 RBM10 RIT1 SALL1 SALL4 SOS1 STK4 STRA6 TBX1 TBX20 TBX5 ZIC3 MMP21
AVS	NOTCH1 SMAD6
AVSD	ACVR2B ALK2 ANKRD11 CFC1 CRELD1 FOXP1 GATA4 GATA5 GATA6 LEFTY2 NODAL NR2F2 PRKD1 PTPN11 ZIC3 DNAI1 DNAH5 DNAH11 MKS1 MKKS EVC NR1D2 MMP21
BAV	GATA5 JAG1 KMT2D NOTCH1 SMAD6
CCTGA	ACVR2B MMP21 NODAL SHROOM3
CoA	CHD4 GATA5 KMT2D NR2F2 NSD1 PACS1 SMAD6
DORV	ACVR2B BCOR CFC1 CHD7 GATA6 GDF1 MMP21 NKX2-5 NKX2-6 NODAL NOTCH1 NPHP4 PITX2 SH3PXD2B TBX1 TBX5 ZFMP2 ZIC3 ZIC3
Dxc	ACVR2B CFC1 CRELD1 GATA4 GATA6 GDF1 LEFTY2 MMP21 NODAL NPHP4 PKD2 RNF20 SHROOM3 ZIC3
EbA	MYH7 NKX2-5
HLHS	CDK13 CFC1 FOXP1 LEFTY2 NKX2-5 NOTCH1 NR2F2 PACS1 ZIC3
Iso	CFC1 ZIC3
IAA	CHD7
MVA	MMP21
PAPVR	GATA4 MMP21 NODAL
PAS	ACVR2B GDF1 NODAL NOTCH2 ZIC3
PDA	CFC1 CHD7 KMT2A NSD1 PACS1 TFAP2B
PVA	NOTCH1
PVS	CDK13 CHD4 JAG1 MMP21 NODAL PRKD1 PTPN11
PAA	CFC1 CRELD1 FOXP1 MMP21 NODAL NSD1 ZIC3
RAA	CHD7 CRELD1
SA	GDF1 ZIC3
SI	CCDC11 DNAH5 DNAI1 GDF1 NKX2-5 NODAL NPHP2 NPHP3 NPHP4 PKD2 ZIC3
SV	FOXP1 MMP21 NODAL ZIC3
TA	CHD7 GATA4 GATA6 NKX2-5 NKX2-6 NOTCH1 NRP1 PRDM1 TBX1
TAPVR	ACVR2B ANKRD1 CFC1 GDF1 NKX2-5 NODAL PDGFRA SEMA3D ZIC3
TGA	ACVR2B ANKRD11 CFC1 GDF1 MED13L MMP21 NODAL PACS1 ZIC3
TOF	ALDH1A2 BRAF CFC1 CHD4 CHD7 FOXC1 FOXC2 FOXH1 GATA4 GATA6 GDF1 GJA5 HAND2 HOXA1 JAG1 KMT2A MAP2K1 MEK1 MESP1 NKX2-5 NKX2-6 NODAL NOTCH1 NOTCH2 RAF1 SALL4 TBX1 TBX20 TBX5 TDGF1 TFAP2B ZFMP2
TVA	MYH6 NFATC1 NKX2-5
VCAb	ACVR2B CFC1 MMP21 NODAL ZIC3
VSD	ACVR2B ANKRD11 B3GALTL B3GAT3 BCOR CDK13 CFC1 CHD4 CHD7 CITED2 DNAH5 DNAI1 DVL1 EVC1 EVC2 FBN1 FGFR3 FOXL2 GATA4 GATA6 GDF3 GPC3 HAS2 HRAS IRX4 JAG1 KMT2D KRAS LBR MESP1 MID1 MKRN2 MLL2 MMP21 MYH7 NF1 NIPBL NKX2-5 NKX2-6 NODAL NOTCH1 NOTCH2 NSD1 PACS1 PITX2 PTPN11 ROR2 SALL1 SALL4 SH3PXD2B SHOC2 SOS1 TAB2 TBX1 TBX20 TBX3 TBX5 TDGF1 TFAP2B TNNI3 WNT5A ZEB2 ZIC3

* Abbreviations are listed in Supplemental Table 2.

Supplemental Table 7. Combinations of diagnose severities in Families. Data is shown as percentage of families. Note that rows sum to >100% because many families have more than one combination of diagnoses.

	**Combination of severity** †					
	1:1	1:2	2:2	1:3	2:3	3:3
All pairs (n=1163) *	46	44	47	15	17	12
Parent-offspring pairs (n=432) *	44	41	41	9	6	7
Sibling-pairs (n=699) *	49	41	43	15	17	13

* The number of families with the specific combination of pairs. † Percentage of pairs with the specific combination of malformation severity is shown. (e.g. 432 families had parent-offspring pairs, and 44% of these pairs presented with the severity combination 1:1). Severity is shown on a scale from 1-3 (1=low severity, 2=intermediate severity, 3=high severity). Severity score of individual malformations are shown in Supplementary Table 2.

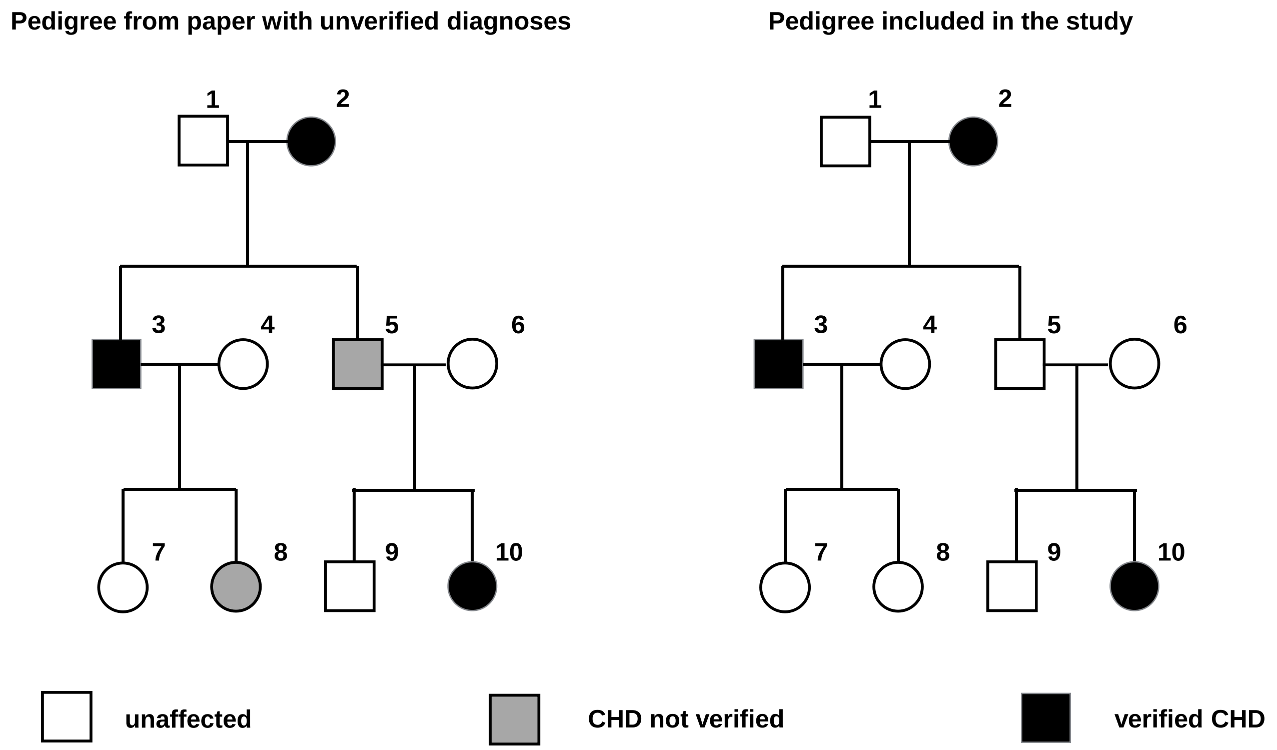

Supplemental Figure 1. Exclusion of affected individuals with unverified diagnoses. Only CHD diagnoses verified by echocardiography, heart catheterization, surgery or autopsy was included in the study. Occasionally, pedigrees from the literature contained individuals with unverified diagnoses (marked with grey). These individuals were excluded before the family was included in the study.

Supplemental Figure 2. Pedigrees of 637 previously unpublished CHD families. The families were collected by the coauthors; SG Ellesøe (Ellesoe, n=201), P Bouvagnet (LYS, n=262), CA Loffredo (Loffredo, n=61), AV Postma (Postma, n=54), KL McBride (McBride, n=59).

Ellesoe_0

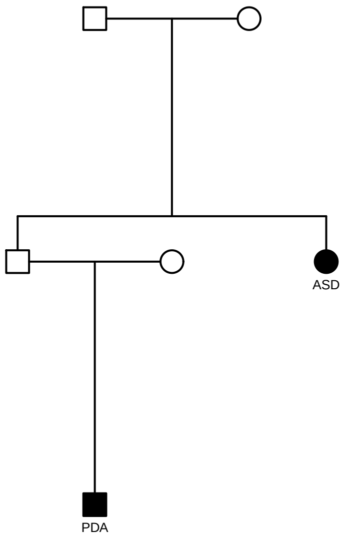

Ellesoe_2

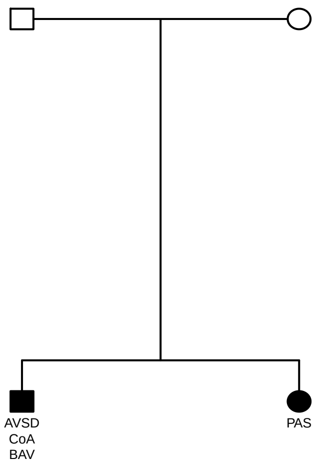

Ellesoe_4

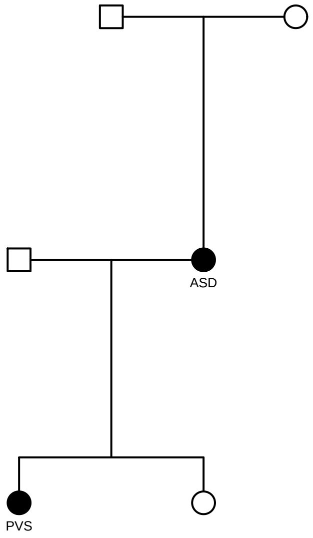

Ellesoe_12

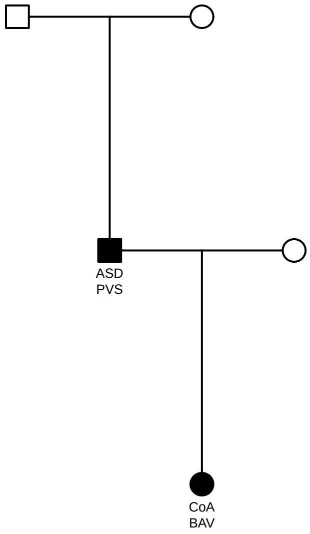

Ellesoe_22

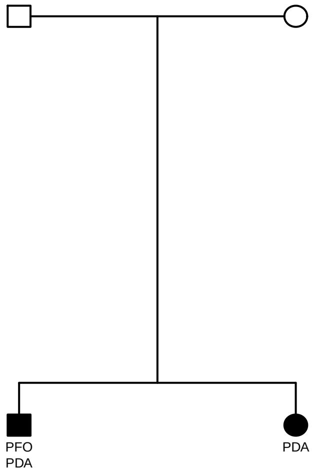

Ellesoe_33

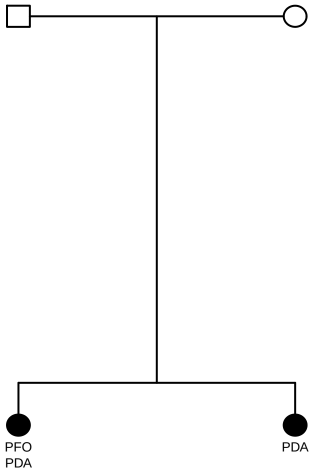

Ellesoe_35

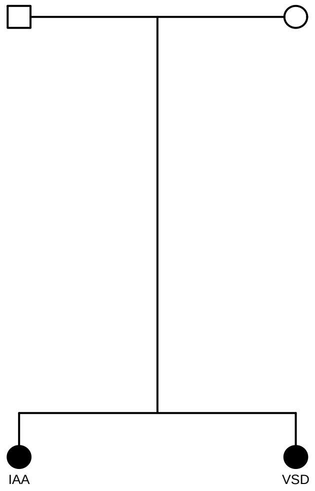

Ellesoe_49

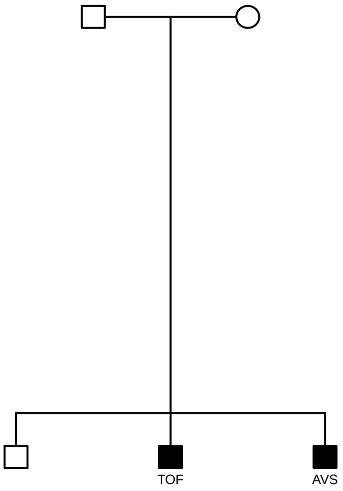

Ellesoe_53

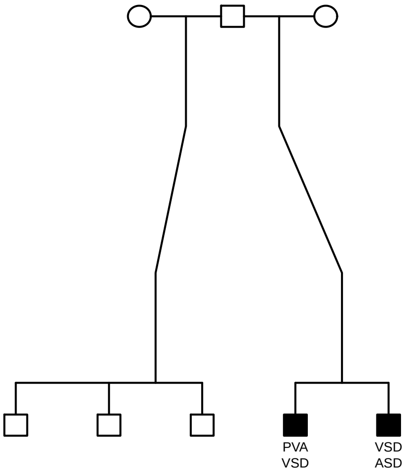

Ellesoe_55

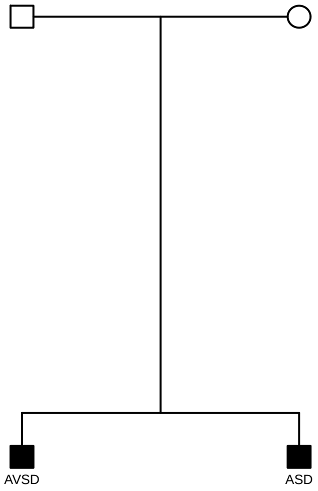

Ellesoe_58

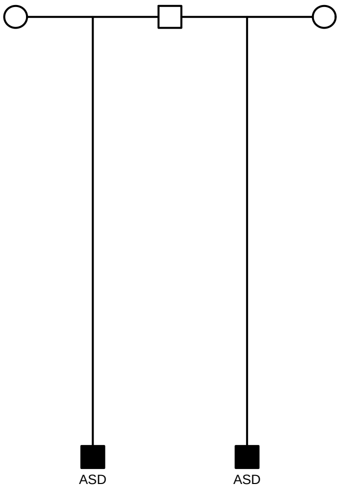

Ellesoe_62

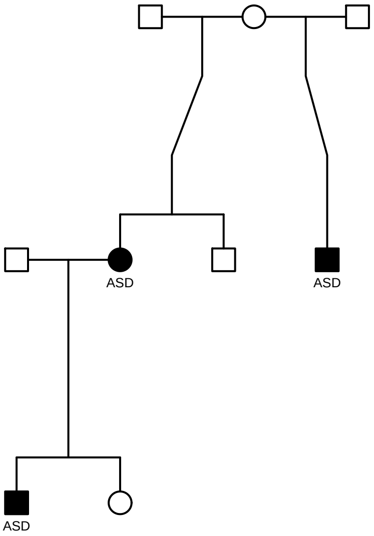

Ellesoe_66

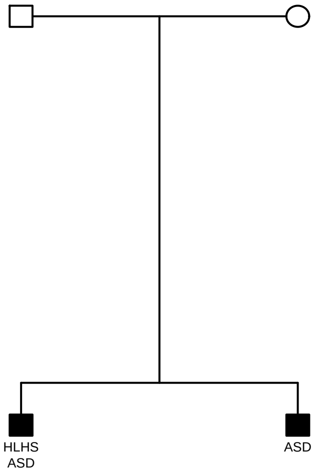

Ellesoe_68

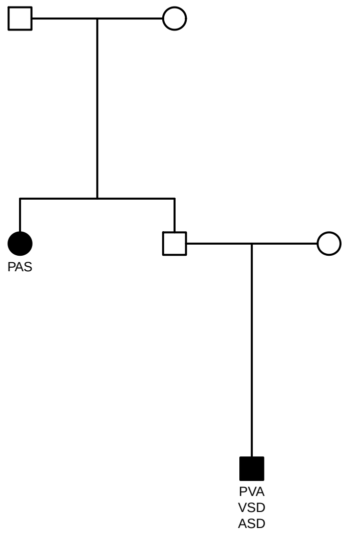

Ellesoe_73

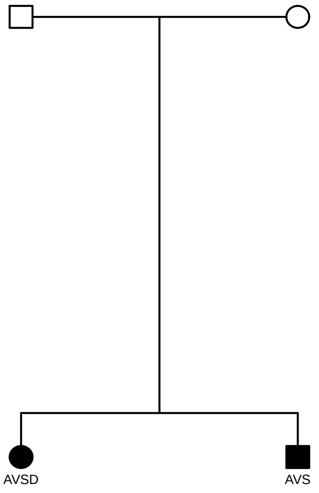

Ellesoe_77

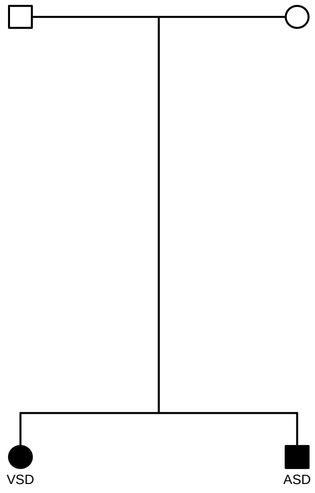

Ellesoe_79

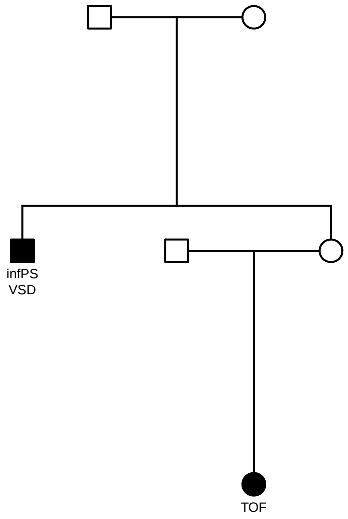

Ellesoe_82

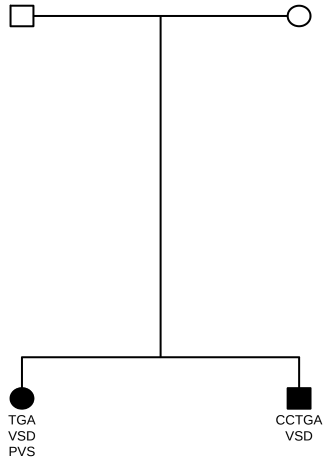

Ellesoe_83

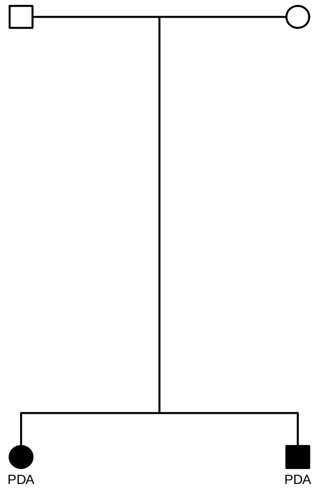

Ellesoe_89

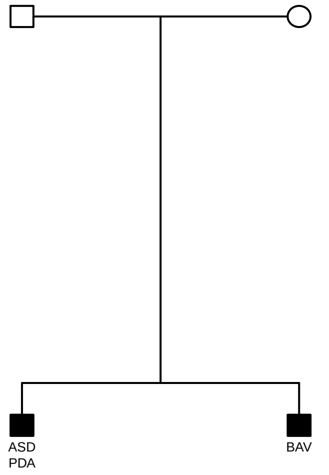

Ellesoe_99

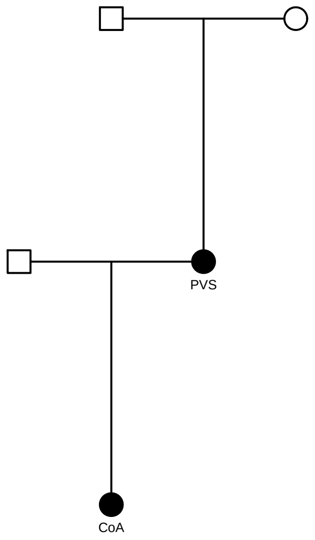

Ellesoe_106

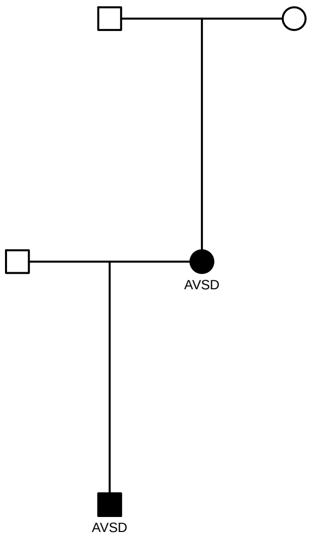

Ellesoe_109

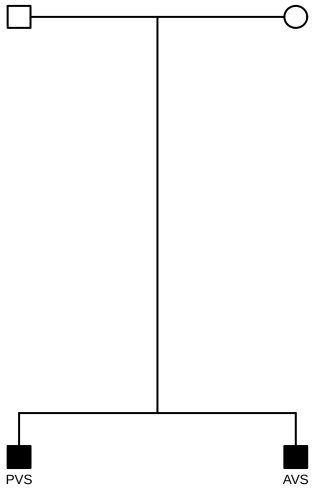

Ellesoe_110

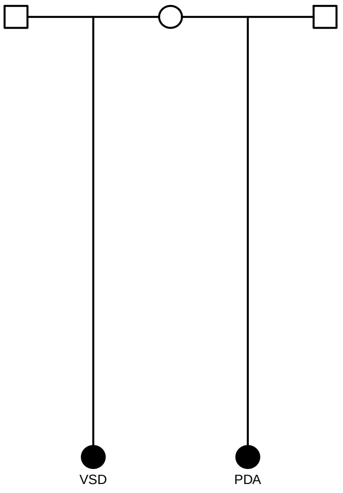

Ellesoe_114

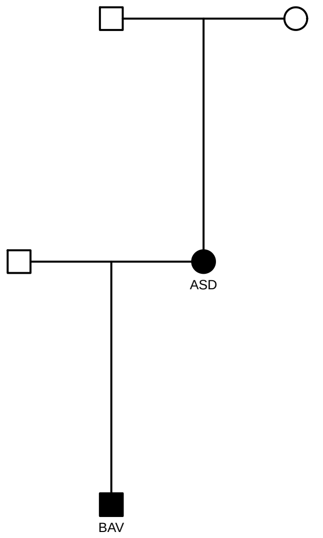

Ellesoe_115

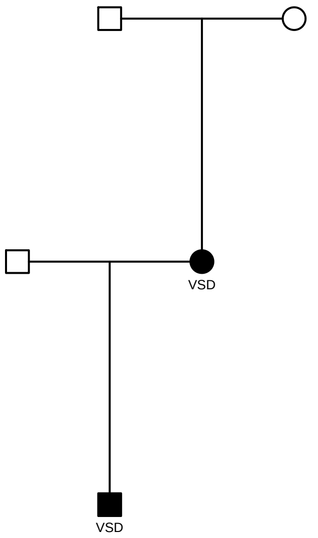

Ellesoe_117

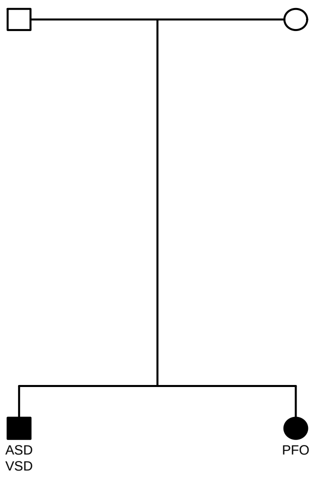

Ellesoe_140

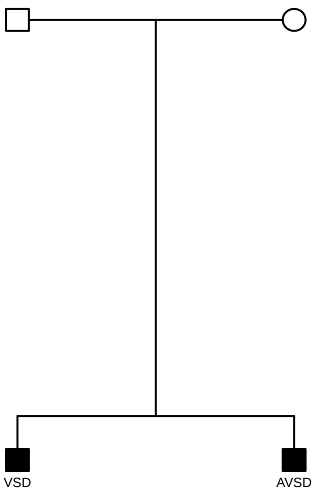

Ellesoe_145

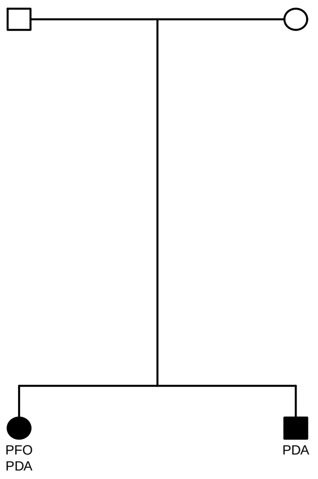

Ellesoe_147

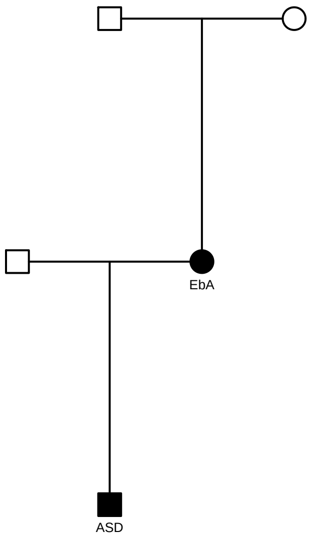

Ellesoe_151

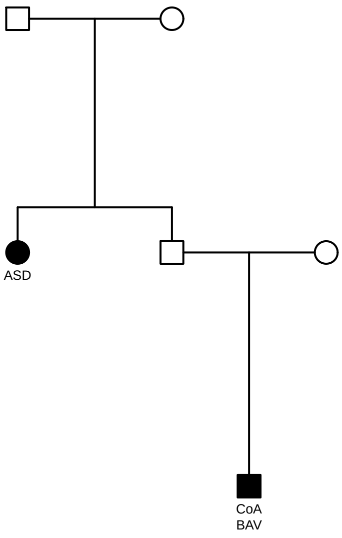

Ellesoe_157

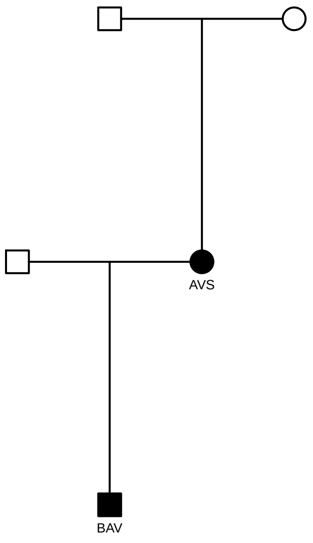

Ellesoe_159

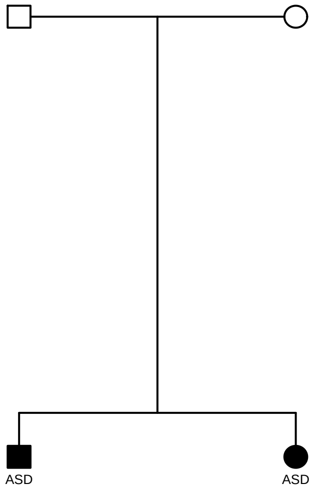

Ellesoe_173

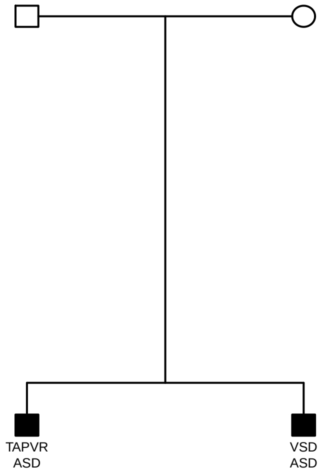

Ellesoe_177

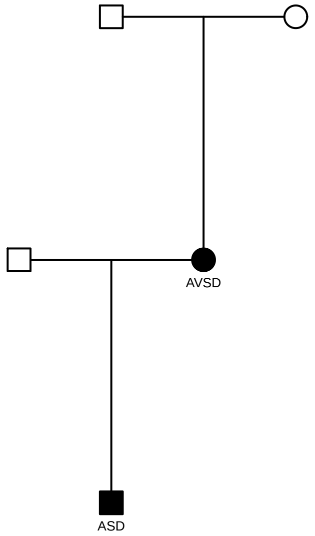

Ellesoe_186

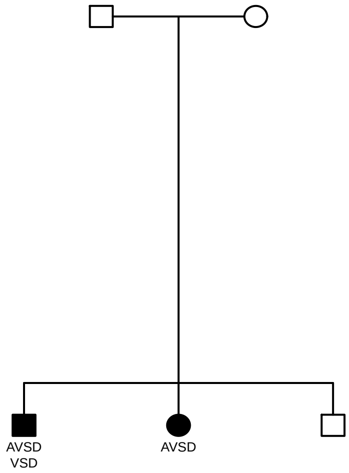

Ellesoe_193

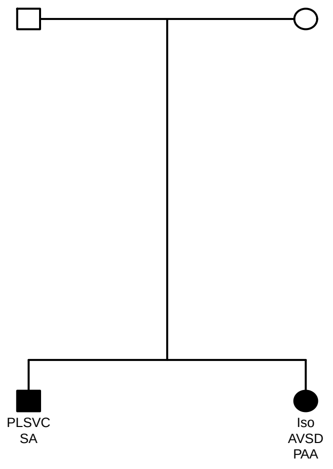

Ellesoe_194

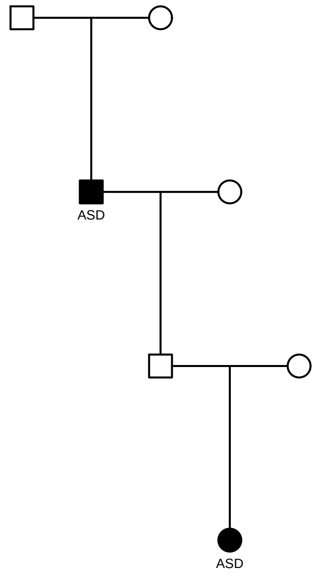

Ellesoe_200

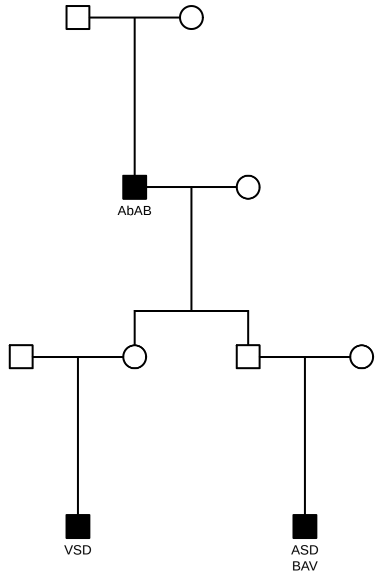

Ellesoe_211

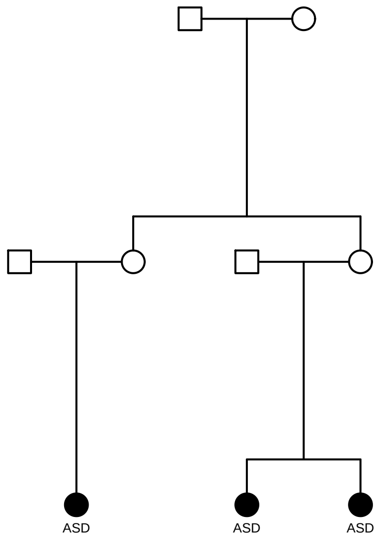

Ellesoe_223

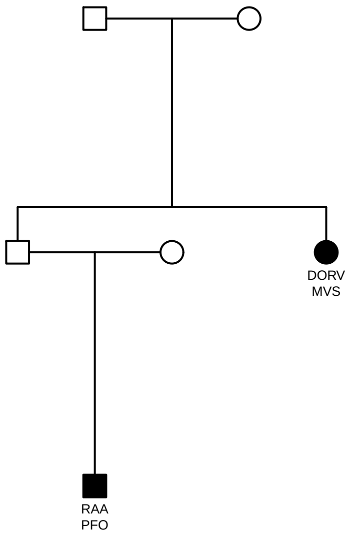

Ellesoe_226

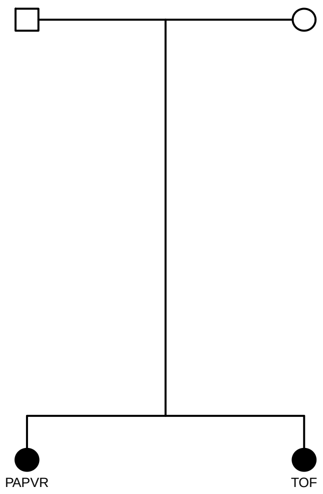

Ellesoe_231

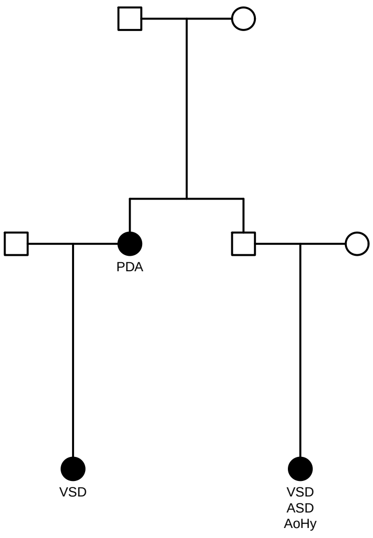

Ellesoe_240

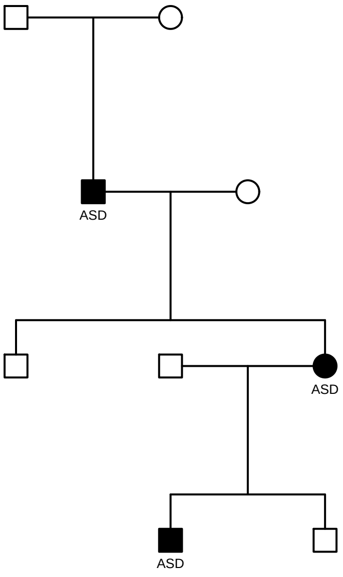

Ellesoe_241

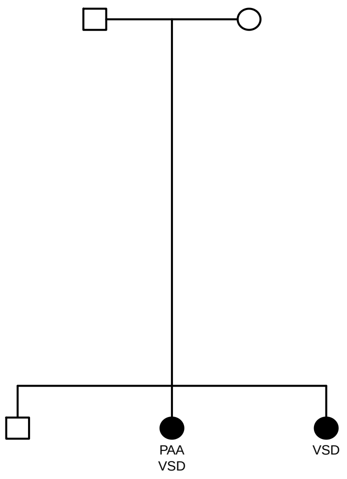

Ellesoe_245

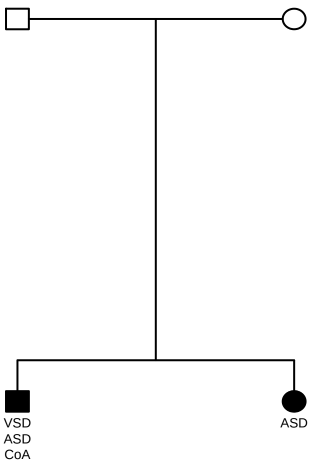

Ellesoe_249

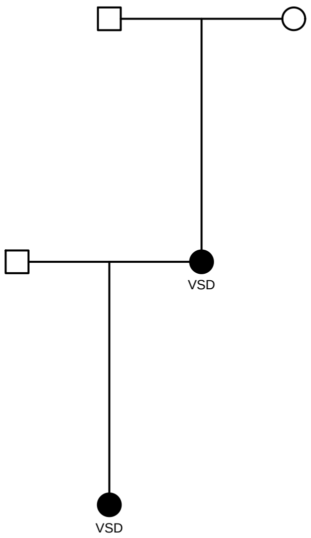

Ellesoe_253

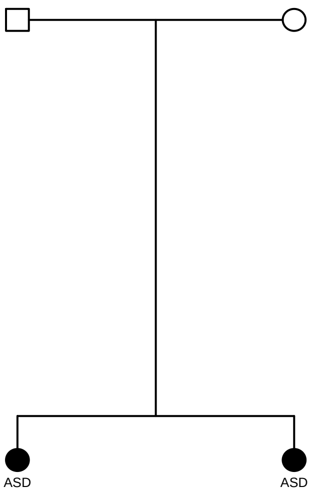

Ellesoe_254

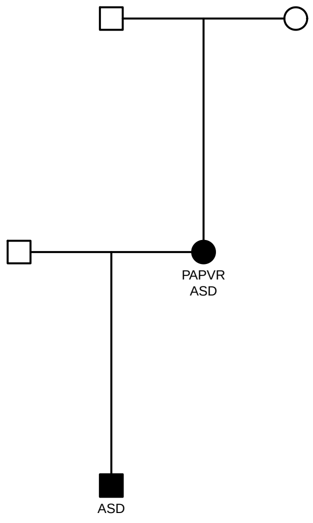

Ellesoe_264

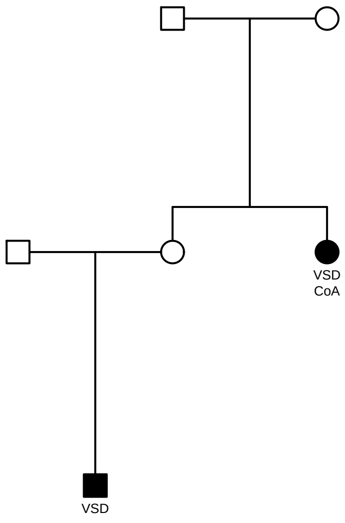

Ellesoe_281

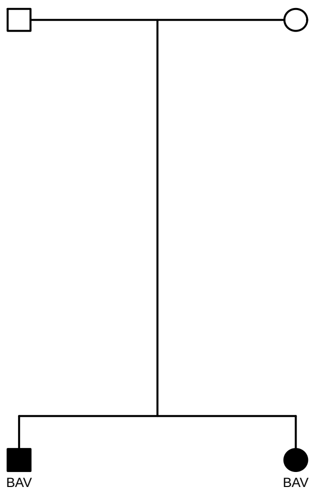

Ellesoe_286

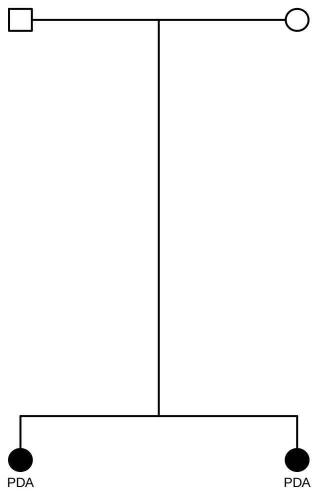

Ellesoe_301

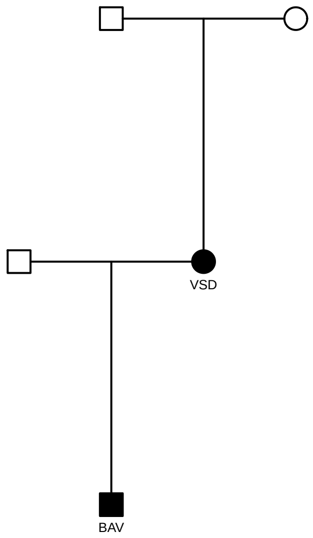

Ellesoe_316

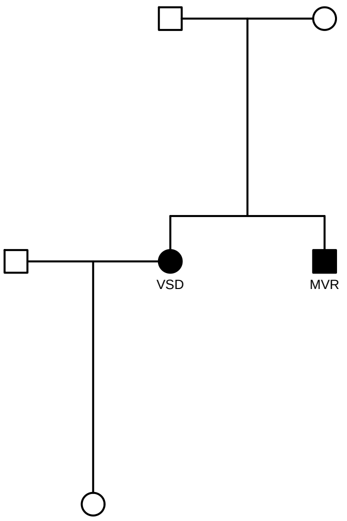

Ellesoe_331

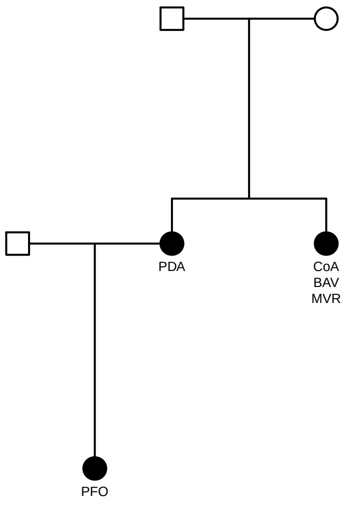

Ellesoe_333

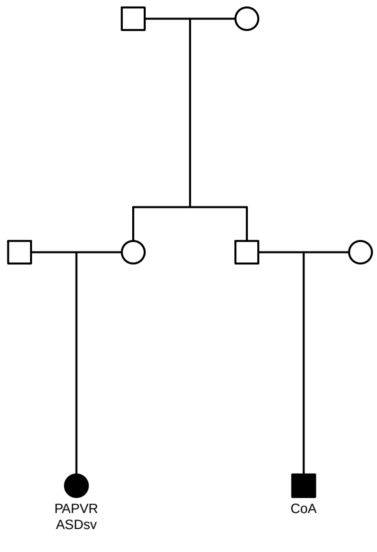

Ellesoe_334

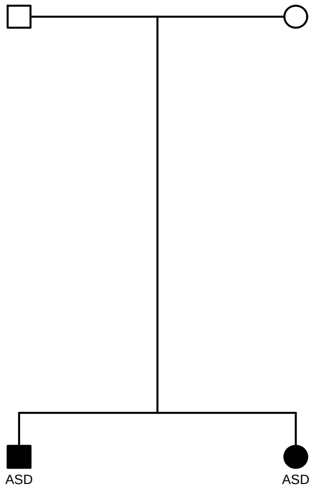

Ellesoe_338

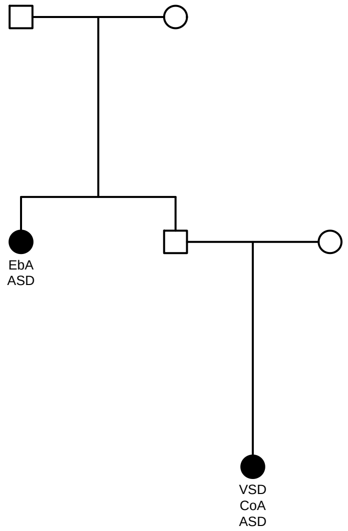

Ellesoe_340

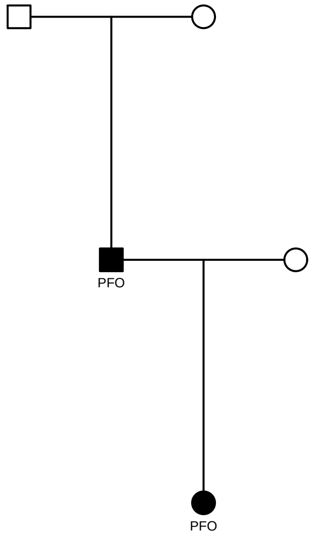

Ellesoe_343

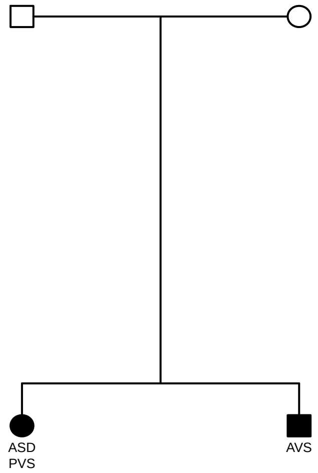

Ellesoe_346

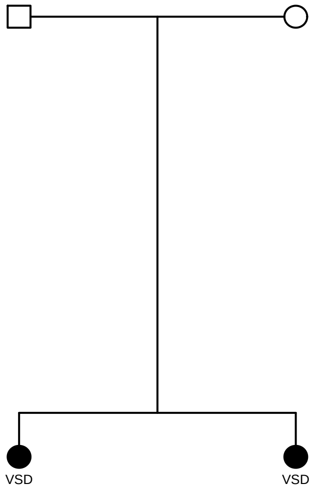

Ellesoe_347

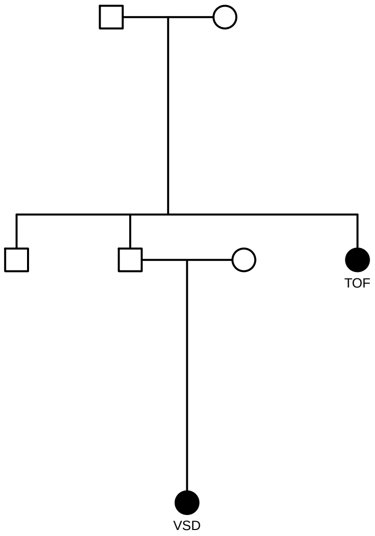

Ellesoe_349

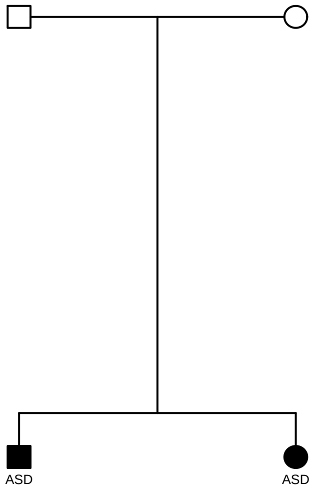

Ellesoe_353

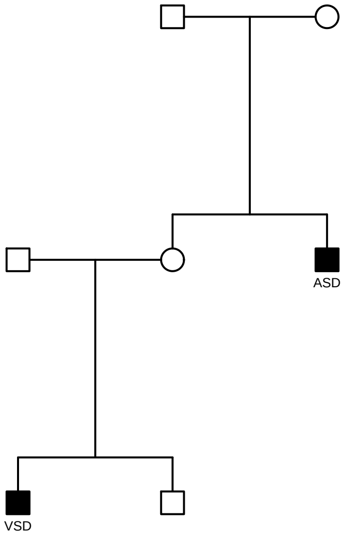

Ellesoe_354

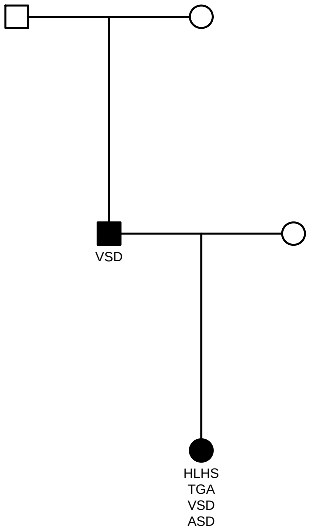

Ellesoe_398

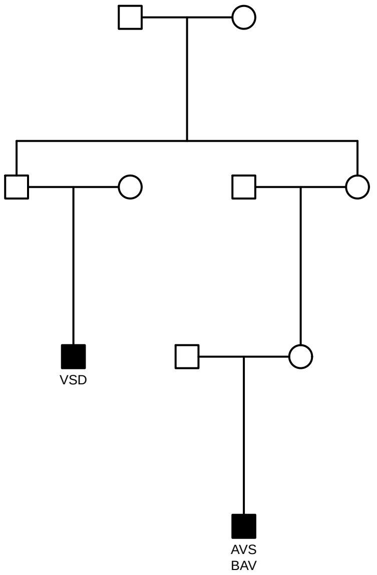

Ellesoe_420

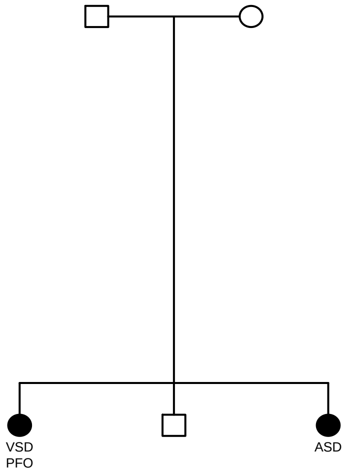

Ellesoe_454

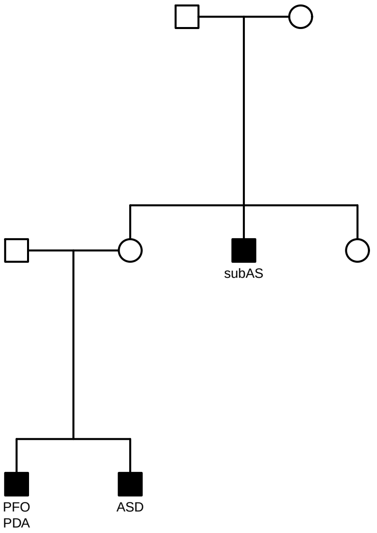

Ellesoe_466

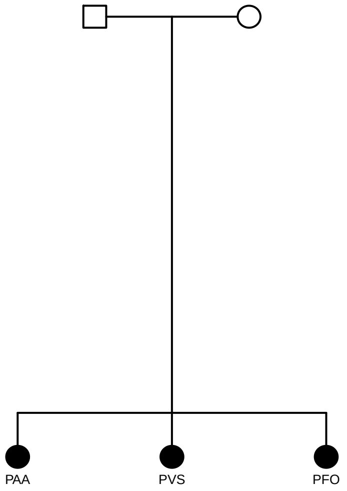

Ellesoe_476

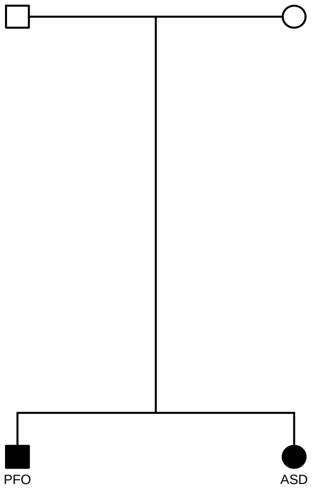

Ellesoe_477

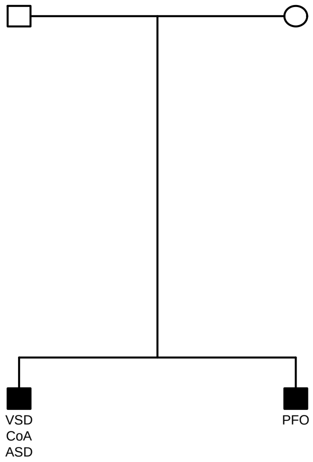

Ellesoe_489

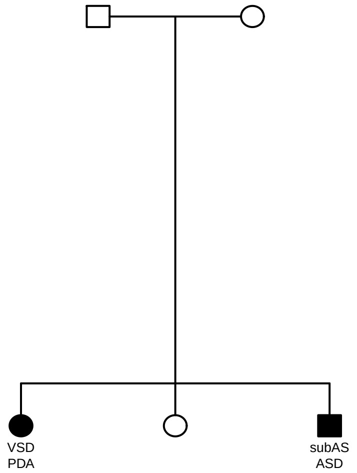

Ellesoe_503

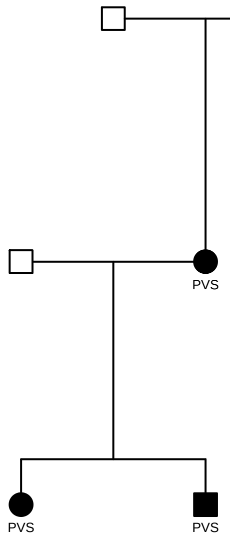

Ellesoe_528

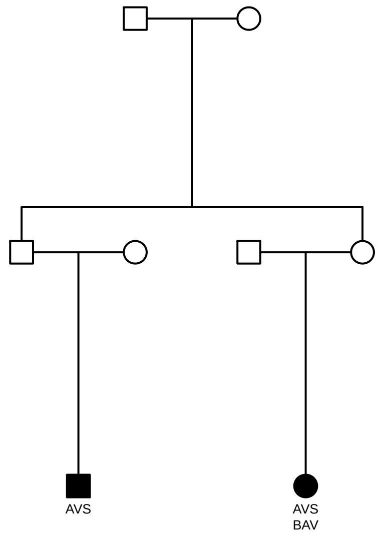

Ellesoe_535

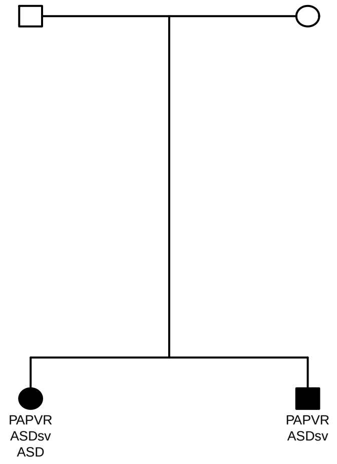

Ellesoe_543

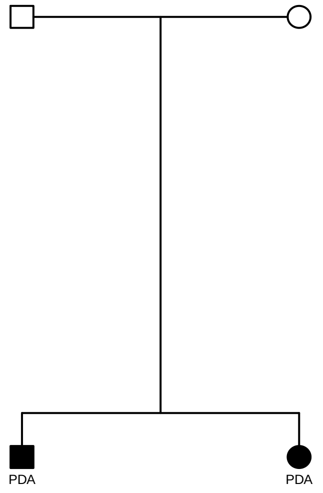

Ellesoe_545

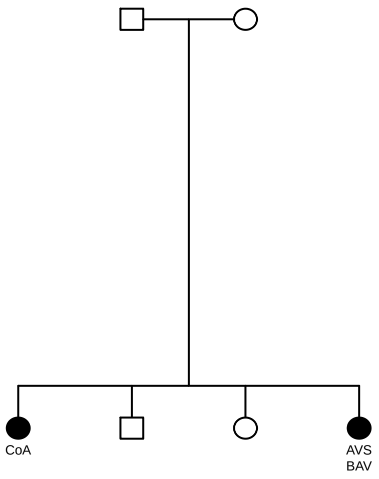

Ellesoe_570

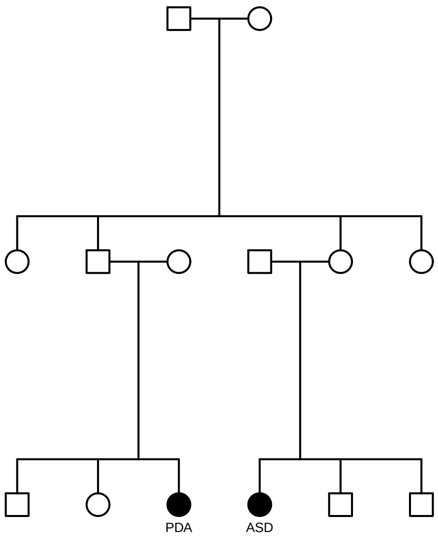

Ellesoe_576

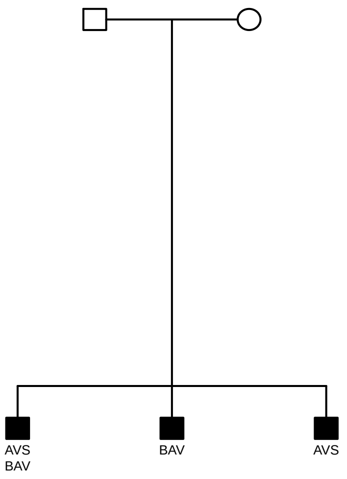

Ellesoe_577

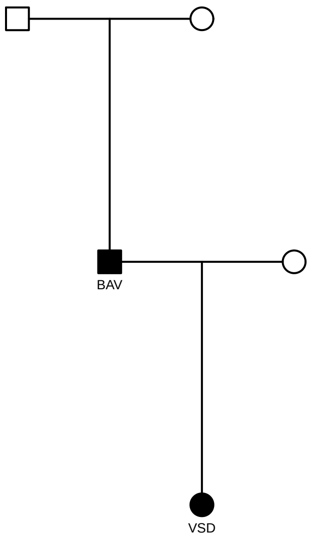

Ellesoe_591

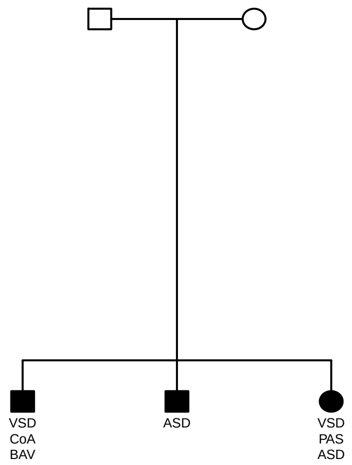

Ellesoe_596

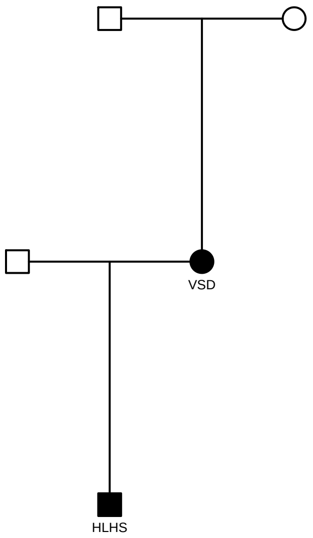

Ellesoe_598

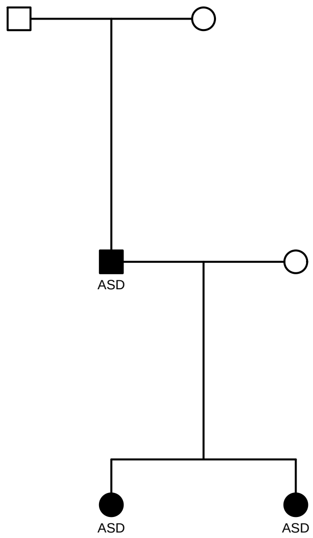

Ellesoe_601

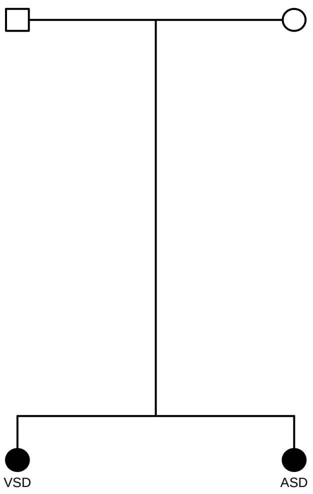

Ellesoe_617

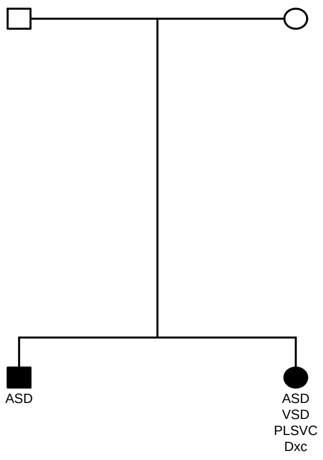

Ellesoe_633

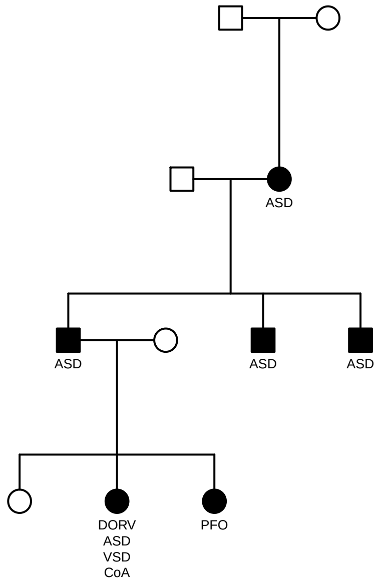

Ellesoe_637

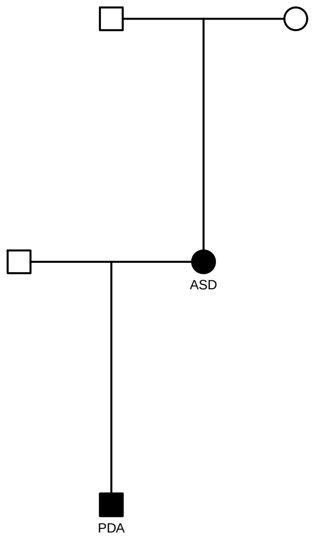

Ellesoe_643

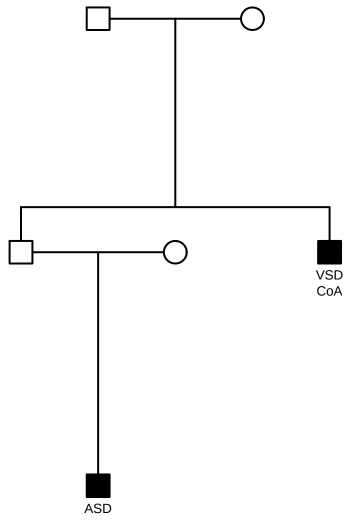

Ellesoe_645

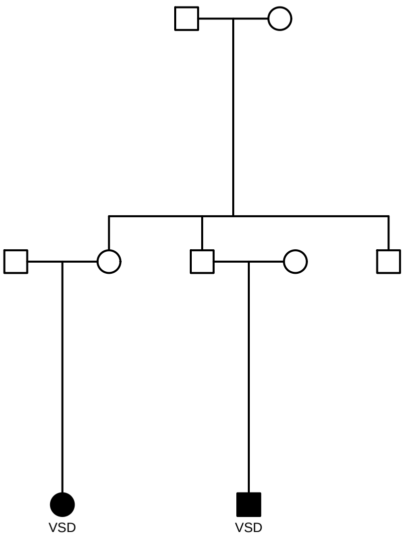

Ellesoe_659

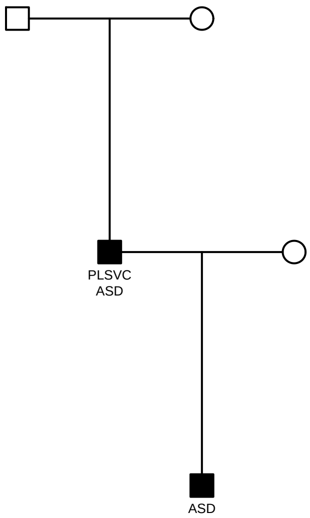

Ellesoe_660

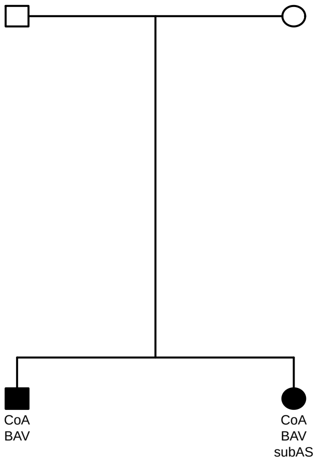

Ellesoe_675

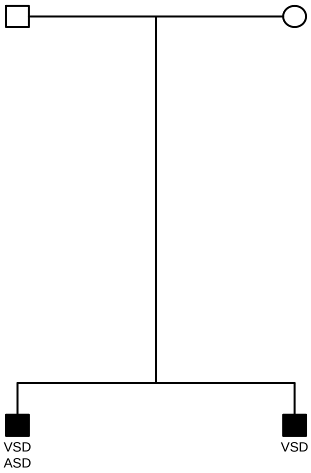

Ellesoe_681

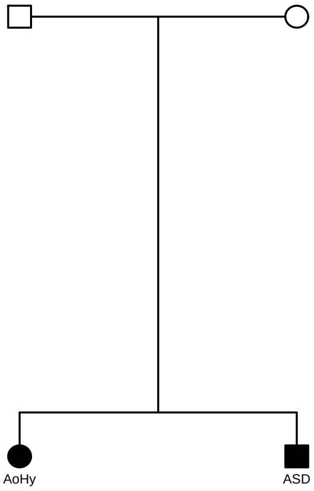

Ellesoe_687

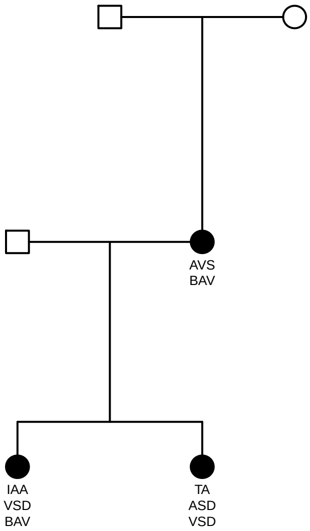

Ellesoe_702

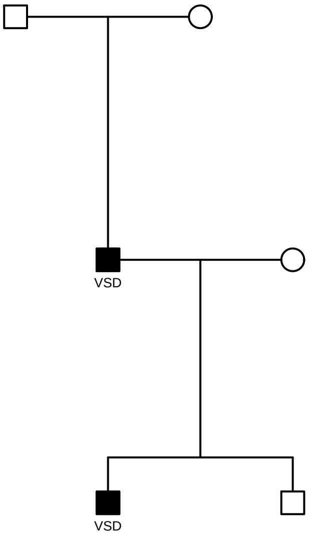

Ellesoe_720

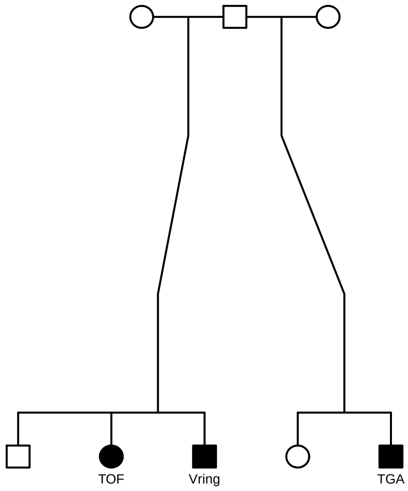

Ellesoe_732

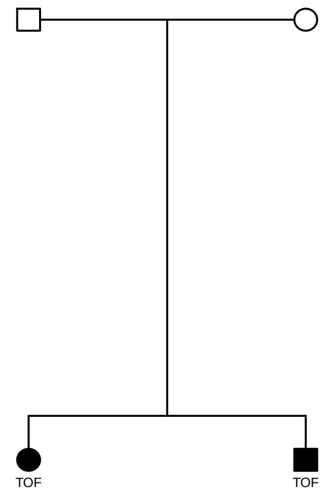

Ellesoe_735

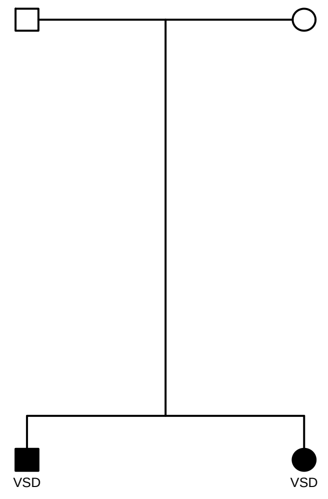

Ellesoe_776

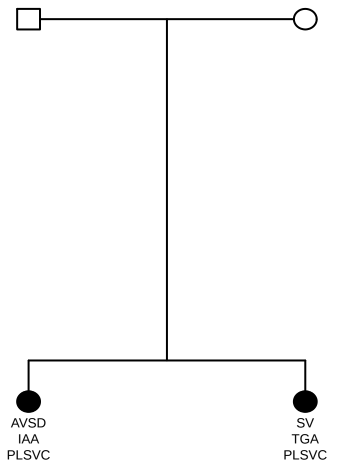

Ellesoe_802

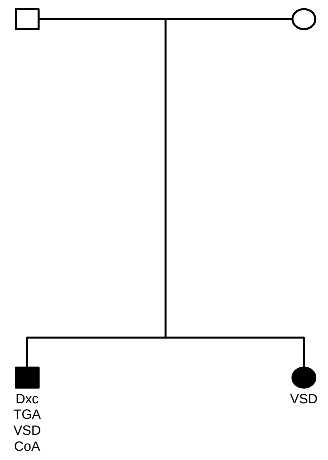

Ellesoe_808

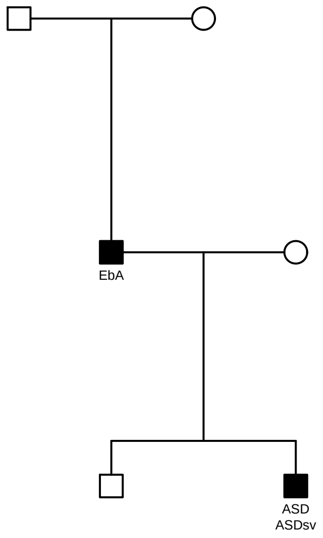

Ellesoe_809

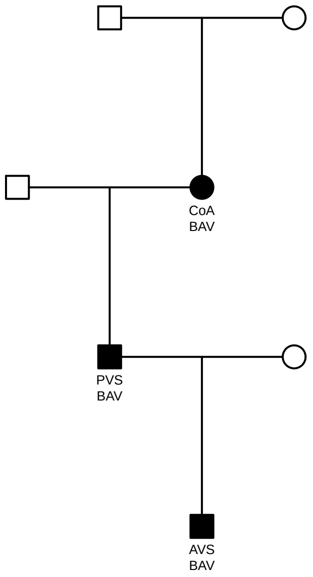

Ellesoe_831

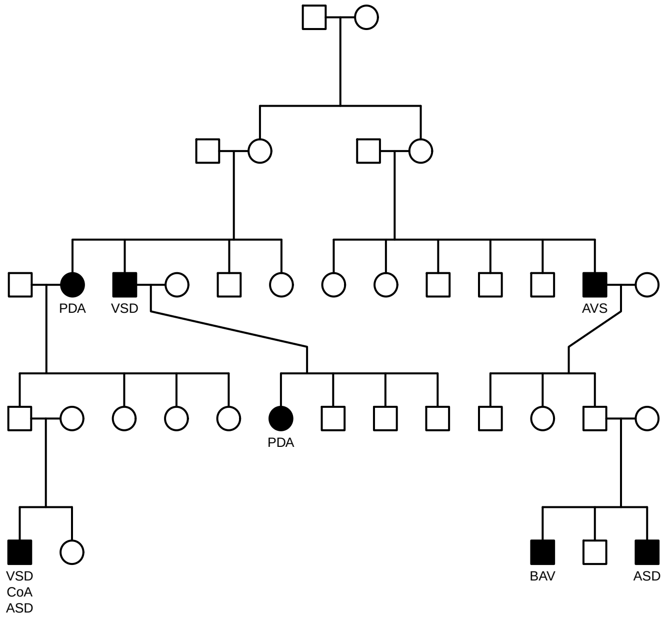

Ellesoe_839

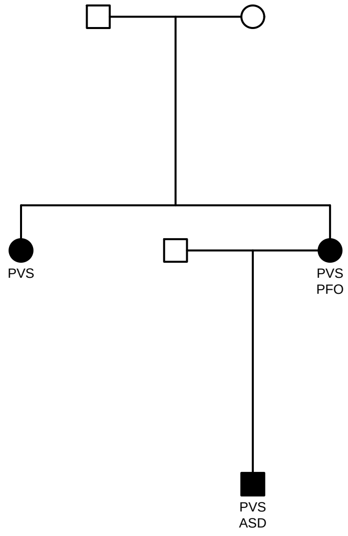

Ellesoe_855

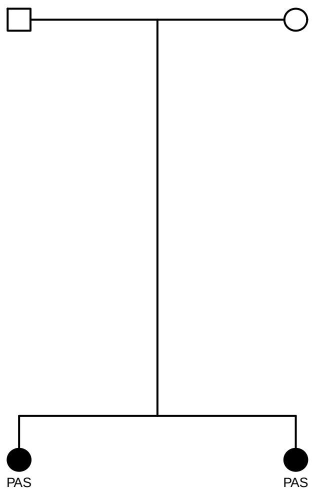

Ellesoe_879

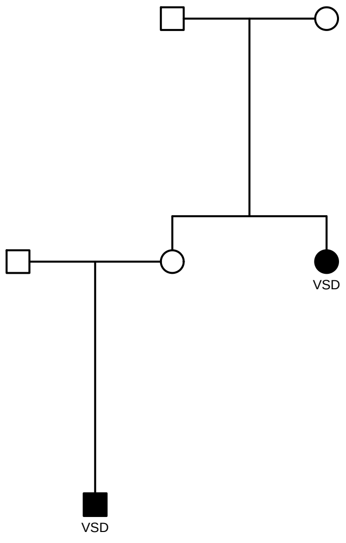

Ellesoe_880

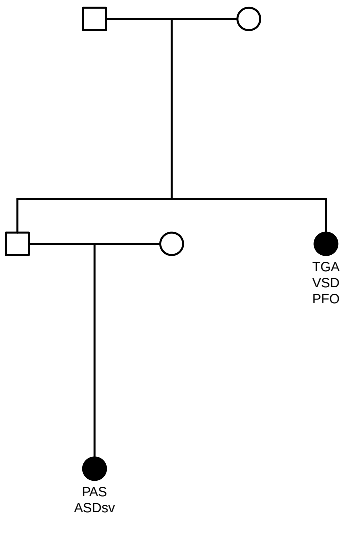

Ellesoe_881

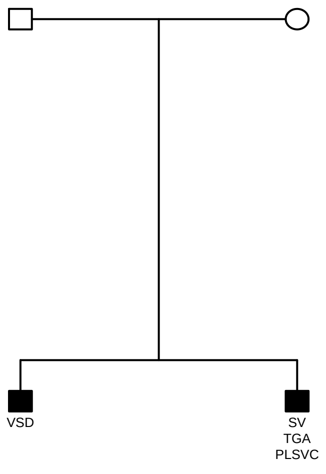

Ellesoe_907

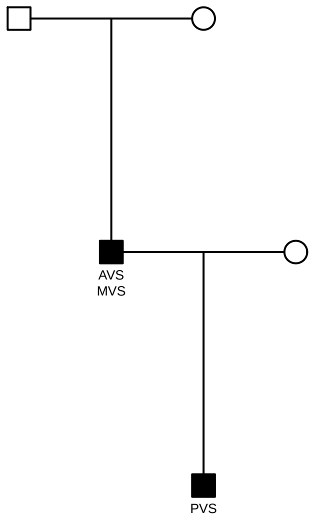

Ellesoe_912

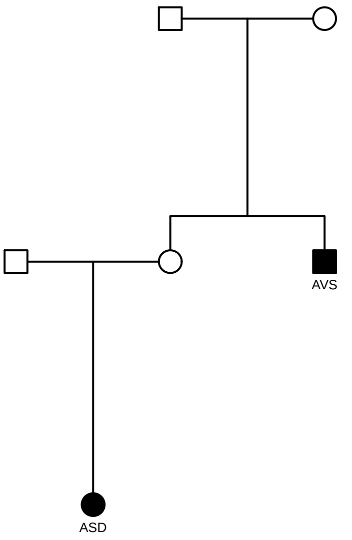

Ellesoe_926

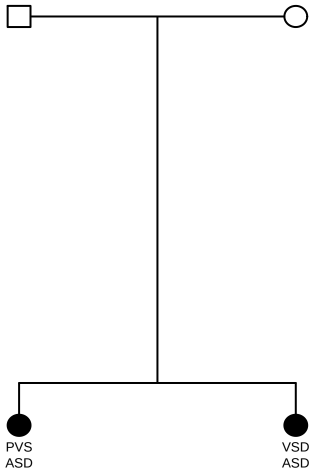

Ellesoe_956

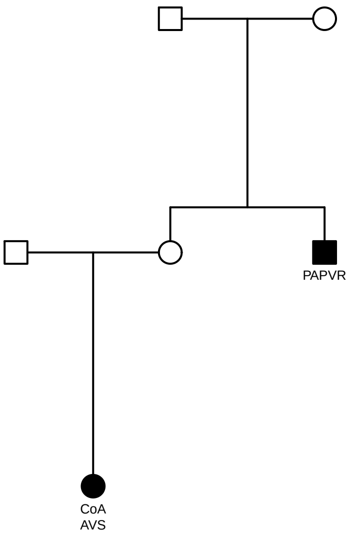

Ellesoe_957

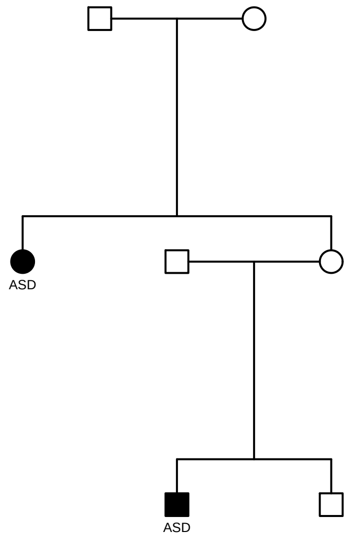

Ellesoe_962

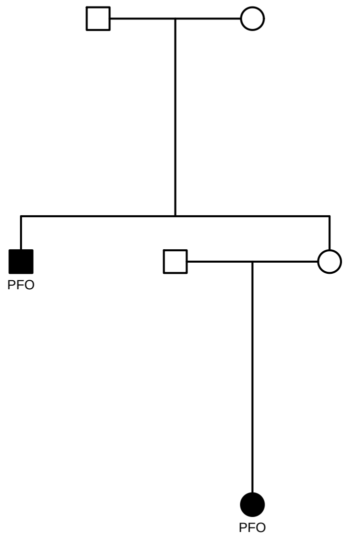

Ellesoe_969

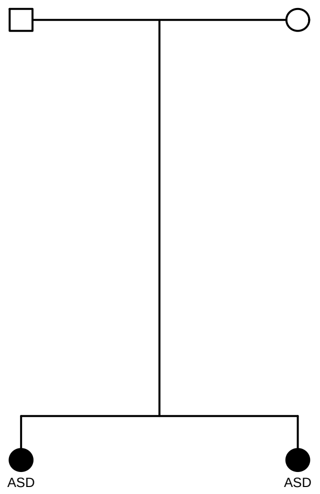

Ellesoe_975

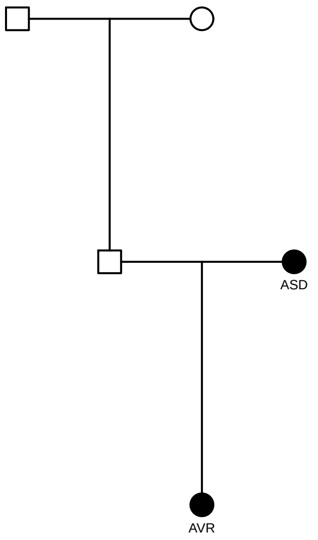

Ellesoe_1006

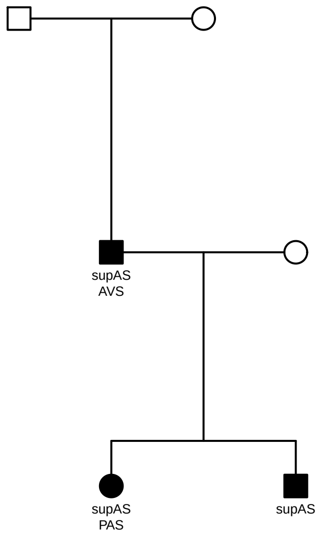

Ellesoe_1016

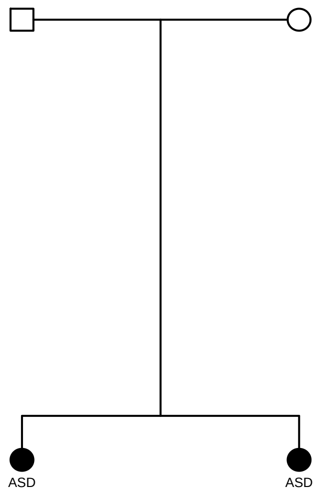

Ellesoe_1019

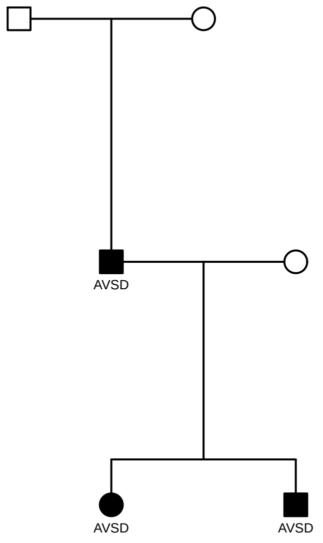

Ellesoe_1027

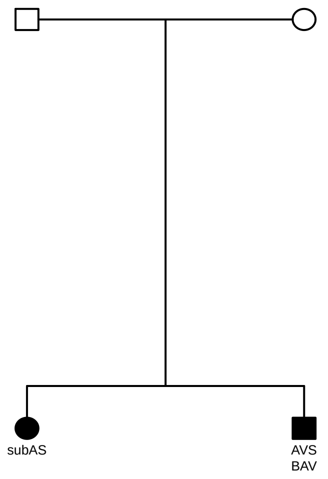

Ellesoe_1028

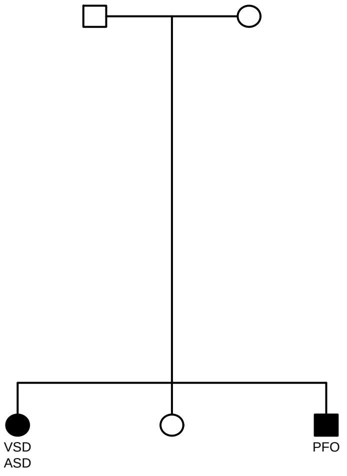

Ellesoe_1056

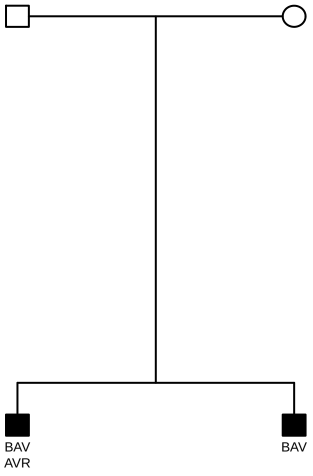

Ellesoe_1061

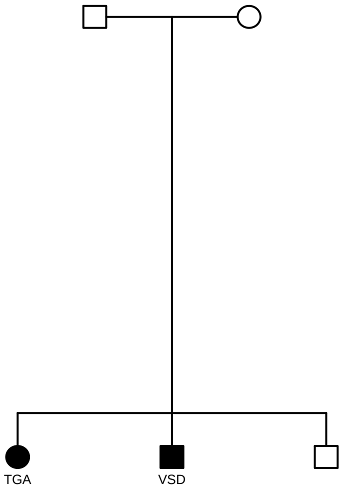

Ellesoe_1077

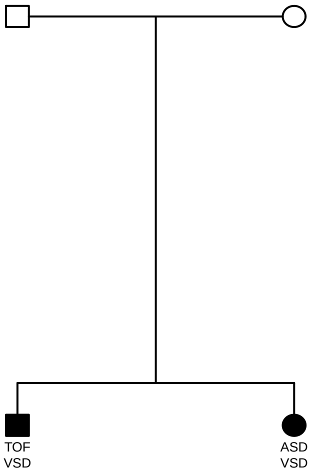

Ellesoe_1090

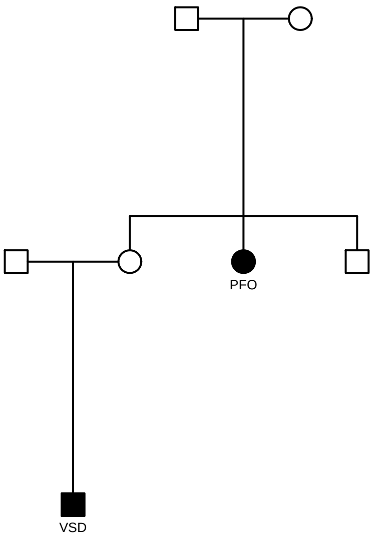

Ellesoe_1093

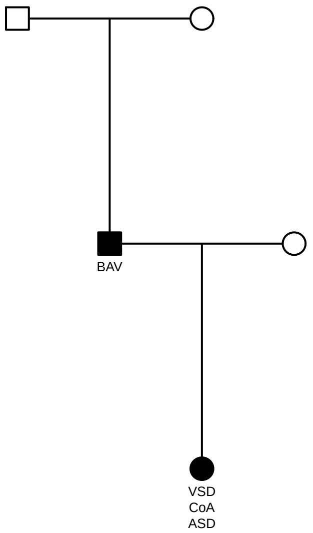

Ellesoe_1099

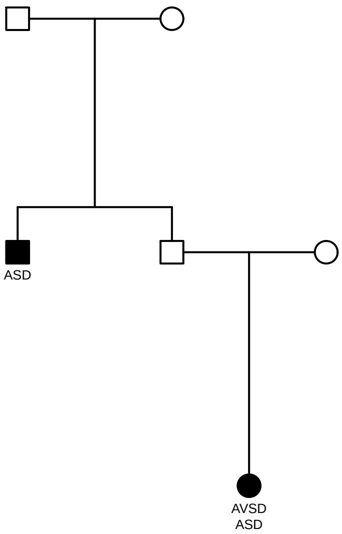

Ellesoe_1117

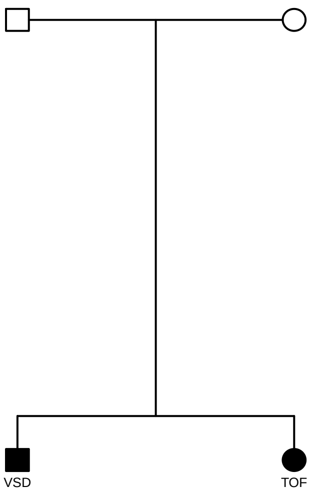

Ellesoe_1121

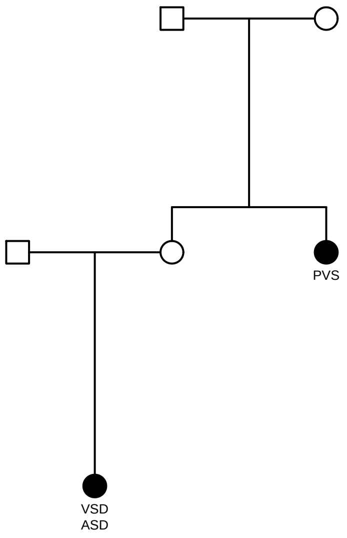

Ellesoe_1151

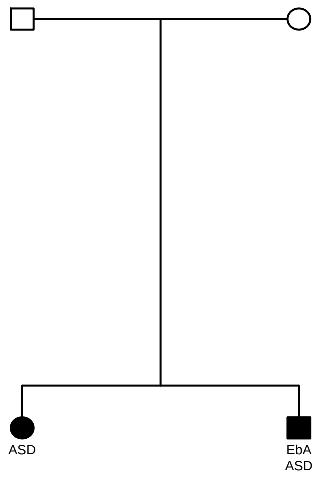

Ellesoe_1164

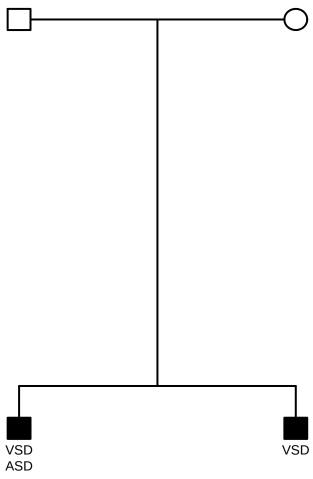

Ellesoe_1166

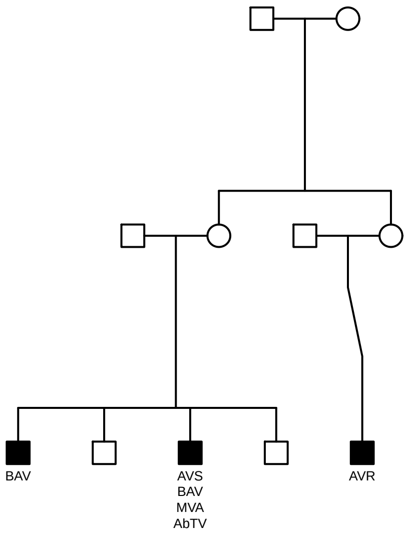

Ellesoe_1236

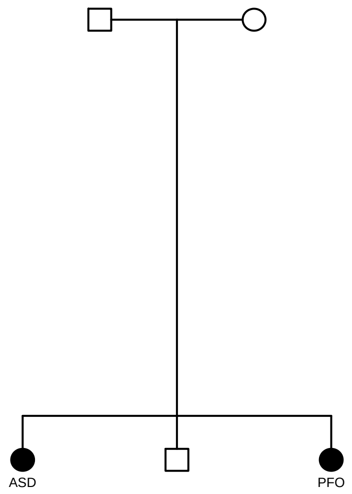

Ellesoe_1244

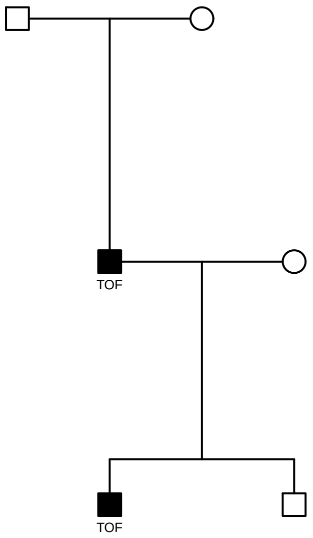

Ellesoe_1254

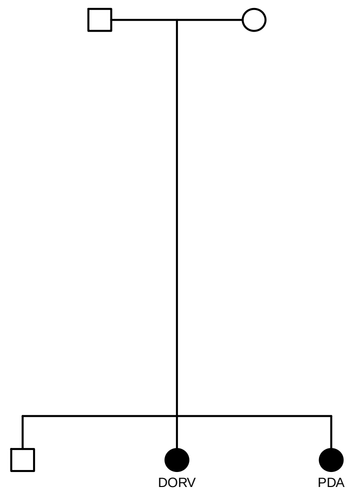

Ellesoe_1260

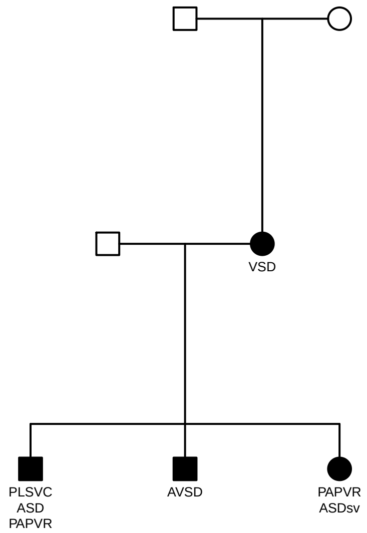

Ellesoe_1273

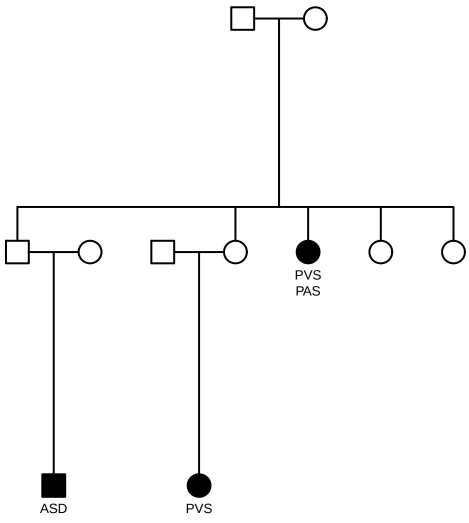

Ellesoe_1275

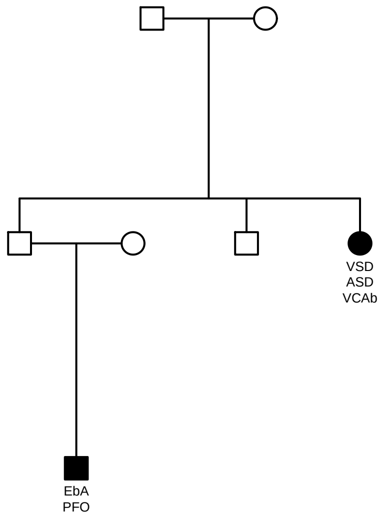

Ellesoe_1319

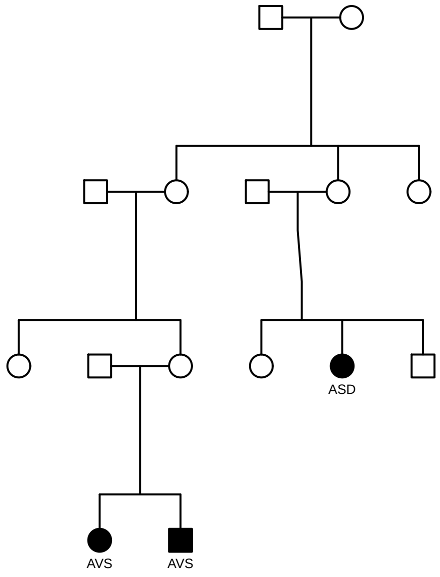

Ellesoe_1349

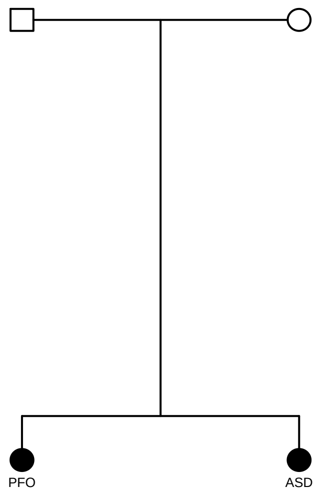

Ellesoe_1361

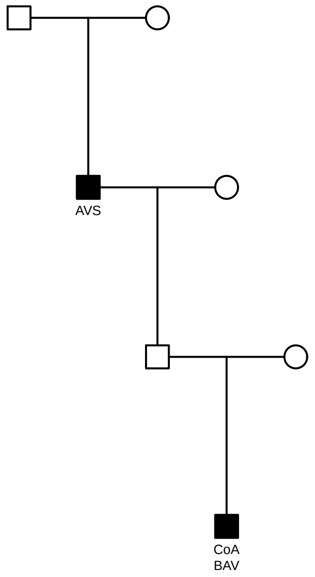

Ellesoe_1364

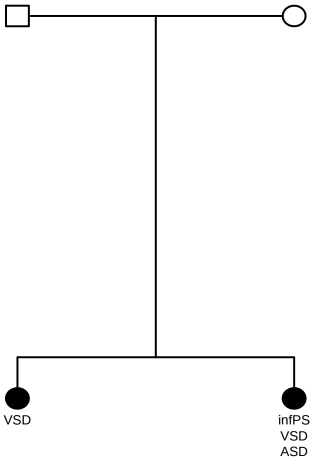

Ellesoe_1382

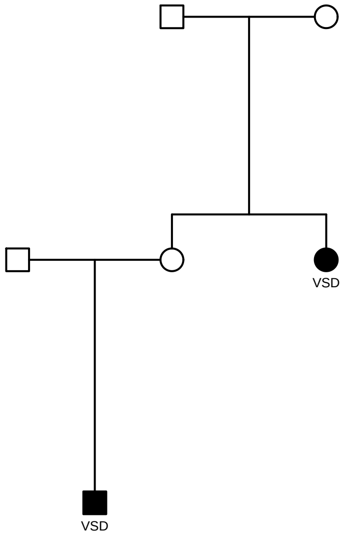

Ellesoe_1387

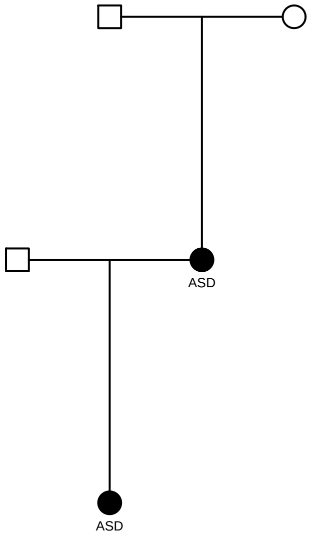

Ellesoe_1560

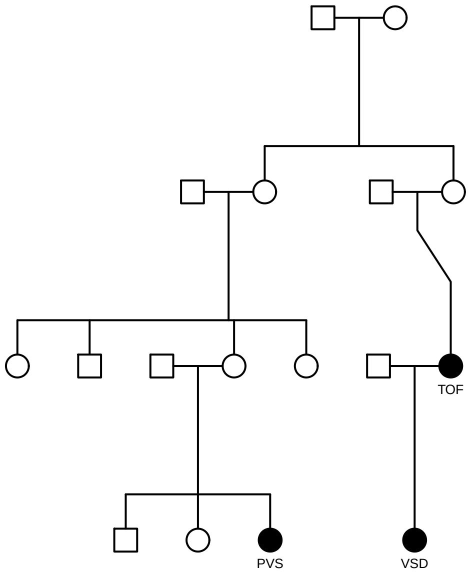

Ellesoe_1575

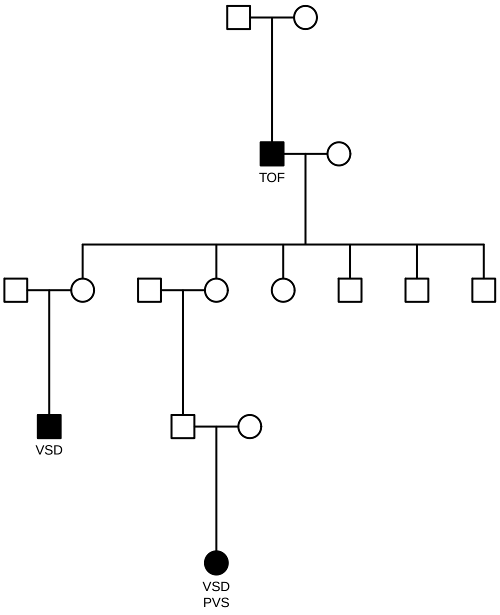

Ellesoe_1579

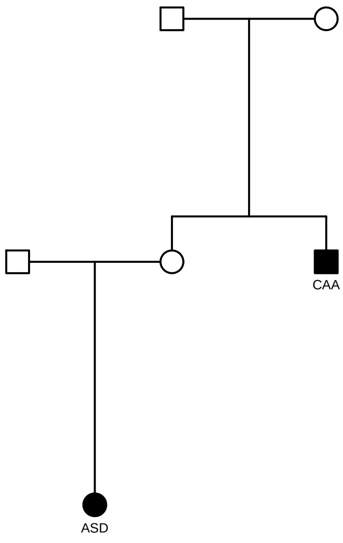

Ellesoe_1586

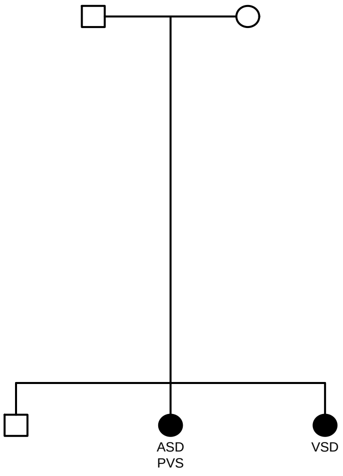

Ellesoe_1600

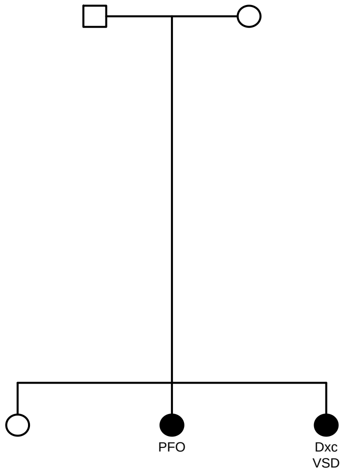

Ellesoe_1710

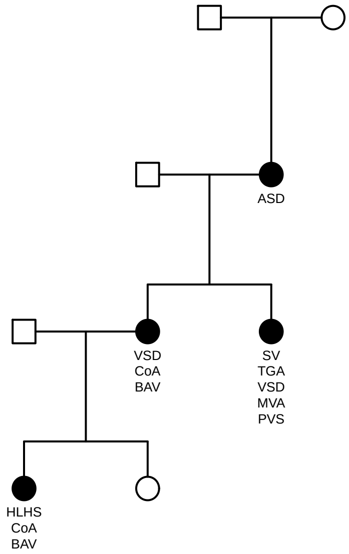

Ellesoe_1722

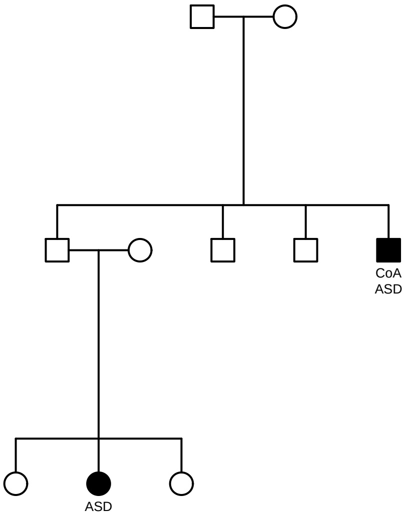

Ellesoe_1743

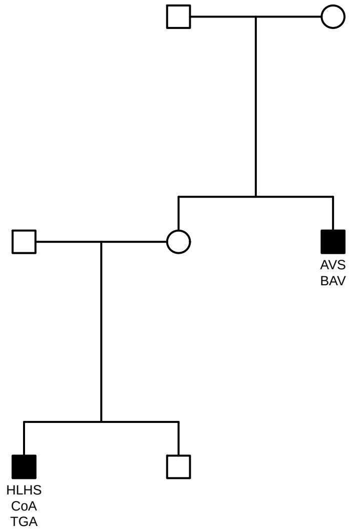

Ellesoe_1752

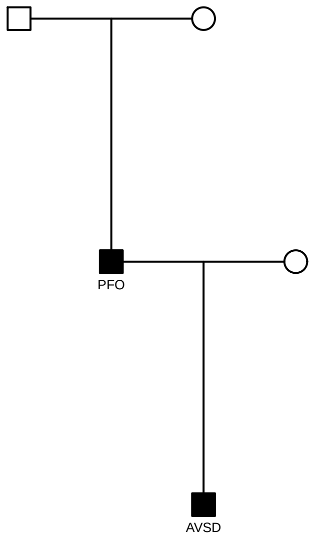

Ellesoe_1790

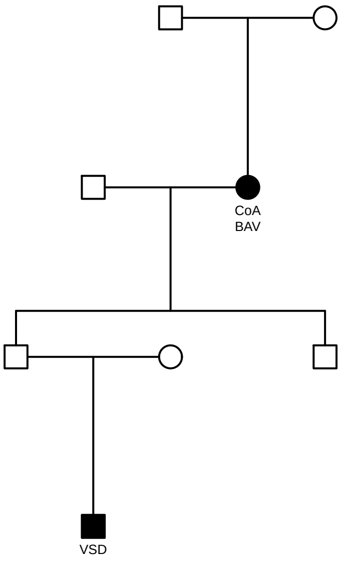

Ellesoe_1813

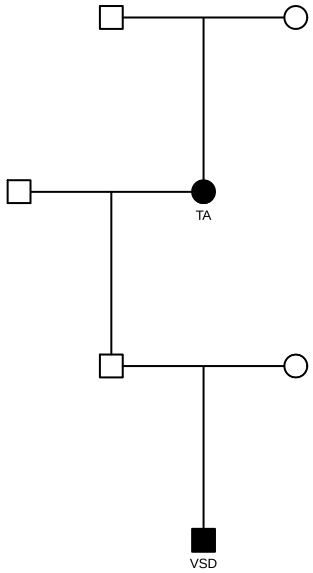

Ellesoe_1817

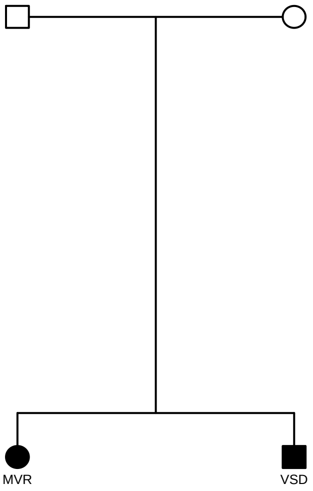

Ellesoe_1870

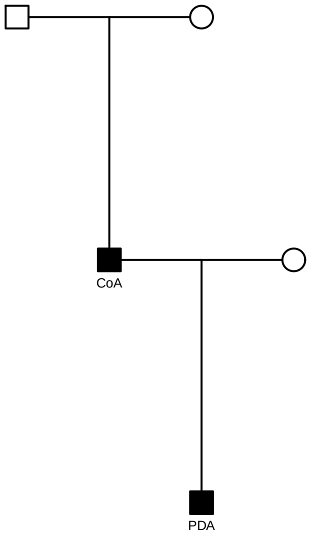

Ellesoe_1909

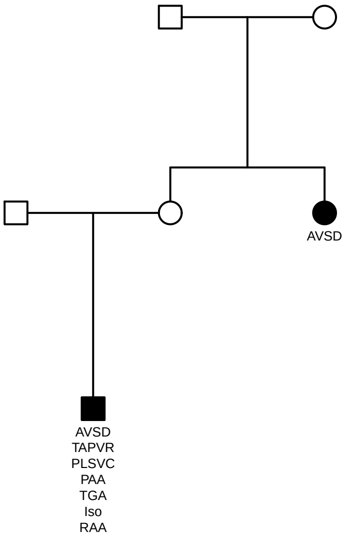

Ellesoe_1938

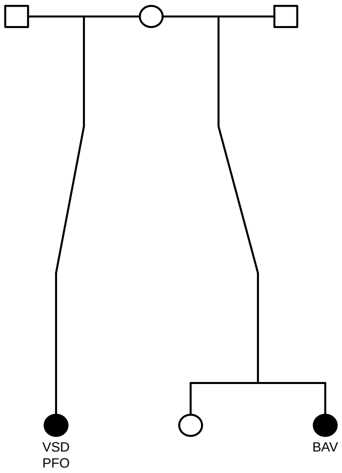

Ellesoe_1973

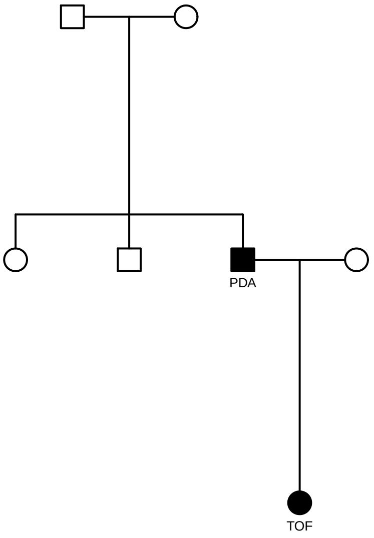

Ellesoe_1996

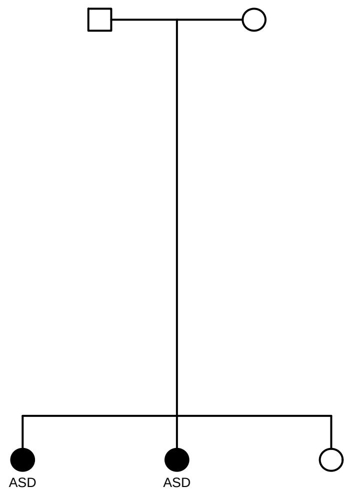

Ellesoe_1997

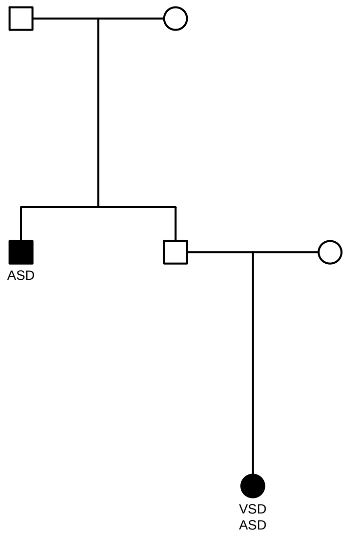

Ellesoe_2036

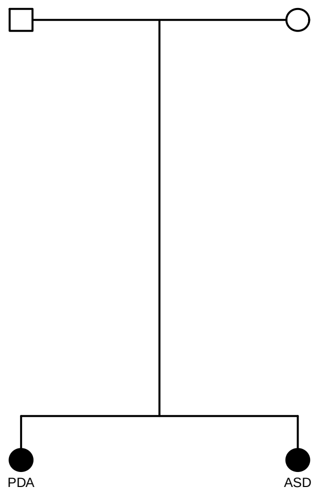

Ellesoe_2077

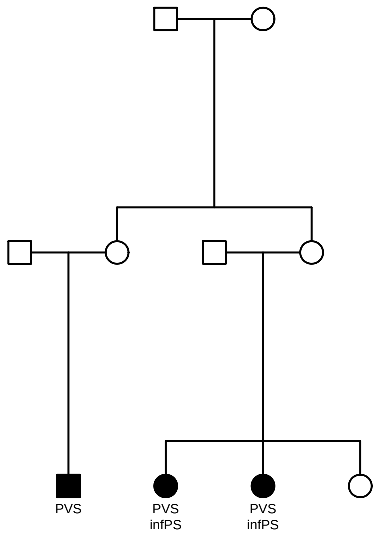

Ellesoe_2163

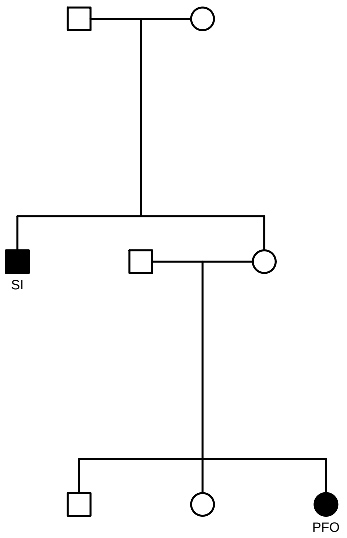

Ellesoe_2169

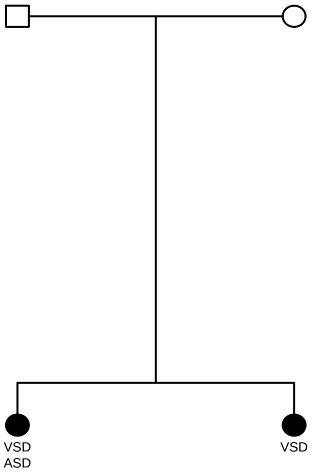

Ellesoe_2174

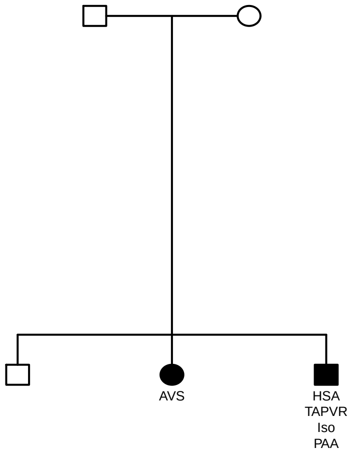

Ellesoe_2198

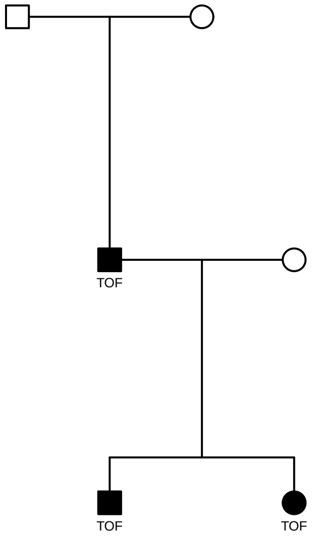

Ellesoe_2261

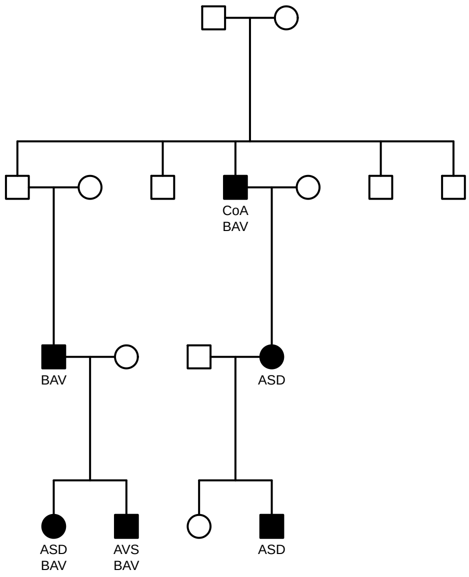

Ellesoe_2265

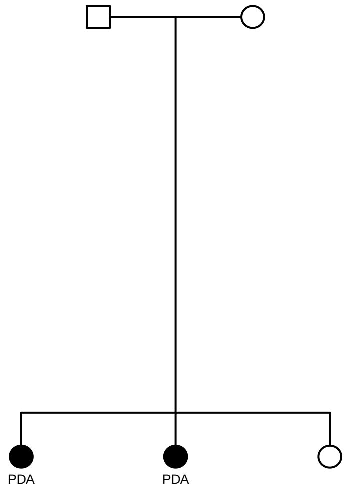

Ellesoe_2273

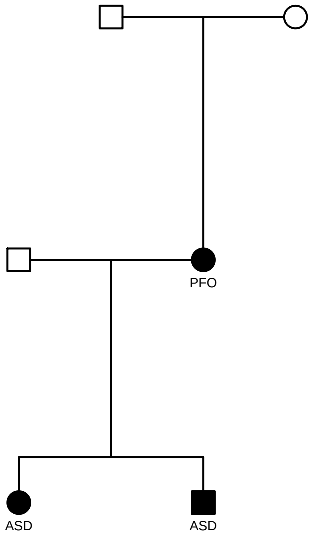

Ellesoe_2398

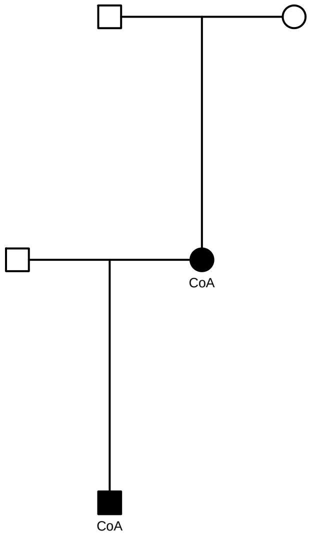

Ellesoe_2425

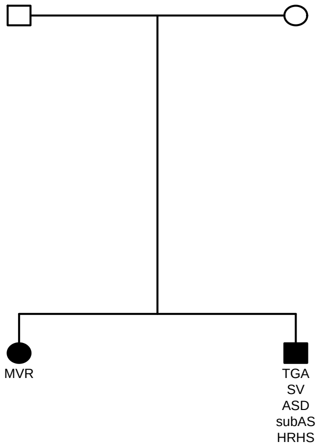

Ellesoe_2441

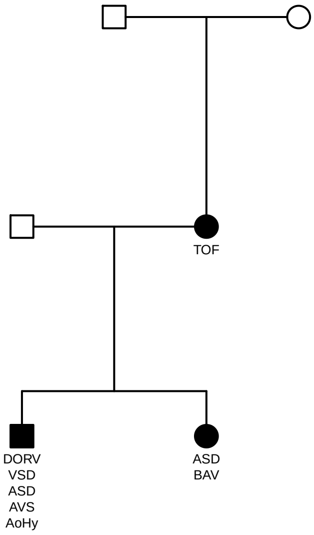

Ellesoe_2446

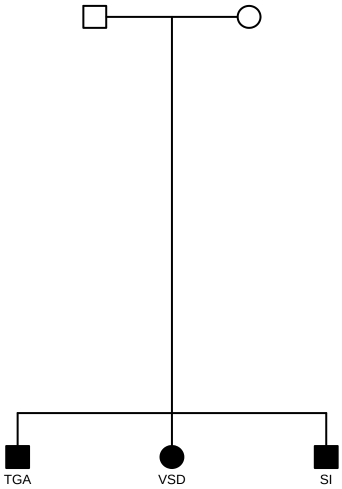

Ellesoe_2474

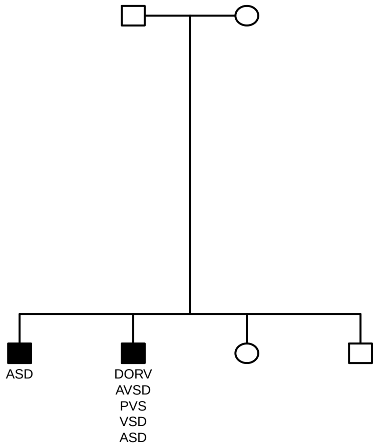

Ellesoe_2510

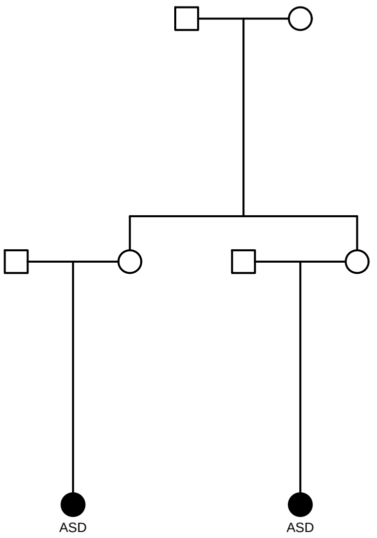

Ellesoe_2558

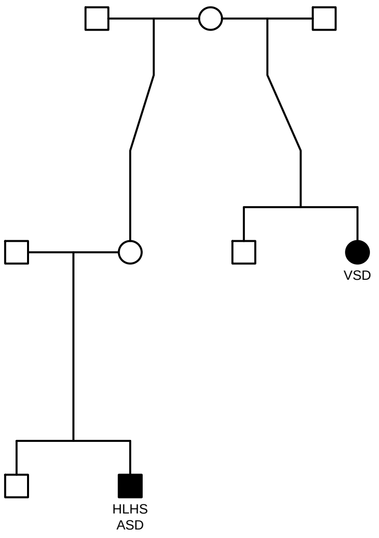

Ellesoe_2641

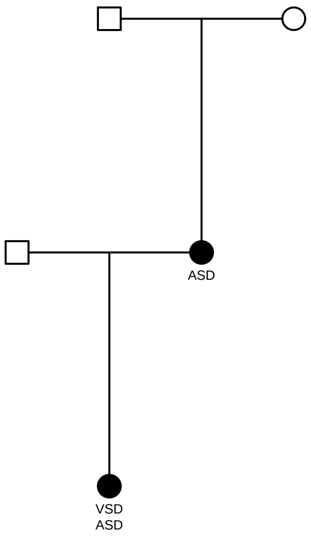

Ellesoe_2649

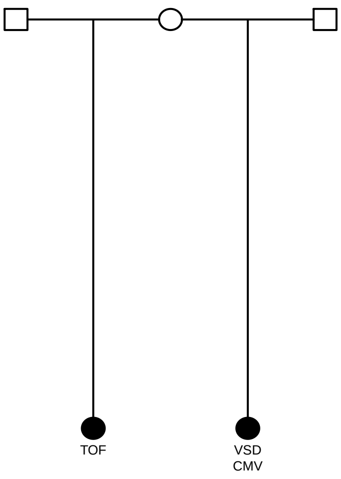

Ellesoe_2701

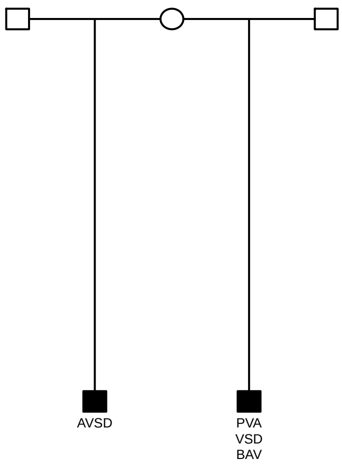

Ellesoe_2748

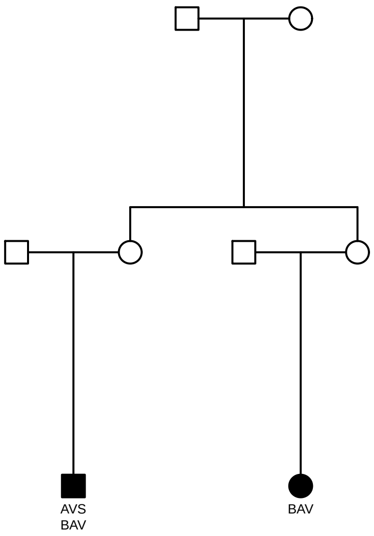

Ellesoe_2750

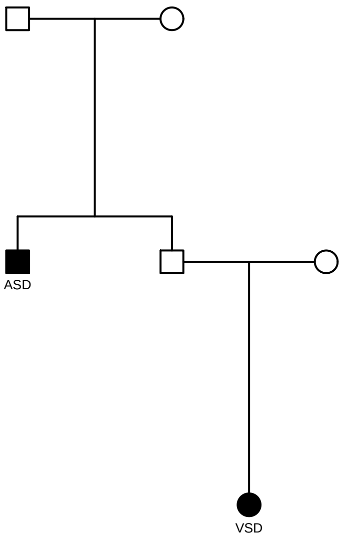

Ellesoe_2853

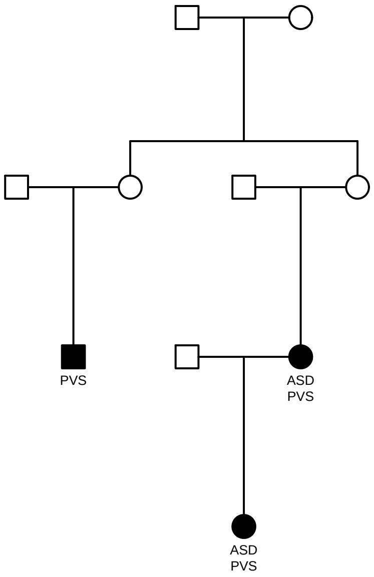

Ellesoe_2875

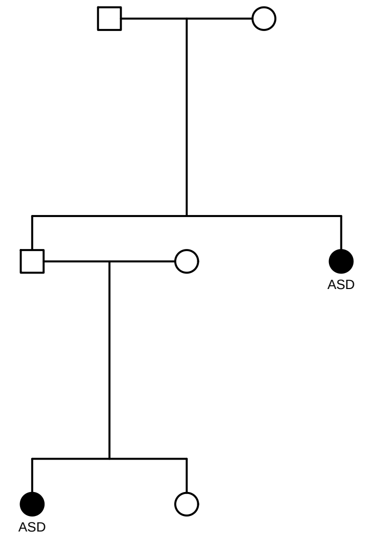

Ellesoe_2893

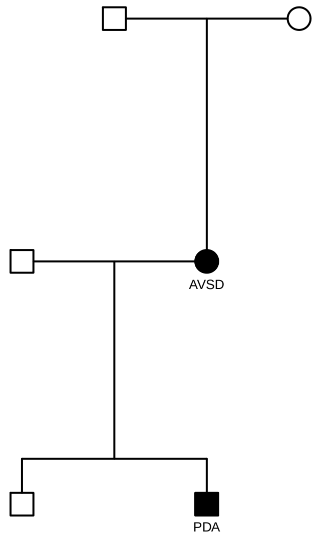

Ellesoe_3305

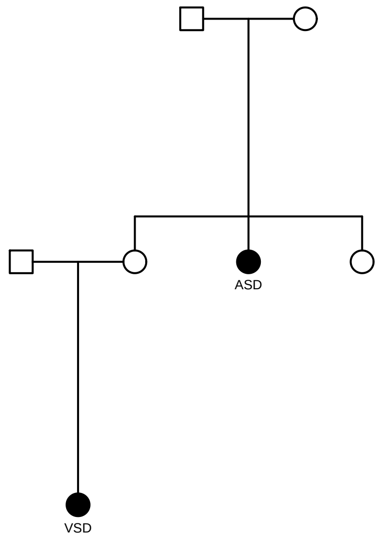

Ellesoe_3315

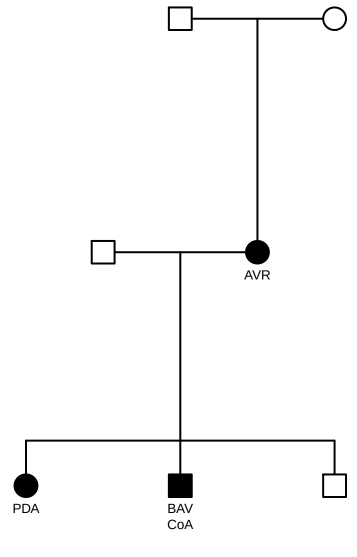

Ellesoe_3344

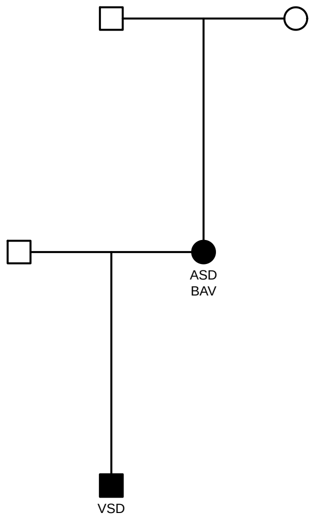

Ellesoe_3352

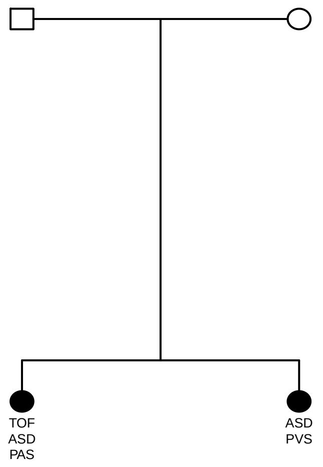

Ellesoe_3364

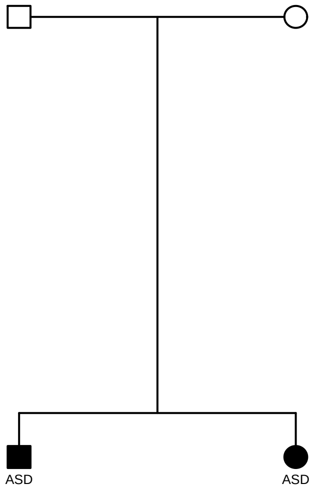

Ellesoe_3369

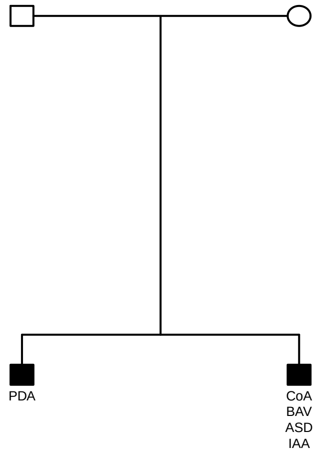

Ellesoe_3378

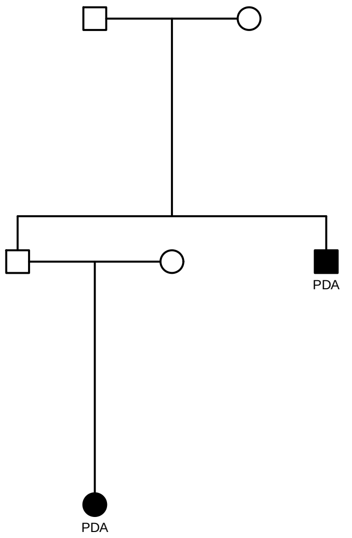

Ellesoe_3500

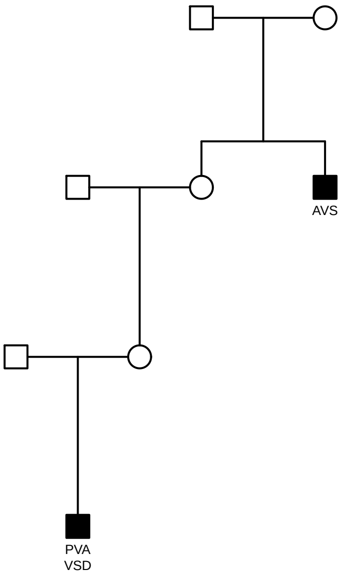

Ellesoe_3501

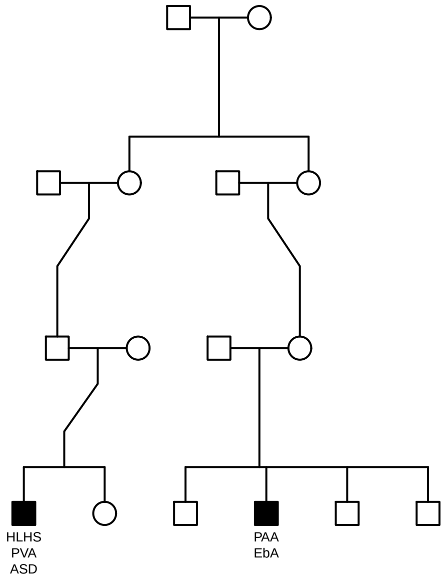

Ellesoe_3503

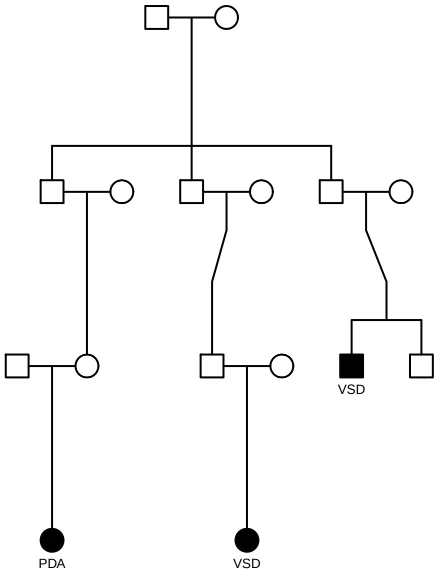

Ellesoe_3505

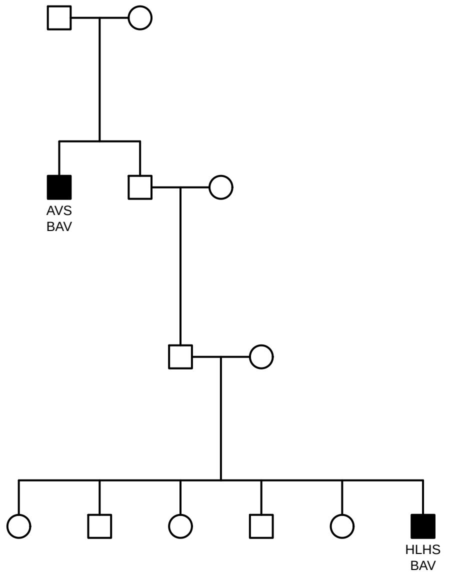

Ellesoe_3506

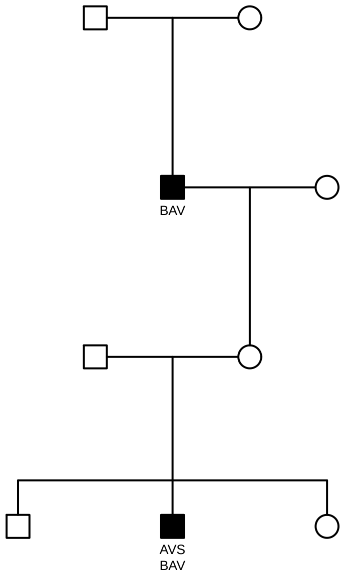

Ellesoe_3508

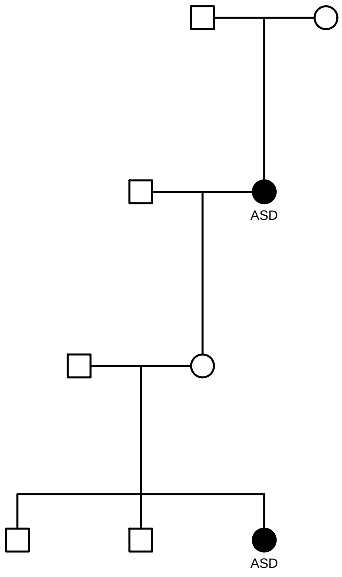

Ellesoe_3540

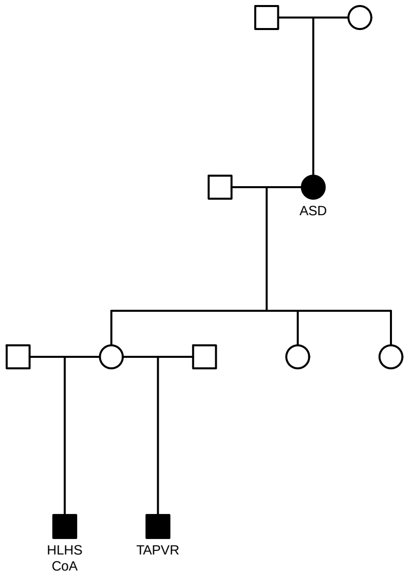

LYS_Z026

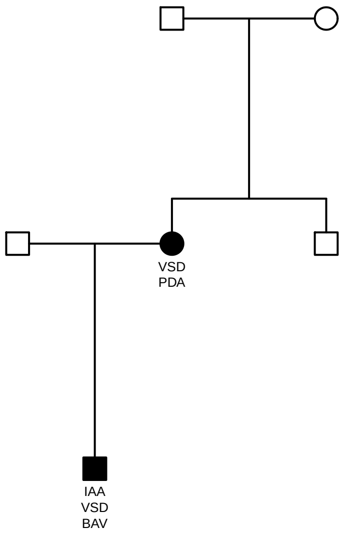

LYS_Z028

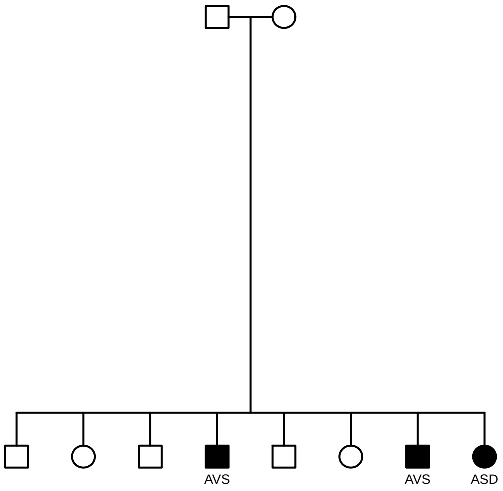

LYS_Z029

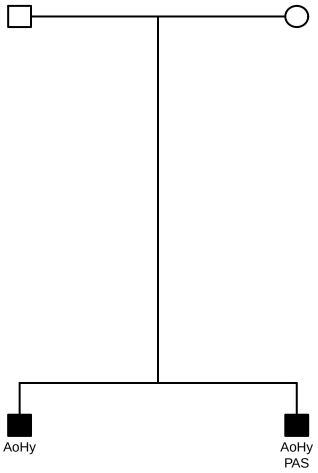

LYS_Z032

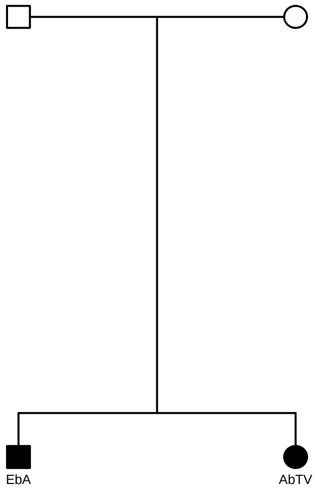

LYS_Z033

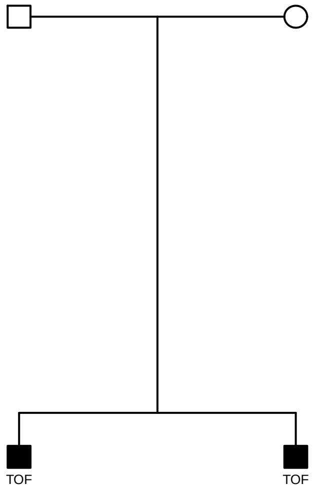

LYS_Z036

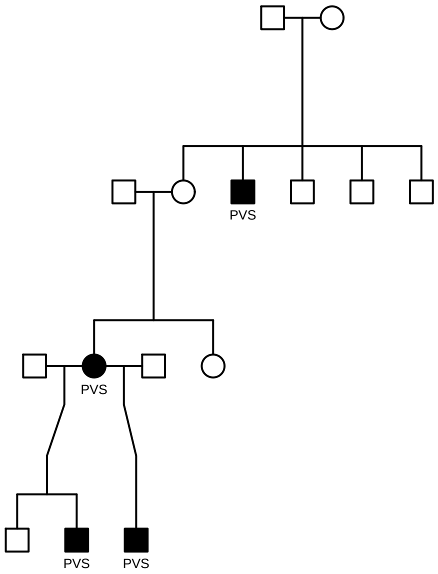

LYS_Z039

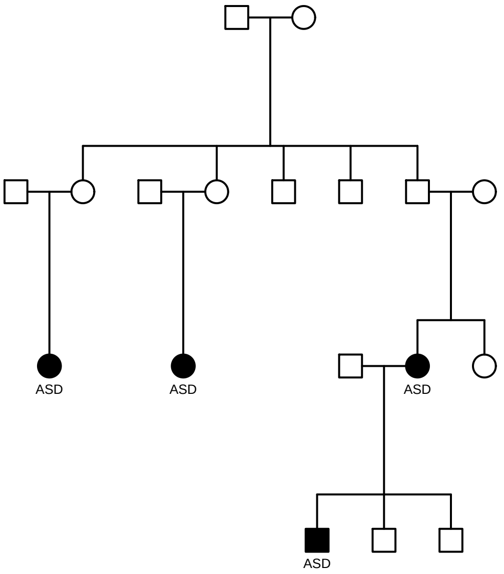

LYS_Z040

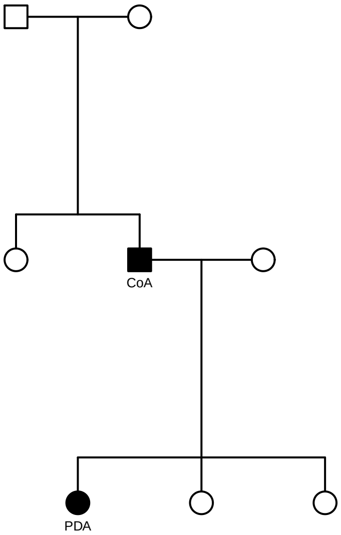

LYS_Z045

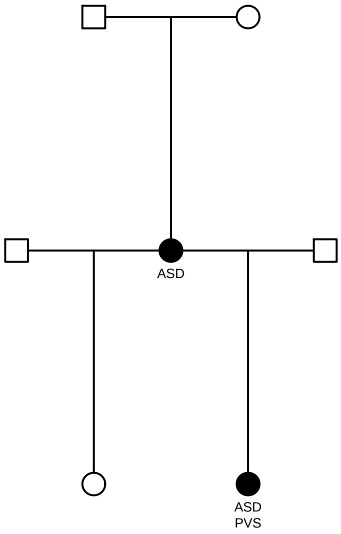

LYS_Z059

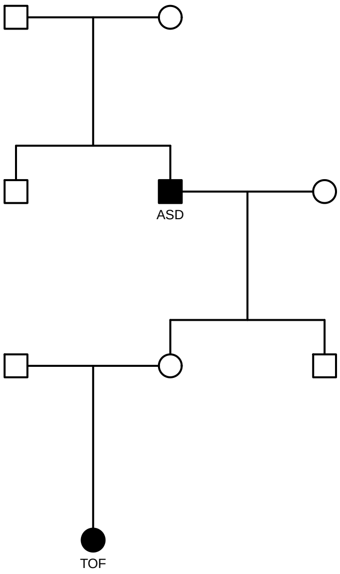

LYS_Z060

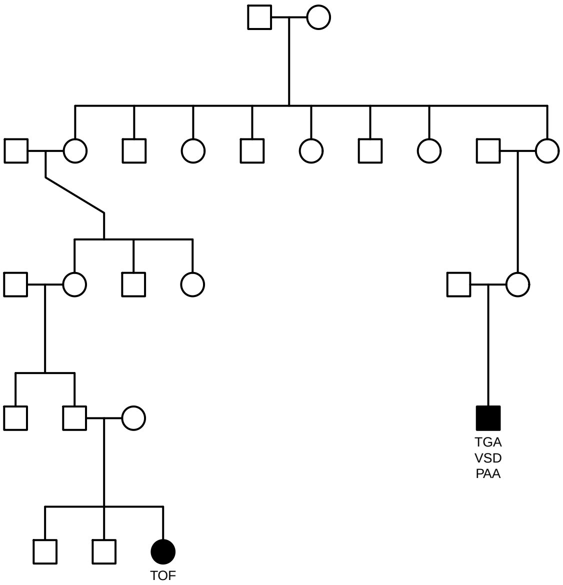

LYS_Z067

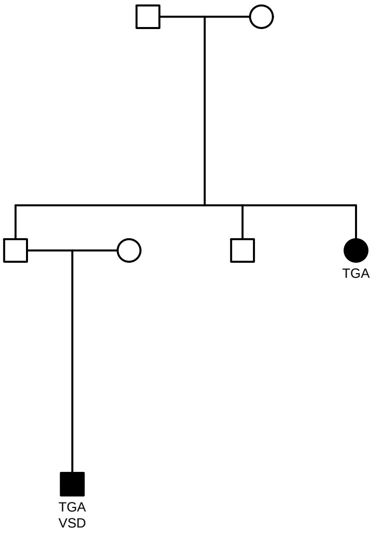

LYS_Z076

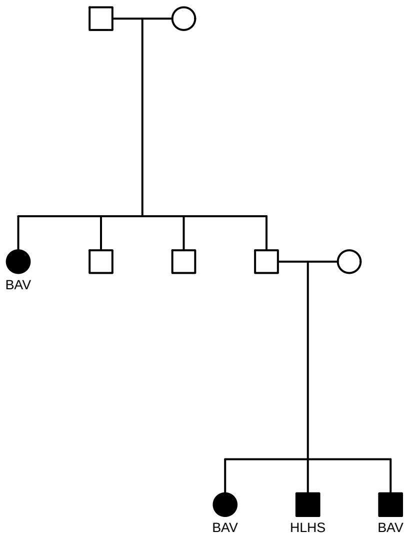

LYS_Z077

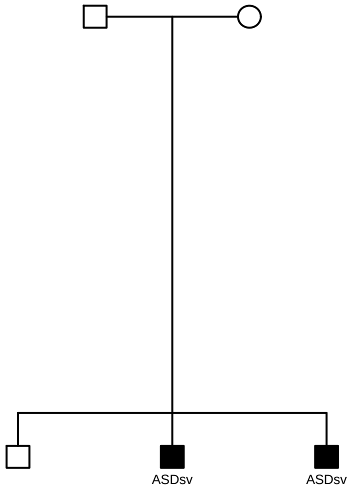

LYS_Z094

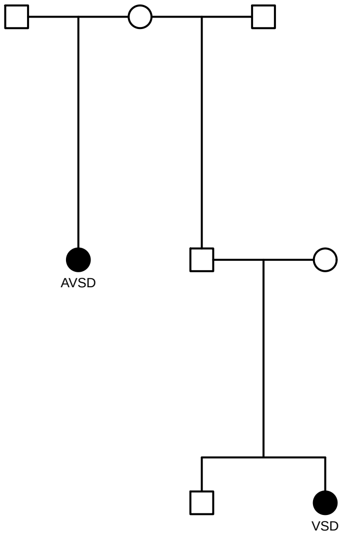

LYS_ZC103

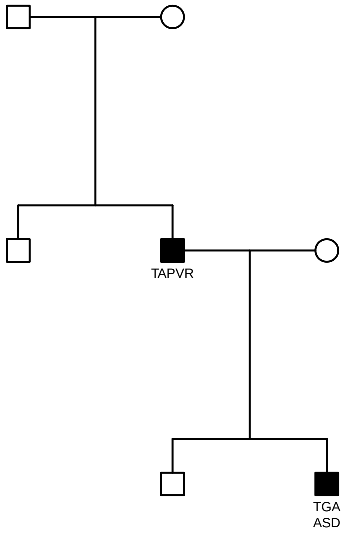

LYS_Z107

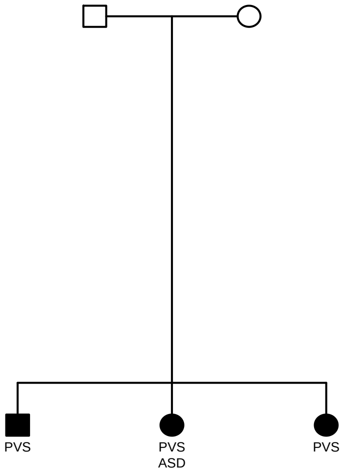

LYS_Z114

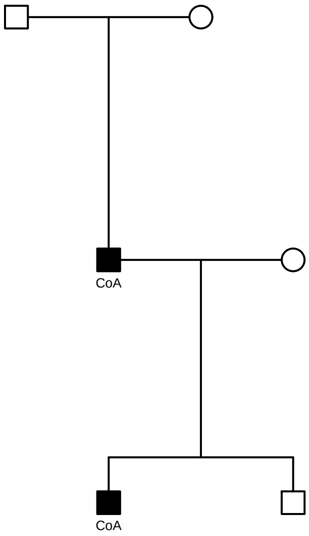

LYS_Z124

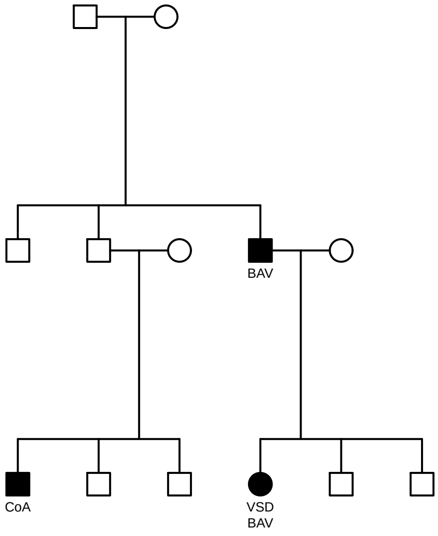

LYS_Z129

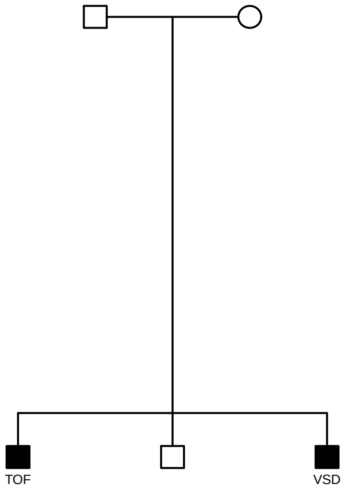

LYS_Z133

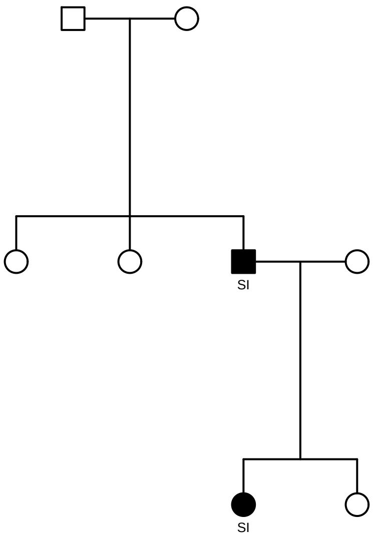

LYS_Z135

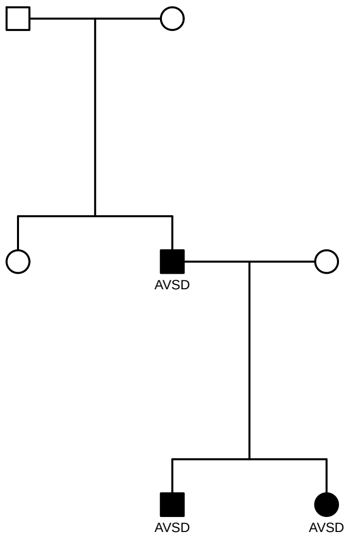

LYS_Z136

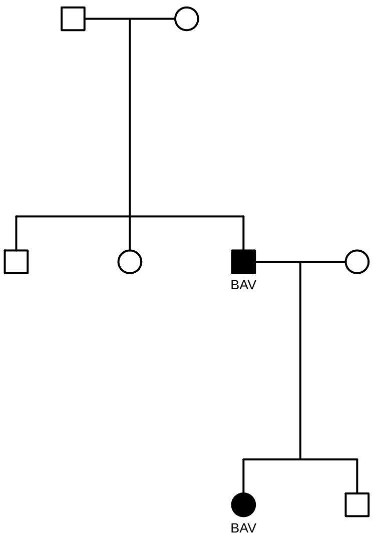

LYS_Z141

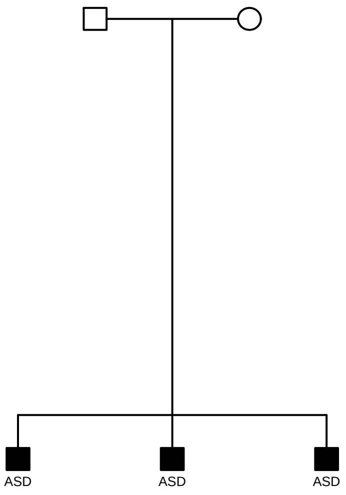

LYS_Z142

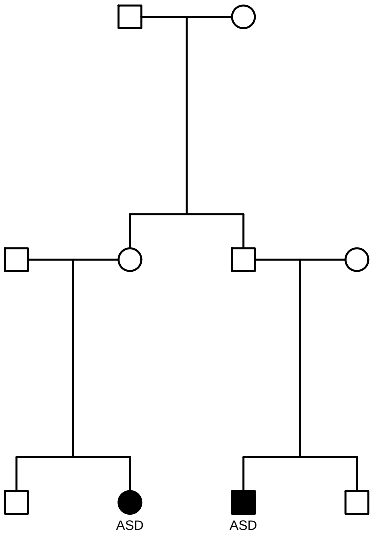

LYS_Z150

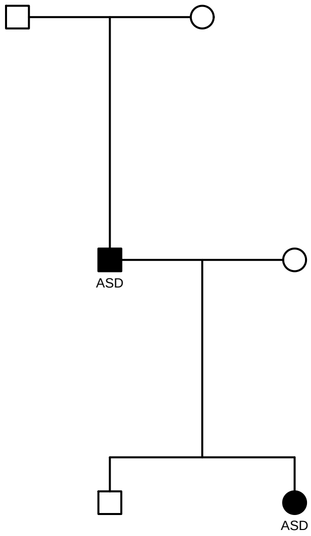

LYS_Z152

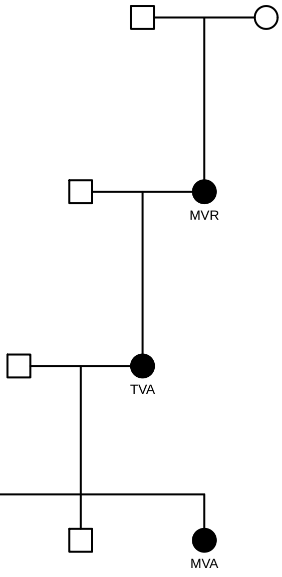

LYS_Z167

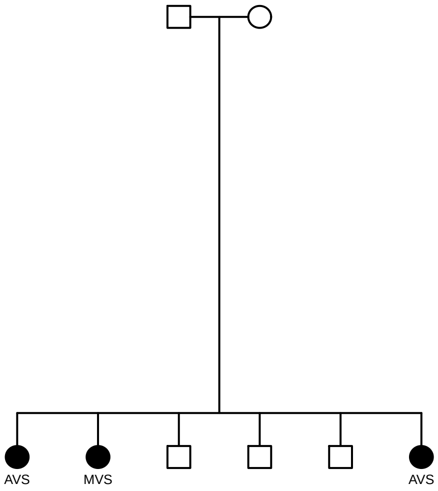

LYS_Z170

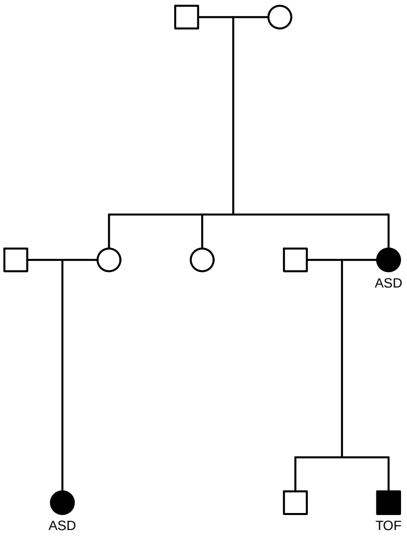

LYS_Z178

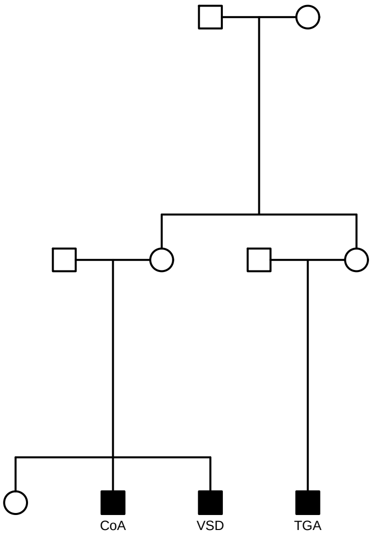

LYS_Z179

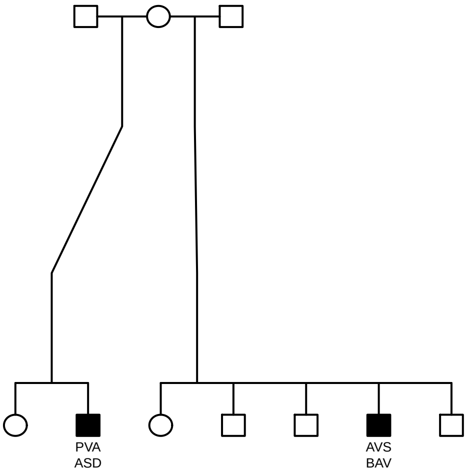

LYS_Z184

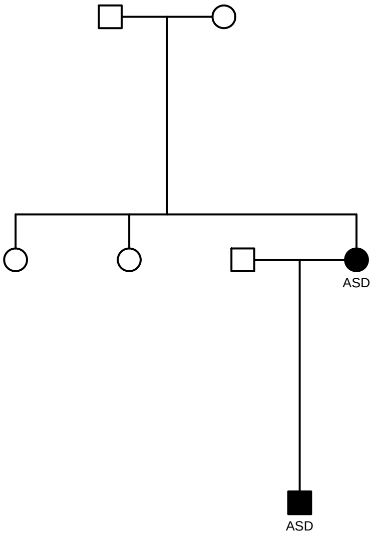

LYS_Z200

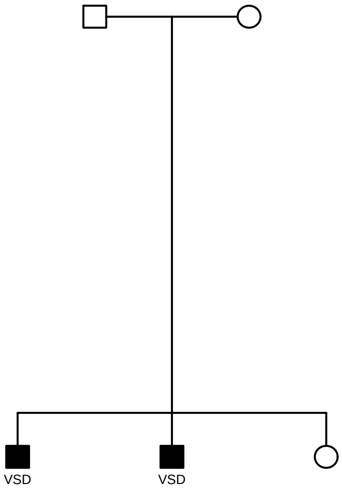

LYS_Z201

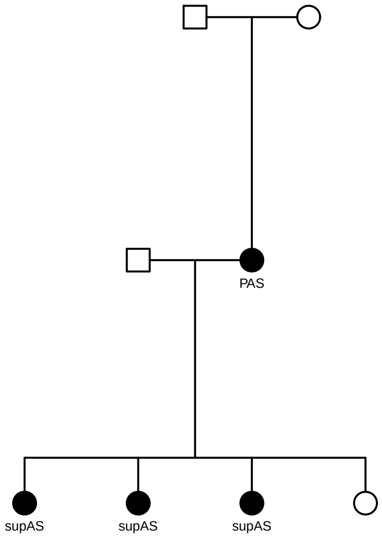

LYS_Z202

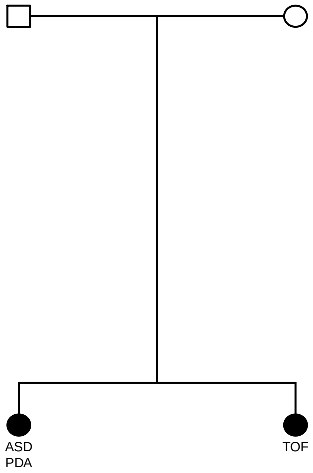

LYS_Z203

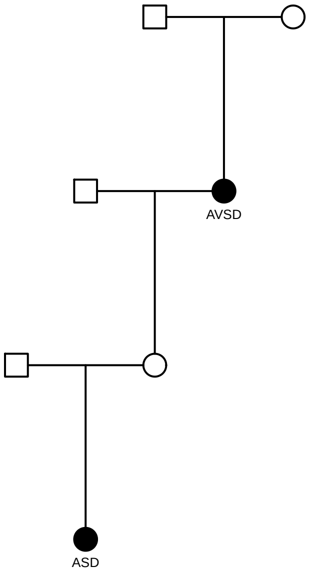

LYS_Z204

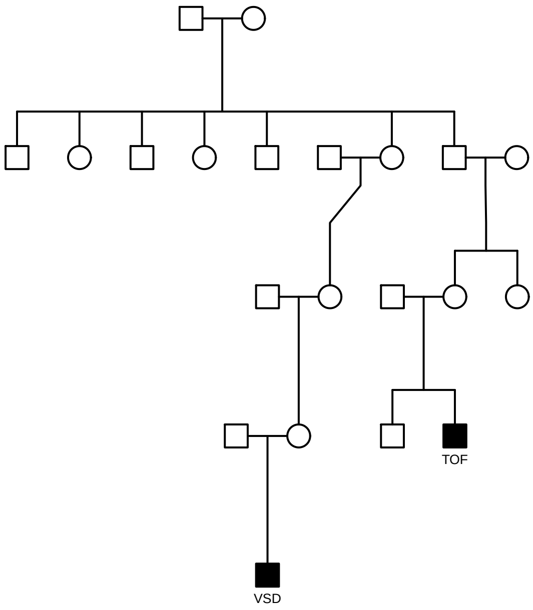

LYS_Z205

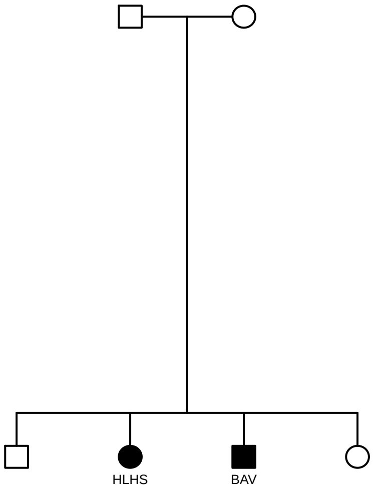

LYS_Z206

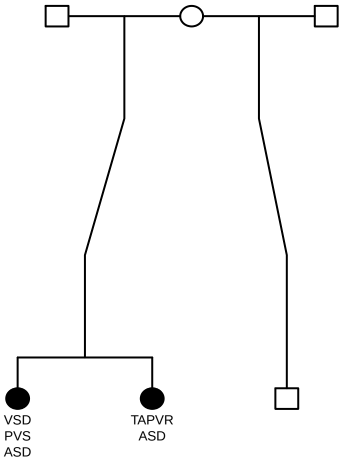

LYS_Z209

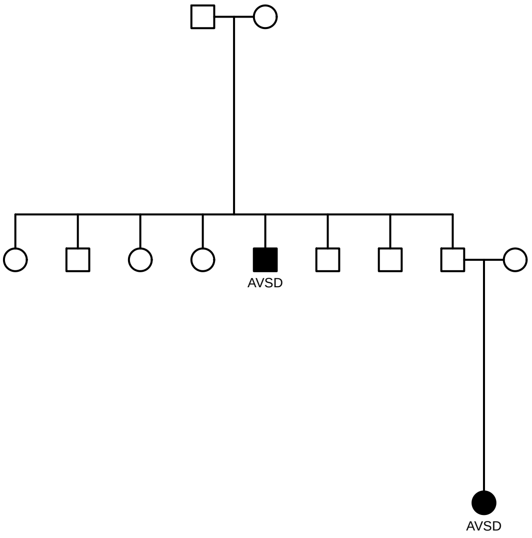

LYS_S003

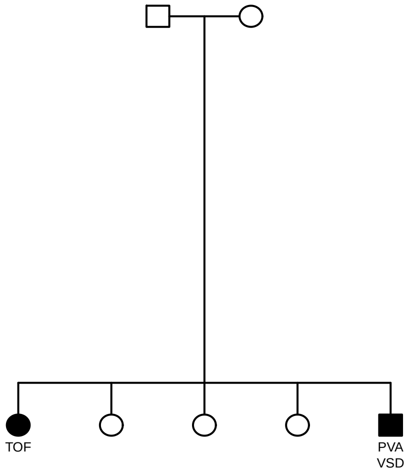

LYS_S020

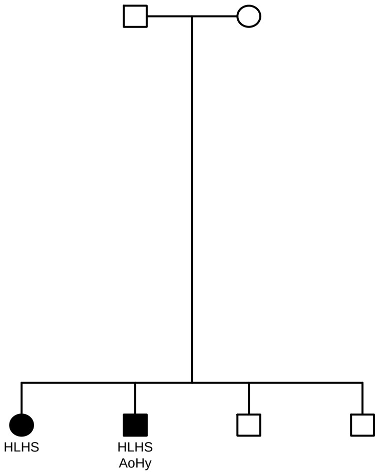

LYS_S023

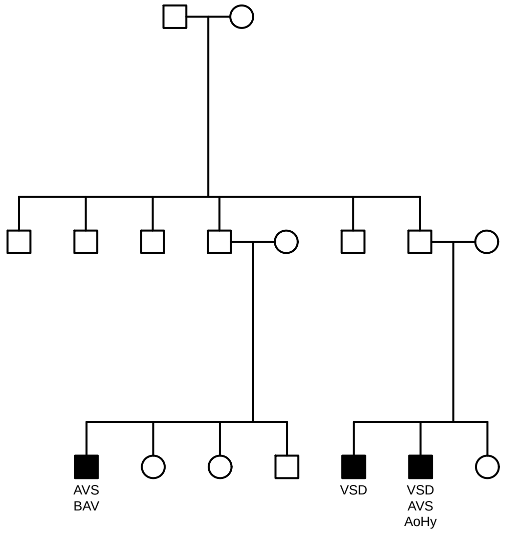

LYS_S049

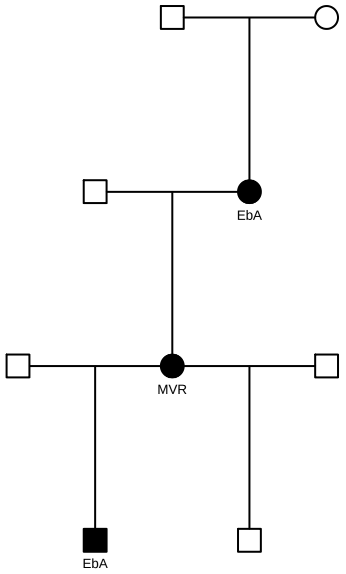

LYS_S071

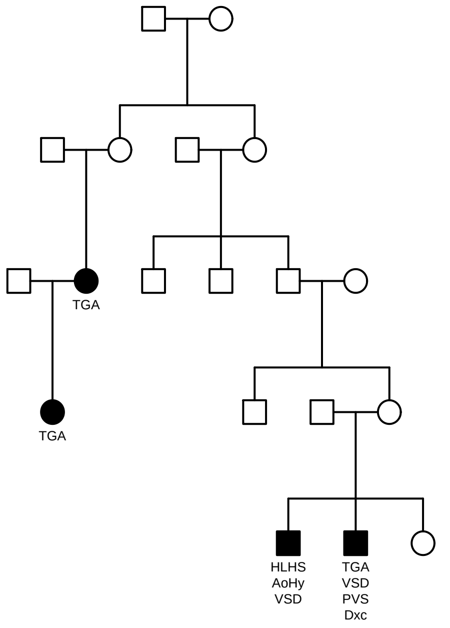

LYS_S144

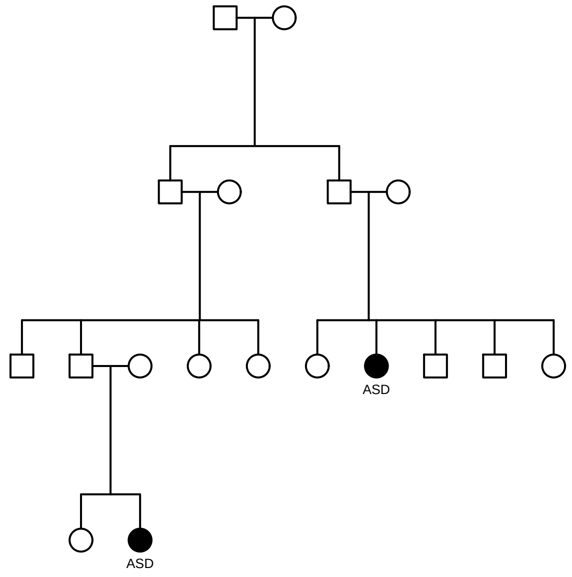

LYS_S149

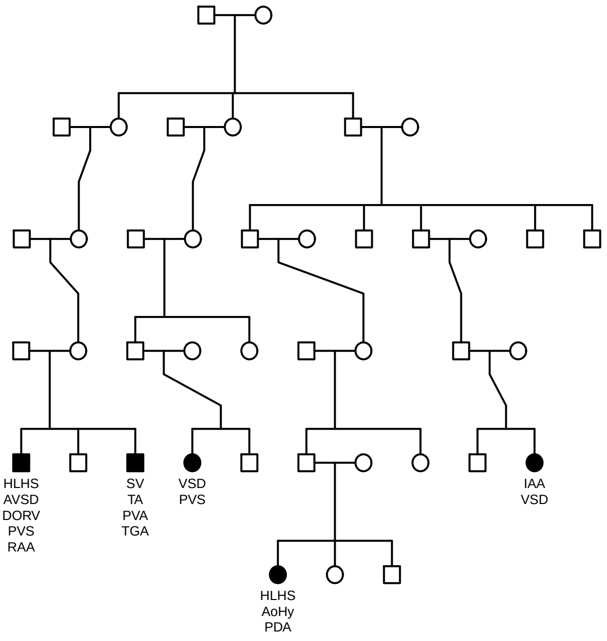

LYS_S154

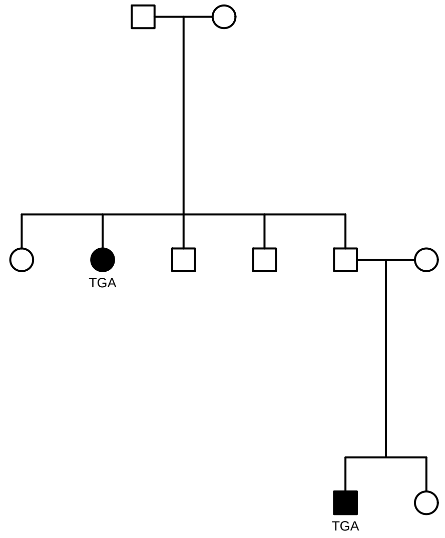

LYS_S196

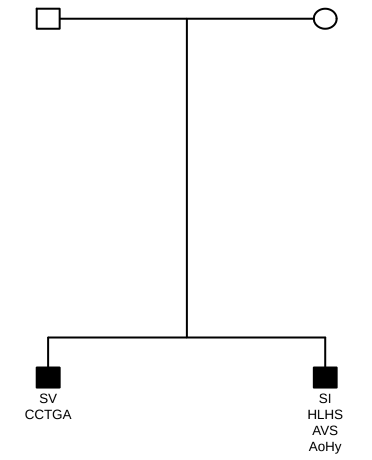

LYS_S197

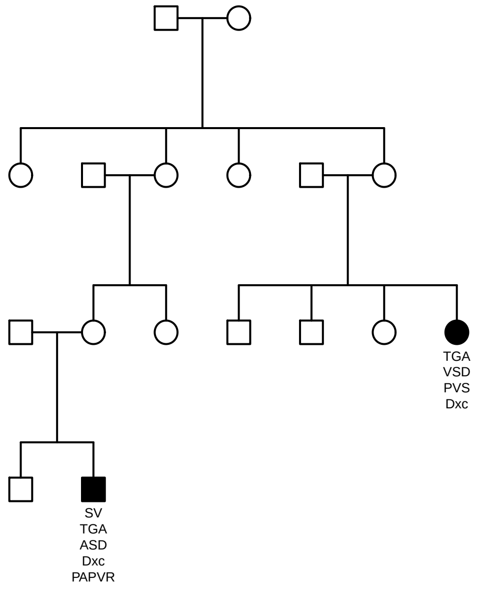

LYS_S198

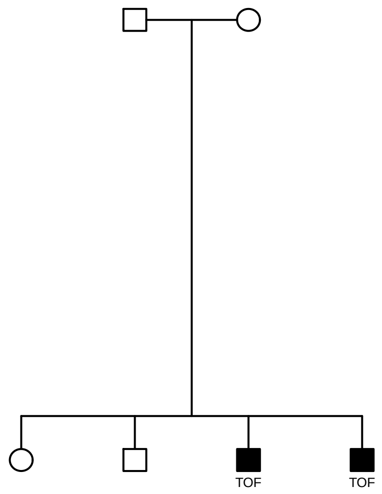

LYS_S199

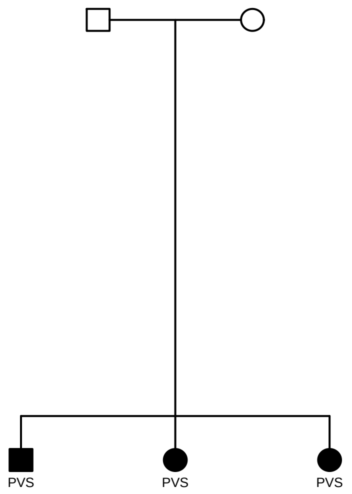

LYS_S209

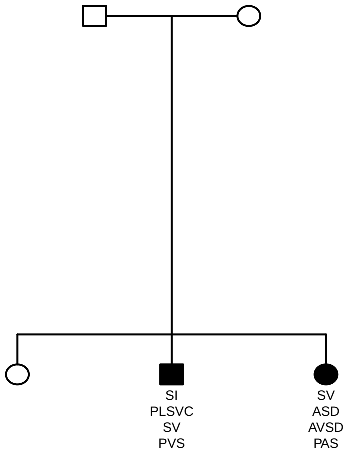

LYS_S210

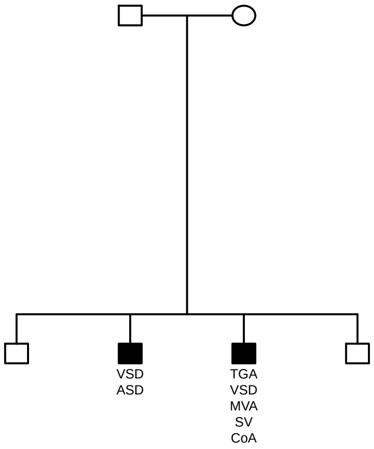

LYS_S212

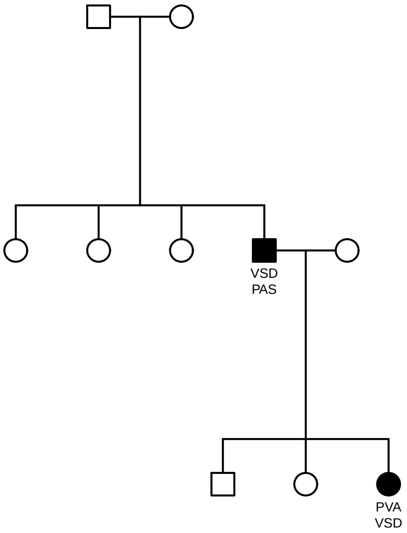

LYS_S213

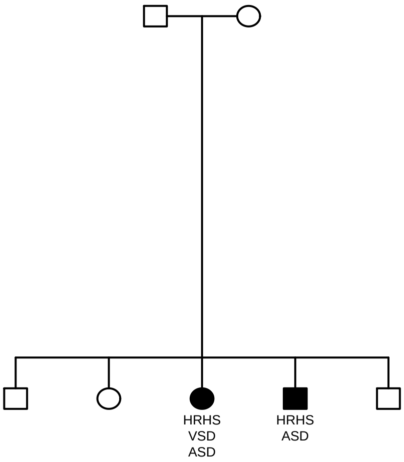

LYS_S219

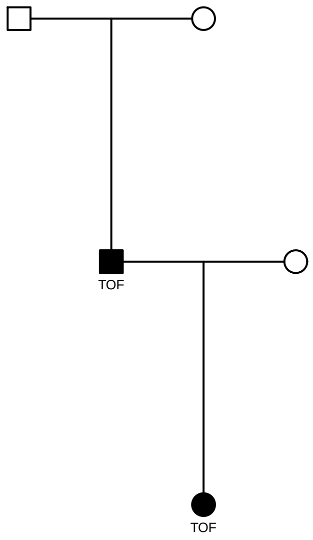

LYS_S241

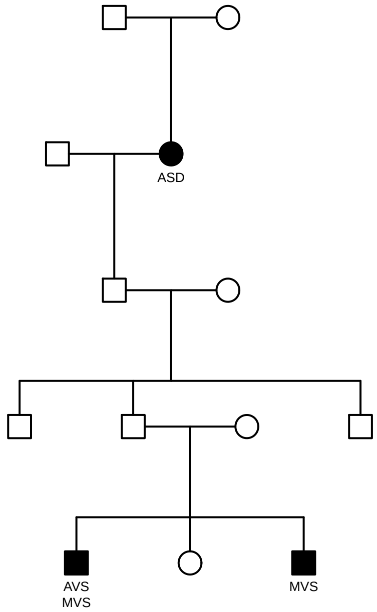

LYS_M001

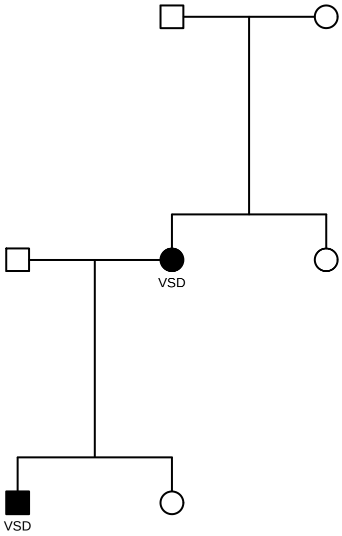

LYS_M002

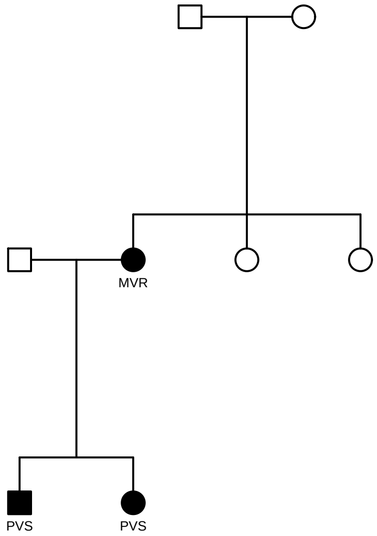

LYS_M004

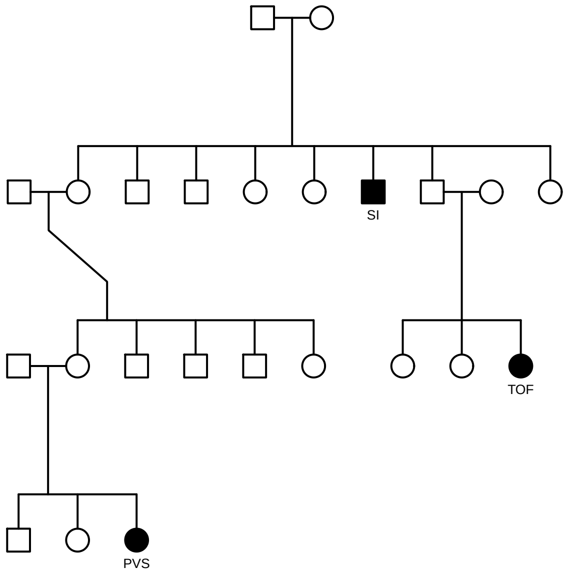

LYS_M005

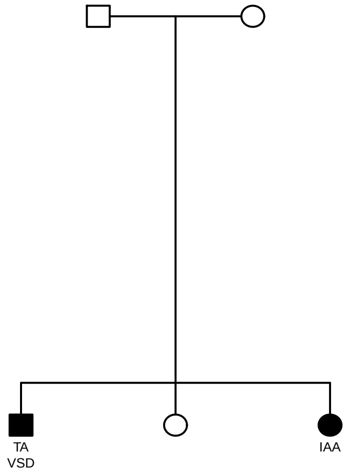

LYS_M006

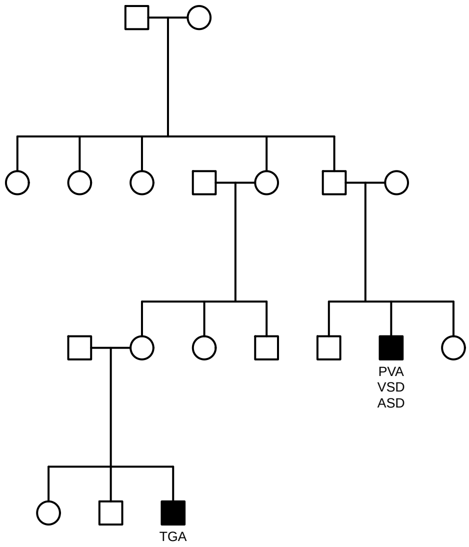

LYS_M007

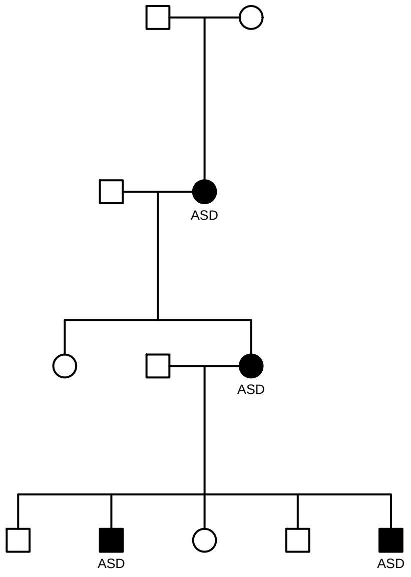

LYS_M008

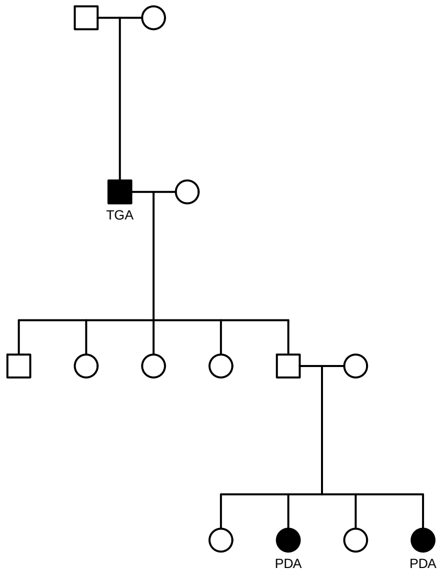

LYS_M009

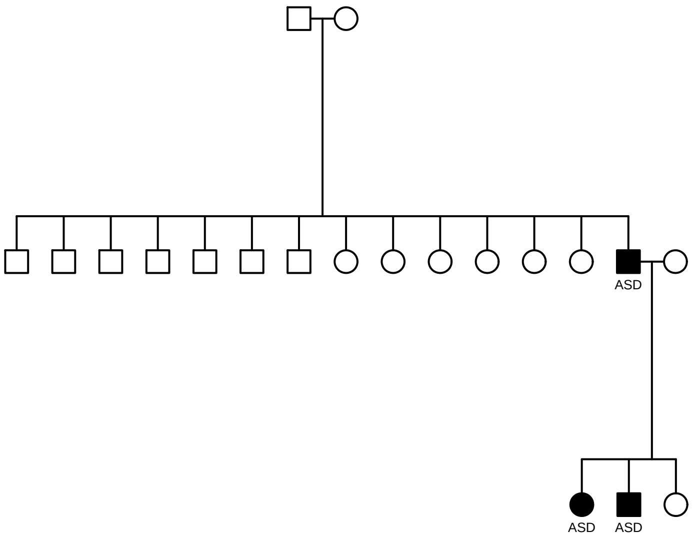

LYS_M011

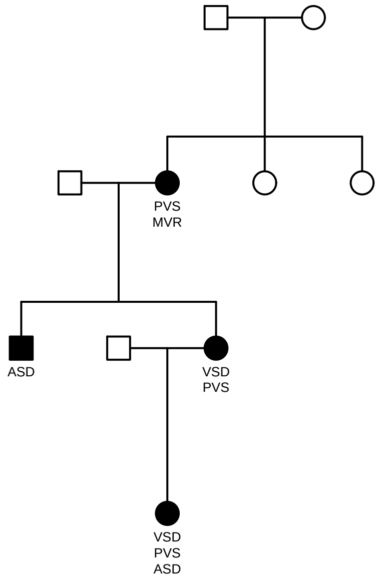

LYS_M012

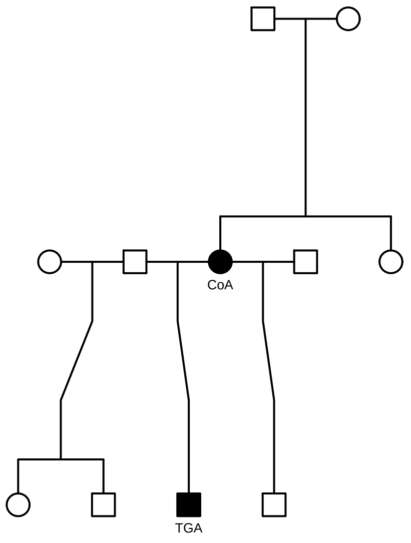

LYS_M013

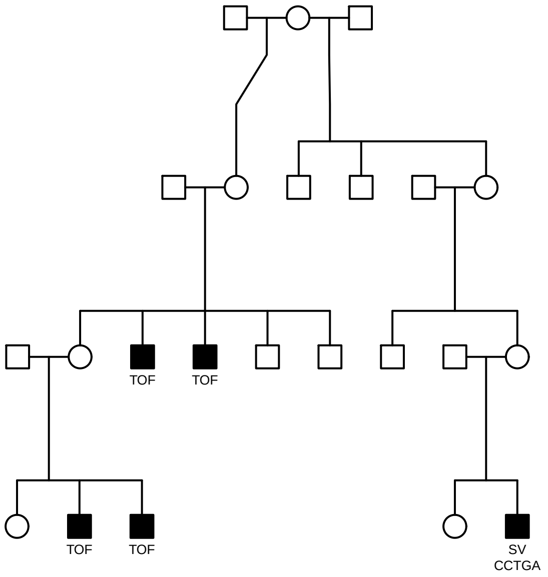

LYS_M014

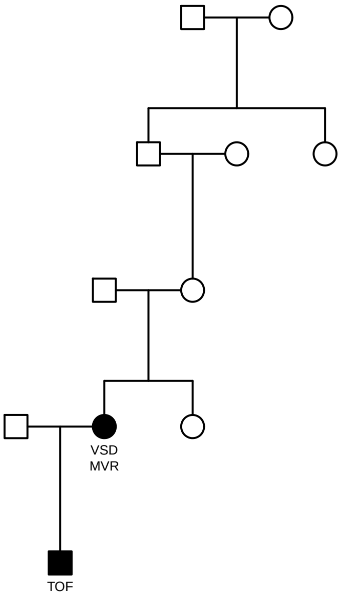

LYS_M015

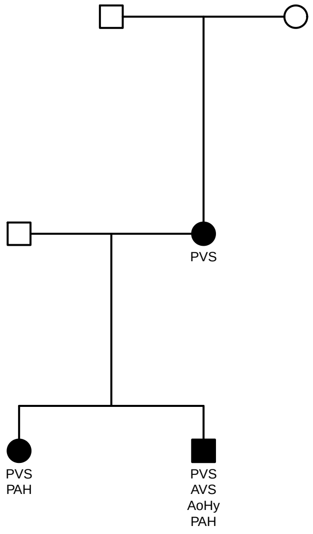

LYS_M016

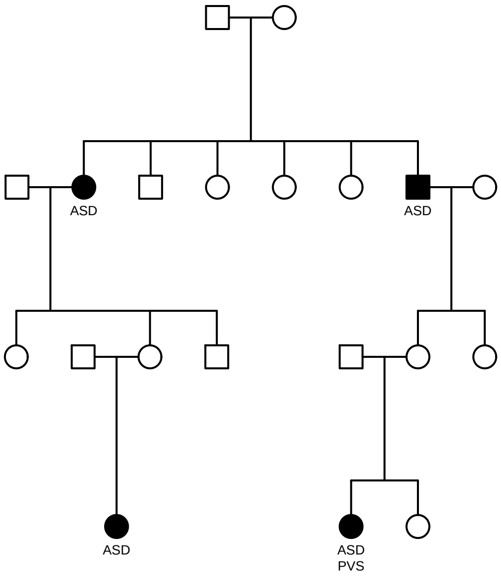

LYS_M017

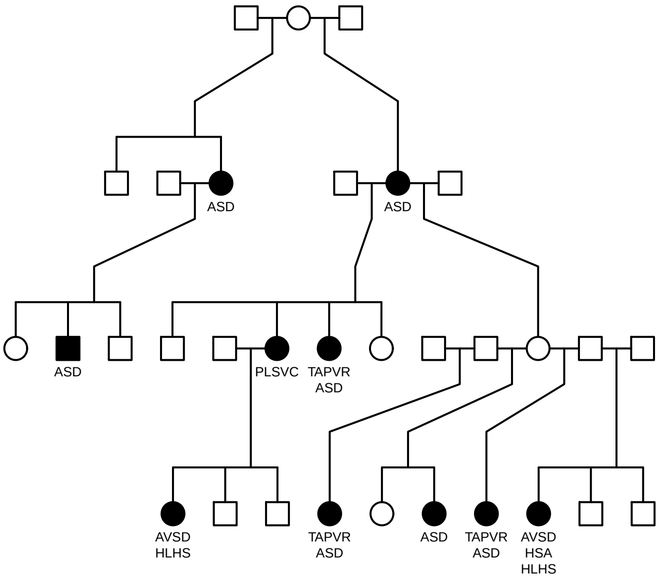

LYS_M018

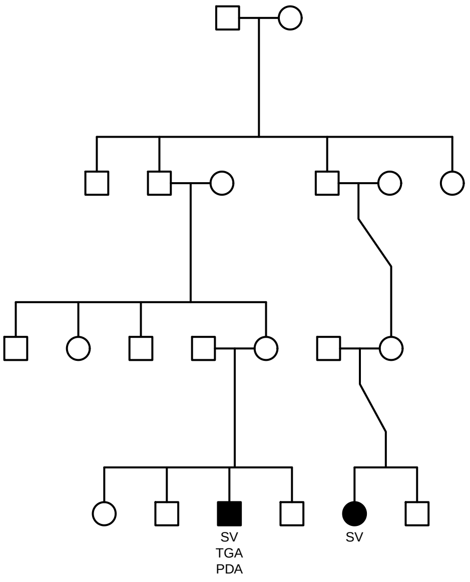

LYS_M019

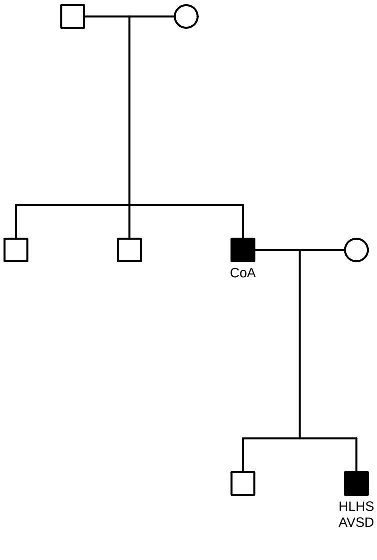

LYS_M021

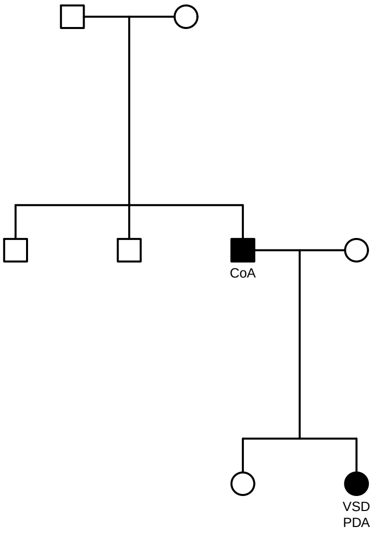

LYS_M022

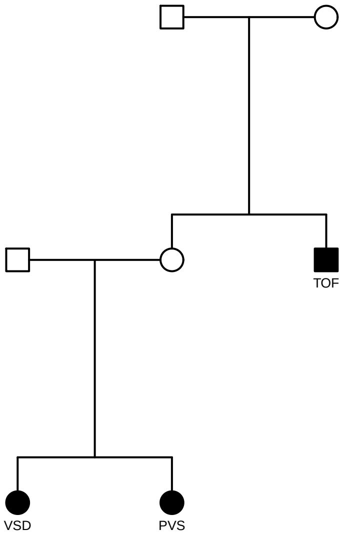

LYS_M024

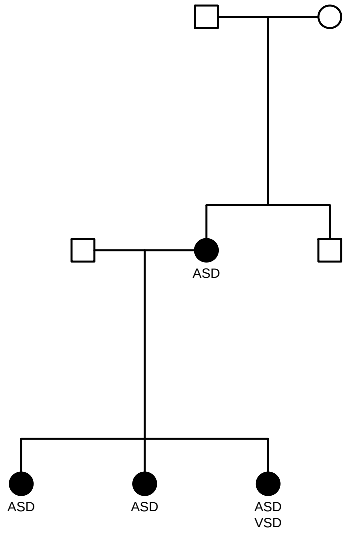

LYS_M025

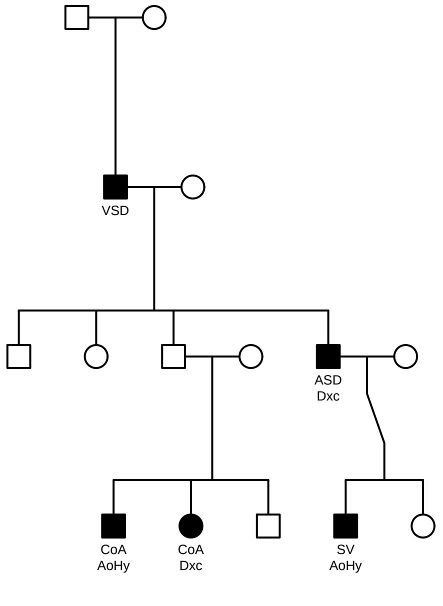

LYS_M027

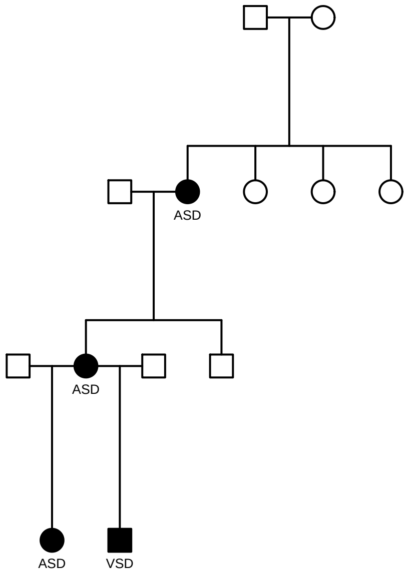

LYS_M030

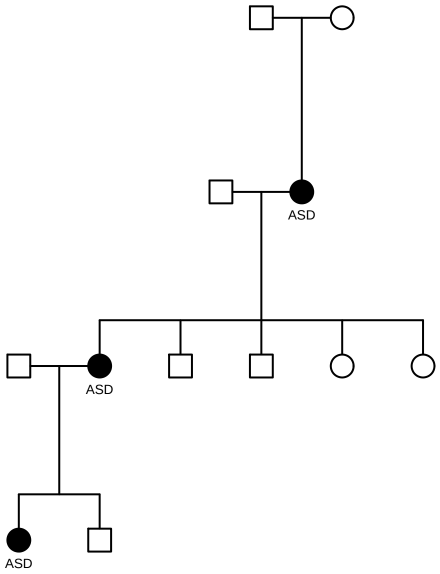

LYS_M031

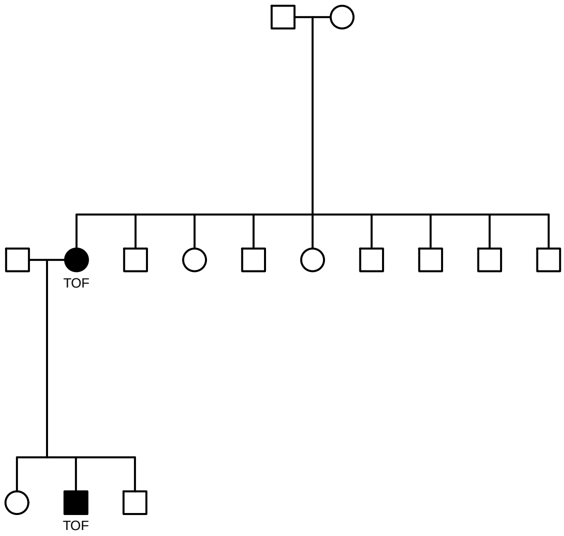

LYS_M034

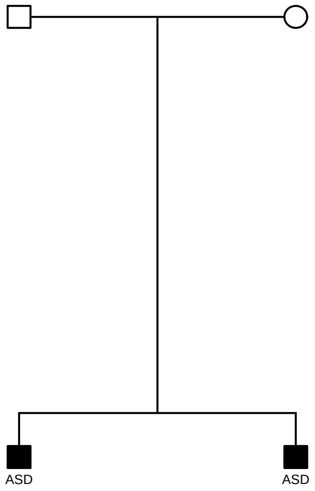

LYS_M035

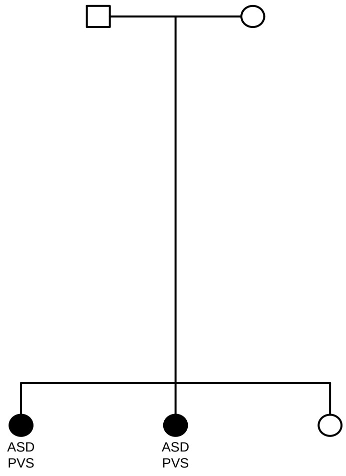

LYS_M037

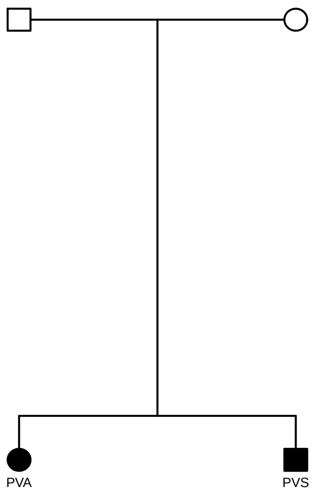

LYS_M038

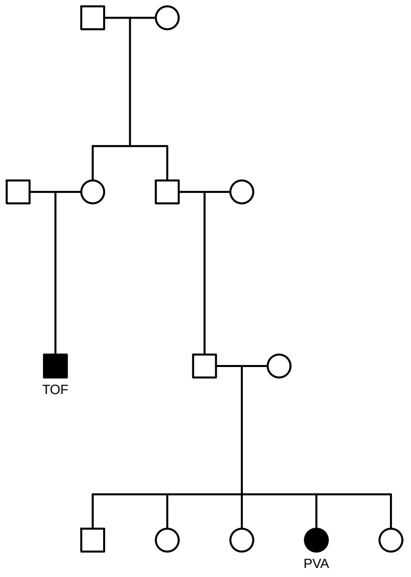

LYS_M041

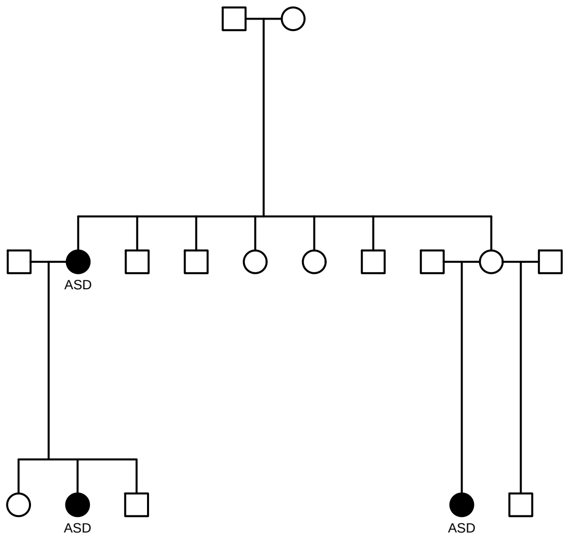

LYS_M042

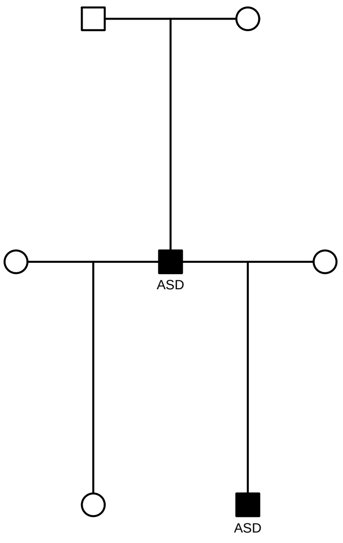

LYS_M043

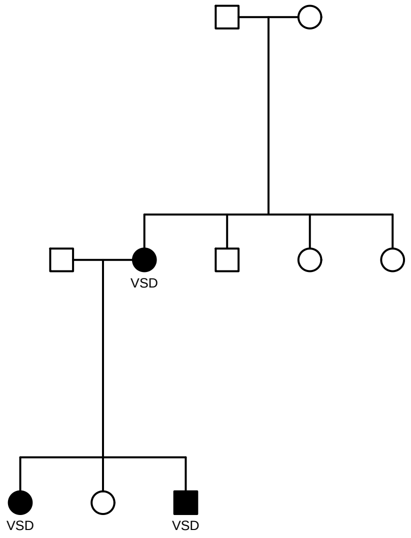

LYS_M046

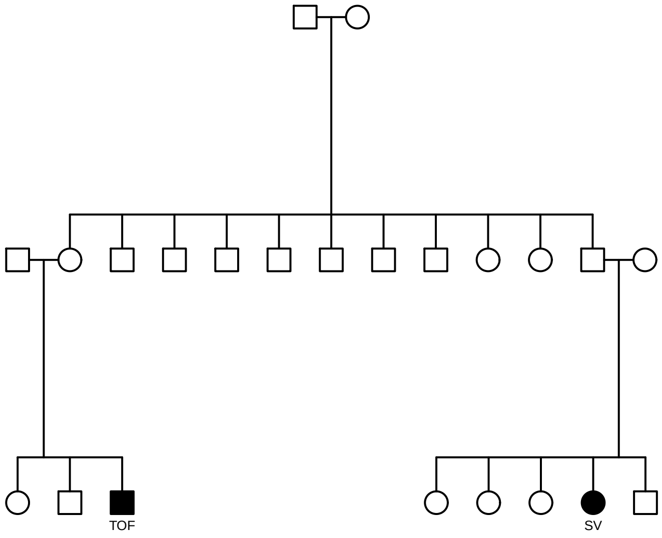

LYS_M047

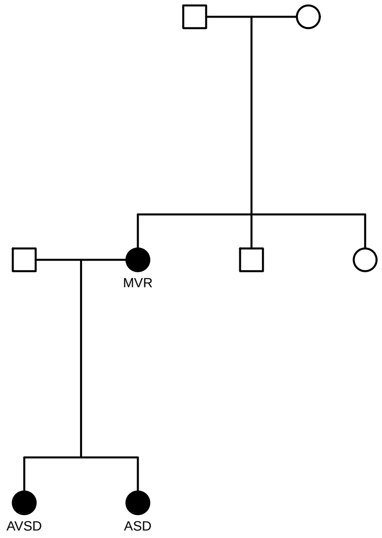

LYS_M050

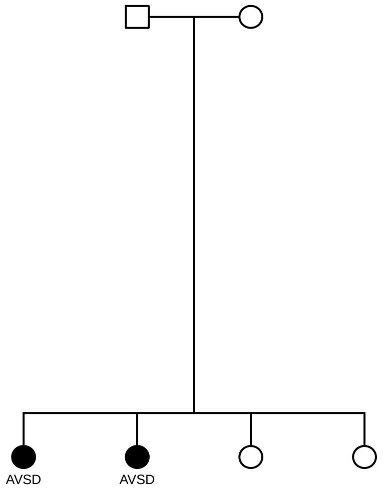

LYS_M051

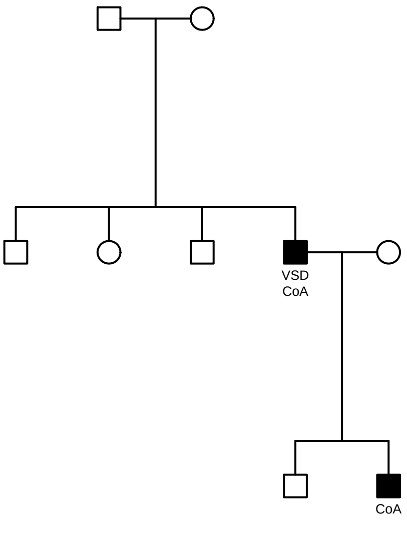

LYS_M052

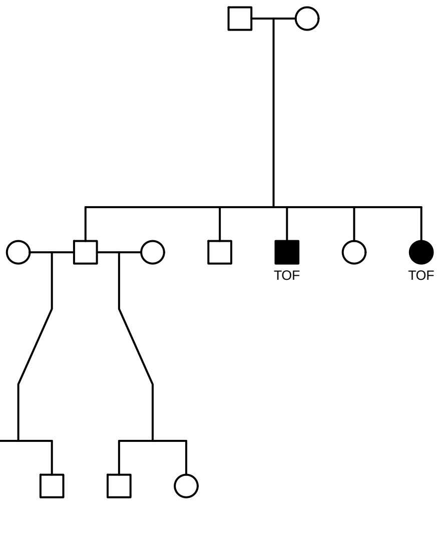

LYS_M053

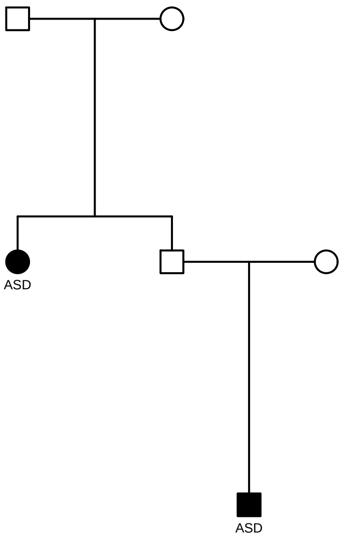

LYS_M054

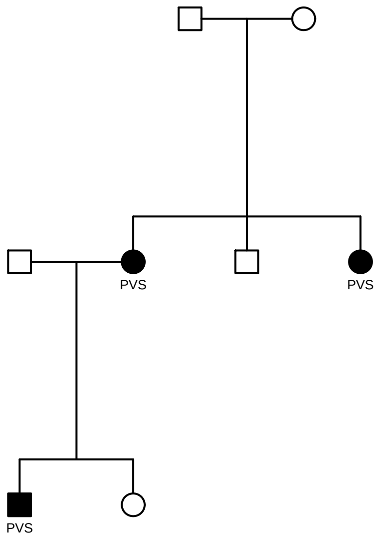

LYS_M056

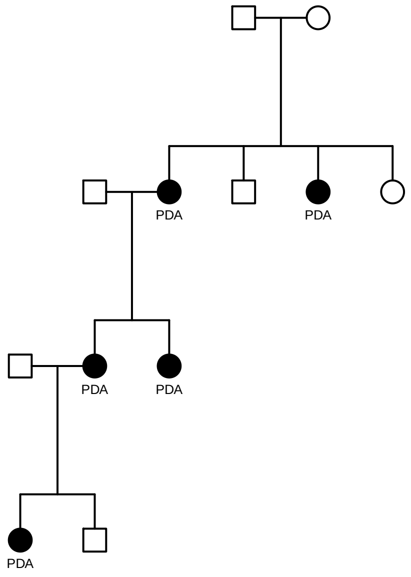

LYS_M057

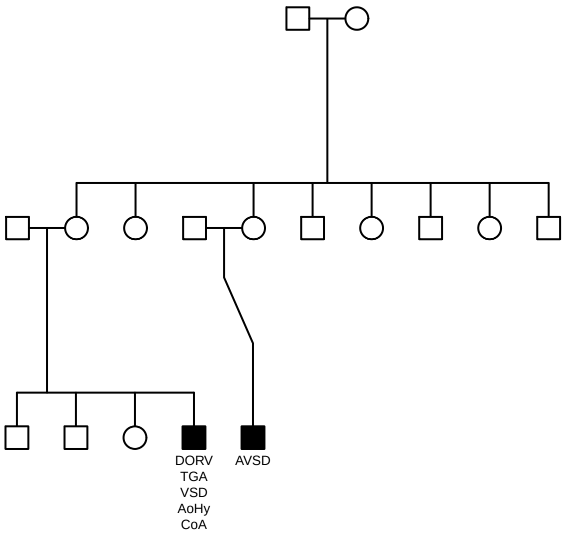

LYS_M058

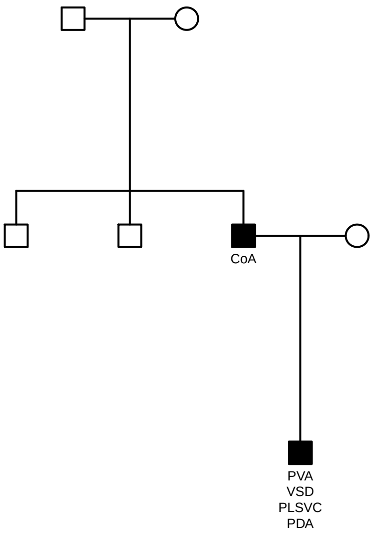

LYS_M061

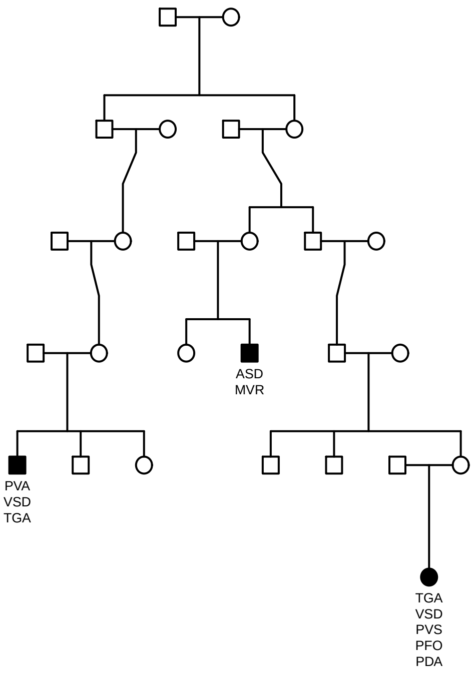

LYS_M062

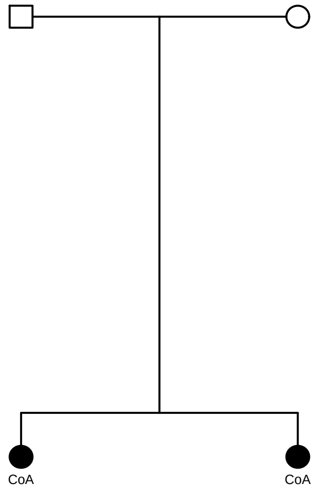

LYS_M063

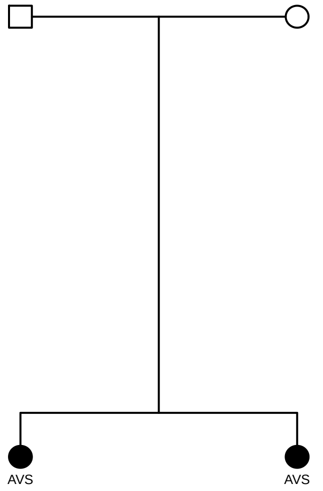

LYS_M065

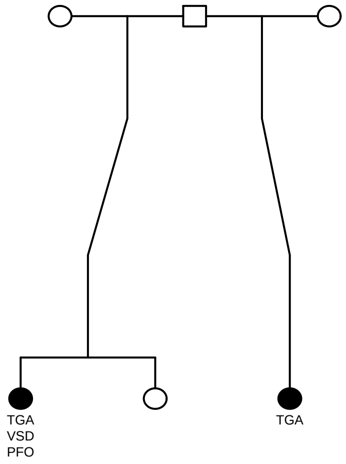

LYS_M066

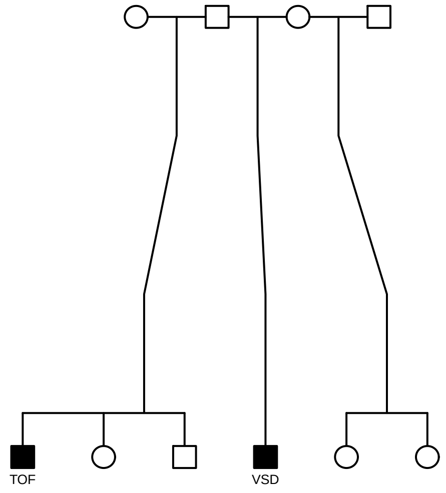

LYS_M068

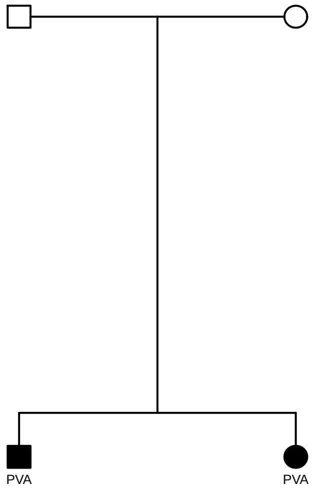

LYS_M069

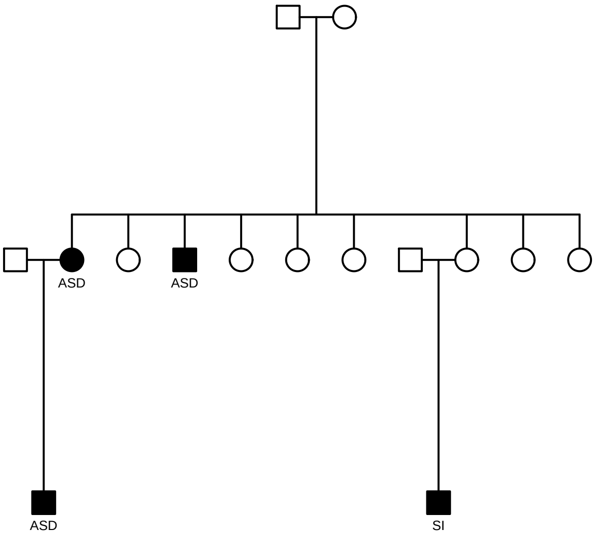

LYS_M070

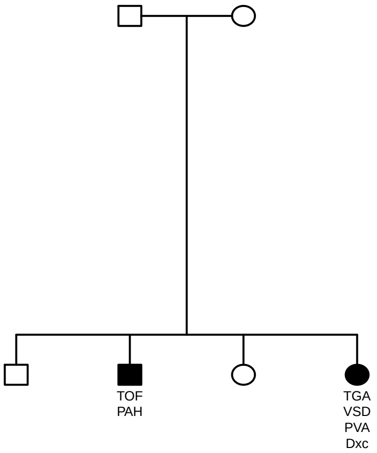

LYS_M073

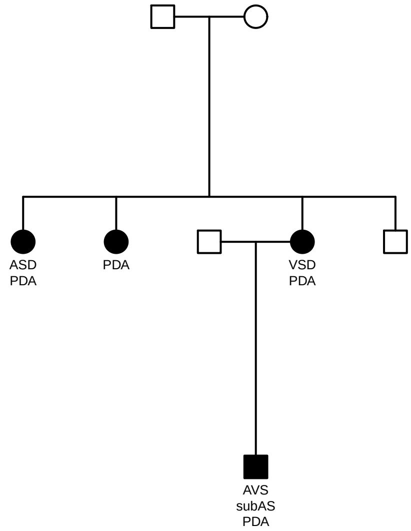

LYS_M074

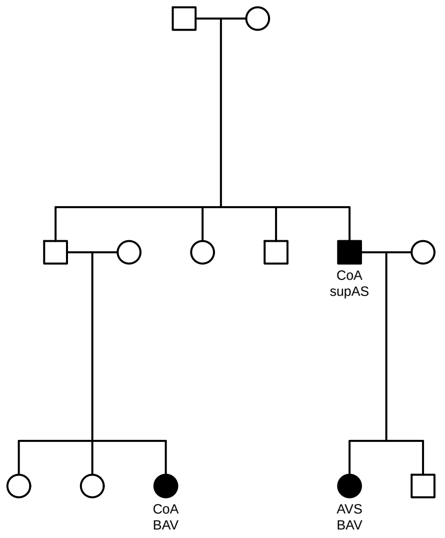

LYS_M075

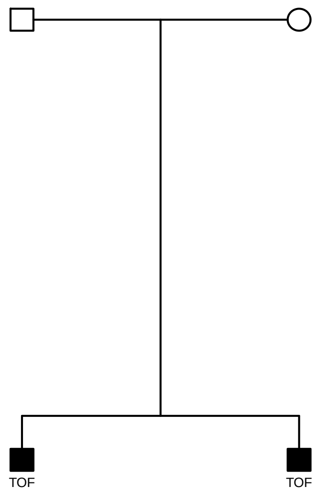

LYS_M078

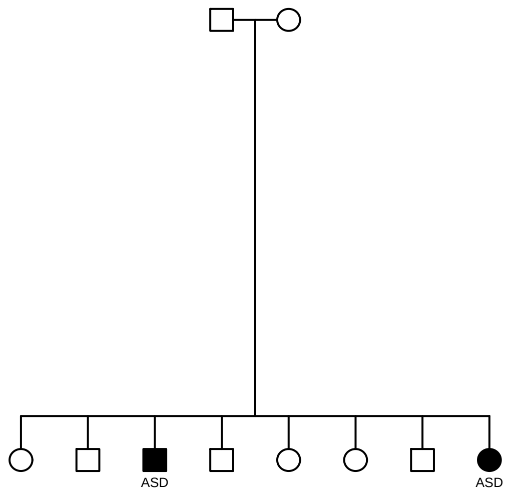

LYS_M079

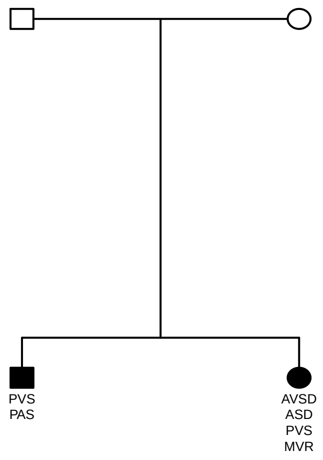

LYS_M080

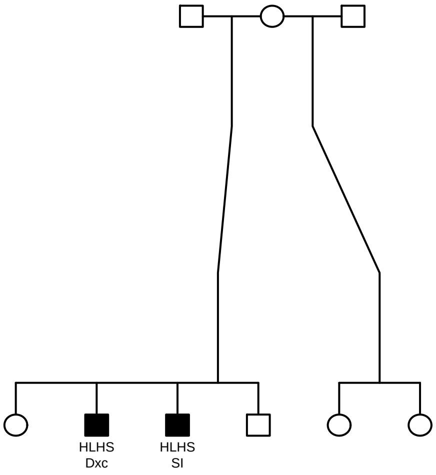

LYS_M081

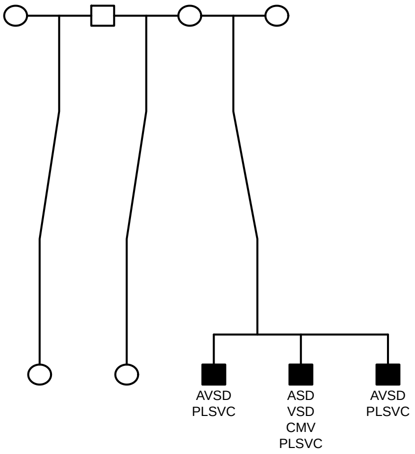

LYS_M082

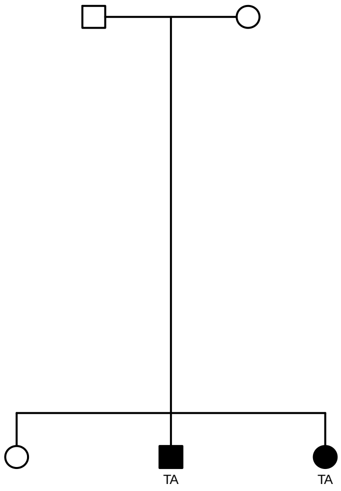

LYS_M083

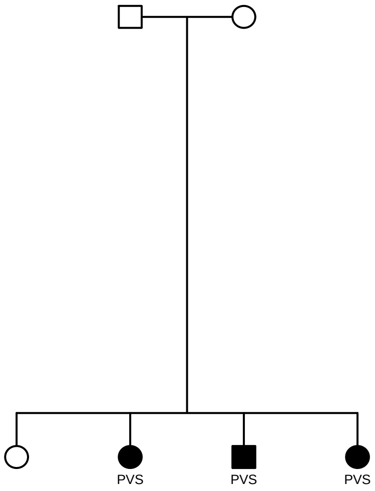

LYS_M084

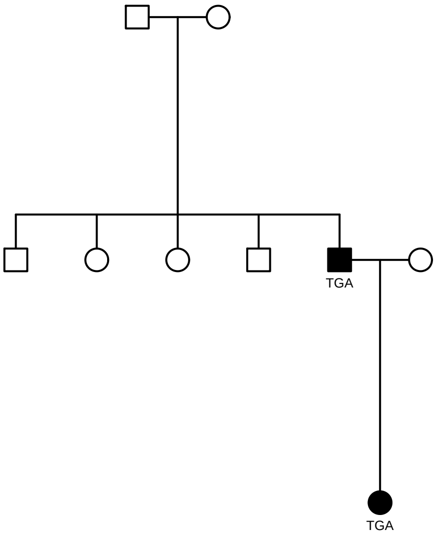

LYS_M085

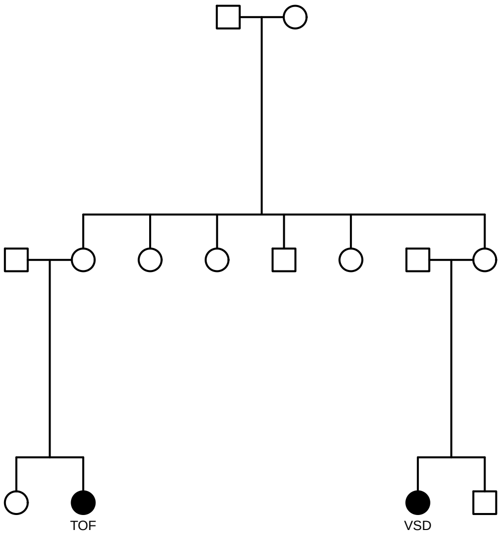

LYS_M086

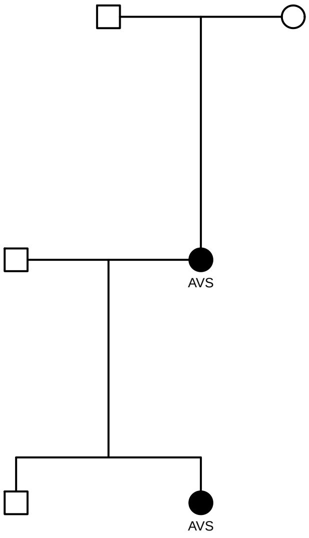

LYS_M087

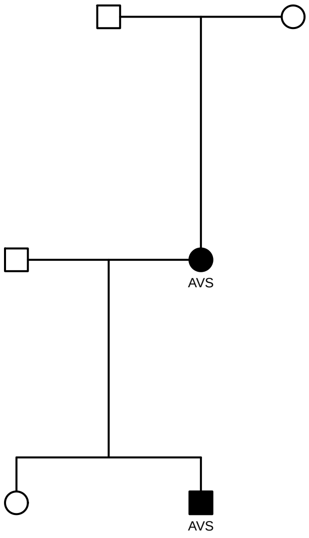

LYS_M088

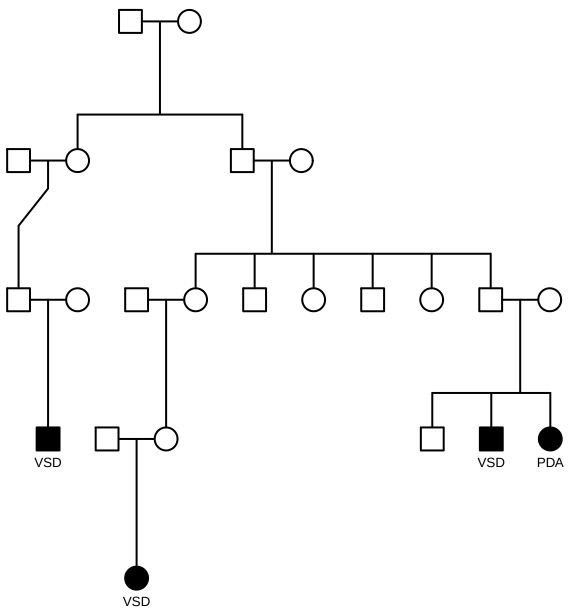

LYS_M089

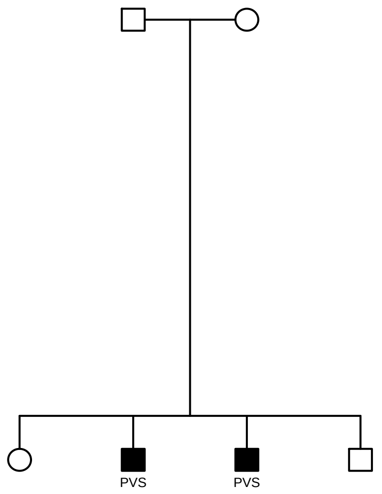

LYS_M090

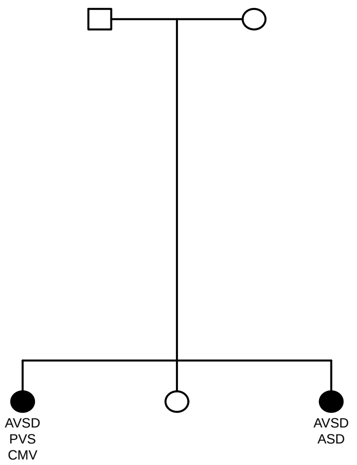

LYS_M091

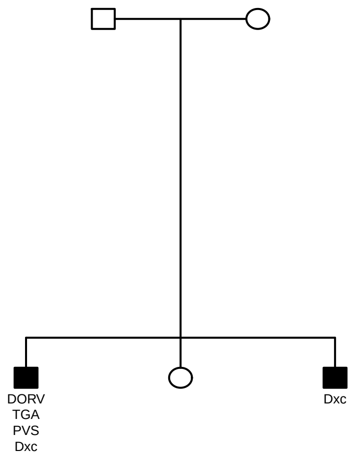

LYS_M092

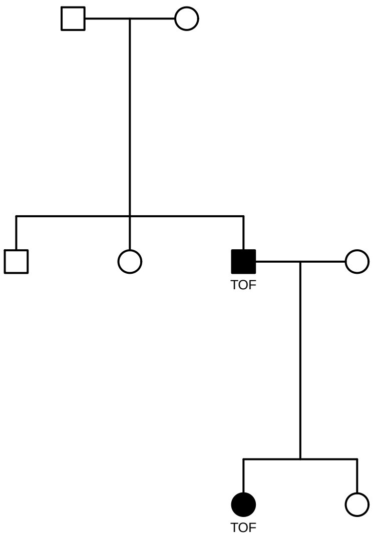

LYS_M093

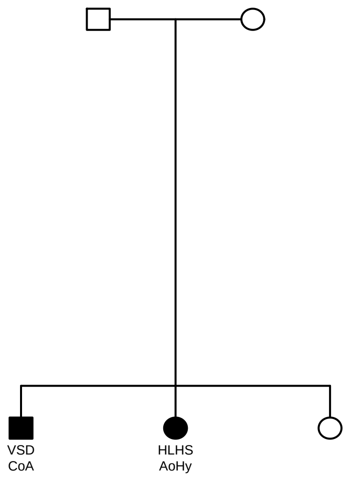

LYS_M095

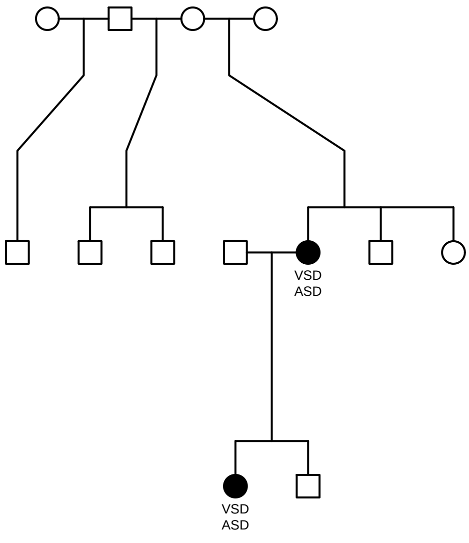

LYS_M096

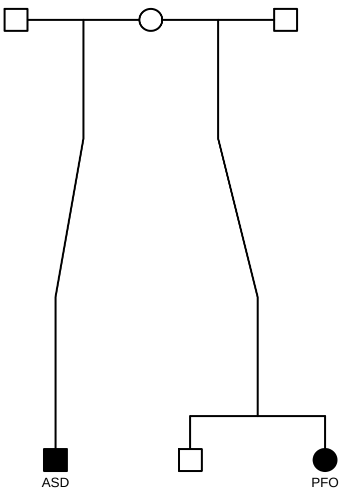

LYS_M097

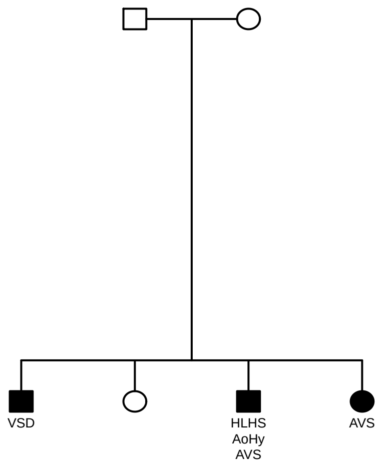

LYS_M098

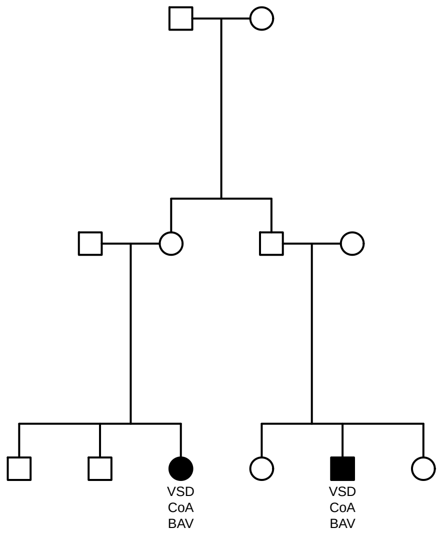

LYS_M099

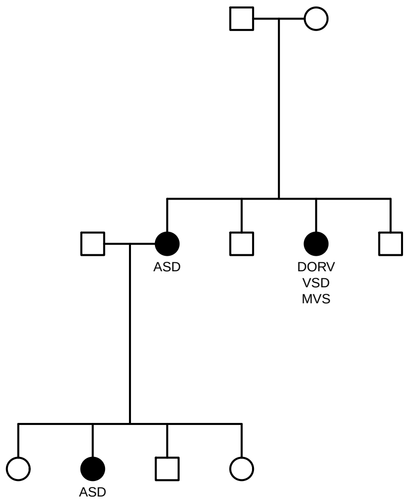

LYS_M100

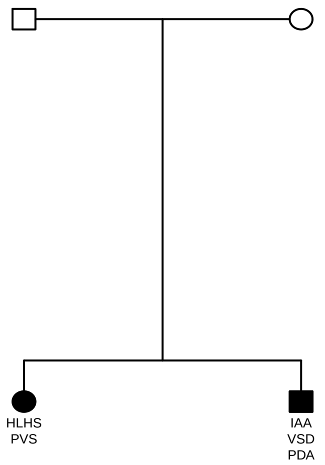

LYS_M101

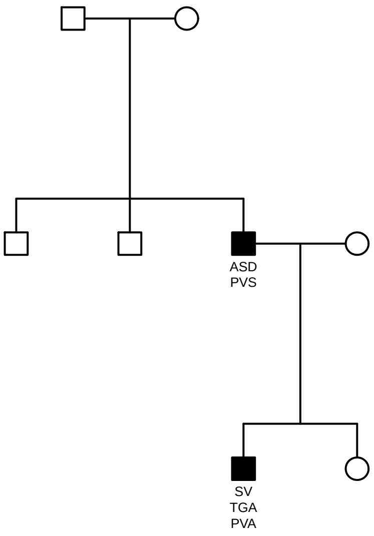

LYS_M102

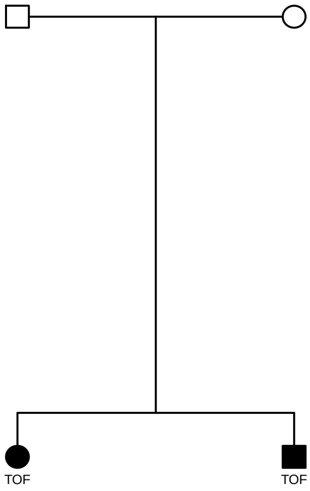

LYS_M104

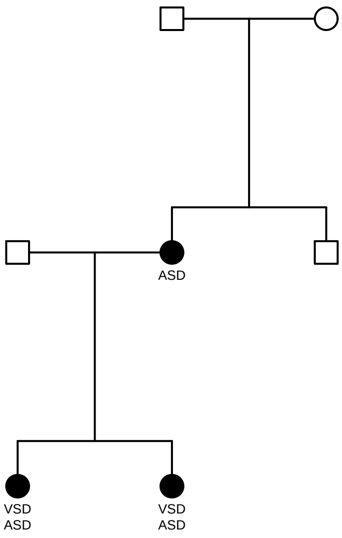

LYS_M105

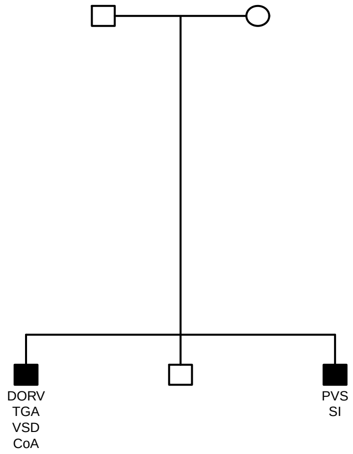

LYS_AKBA

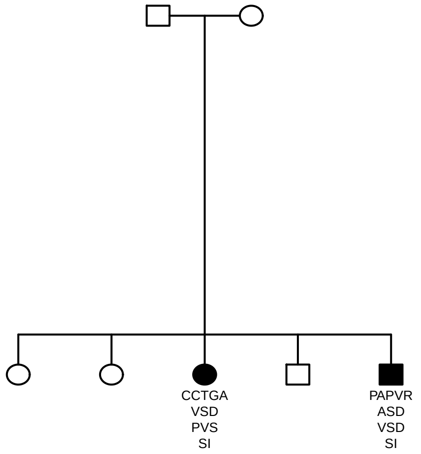

LYS_ALLA

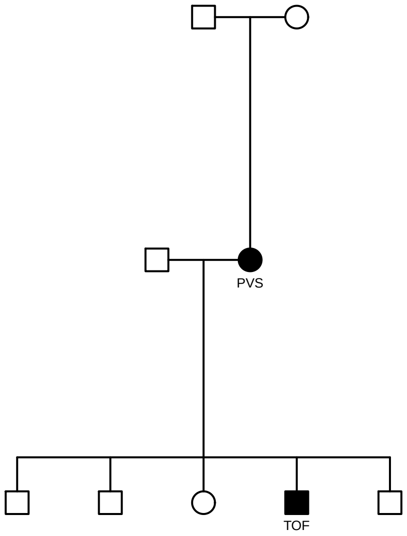

LYS_CARD

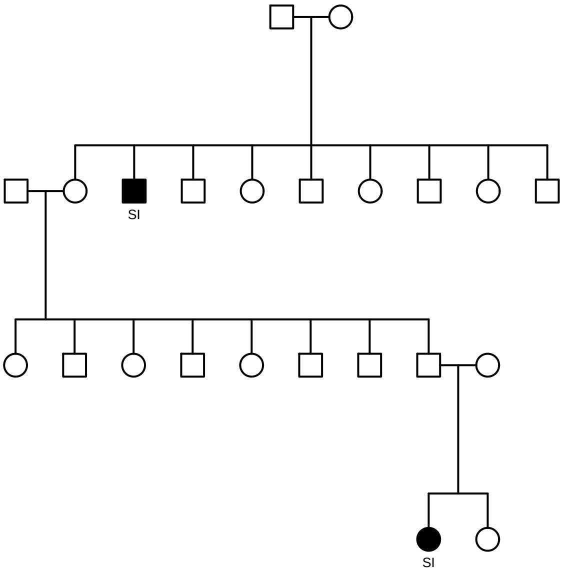

LYS_DEDD

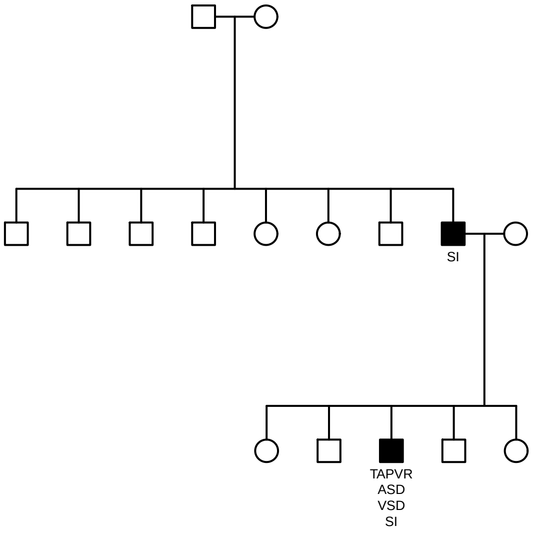

LYS_DELO

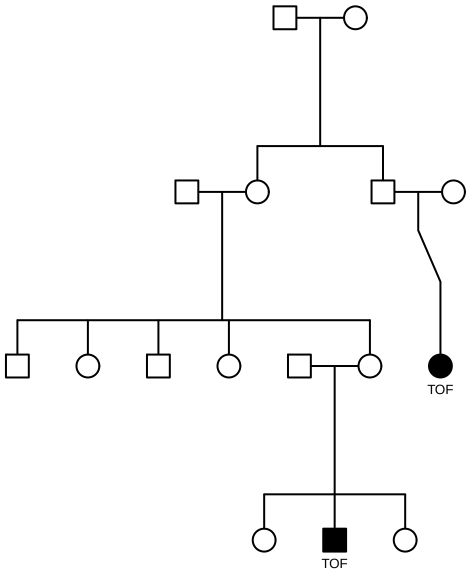

LYS_EBRI

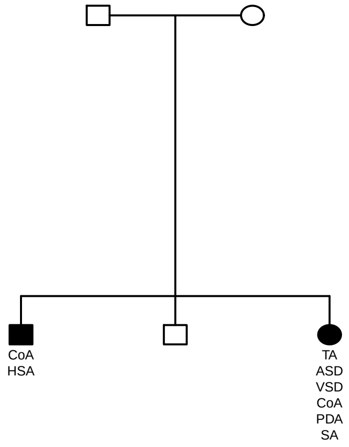

LYS_ELRA

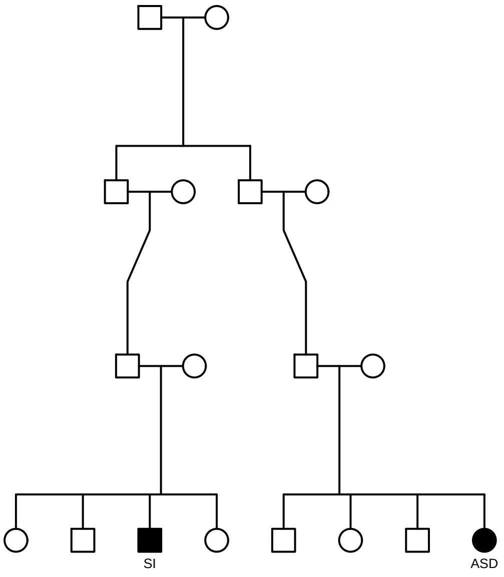

LYS_ELSA

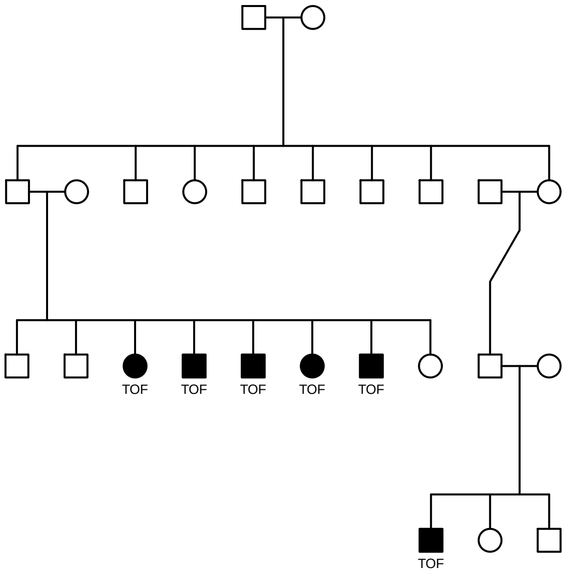

LYS_GIM1

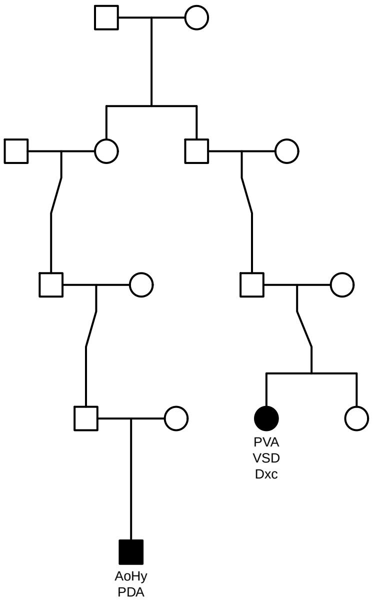

LYS_GINI

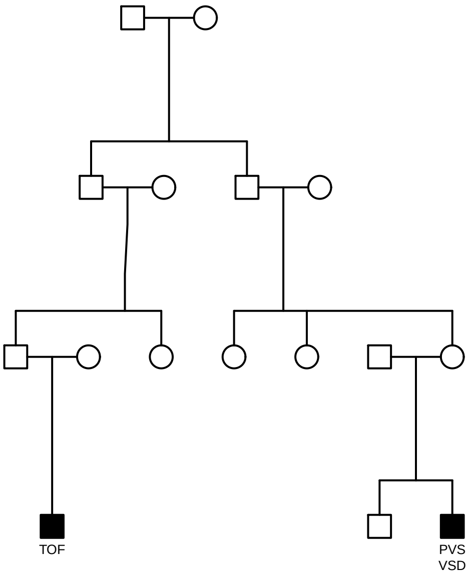

LYS_HERK

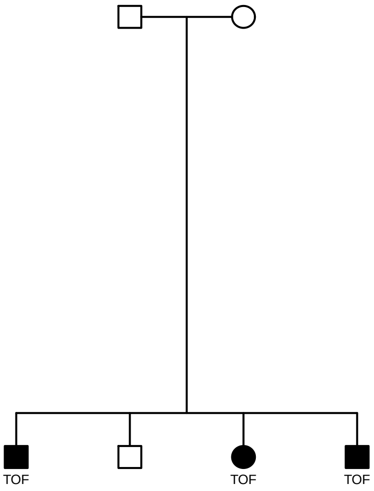

LYS_JARR

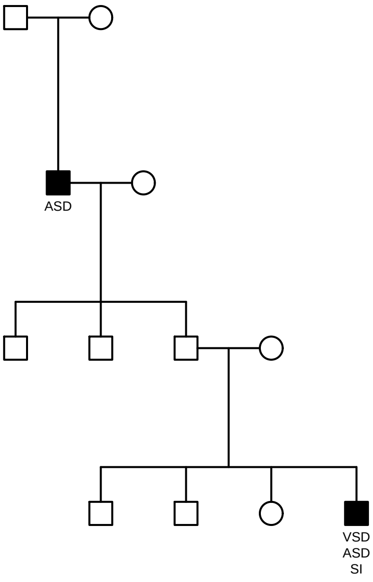

LYS_JOBS

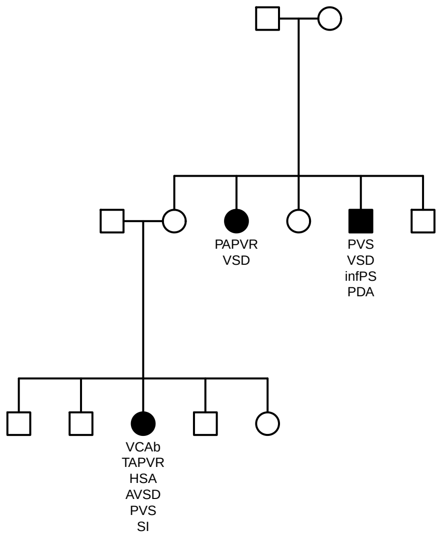

LYS_KAHA

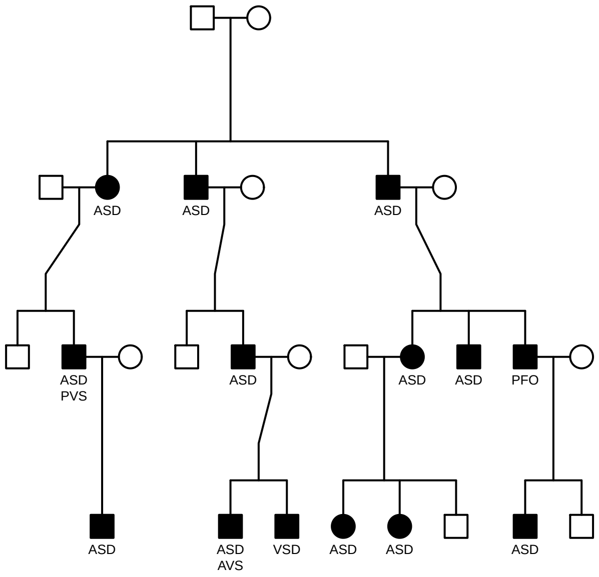

LYS_KAND

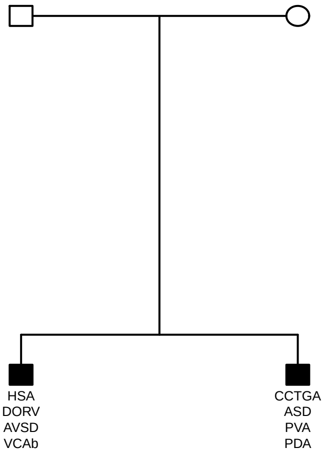

LYS_KHAS

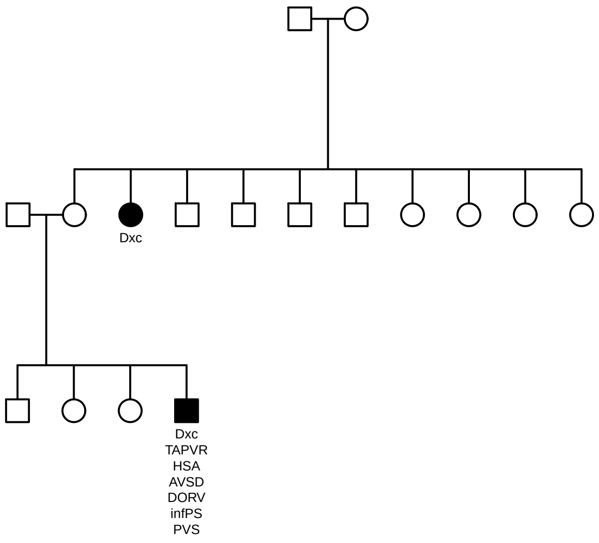

LYS_KOCO

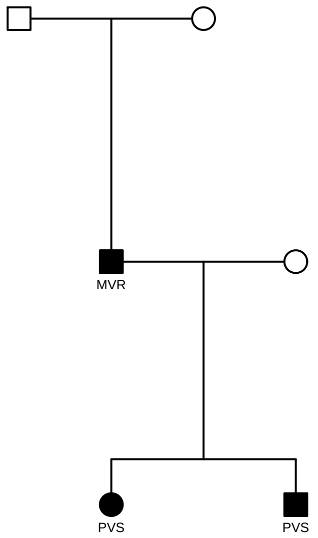

LYS_KUP

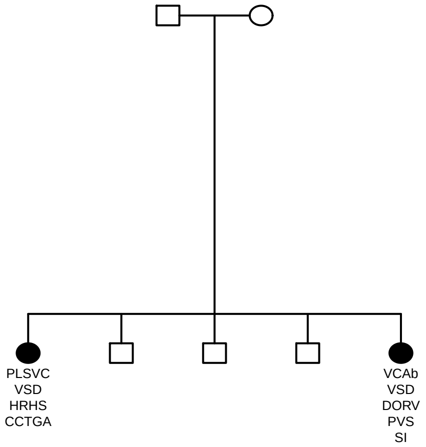

LYS_LEBR

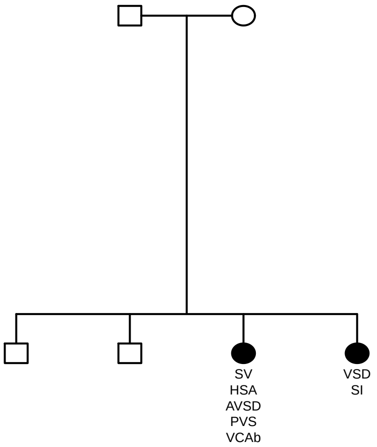

LYS_LUBI

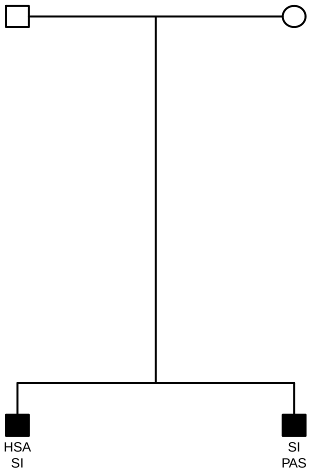

LYS_MACH

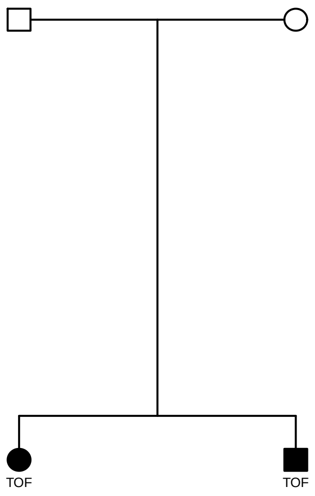

LYS_MAHE

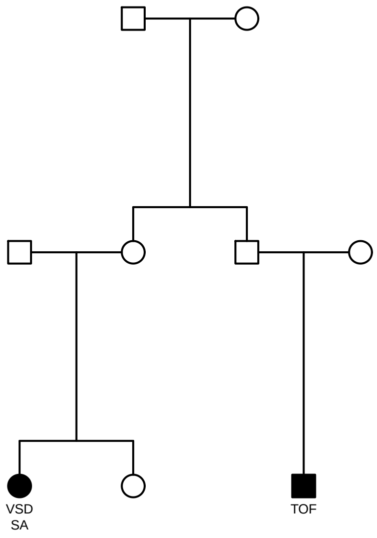

LYS_MASS

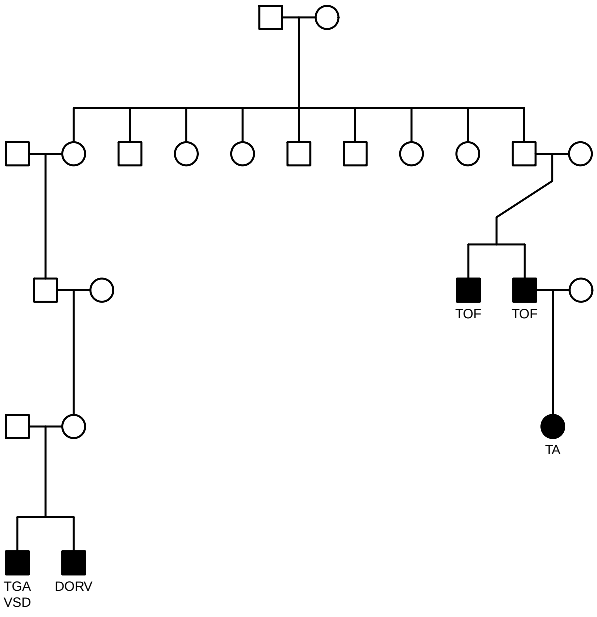

LYS_MINT

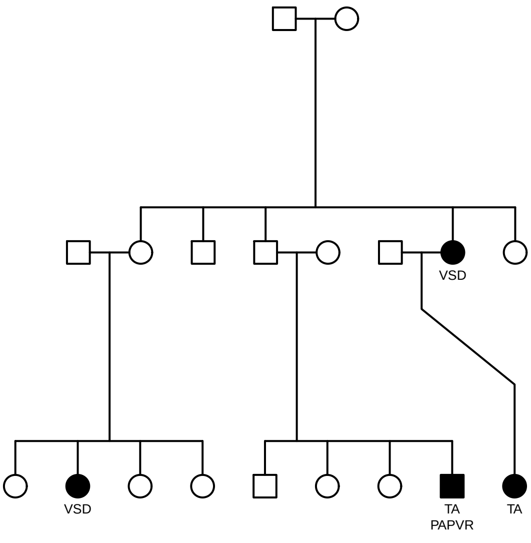

LYS_MULL

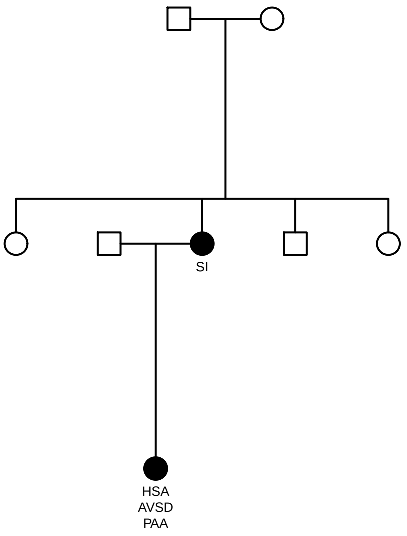

LYS_NAYR

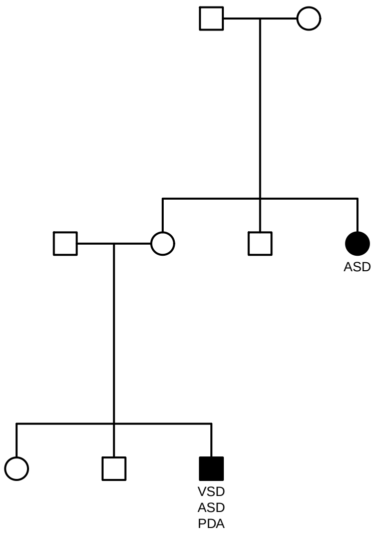

LYS_OTT

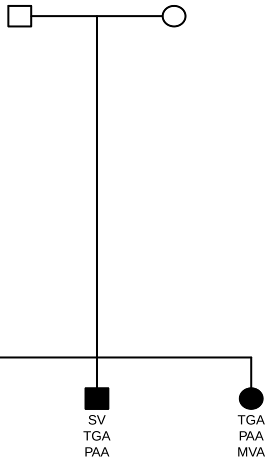

LYS_PAPE

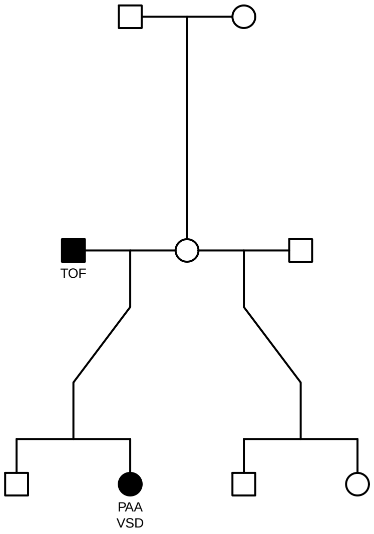

LYS_RAGI

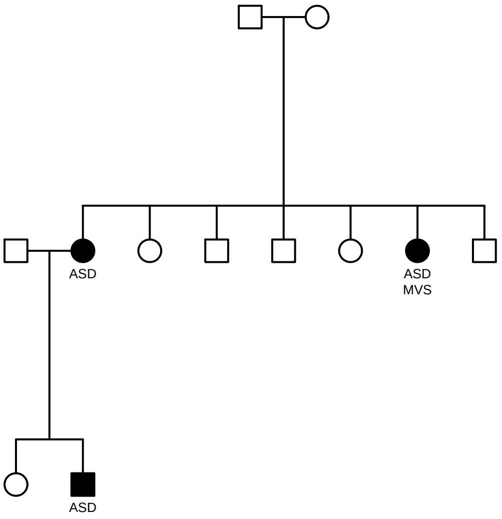

LYS_RAUS

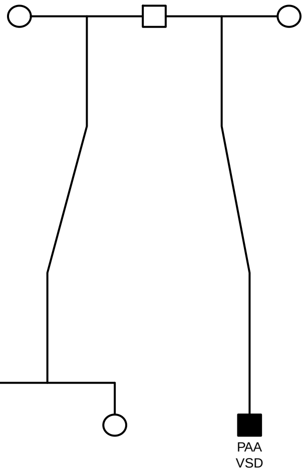

LYS_REIT

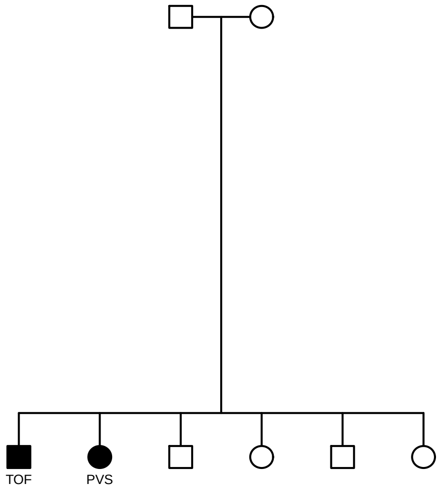

LYS_SALE

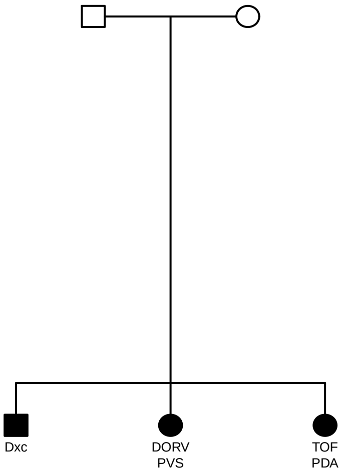

LYS_SCHN

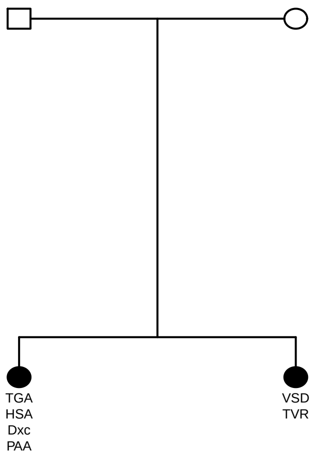

LYS_SEEG

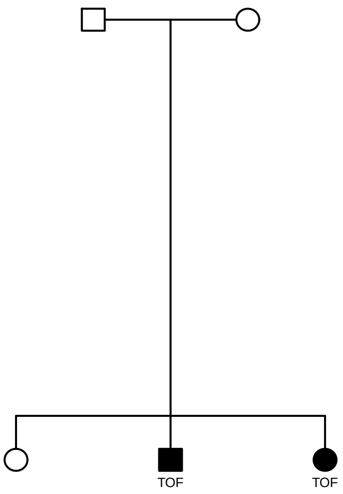

LYS_SENF

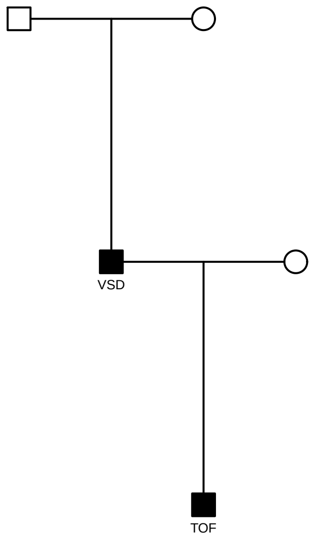

LYS_STEI

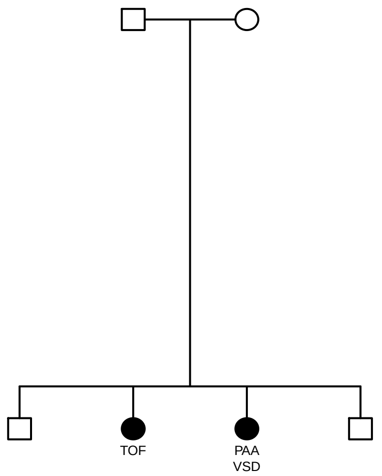

LYS_TACK

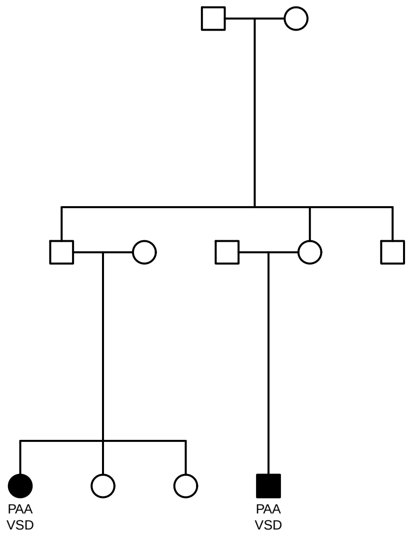

LYS_TARI

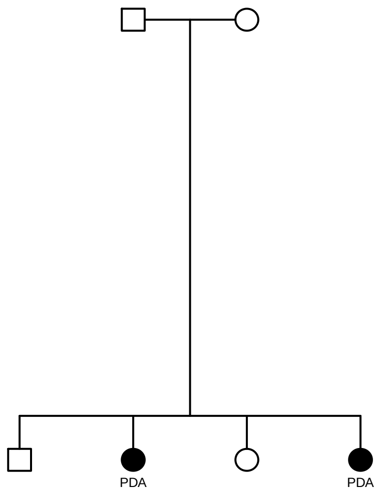

LYS_UIBO

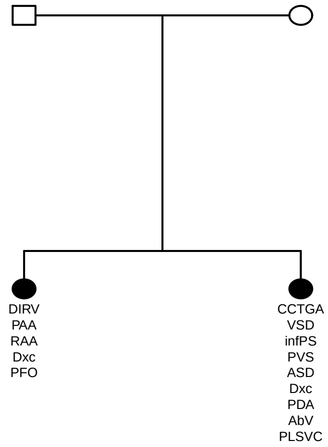

LYS_VERD

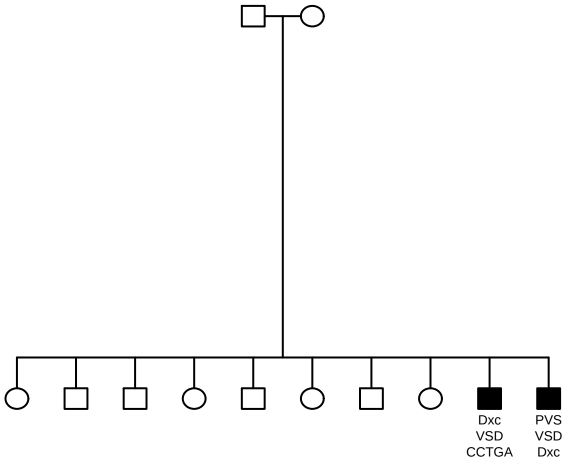

LYS_YUCE

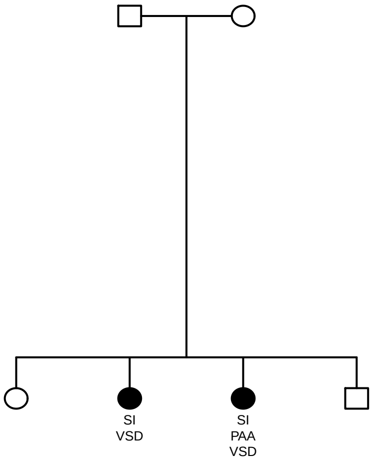

LYS_ZS&S

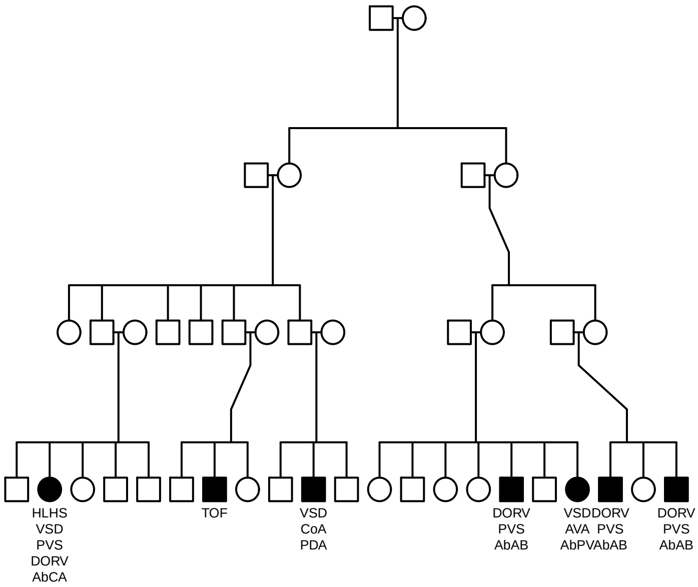

LYS_M106

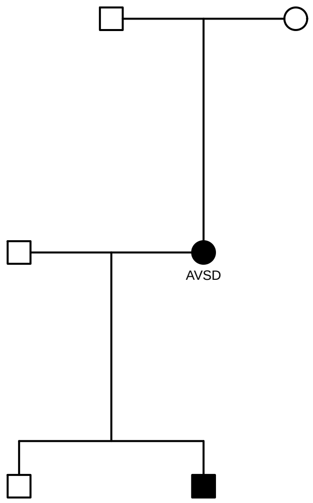

LYS_M108

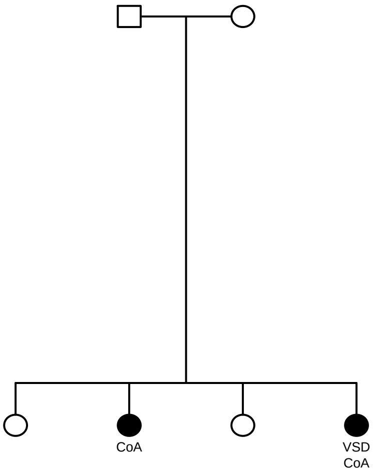

LYS_M109

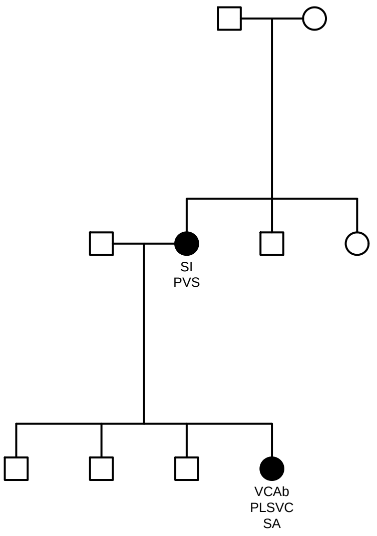

LYS_M111

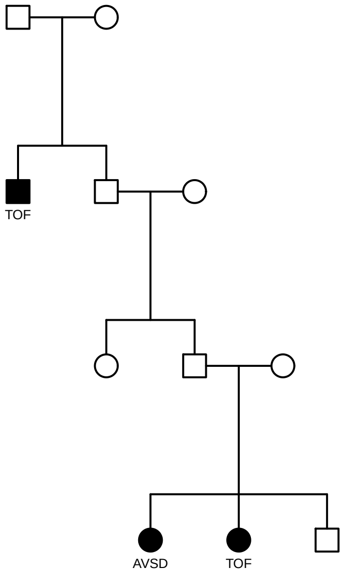

LYS_M112

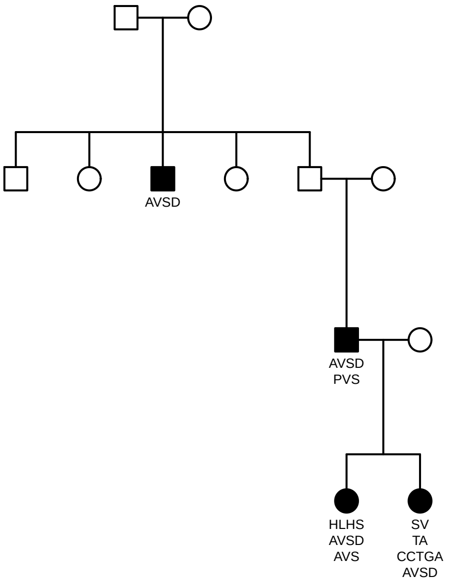

LYS_M113

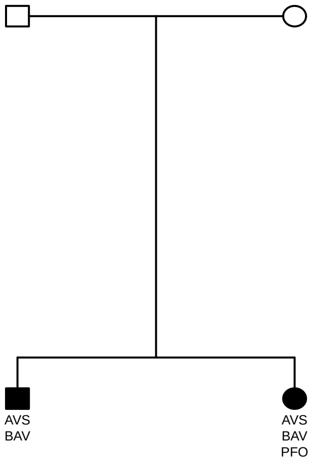

LYS_M115

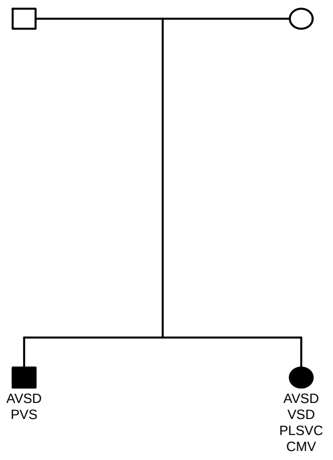

LYS_M116

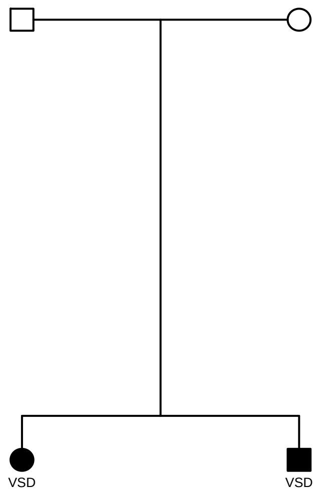

LYS_M117

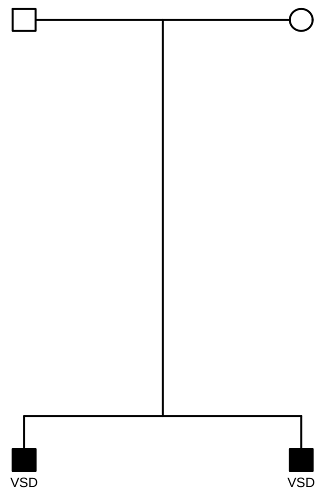

LYS_M118

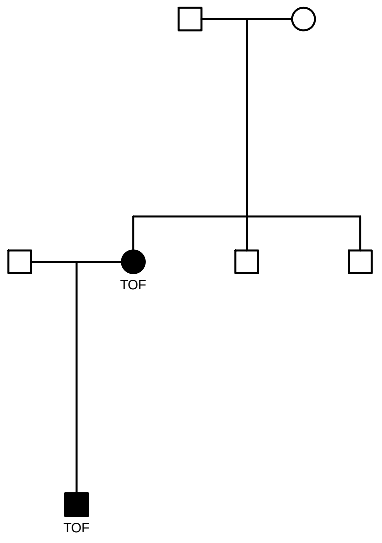

LYS_M119

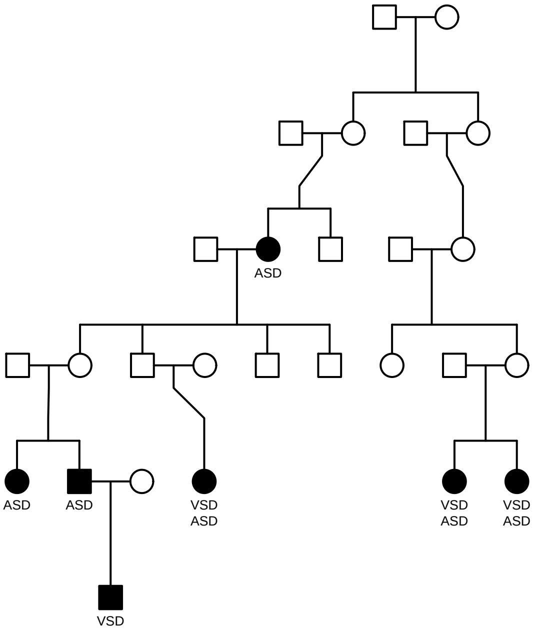

LYS_M120

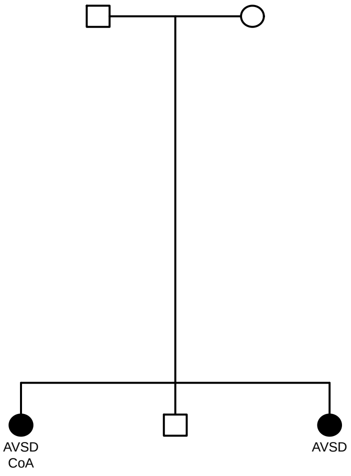

LYS_M121

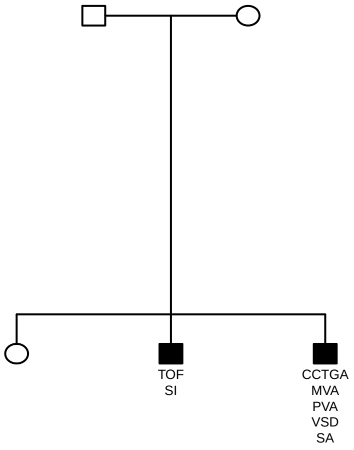

LYS_M122

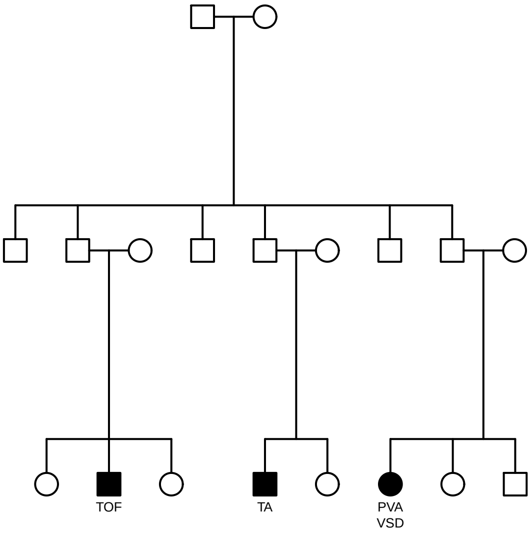

LYS_M125

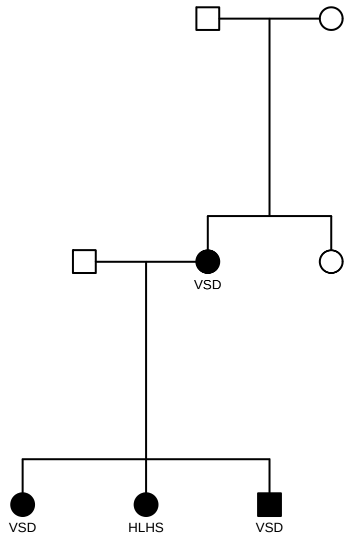

LYS_M126

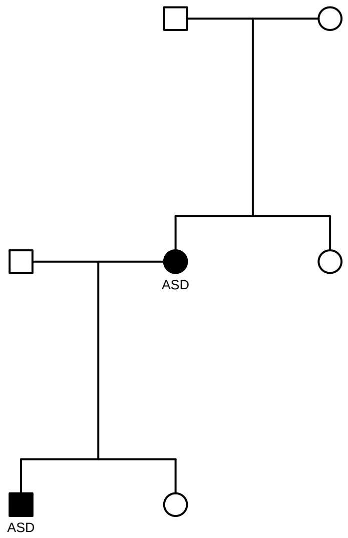

LYS_M127

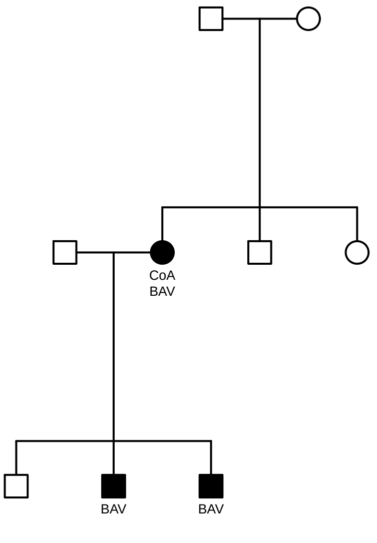

LYS_M128

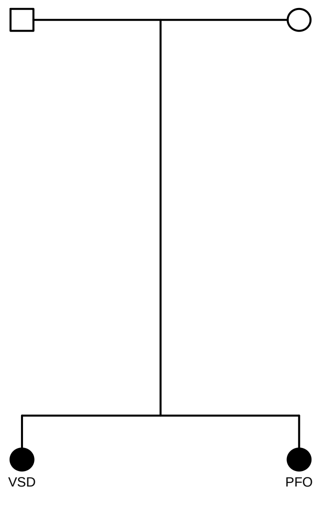

LYS_M130

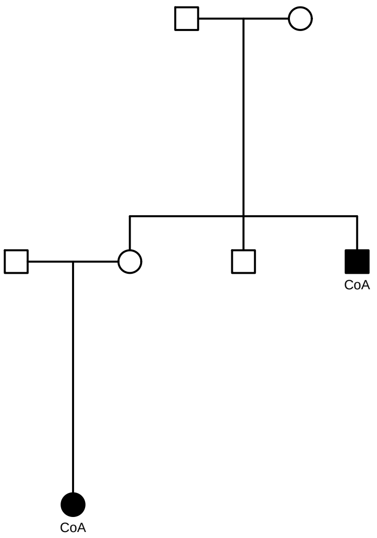

LYS_M132

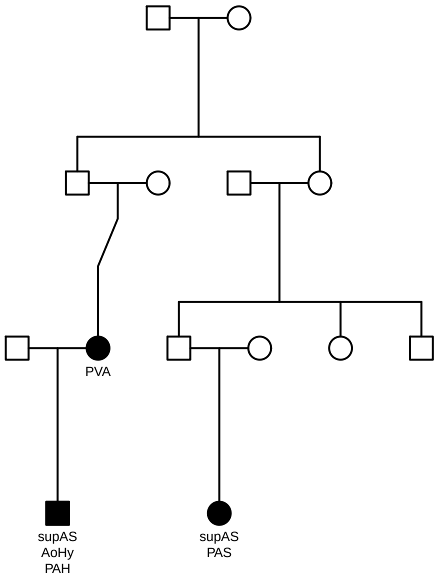

LYS_M134

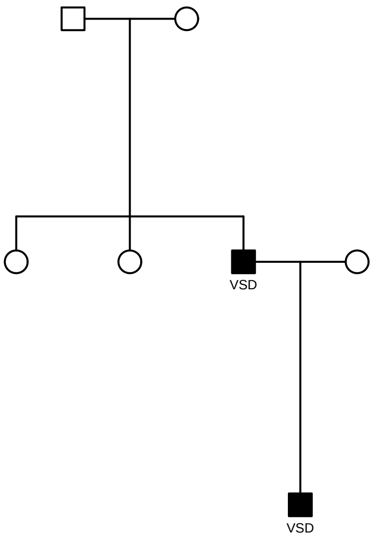

LYS_M137

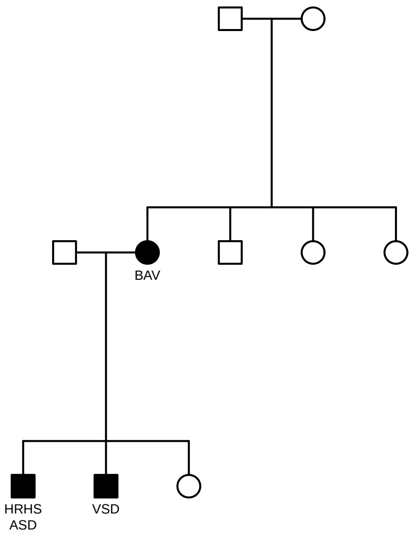

LYS_M138

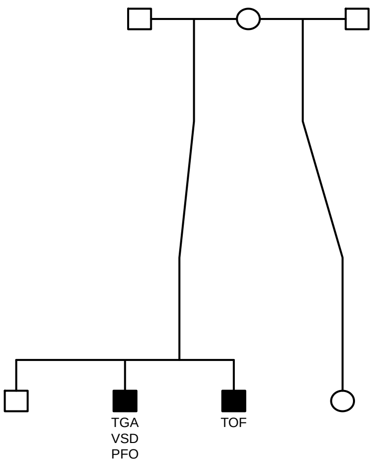

LYS_M139

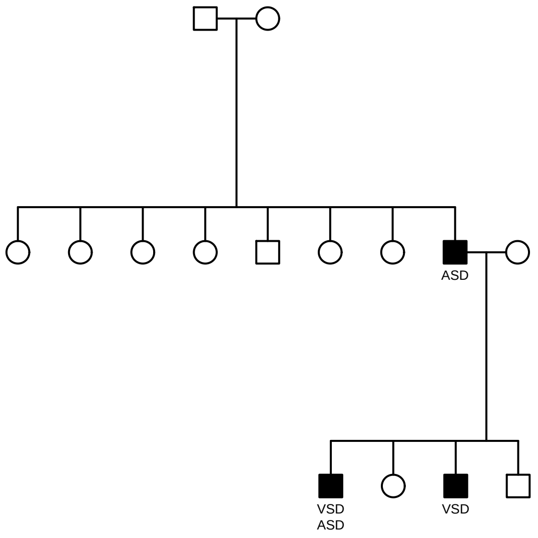

LYS_M140

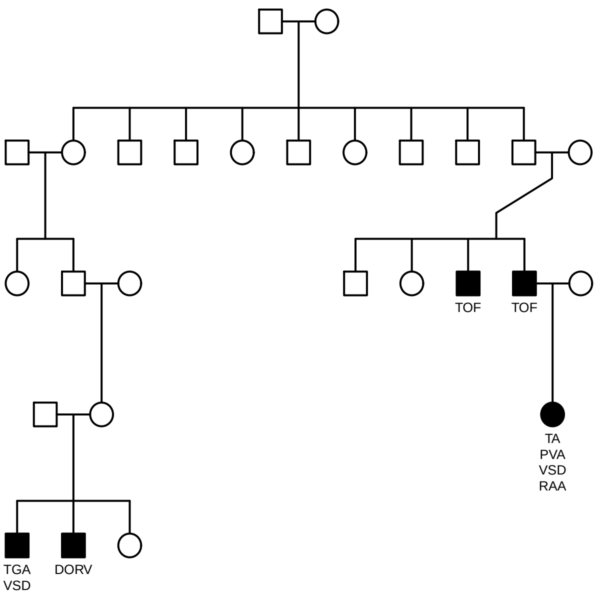

LYS_M143

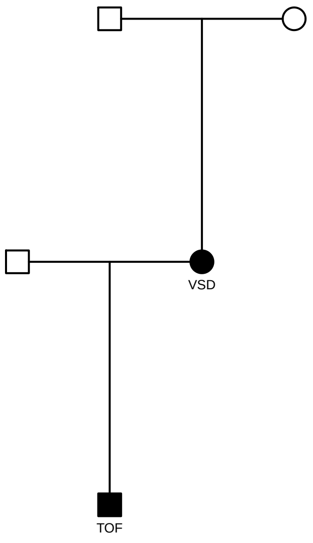

LYS_M146

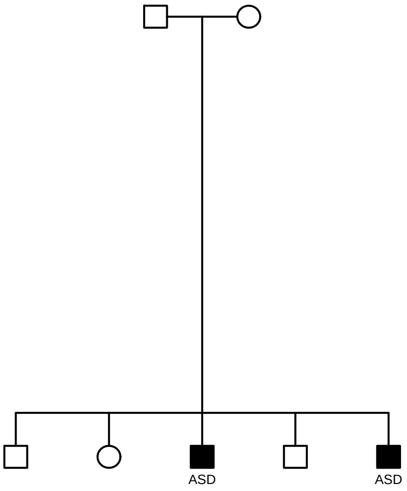

LYS_M147

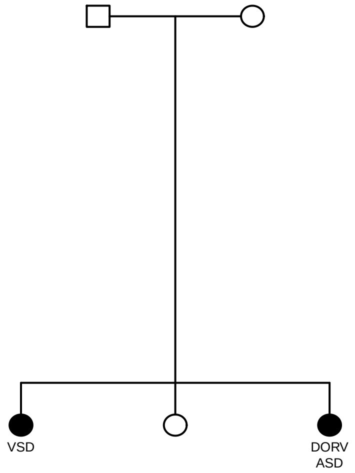

LYS_M148

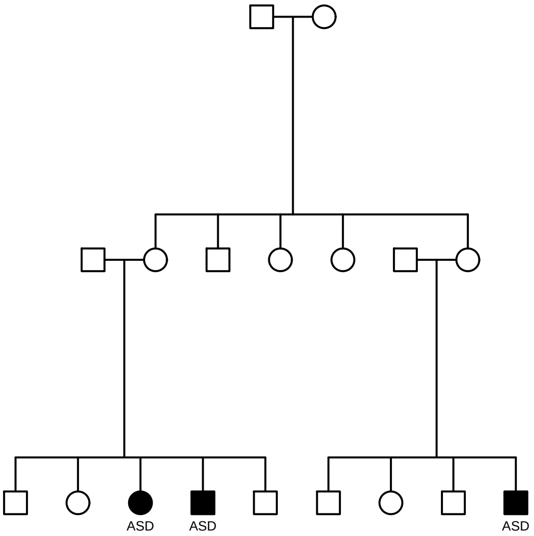

LYS_M151

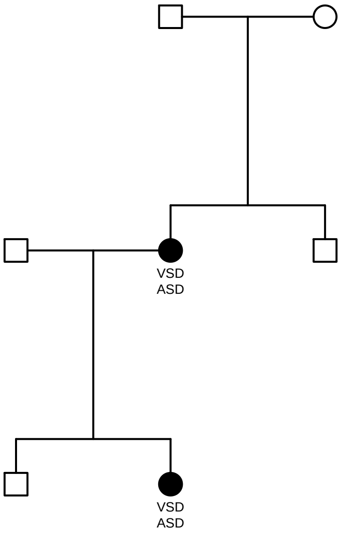

LYS_M153

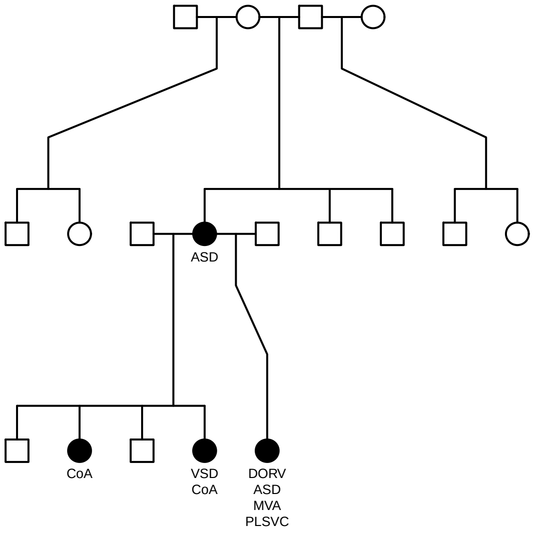

LYS_M155

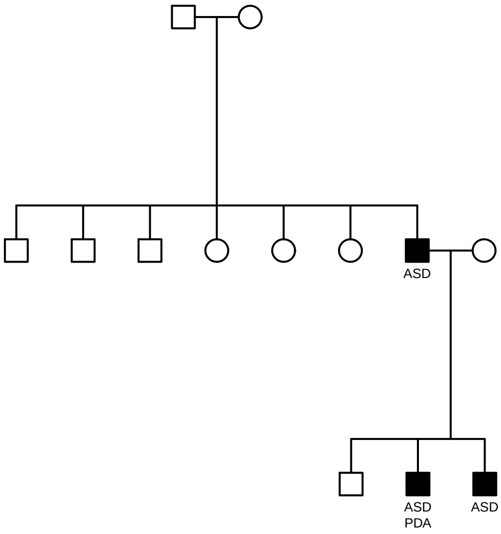

LYS_M156

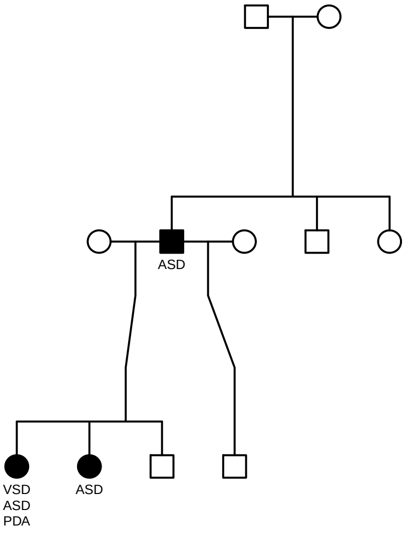

LYS_M157

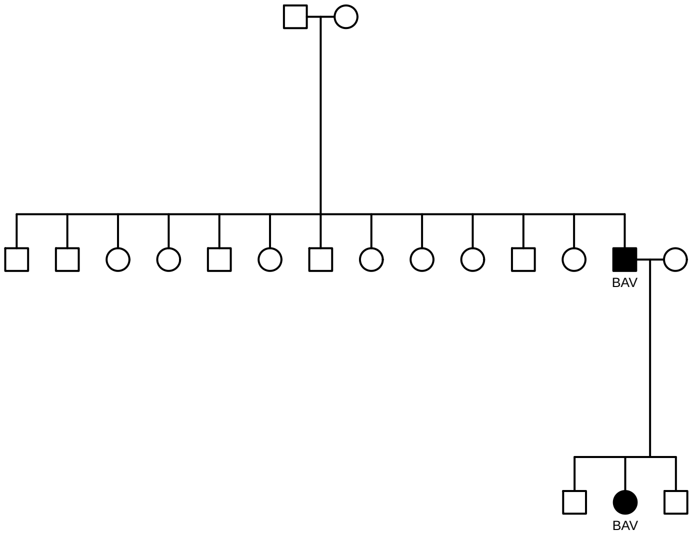

LYS_M158

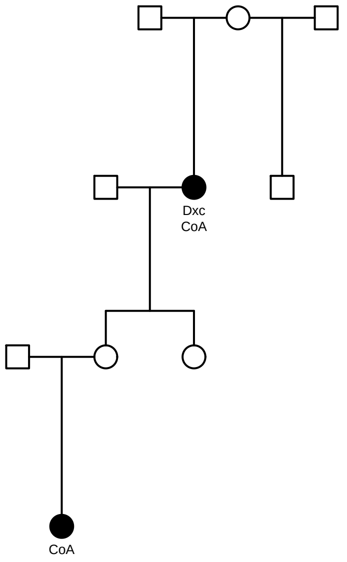

LYS_M159

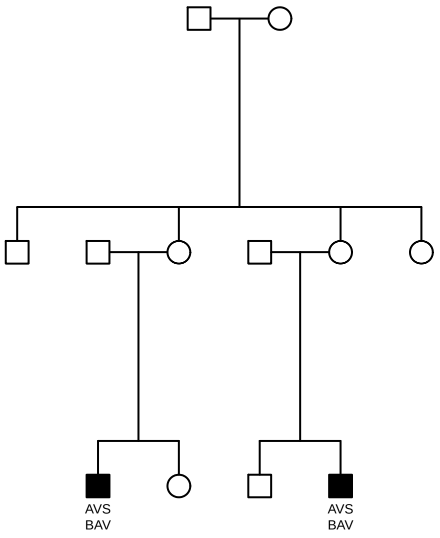

LYS_M162

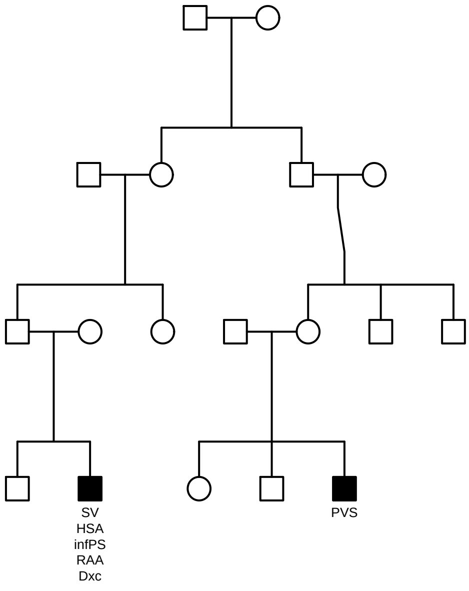

LYS_M163

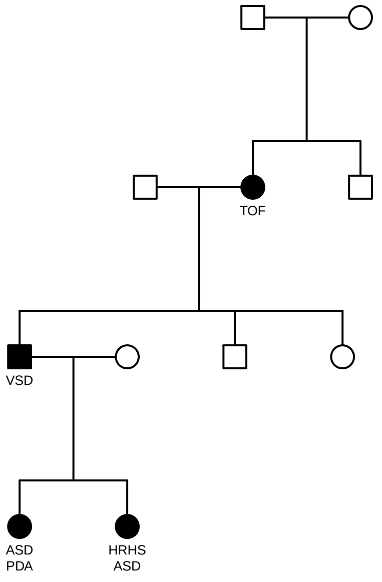

LYS_M165

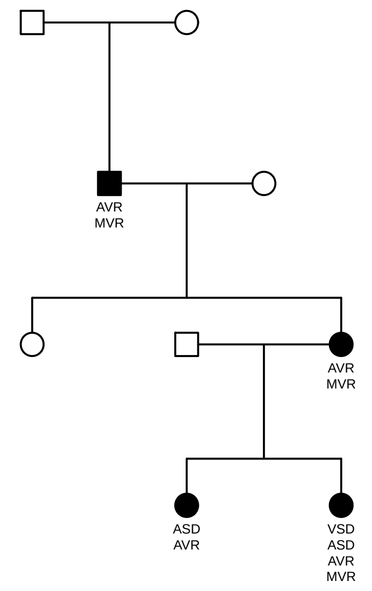

LYS_M166

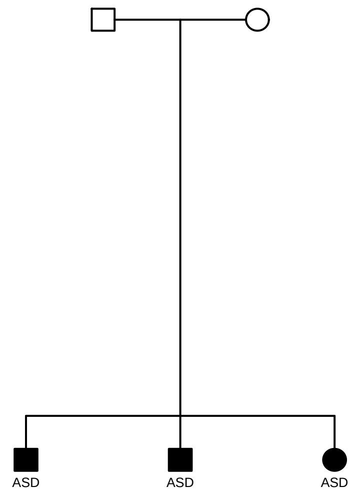

LYS_M169

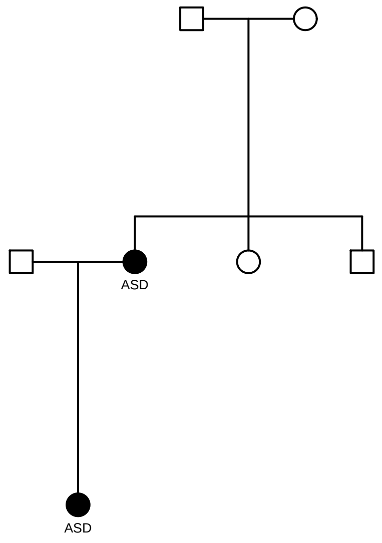

LYS_M171

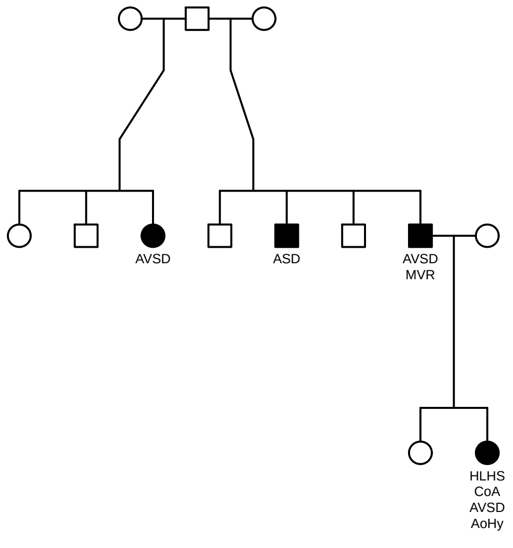

LYS_M172

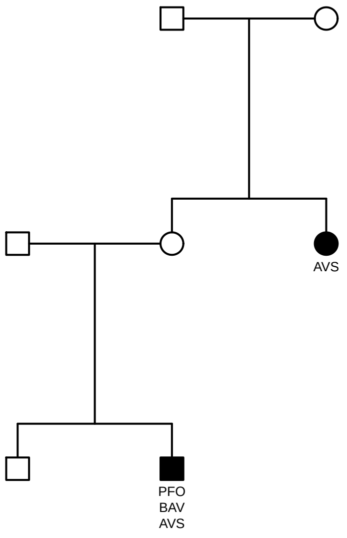

LYS_M173

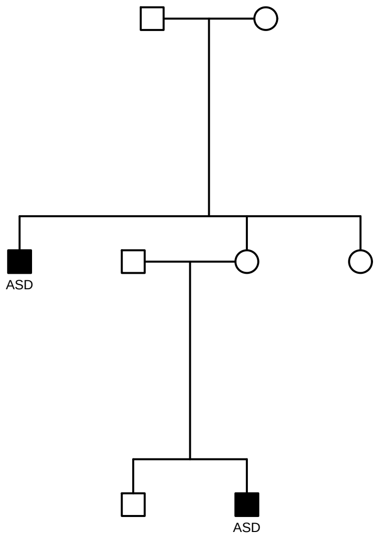

LYS_M174

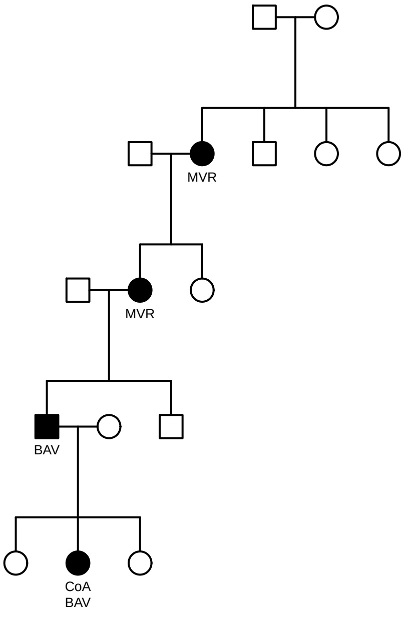

LYS_M175

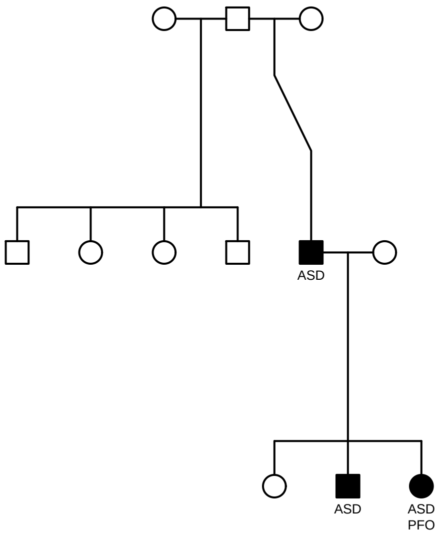

LYS_M176

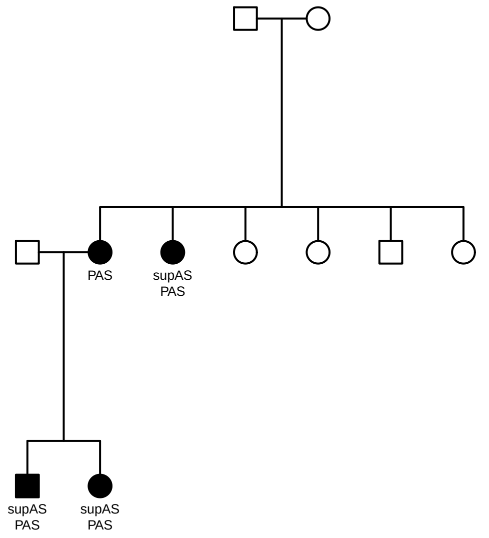

LYS_M180

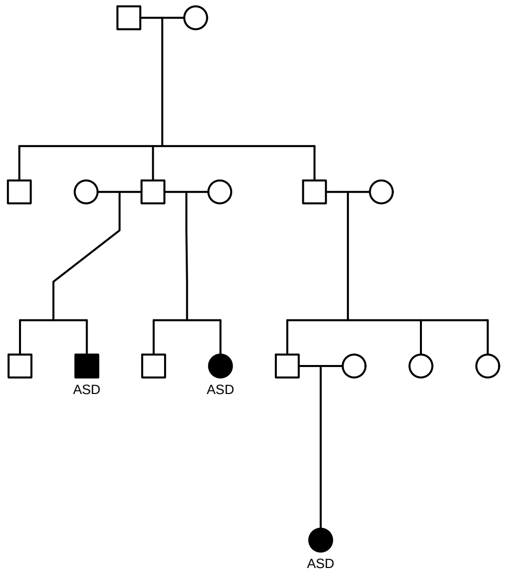

LYS_M181

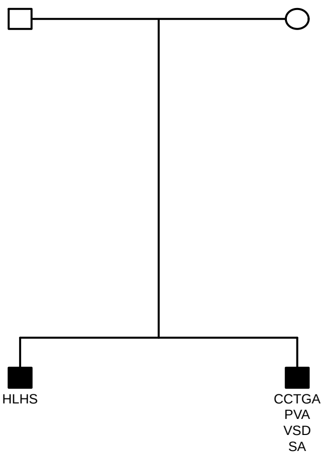

LYS_M182

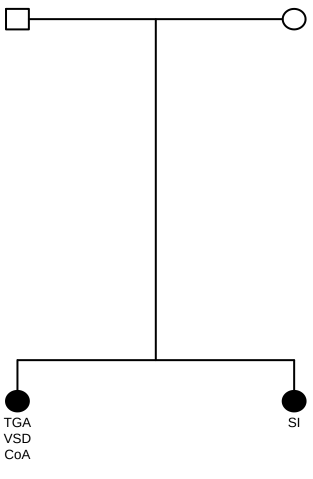

LYS_M185

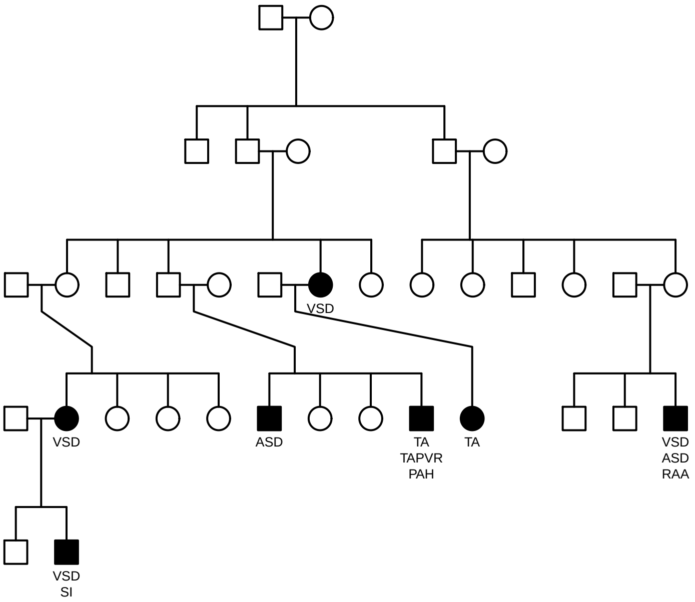

LYS_M186

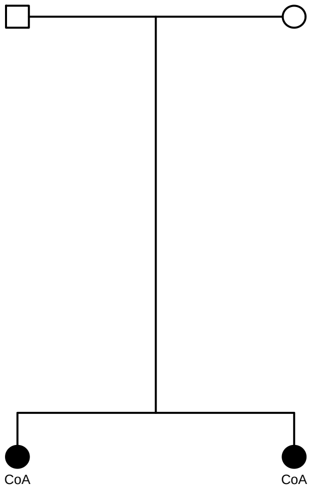

LYS_M187

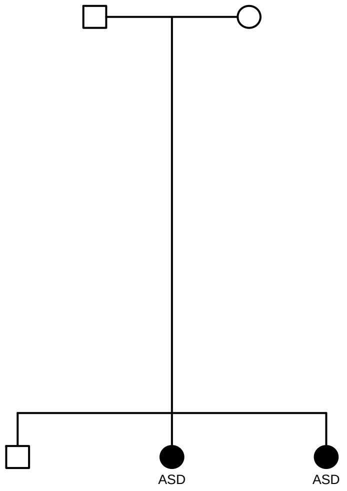

LYS_M188

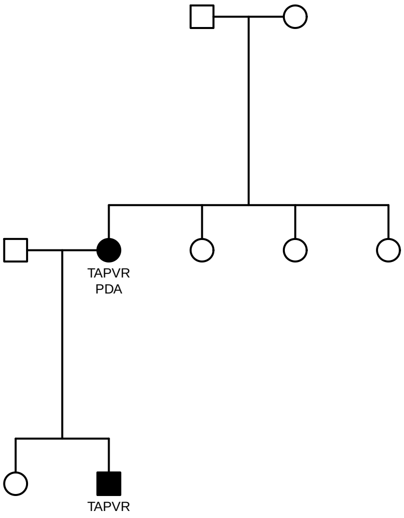

LYS_M189

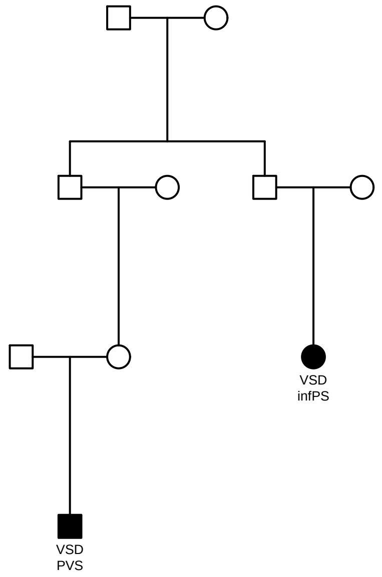

LYS_M190

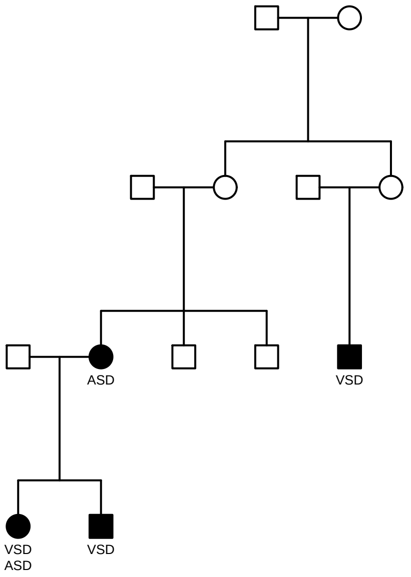

LYS_M191

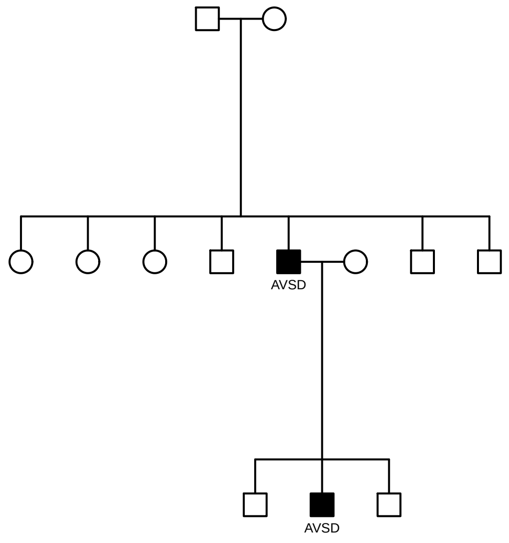

LYS_M192

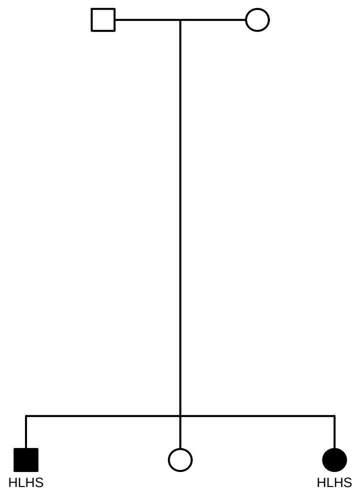

LYS_M193

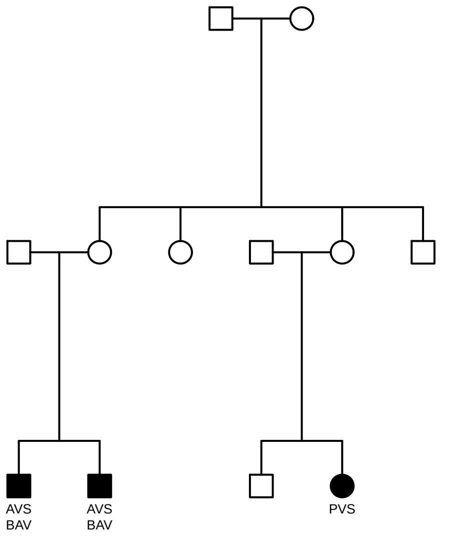

LYS_M208

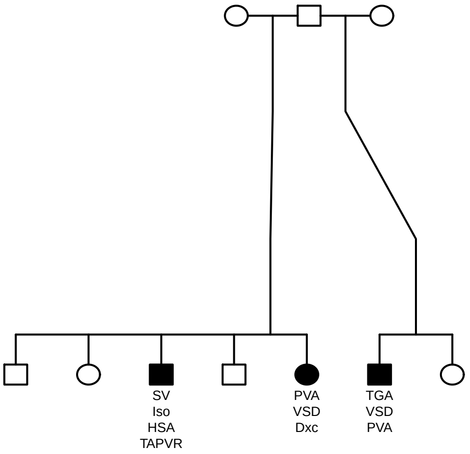

LYS_M215

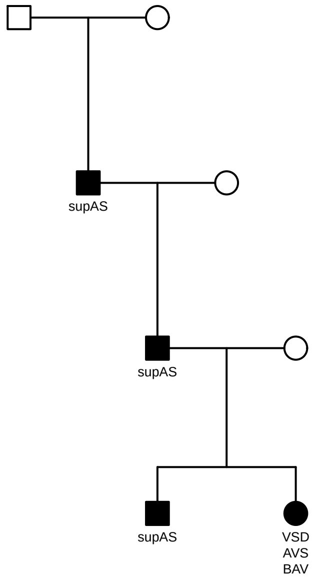

LYS_M216

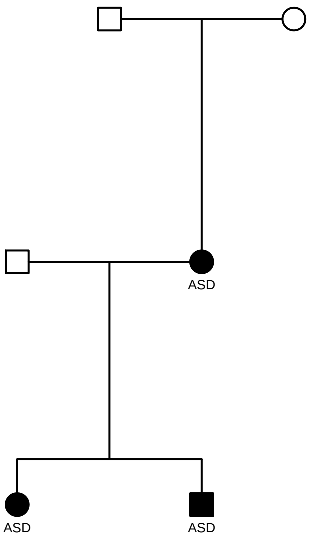

LYS_M217

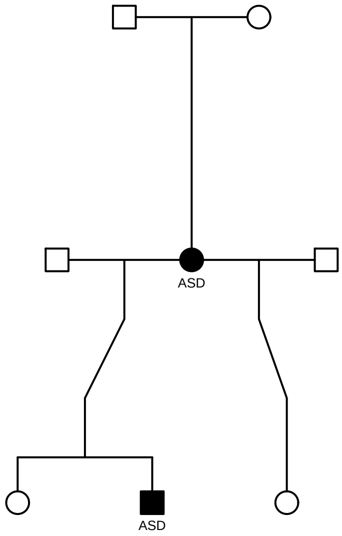

LYS_M218

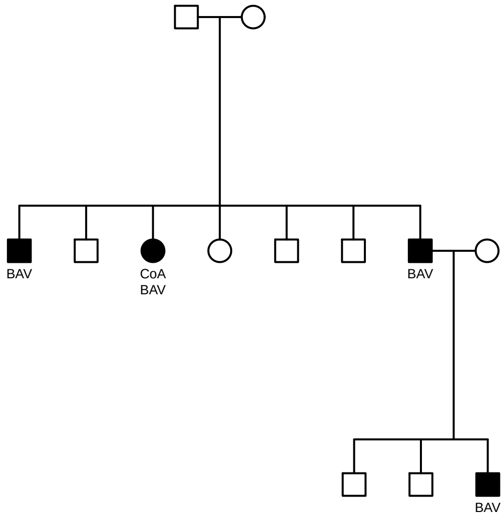

LYS_M220

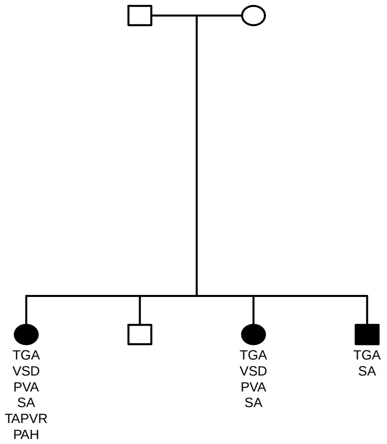

LYS_M221

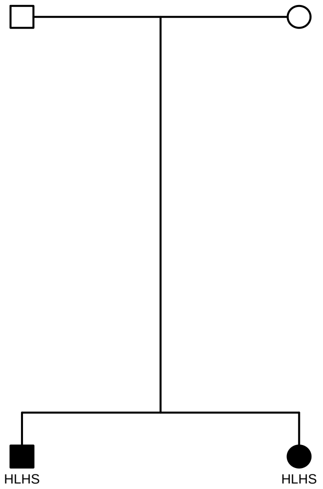

LYS_M222

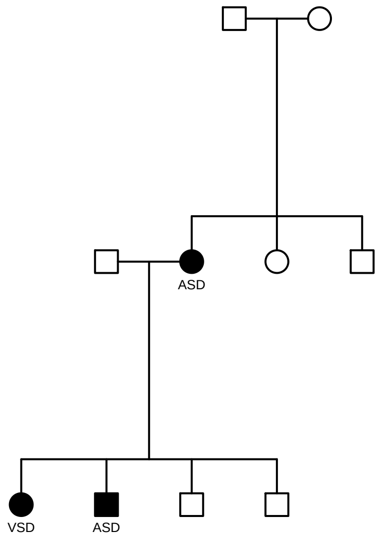

LYS_M223

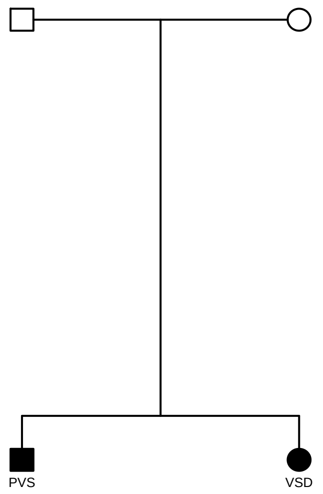

LYS_M224

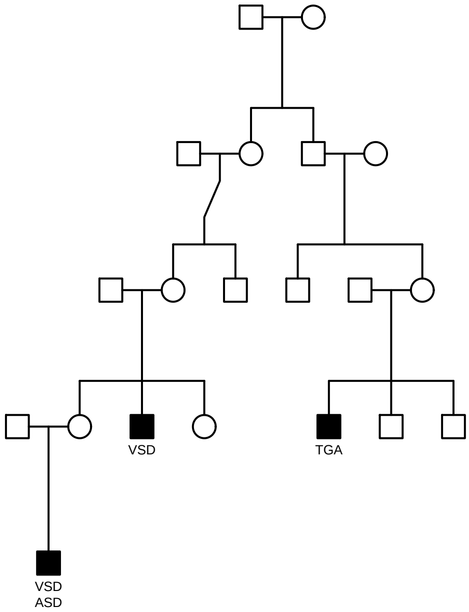

LYS_M225

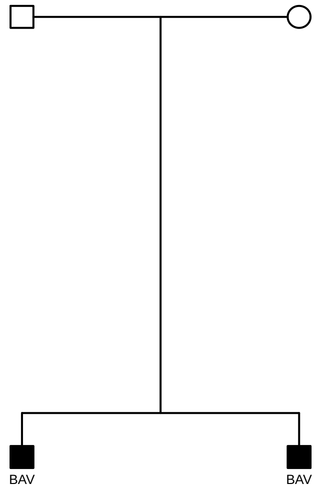

LYS_M226

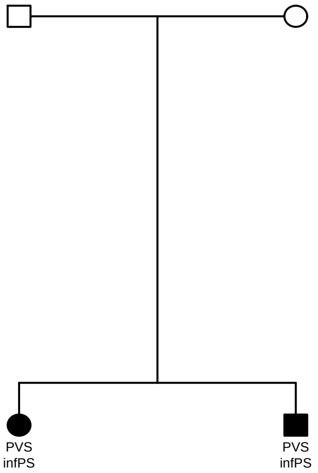

LYS_M228

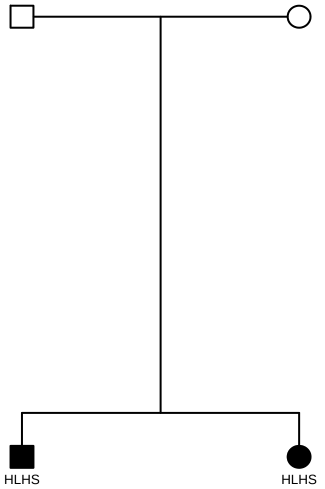

LYS_M230

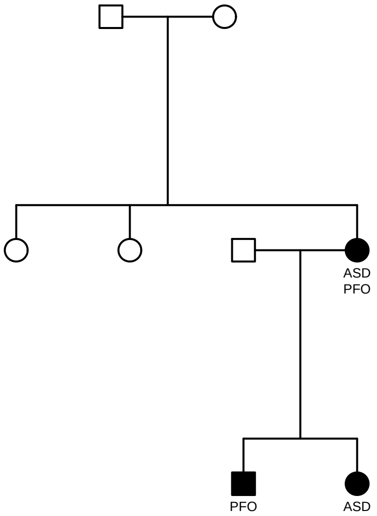

LYS_MC231

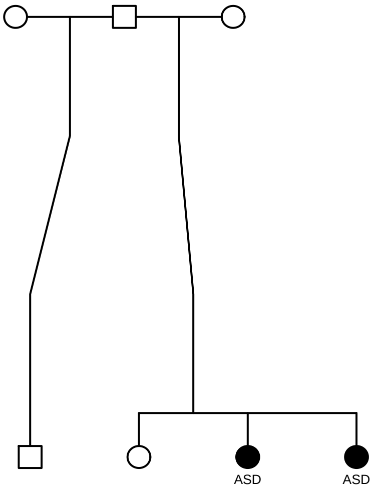

LYS_M232

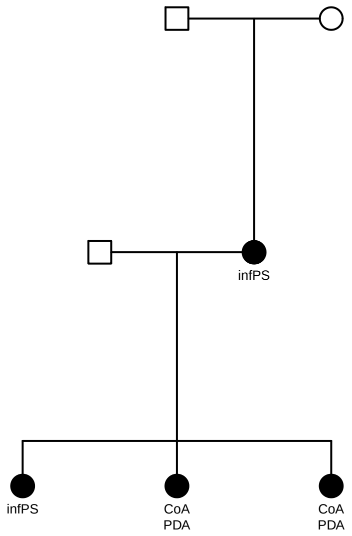

LYS_M233

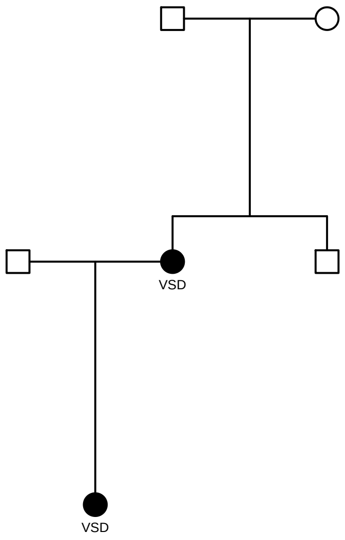

LYS_M234

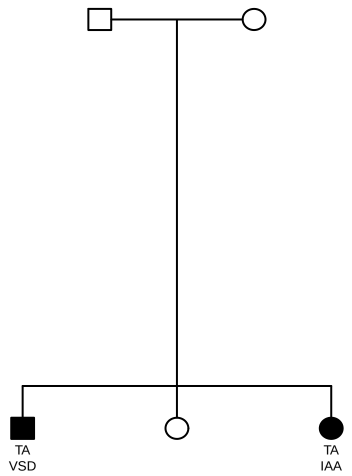

LYS_M235

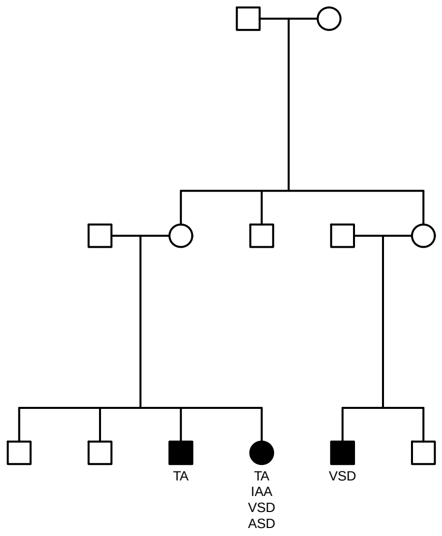

LYS_M236

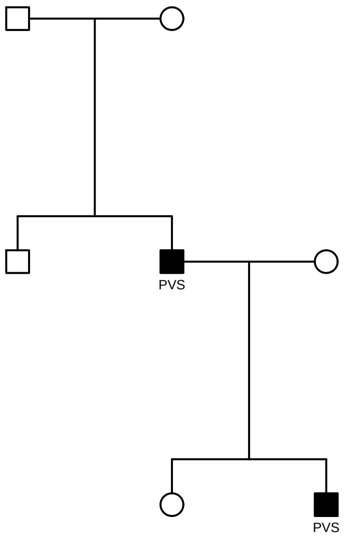

LYS_M237

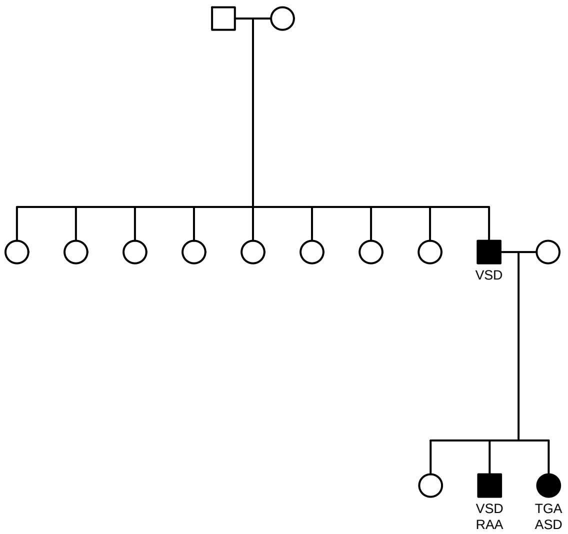

LYS_M238

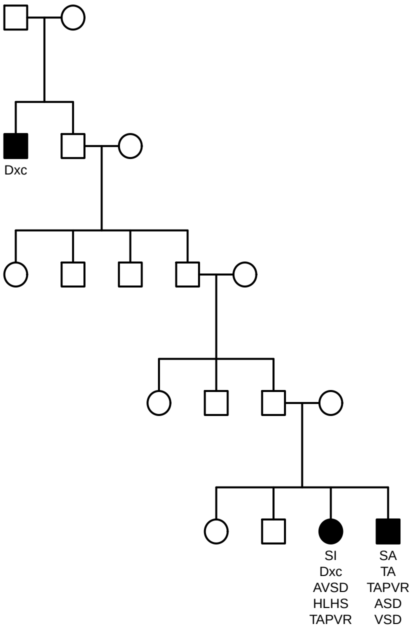

LYS_M239

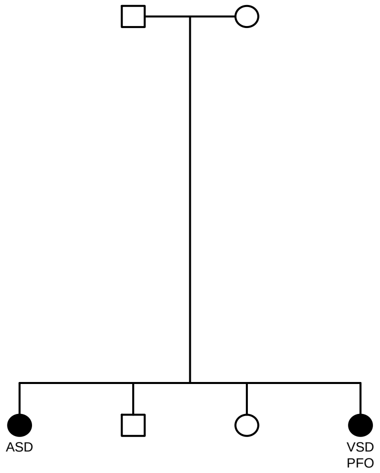

LYS_M240

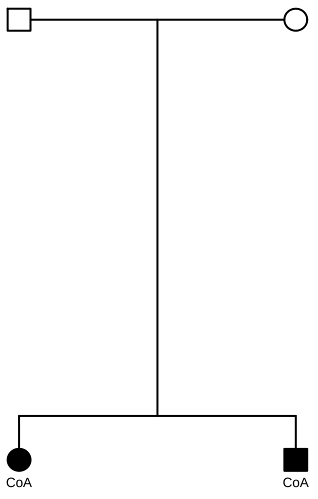

LYS_M242

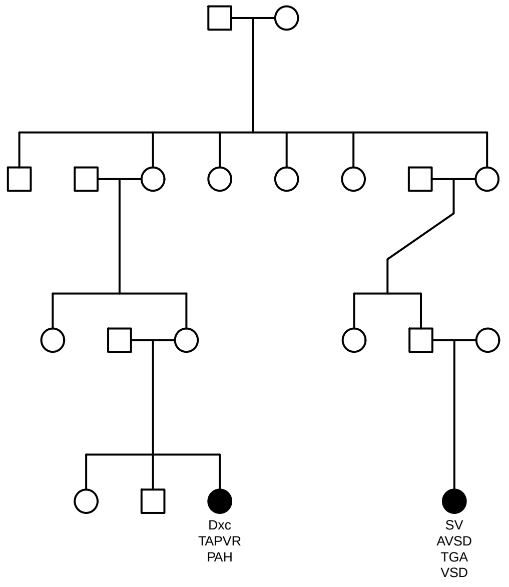

Loffredo_100

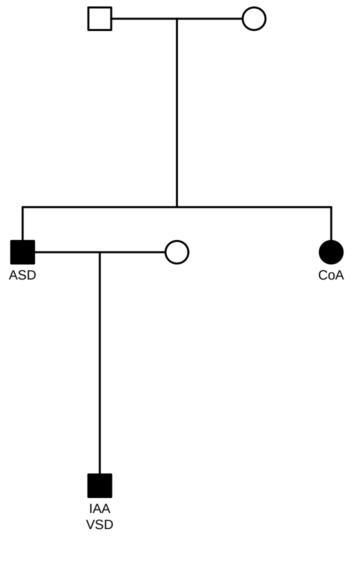

Loffredo_101

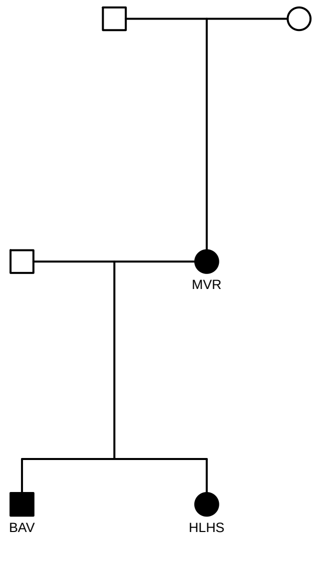

Loffredo_106

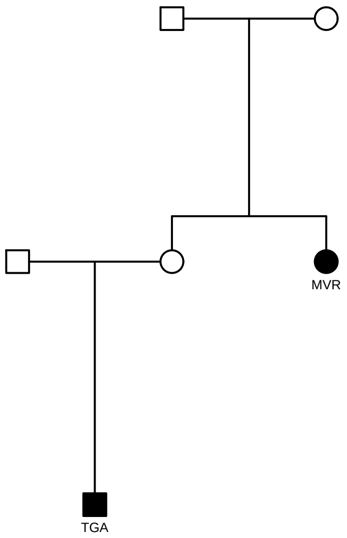

Loffredo_110

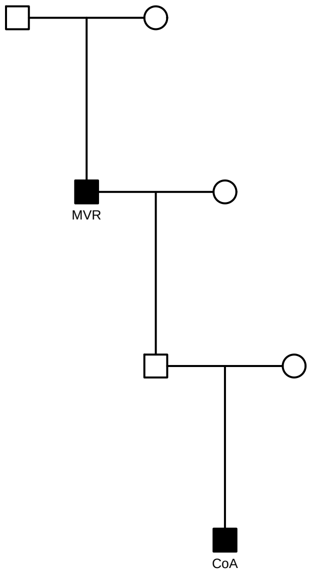

Loffredo_113

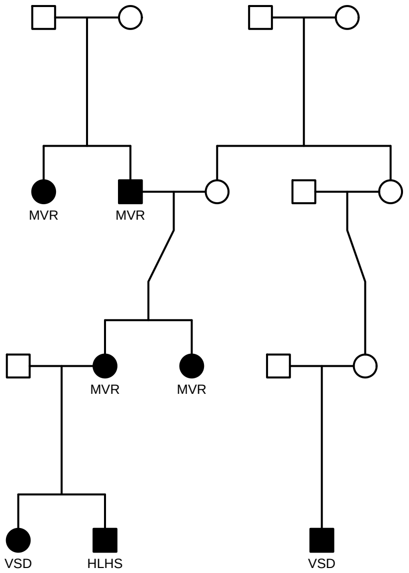

Loffredo_116

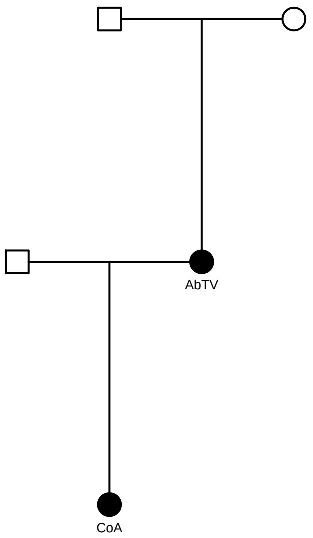

Loffredo_120

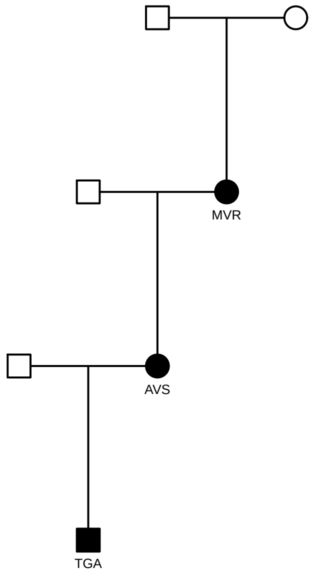

Loffredo_121

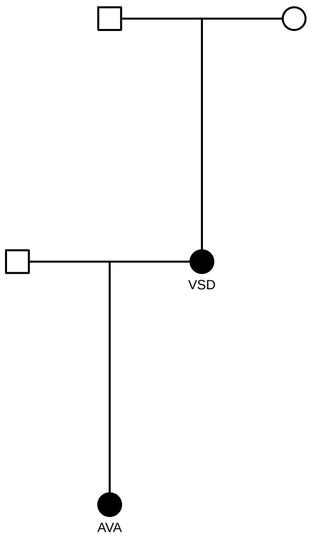

Loffredo_122

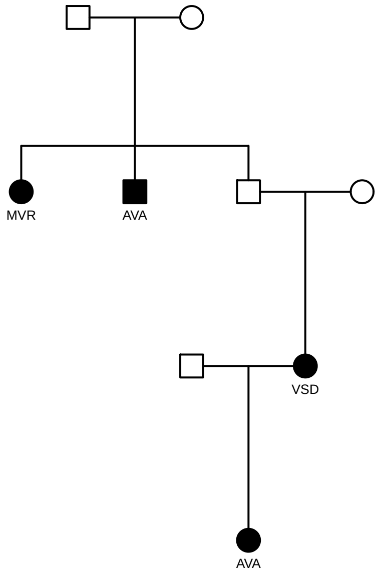

Loffredo_123

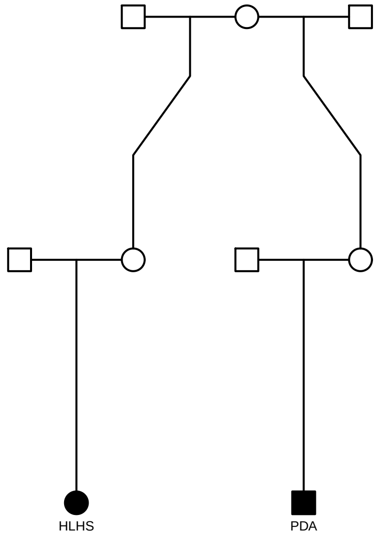

Loffredo_125

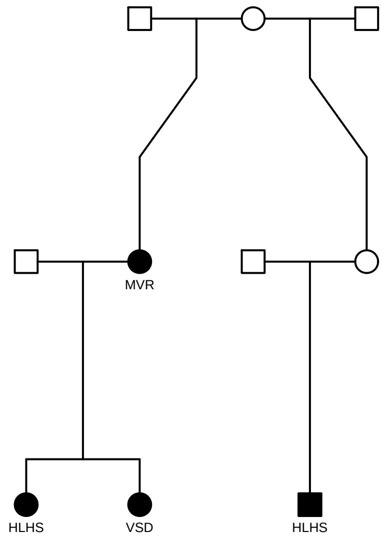

Loffredo_128

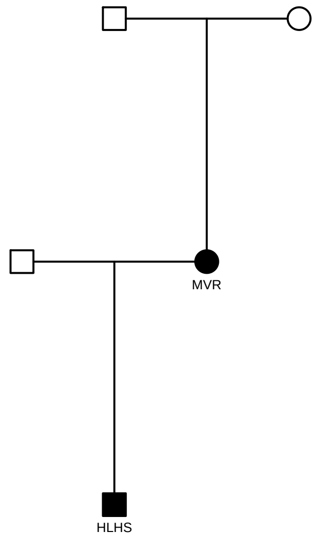

Loffredo_137

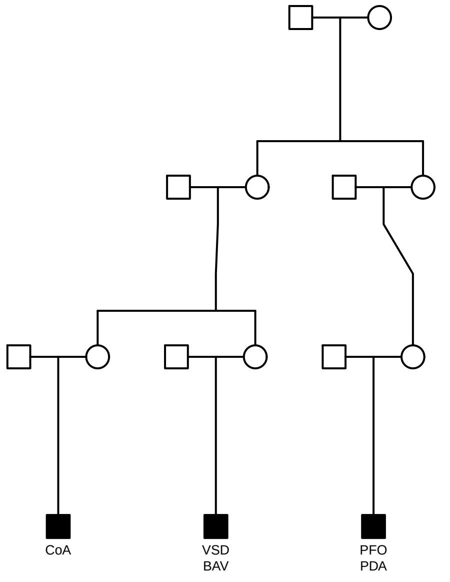

Loffredo_140

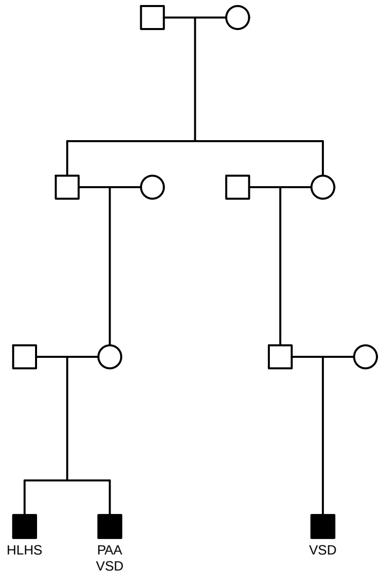

Loffredo_141

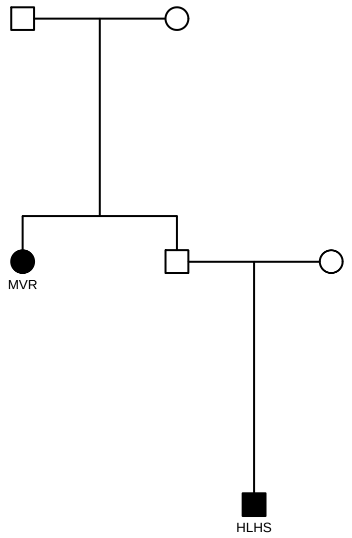

Loffredo_142

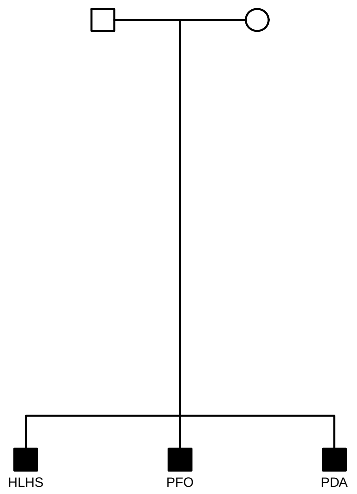

Loffredo_145

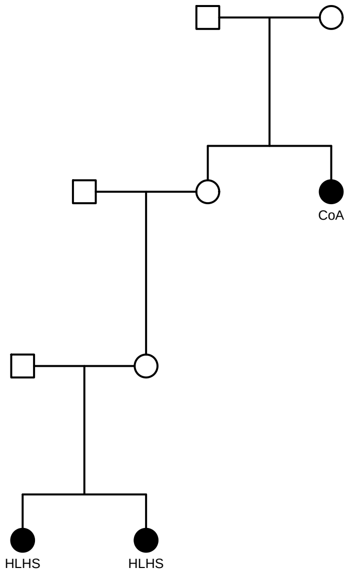

Loffredo_147

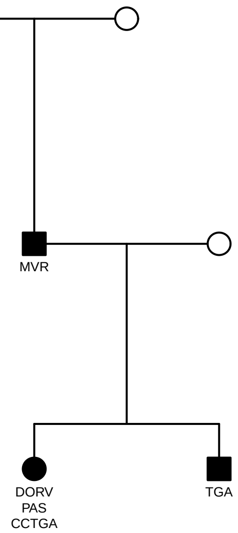

Loffredo_151

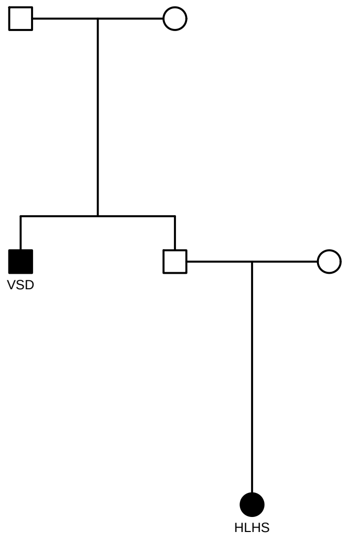

Loffredo_154

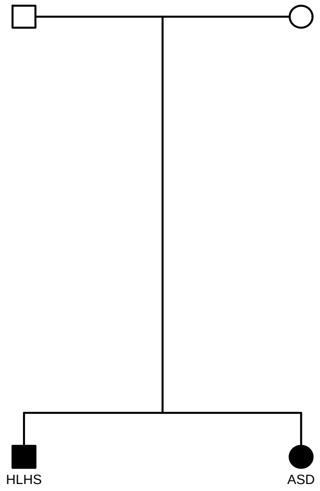

Loffredo_155

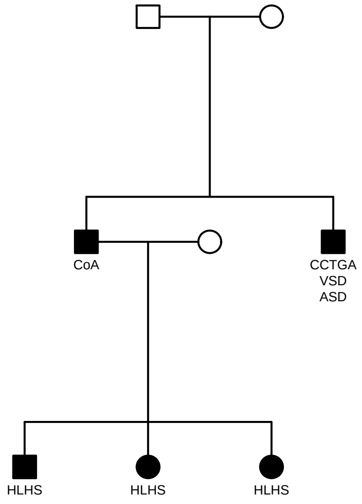

Loffredo_160

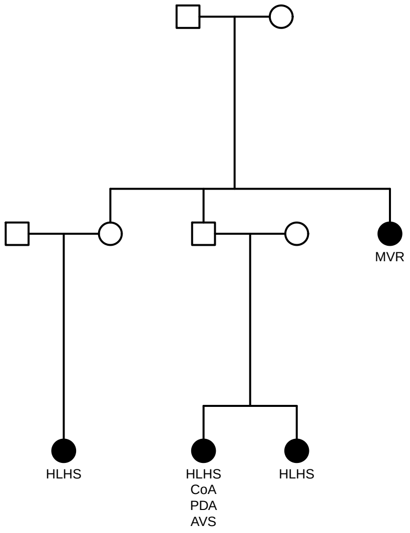

Loffredo_166

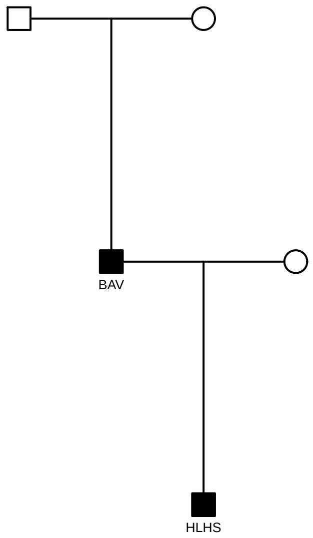

Loffredo_167

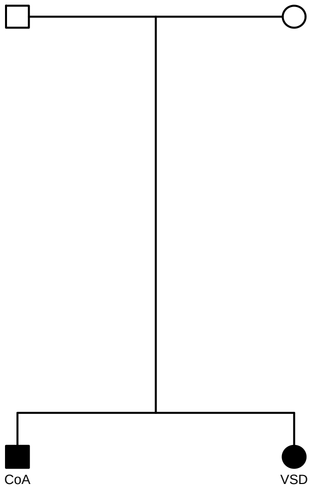

Loffredo_300

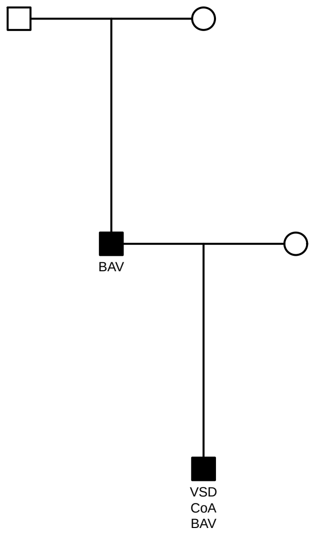

Loffredo_304

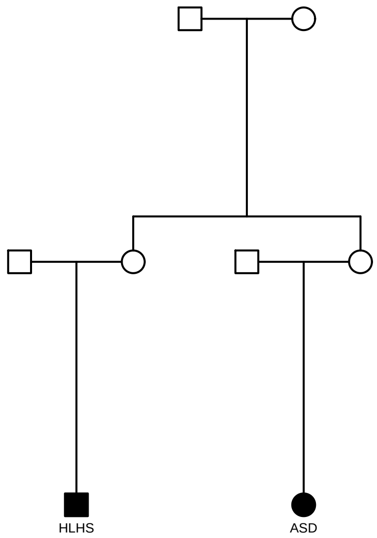

Loffredo_401

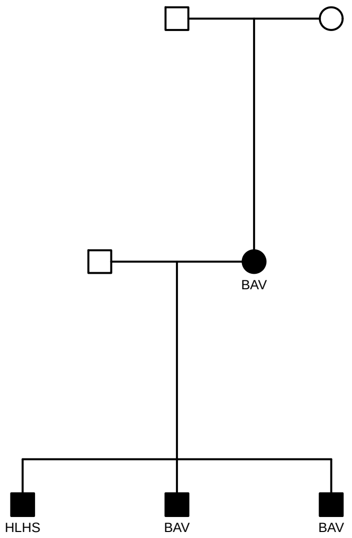

Loffredo_403

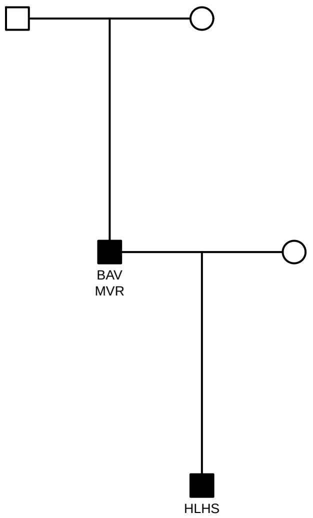

Loffredo_406

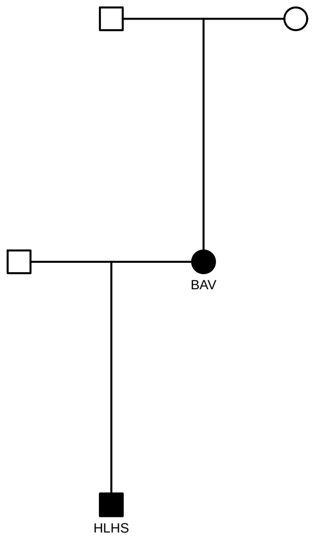

Loffredo_408

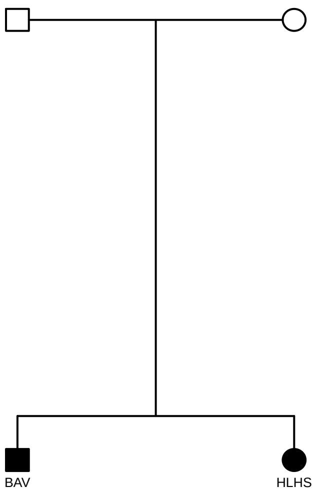

Loffredo_424

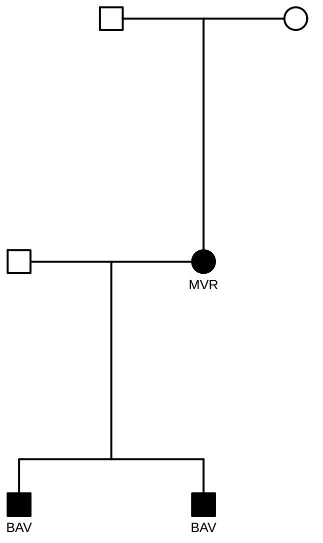

Loffredo_429

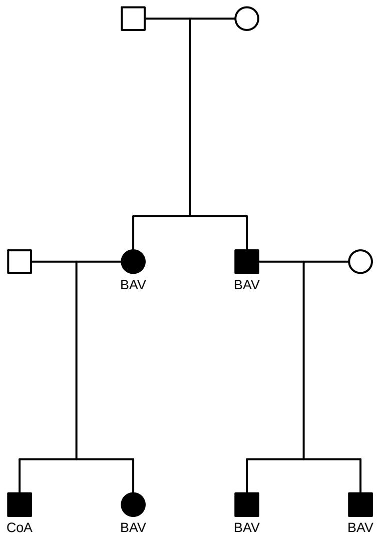

Loffredo_430

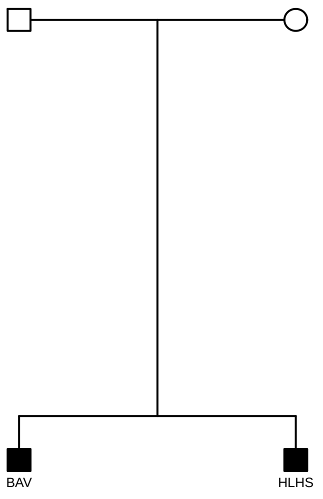

Loffredo_431

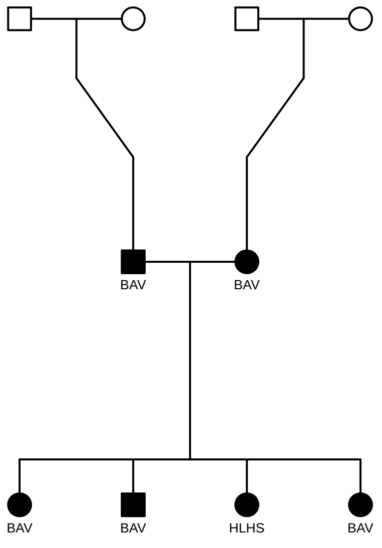

Loffredo_432

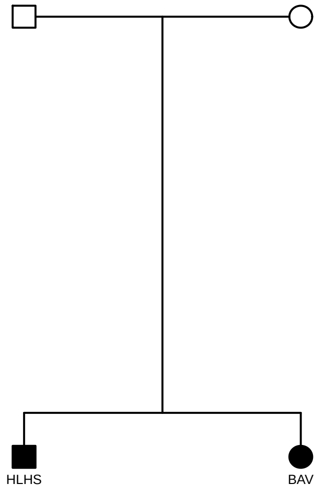

Loffredo_484

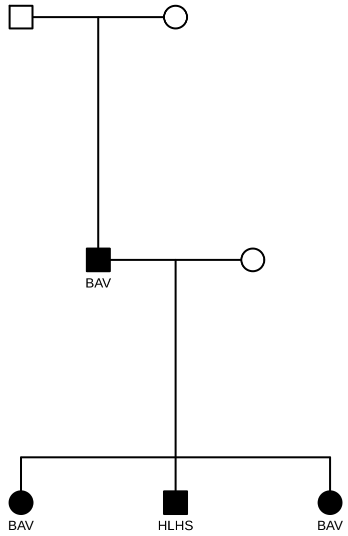

Loffredo_486

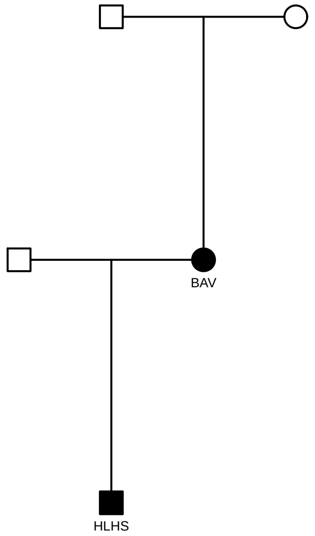

Loffredo_487

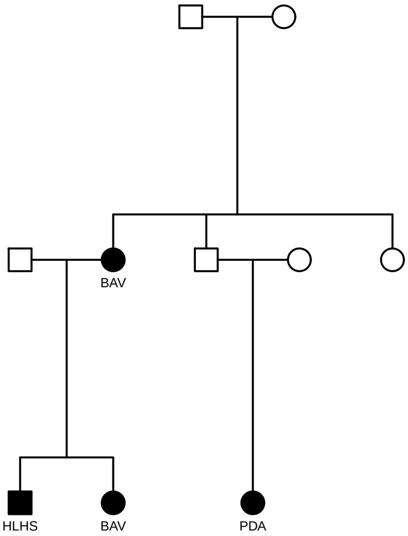

Loffredo_490

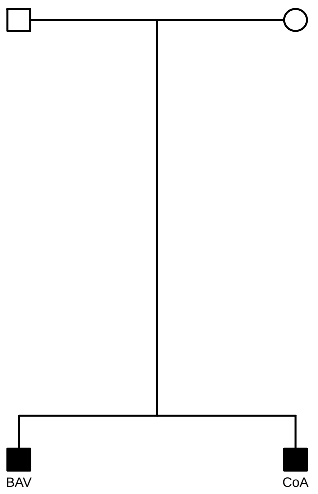

Loffredo_491

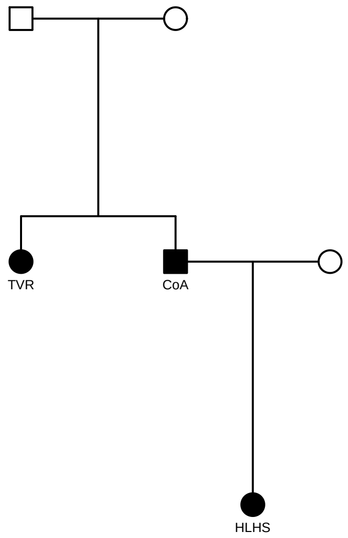

Loffredo_493

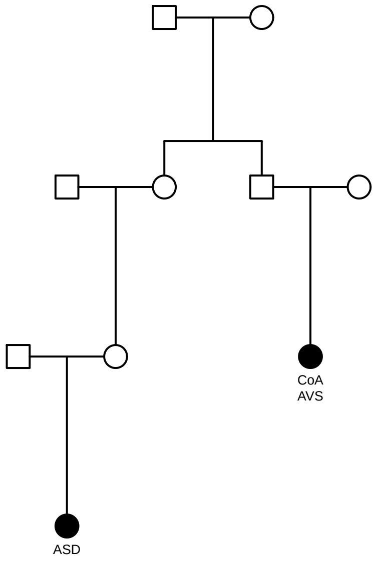

Loffredo_503

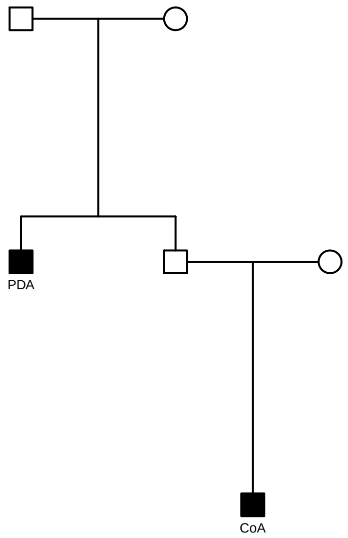

Loffredo_504

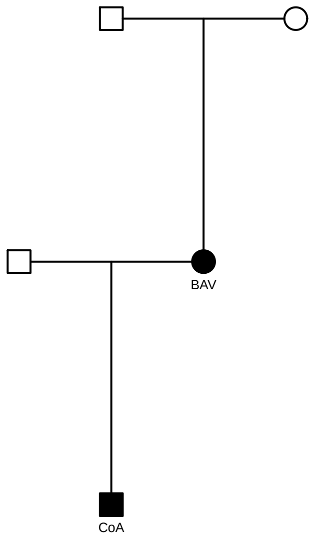

Loffredo_507

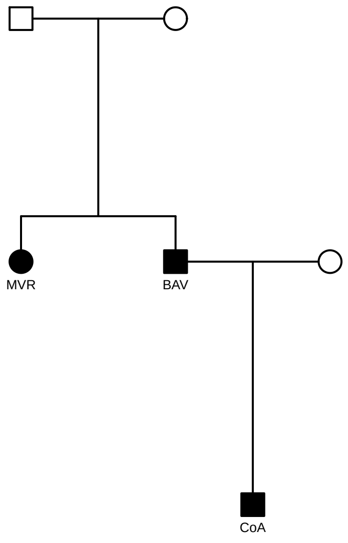

Loffredo_509

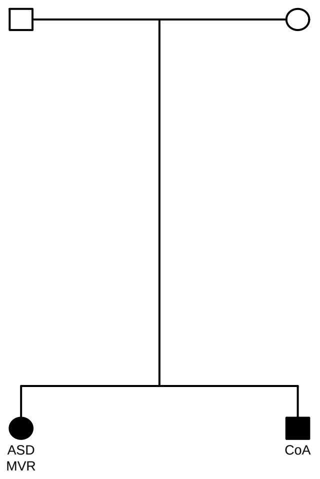

Loffredo_510

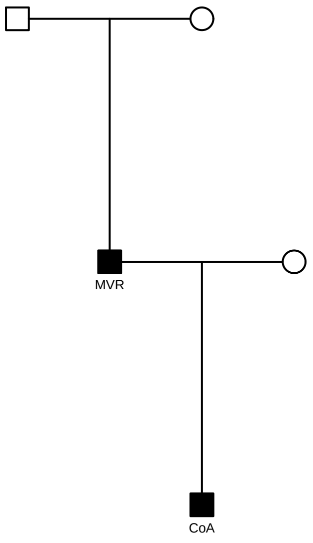

Loffredo_512

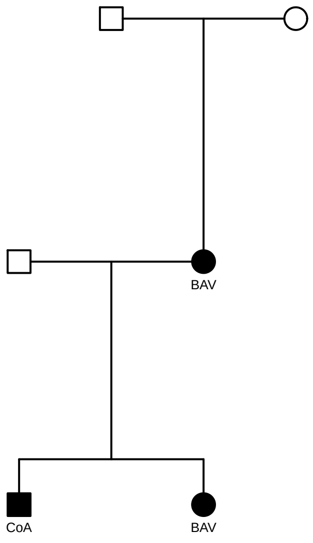

Loffredo_515

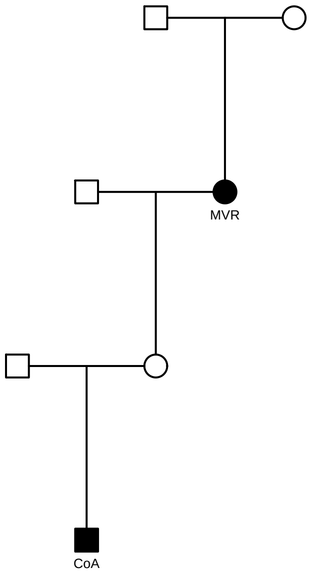

Loffredo_522

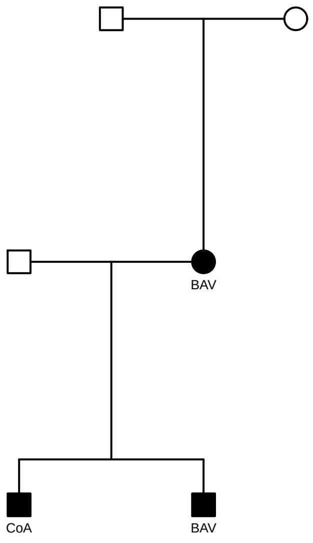

Loffredo_536

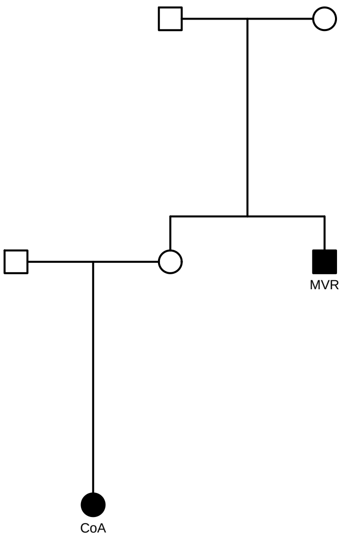

Loffredo_547

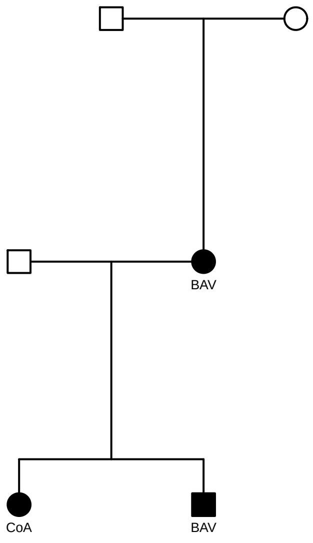

Loffredo_561

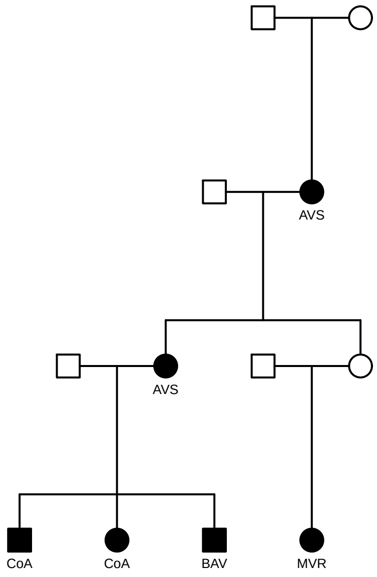

Loffredo_570

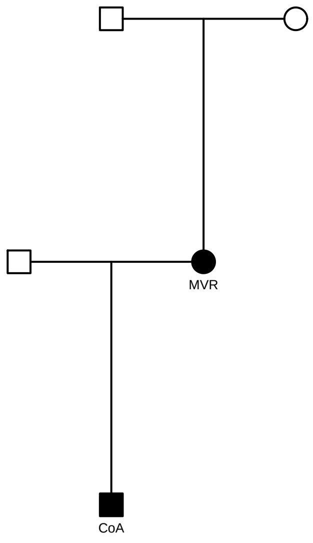

Loffredo_572

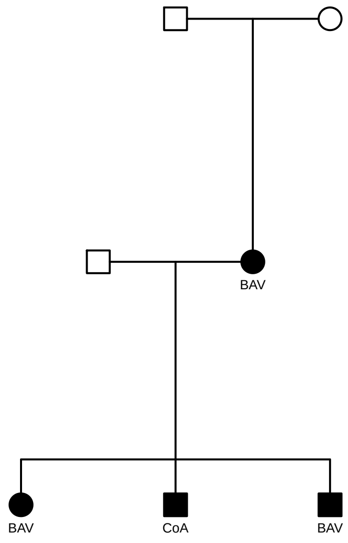

Loffredo_589

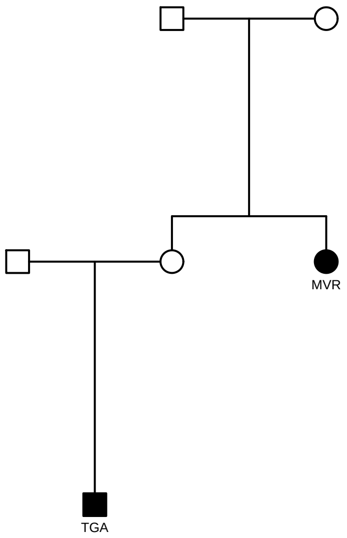

Loffredo_604

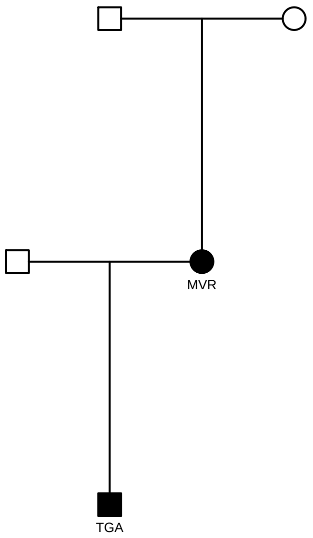

Postma_041

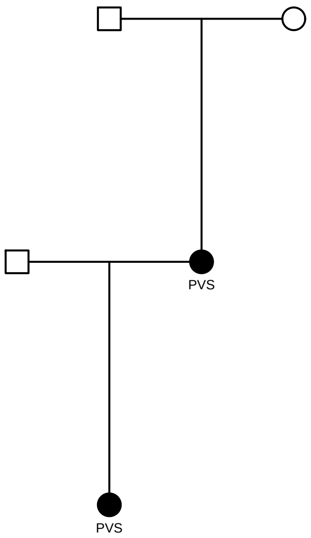

Postma_150

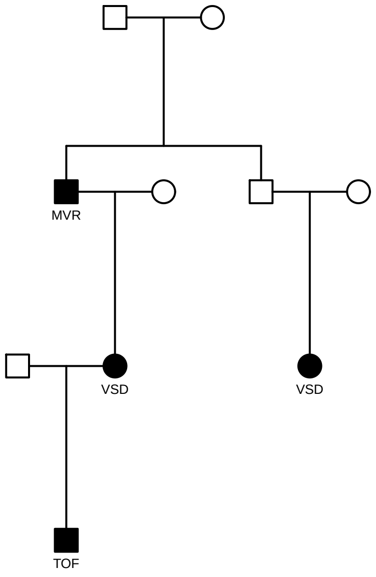

Postma_190

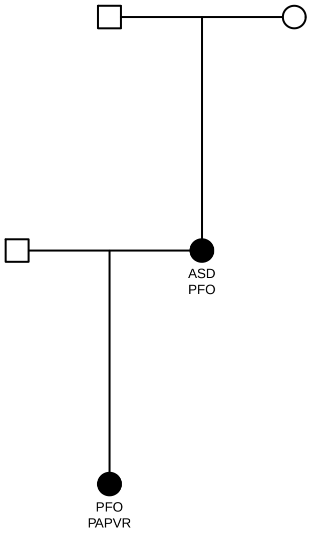

Postma_217

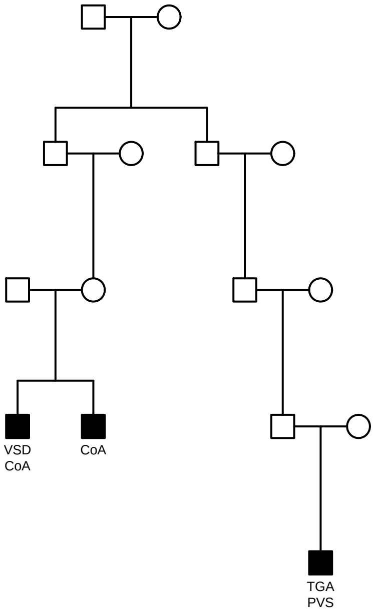

Postma_251

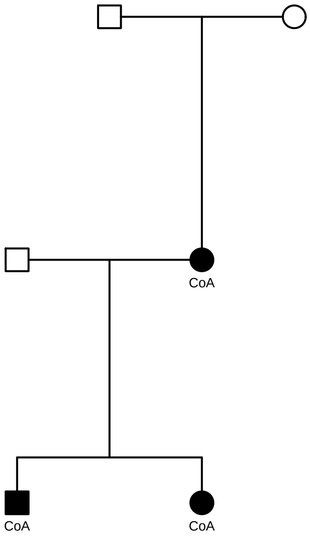

Postma_270

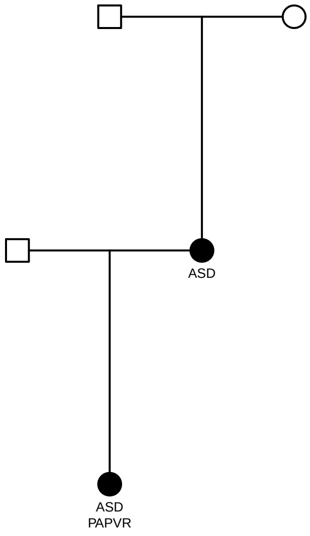

Postma_333

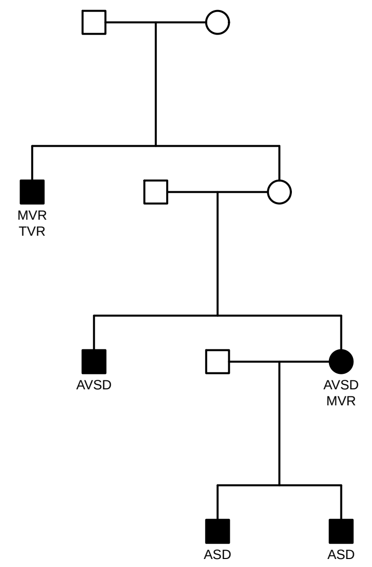

Postma_344

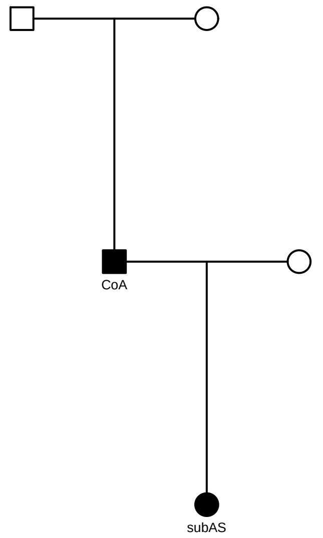

Postma_404

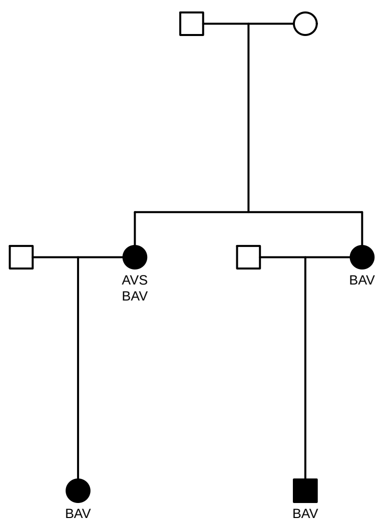

Postma_419

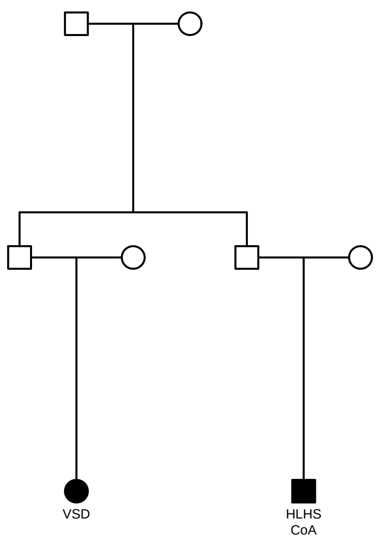

Postma_469

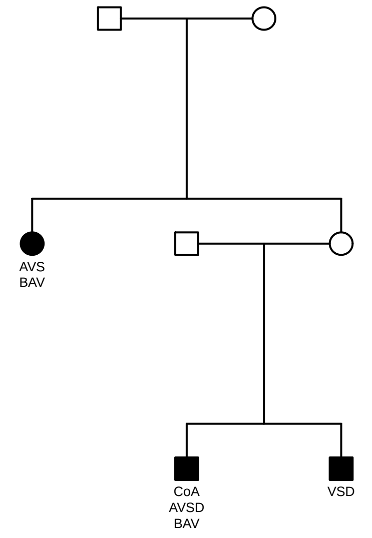

Postma_484

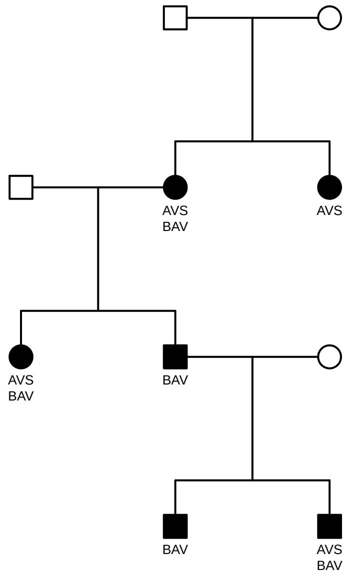

Postma_536

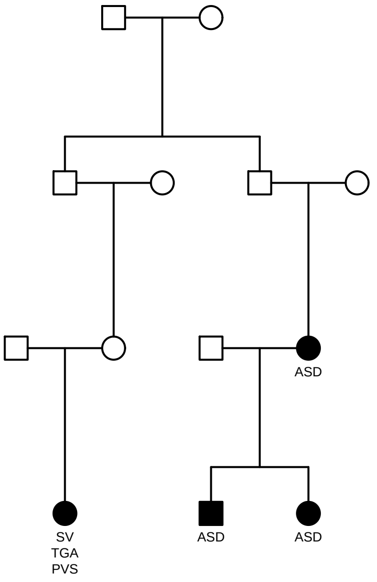

Postma_538

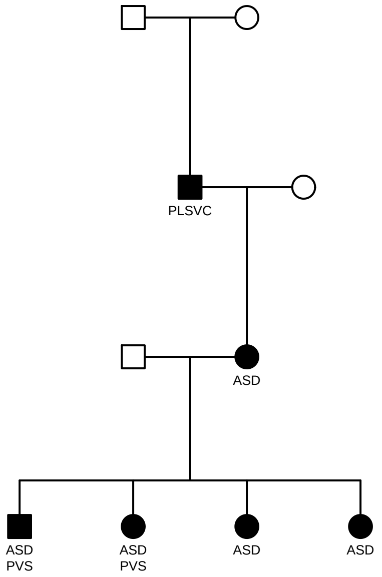

Postma_755

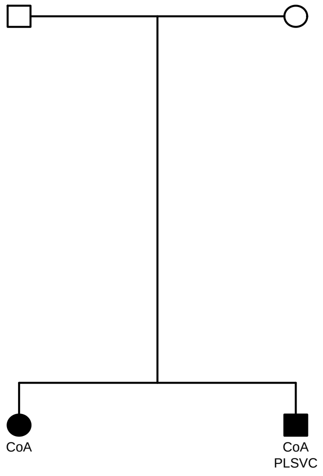

Postma_801

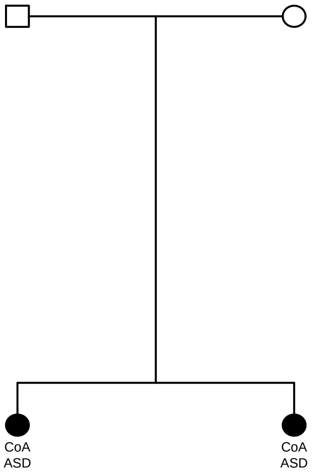

Postma_805

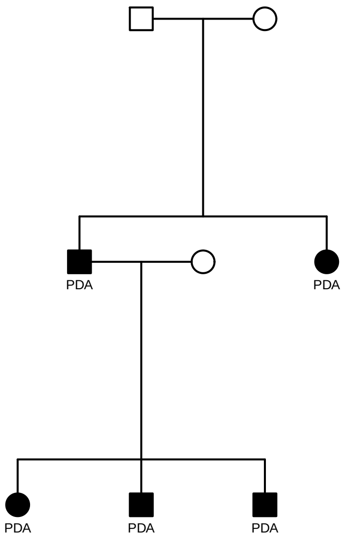

Postma_820

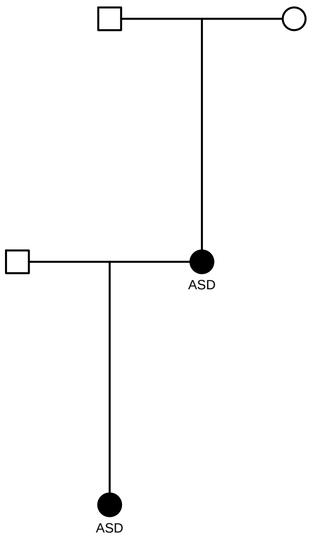

Postma_838

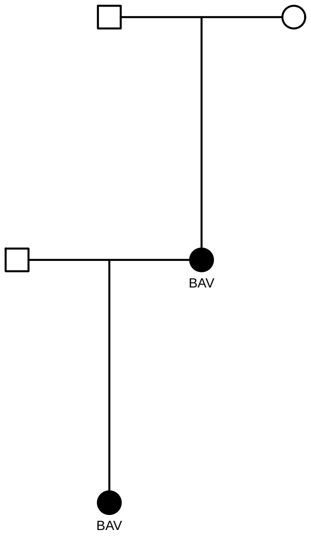

Postma_022

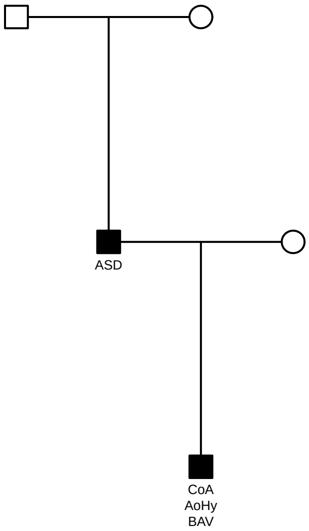

Postma_052

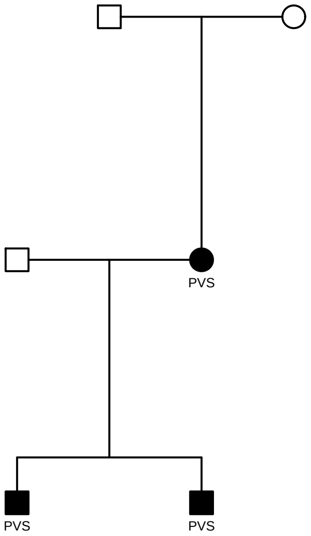

Postma_054

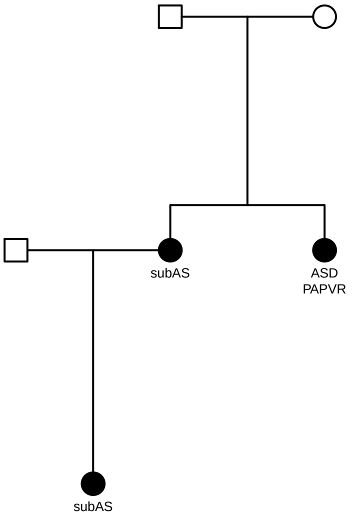

Postma_107

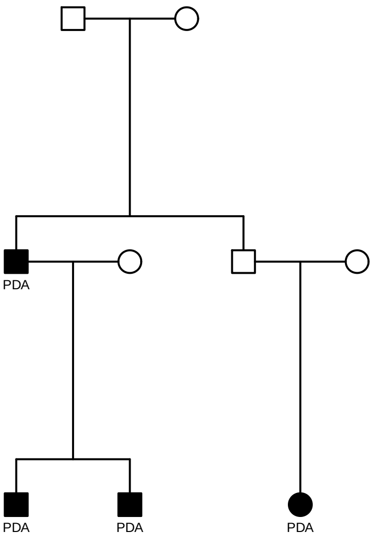

Postma_146

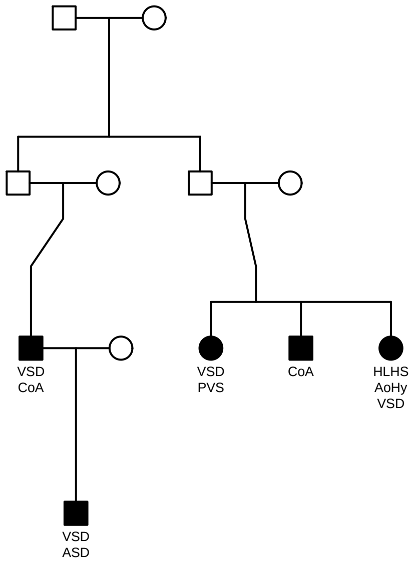

Postma_193

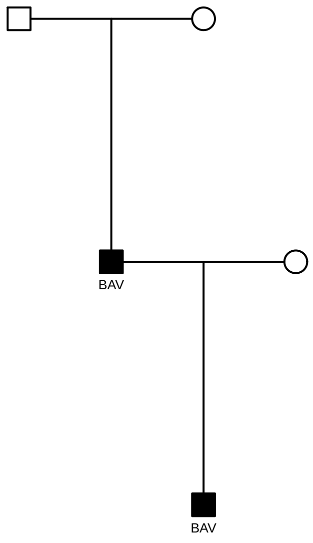

Postma_204

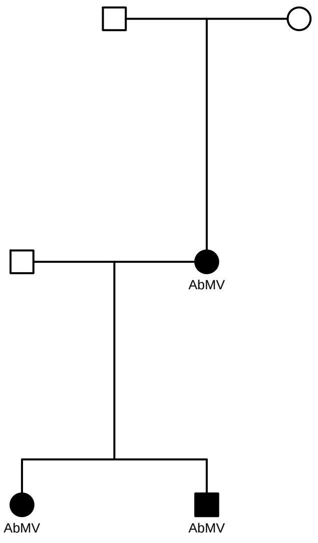

Postma_240

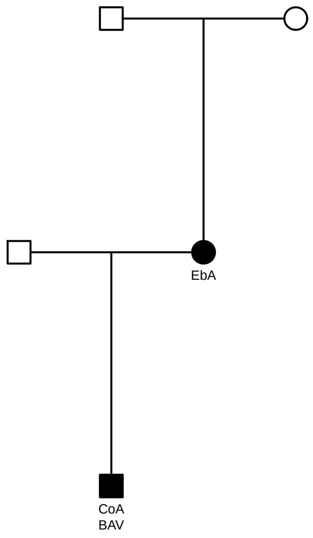

Postma_247

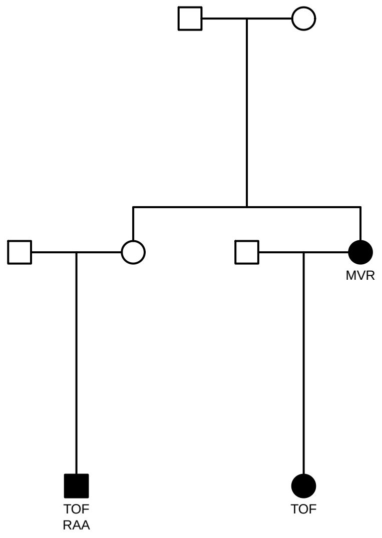

Postma_292

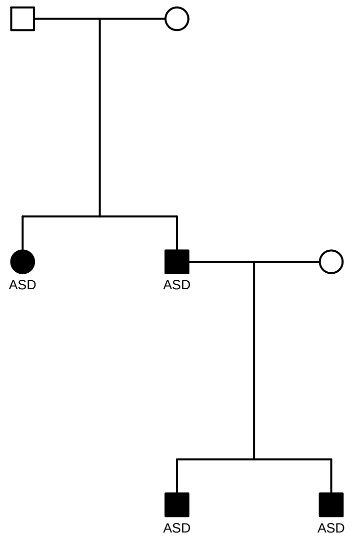

Postma_365

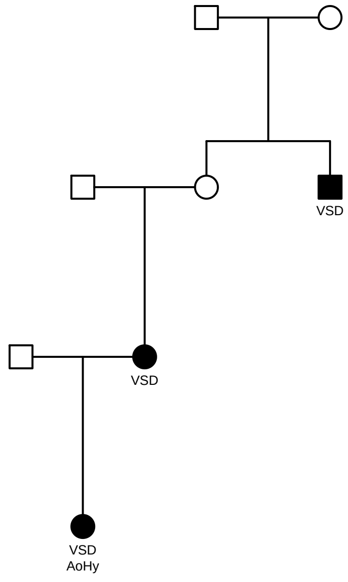

Postma_368

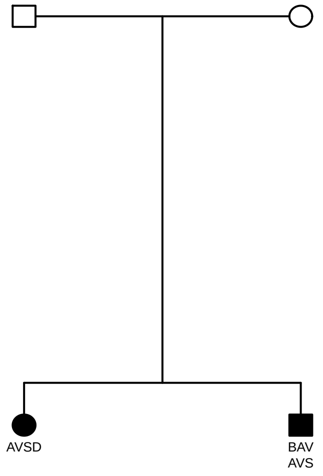

Postma_418

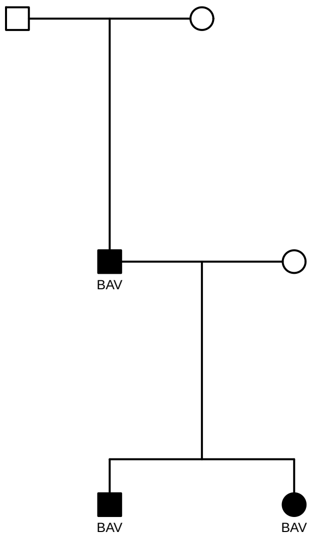

Postma_472

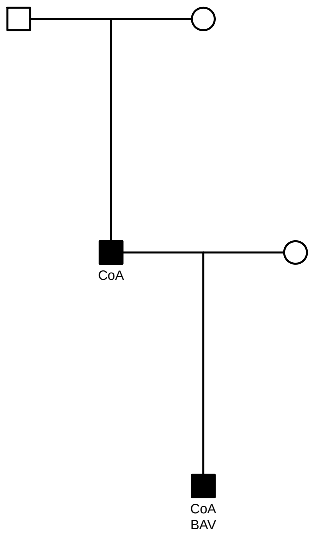

Postma_511

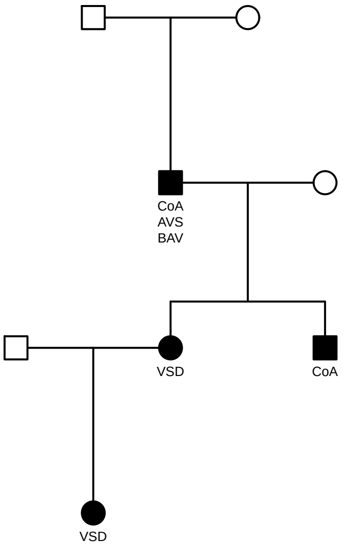

Postma_539

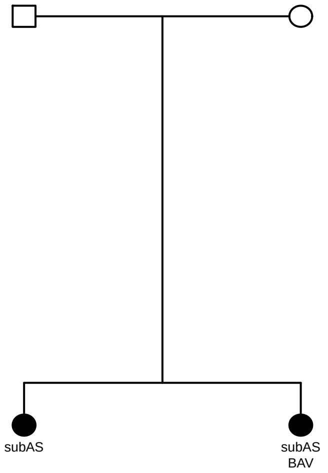

Postma_548

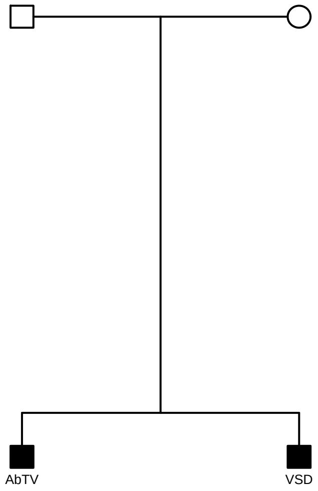

Postma_572

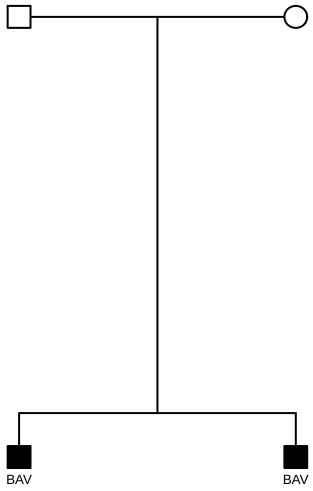

Postma_589

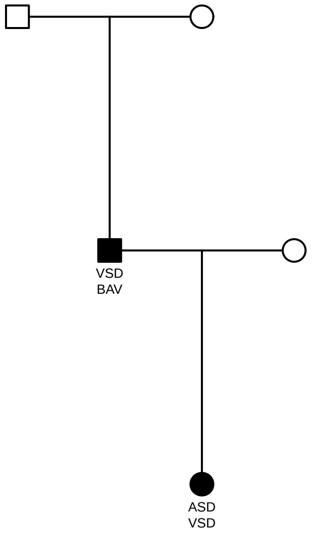

Postma_612

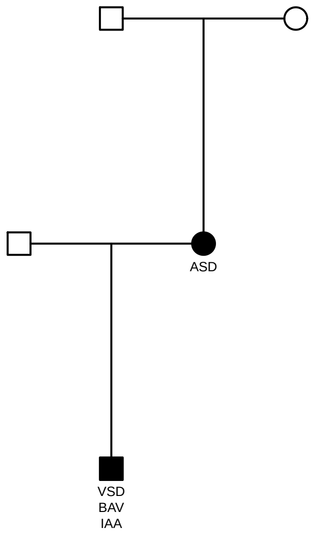

Postma_622

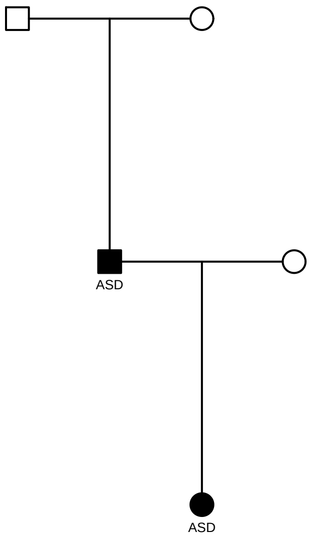

Postma_647

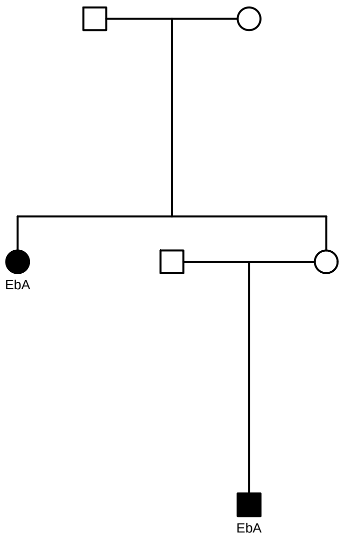

Postma_761

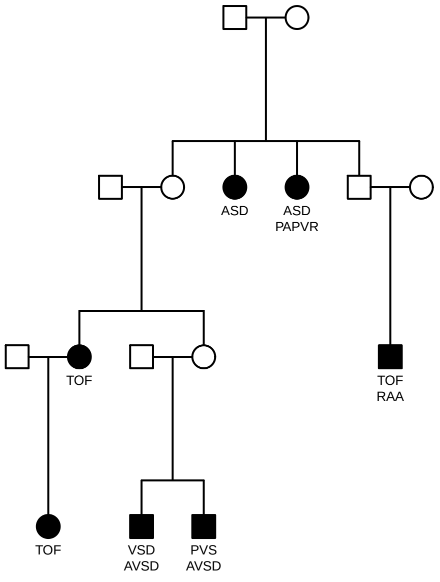

Postma_775

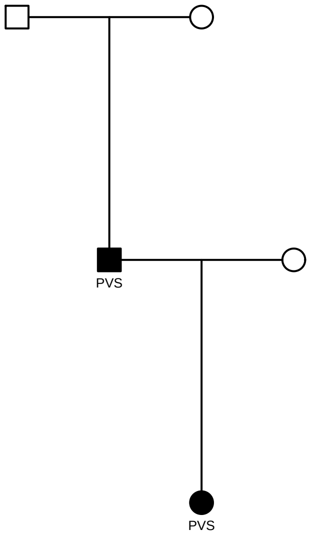

Postma_807

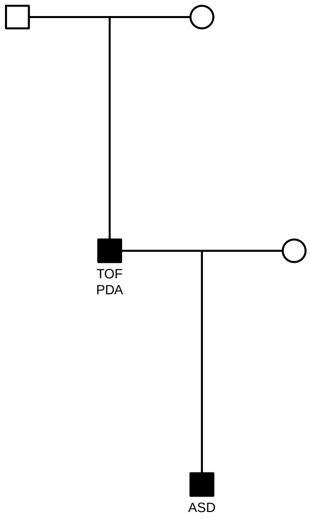

Postma_810

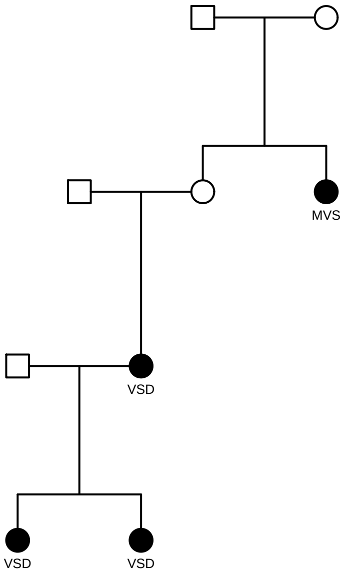

Postma_818

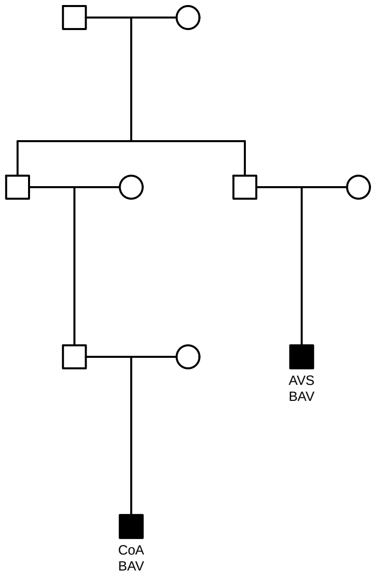

Postma_885

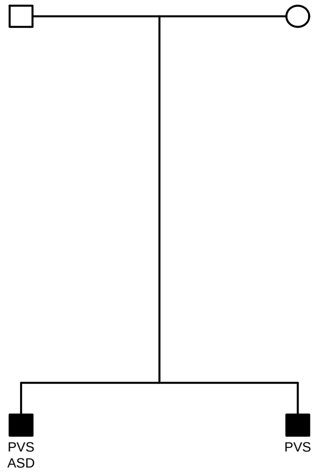

Postma_912

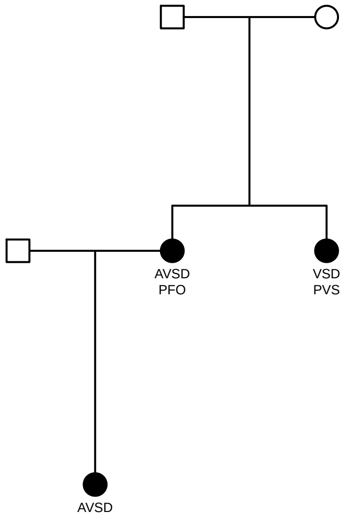

Postma_930

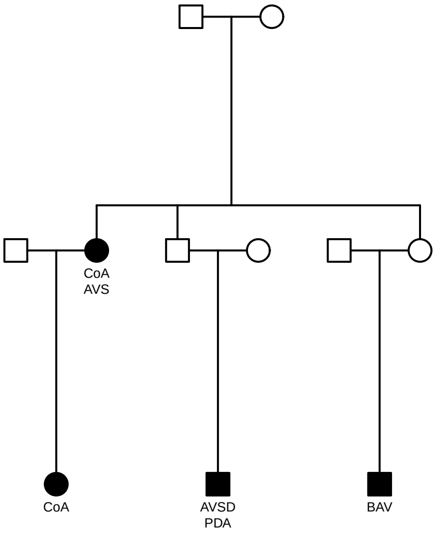

Postma_956

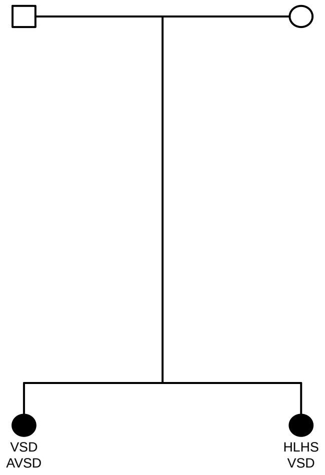

Postma_XXA

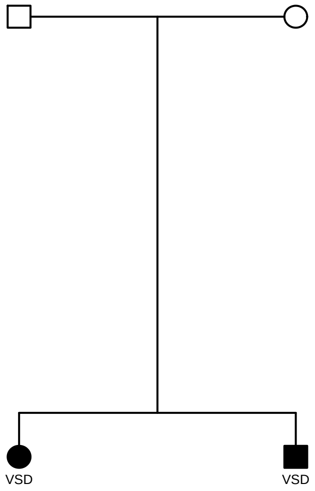

Postma_XXC

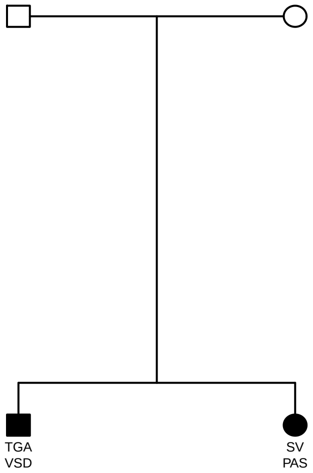

Postma_XXD

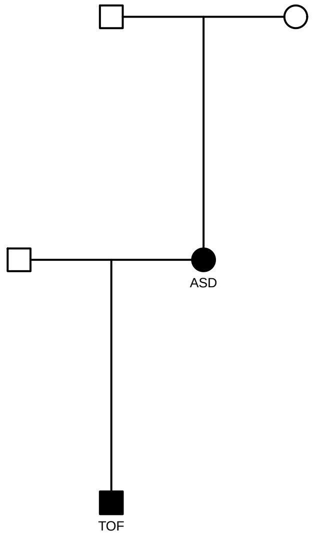

Postma_XXE

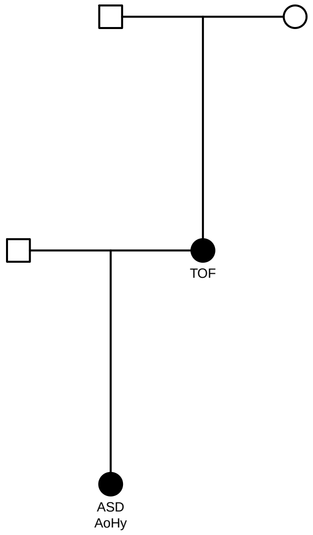

McBride_7

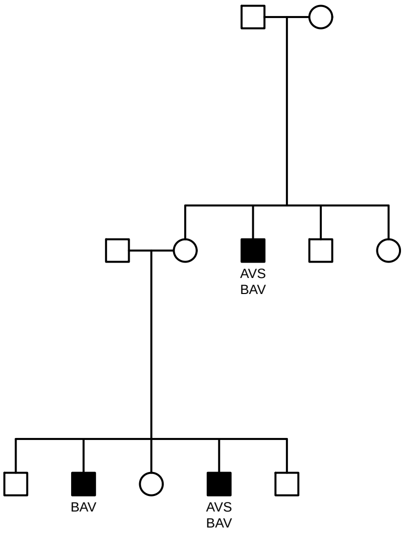

McBride_11

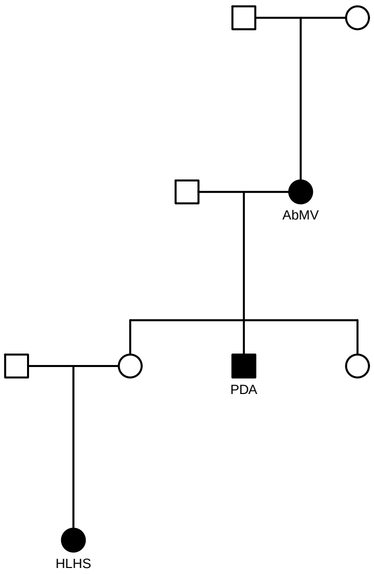

McBride_16

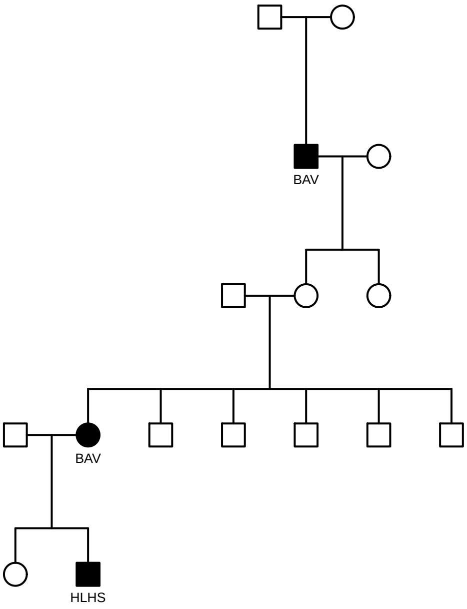

McBride_19

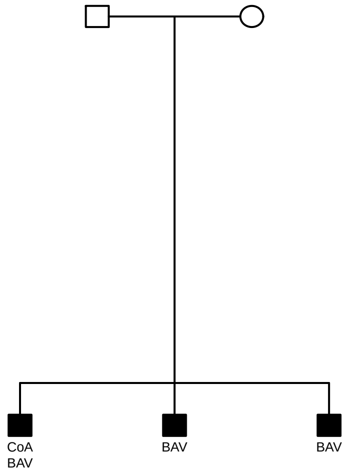

McBride_27

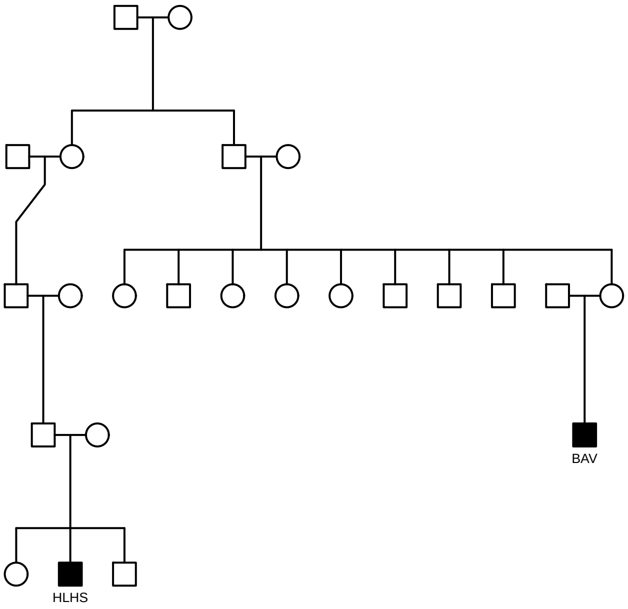

McBride_34

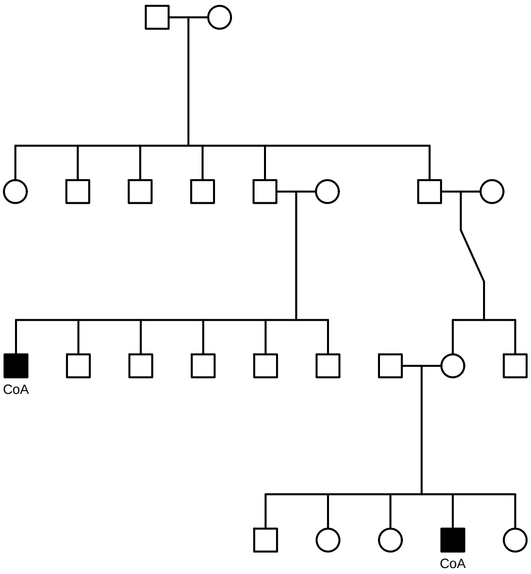

McBride_37

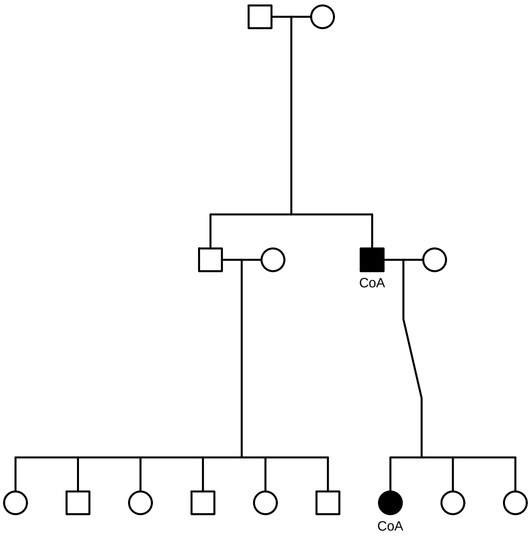

McBride_40

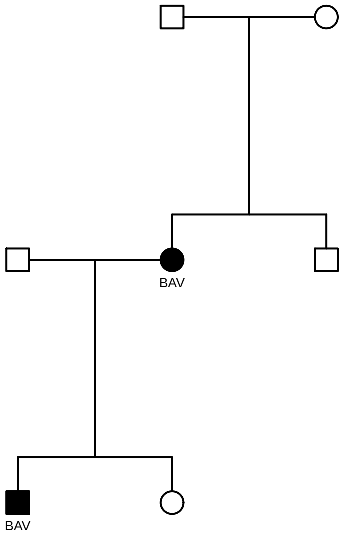

McBride_44

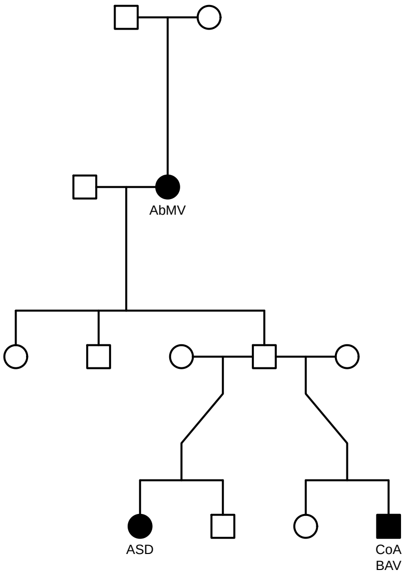

McBride_58

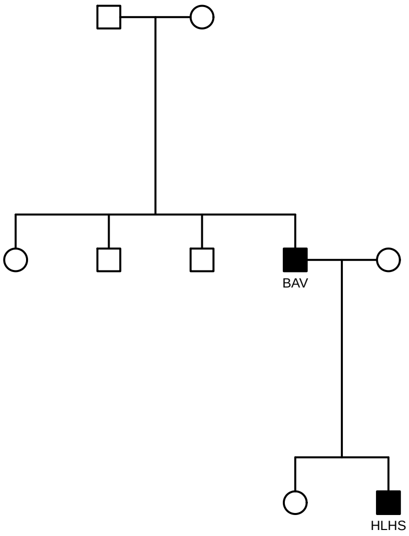

McBride_72

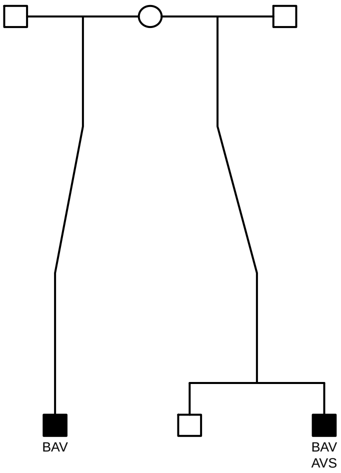

McBride_74

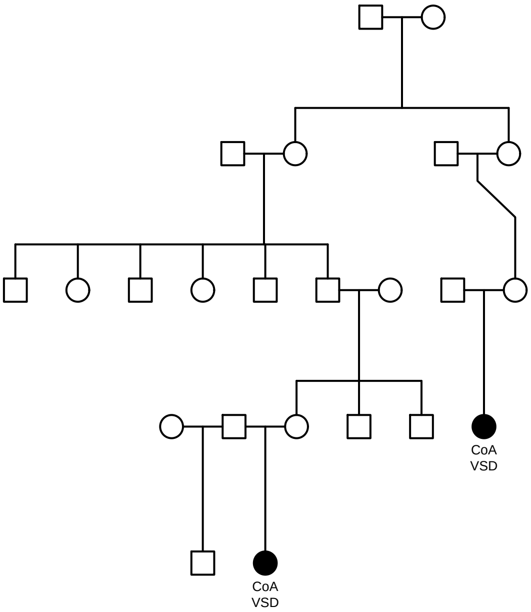

McBride_91

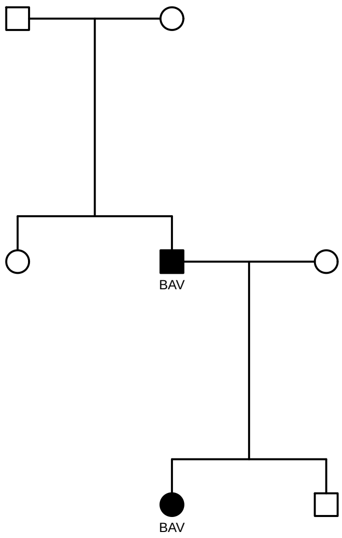

McBride_93

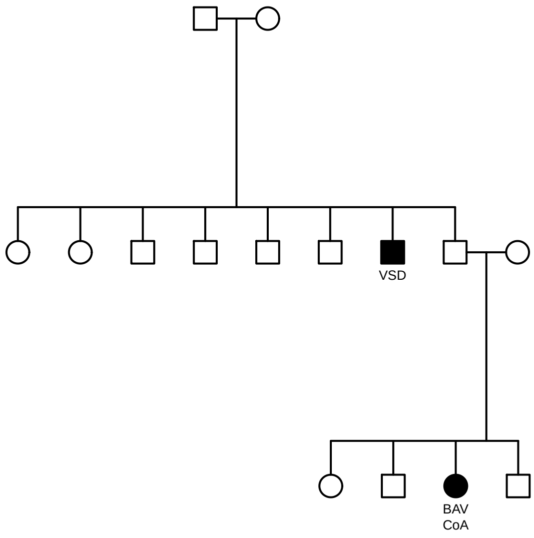

McBride_105

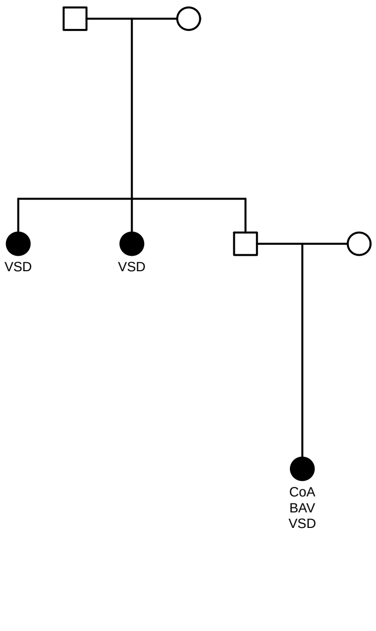

McBride_110

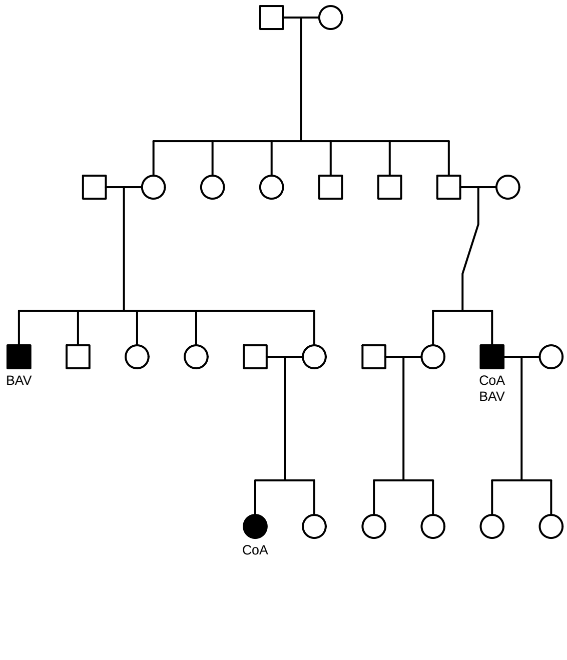

McBride_118

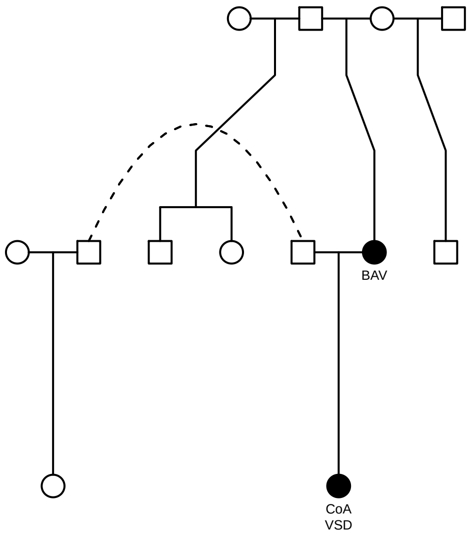

McBride_123

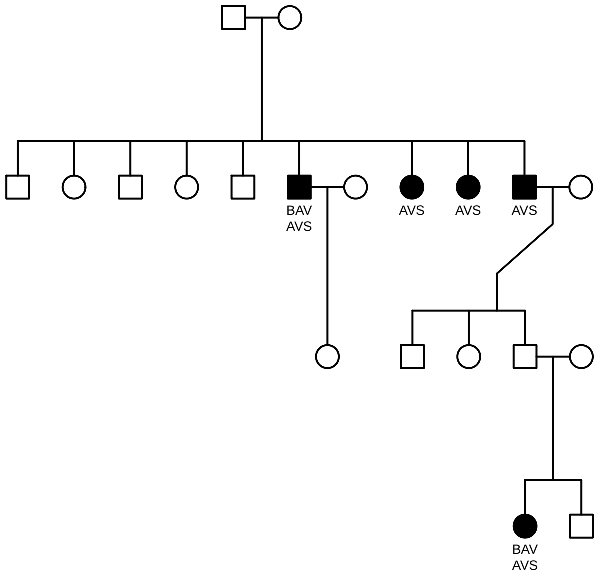

McBride_125

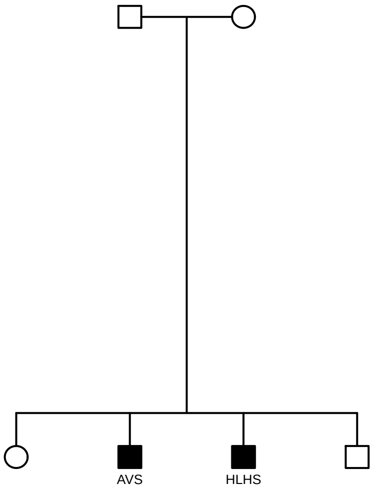

McBride_135

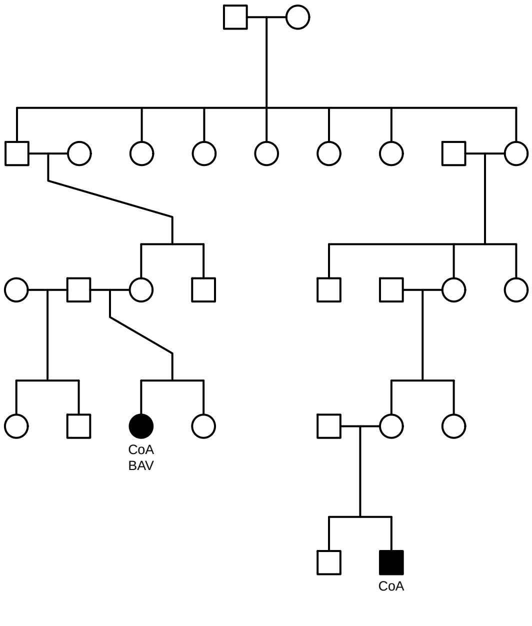

McBride_139

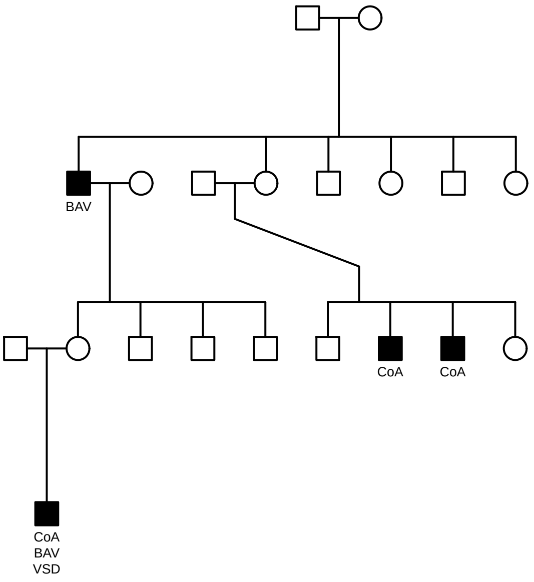

McBride_149

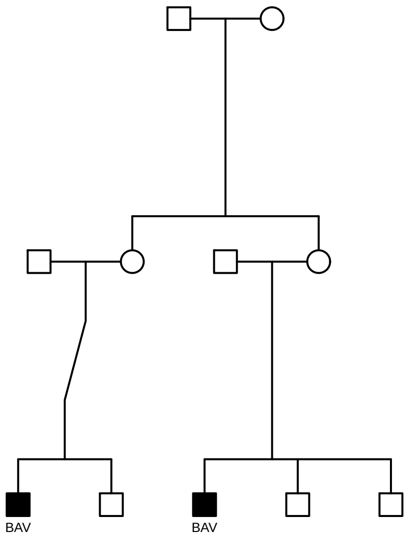

McBride_154

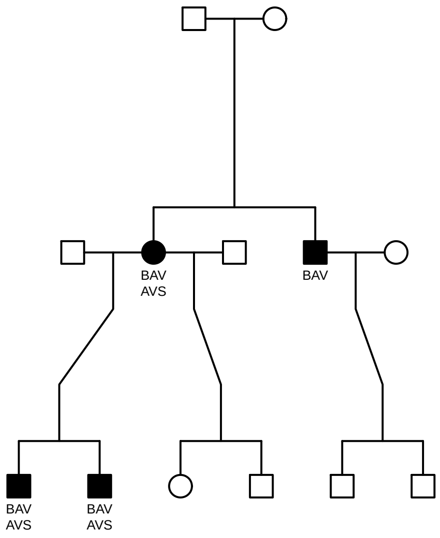

McBride_155

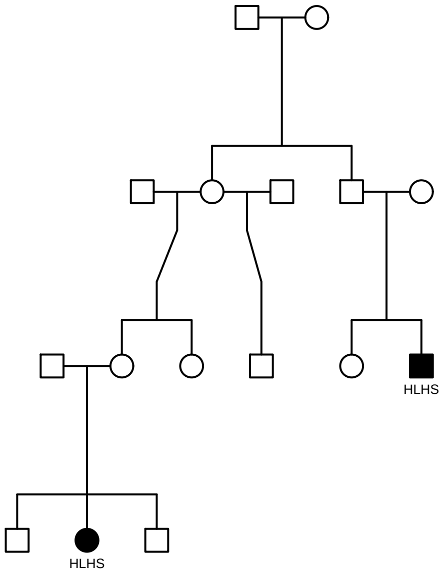

McBride_156_b

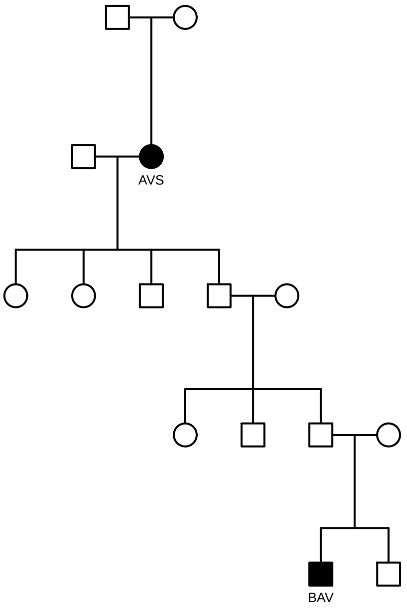

McBride_157

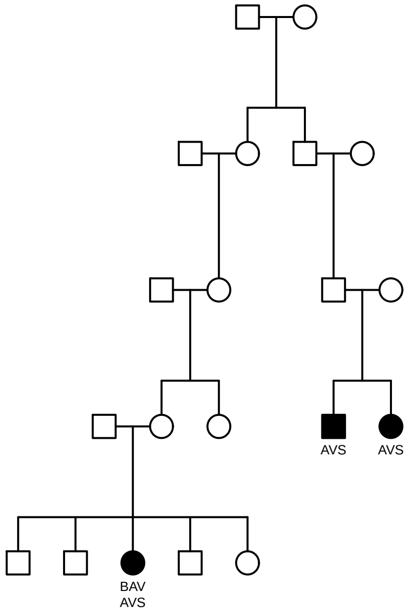

McBride_159

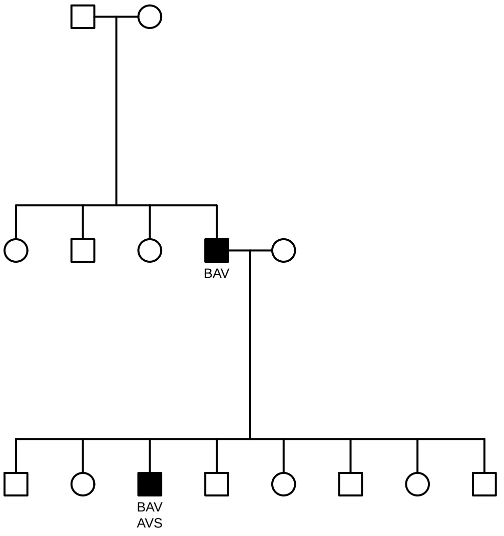

McBride_160

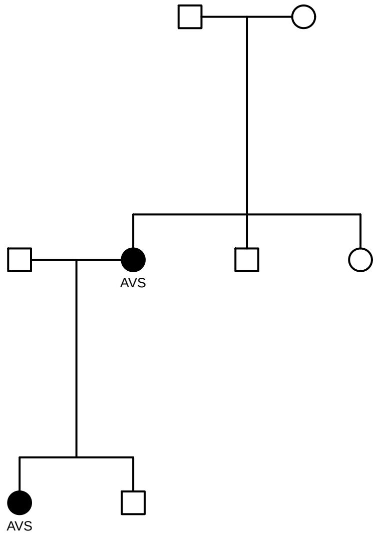

McBride_164

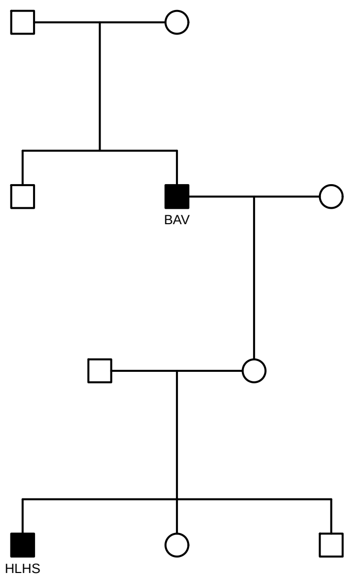

McBride_183

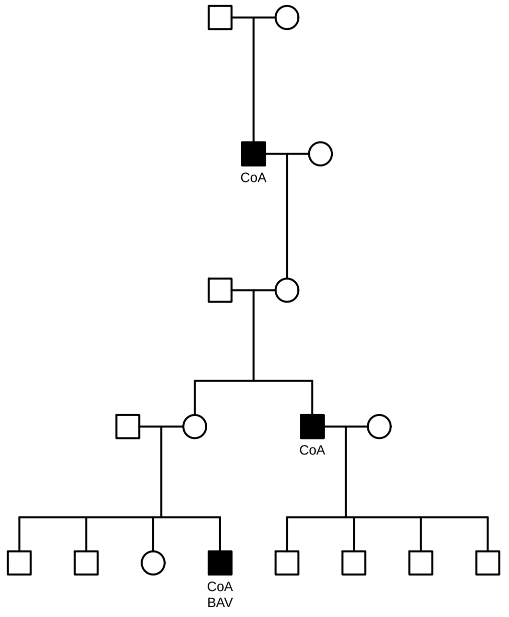

McBride_190

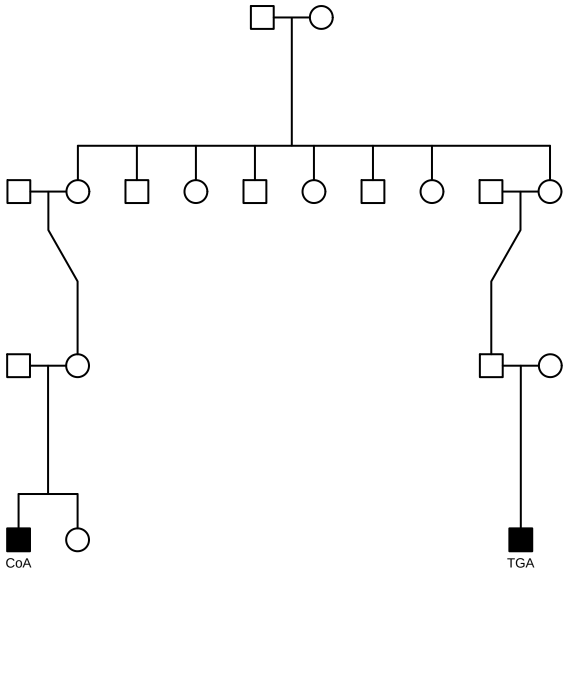

McBride_195

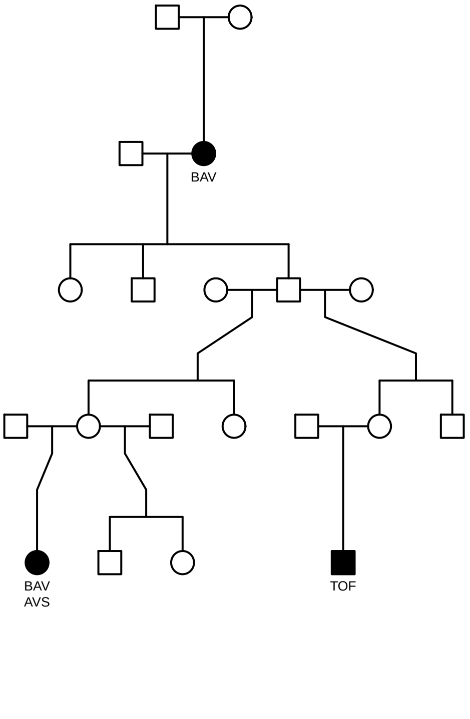

McBride_207

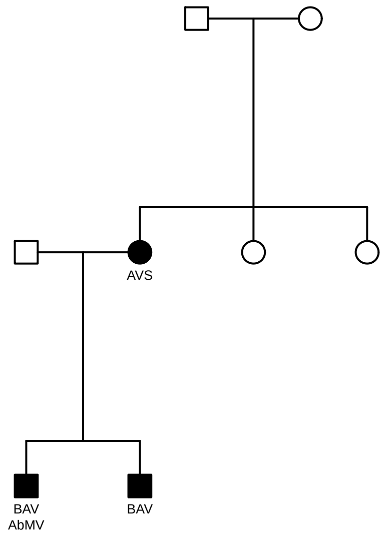

McBride_210

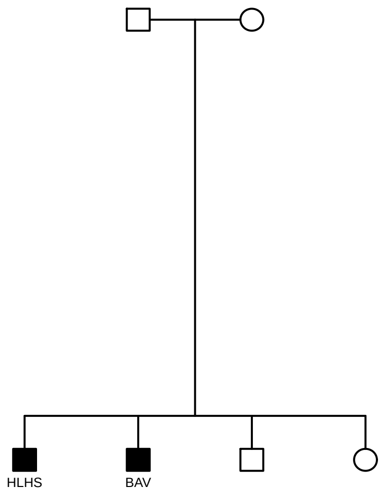

McBride_237

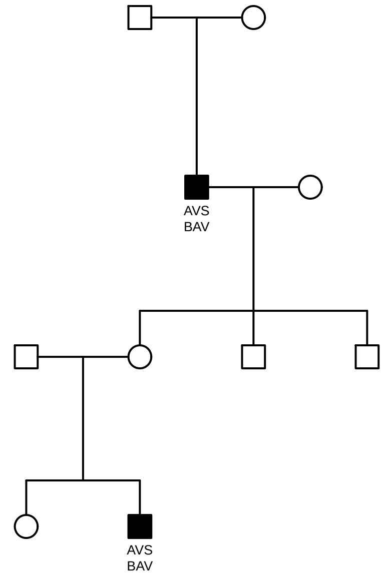

McBride_247

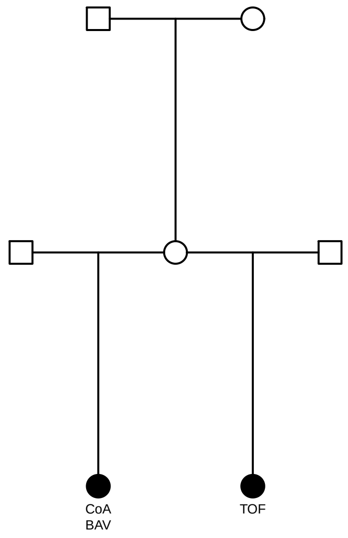

McBride_254

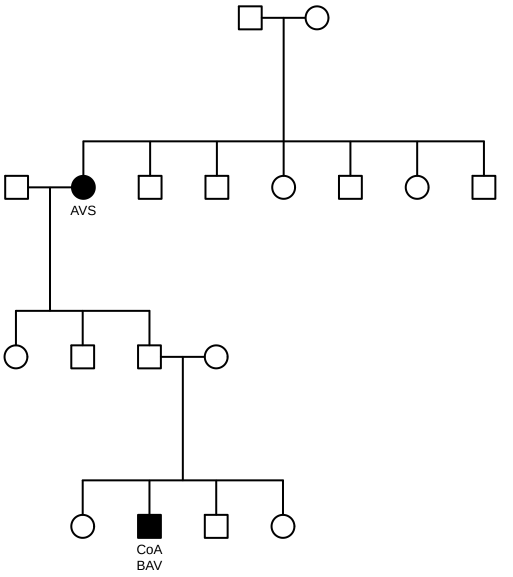

McBride_275

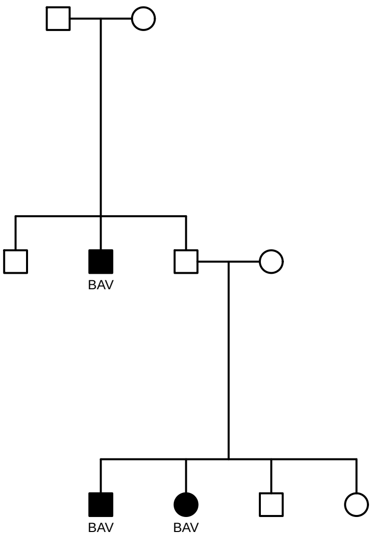

McBride_278

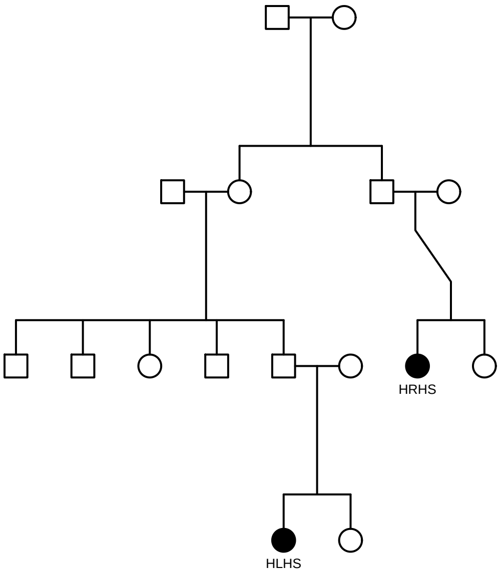

McBride_286

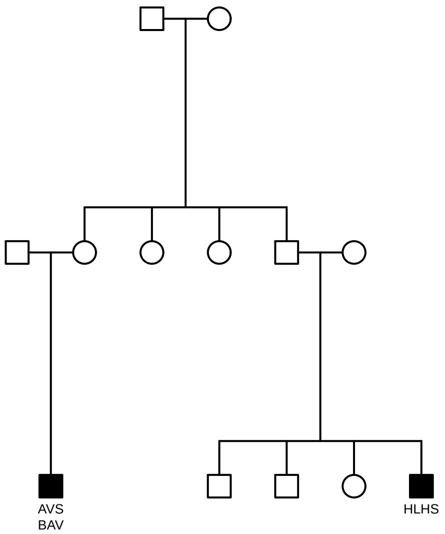

McBride_289

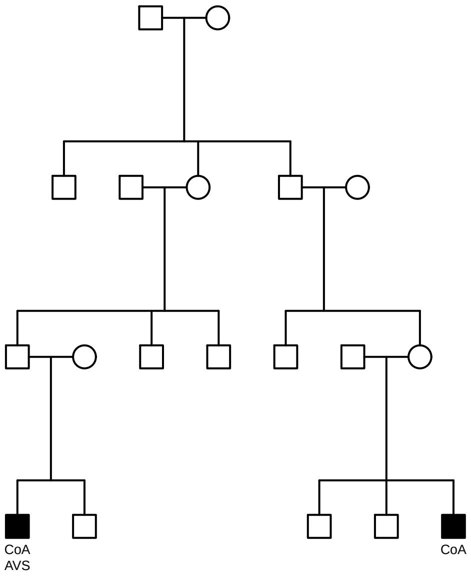

McBride_290

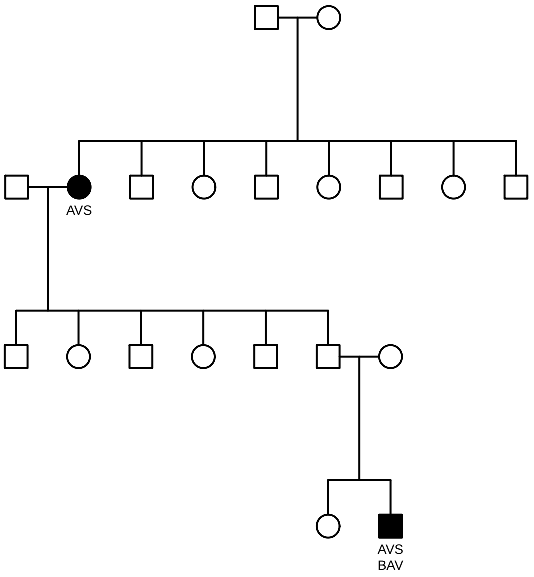

McBride_368

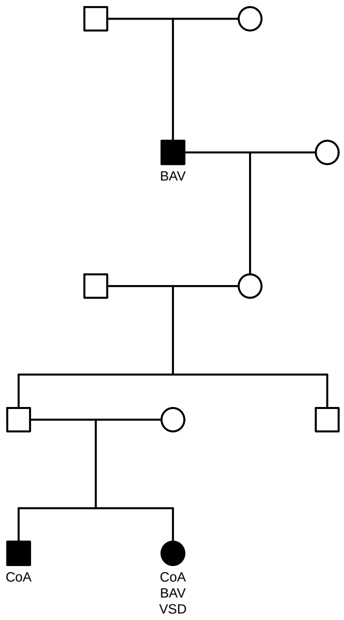

McBride_379

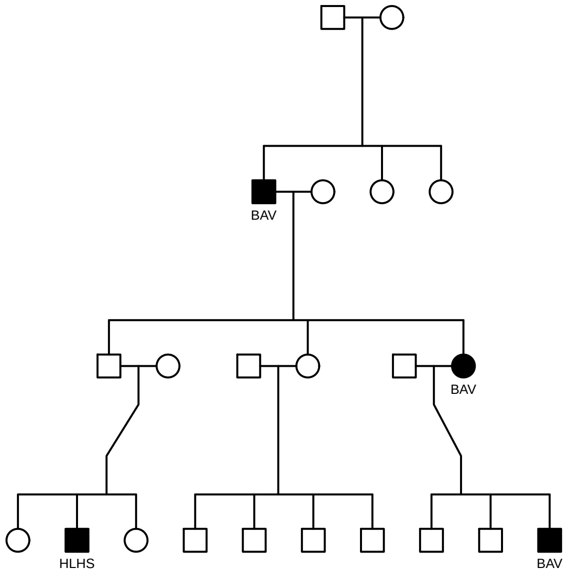

McBride_380

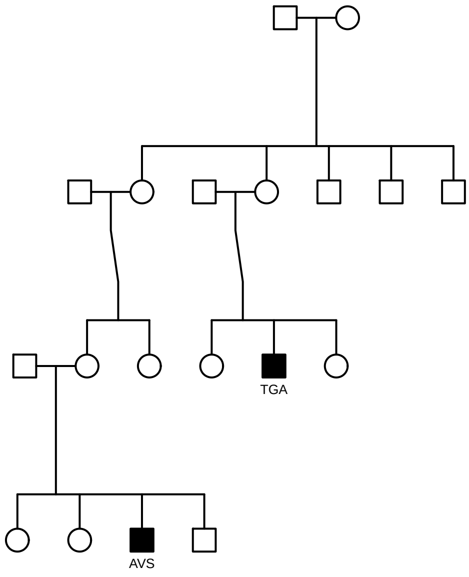

McBride_381

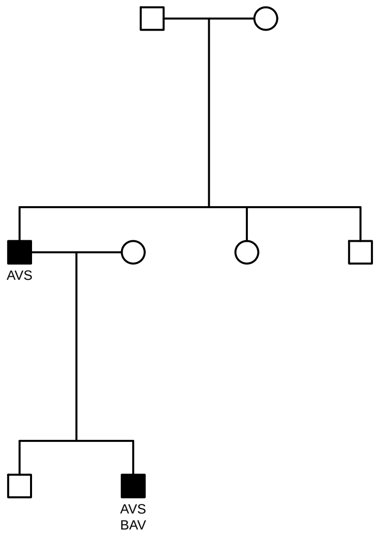

McBride_382

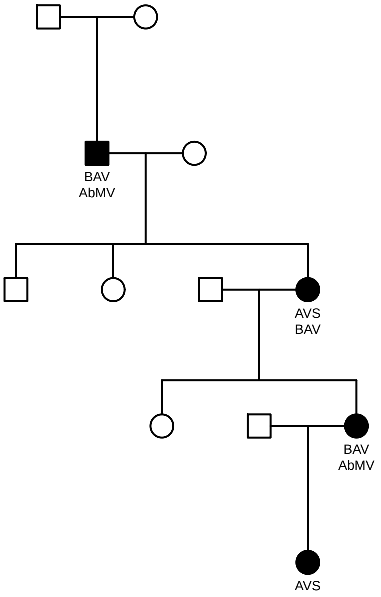

McBride_393

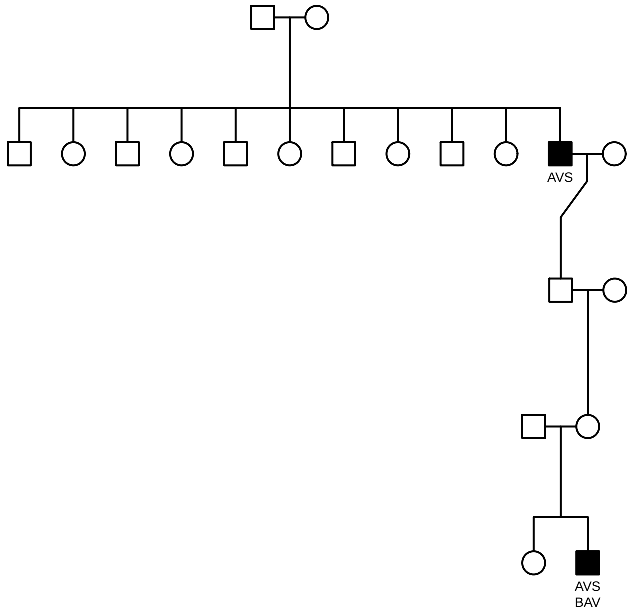

McBride_400

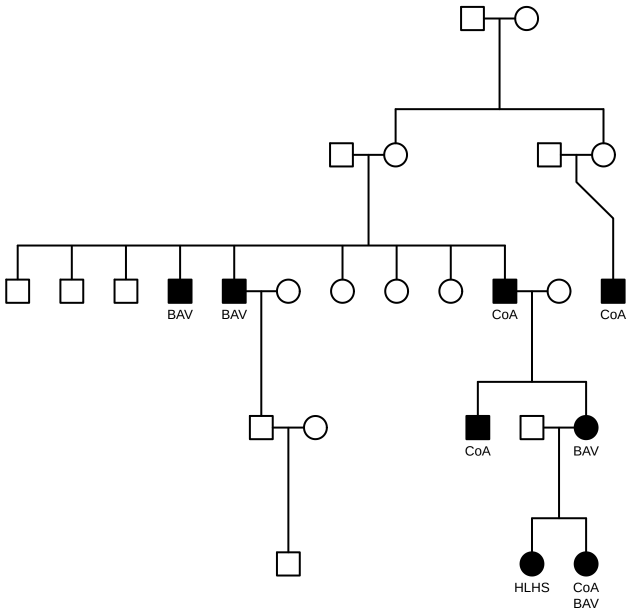

McBride_411

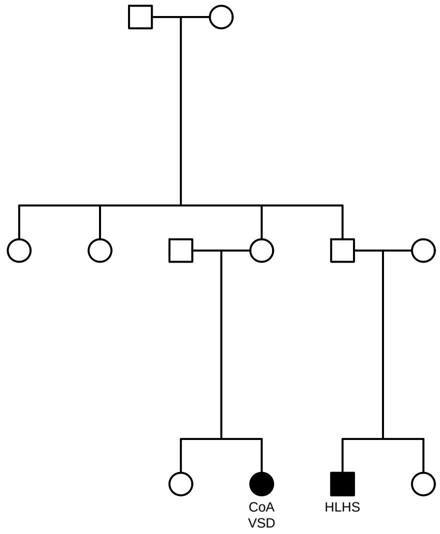

McBride_412

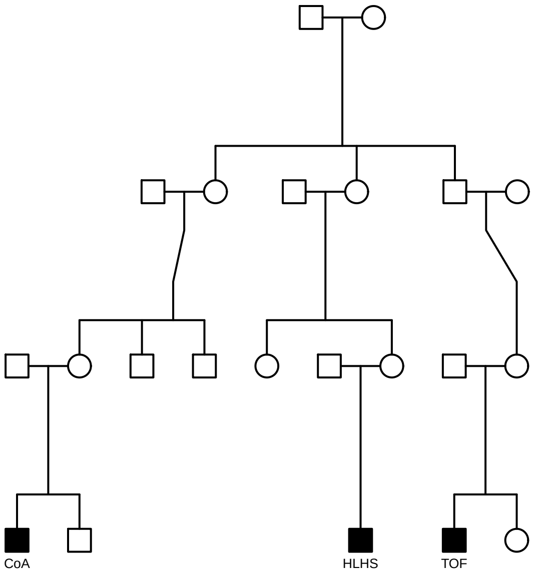

McBride_439

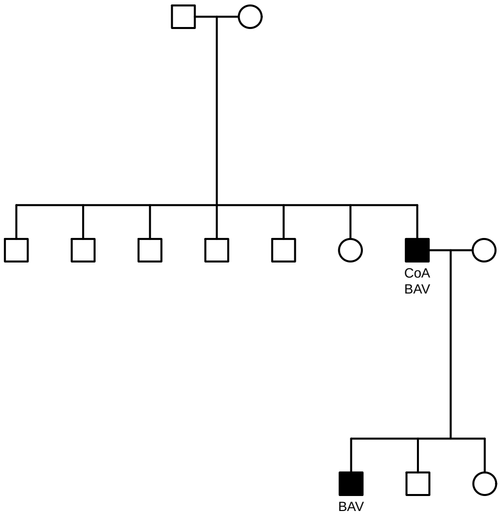

McBride_442

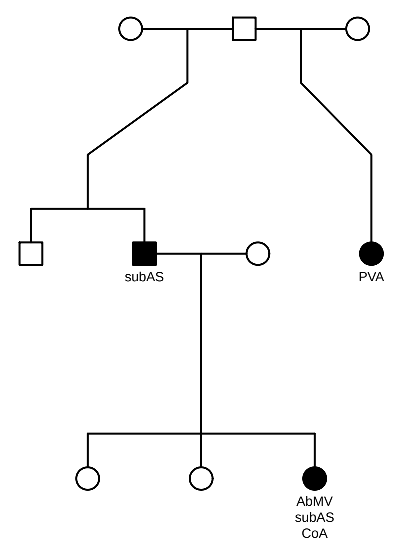

McBride_450

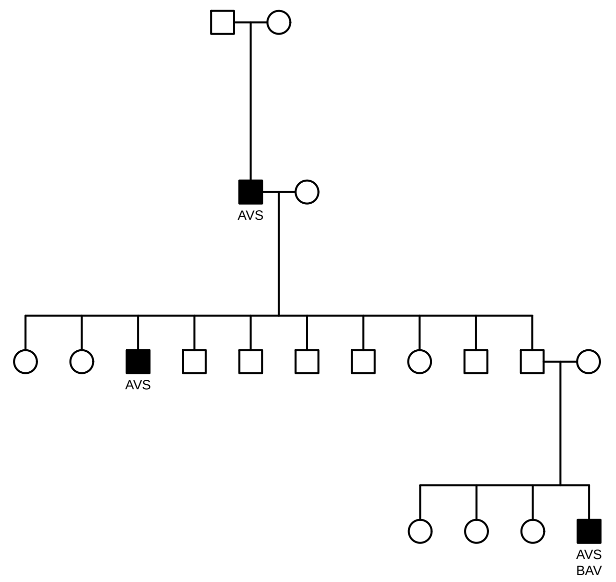

McBride_456

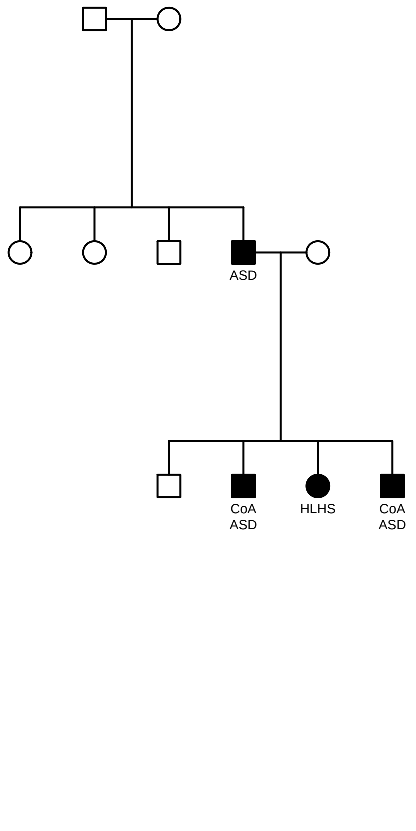

McBride_469

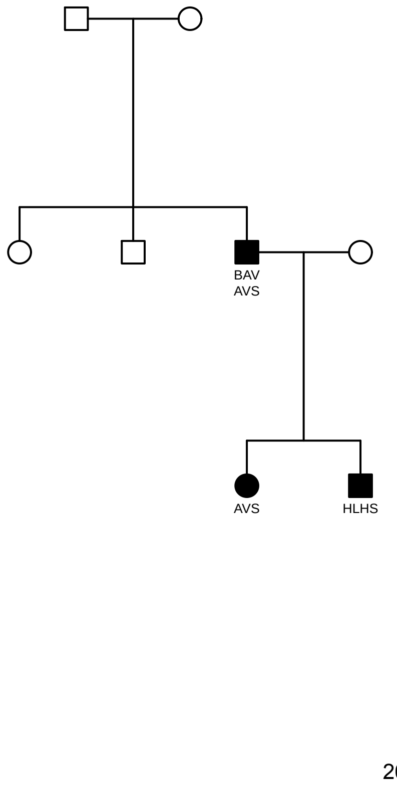

McBride_473

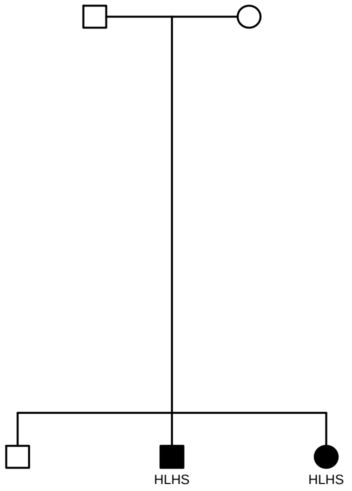

McBride_481

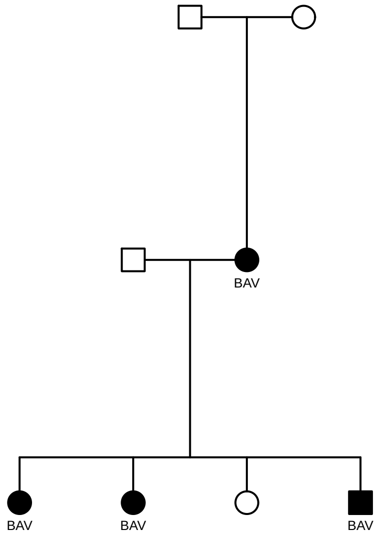

McBride_485

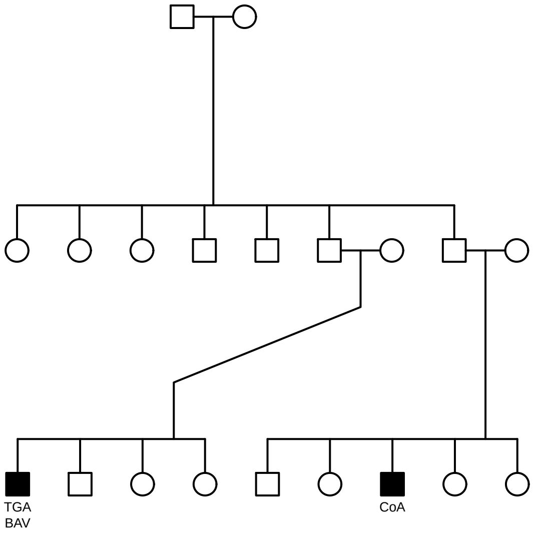

Ellesoe_3148

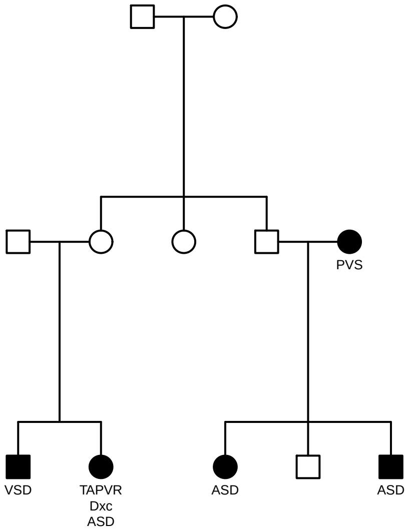

Loffredo_102

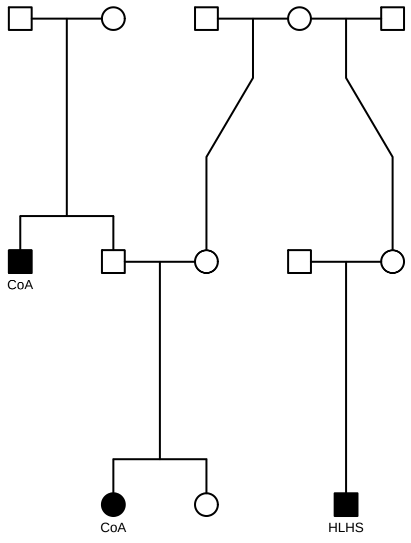

Loffredo_126

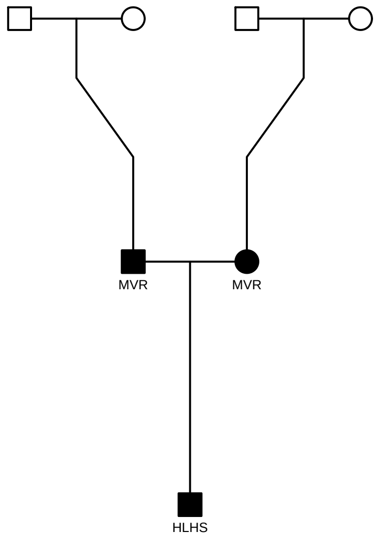

Loffredo_136

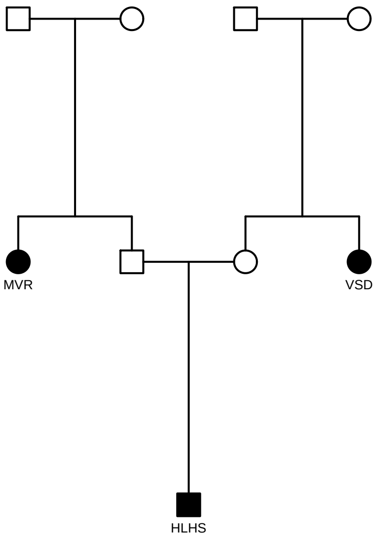

Loffredo_301

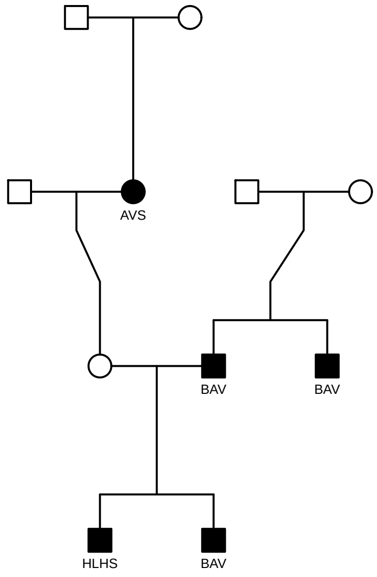

Loffredo_407

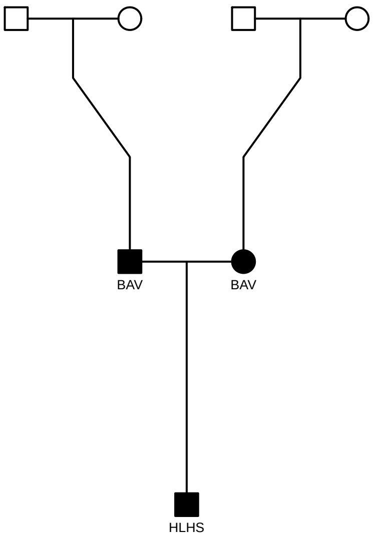

Supplemental Figure 3. Pedigrees of 526 previously published CHD families. The paper in which the family was described can be identified by a PubMed database (www.pubmed.gov) identification number (PMID). A list of complete references of these papers is shown in Supplemental Table 1.

McBride_156_a PMID:19142209

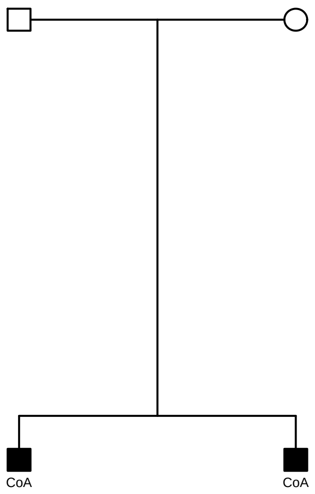

McBride_1568 PMID:19142209

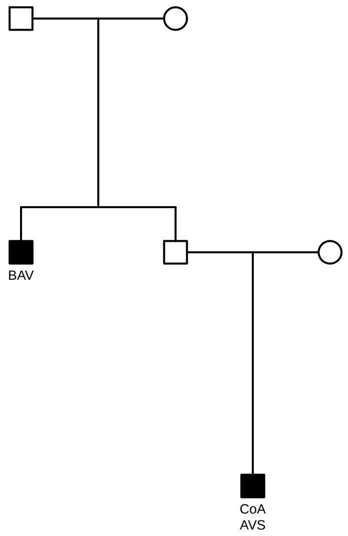

McBride_1574 PMID:19142209

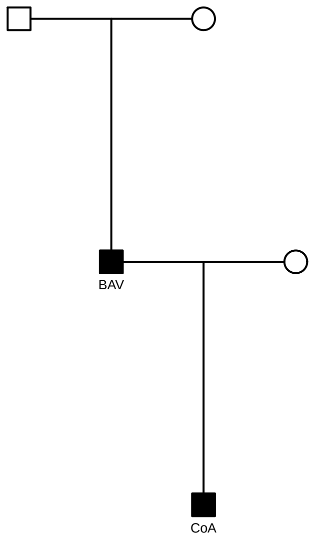

McBride_1646 PMID:19142209

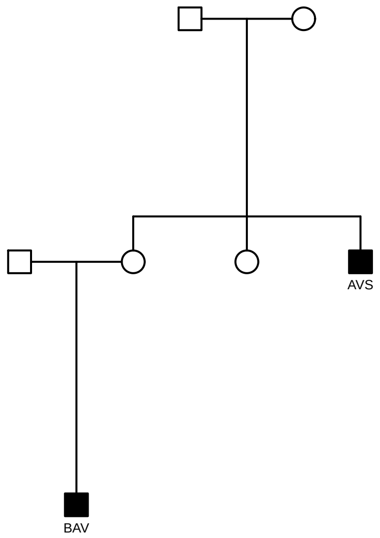

McBride_1685 PMID:19142209

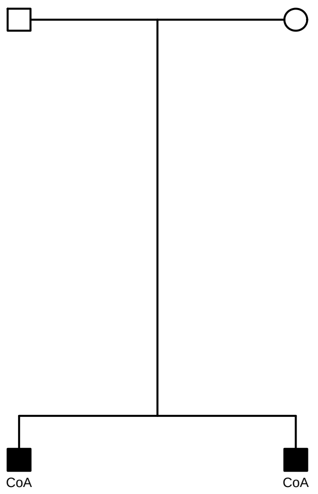

McBride_1705 PMID:19142209

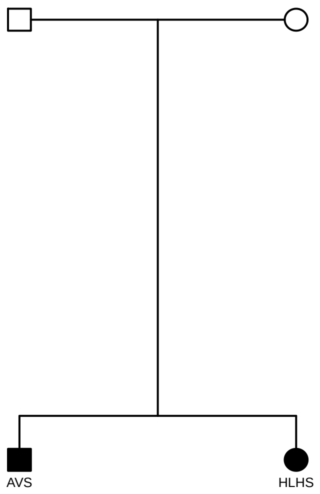

McBride_90 PMID:19142209

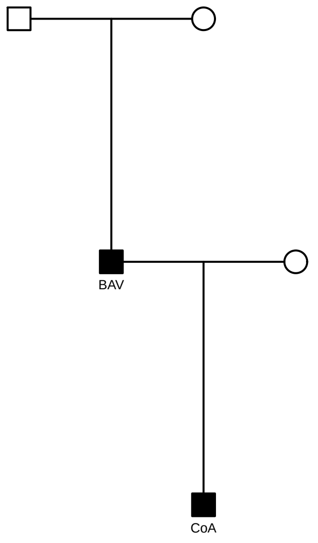

McBride_219 PMID:19142209

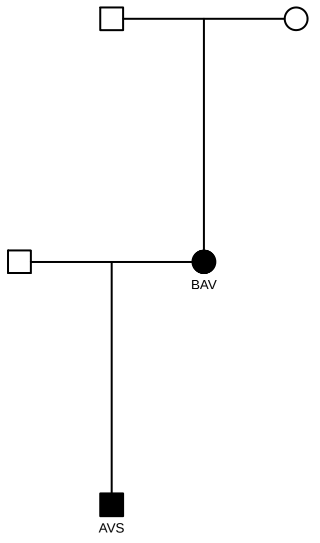

McBride_234 PMID:19142209

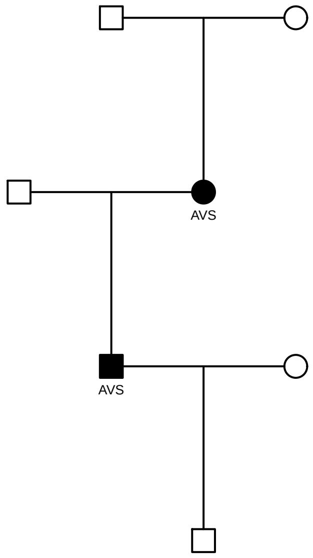

McBride_238 PMID:19142209

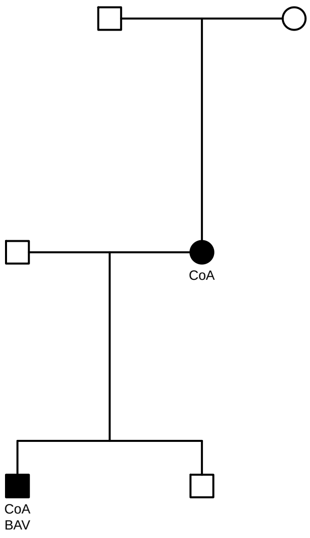

McBride_242 PMID:19142209

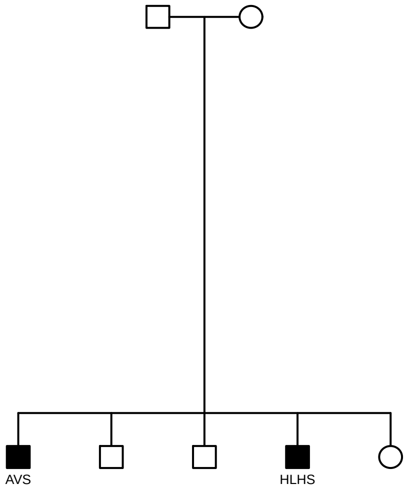

McBride_258 PMID:19142209

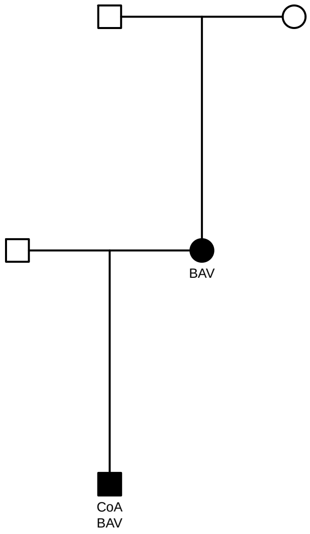

McBride_974 PMID:19142209

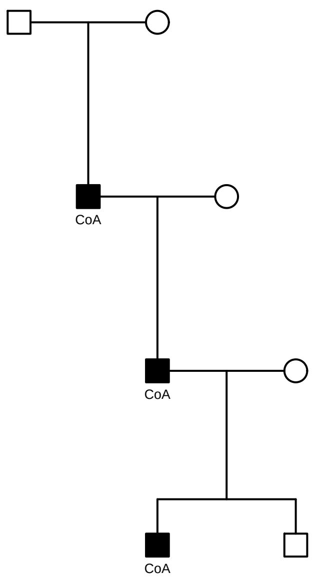

McBride_80 PMID:19142209

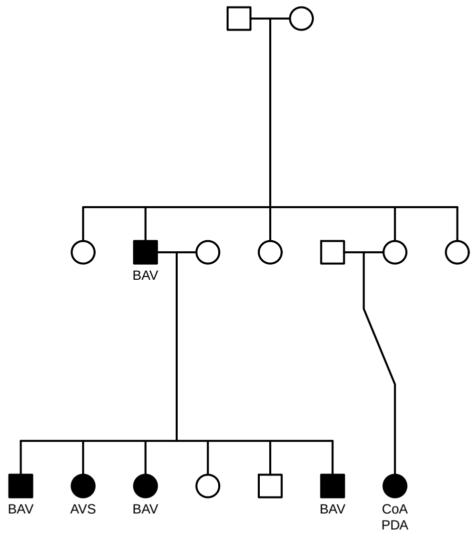

McBride_648 PMID:19142209

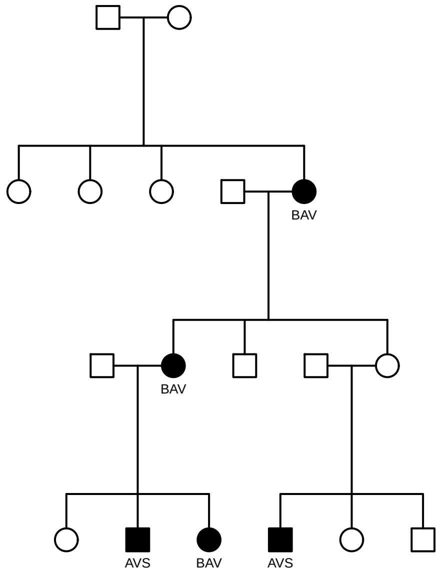

McBride_268 PMID:19142209

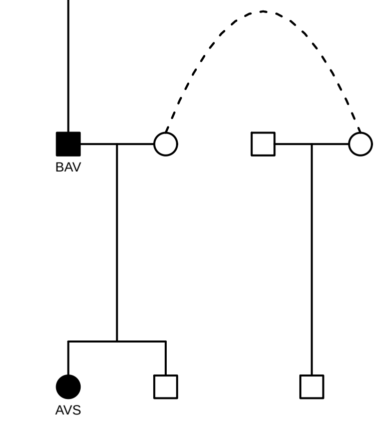

McBride_304 PMID:19142209

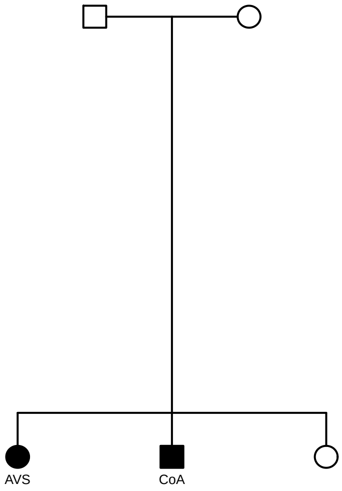

McBride_321 PMID:19142209

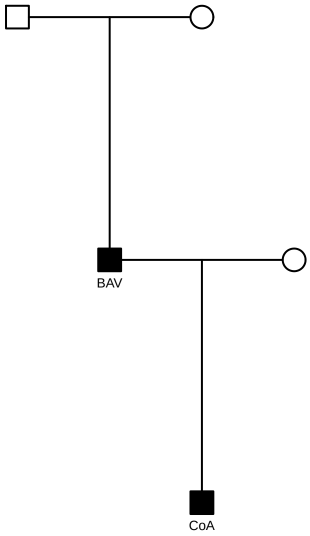

McBride_324 PMID:19142209

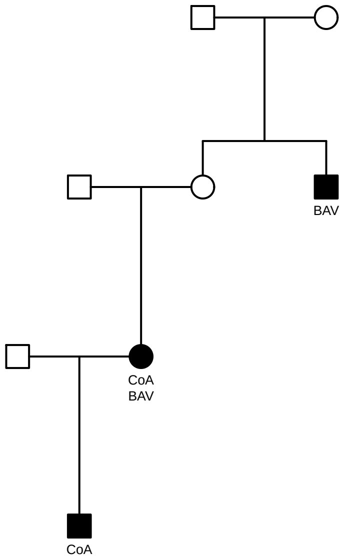

McBride_329 PMID:19142209

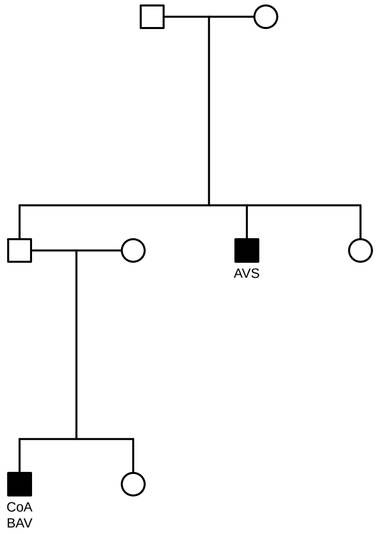

McBride_341 PMID:19142209

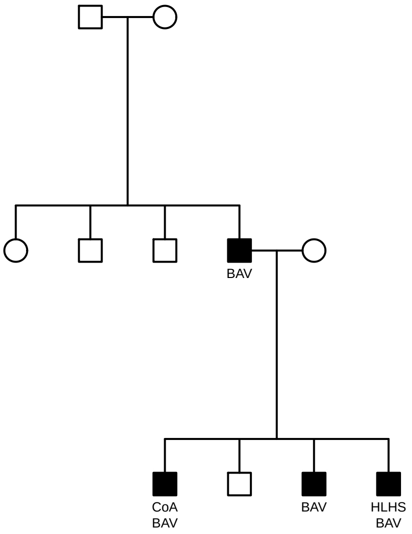

McBride_344 PMID:19142209

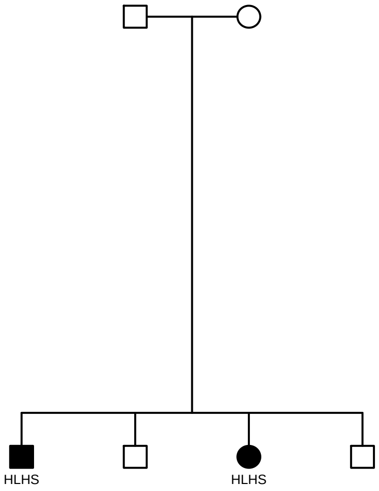

McBride_471 PMID:19142209

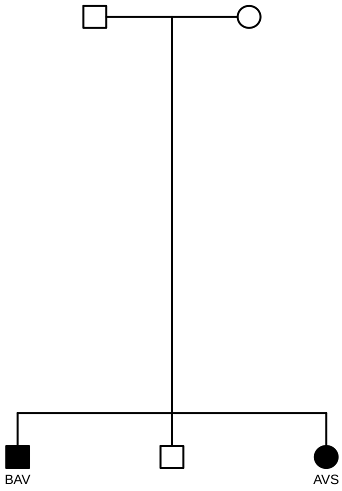

McBride_523 PMID:19142209

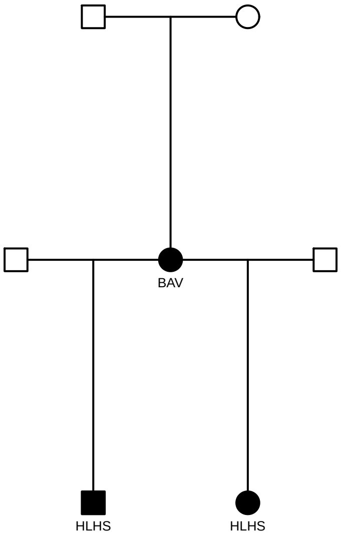

McBride_546 PMID:19142209

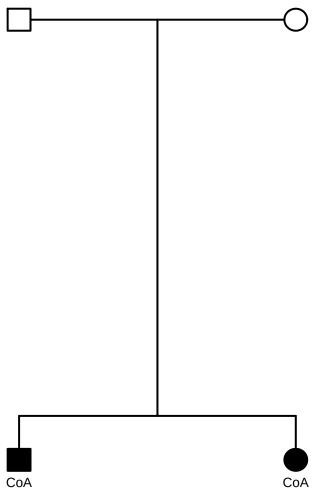

McBride_55007 PMID:19142209

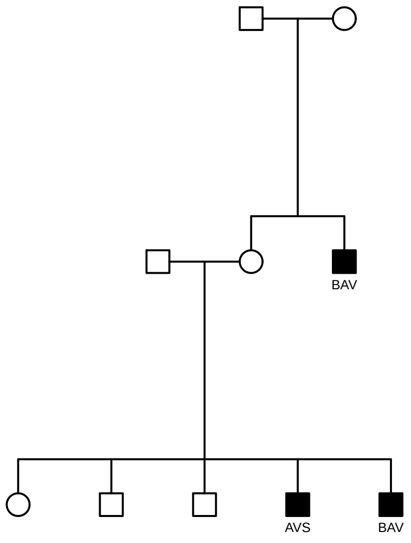

McBride_564 PMID:19142209

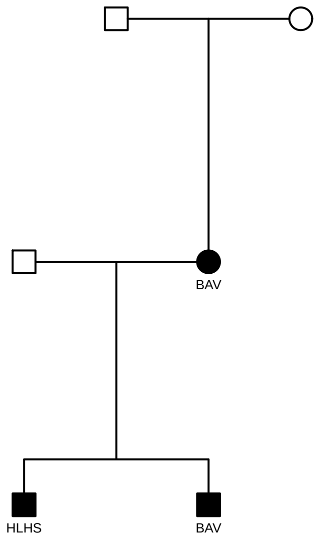

McBride_614 PMID:19142209

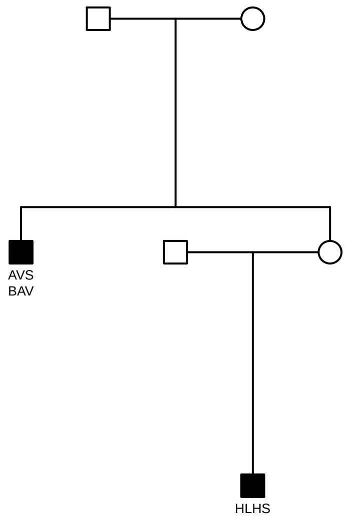

McBride_630 PMID:19142209

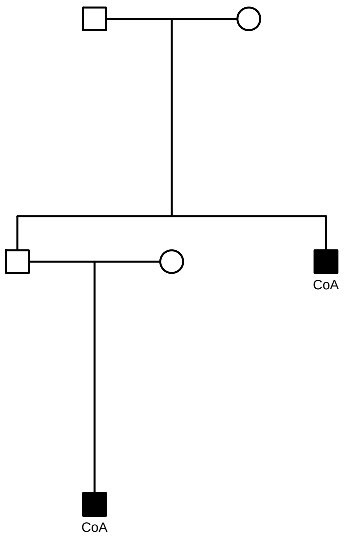

McBride_1022 PMID:19142209

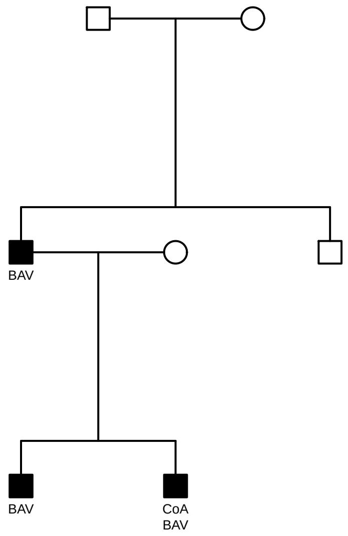

McBride_1154 PMID:19142209

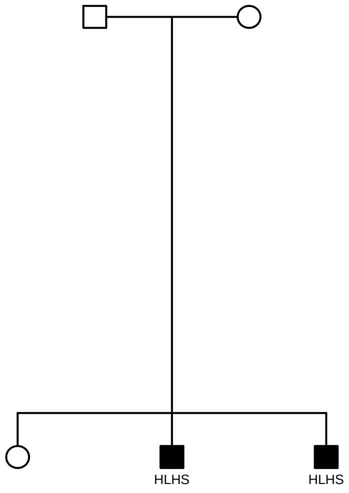

McBride_1206 PMID:19142209

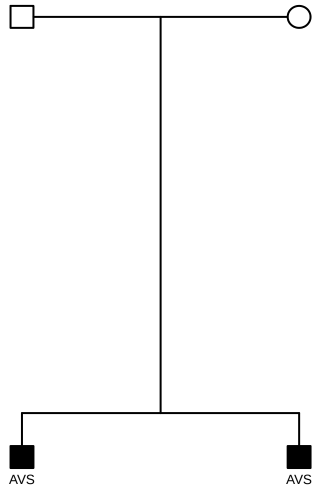

McBride_1254 PMID:19142209

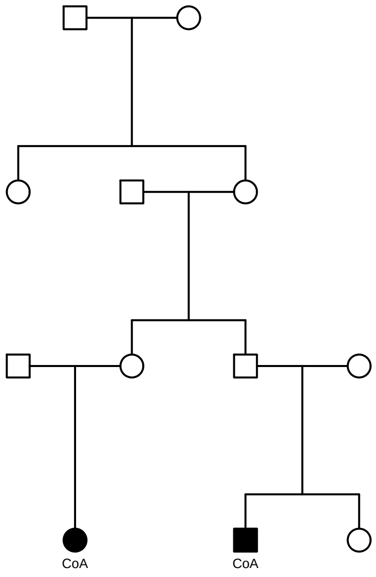

McBride_1298 PMID:19142209

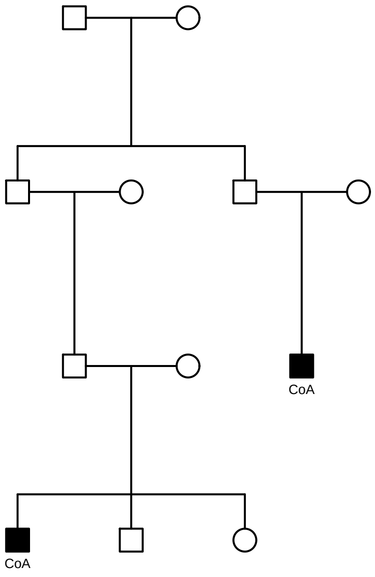

McBride_977 PMID:19142209

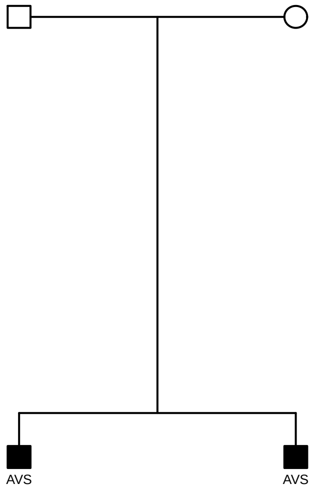

McBride_134 PMID:19142209

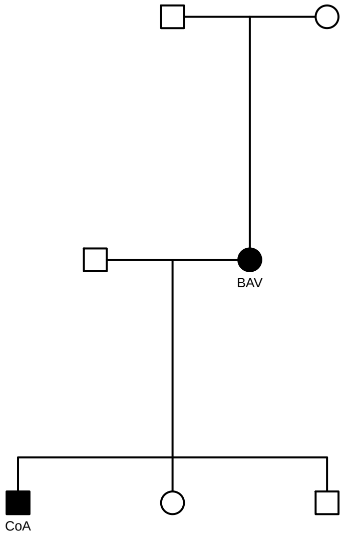

McBride_137 PMID:19142209

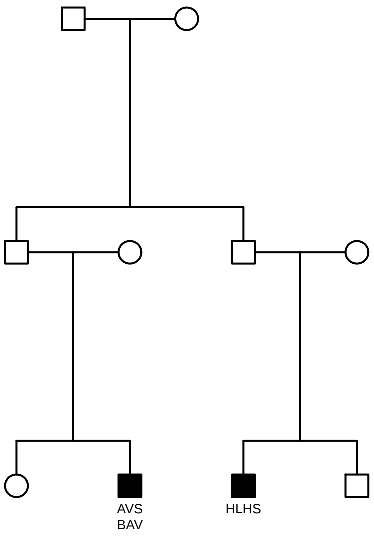

McBride_1374 PMID:19142209

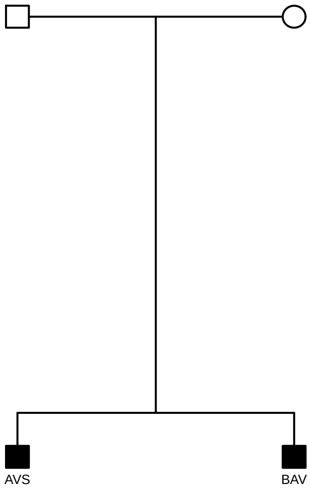

McBride_1426 PMID:19142209

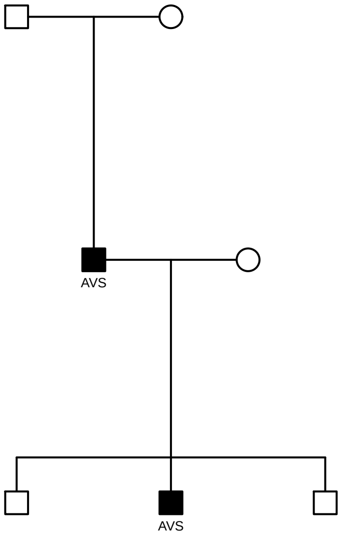

McBride_1541 PMID:19142209

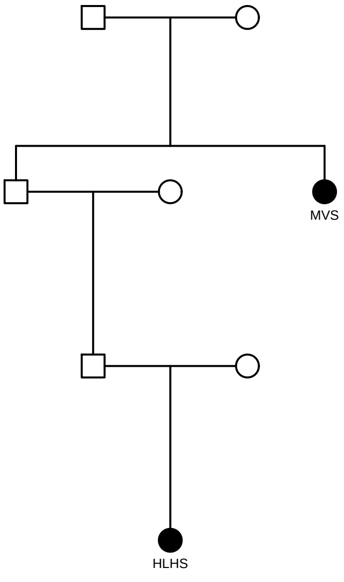

Gale_1977 PMID:913155

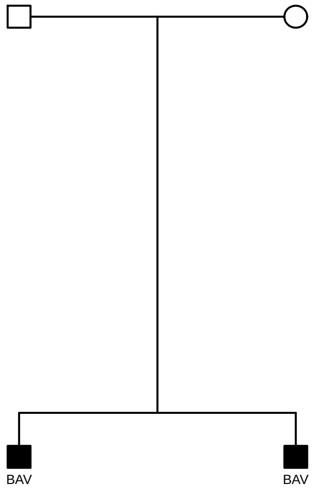

Glick_II PMID:8109558

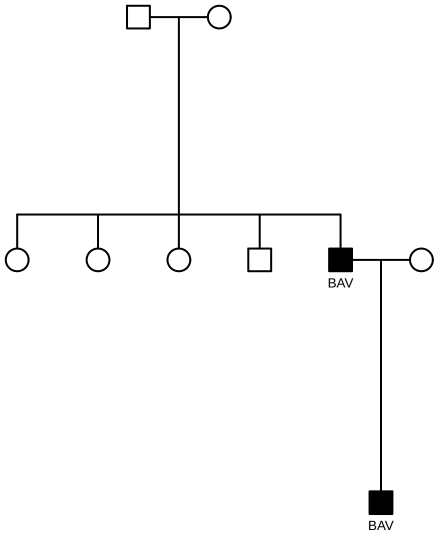

Glick_III PMID:8109558

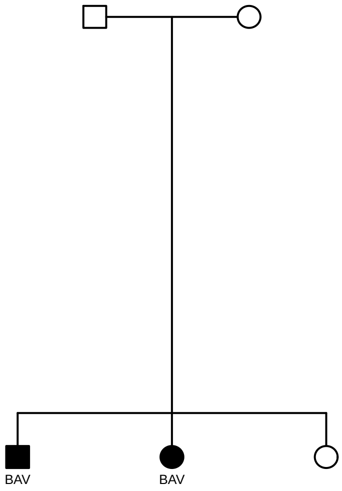

Glick_IV PMID:8109558

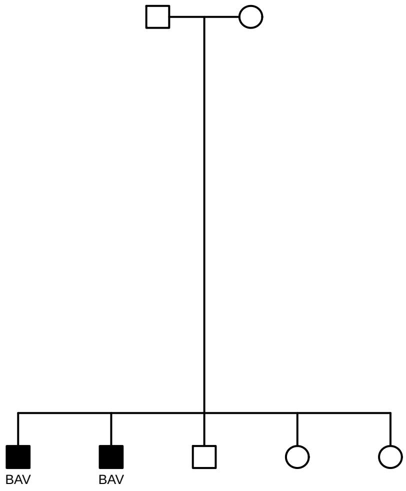

Glick_V PMID:8109558

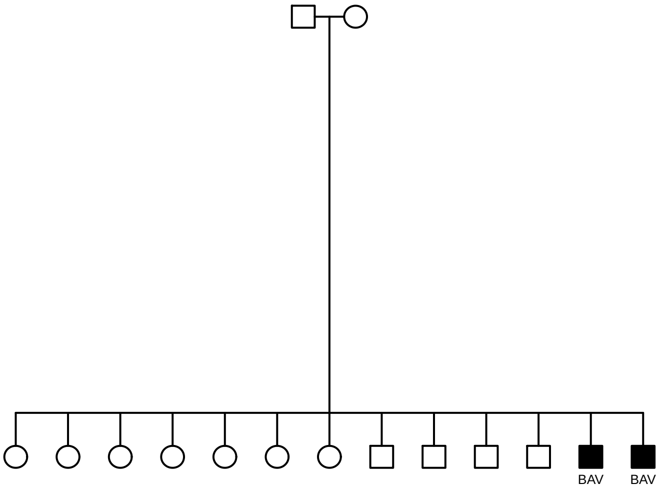

Glick_VI PMID:8109558

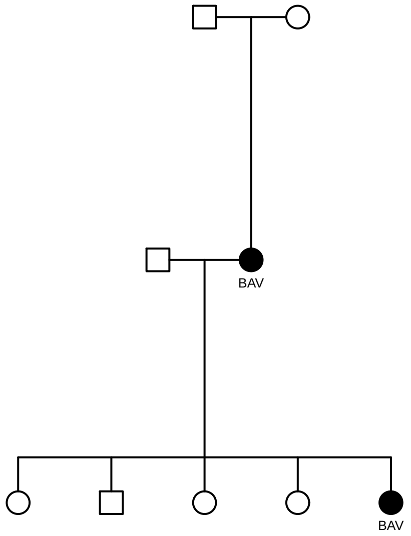

Schonfeld_1958 PMID:13640457

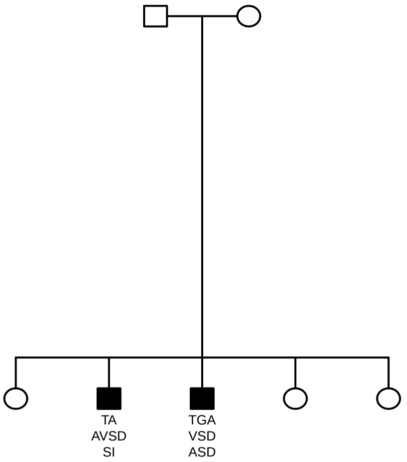

Seides_1979 PMID:575295

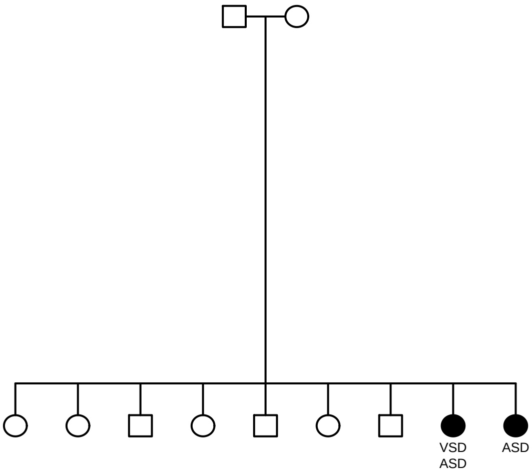

Kodo_2009 PMID:19666519

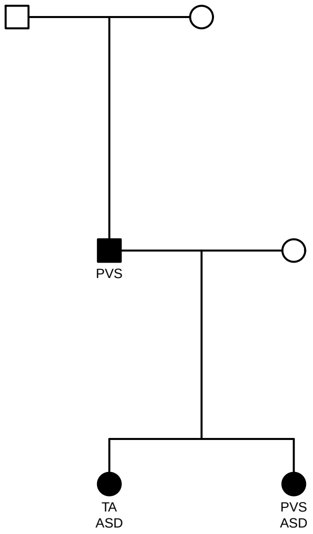

Wang_1 PMID:23021226

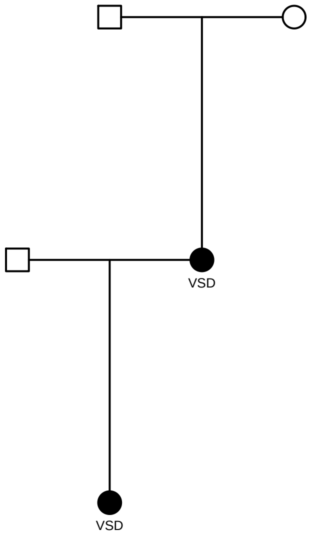

Wang_2 PMID:23021226

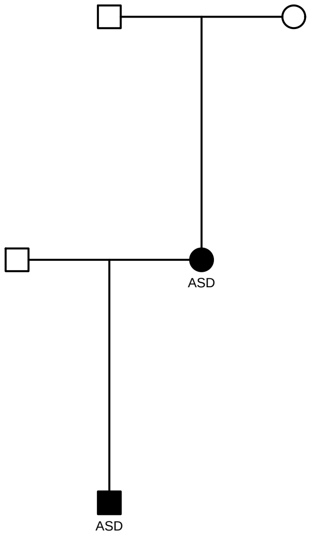

Wang_3 PMID:23021226

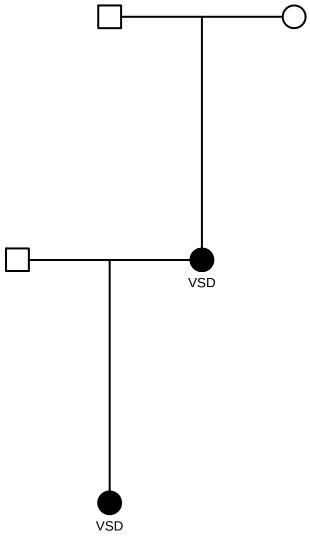

Wang_4 PMID:23021226

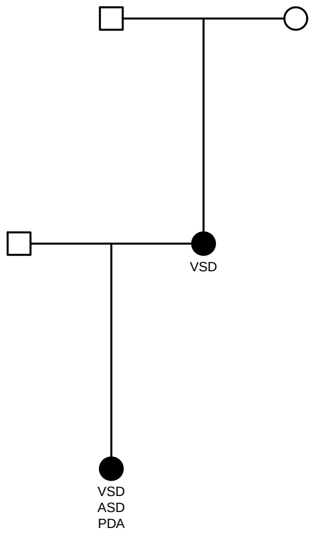

Wang_5 PMID:23021226

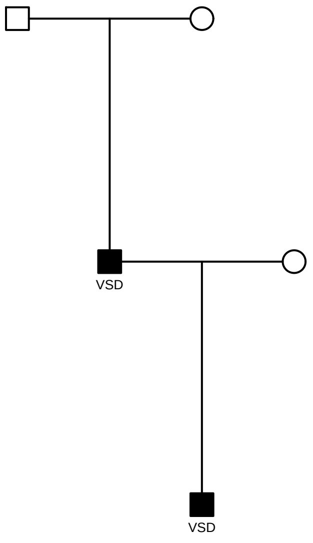

Wang_6 PMID:23021226

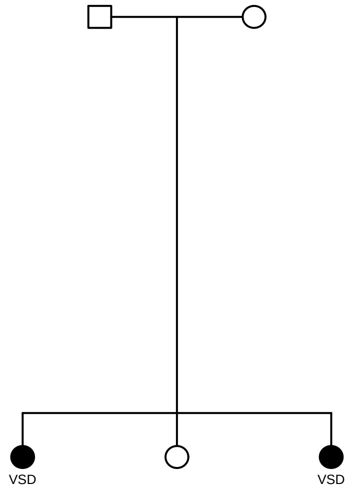

Wang_7 PMID:23021226

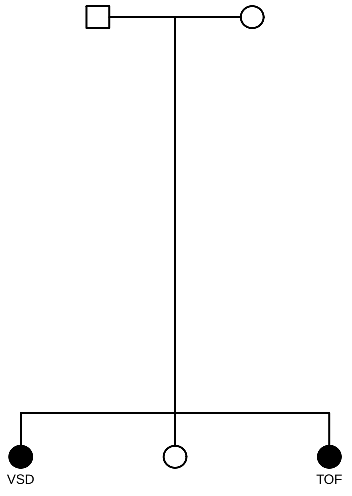

Wang_8 PMID:23021226

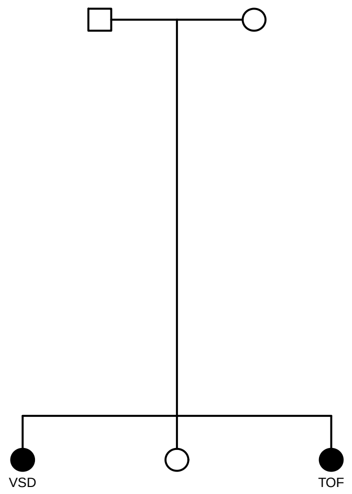

Wang_9 PMID:23021226

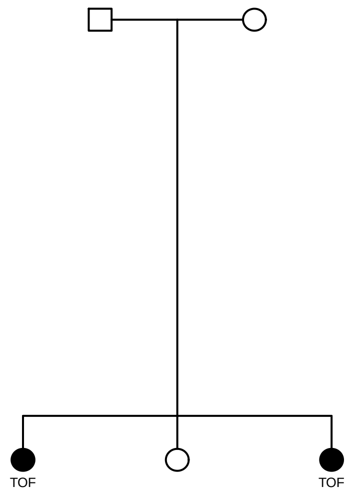

Wang_10 PMID:23021226

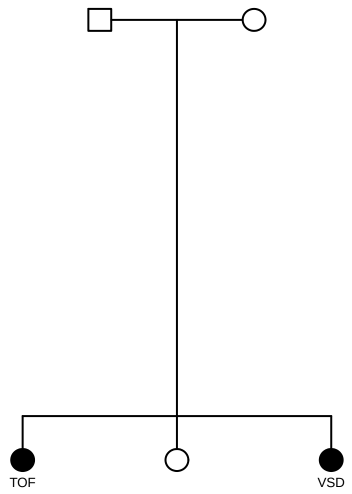

Wang_11 PMID:23021226

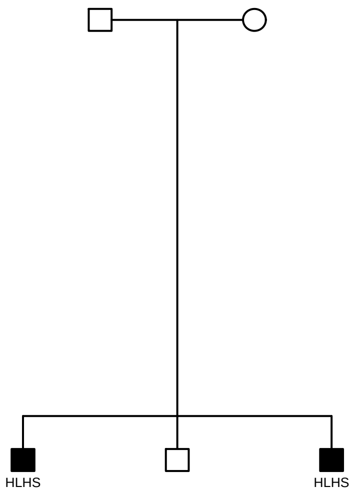

Wang_12 PMID:23021226

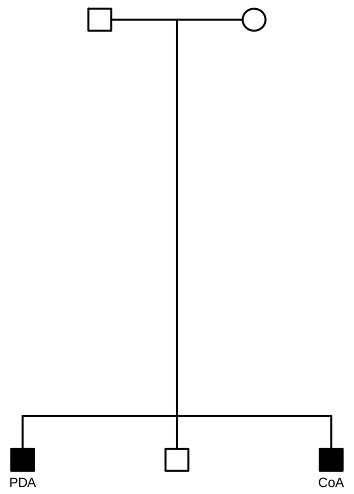

Wang_13 PMID:23021226

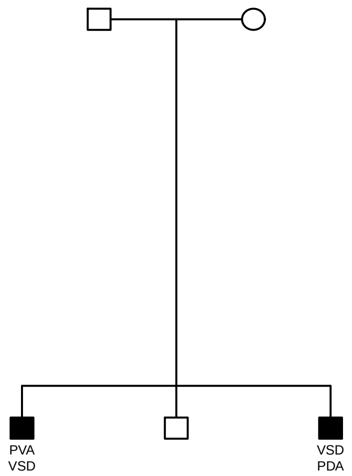

Wang_15 PMID:23021226

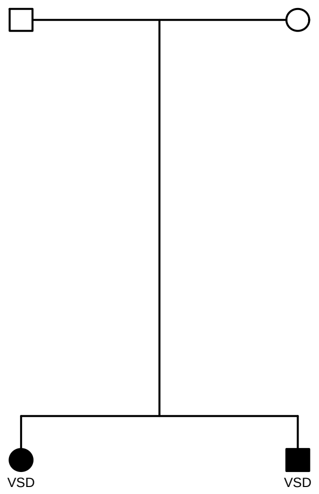

Wang_16 PMID:23021226

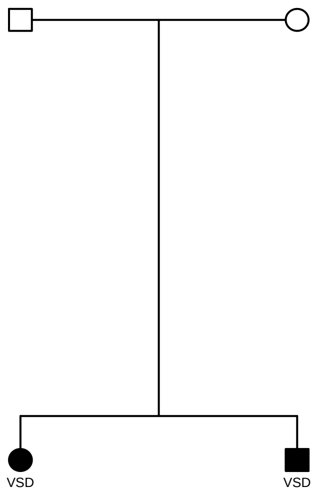

Wang_17 PMID:23021226

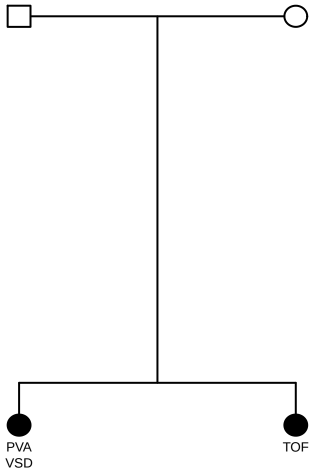

Wang_19 PMID:23021226

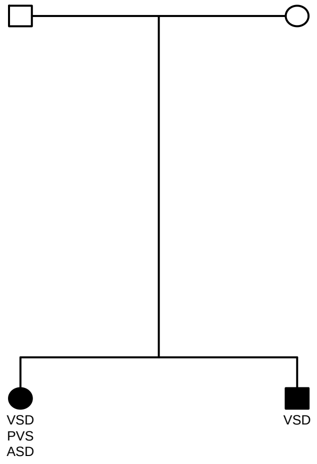

Wang_20 PMID:23021226

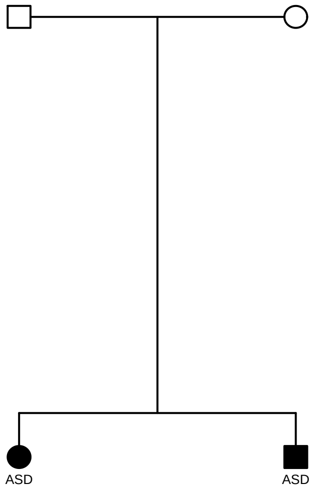

Wang_21 PMID:23021226

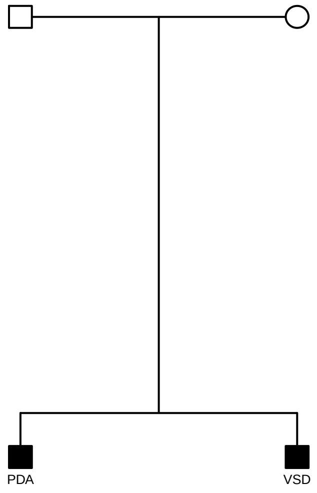

Yang_1 PMID:22648249

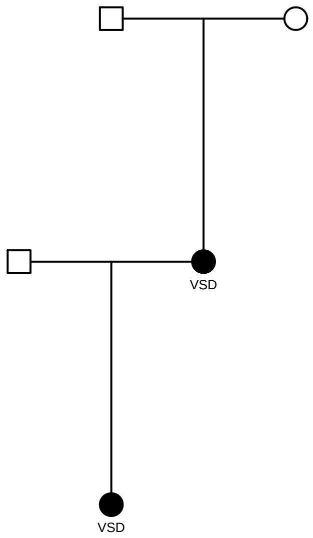

Yang_2 PMID:22648249

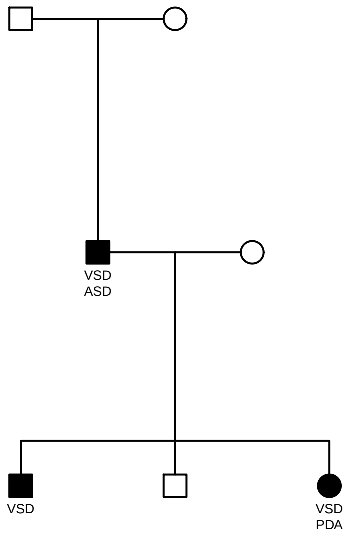

Yang_3 PMID:22648249

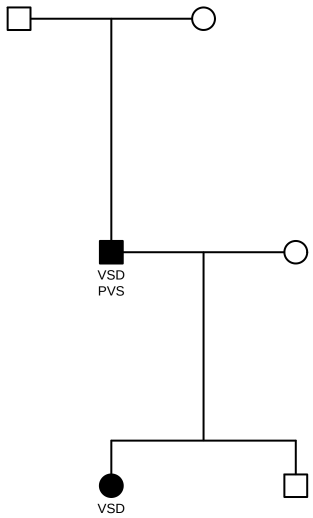

Zheng_2012 PMID:22407241

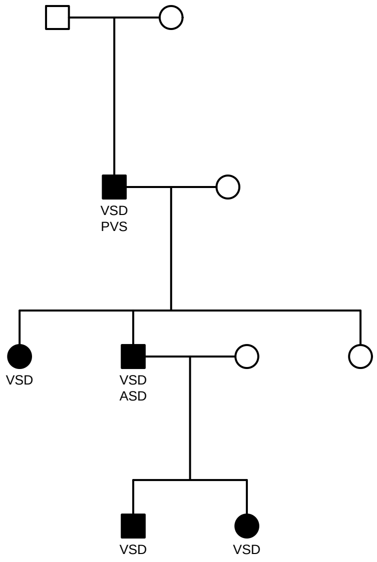

Yang_2012_2 PMID:22101736

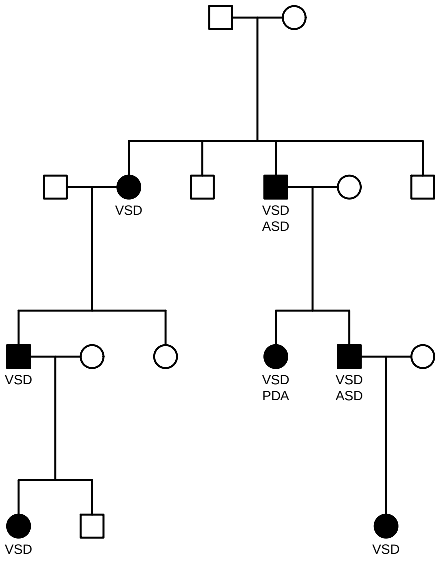

Zhang_2008 PMID:18672102

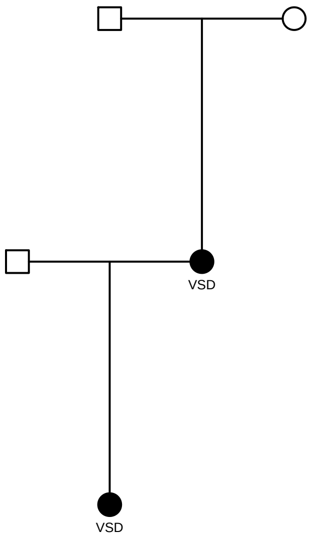

Wang_2011 PMID:21165553

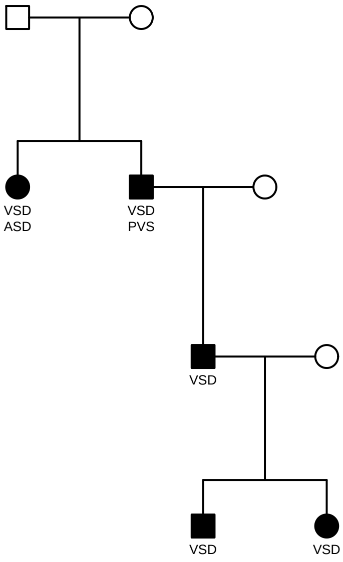

Cymbron_2006 PMID:16612061

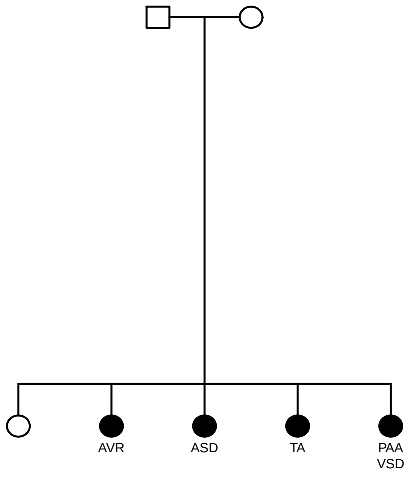

Nicolae_2007 PMID:17637070

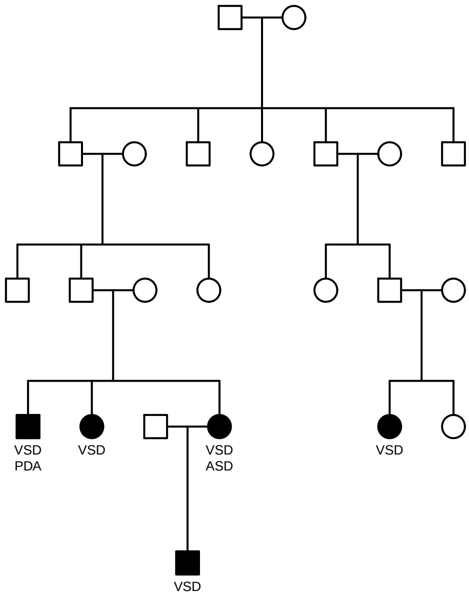

Czeizel_1 PMID:7215392

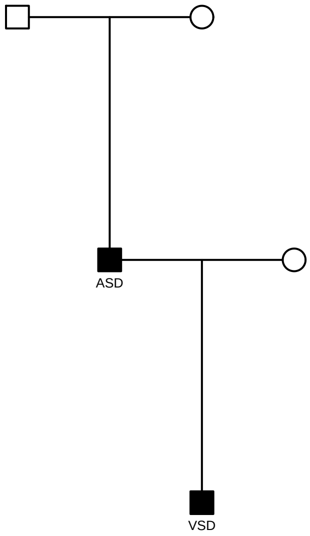

Czeizel_2 PMID:7215392

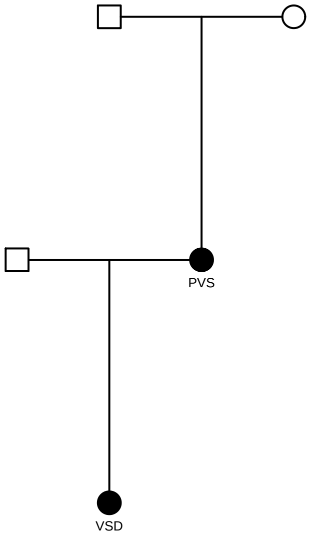

Czeizel_3 PMID:7215392

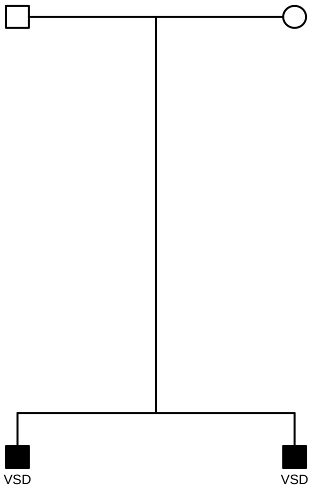

Czeizel_4 PMID:7215392

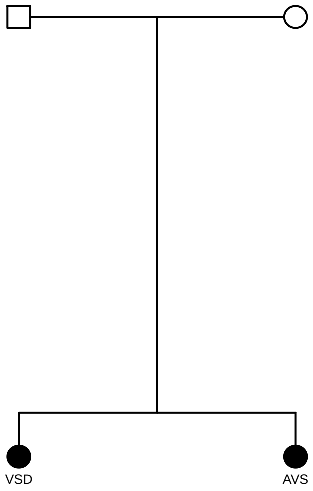

Czeizel_5 PMID:7215392

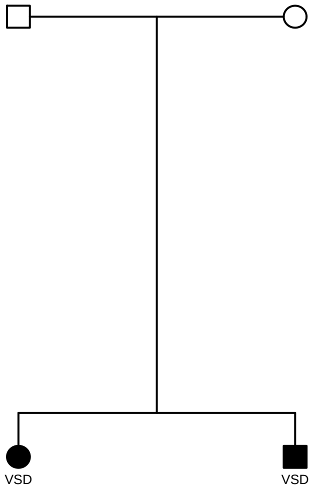

Czeizel_6 PMID:7215392

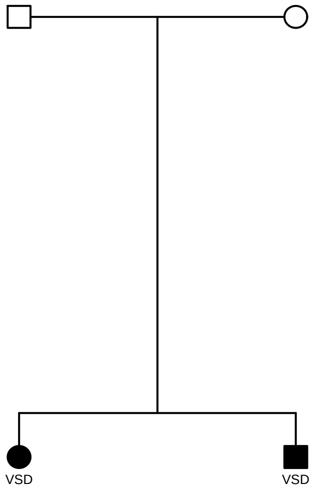

Czeizel_7 PMID:7215392

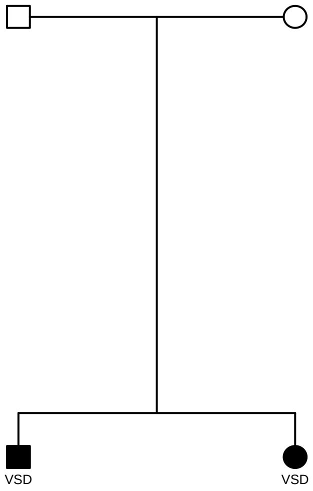

Czeizel_8 PMID:7215392

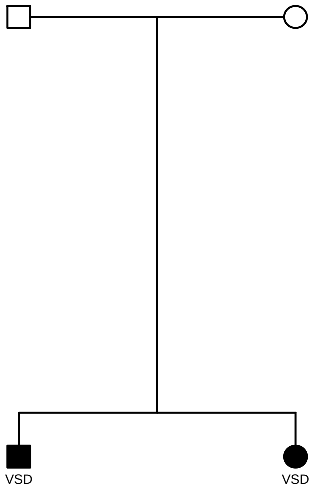

Czeizel_9 PMID:7215392

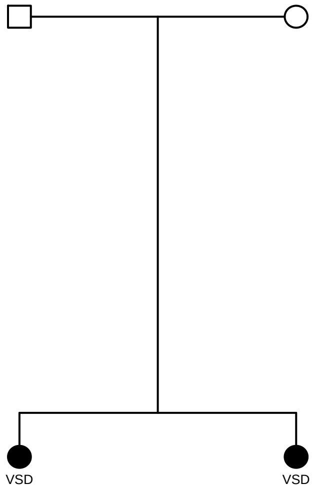

Czeizel_10 PMID:7215392

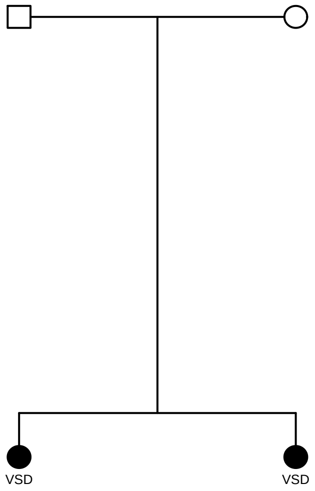

Solymar_1 PMID:3564987

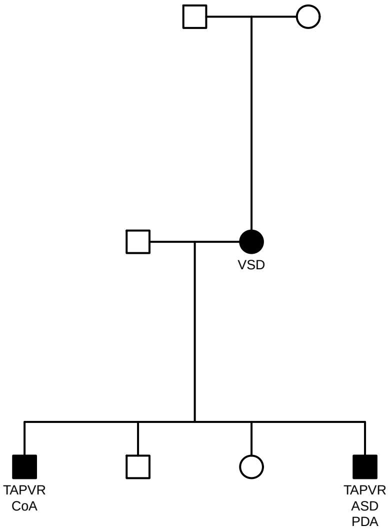

Solymar_2 PMID:3564987

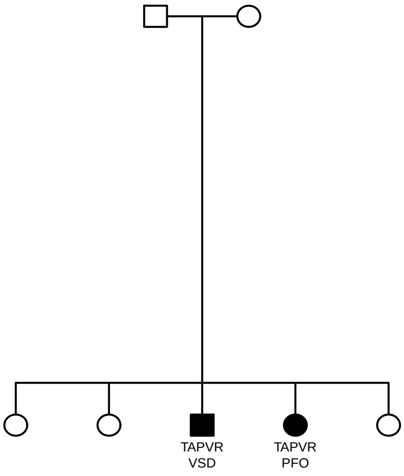

Solymar_3 PMID:3564987

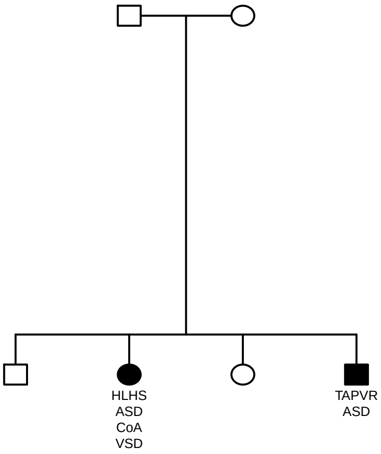

Gleason_1989 PMID:2686385

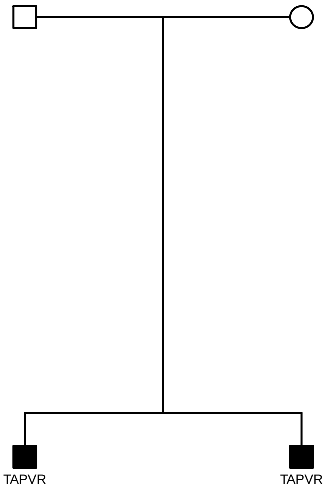

Baron_1982 PMID:7137007

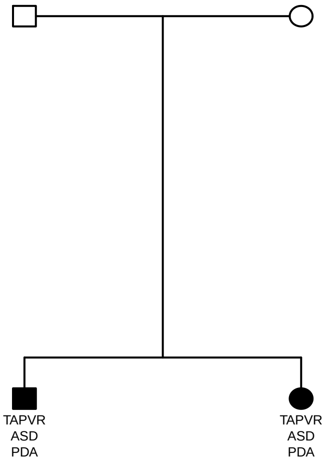

Ferrero_1997 PMID:9311745

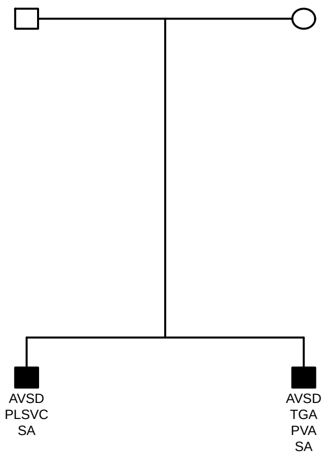

Milner_1 PMID:564667

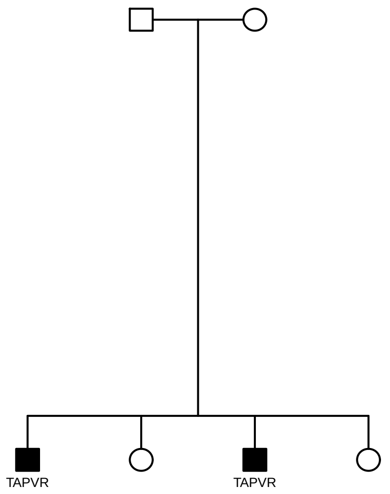

Ruggieri_2003 PMID:12494437

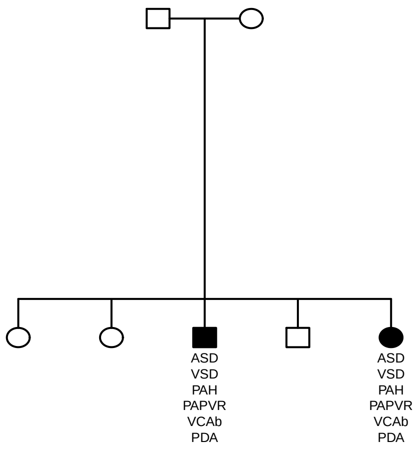

Ashida_2001 PMID:11425786

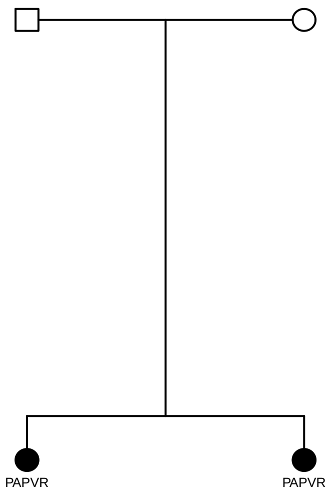

Bleyl_A PMID:7747759

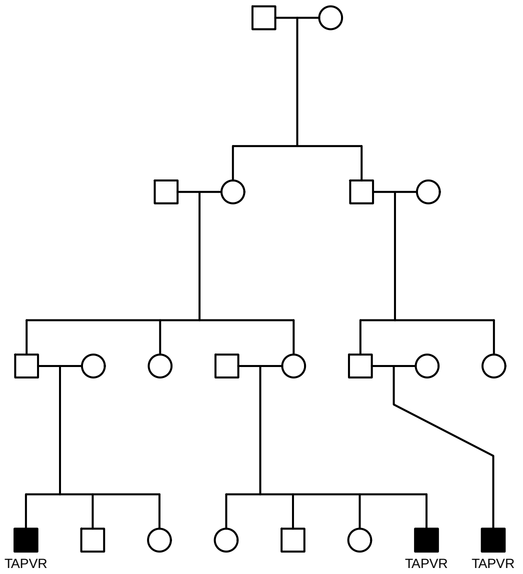

Bleyl_B PMID:7747759

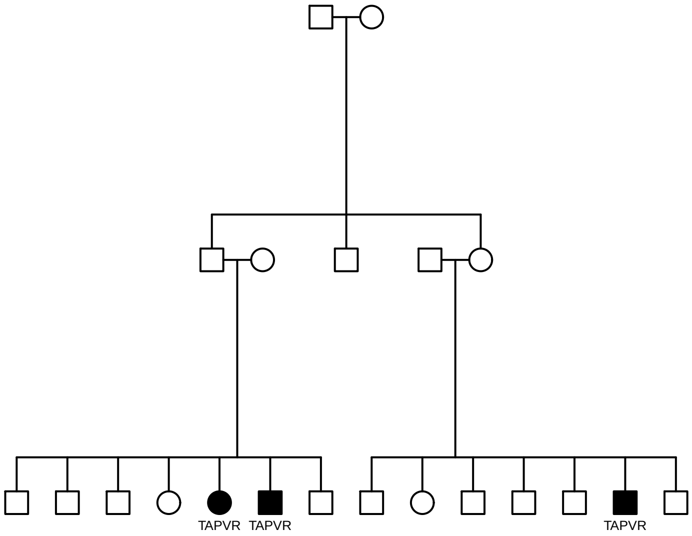

Bleyl_C PMID:7747759

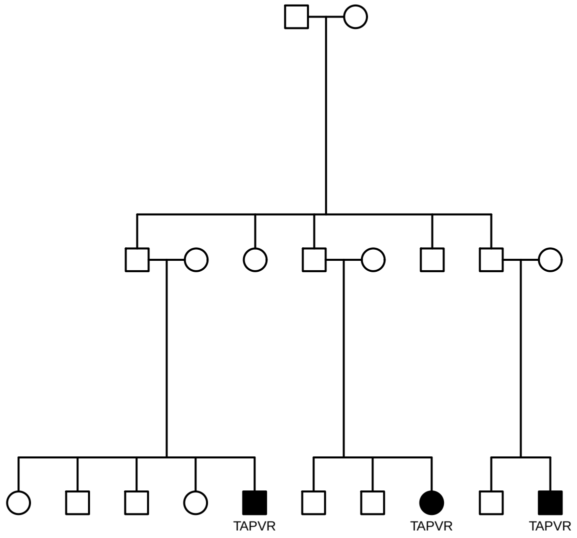

Paz_1971 PMID:5097138

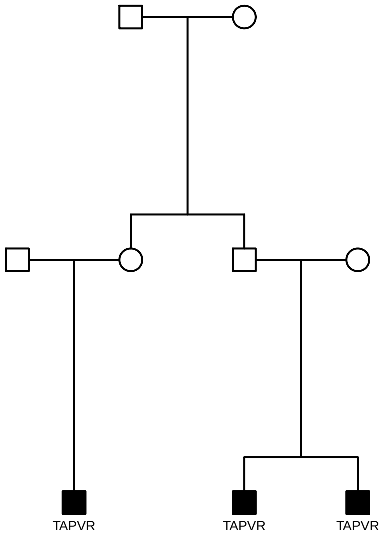

Devriendt_1994 PMID:7813525

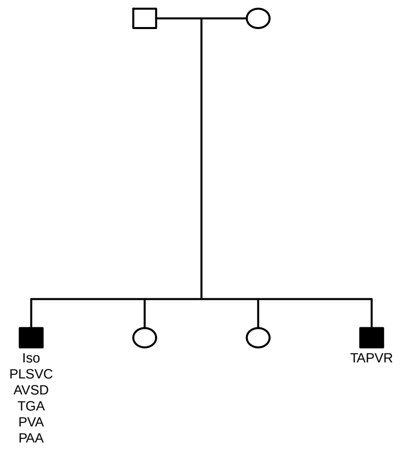

Raisher_1991 PMID:1887837

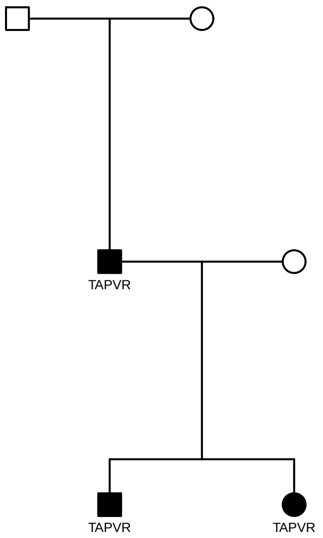

Vergara_2006_B PMID:16523515

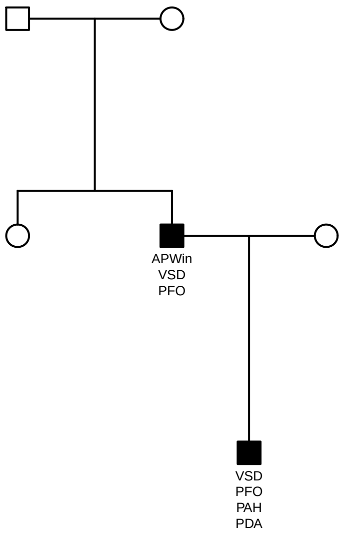

Dennis_8 PMID:7253006

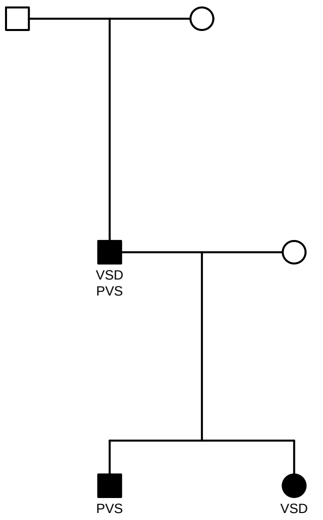

Dennis_16 PMID:7253006

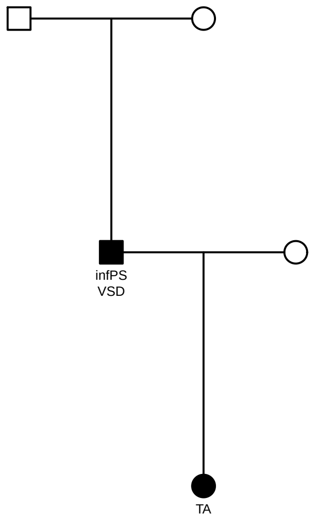

Dennis_29 PMID:7253006

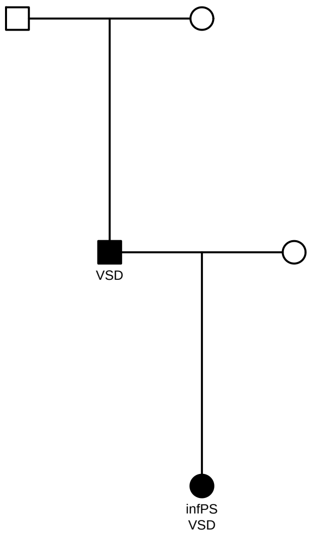

Dennis_32 PMID:7253006

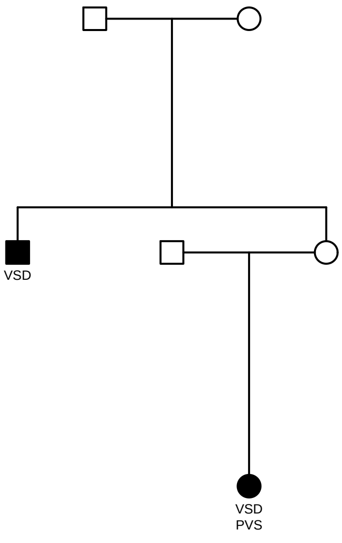

Dennis_70 PMID:7253006

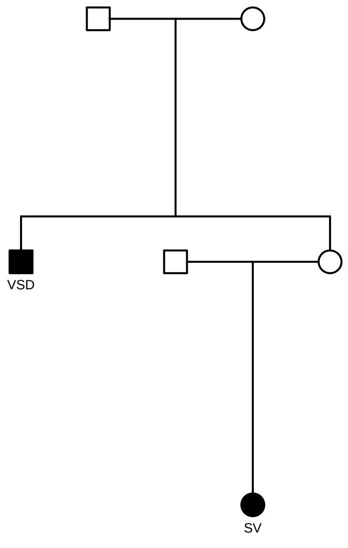

Dennis_105 PMID:7253006

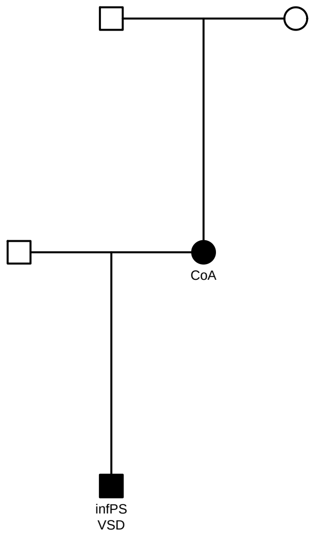

Dennis_185 PMID:7253006

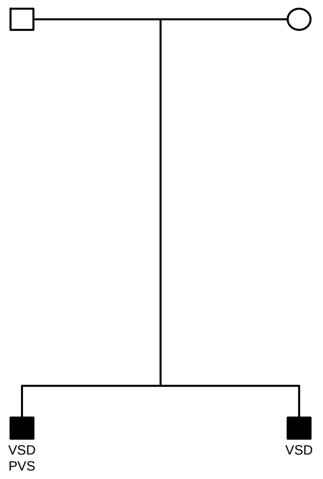

Dennis_215 PMID:7253006

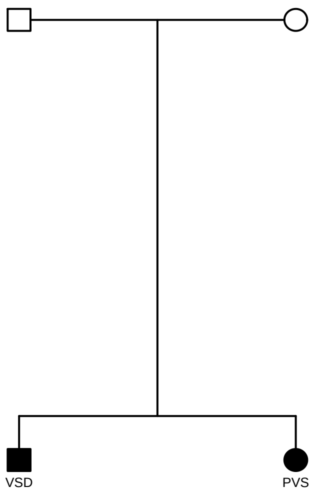

Dennis_240 PMID:7253006

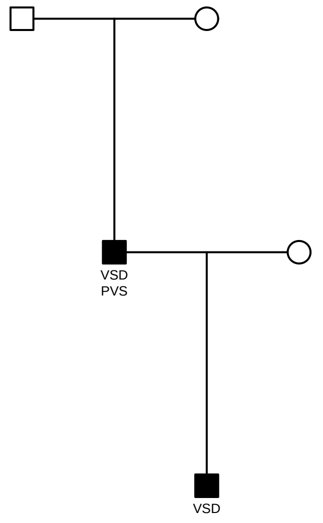

Dennis_254 PMID:7253006

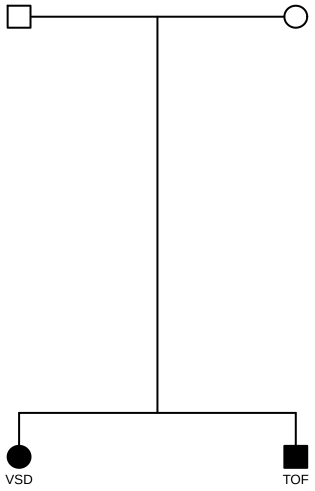

Dennis_261 PMID:7253006

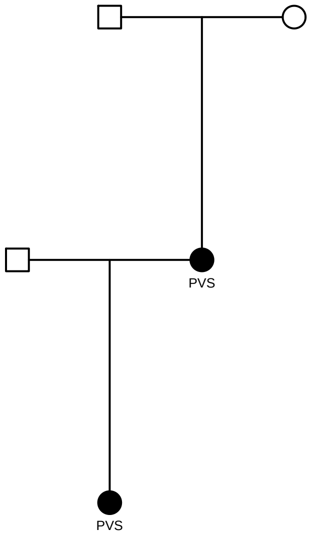

Dennis_276 PMID:7253006

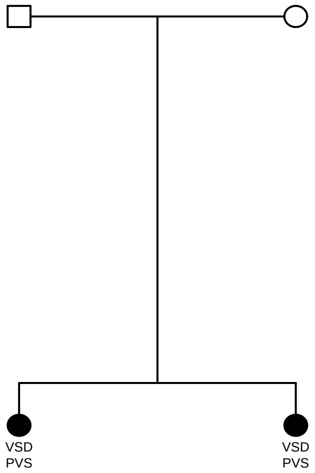

Dennis_284 PMID:7253006

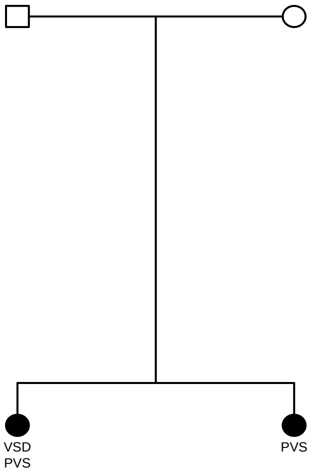

Dennis_286 PMID:7253006

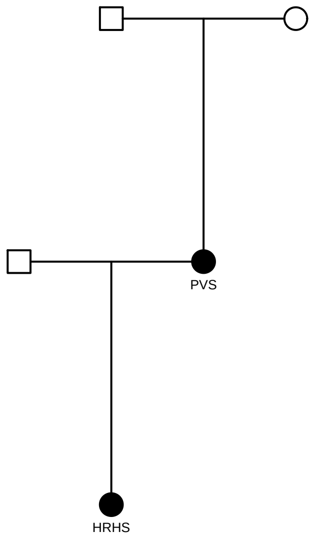

Dennis_426 PMID:7253006

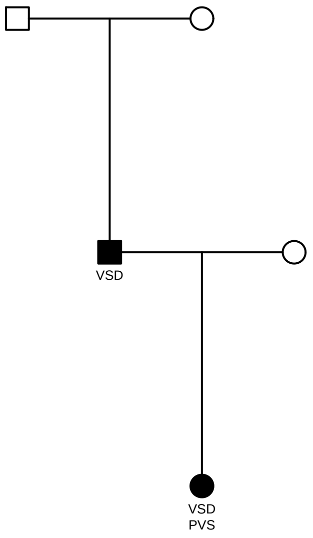

Weigel_1 PMID:2801528

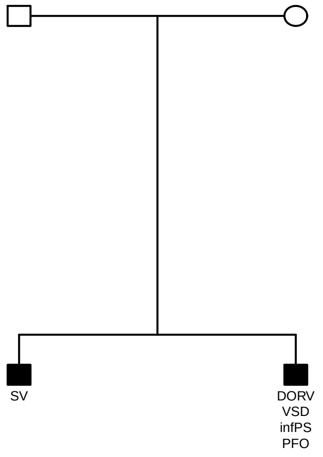

Weigel_2 PMID:2801528

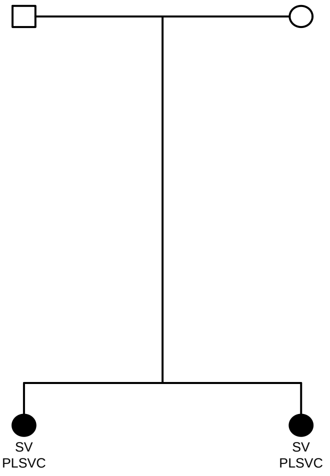

Weigel_3 PMID:2801528

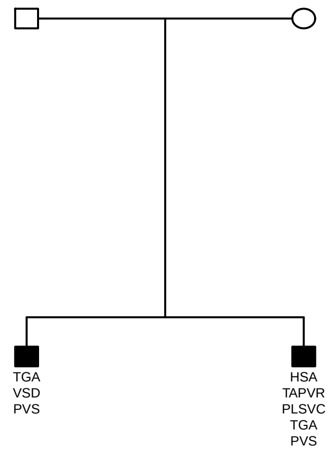

Weigel_4 PMID:2801528

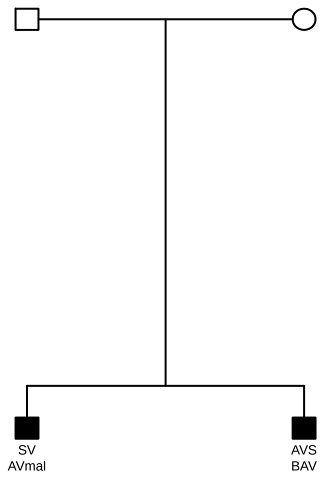

Weigel_5 PMID:2801528

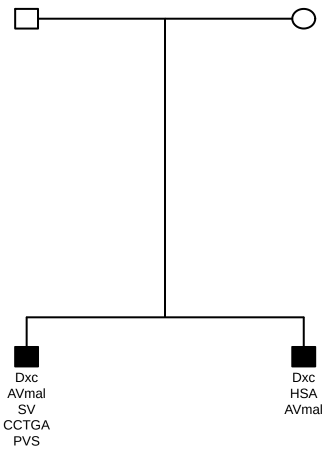

Weigel_6 PMID:2801528

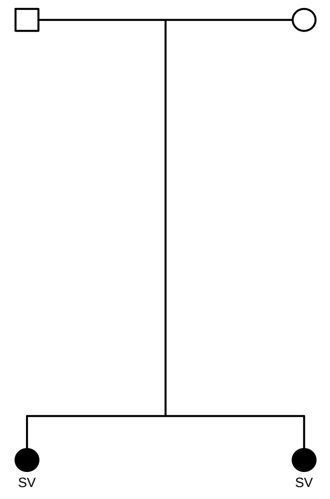

Weigel_7 PMID:2801528

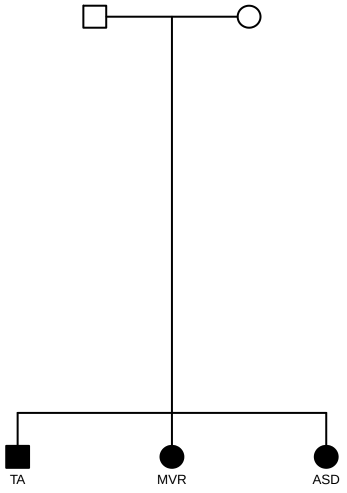

Shapiro_1981 PMID:6455913

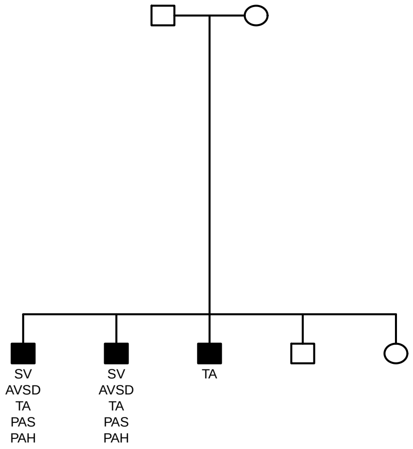

Bonnet_A PMID:10227411

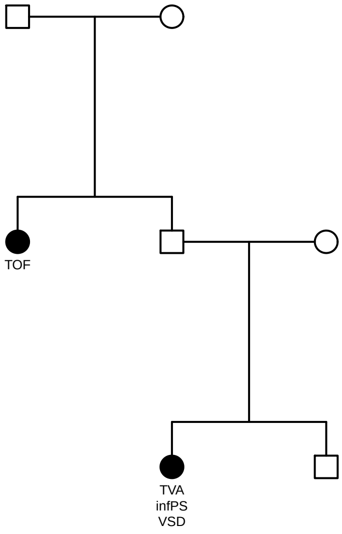

Bonnet_B PMID:10227411

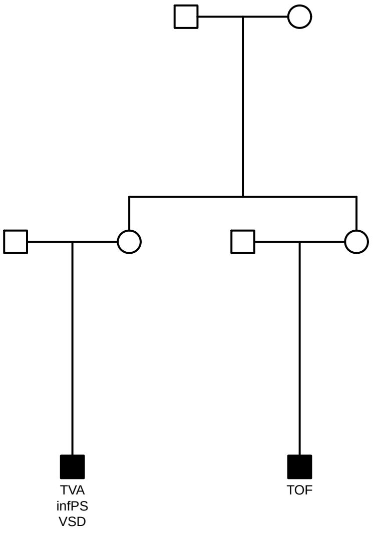

Bonnet_C PMID:10227411

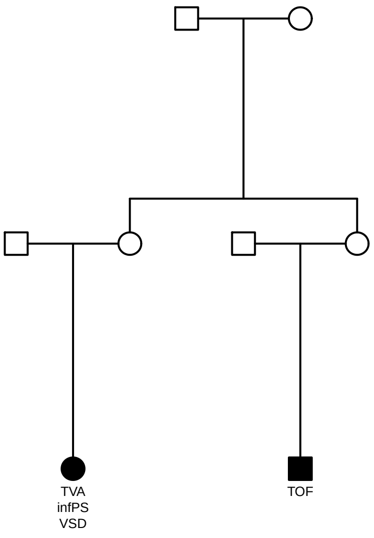

Bonnet_D PMID:10227411

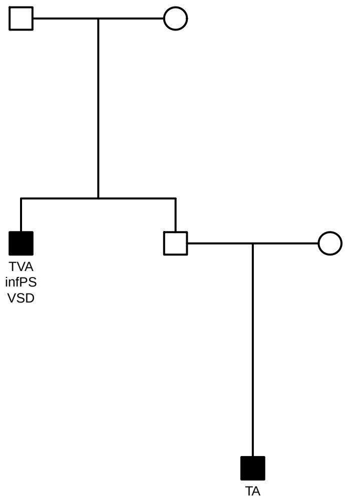

Bonnet_E PMID:10227411

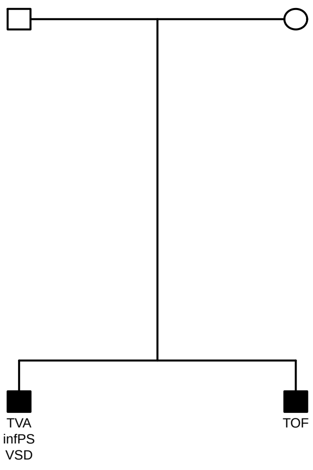

Davachi_1967 PMID:6070007

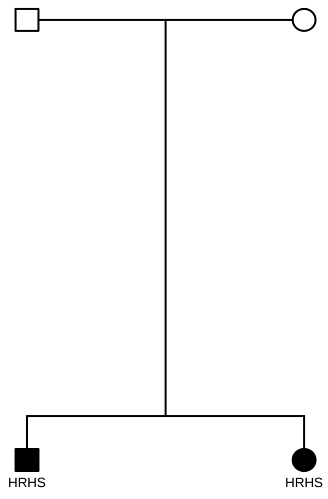

Medd_1960 PMID:13768823

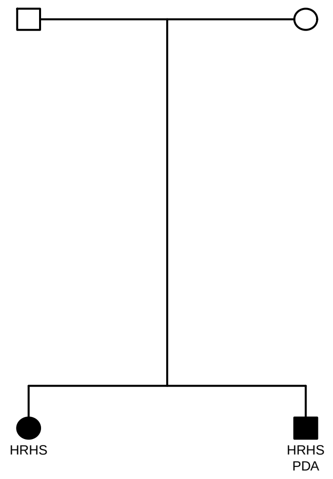

Sackner_1961 PMID:14495868

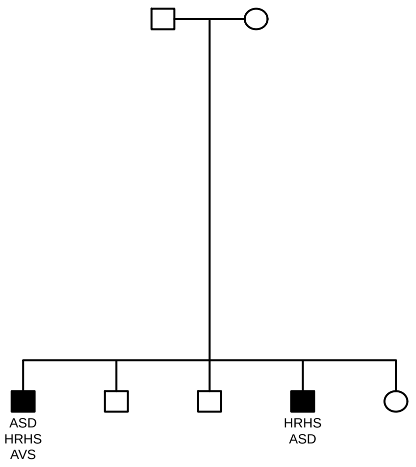

Pierard_1985 PMID:4005080

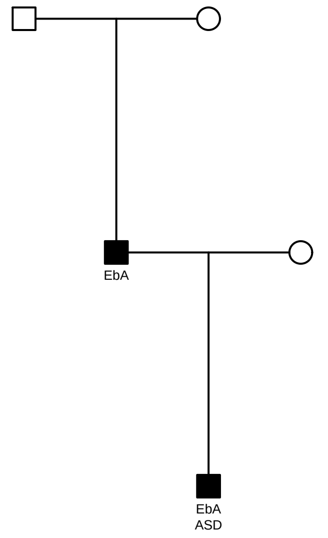

McIntosh_1992 PMID:1536167

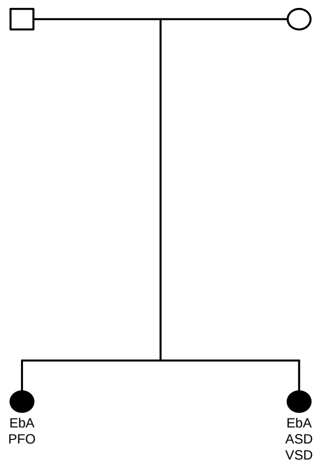

Kumar_1994_b PMID:7991439

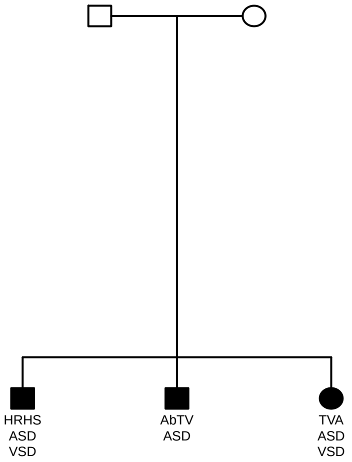

Grant_1996 PMID:8660450

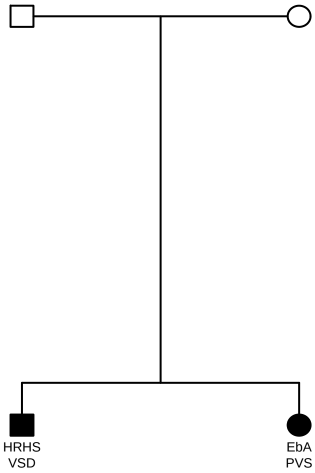

Balaji_1991 PMID:1854572

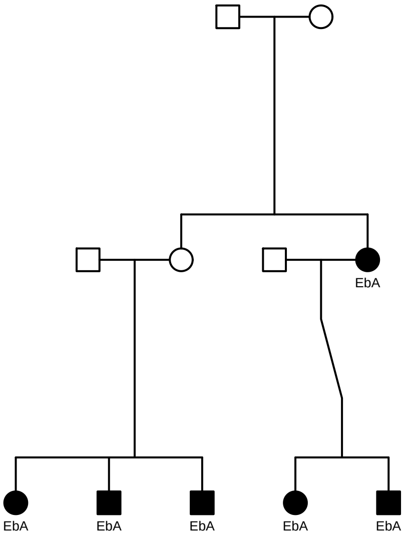

Gueron_1966 PMID:5938901

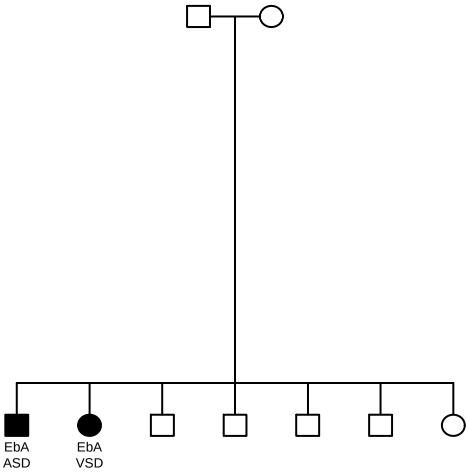

Rosenmann_1 PMID:1018315

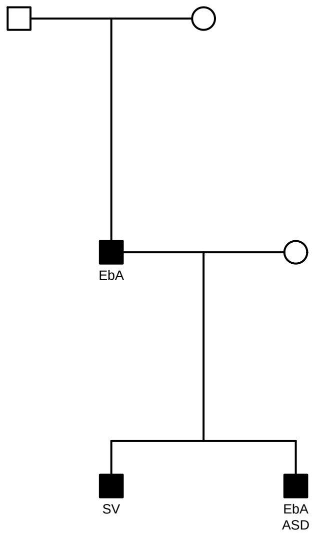

Donegan_1968 PMID:5638476

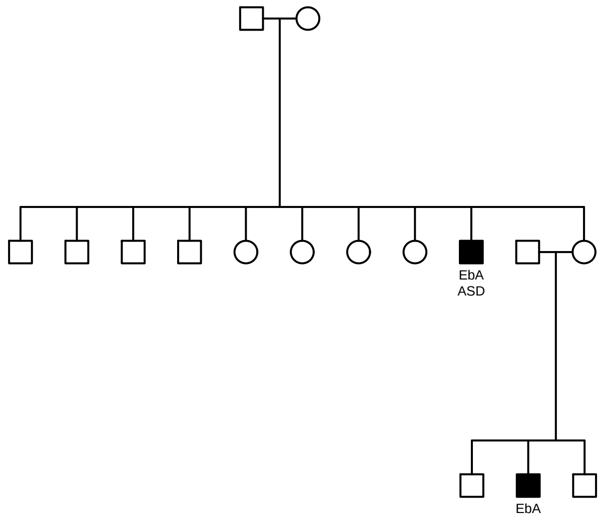

Lo_1979 PMID:476731

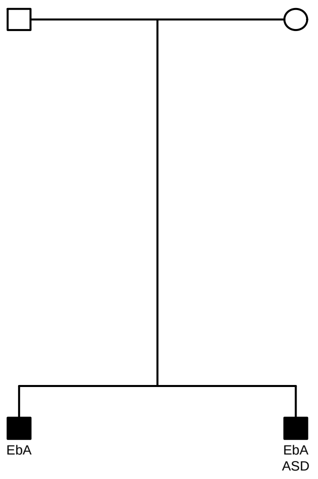

Uyan_2002 PMID:12537411

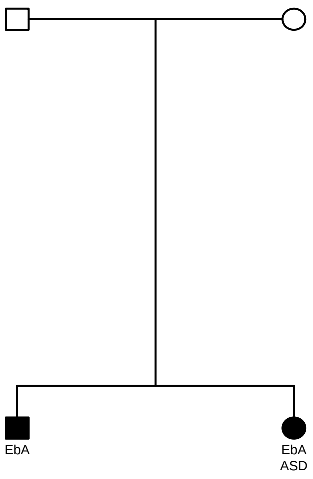

Disegni_1 PMID:4003317

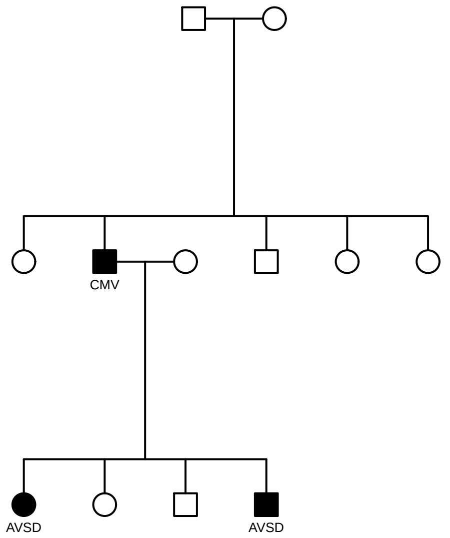

Disegni_2 PMID:4003317

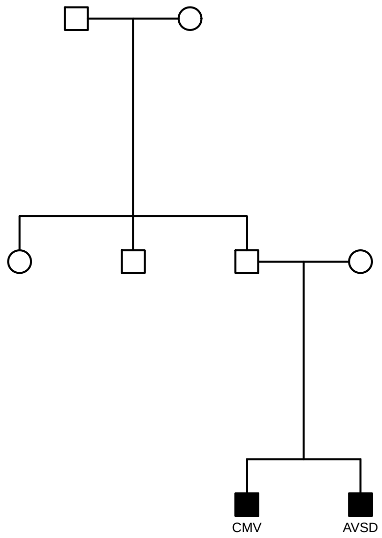

Disegni_3 PMID:4003317

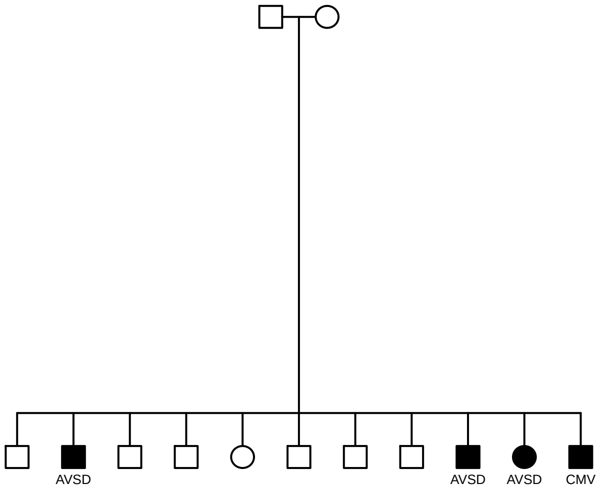

Disegni_4 PMID:4003317

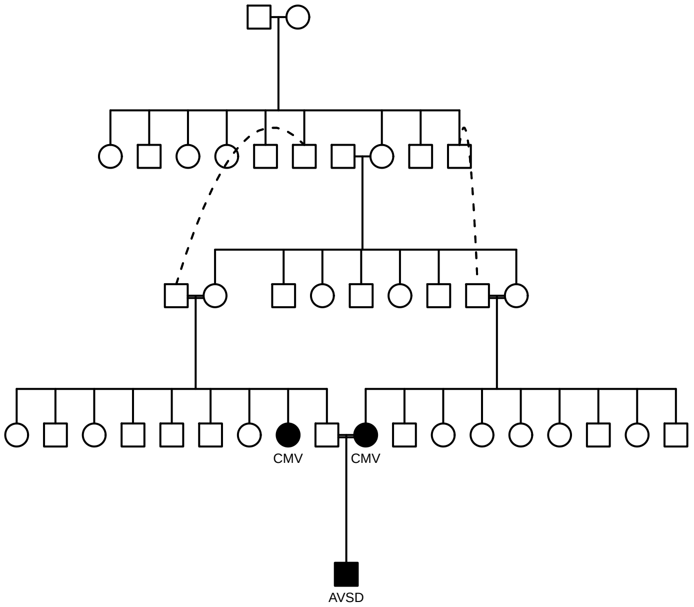

Yao_1968 PMID:20329145

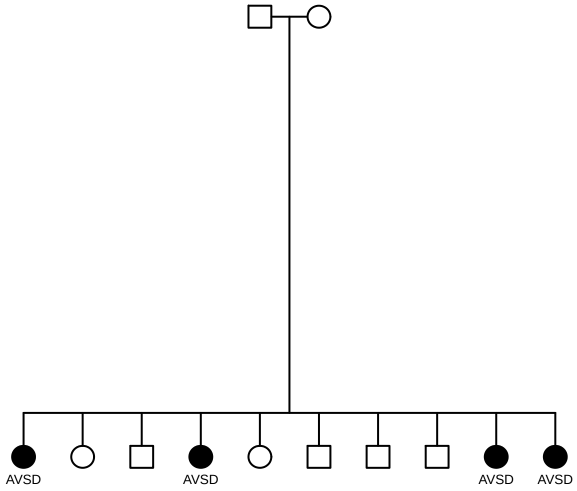

Gelernter−Yaniv_1 PMID:17462063

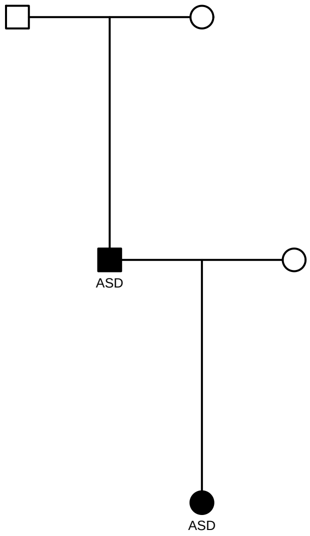

Gelernter−Yaniv_2 PMID:17462063

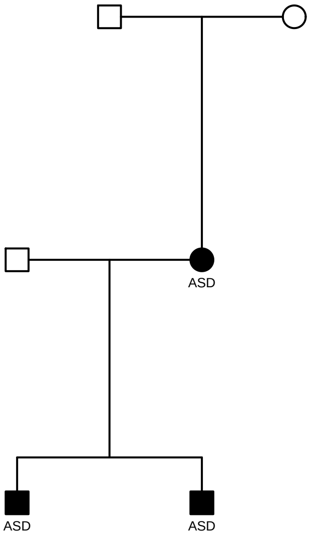

Gelernter−Yaniv_3 PMID:17462063

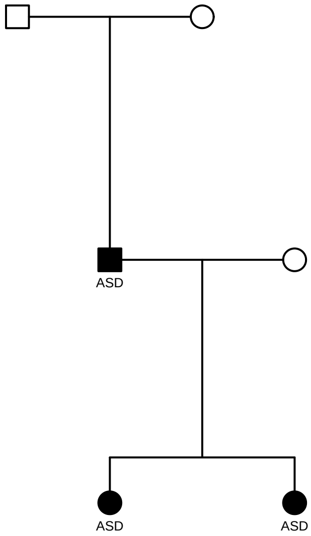

Gelernter−Yaniv_4 PMID:17462063

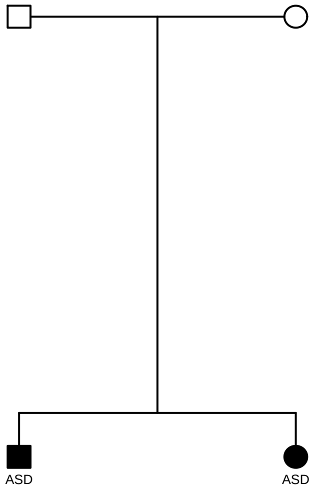

Gelernter−Yaniv_5 PMID:17462063

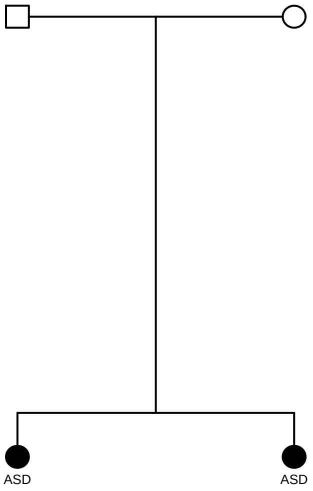

Gelernter−Yaniv_6 PMID:17462063

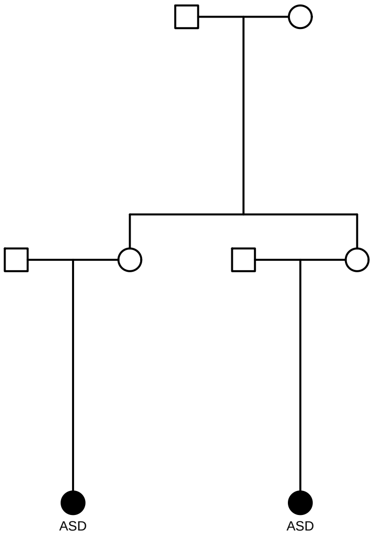

Schunkert_1997 PMID:9382022

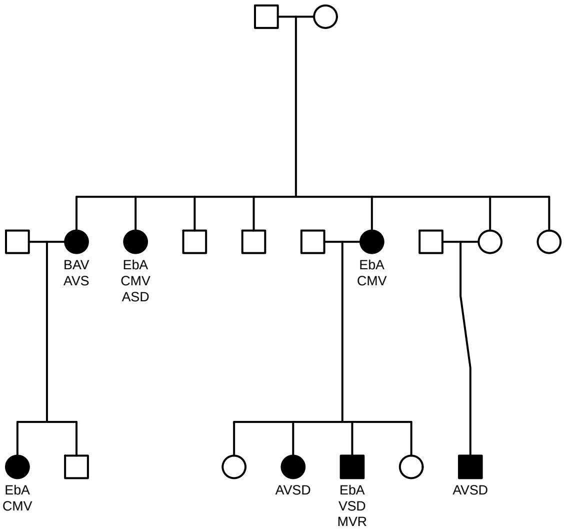

Amati_1 PMID:7677156

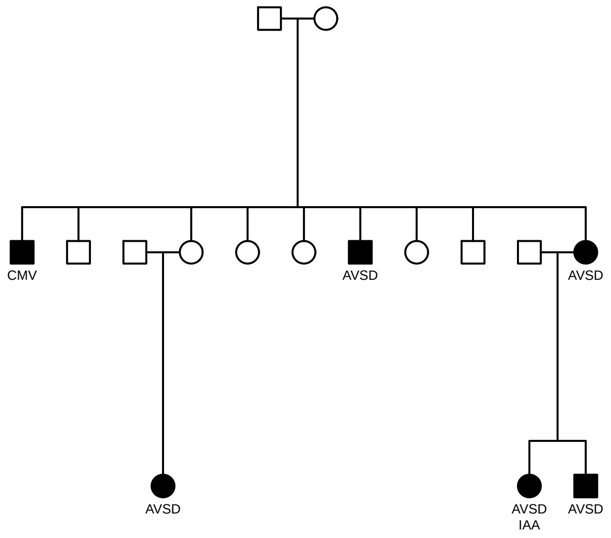

Amati_2 PMID:7677156

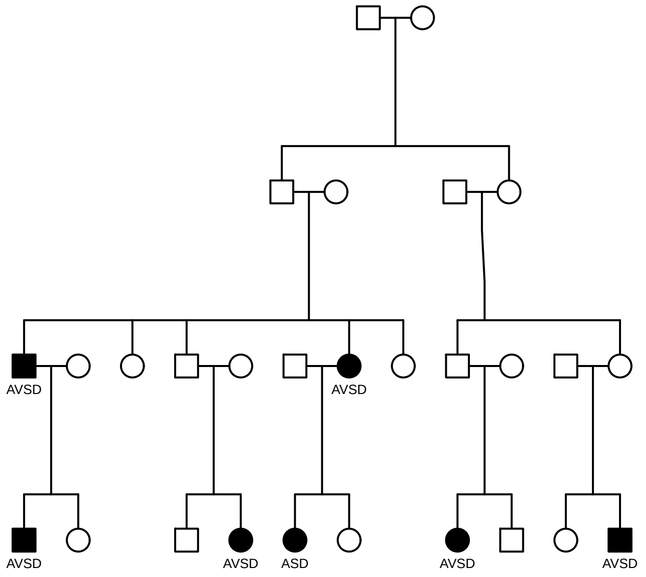

Emanuel_1 PMID:6824534

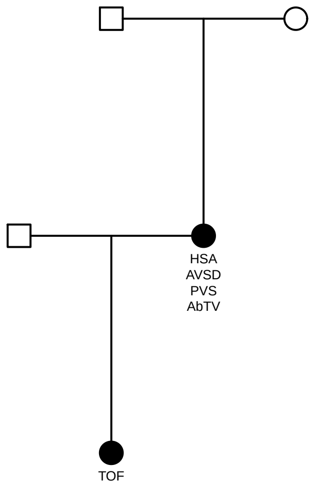

Emanuel_2 PMID:6824534

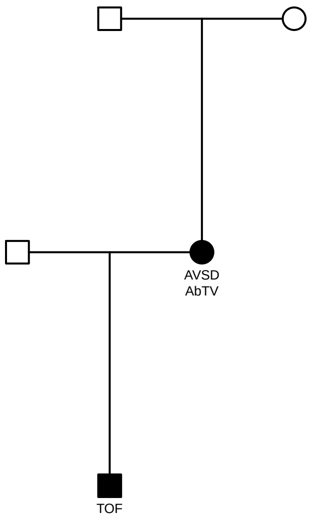

Emanuel_3 PMID:6824534

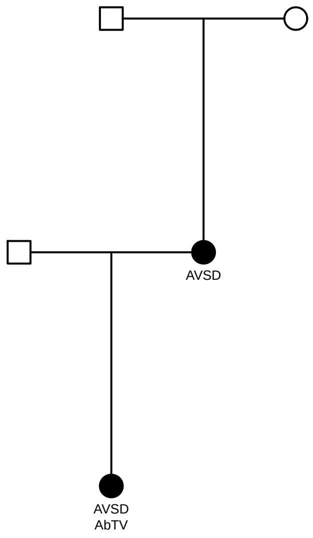

Emanuel_4 PMID:6824534

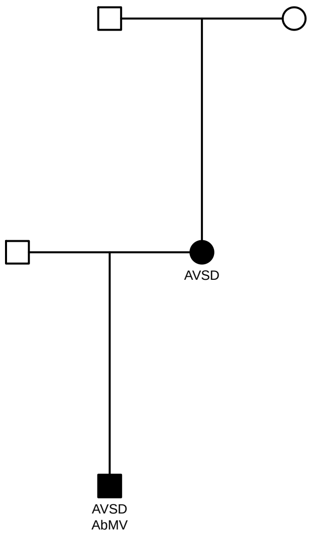

Digilio_1993_1 PMID:8249947

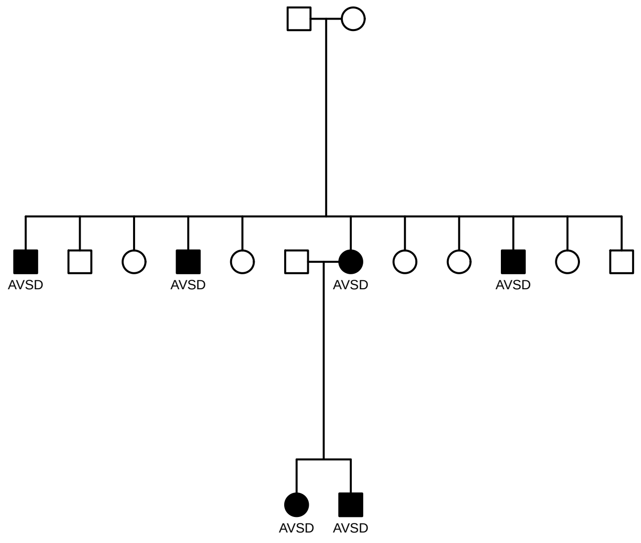

Digilio_1993_2 PMID:8249947

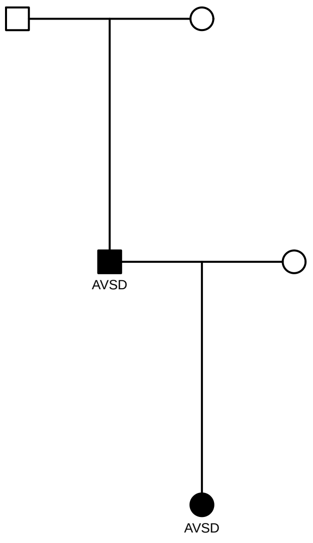

Digilio_1993_3 PMID:8249947

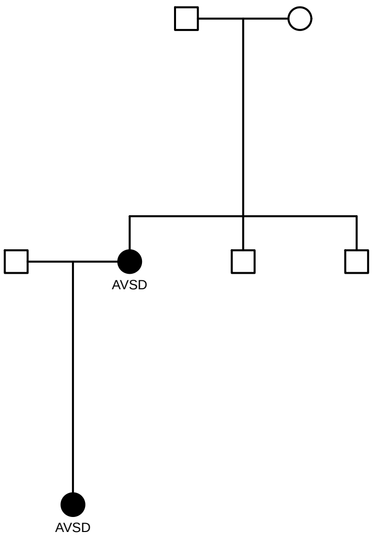

Digilio_1993_4 PMID:8249947

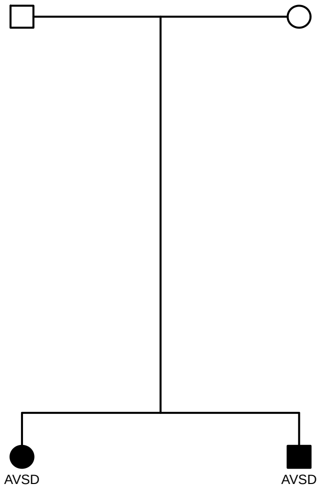

Digilio_1993_5 PMID:8249947

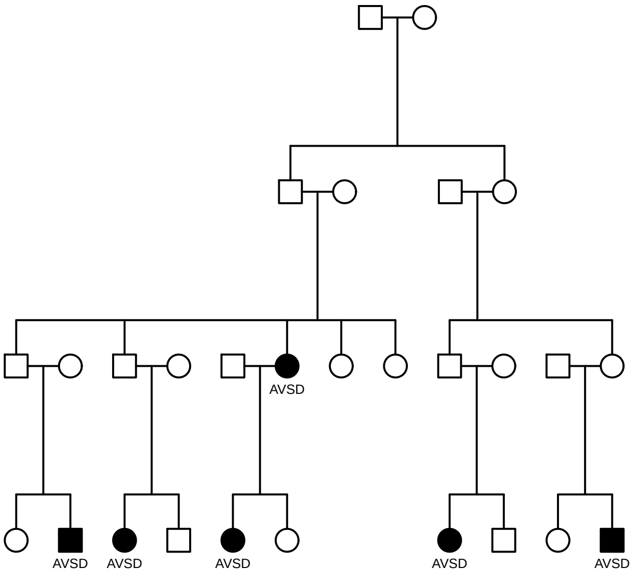

Cousineau_1994 PMID:8112730

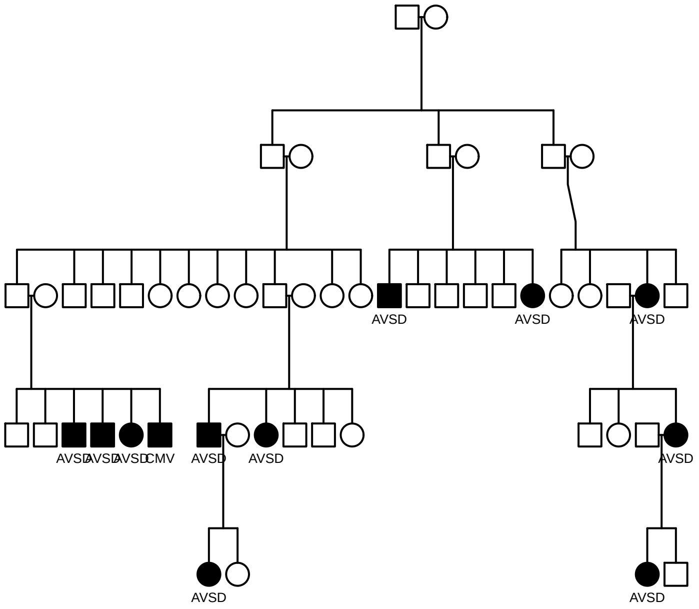

Wilson_1993 PMID:8250042

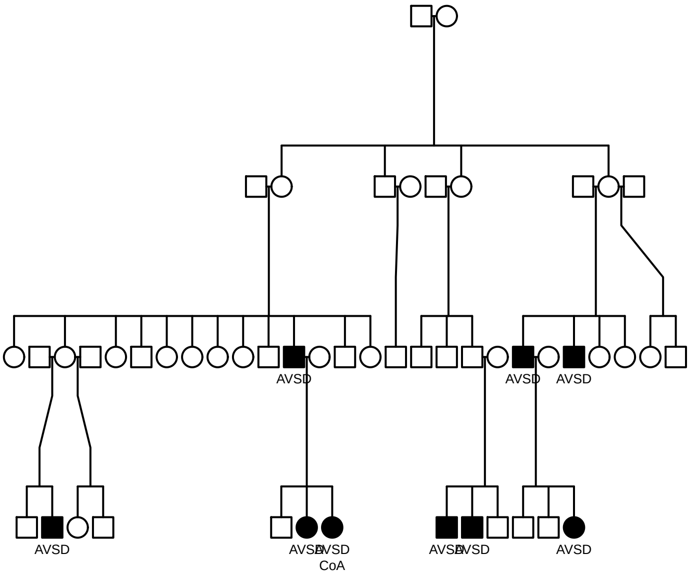

ONoullain_1977 PMID:884239

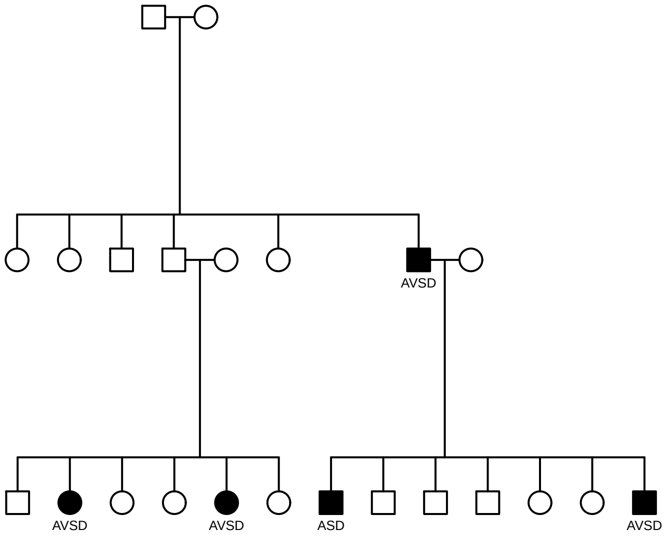

Digilio_1998_1 PMID:9805136

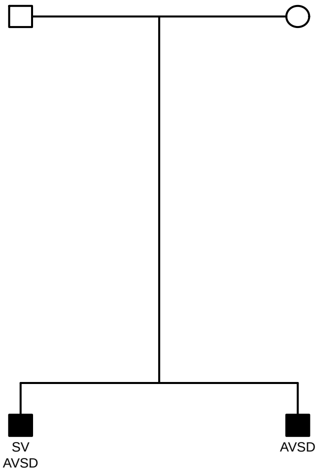

Digilio_1998_2 PMID:9805136

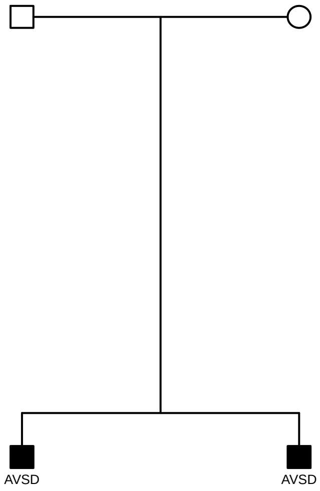

Digilio_1998_3 PMID:9805136

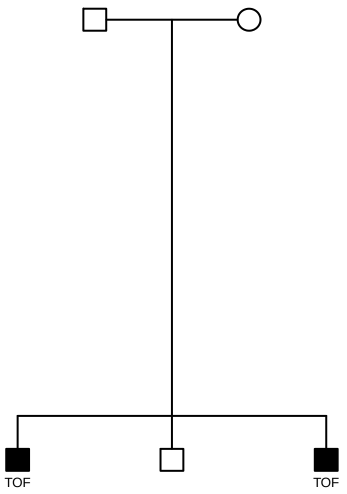

Digilio_1994 PMID:9805136

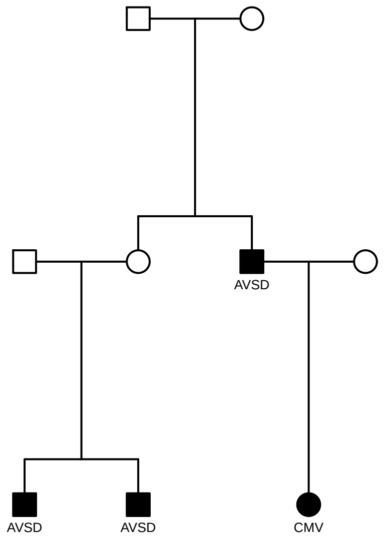

Kumar_1994_a PMID:8297702

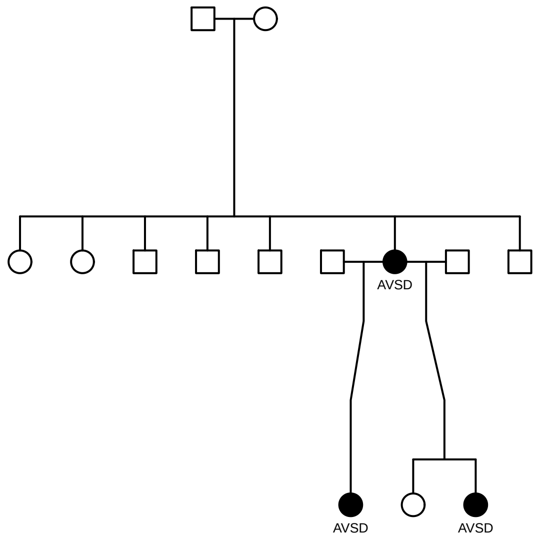

Weinstein_1958 PMID:13598778

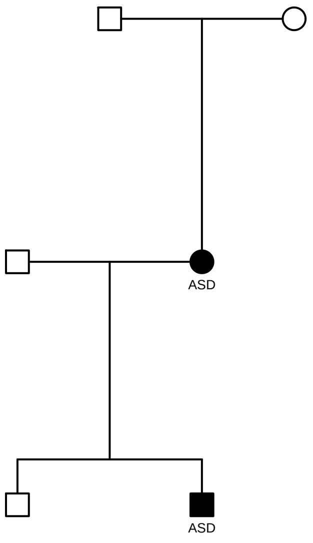

Weil_1959 PMID:Circulation:
Proceedings of the 32nd Scientific Session pg 782

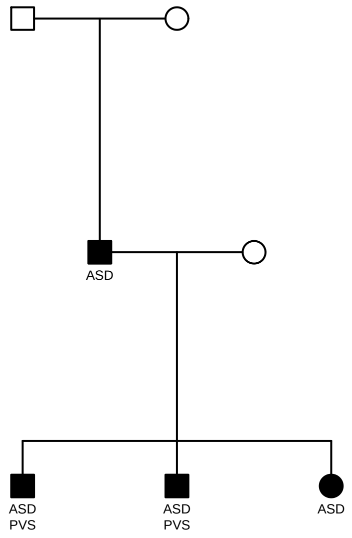

Zuckerman_1962 PMID:American Jounal of Cardiology; 1962;9;4;515−520

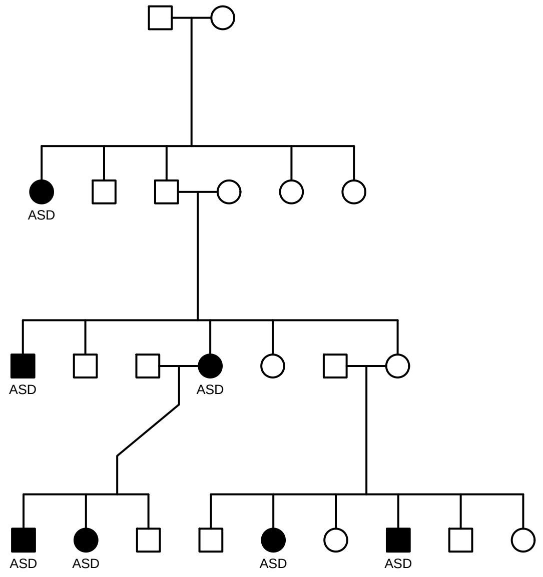

Skelton_1958 PMID:13608370

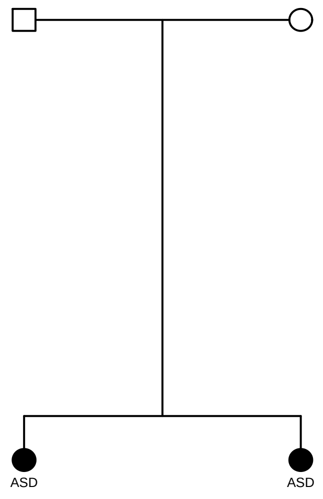

Williamson_15 PMID:5345096

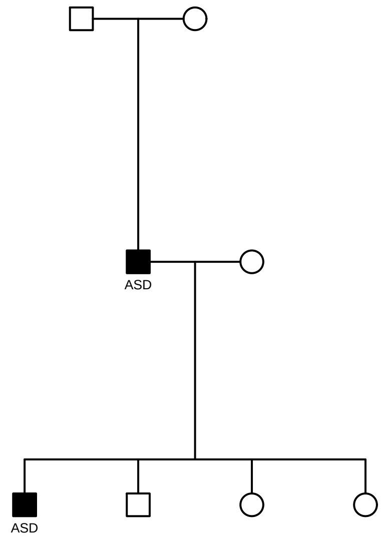

Williamson_16 PMID:5345096

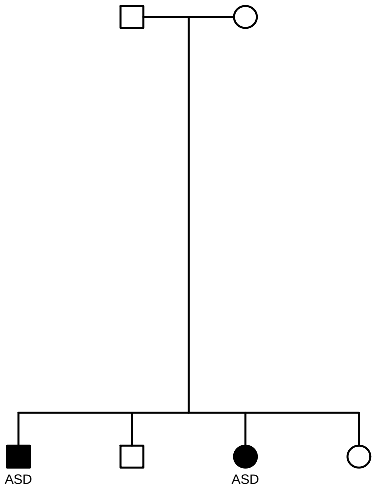

Williamson_17 PMID:5345096

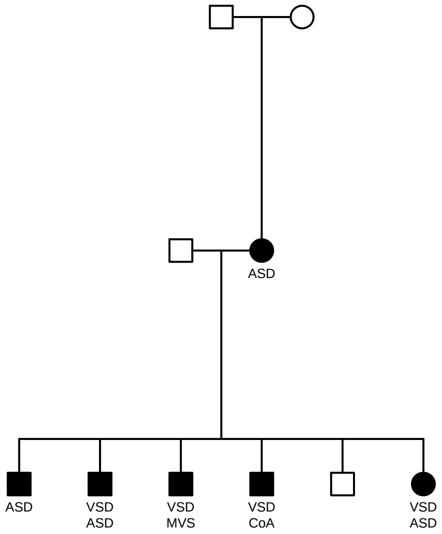

Williamson_34 PMID:5345096

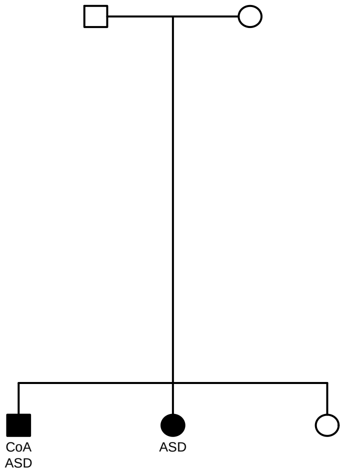

Williamson_47 PMID:5345096

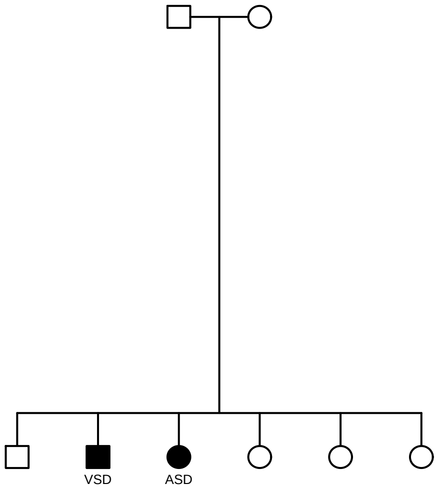

Williamson_54 PMID:5345096

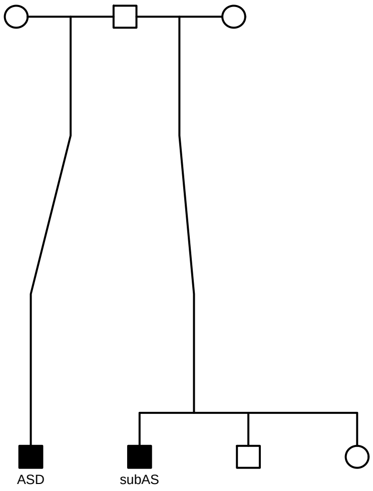

Williamson_71 PMID:5345096

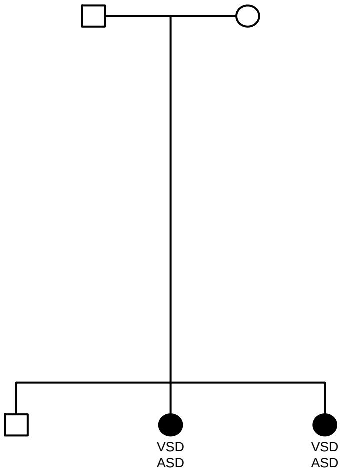

Williamson_83 PMID:5345096

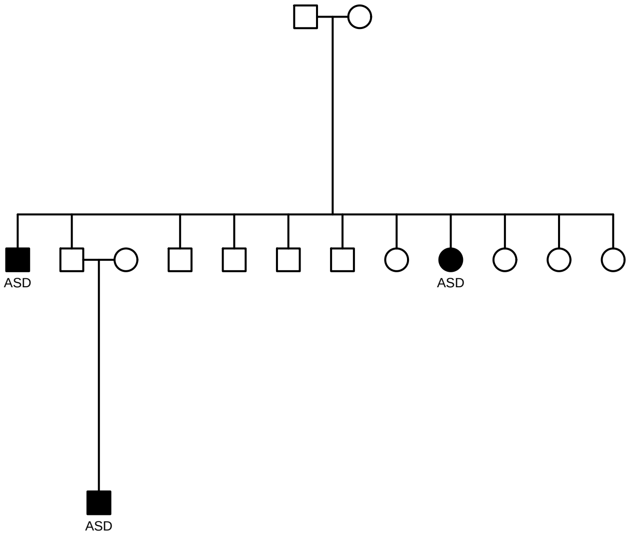

Williamson_87 PMID:5345096

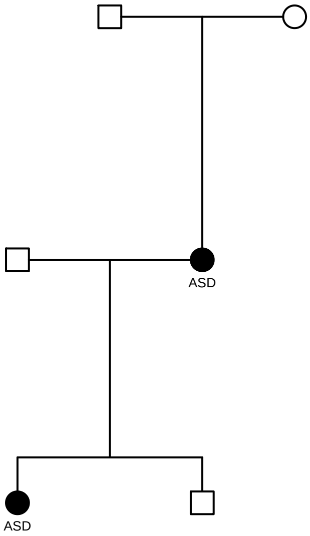

Williamson_88 PMID:5345096

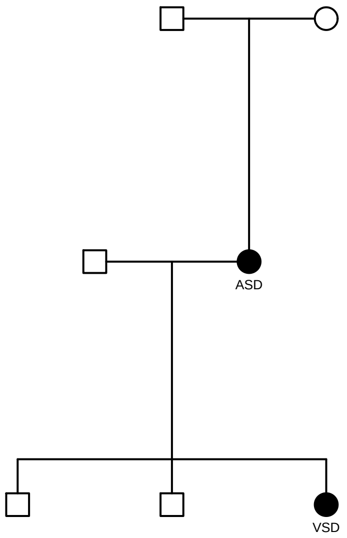

Williamson_111 PMID:5345096

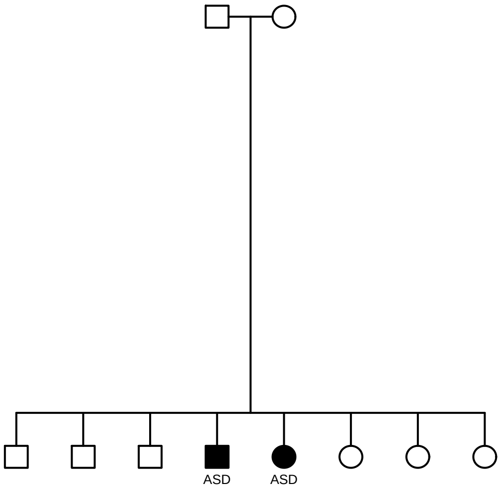

Williamson_117 PMID:5345096

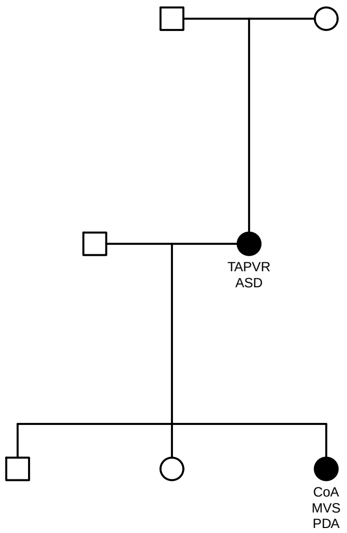

Williamson_121 PMID:5345096

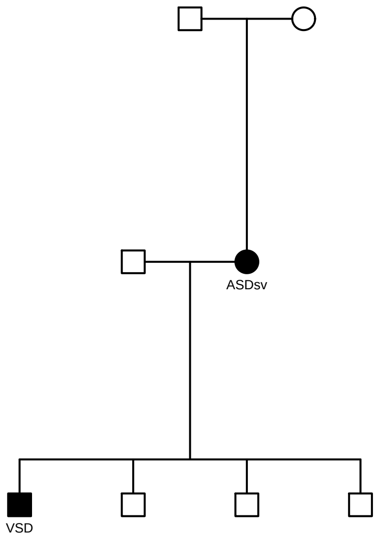

Maron_1978 PMID:718771

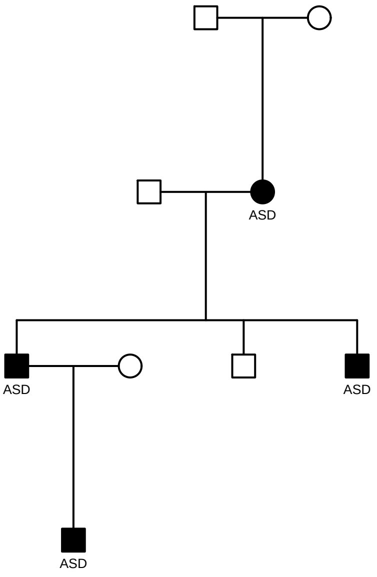

Megarbane_1999 PMID:10096596

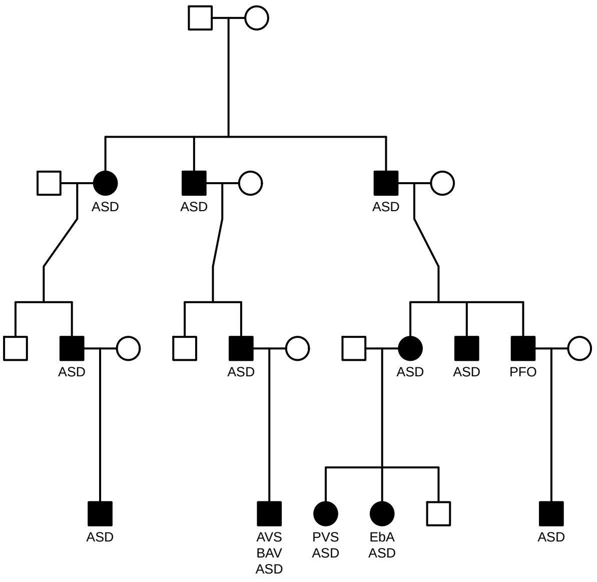

Gunal_1997 PMID:9363669

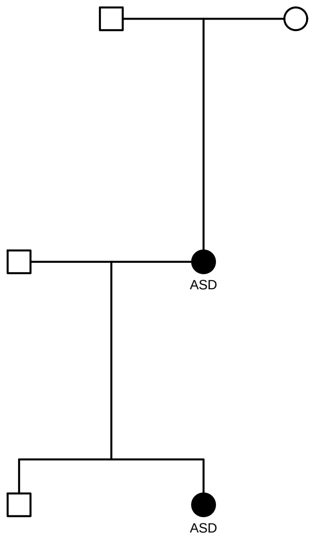

Bizarro_1970 PMID:5437412

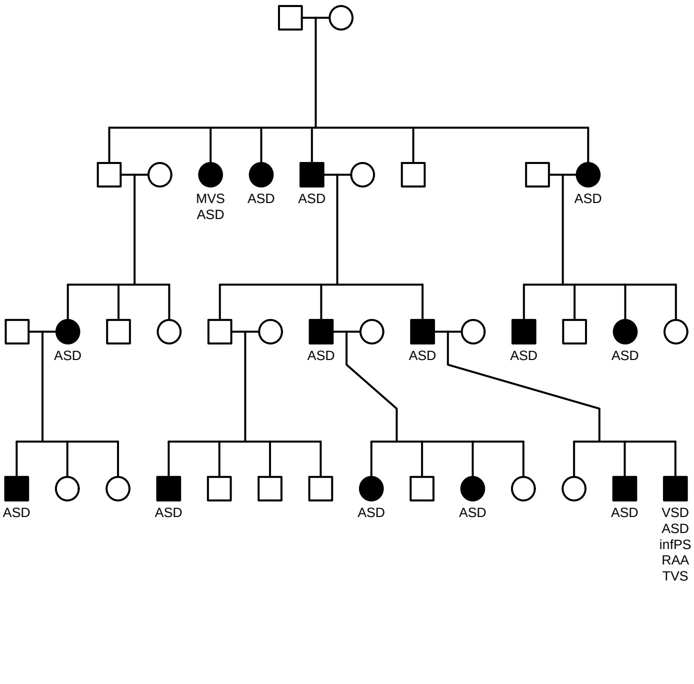

Wilmshurst_2004_2 PMID:15486131

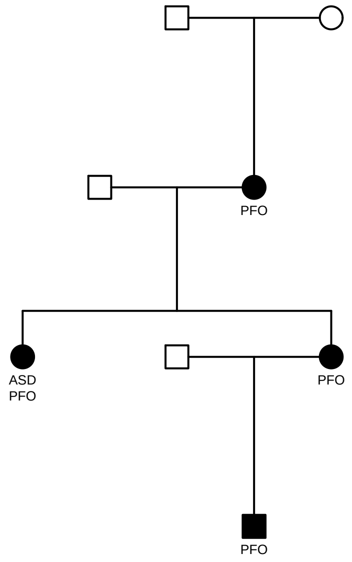

Wilmshurst_2004_3 PMID:15486131

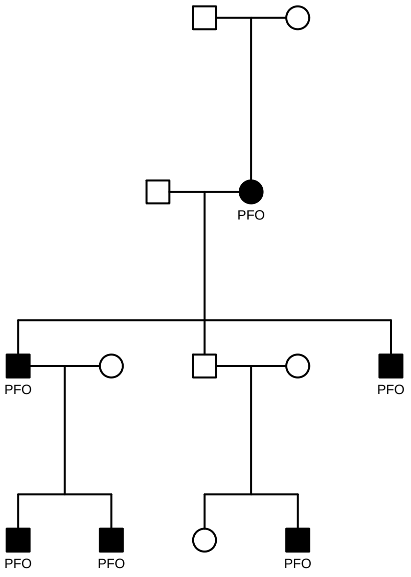

Wilmshurst_2004_4 PMID:15486131

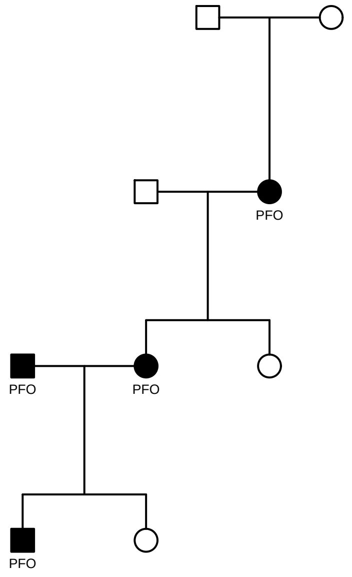

Wilmshurst_2004_5 PMID:15486131

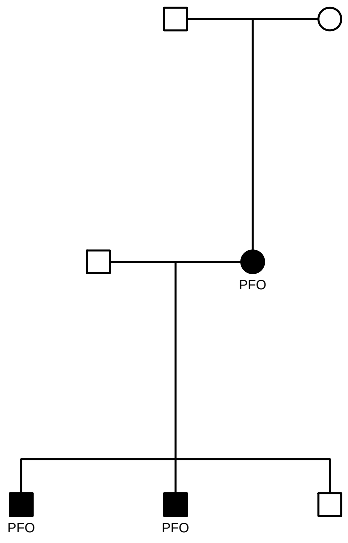

Wilmshurst_2004_6 PMID:15486131

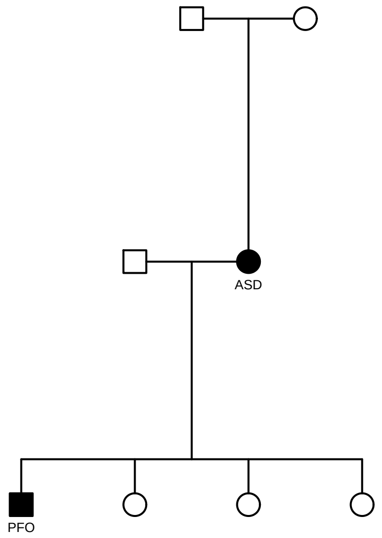

Wilmshurst_2009 PMID:15486131

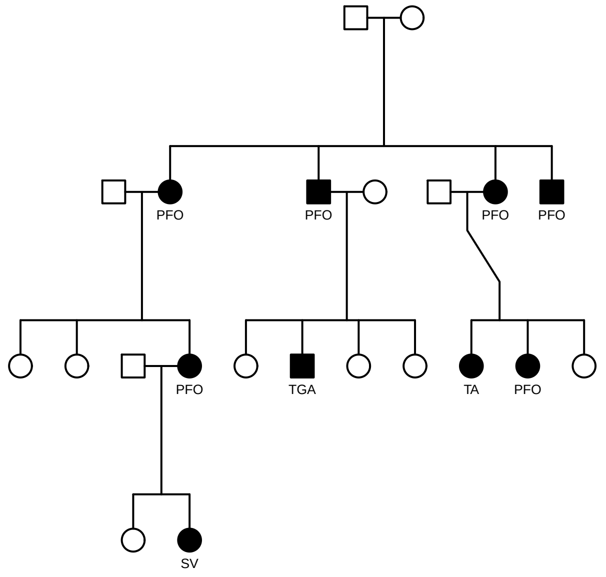

Chen_2010_1 PMID:20659440

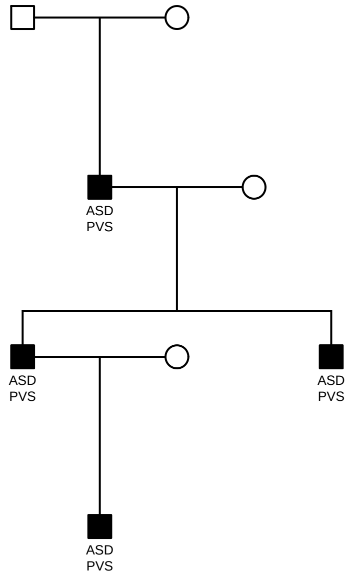

Benson_1998_A PMID:9610535

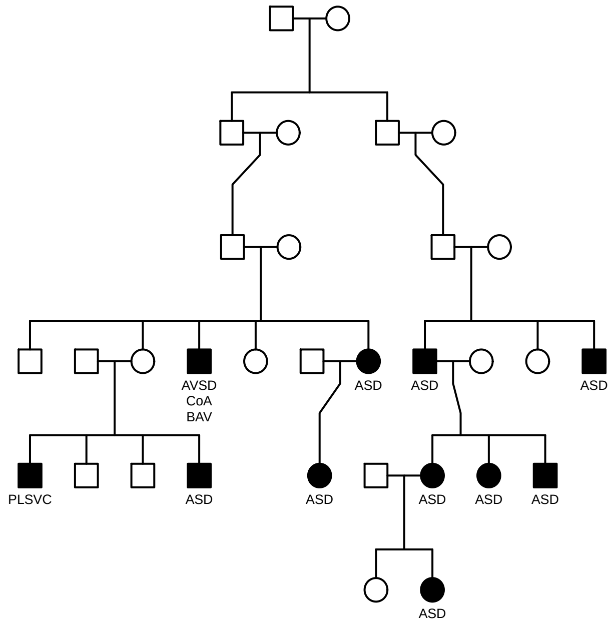

Benson_1998_B PMID:9610535

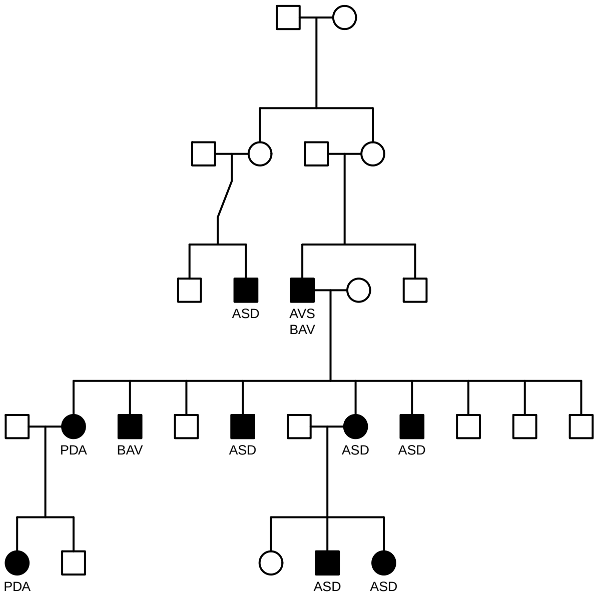

Benson_1998_C PMID:9610535

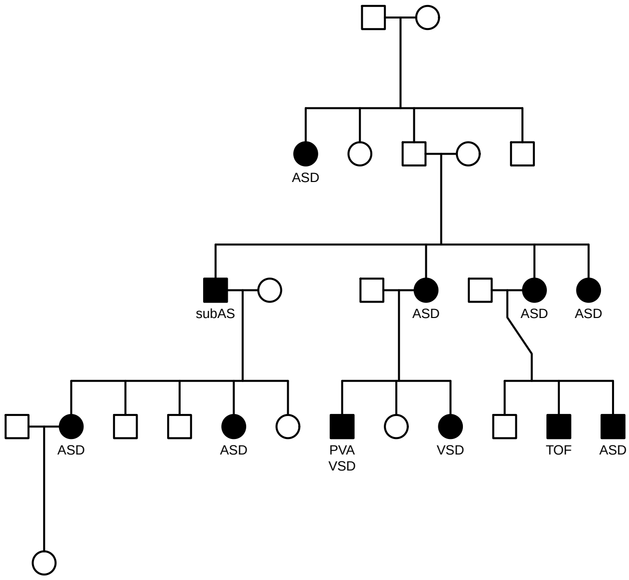

Emanuel_1975_A PMID:1191421

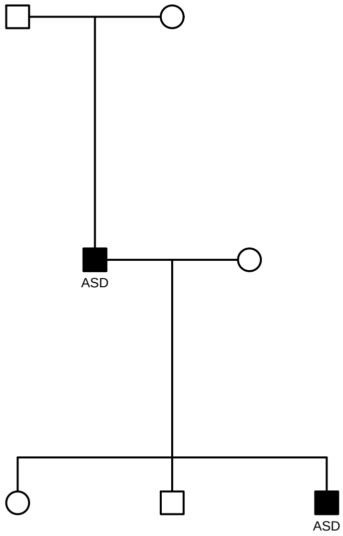

Emanuel_1975_B PMID:1191421

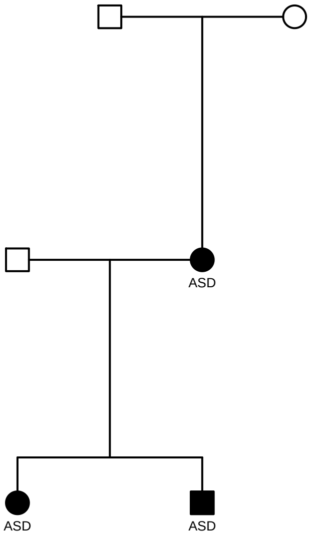

Emanuel_1975_C PMID:1191421

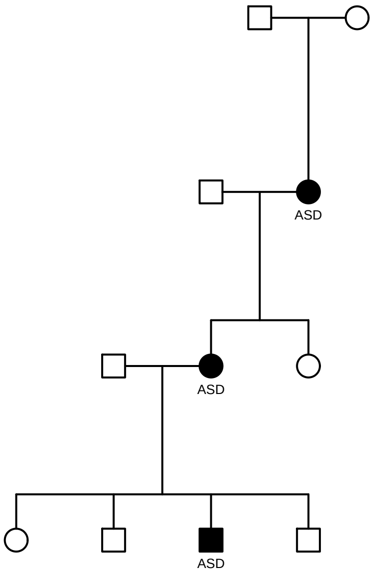

Hirayama−Yamada_1 PMID:15810002

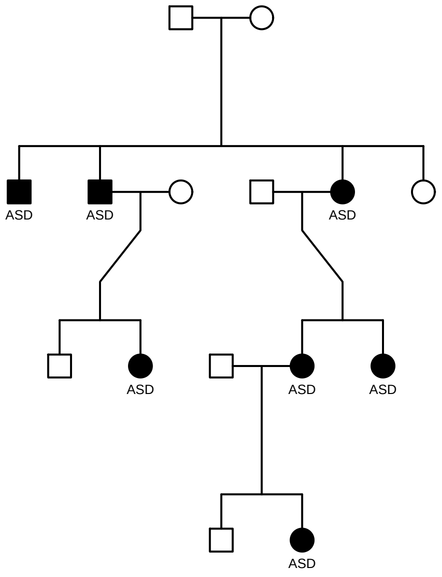

Hirayama−Yamada_2 PMID:15810002

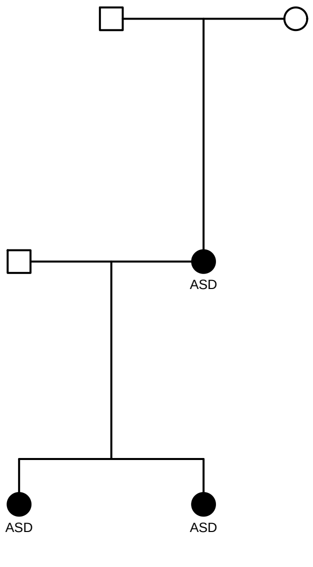

Hirayama−Yamada_3 PMID:15810002

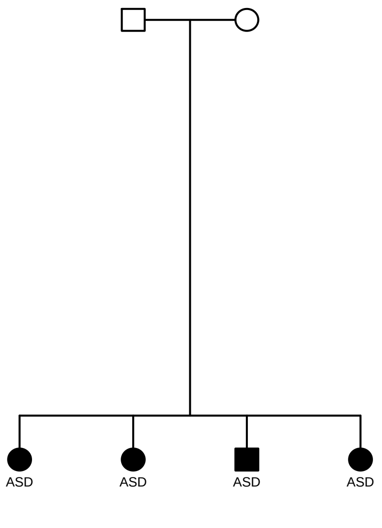

Hirayama−Yamada_4 PMID:15810002

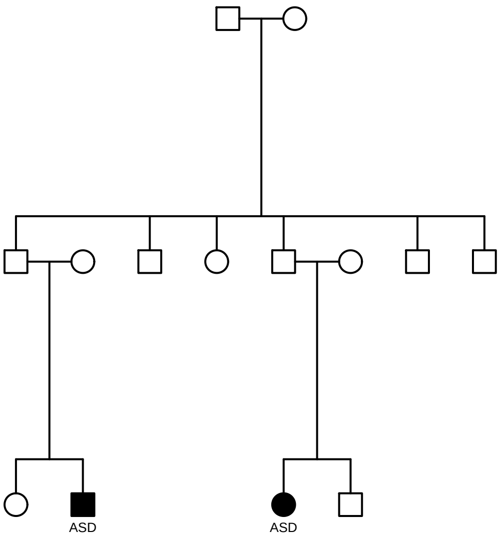

Hirayama−Yamada_5 PMID:15810002

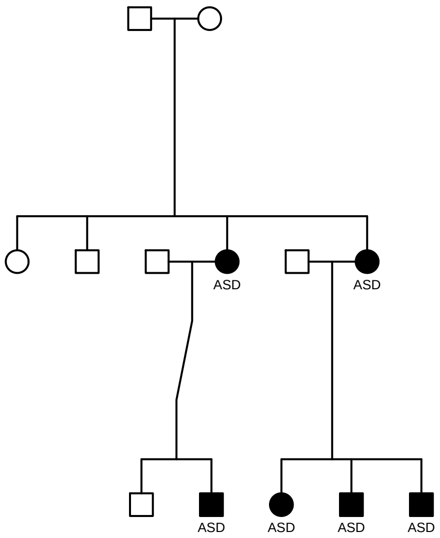

DiGiovanna_1999 PMID:10641493

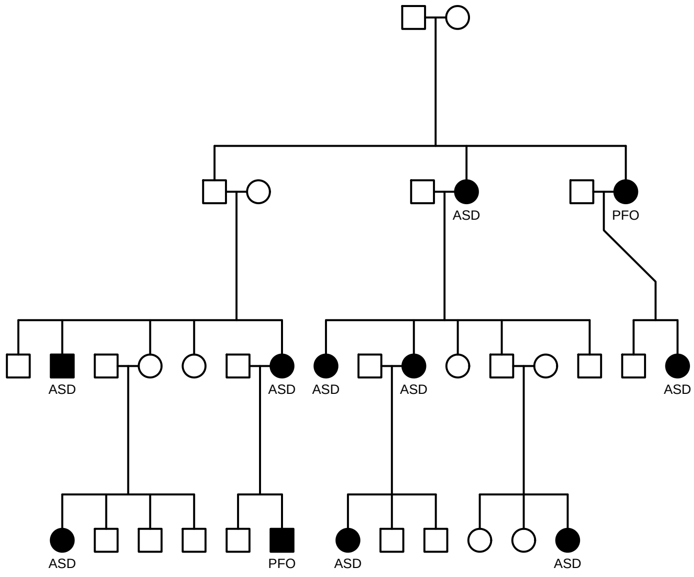

Elliott_1024 PMID:12798584

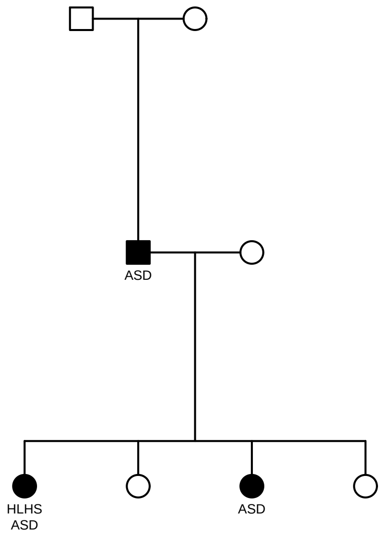

Elliott_AF1 PMID:12798584

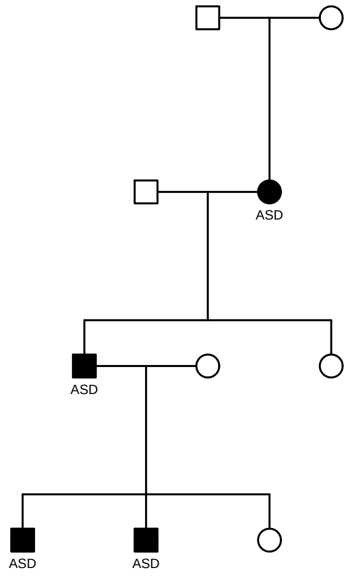

Chen_2010_2 PMID:20659440

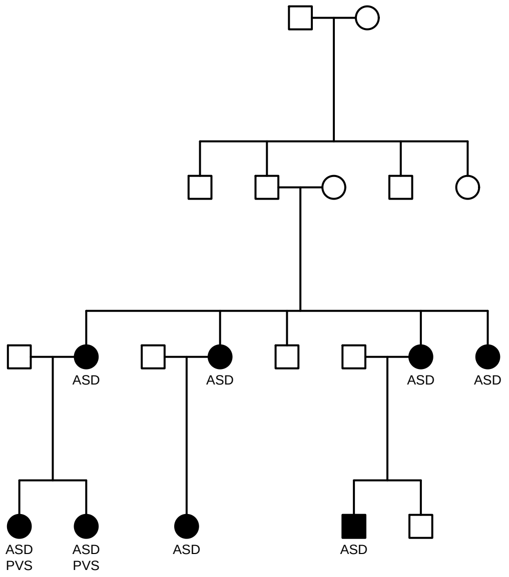

Ehlers_1 PMID:5922714

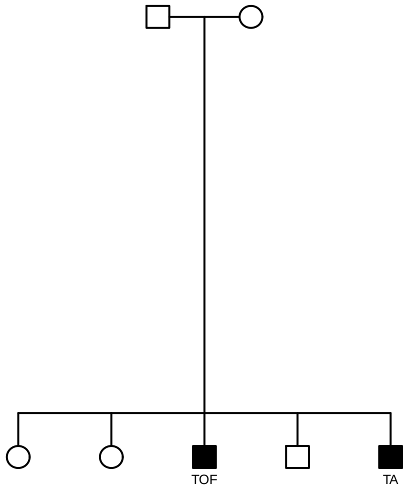

Ehlers_2 PMID:5922714

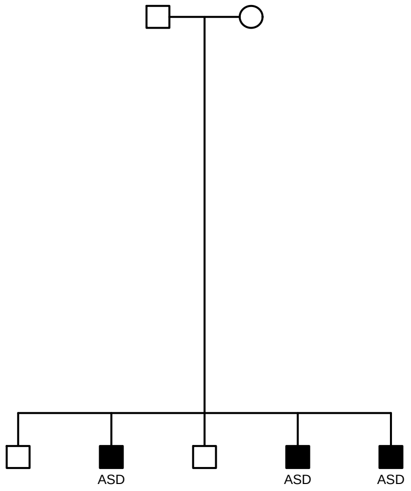

Ehlers_3 PMID:5922714

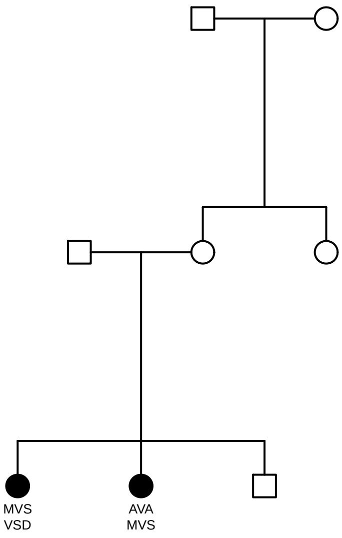

Ehlers_4 PMID:5922714

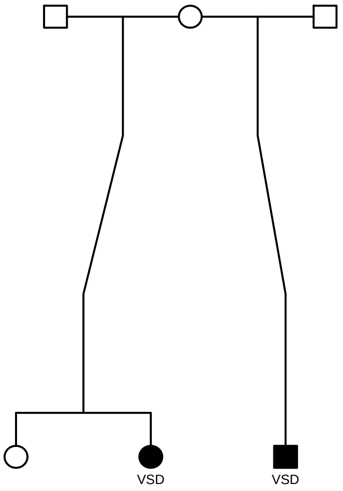

Ehlers_5 PMID:5922714

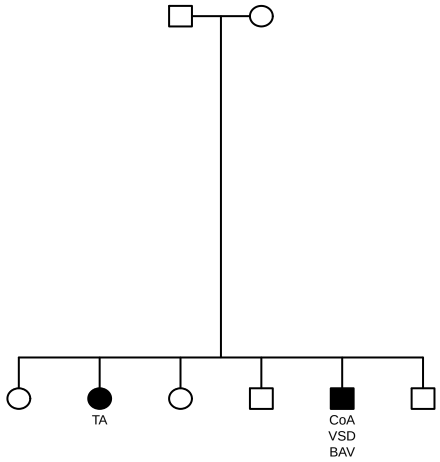

Ehlers_6 PMID:5922714

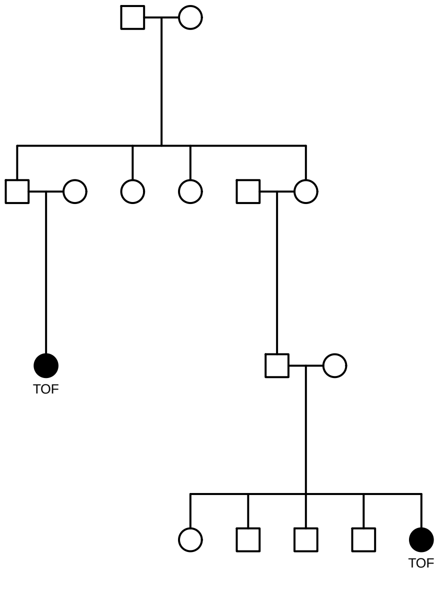

Ehlers_7 PMID:5922714

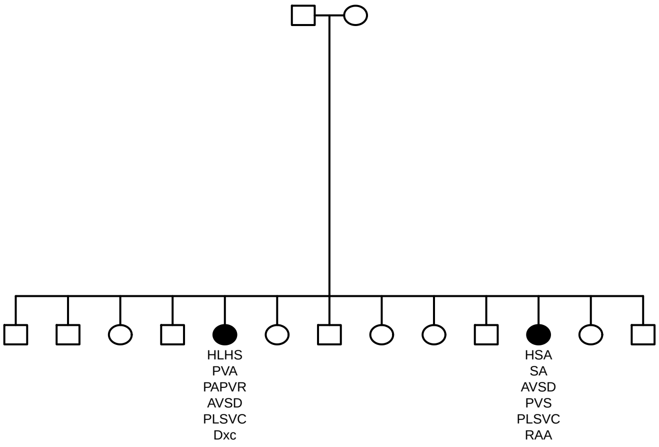

Ehlers_8 PMID:5922714

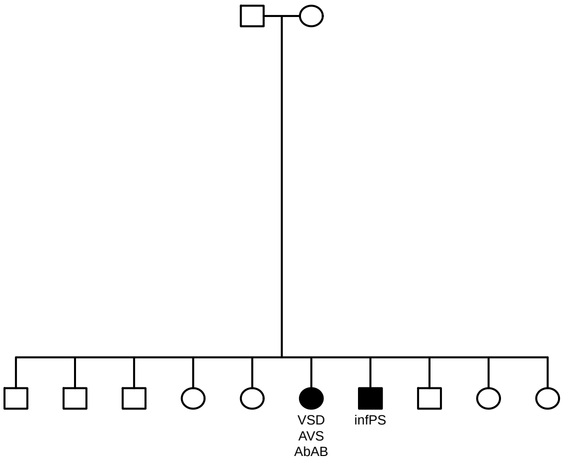

Ehlers_9 PMID:5922714

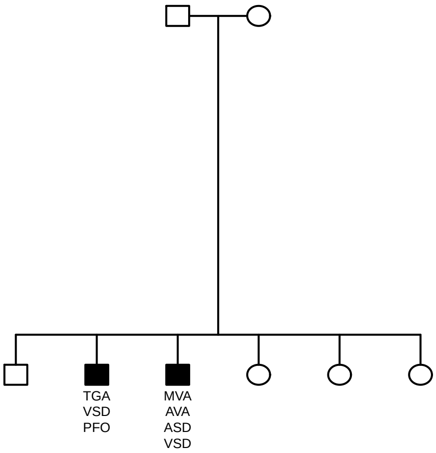

Ehlers_10 PMID:5922714

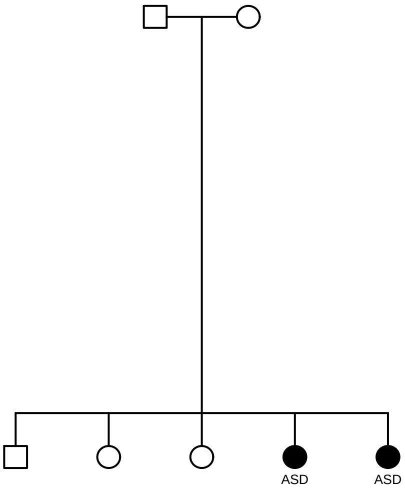

Ehlers_11 PMID:5922714

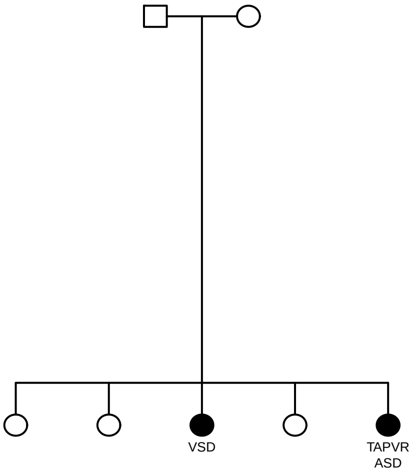

Ehlers_12 PMID:5922714

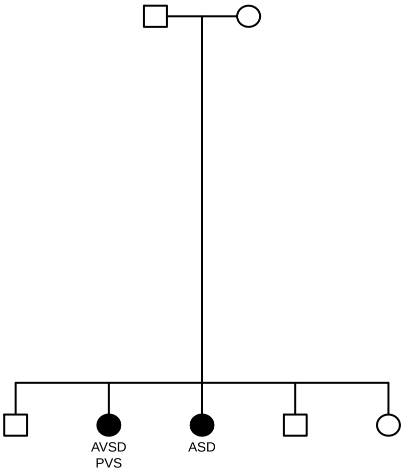

Ehlers_13 PMID:5922714

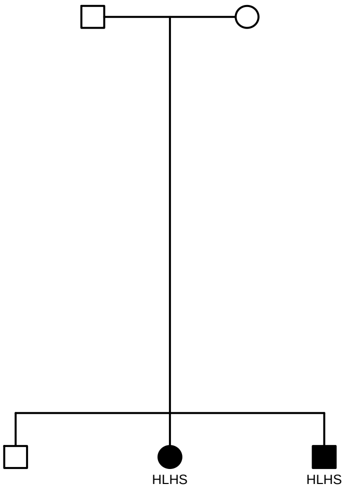

Ehlers_14 PMID:5922714

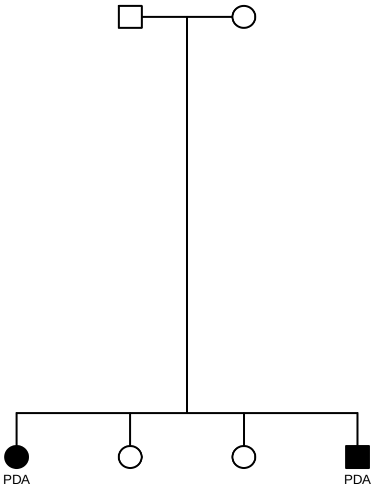

Ehlers_15 PMID:5922714

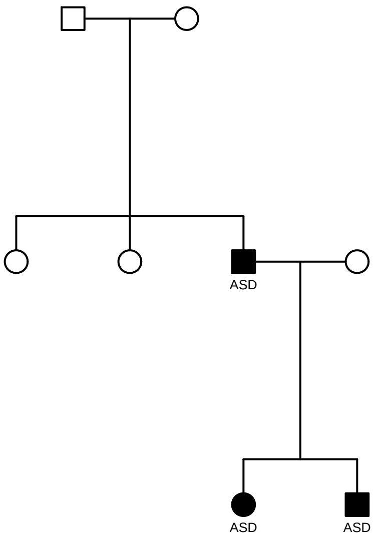

Ehlers_17 PMID:5922714

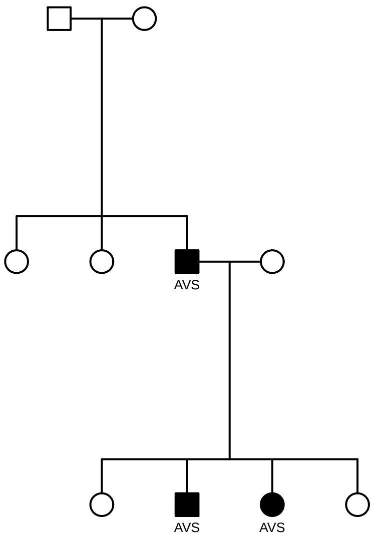

Ehlers_18 PMID:5922714

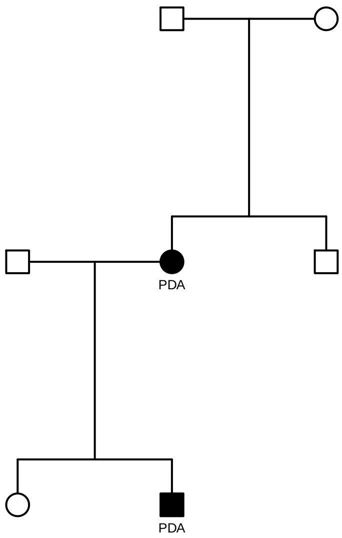

Ehlers_19 PMID:5922714

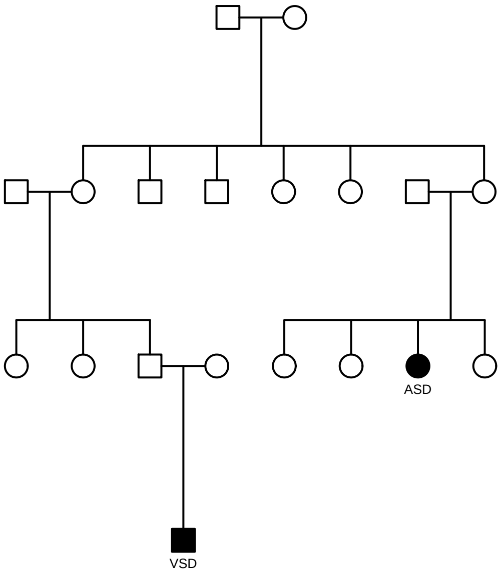

Ehlers_20 PMID:5922714

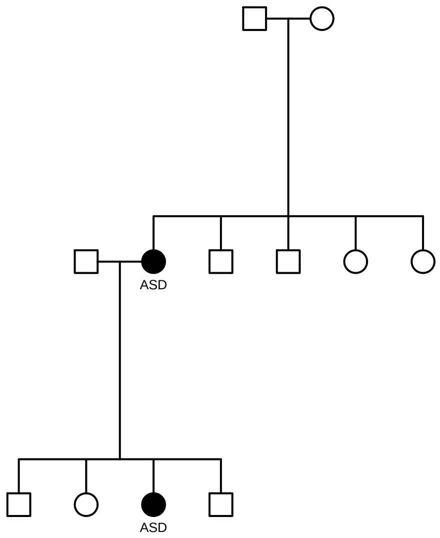

Ehlers_21 PMID:5922714

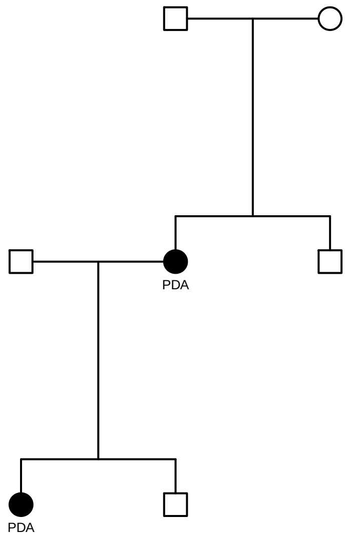

Ehlers_22 PMID:5922714

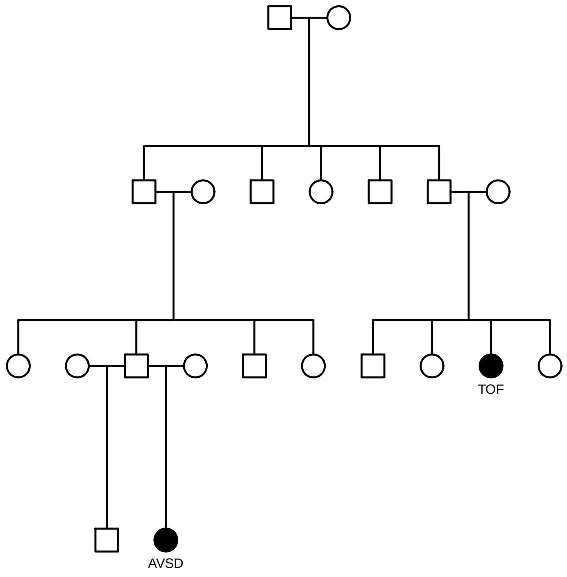

Ehlers_23 PMID:5922714

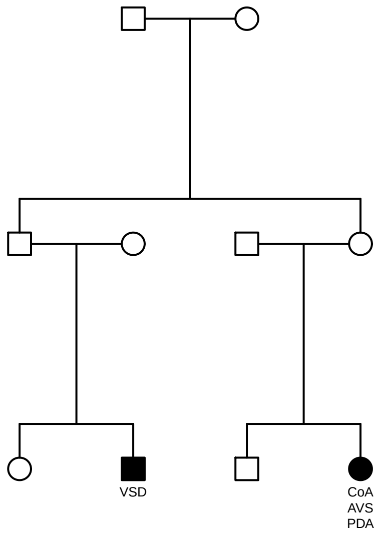

Ehlers_24 PMID:5922714

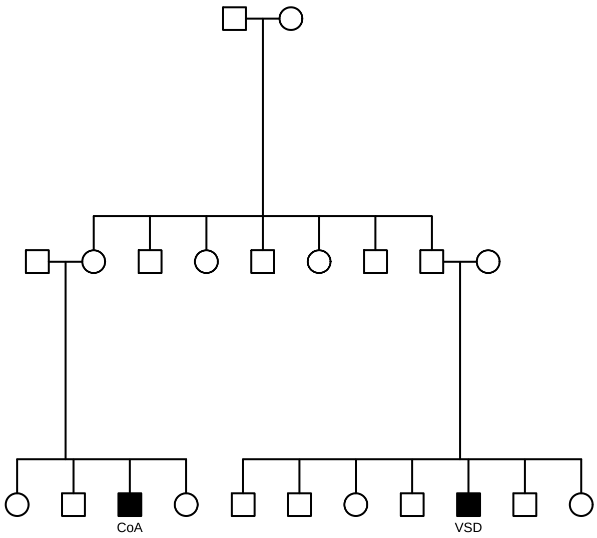

Ehlers_25 PMID:5922714

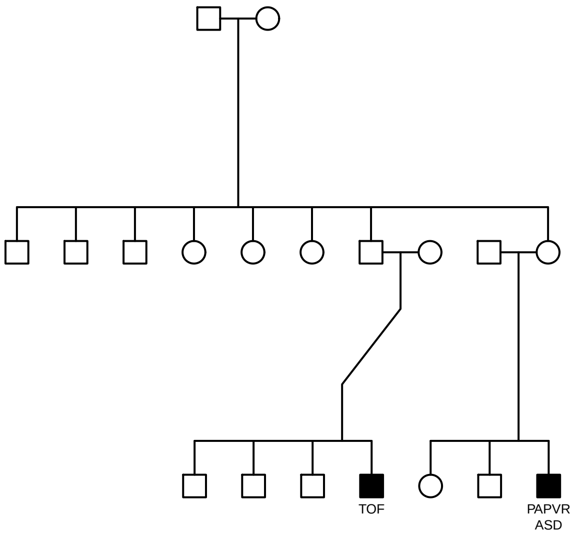

Ehlers_26 PMID:5922714

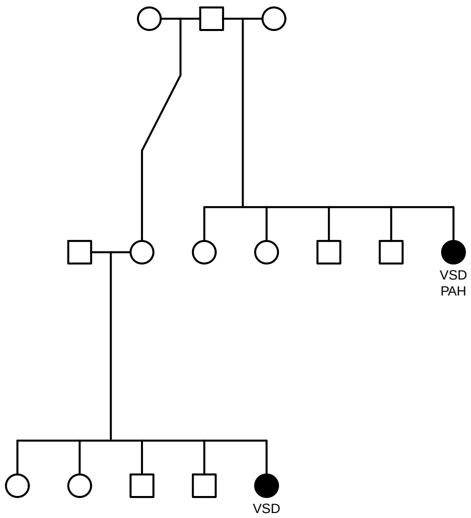

Ehlers_27 PMID:5922714

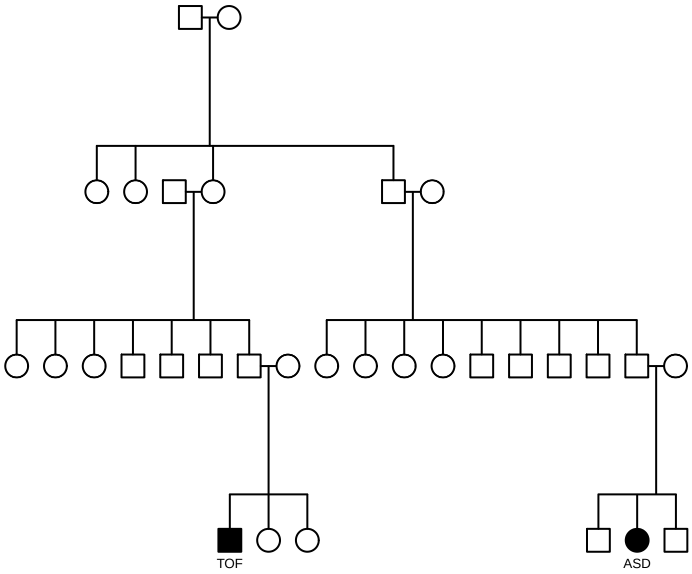

Ehlers_29 PMID:5922714

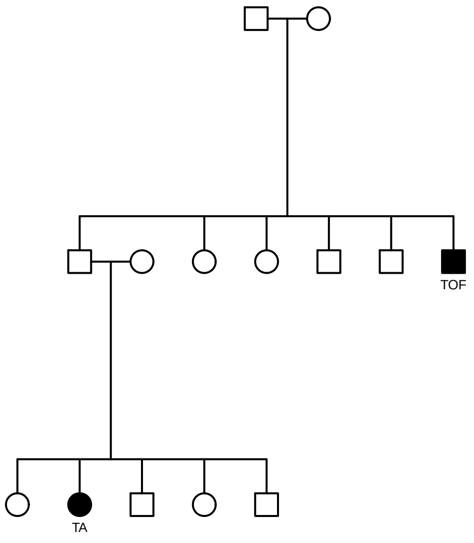

Ehlers_30 PMID:5922714

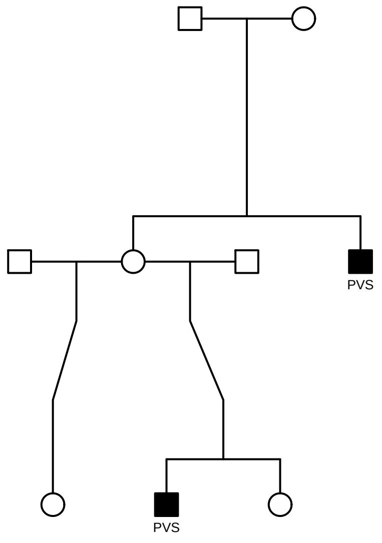

Ehlers_31 PMID:5922714

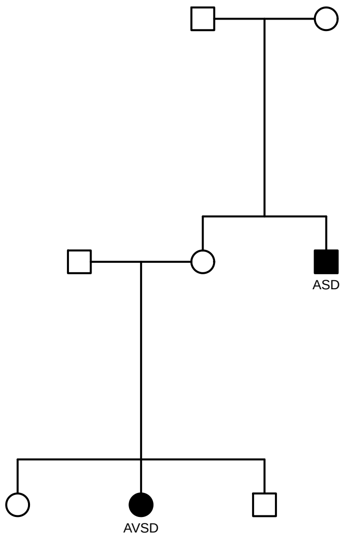

Ehlers_32 PMID:5922714

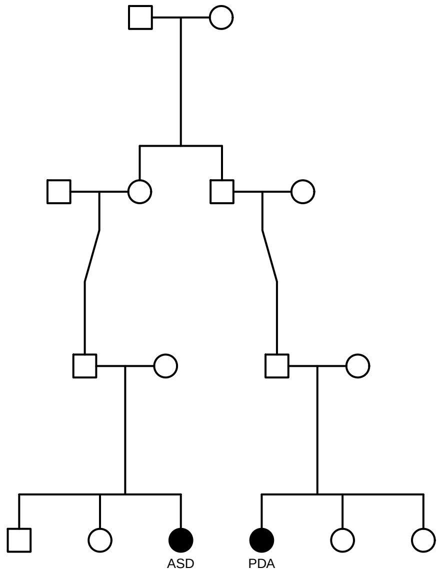

Miller_1 PMID:450526

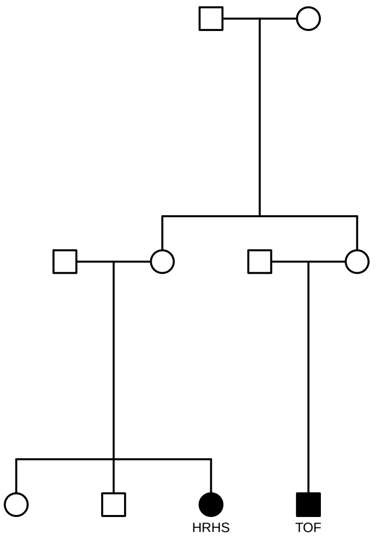

Miller_2 PMID:450526

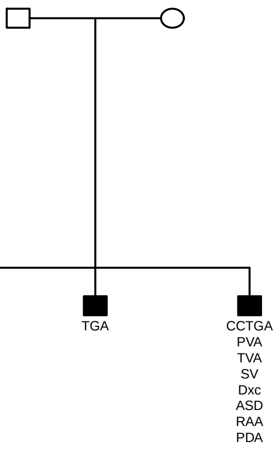

Wulfsberg_1991 PMID:1884513

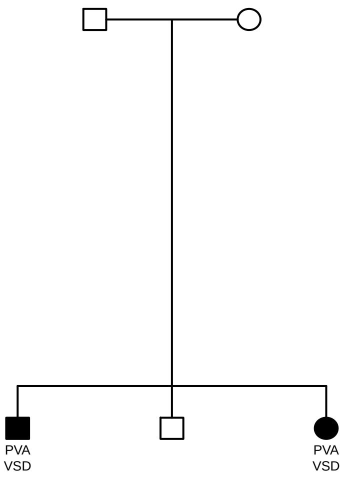

Wilson_1992_1 PMID:1355155

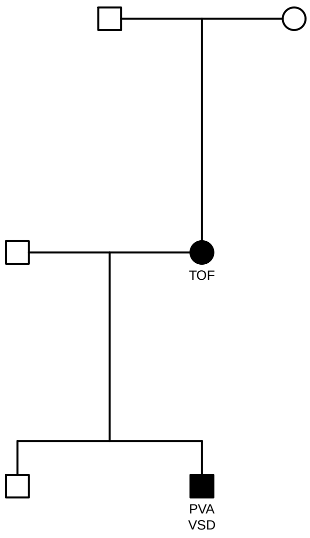

Wilson_1992_2 PMID:1355155

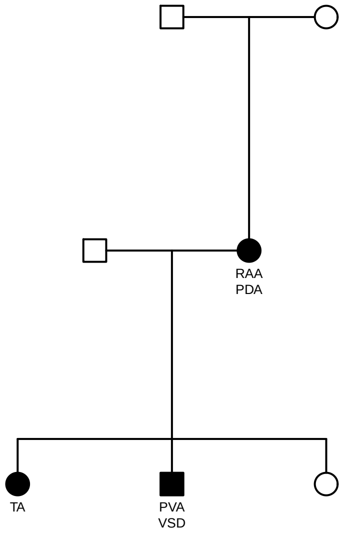

Wilson_1992_4 PMID:1355155

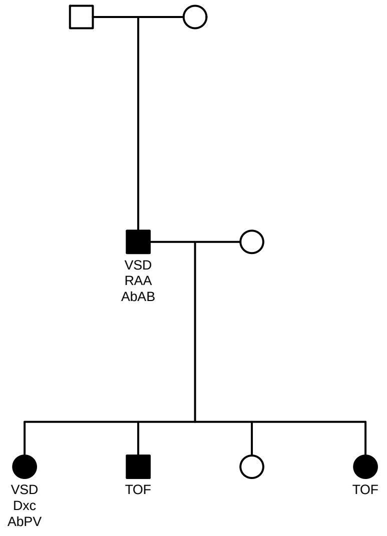

Wilson_1992_5 PMID:1355155

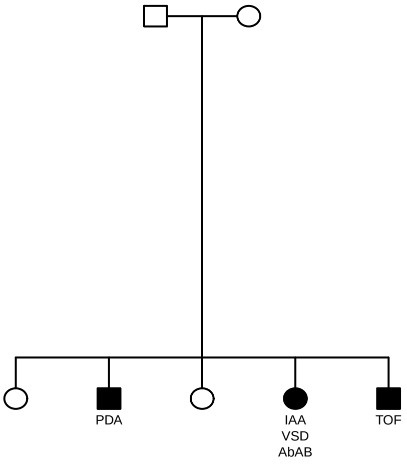

Eldadah_2001 PMID:11152664

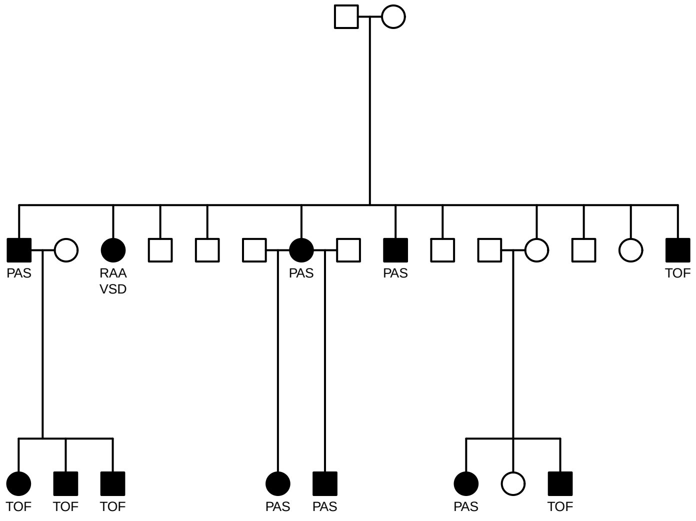

Zellers_1990_1 PMID:2305694

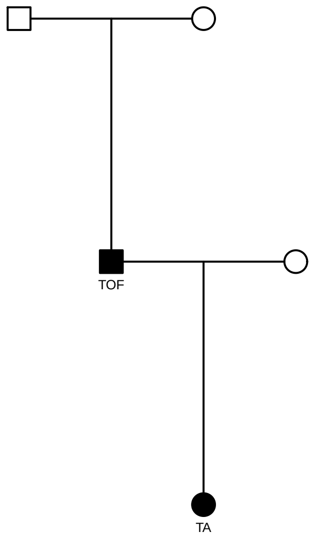

Zellers_1990_2 PMID:2305694

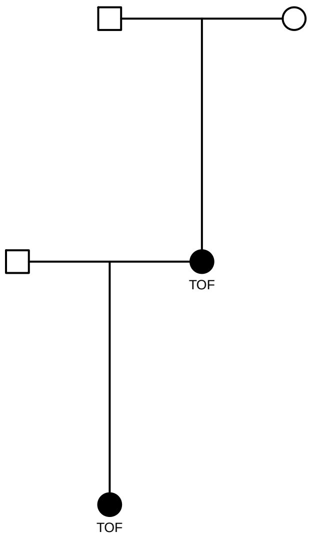

Adams_1974 PMID:15215987

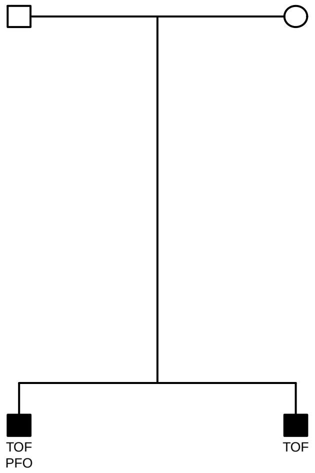

Boon_1972_1 PMID:5065286

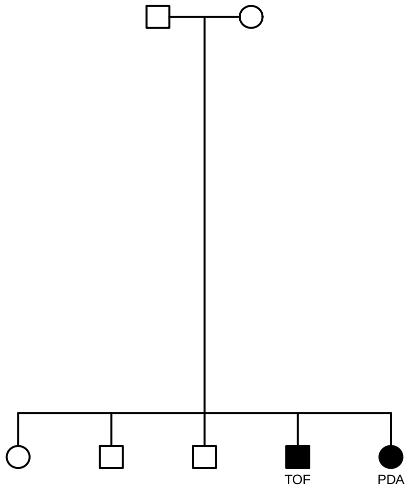

Digilio_1997_1_a PMID:9132487

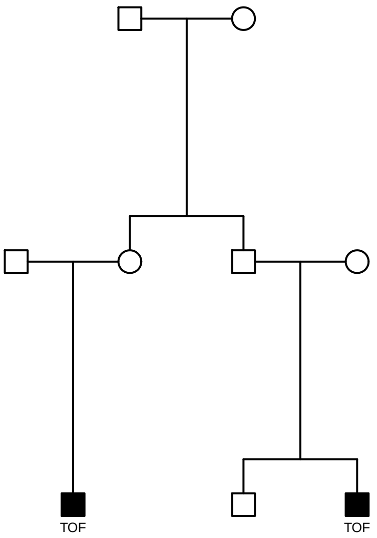

Digilio_1997_1_b PMID:9132487

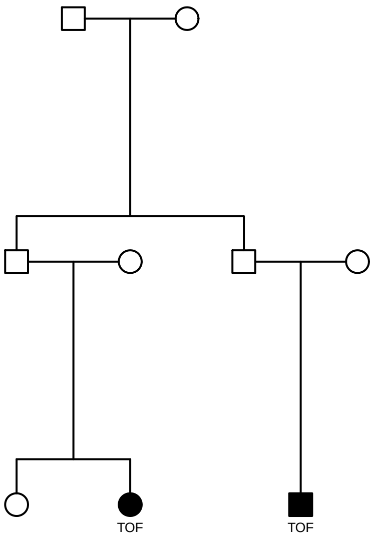

Digilio_1997_1_c PMID:9132487

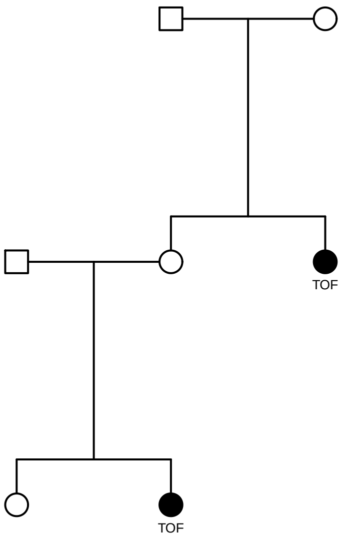

Digilio_1997_1_d PMID:9132487

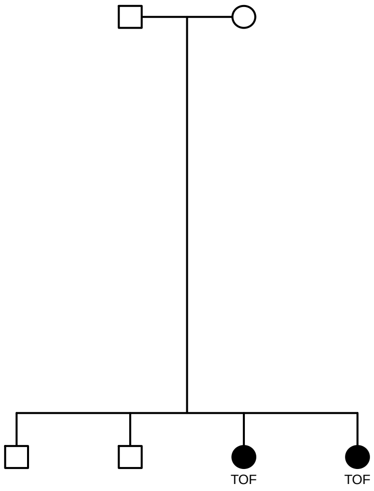

Digilio_1997_1_e PMID:9132487

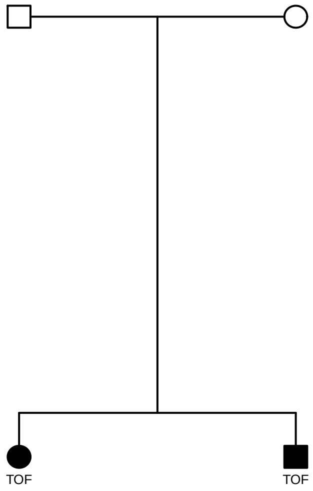

Digilio_1997_2_a PMID:9132487

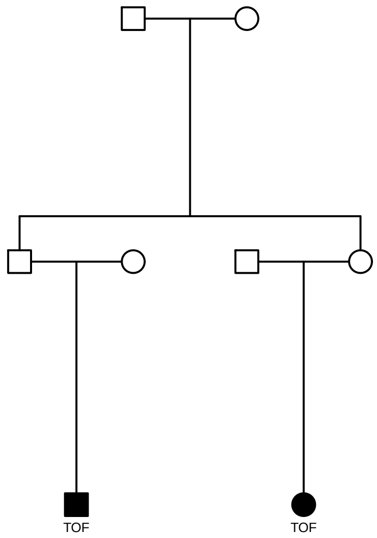

Digilio_1997_2_b PMID:9132487

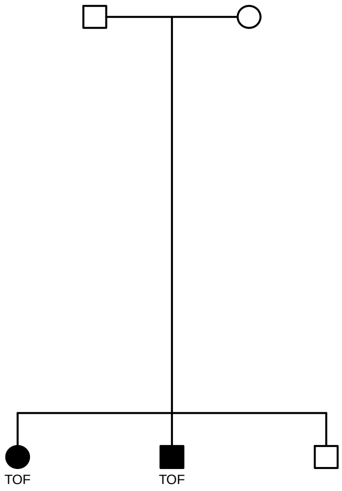

Digilio_1997_2_c PMID:9132487

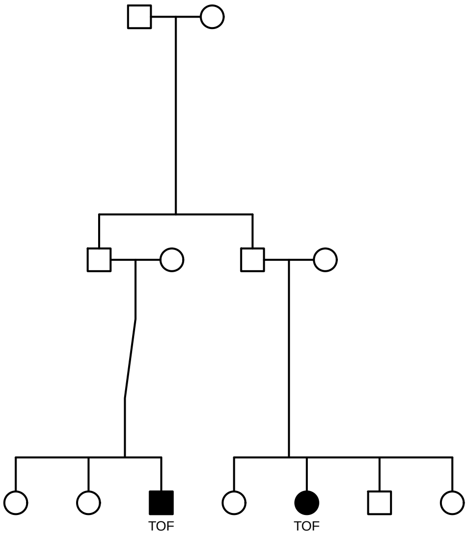

Debrus_1996_A PMID:8566942

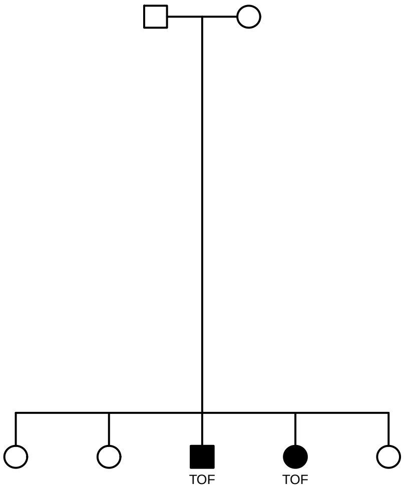

Debrus_1996_C PMID:8566942

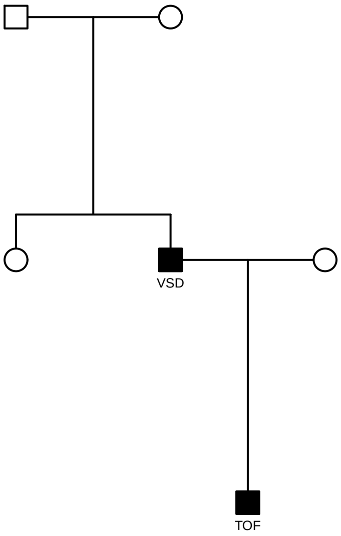

Debrus_1996_D PMID:8566942

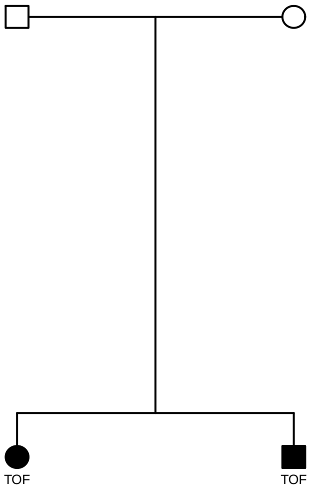

Debrus_1996_E PMID:8566942

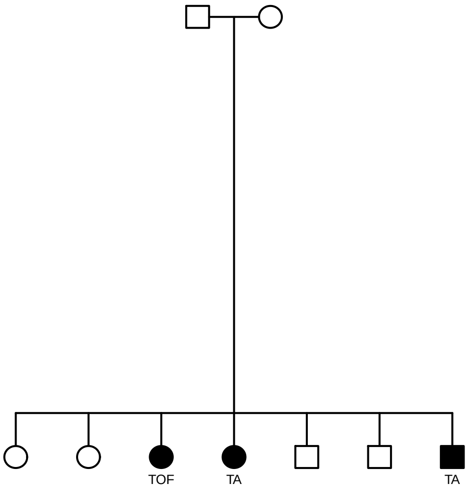

Debrus_1996_F PMID:8566942

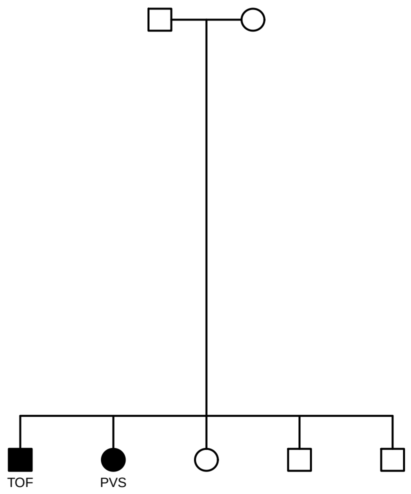

Debrus_1996_G PMID:8566942

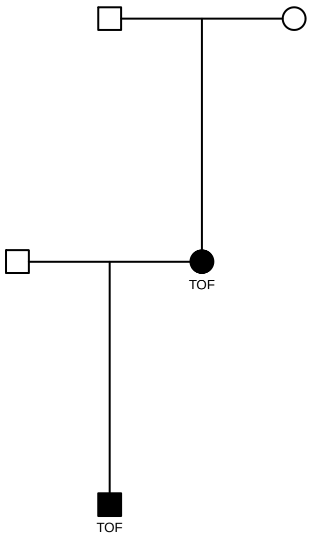

Debrus_1996_H PMID:8566942

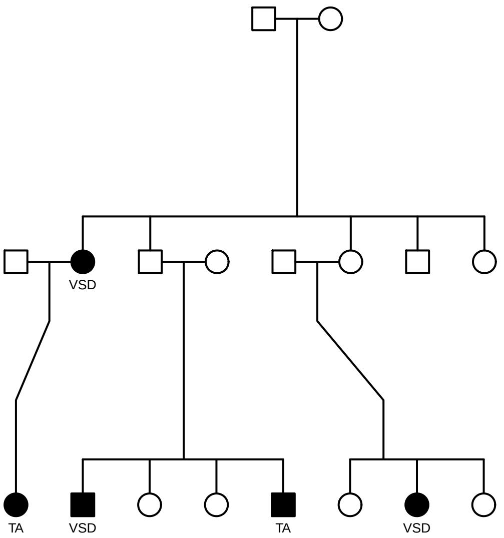

Debrus_1996_I PMID:8566942

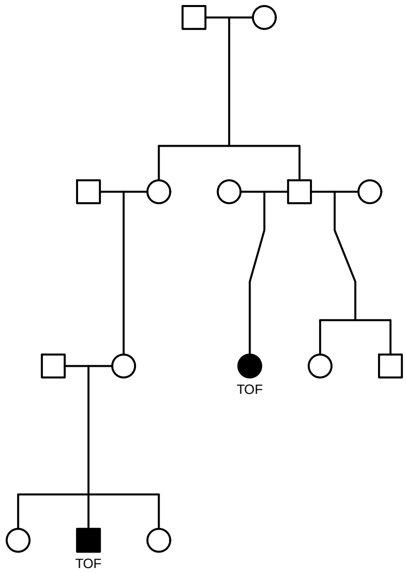

Debrus_1996_J PMID:8566942

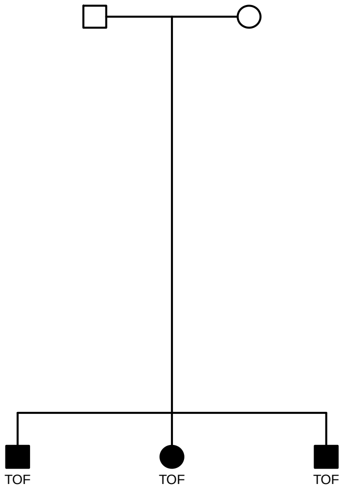

Debrus_1996_K PMID:8566942

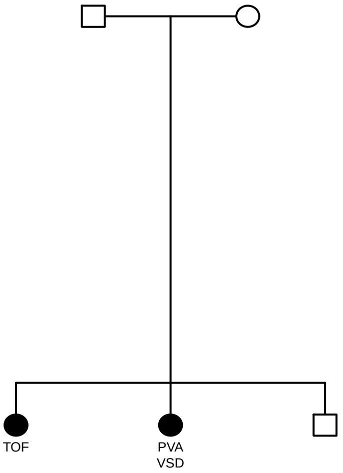

Debrus_1996_L PMID:8566942

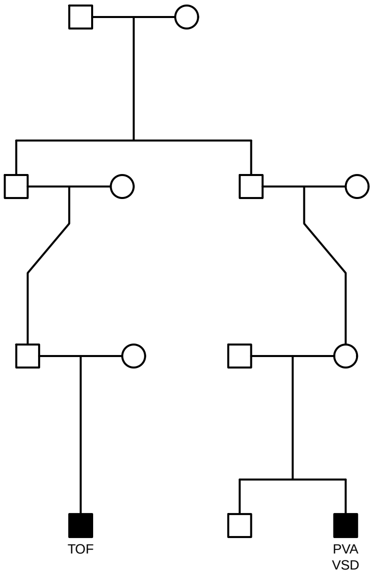

Debrus_1996_M PMID:8566942

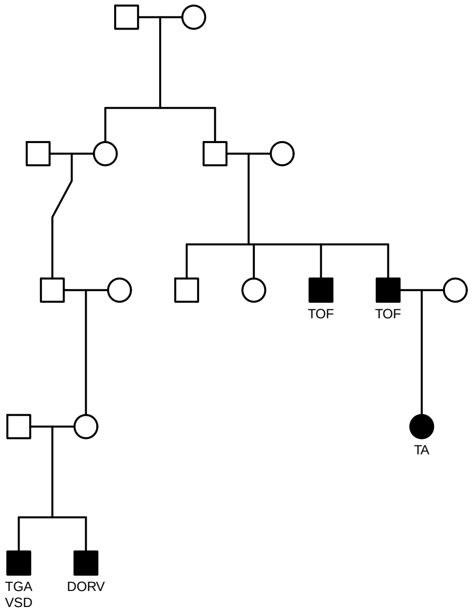

Debrus_1996_N PMID:8566942

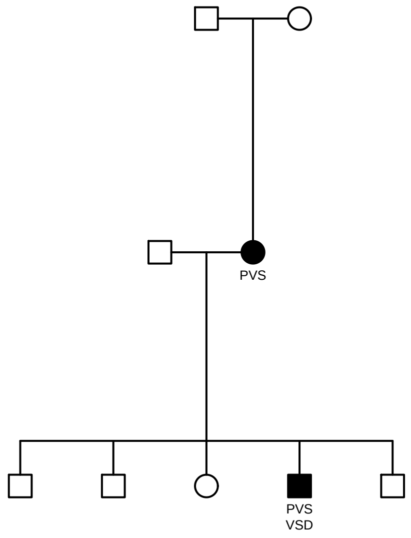

Debrus_1996_O PMID:8566942

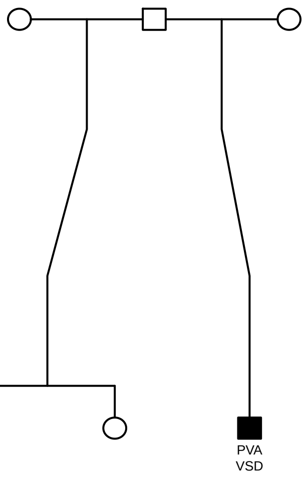

Debrus_1996_P PMID:8566942

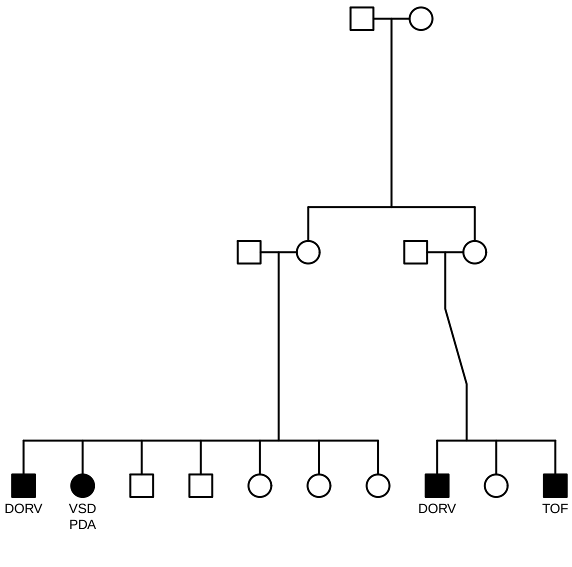

Der_Kaloustian_1985 PMID:4003436

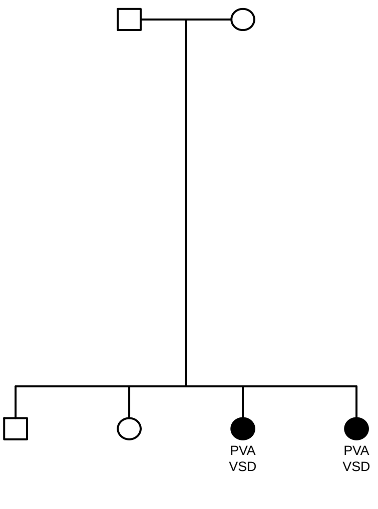

Cassidy_1991 PMID:2042580

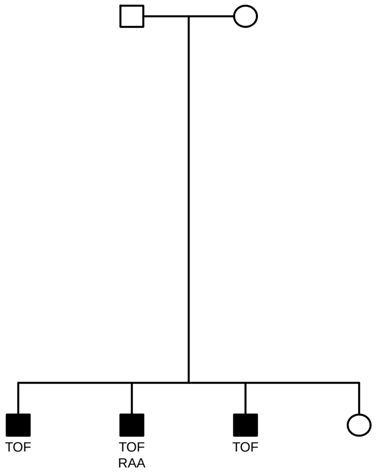

Silver_1972 PMID:5035578

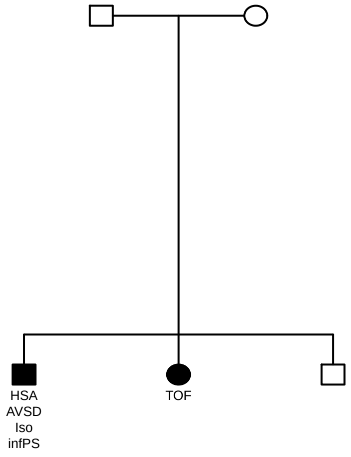

de_la_Chapelle_1981 PMID:7250965

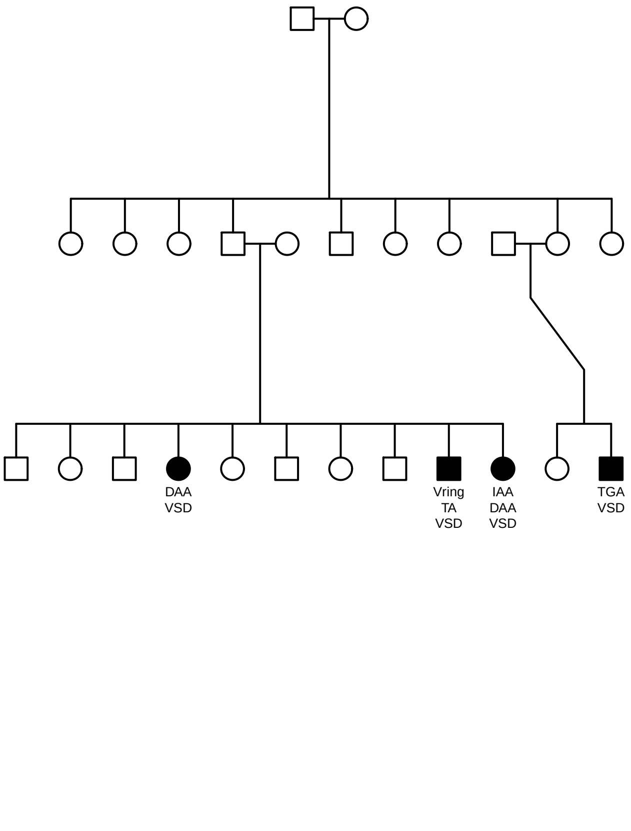

Rohn_1984 PMID:6737148

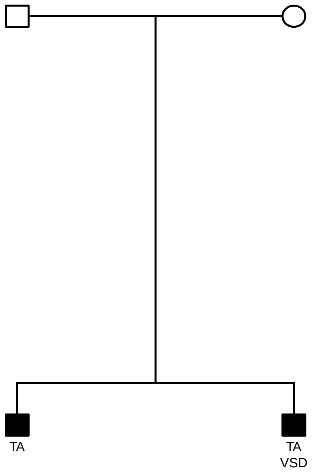

Wilson_1991 PMID:1747284

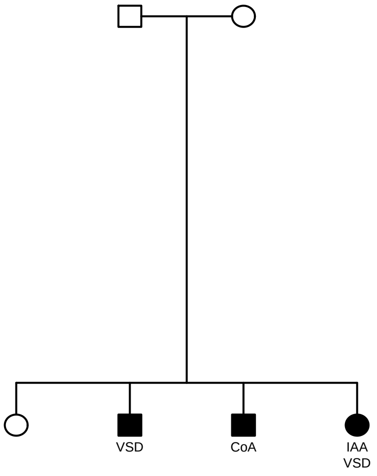

Raatikka_1981 PMID:7243440

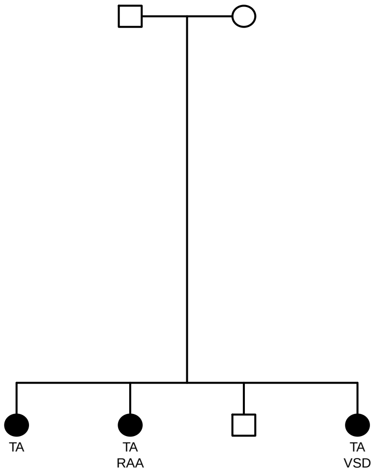

Fuhrmann_1 PMID:5699890

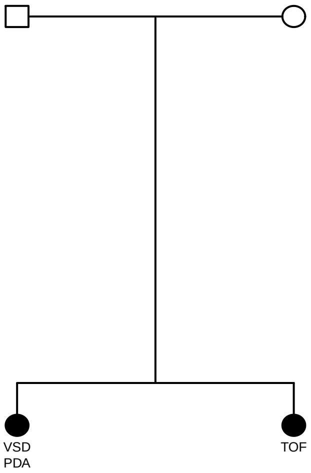

Fuhrmann_2 PMID:5699890

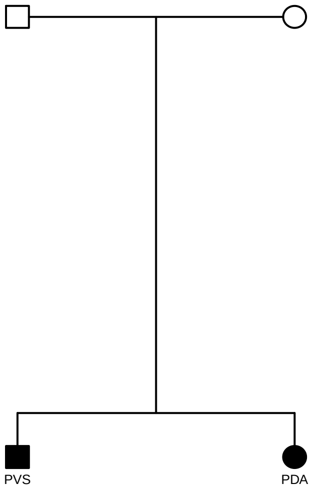

Fuhrmann_3 PMID:5699890

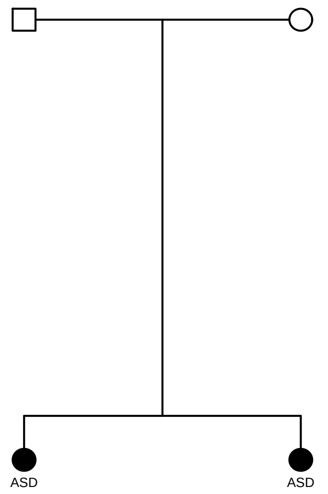

Fuhrmann_5 PMID:5699890

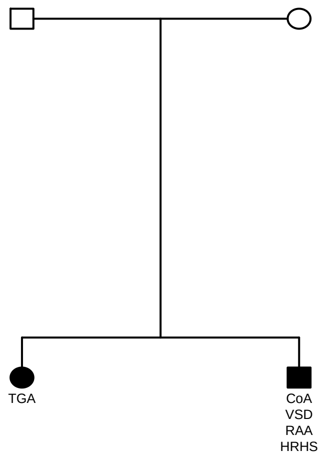

Fuhrmann_6 PMID:5699890

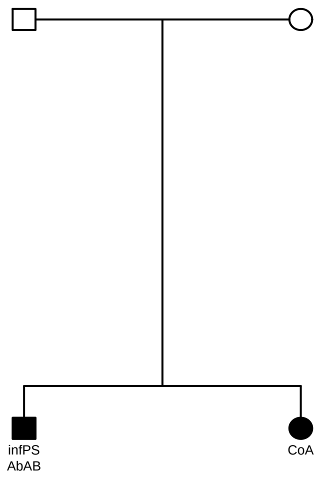

Fuhrmann_7 PMID:5699890

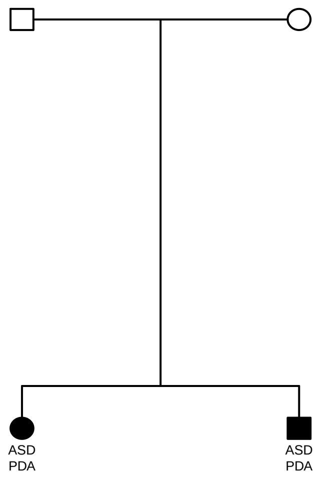

Fuhrmann_9 PMID:5699890

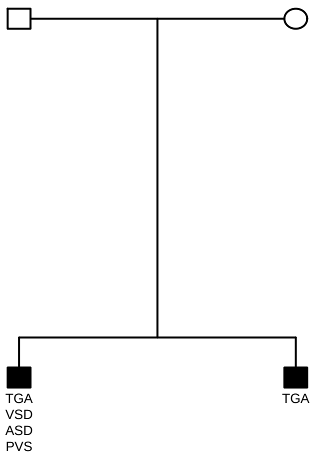

Fuhrmann_11 PMID:5699890

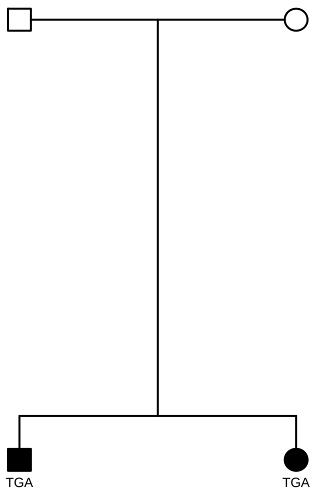

Fuhrmann_12 PMID:5699890

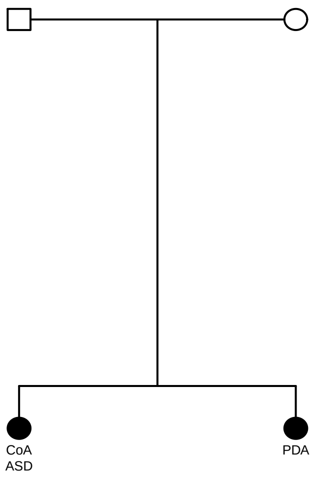

Fuhrmann_13 PMID:5699890

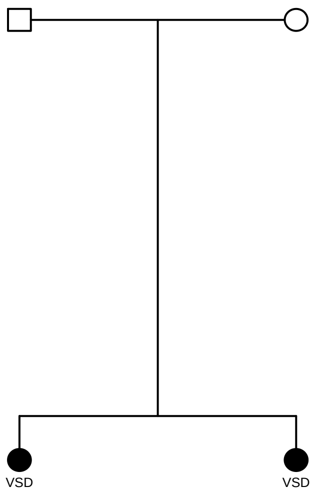

Fuhrmann_17 PMID:5699890

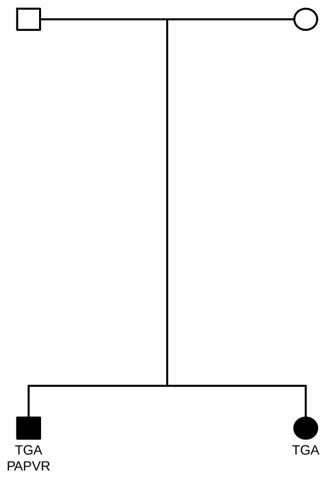

Fuhrmann_20 PMID:5699890

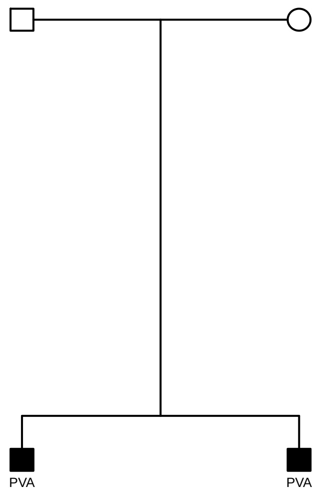

Fuhrmann_21 PMID:5699890

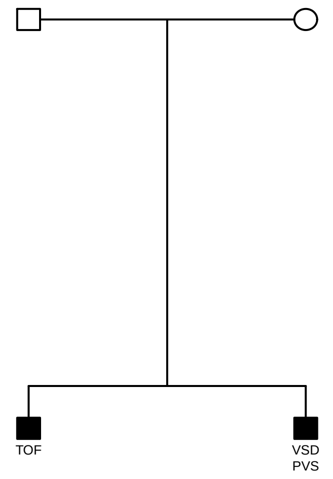

Fuhrmann_22 PMID:5699890

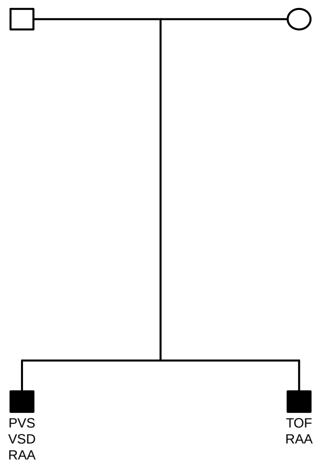

Fuhrmann_25 PMID:5699890

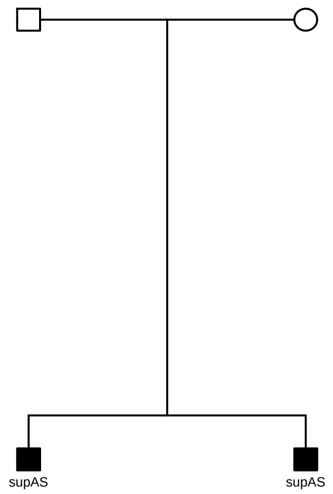

Fuhrmann_27 PMID:5699890

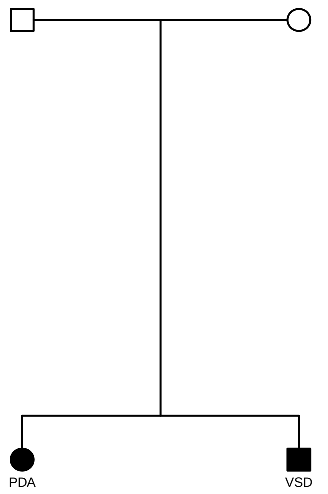

Fuhrmann_28 PMID:5699890

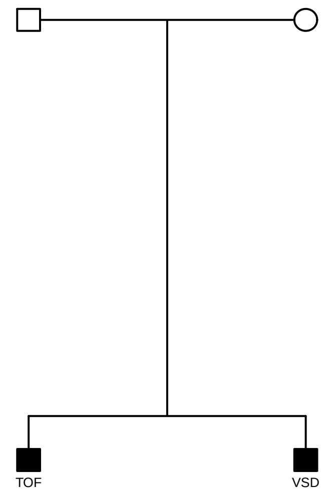

Fuhrmann_31 PMID:5699890

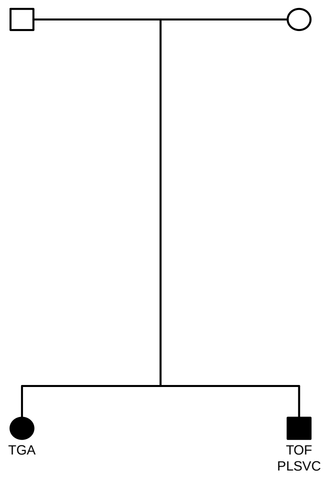

Fuhrmann_33 PMID:5699890

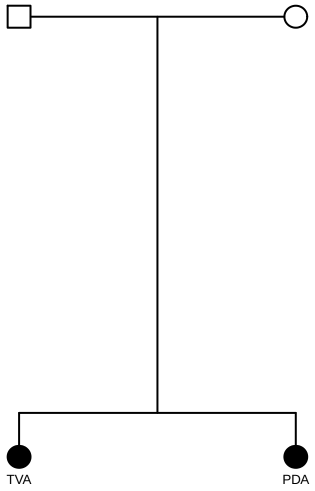

Fuhrmann_34 PMID:5699890

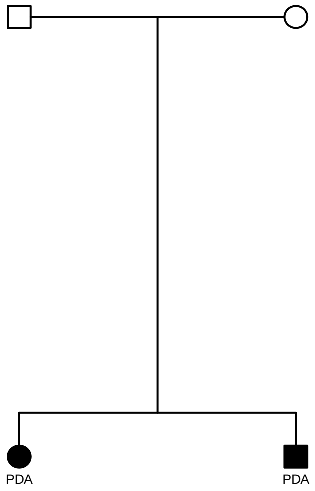

Fuhrmann_35 PMID:5699890

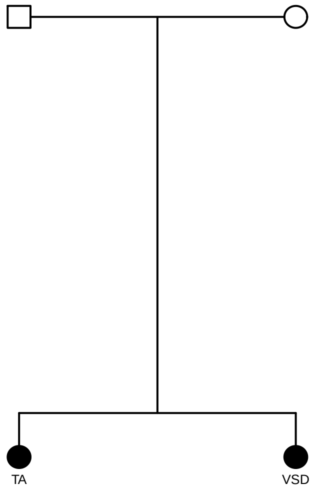

Fuhrmann_36 PMID:5699890

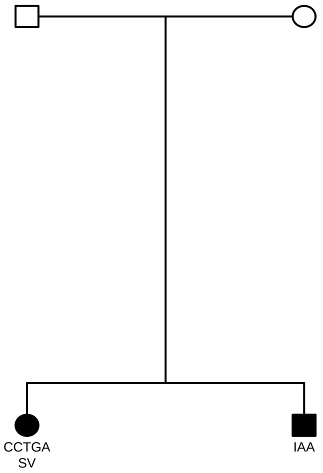

Fuhrmann_41 PMID:5699890

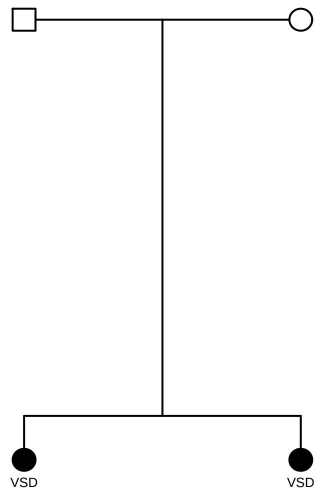

Fuhrmann_42 PMID:5699890

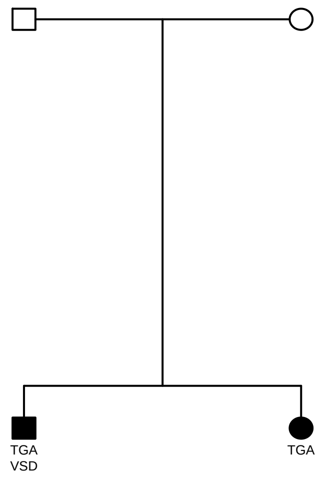

Fuhrmann_44 PMID:5699890

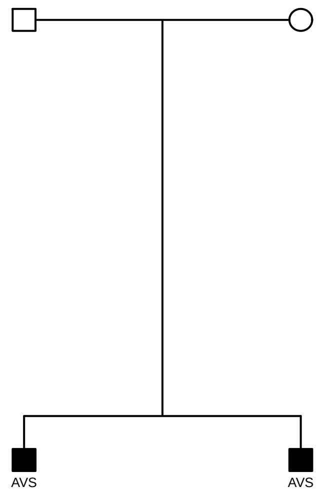

Fuhrmann_45 PMID:5699890

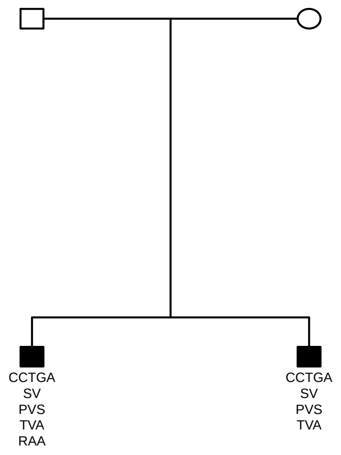

Fuhrmann_46 PMID:5699890

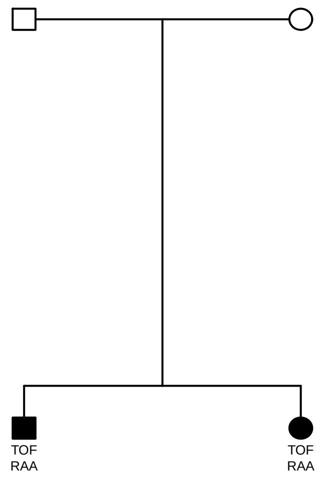

Fuhrmann_47 PMID:5699890

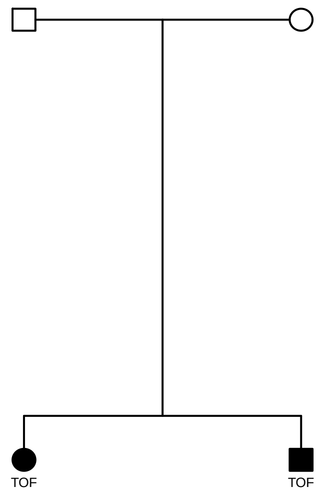

Fuhrmann_49 PMID:5699890

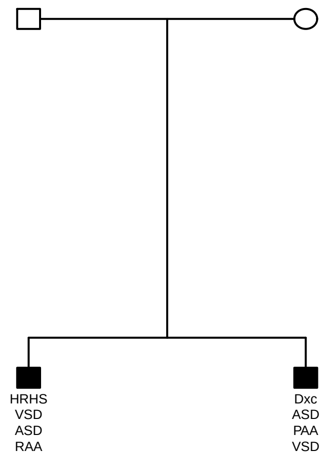

Fuhrmann_52 PMID:5699890

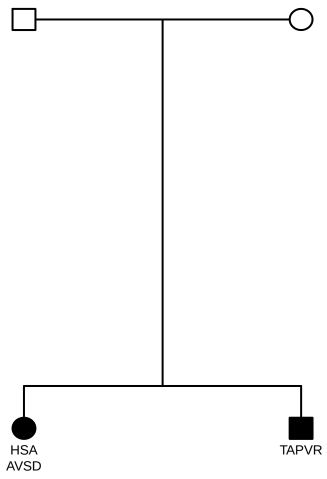

Fuhrmann_53 PMID:5699890

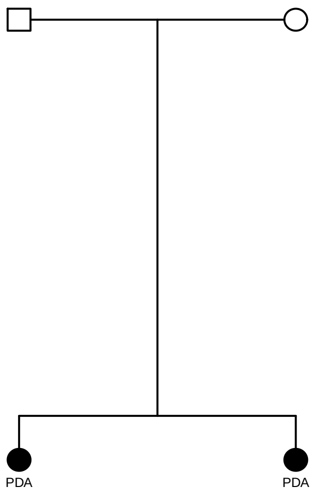

Fuhrmann_58 PMID:5699890

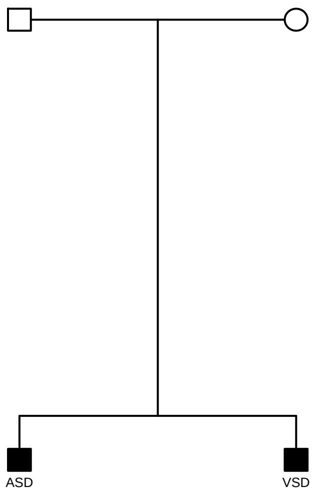

Fuhrmann_59 PMID:5699890

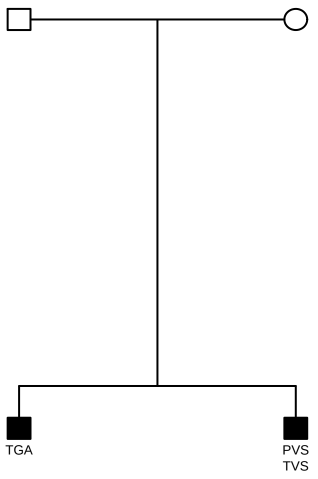

Fuhrmann_61 PMID:5699890

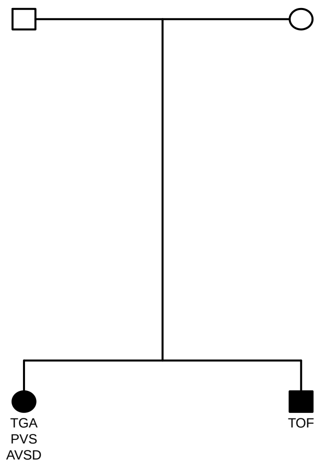

Fuhrmann_63 PMID:5699890

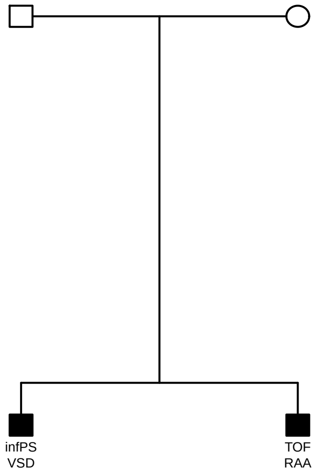

Fuhrmann_64 PMID:5699890

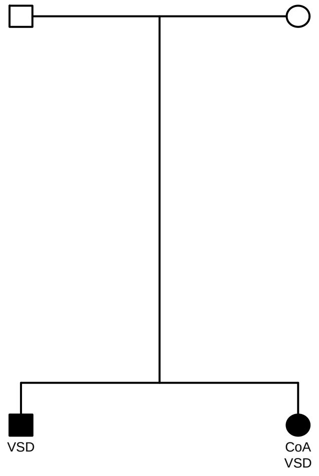

Fuhrmann_72 PMID:5699890

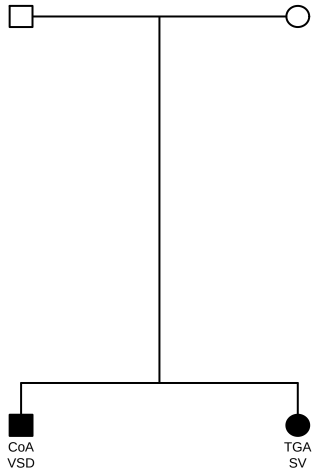

Fuhrmann_74 PMID:5699890

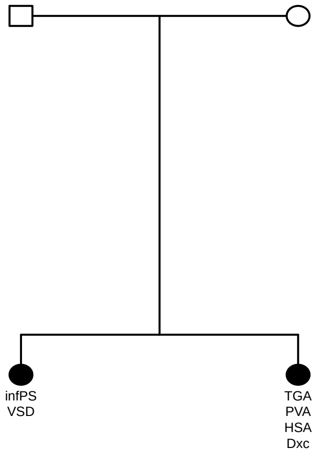

Fuhrmann_77 PMID:5699890

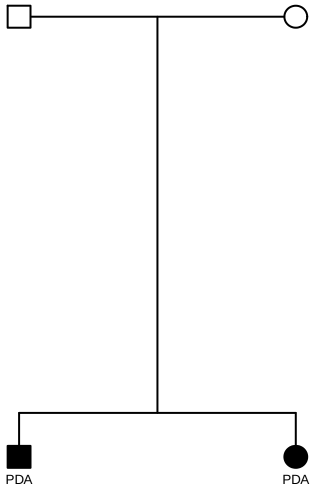

Fuhrmann_78 PMID:5699890

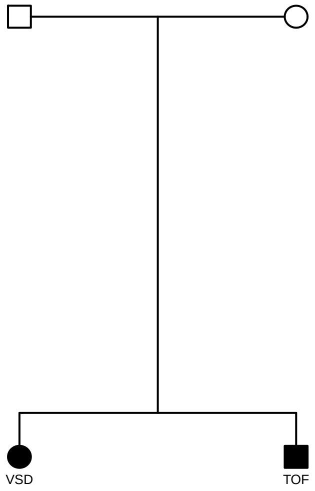

Fuhrmann_a PMID:5699890

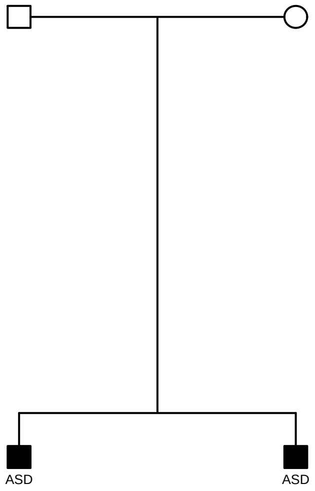

Fuhrmann_b PMID:5699890

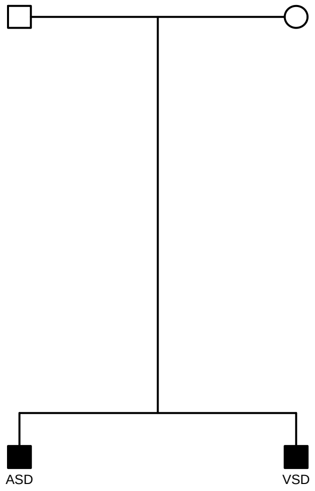

Lynch_1966 PMID:5904472

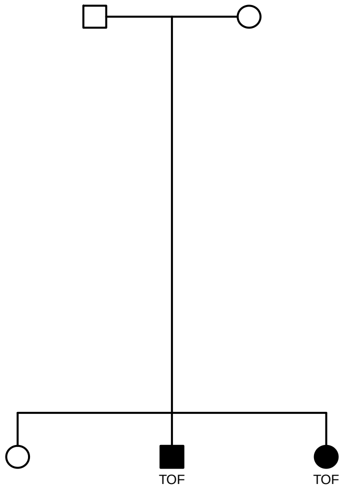

Thammineni_2011_1 PMID:22037157

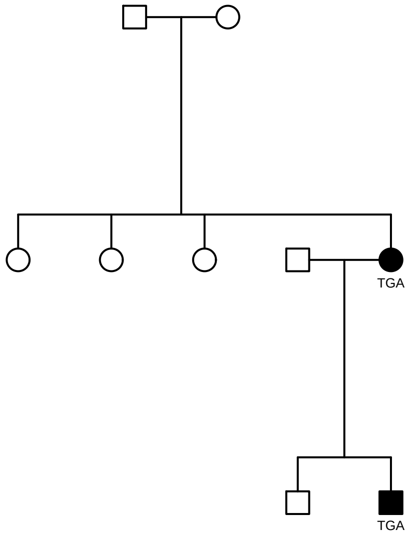

Thammineni_2011_2 PMID:22037157

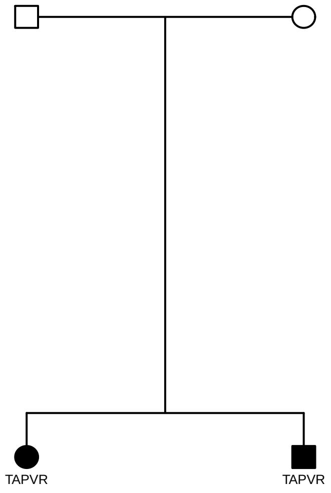

De_Luca_1 PMID:19933292

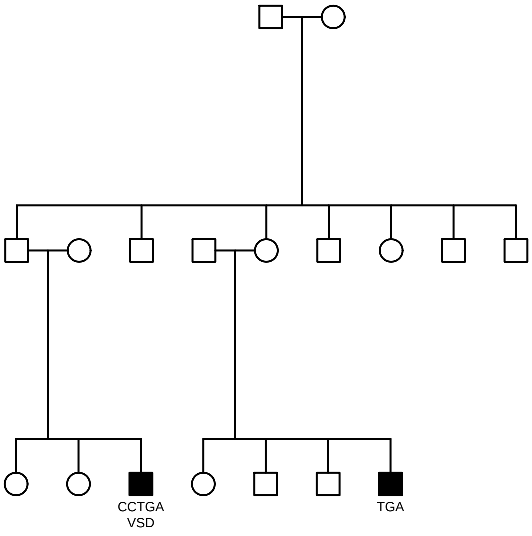

De_Luca_2 PMID:19933292

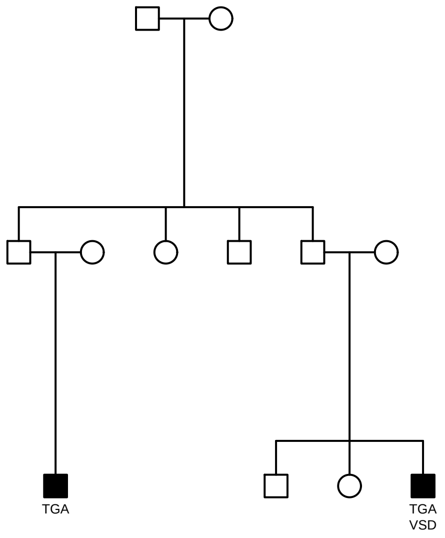

De_Luca_3 PMID:19933292

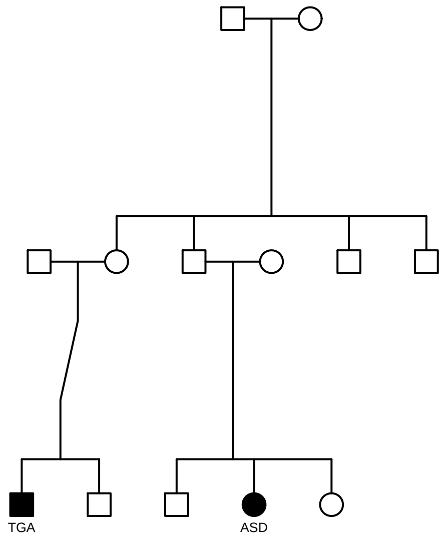

De_Luca_4 PMID:19933292

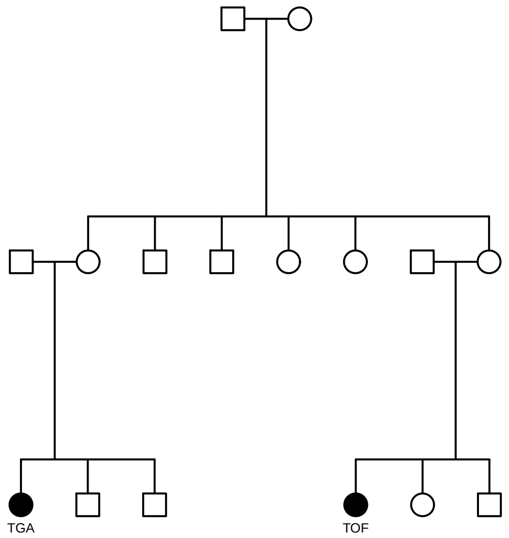

De_Luca_5 PMID:19933292

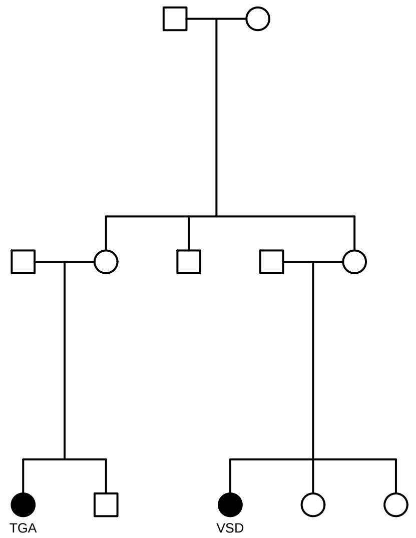

De_Luca_6 PMID:19933292

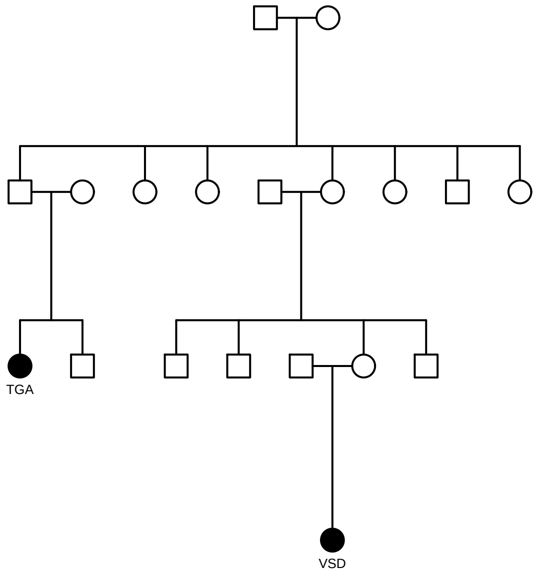

De_Luca_7 PMID:19933292

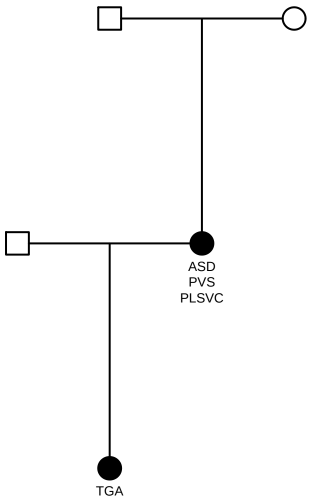

Zlotogora_1980 PMID:7277426

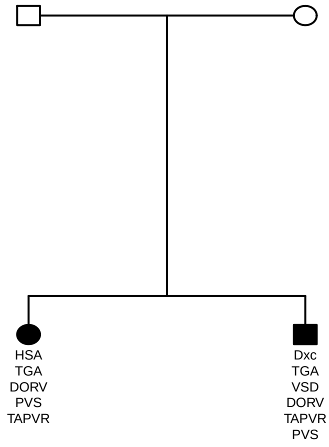

Eronen_2004 PMID:14648004

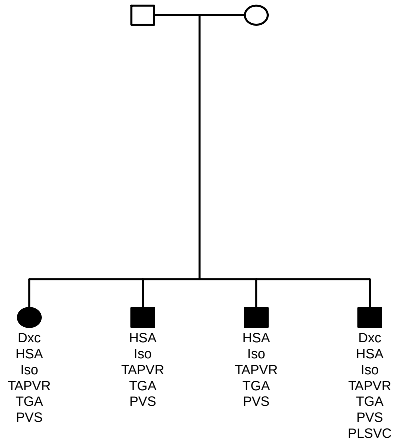

Delatycki_1997 PMID:9098496

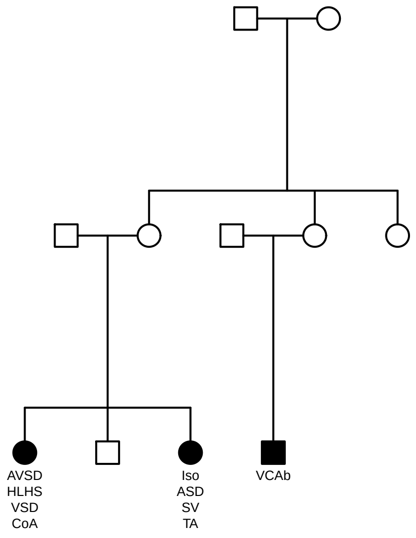

Morelli_2001_1 PMID:11471163

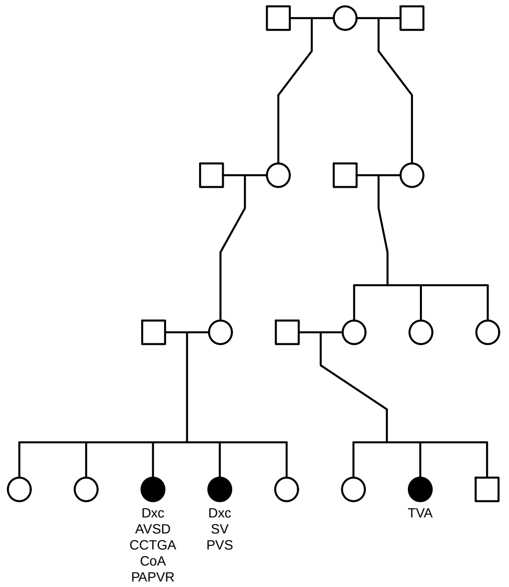

Morelli_2001_2 PMID:11471163

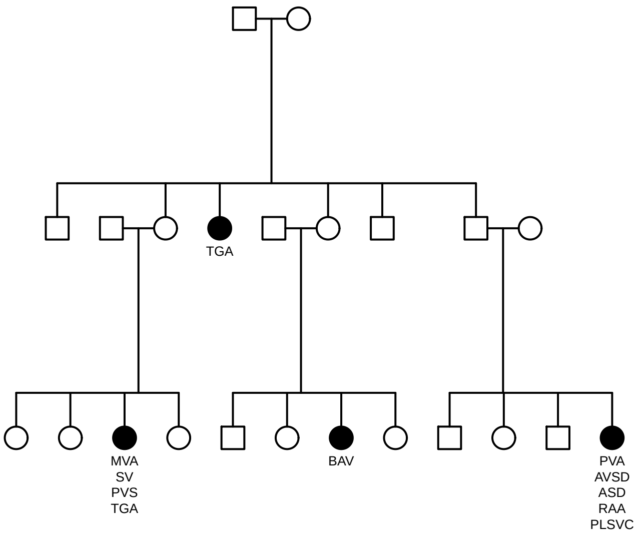

Morelli_2001_3 PMID:11471163

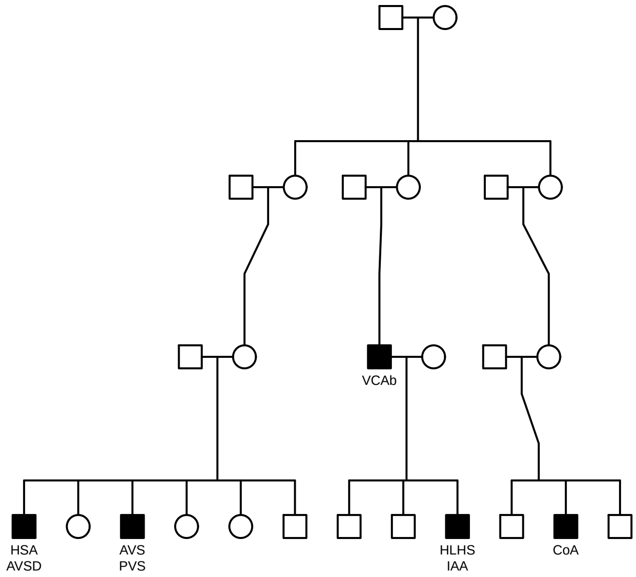

Soltan_1974 PMID:4839027

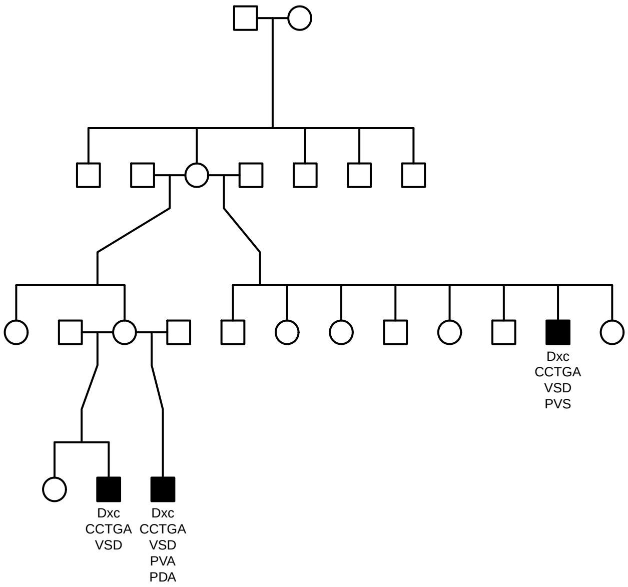

Toriello_1986 PMID:3146297

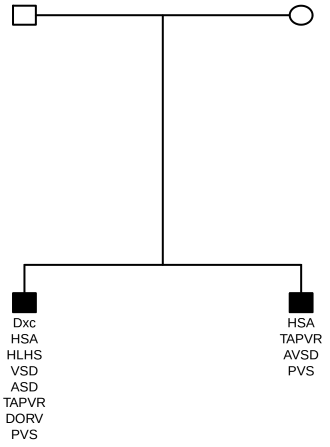

Mathias_1987 PMID:3674105

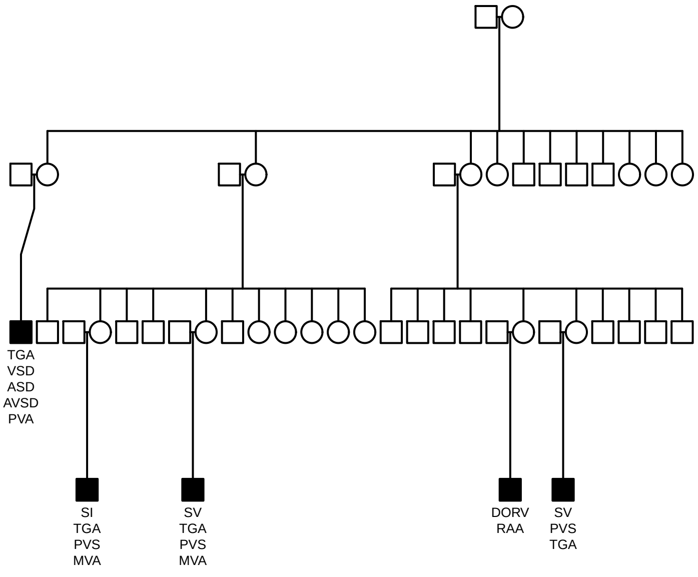

de_Meeus_1997 PMID:9021011

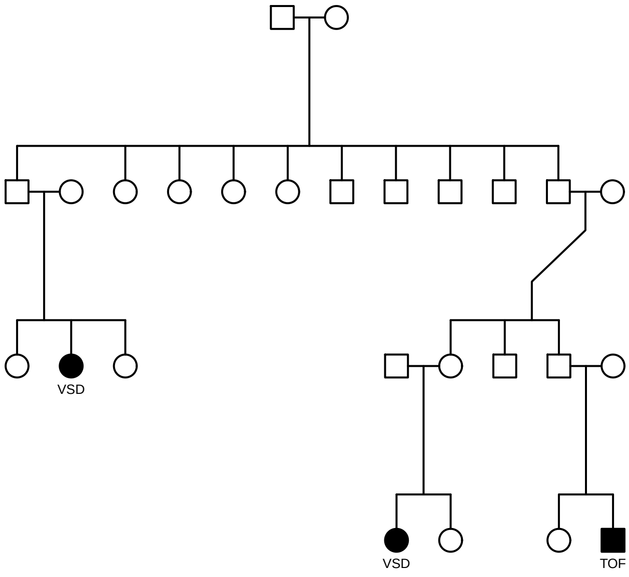

Arnold_1983 PMID:6638068

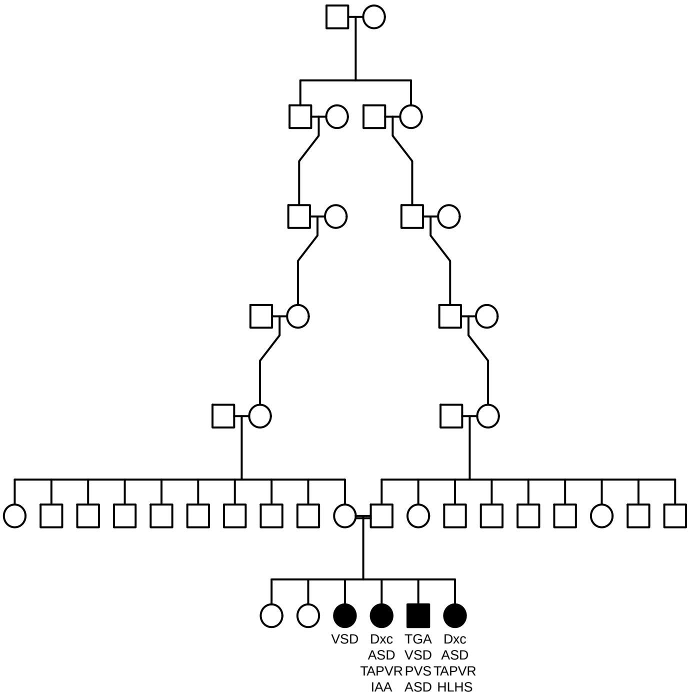

Rogers_1992 PMID:1433244

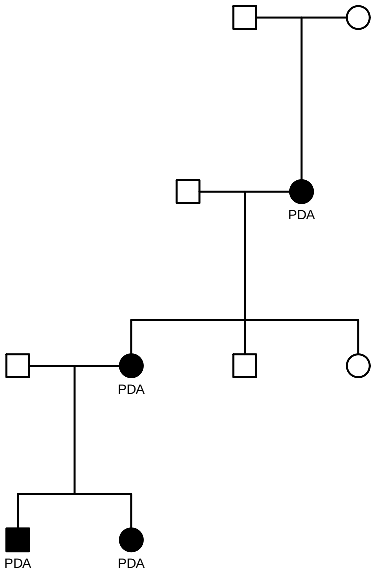

Slavotinek_1997 PMID:9217229

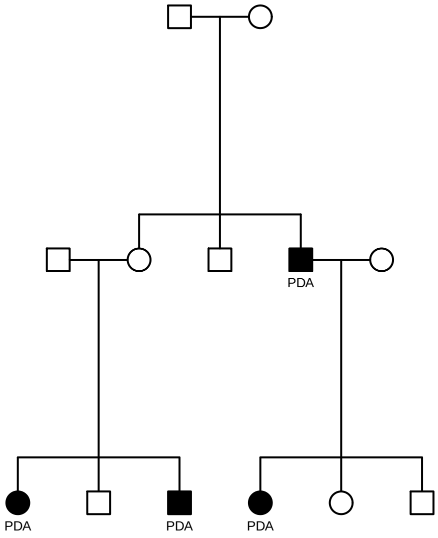

Lynch_1965_A PMID:5897316

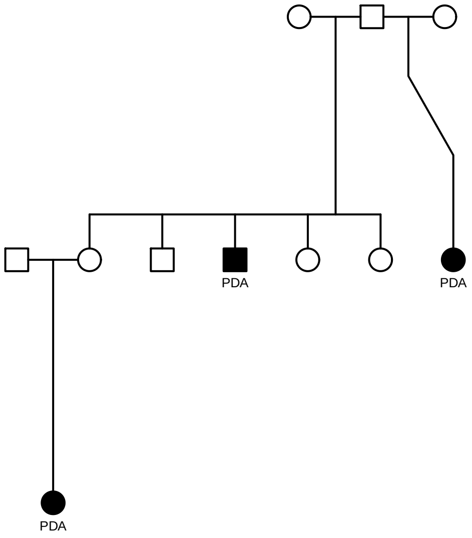

Lynch_1965_B PMID:5897316

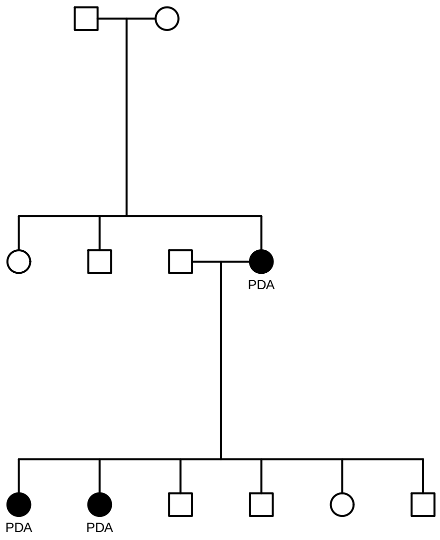

Burman_1961 PMID:13689120

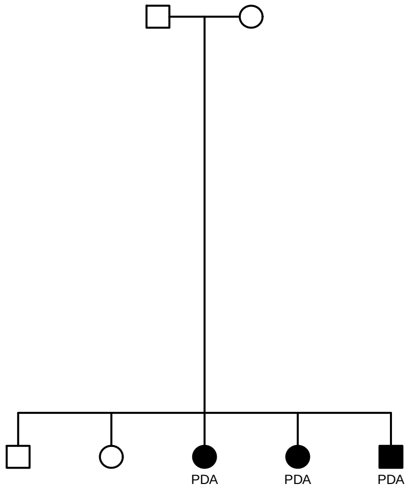

Record_2 PMID:13093871

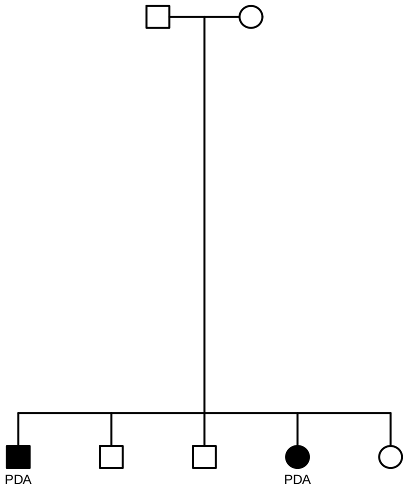

Record_3 PMID:13093871

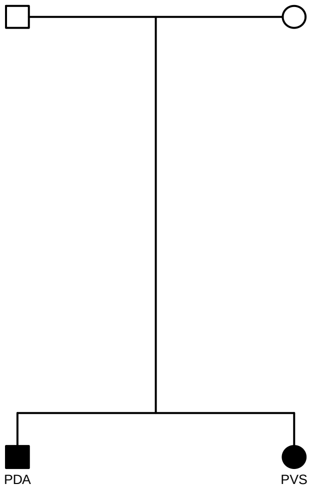

Record_7 PMID:13093871

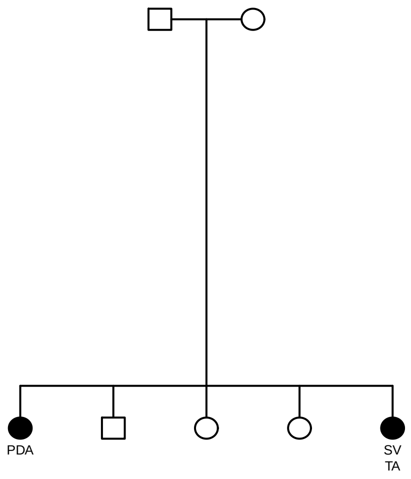

Sletten_1995 PMID:7645594

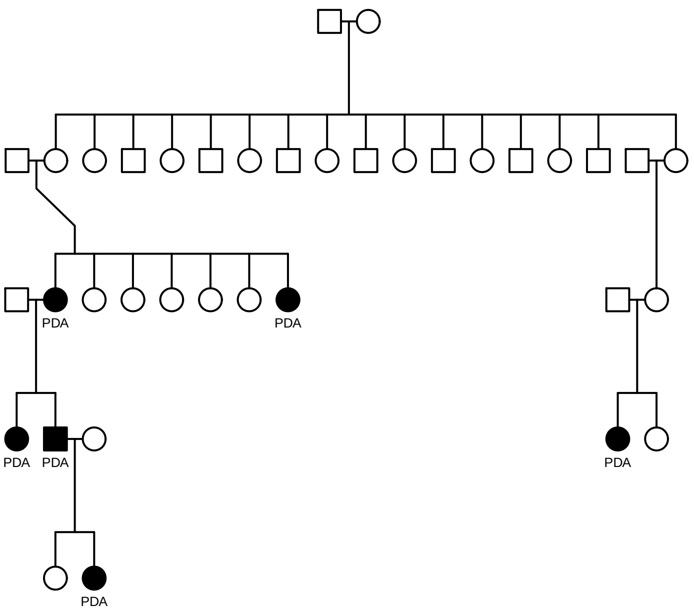

Ekstrom_S6 PMID:Acta Chirurgica Scandinavica[Suppl]1952;169:1−197

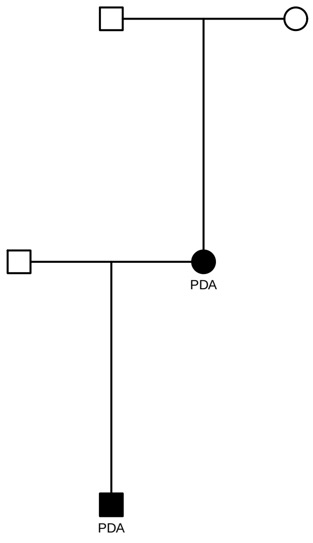

Ekstrom_KLB_9 PMID:Acta Chirurgica Scandinavica[Suppl] 1952;169:1−198

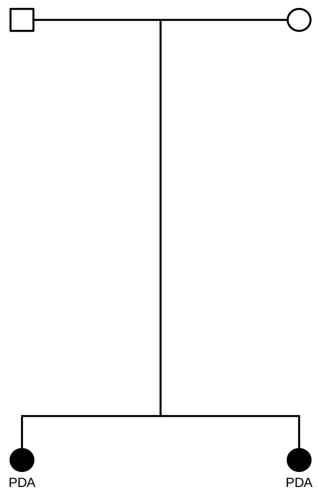

Ekstrom_KLB_30 PMID:Acta Chirurgica Scandinavica[Suppl]1952;169:1−199

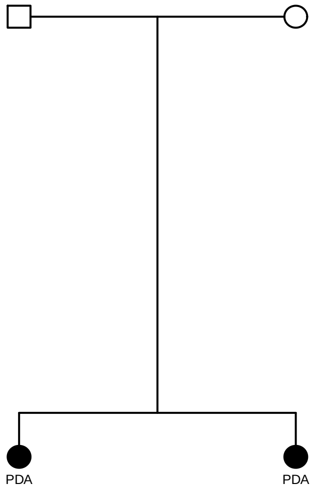

Ekstrom_KLB_52 PMID:Acta Chirurgica Scandinavica[Suppl]1952;169:1−200

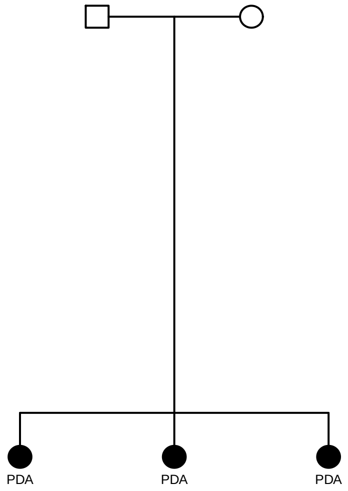

Ekstrom_S146 PMID:Acta Chirurgica Scandinavica[Suppl]1952;169:1−201

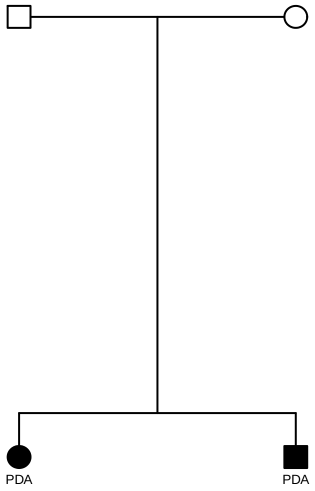

Niikowa_1983 PMID:6638069

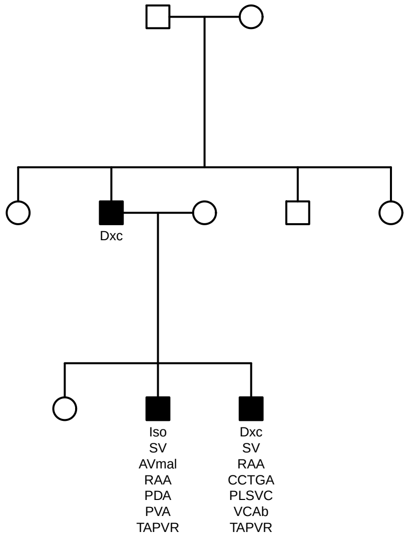

Mikkila_1994 PMID:8160739

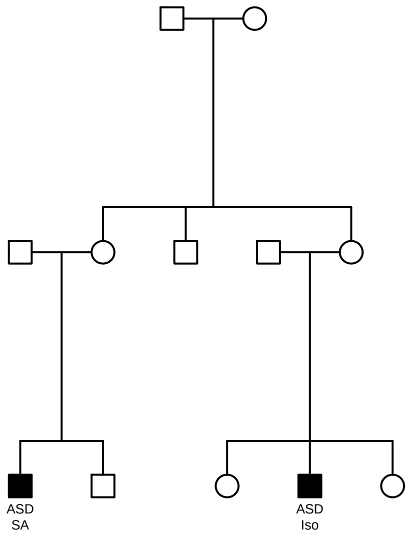

Chen_1977_a PMID:874654

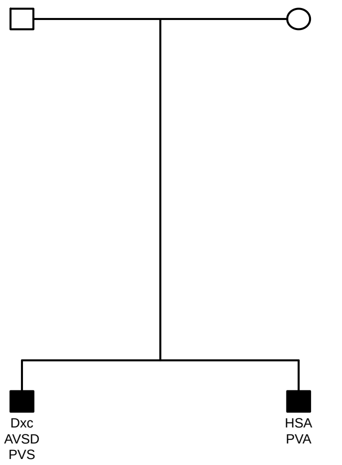

Chen_1977_b PMID:874654

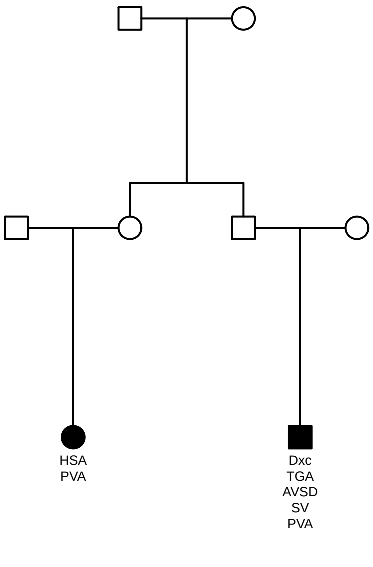

De_la_Monte_1985 PMID:4003441

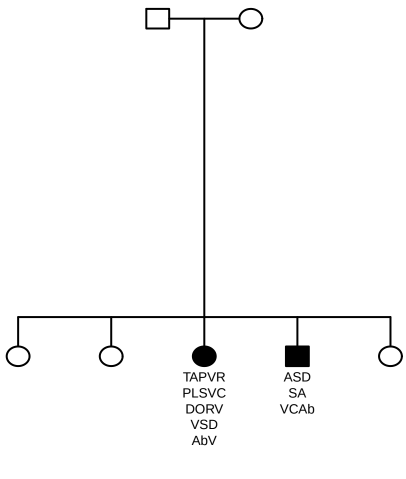

Katcher_1980 PMID:7360556

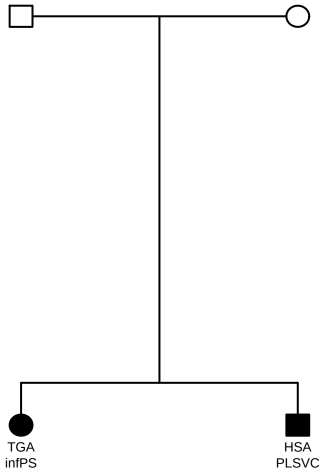

Cesko_1999 PMID:11125242

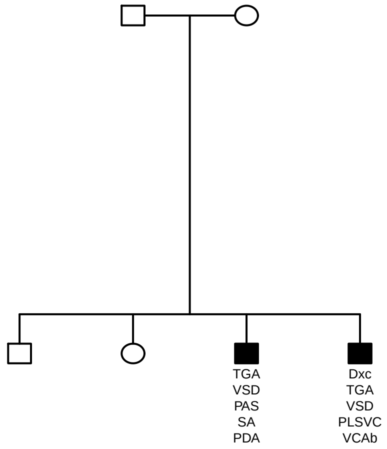

Cesko_1997 PMID:9152295

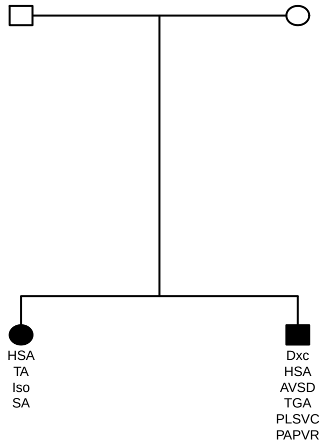

Simpson_1973 PMID:4774542

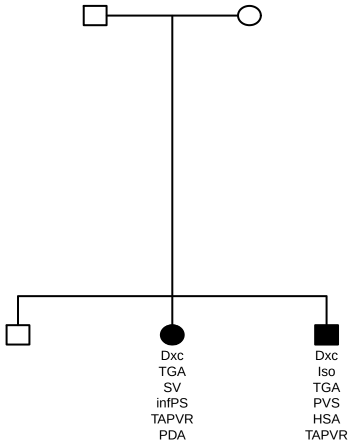

Ruttenberg_1964 PMID:14128648

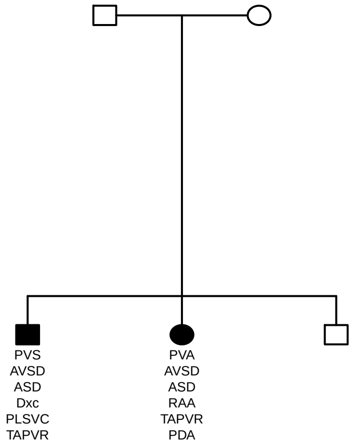

Alonso_2 PMID:7747776

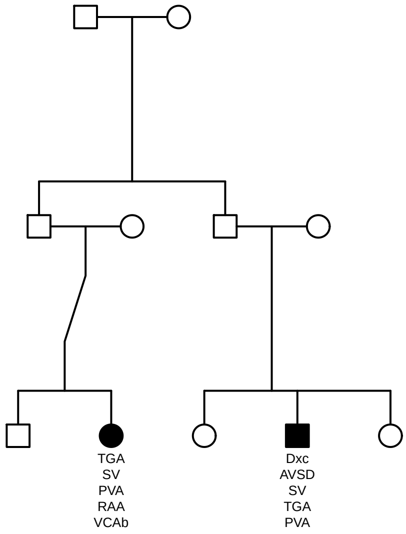

Alonso_3 PMID:7747776

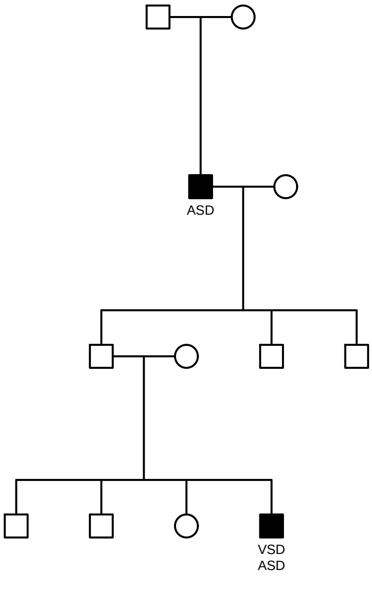

Alonso_6 PMID:7747776

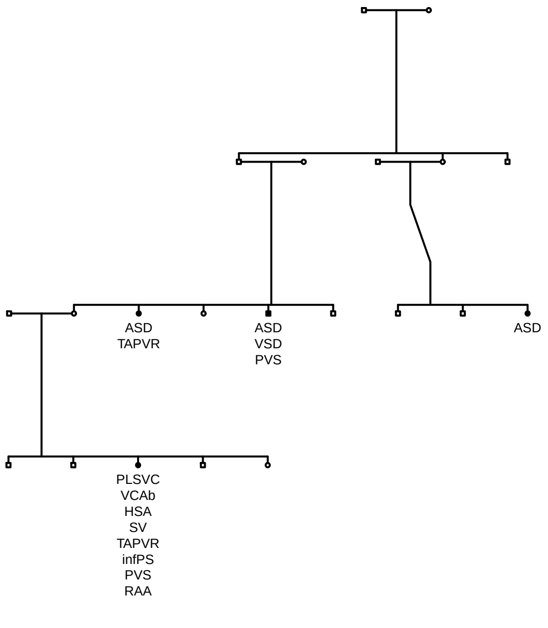

Casey_1996 PMID:8834043

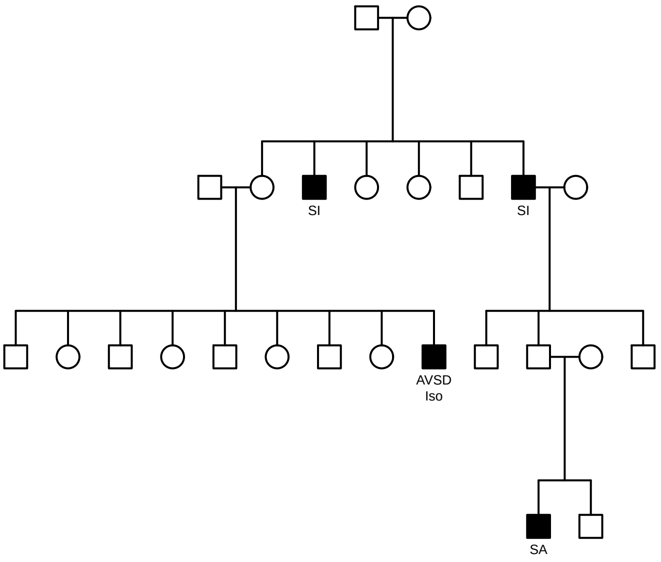

Gerboni_1993 PMID:8487284

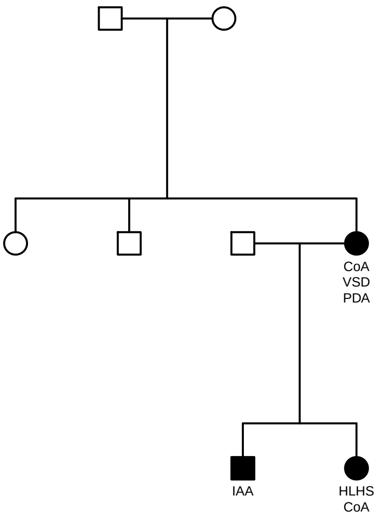

Shokeir_1971_A PMID:5111754

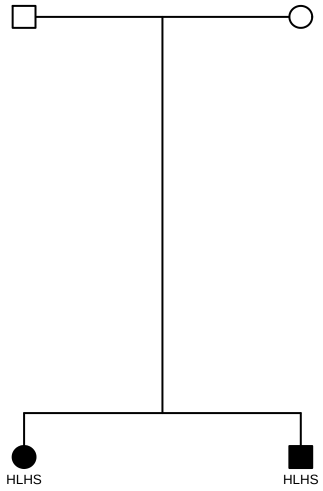

Shokeir_1971_B PMID:5111754

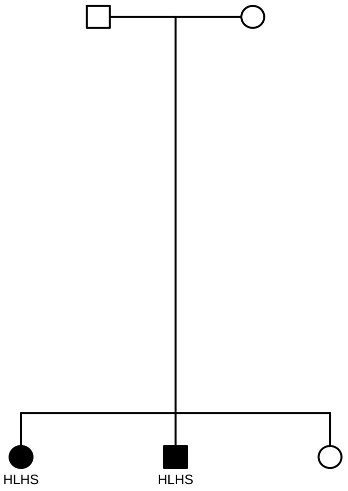

Shokeir_1971_C PMID:5111754

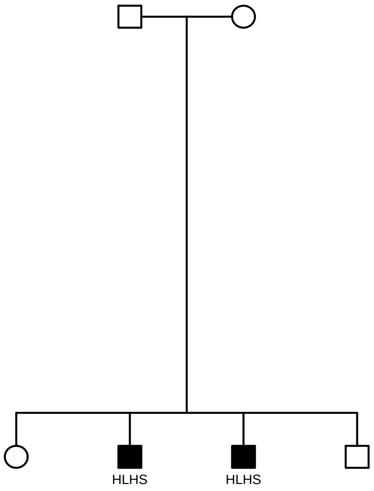

Shokeir_1971_D PMID:5111754

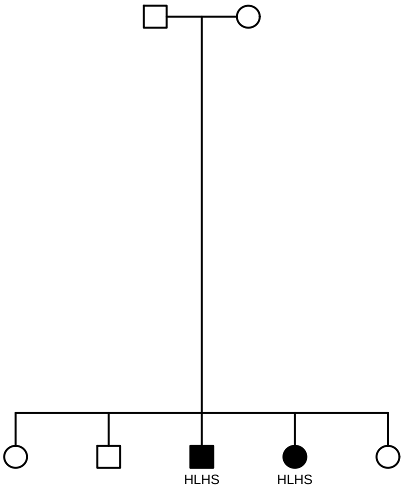

Shokeir_1971_E PMID:5111754

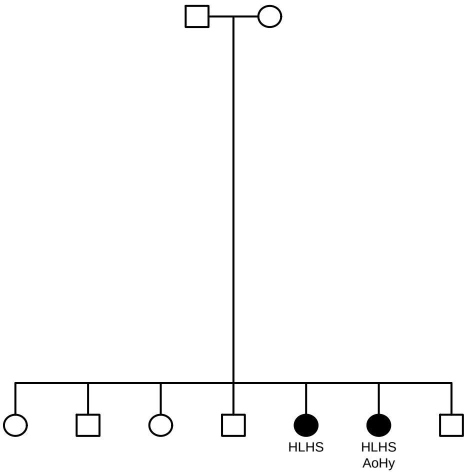

Brenner_1 PMID:2589285

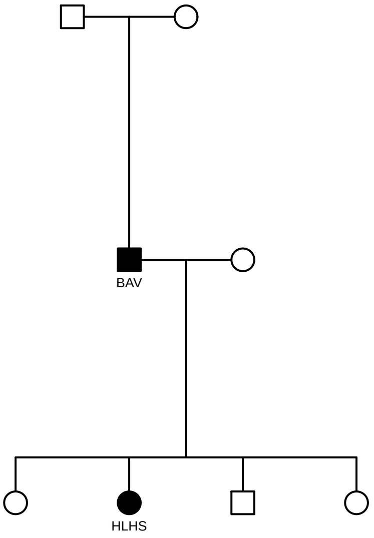

Brenner_2 PMID:2589285

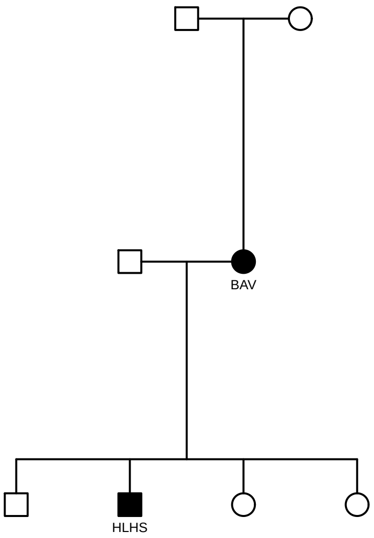

Brenner_4 PMID:2589285

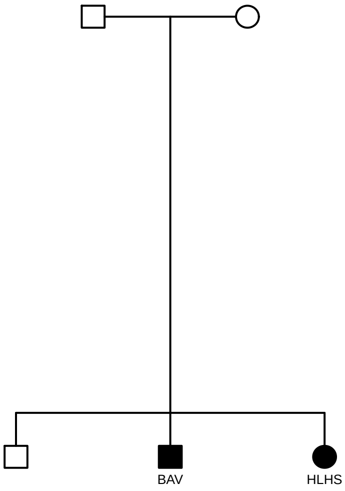

Mu_2005 PMID:16235019

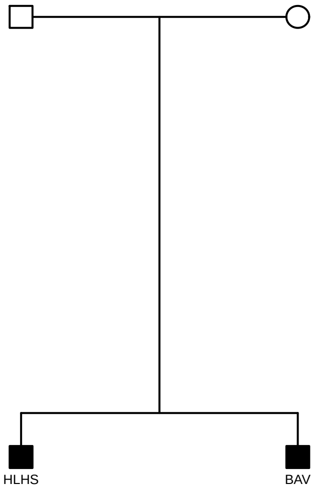

Menaheim_1990_1 PMID:2269543

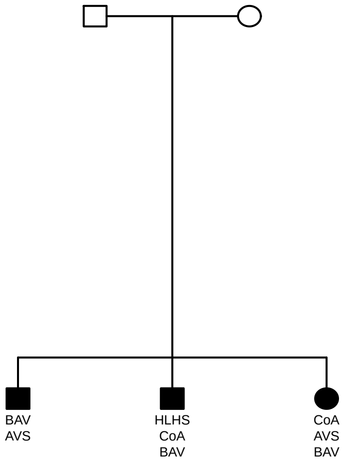

Menaheim_1990_2 PMID:2269543

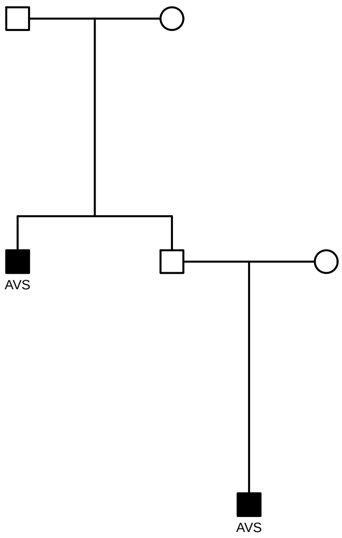

Fatimi_2006 PMID:17140990

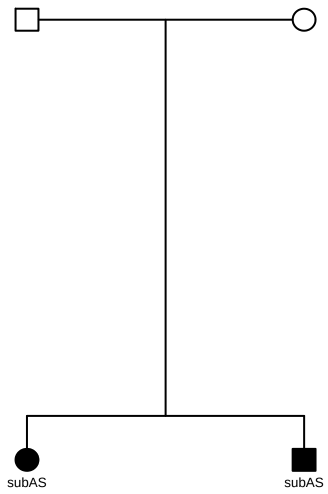

Digilio_1992 PMID:8354327

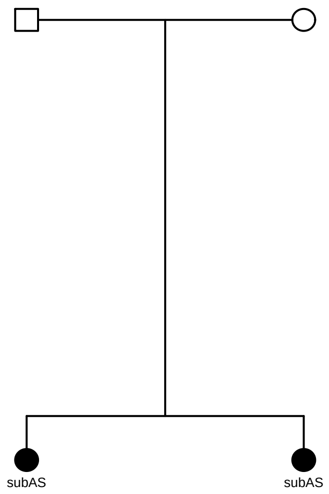

Abdallah_1994 PMID:7991438

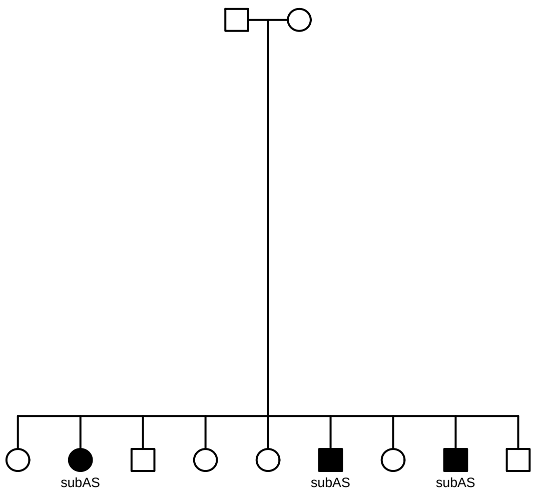

Richardson_1991 PMID:1829065

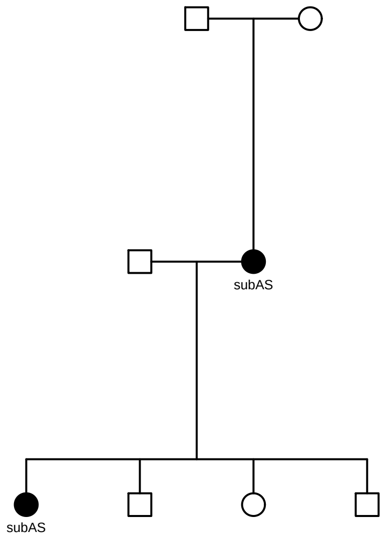

Onat_1984 PMID:6537946

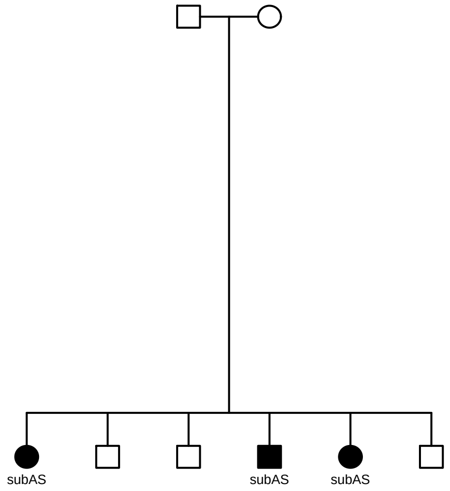

Fryns_1979 PMID:573203

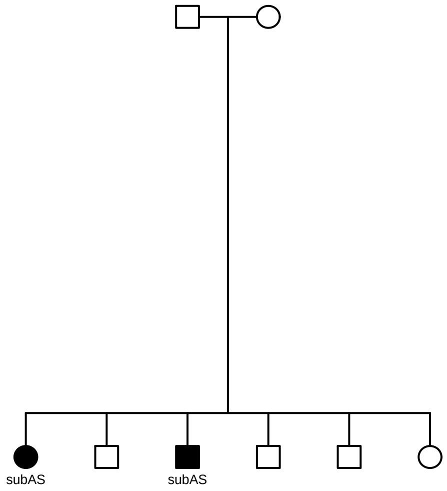

Urbach_1 PMID:4053117

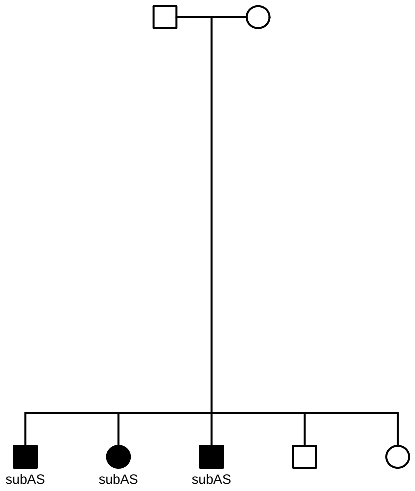

Urbach_2 PMID:4053117

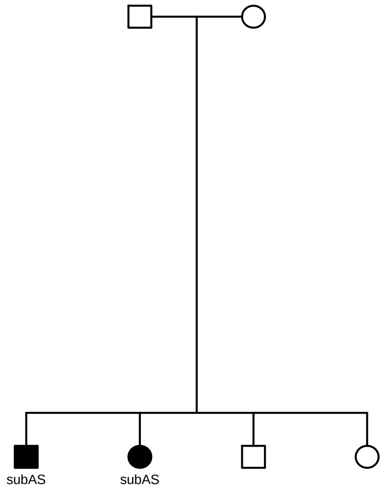

Urbach_3 PMID:4053117

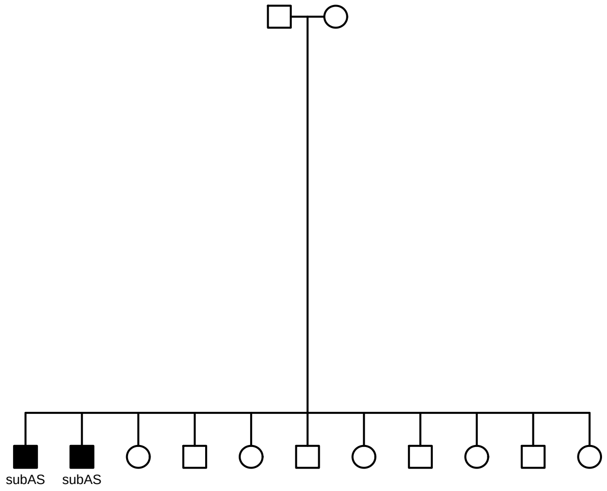

Gale_1974 PMID:4533948

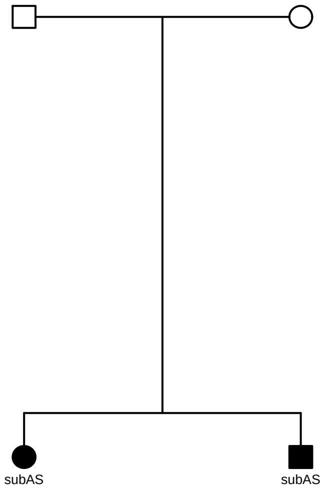

Goodyear_1960 PMID:13707075

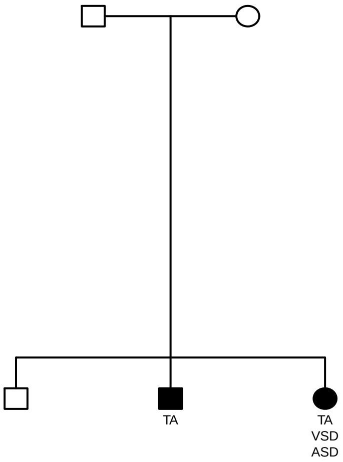

Brunson_1978 PMID:685815

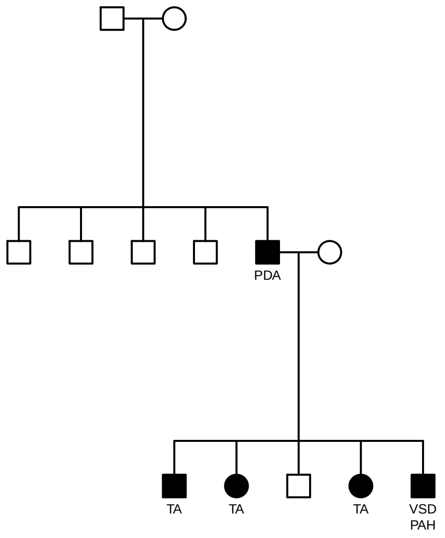

Abushaban_2003 PMID:12574981

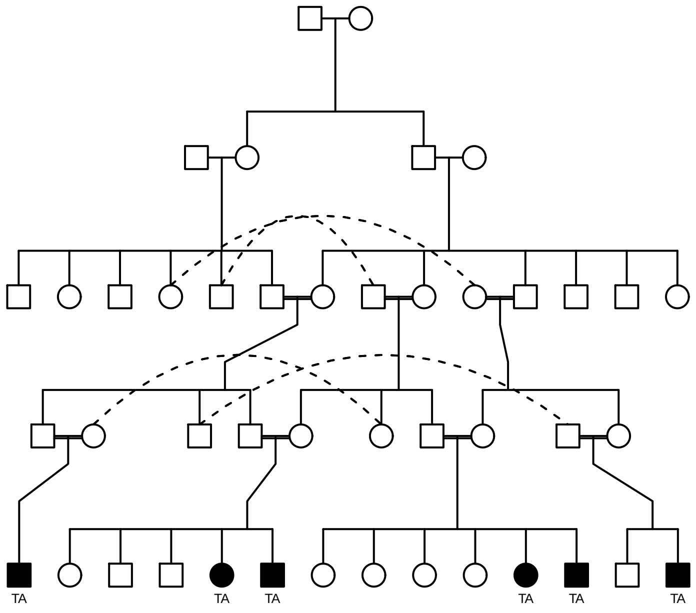

Kwiatkowska_2006 PMID:17295158

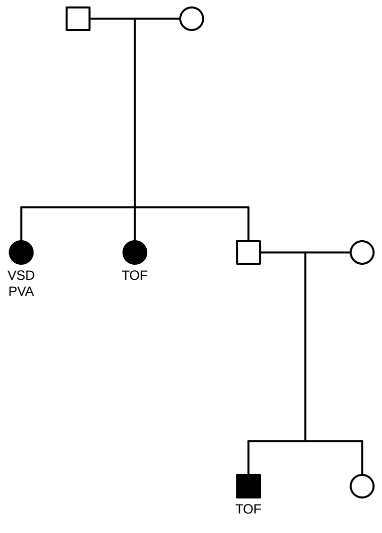

Nakada_1996 PMID:8992863

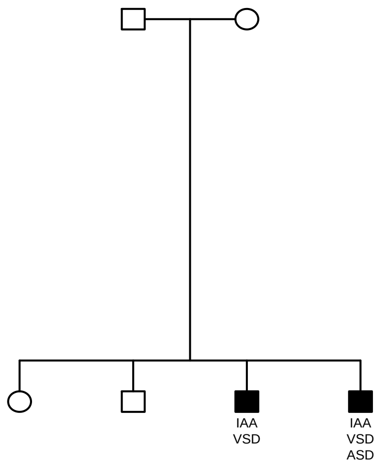

Buch_1980 PMID:7211363

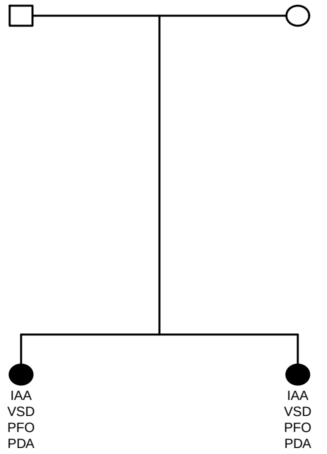

Kawashima_1987 PMID:3478461

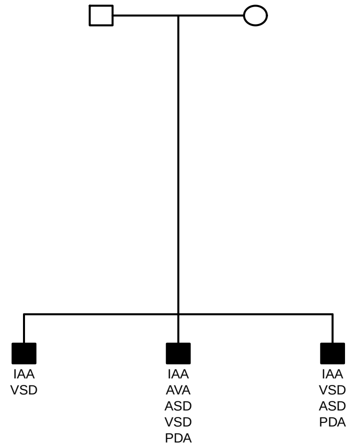

Pankau_1990 PMID:2368805

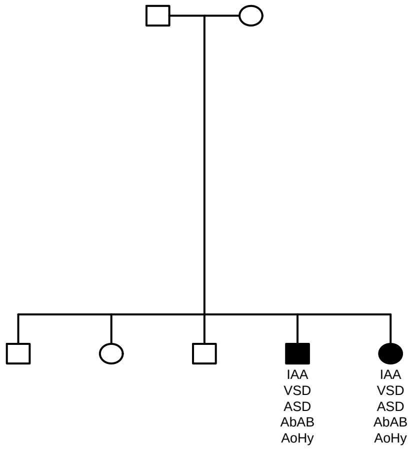

Neufeld_1960 PMID:13728505

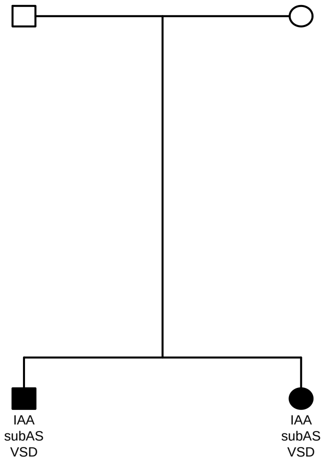

Gobel_1993 PMID:8469627

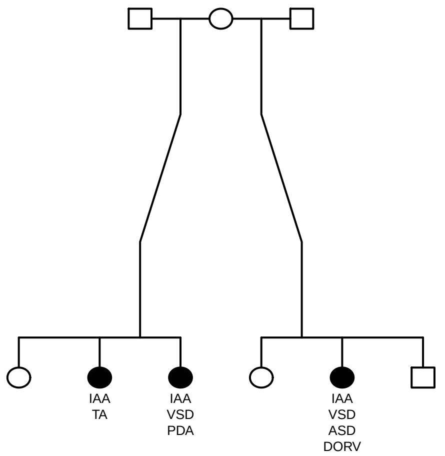

Cripe_a PMID:15234422

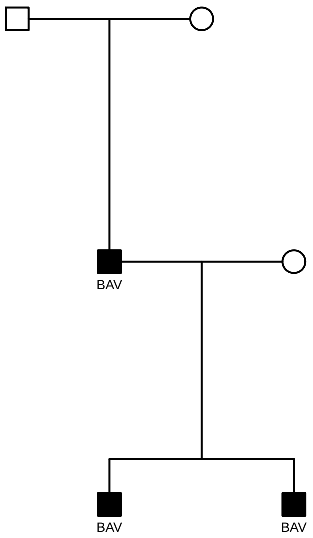

Cripe_b PMID:15234422

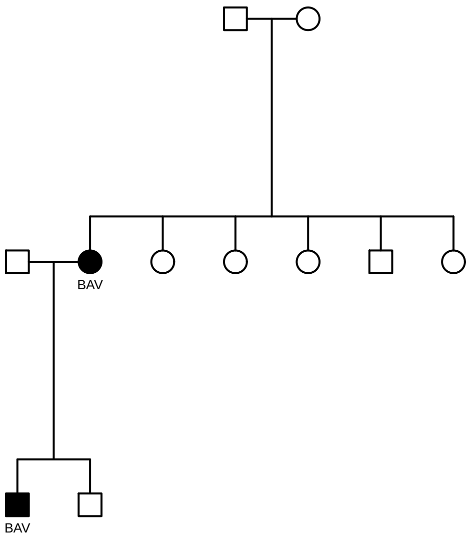

Cripe_c PMID:15234422

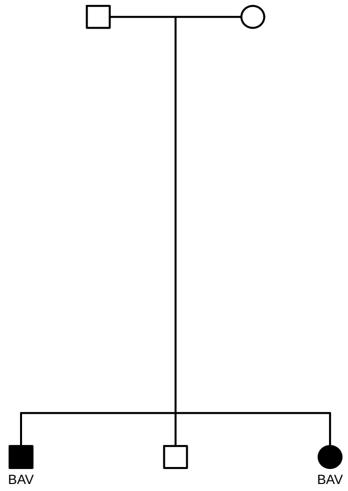

Cripe_d PMID:15234422

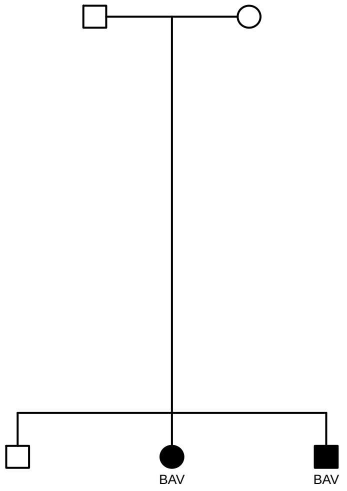

Cripe_e PMID:15234422

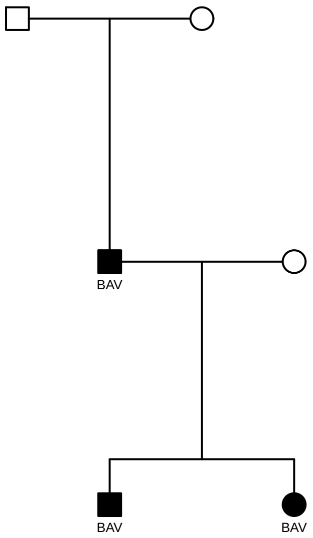

Cripe_f PMID:15234422

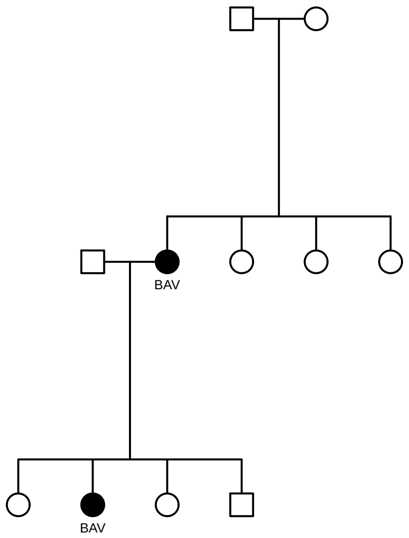

Cripe_g PMID:15234422

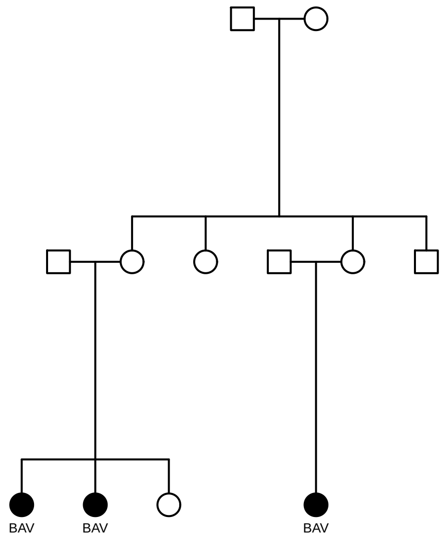

Cripe_h PMID:15234422

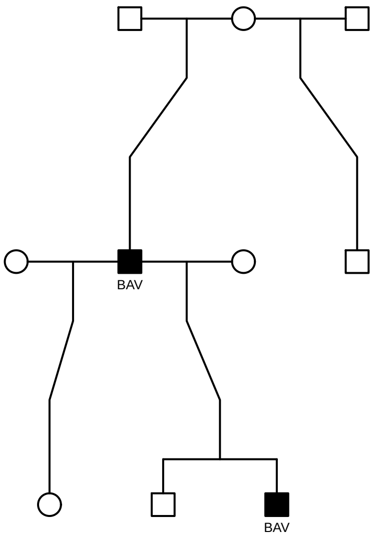

Cripe_i PMID:15234422

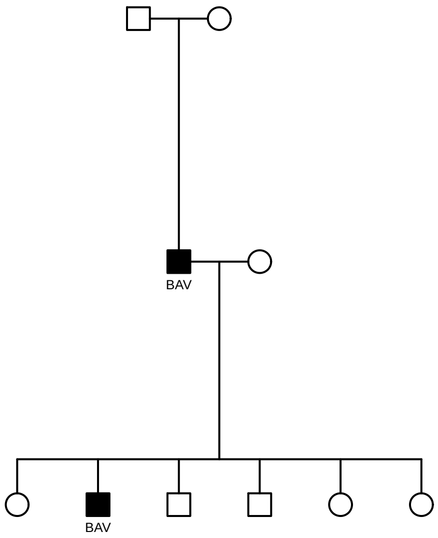

Godden_1987 PMID:3582390

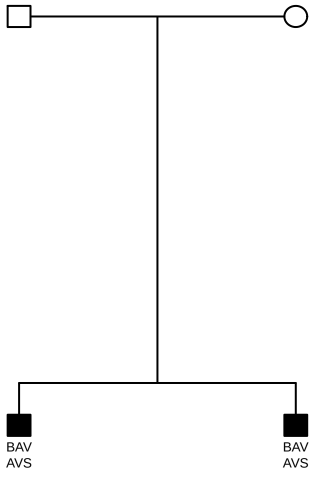

Clementi_1996 PMID:8723060

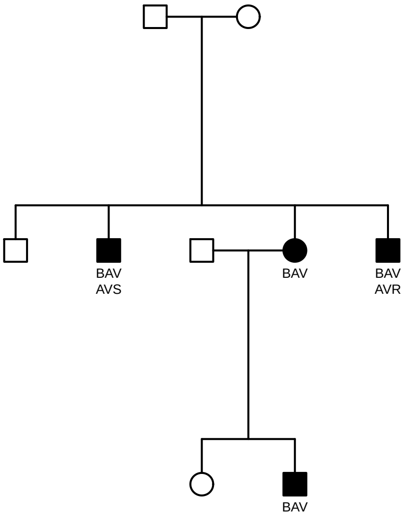

Huntington_1 PMID:9385911

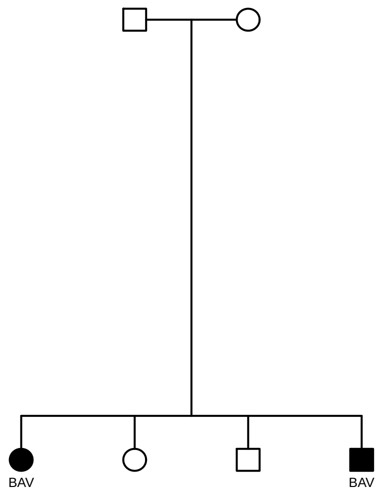

Huntington_2 PMID:9385911

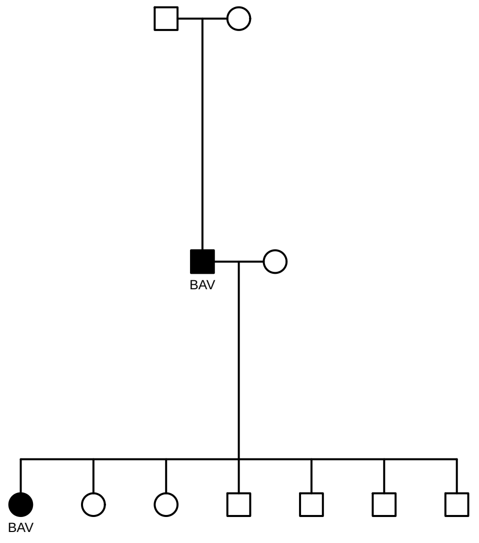

Huntington_3 PMID:9385911

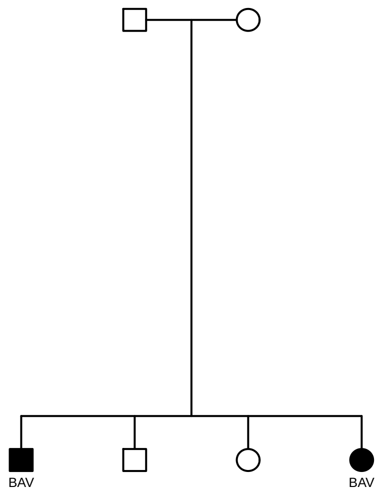

Huntington_4 PMID:9385911

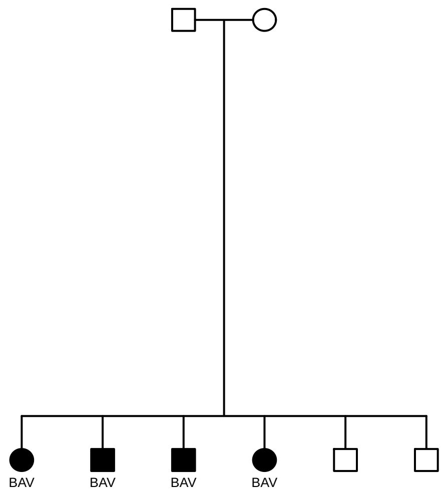

Huntington_10 PMID:9385911

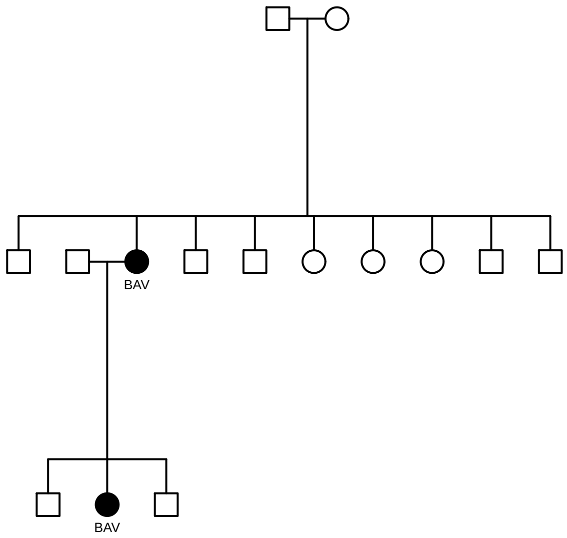

Huntington_9 PMID:9385911

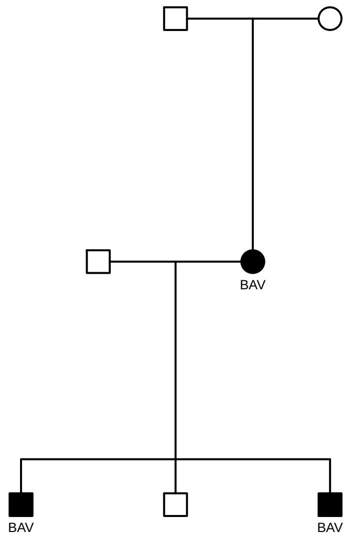

Huntington_23 PMID:9385911

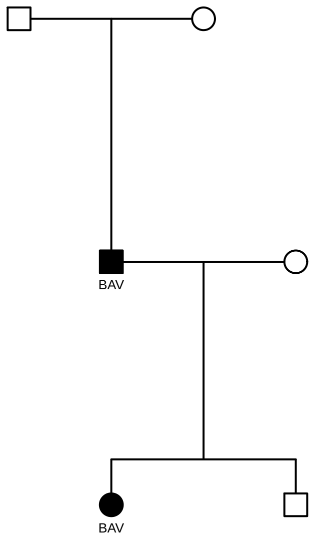

Huntington_26 PMID:9385911

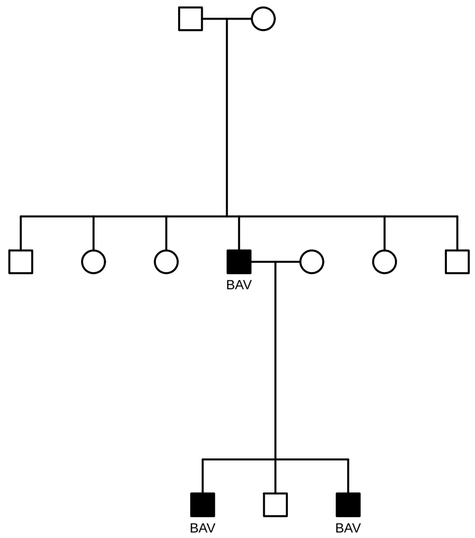

Huntington_27 PMID:9385911

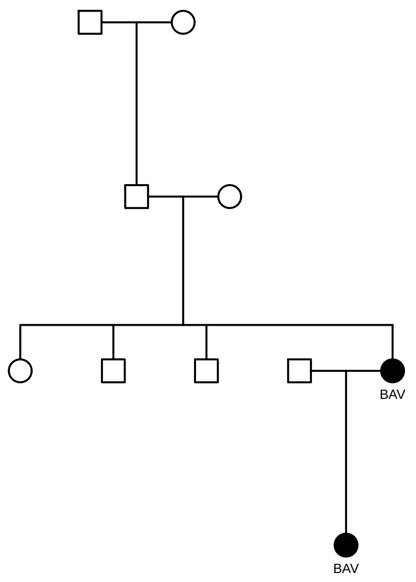

Huntington_28 PMID:9385911

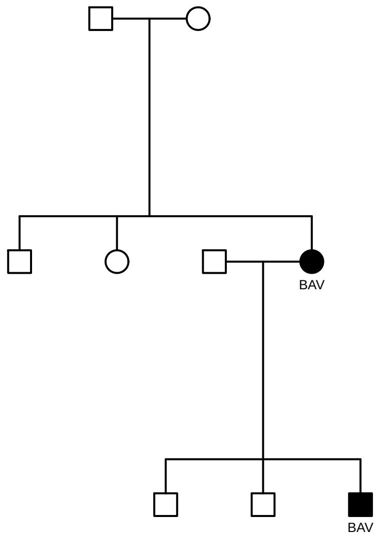

Brekke_1952 PMID:13050604

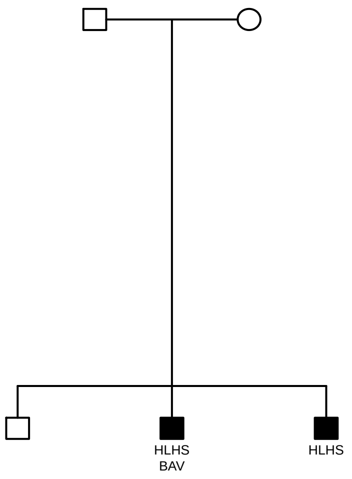

Moss_1955 PMID:14368483

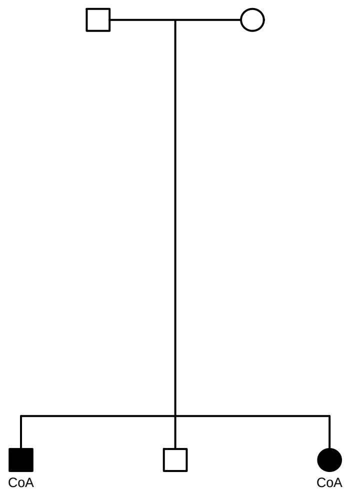

Taylor_1952 PMID:13030400

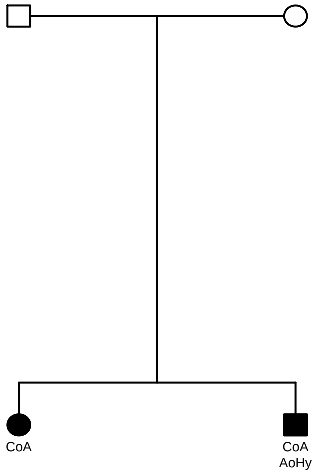

Campbell_1961 PMID:13690260

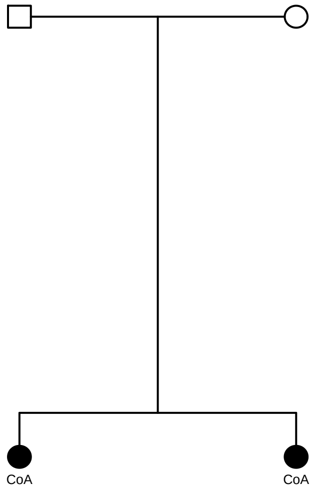

Beekman_1985 PMID:4061317

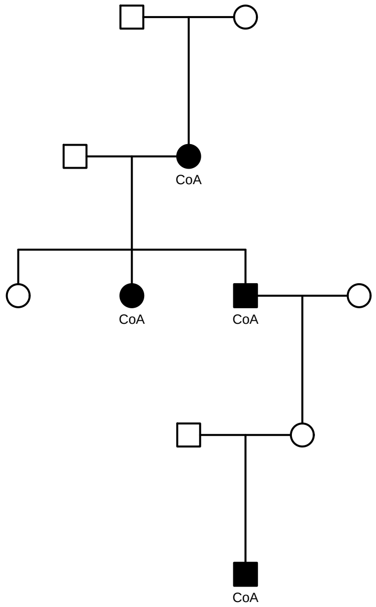

Sehested PMID:7200795

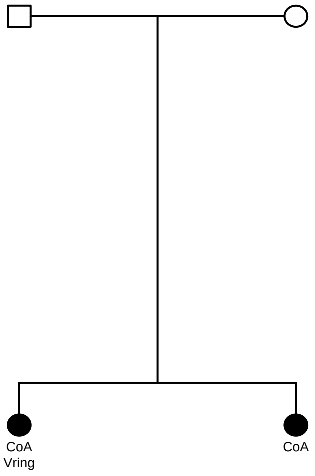

Keller_1965 PMID:14314175

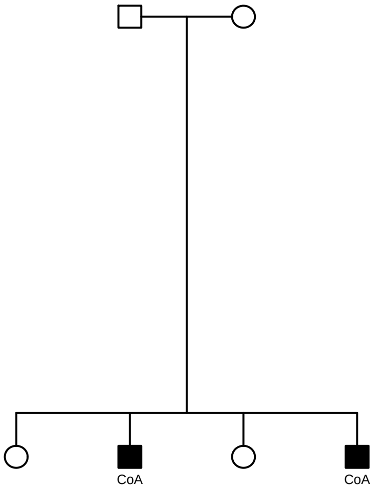

Khau_van_Kien_2004 PMID:14722581

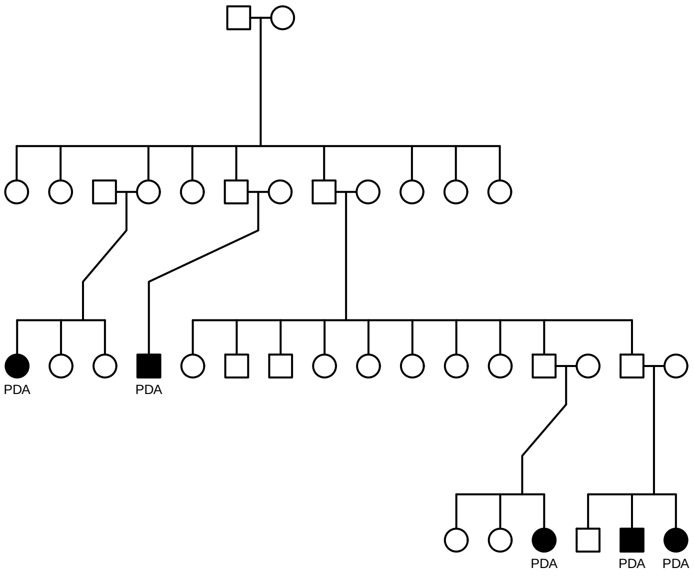

Joyce_1954 PMID:13160445

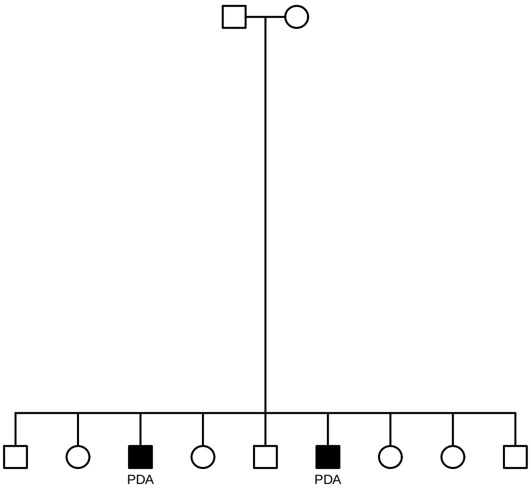

Davidson_1992 PMID:8326495

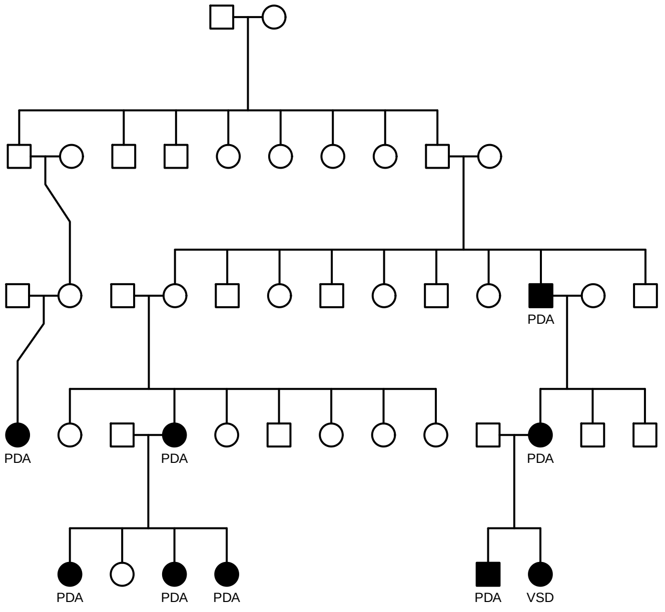

Glancy_2001 PMID:11249915

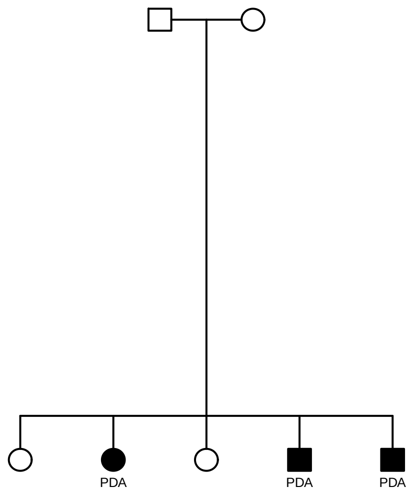

Martin PMID:3767421

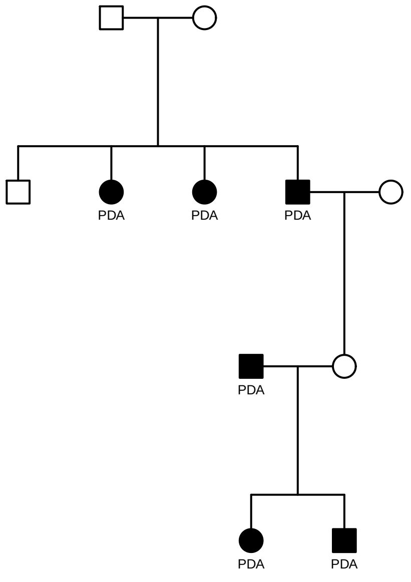

Gelb_1999 PMID:10533032

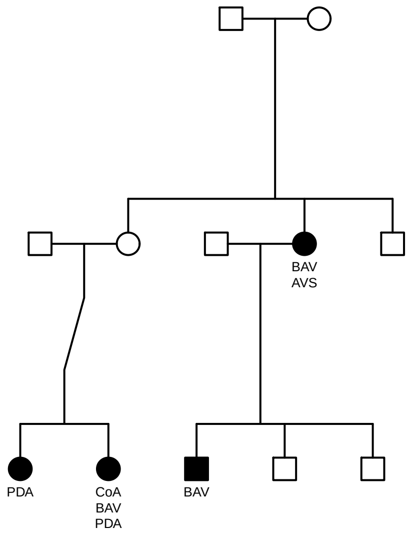

Wei_1984 PMID:6741821

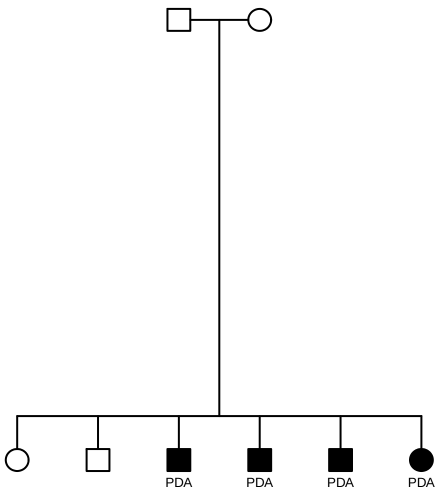

Grobman_1996 PMID:8841248

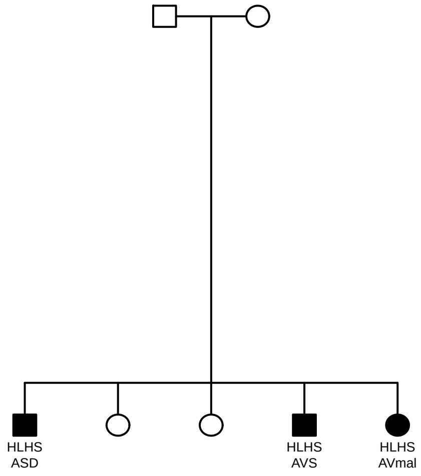

Simon_1974_1 PMID:4426202

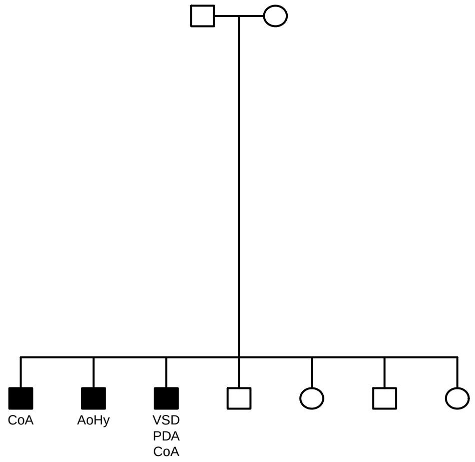

Simon_1974_2 PMID:4426202

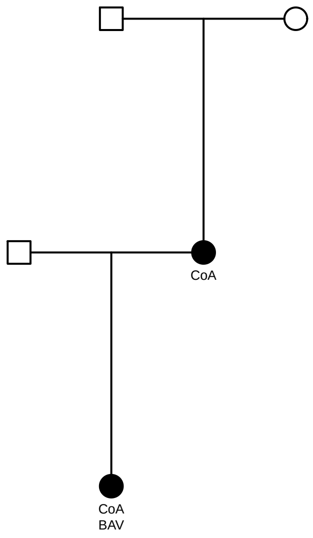

Simon_1974_3 PMID:4426202

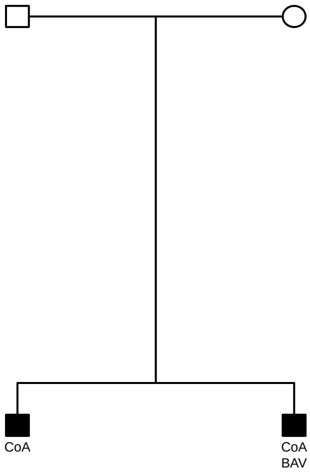

Simon_1974_4 PMID:4426202

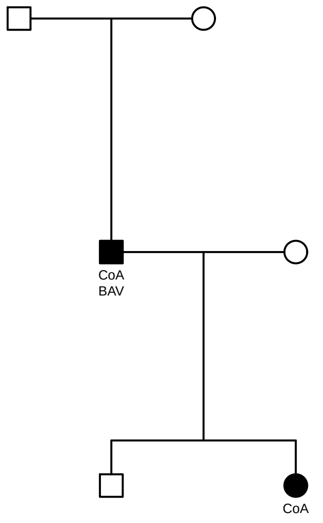

Simon_1974_5 PMID:4426202

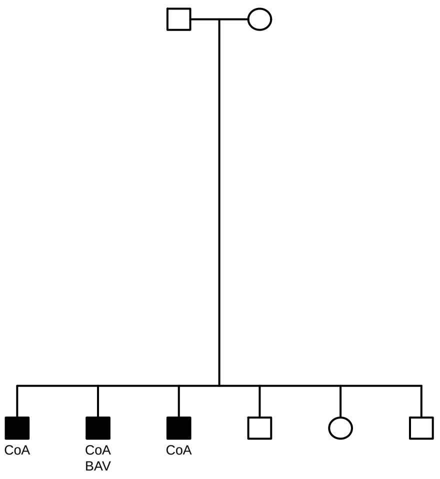

Lang_1991 PMID:1997221

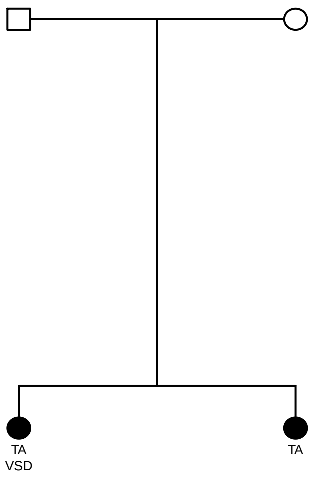

McDonald_1989 PMID:2767077

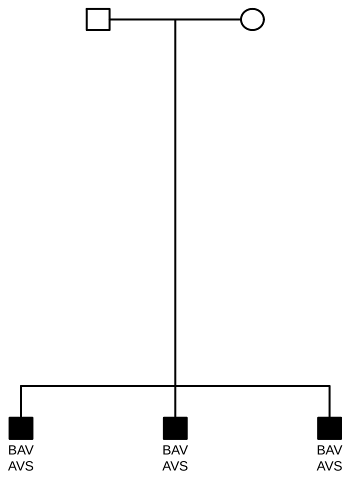

Digilio_1997_a PMID:9375931

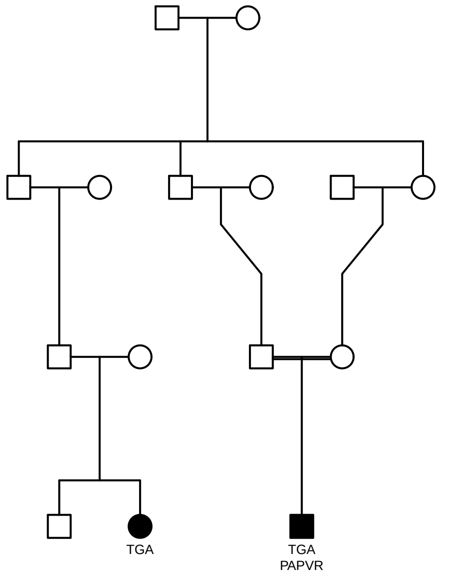

Digilio_2001_1 PMID:11733399

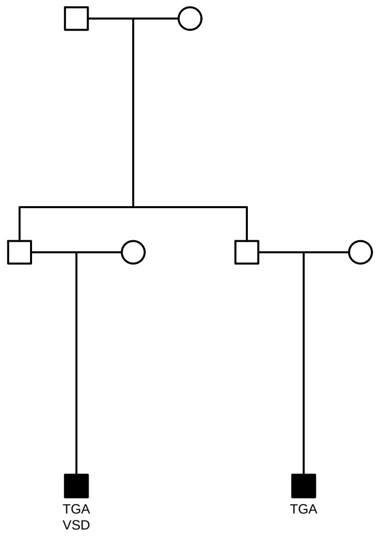

Digilio_2001_3 PMID:11733399

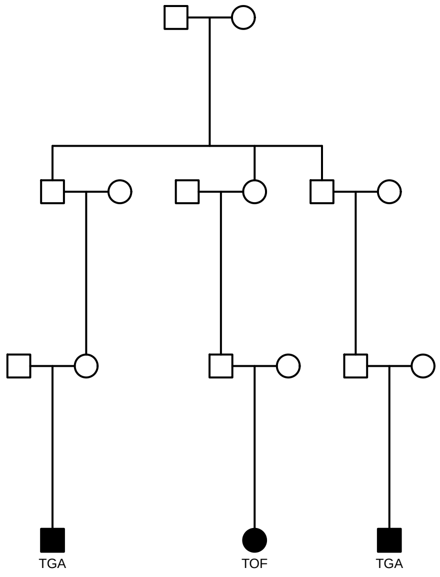

Digilio_2001_4 PMID:11733399

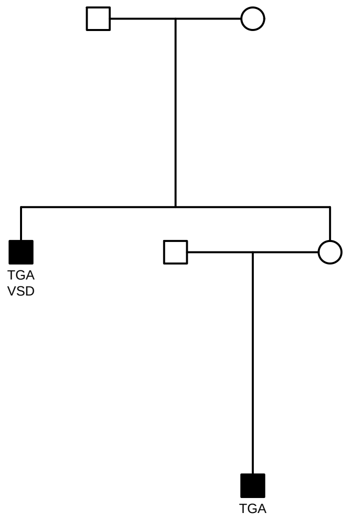

Digilio_2001_5 PMID:11733399

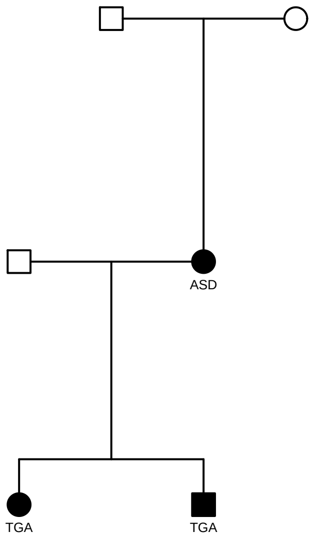

Digilio_2001_6 PMID:11733399

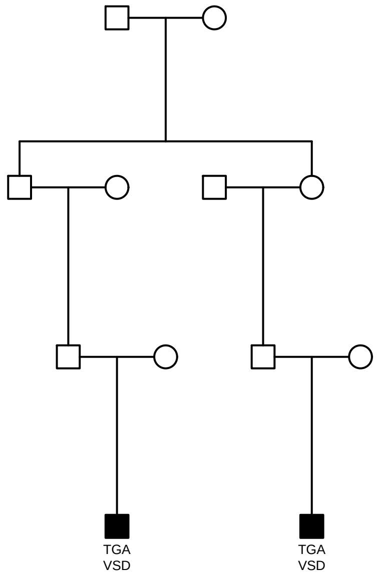

Digilio_2001_7 PMID:11733399

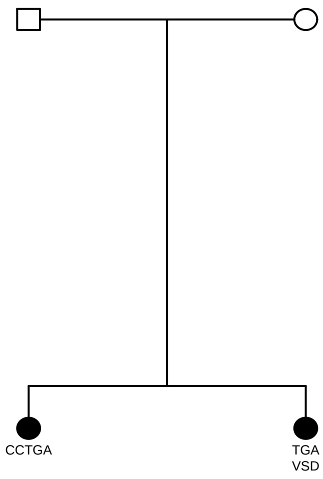

Digilio_2001_8 PMID:11733399

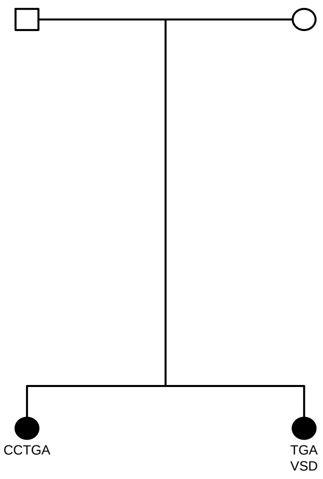

Digilio_2001_9 PMID:11733399

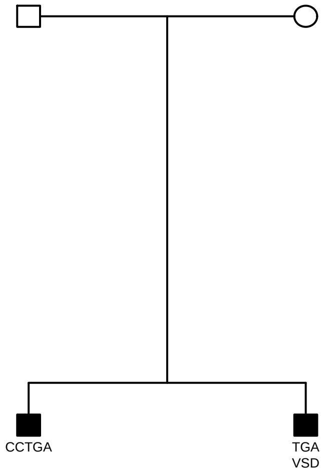

Digilio_2001_10 PMID:11733399

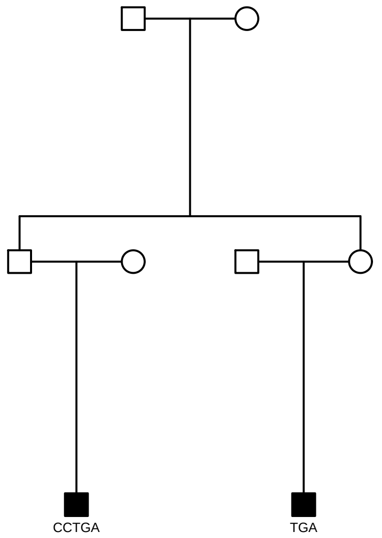

Digilio_2001_11 PMID:11733399

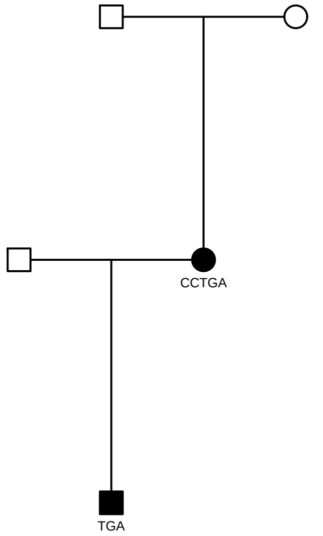

Zlotogora_1987 PMID:3812559

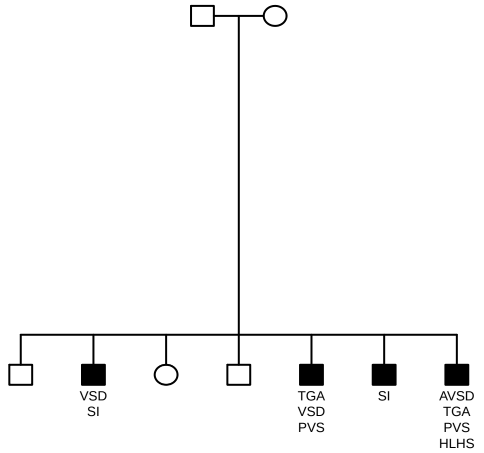

Van_de_Meerakker_2011 PMID:21386876

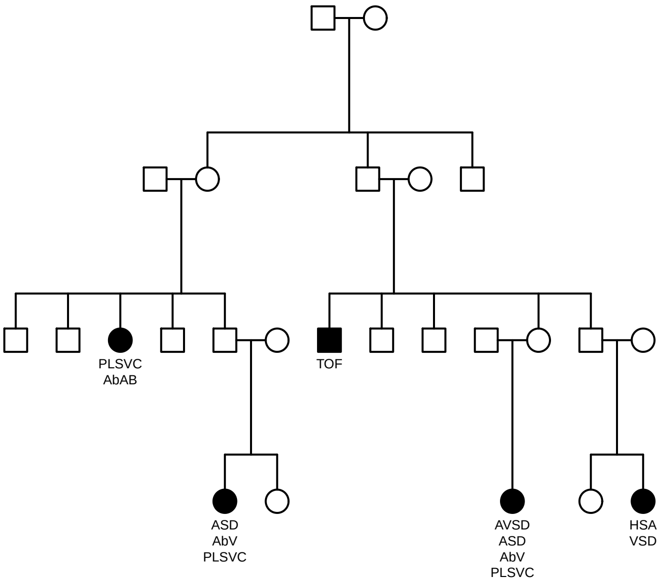

McKusick_1972 PMID:4112361

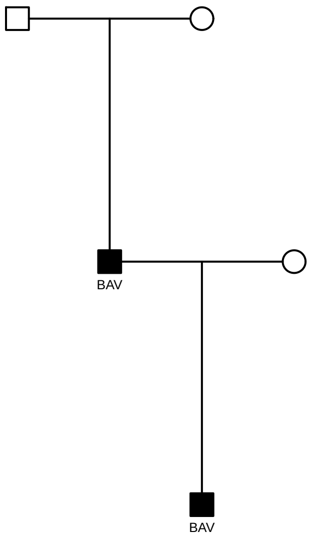

Woods_1994 PMID:7815430

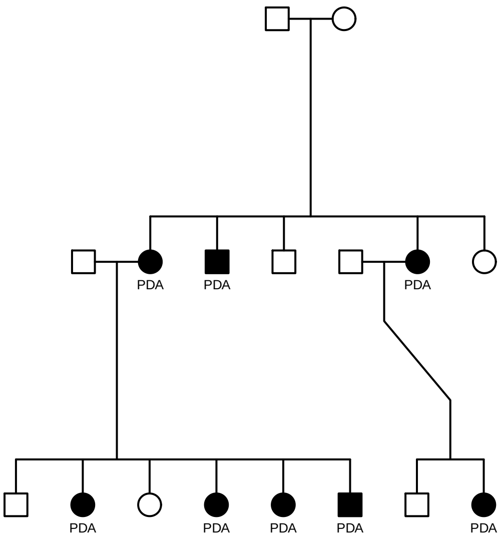

Davison_120 PMID:6082900

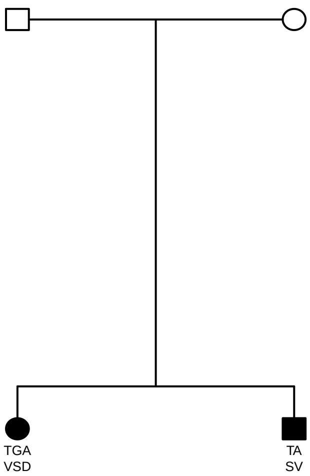

Davison_209 PMID:6082900

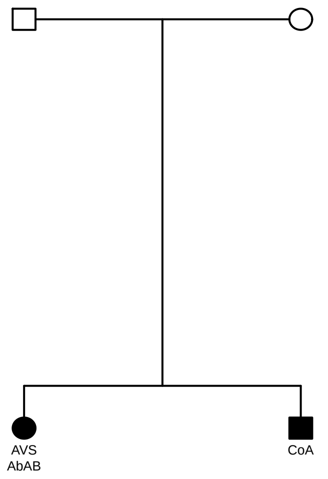

Davison_205 PMID:6082900

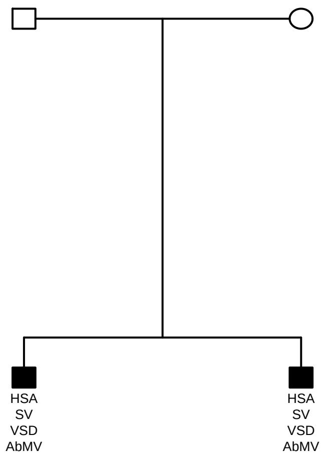

Davison_13 PMID:6082900

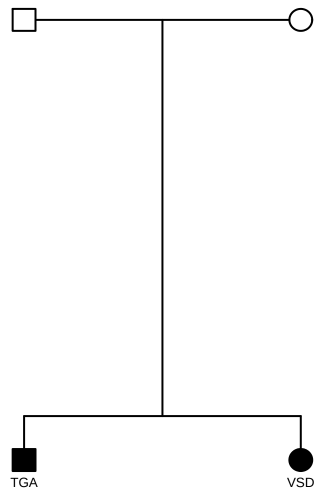

Davison_69 PMID:6082900

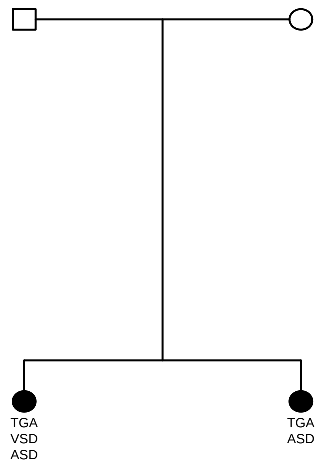

Davison_108 PMID:6082900

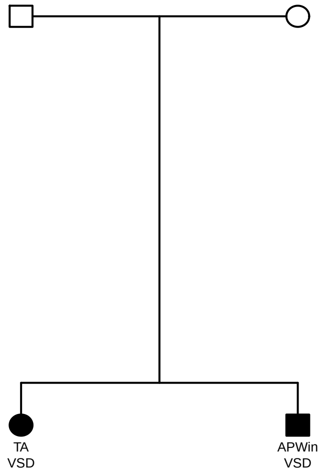

Davison_134 PMID:6082900

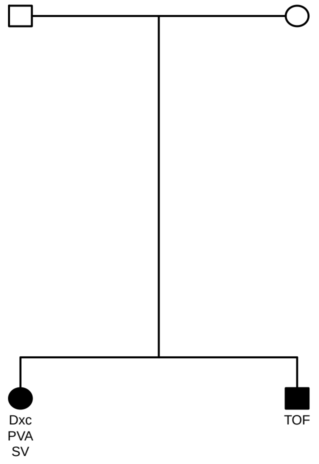

Davison_4 PMID:6082900

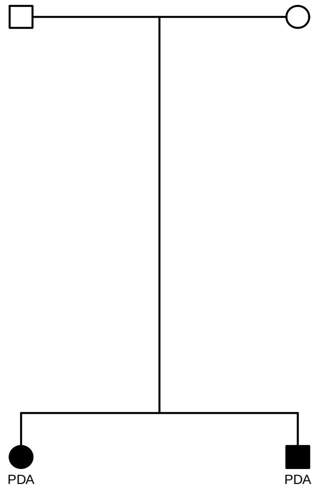

DiChiara_1980 PMID:7377161

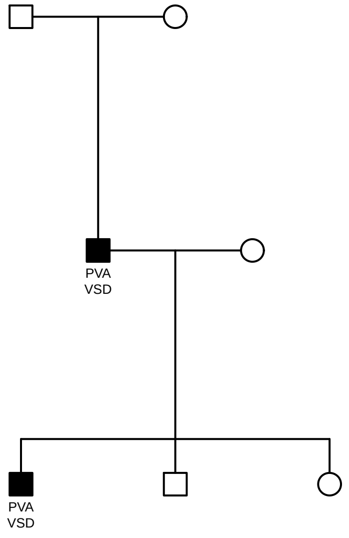

Udwadia_1996 PMID:8781095

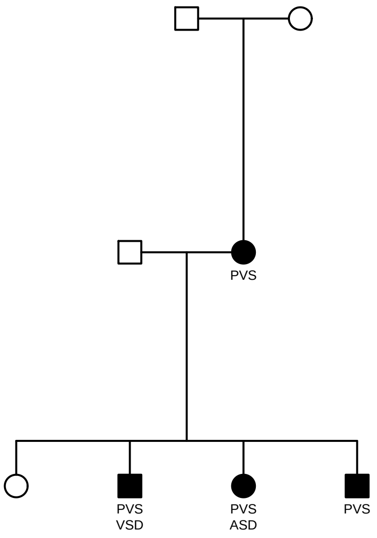

Klinge_1975 PMID:1111560

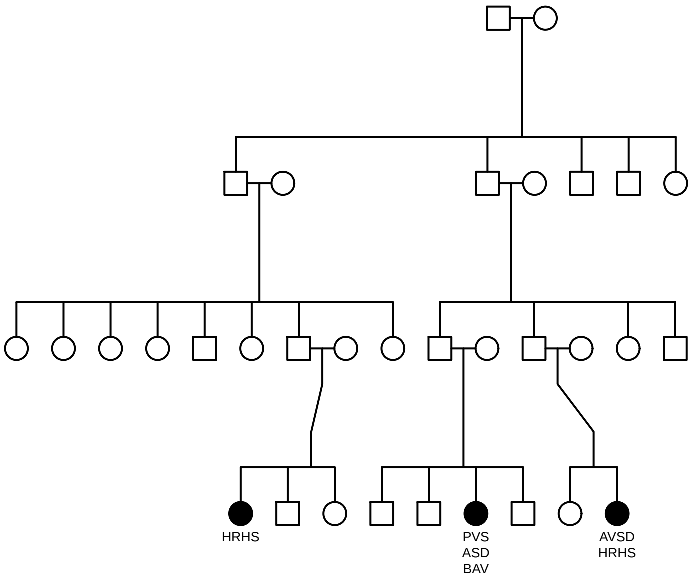

Friedberg_1974 PMID:4834778

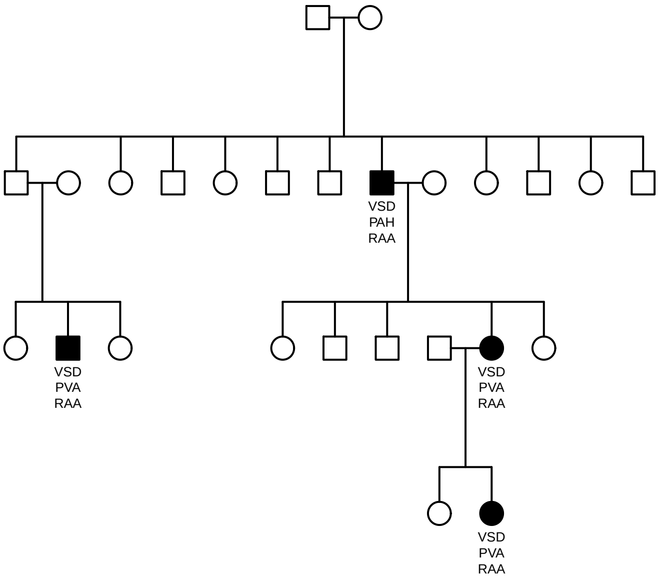

Schwartz_1993 PMID:8357105

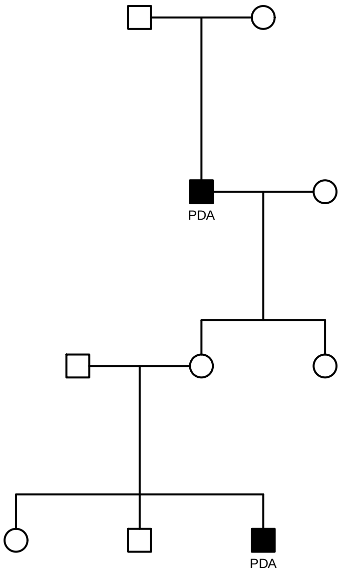

Burnell_1971 PMID:5095166

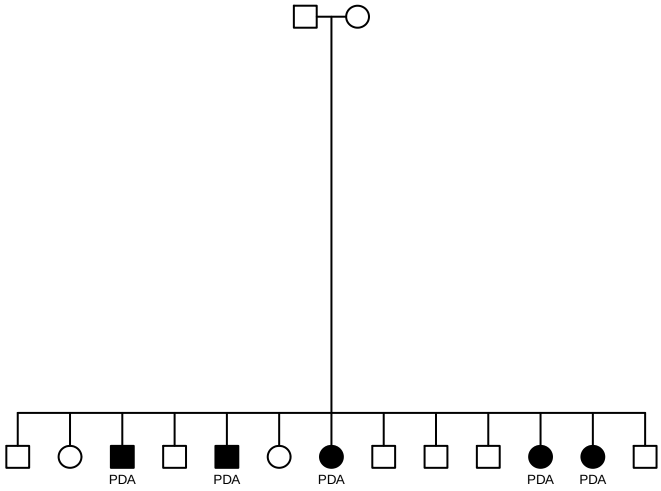

Polani_G79 PMID:13736684

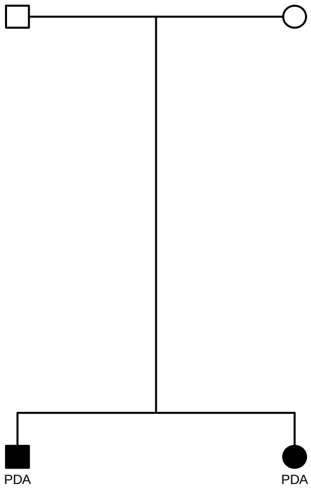

Polani_G65 PMID:13736684

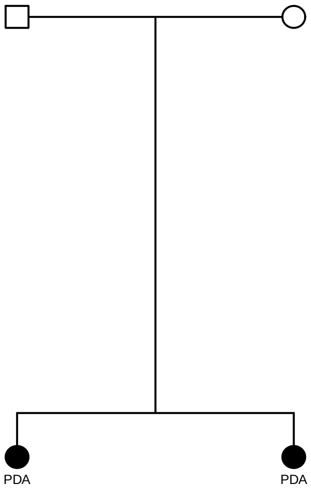

Polani_56 PMID:13736684

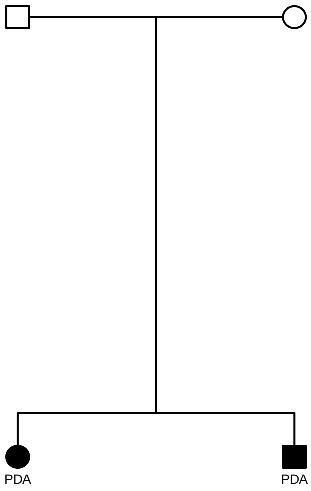

Polani_67 PMID:13736684

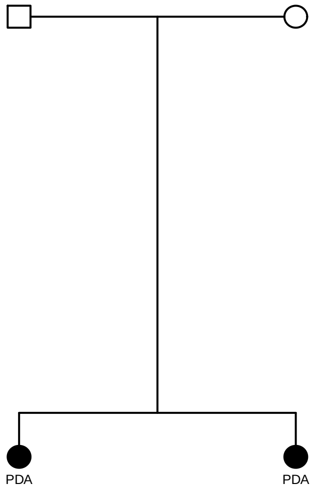

Hurwitz_1982 PMID:7172476

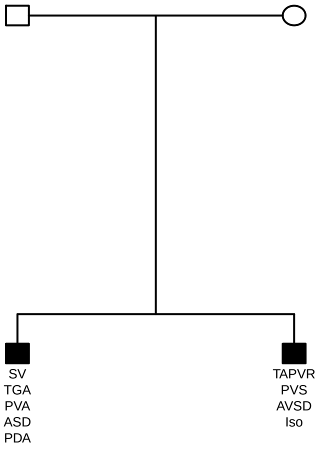

Zetterqvist_1960 PMID:13788275

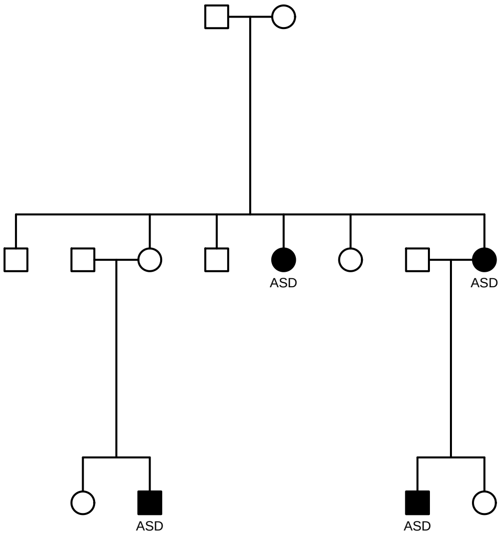

Lynch_1978 PMID:148839

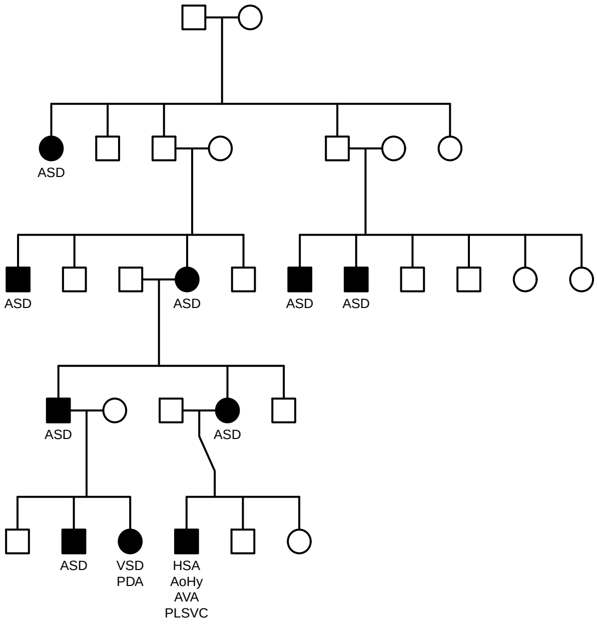

Okubo_2004 PMID:15235040

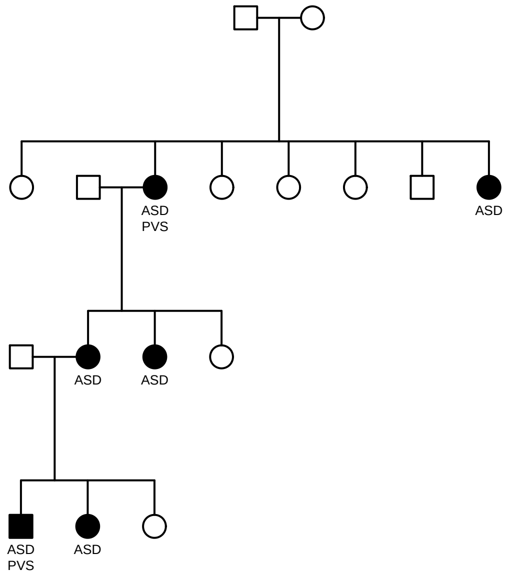

Kaufman_1972 PMID:Birth Defects: Original article series(1972):8;5

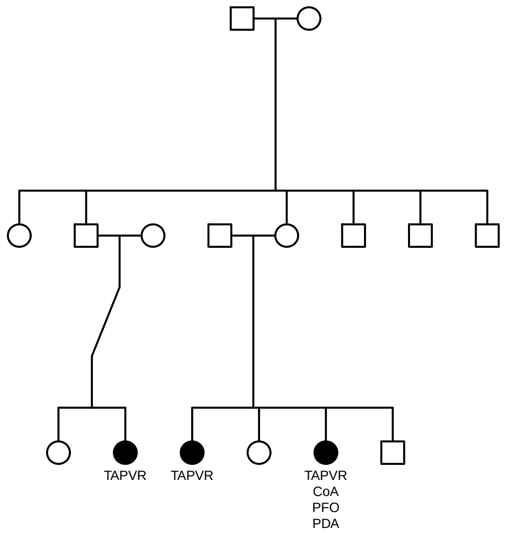

Petsas_1998 PMID:9474469

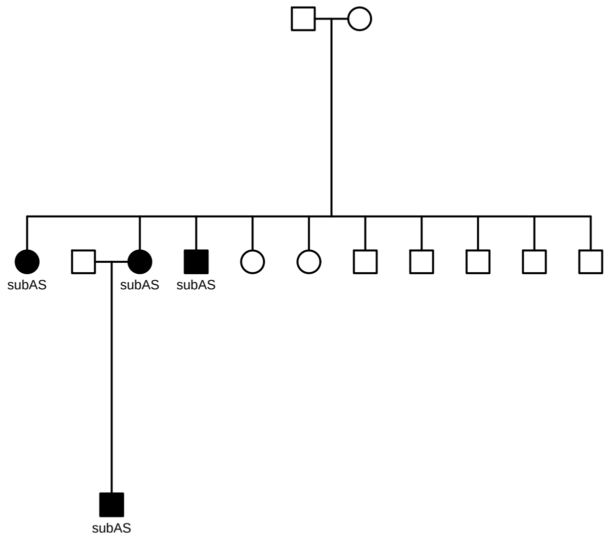

Vinh_1968 PMID:Arc Franc Ped 25:1141−9

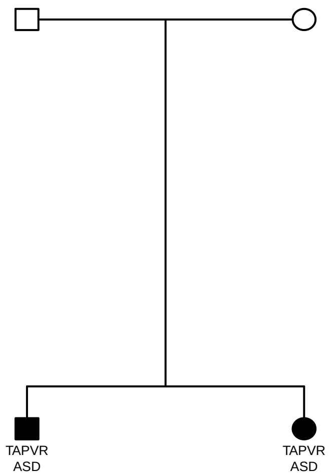

Shokeir_1974_1 PMID:4470892

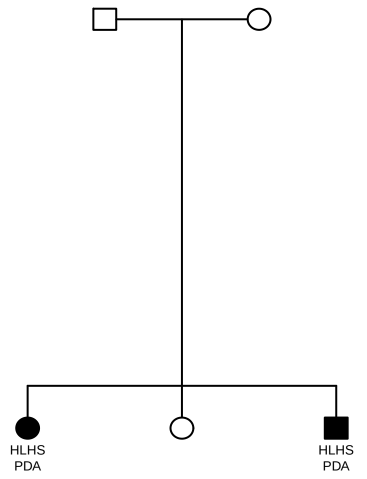

Shokeir_1974_2 PMID:4470892

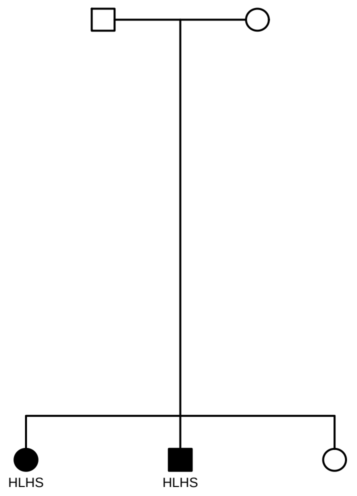

Shokeir_1974_3 PMID:4470892

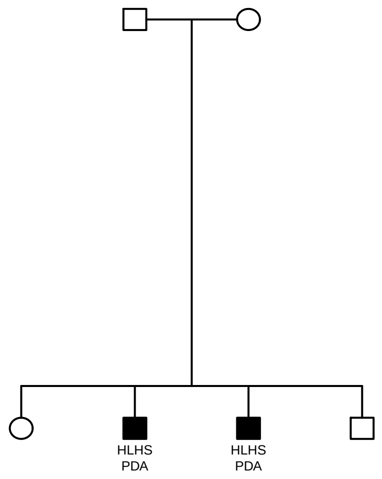

Shokeir_1974_4 PMID:4470892

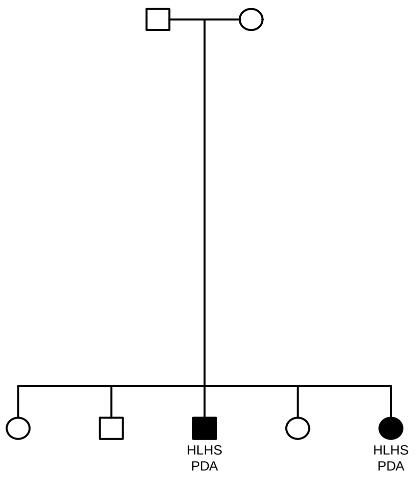

Shokeir_1974_5 PMID:4470892

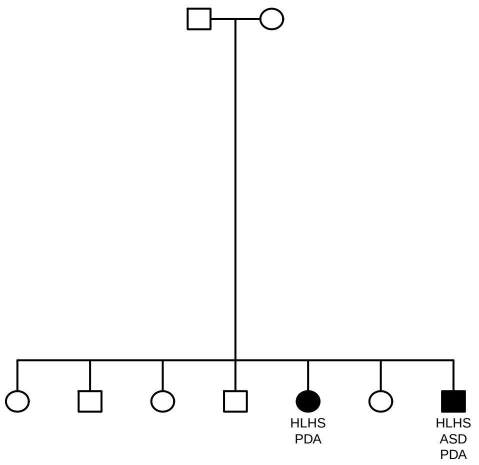

Hinton_1 PMID:17936159

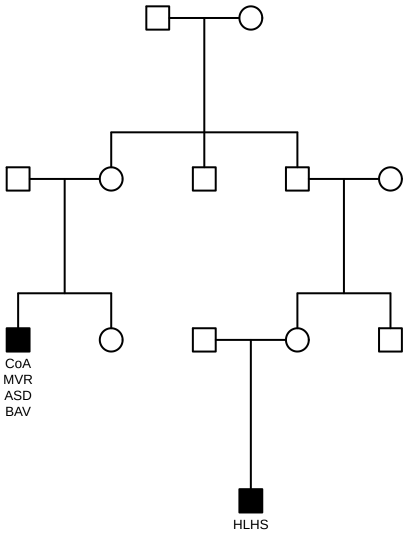

Hinton_2 PMID:17936159

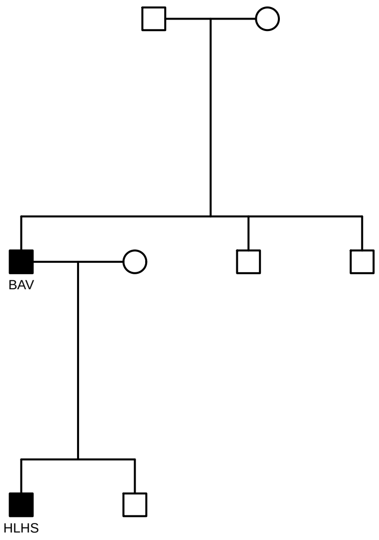

Hinton_3 PMID:17936159

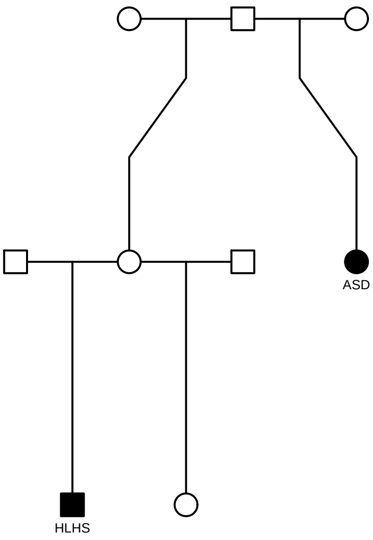

Hinton_4 PMID:17936159

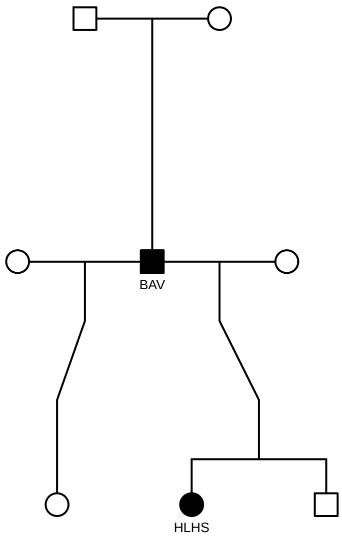

Hinton_5 PMID:17936159

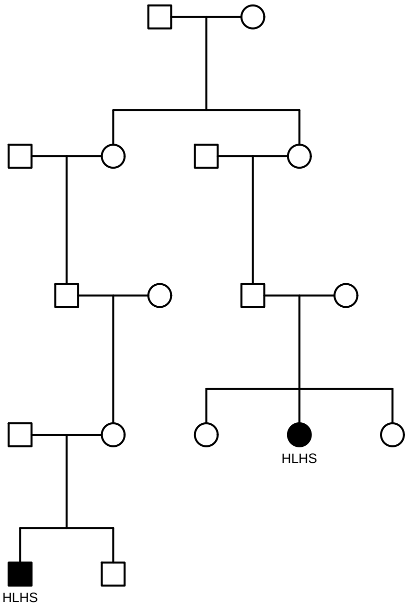

Hinton_6 PMID:17936159

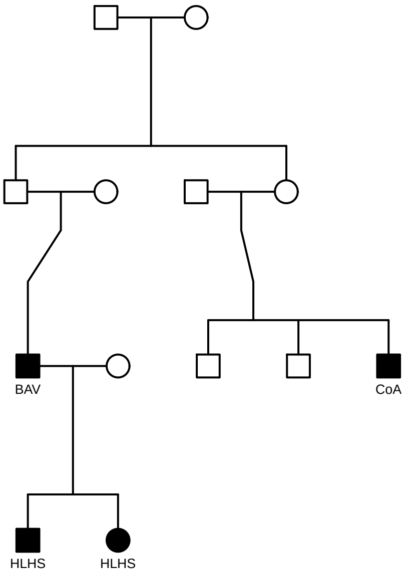

Hinton_7 PMID:17936159

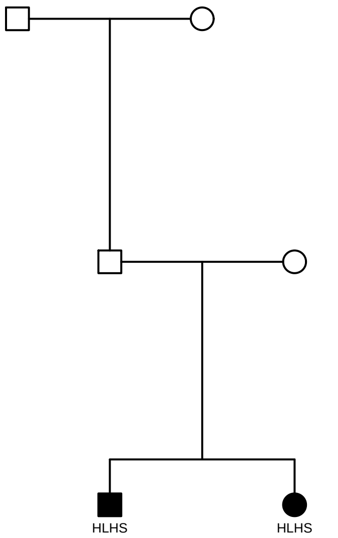

Hinton_8 PMID:17936159

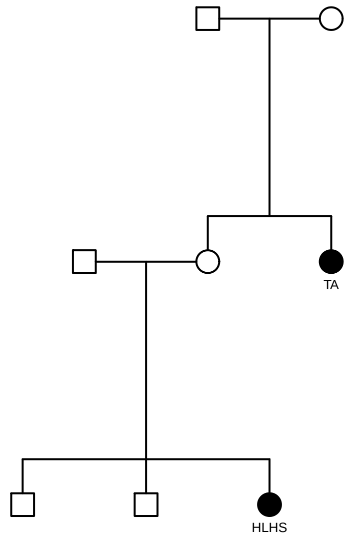

Hinton_9 PMID:17936159

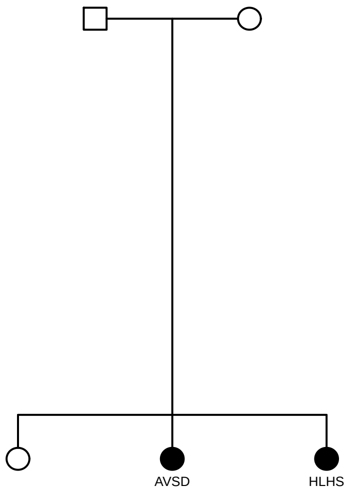

Hinton_10 PMID:17936159

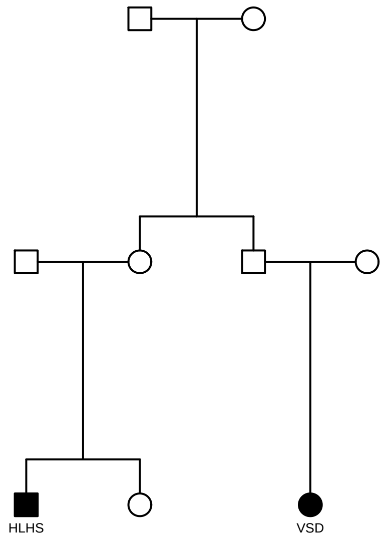

Hinton_11 PMID:17936159

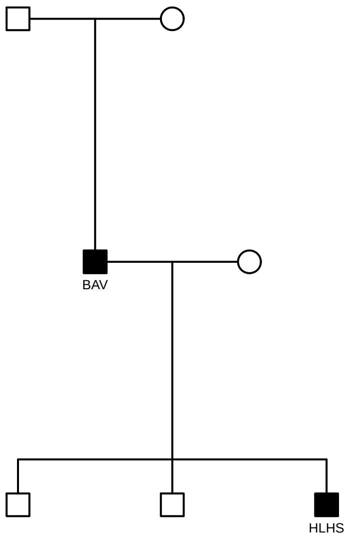

Hinton_12 PMID:17936159

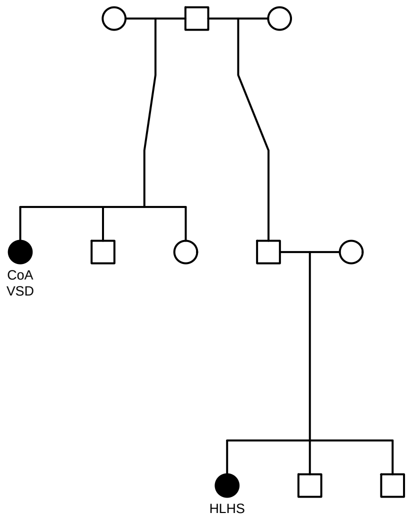

Hinton_13 PMID:17936159

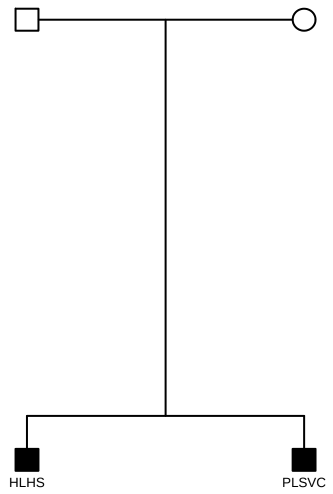

Hinton_14 PMID:17936159

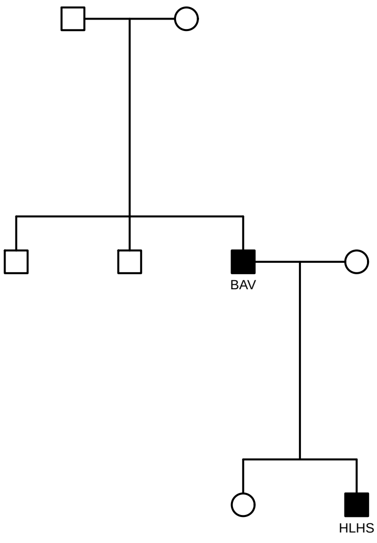

Hinton_15 PMID:17936159

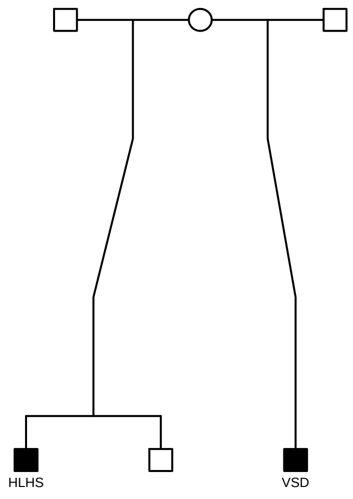

Hinton_16 PMID:17936159

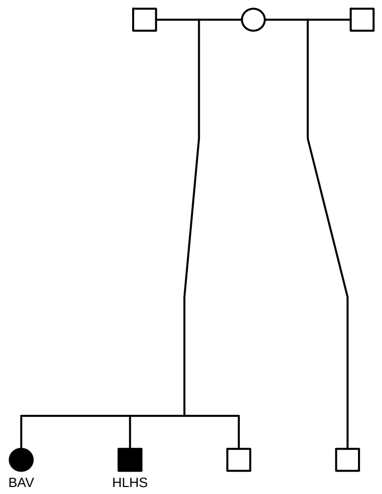

Hinton_17 PMID:17936159

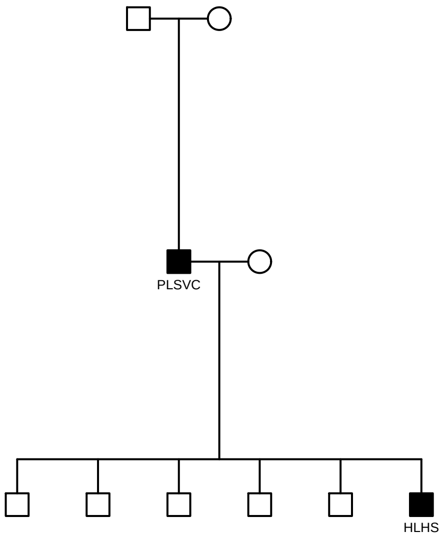

Hinton_18 PMID:17936159

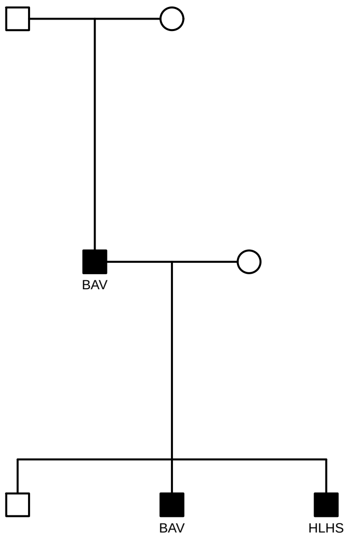

Hinton_19 PMID:17936159

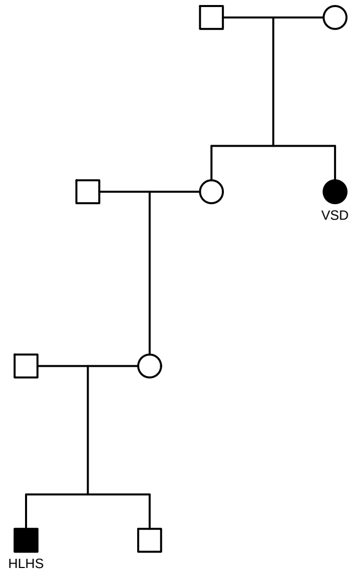

Hinton_21 PMID:17936159

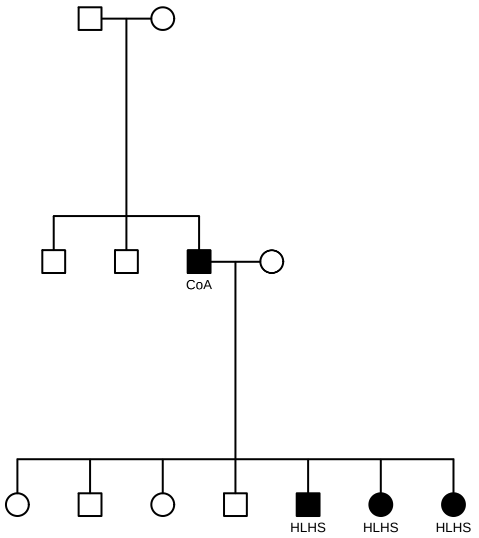

Alonso_4 PMID:7747776

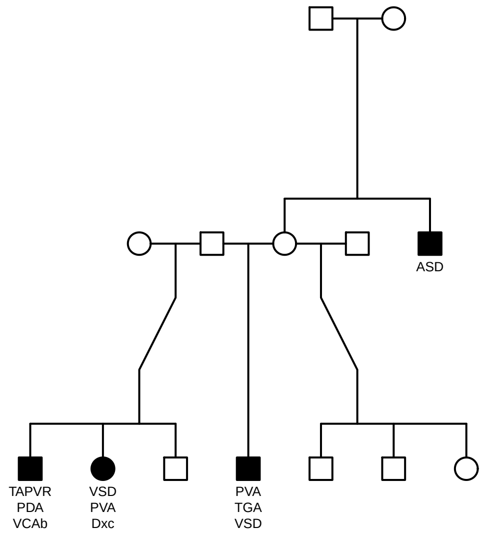

Boon_1972_2 PMID:5065286

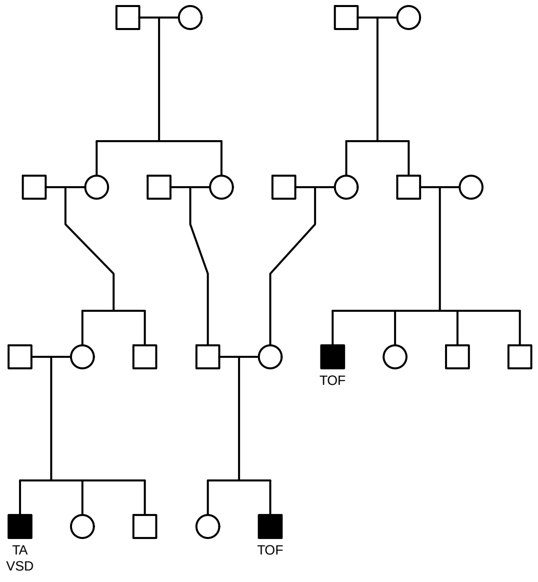

Brenner_3 PMID:2589285

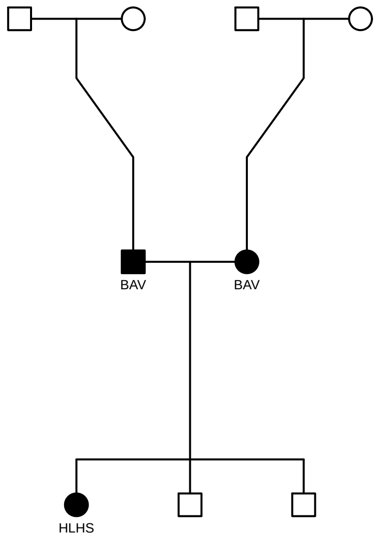

Hinton_20 PMID:17936159

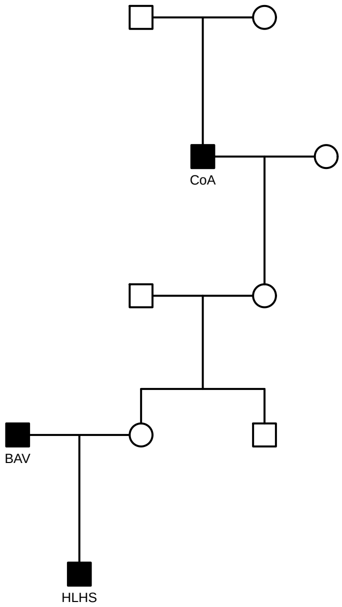

Wilmshurst_2004_1 PMID:15486131

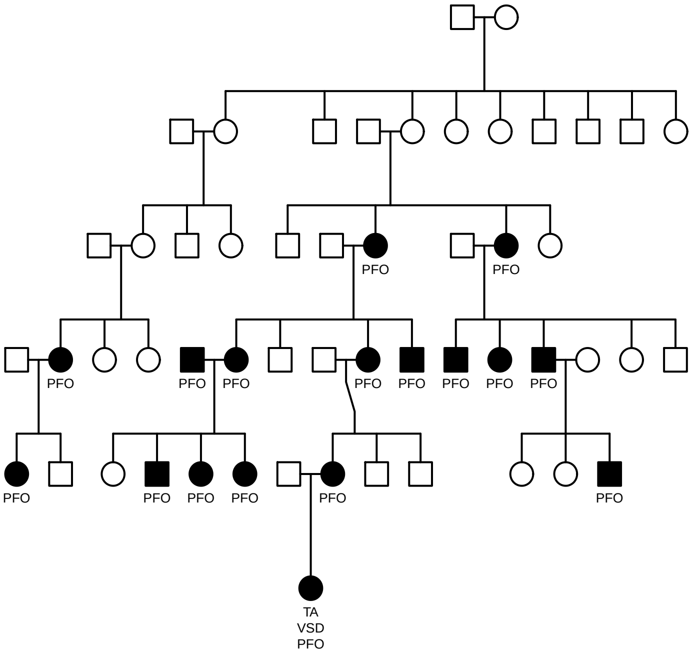

Abushaban_2003 PMID:12574981

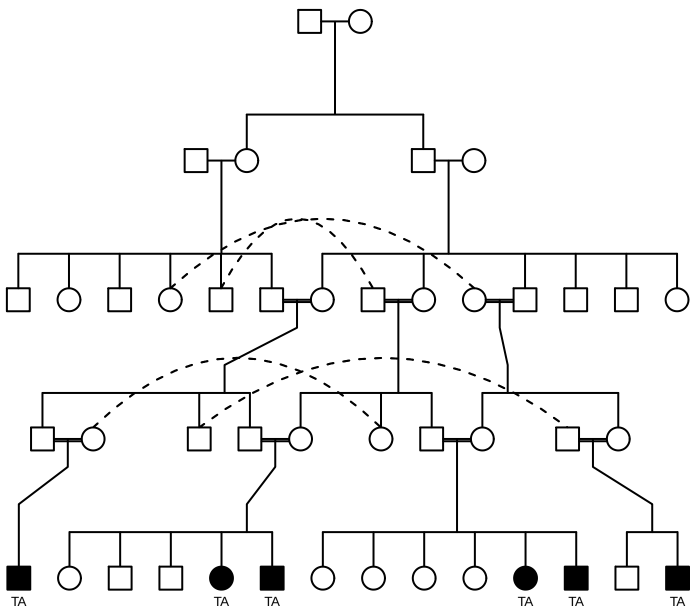

Arnold_1983 PMID:6638068

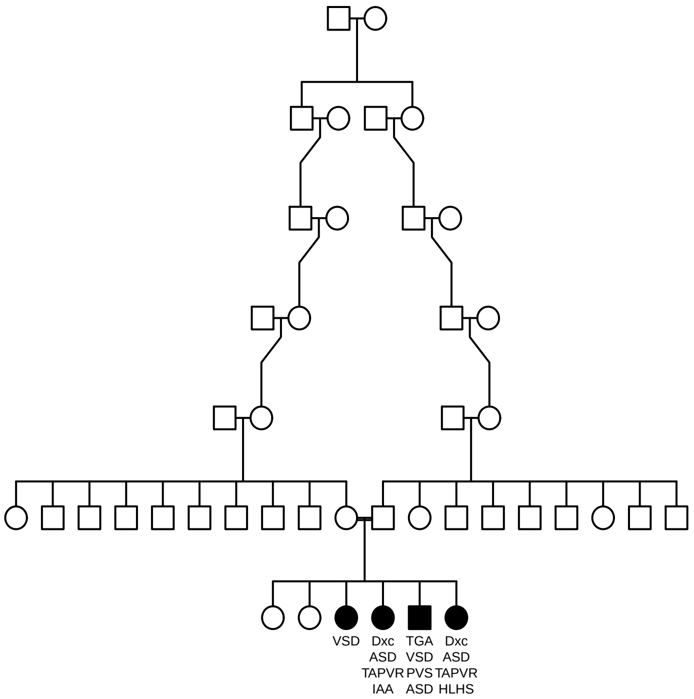

Digilio_1997_a PMID:9375931

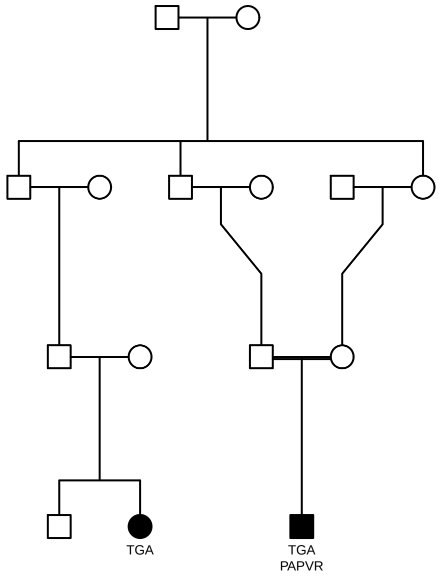

Disegni_4 PMID:4003317

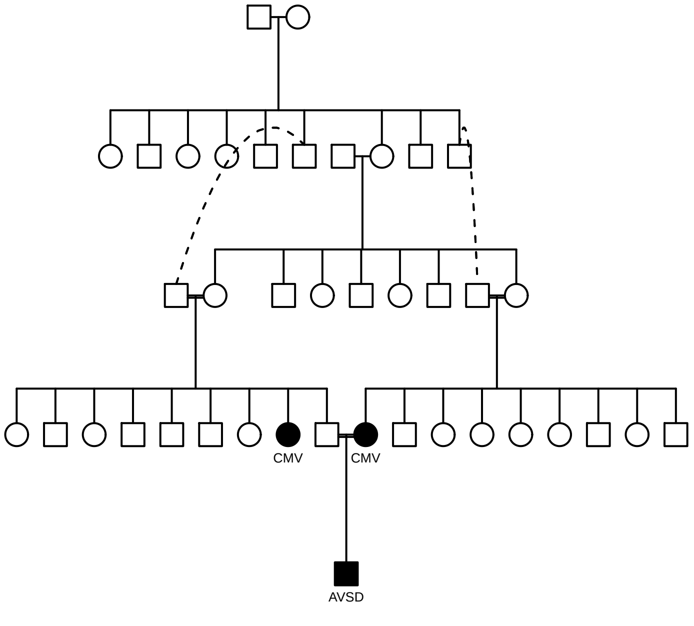

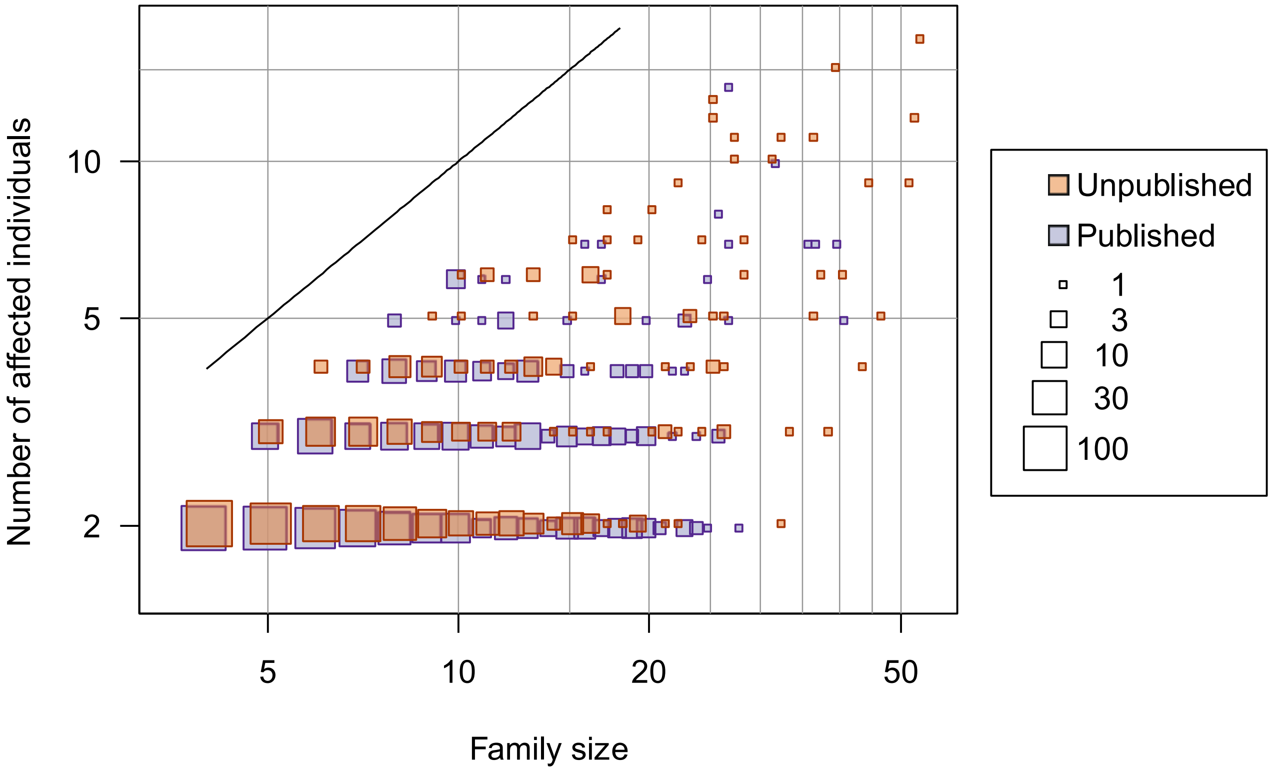

Supplemental Figure 4. Distribution of family size (total number of individuals in family) and number of affected individuals in the family, among 1,163 CHD families. Blue squares: *Published* families, Red squares: *Unpublished* families. The size of the square reflects the number of families. Note that this figure is based on individuals with clinically verified diagnoses. Numbers of non-validated individuals per family are listed in Supplemental table 3.

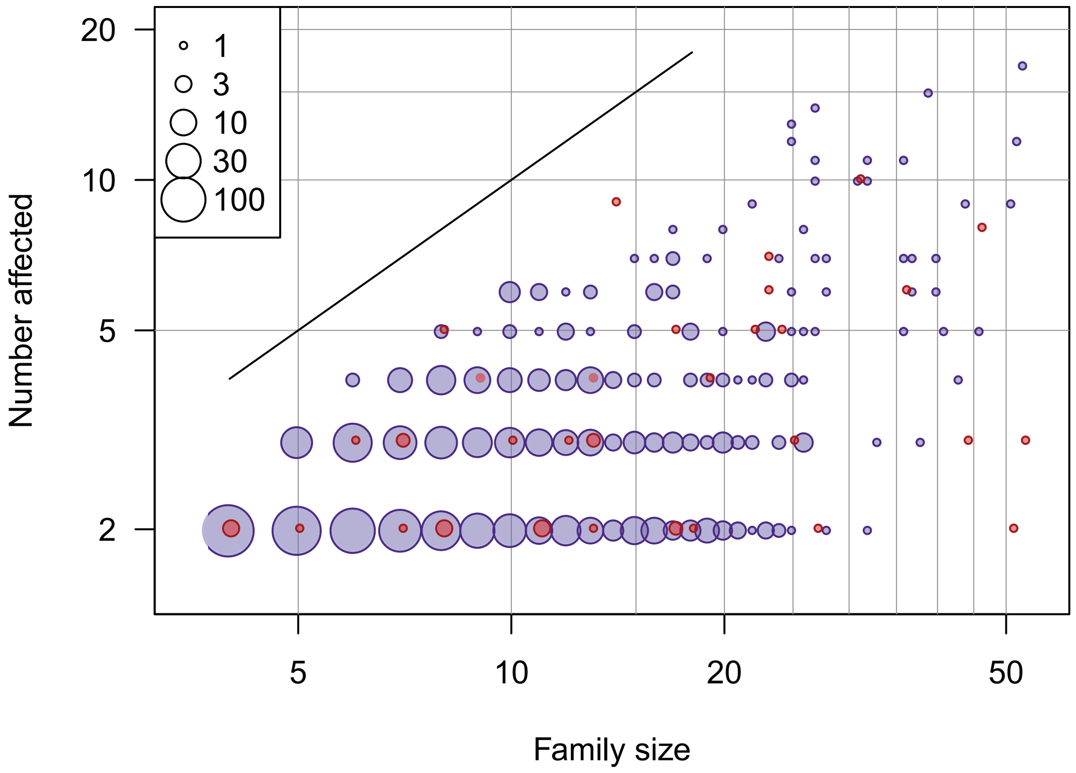

Supplemental Figure 5. Distribution of family size and affected individuals in families with and without verified diagnoses. Blue circles: families with verified diagnoses only. Red circles: families which include individuals with unverified diagnoses. Area of the circles represent the number of families.

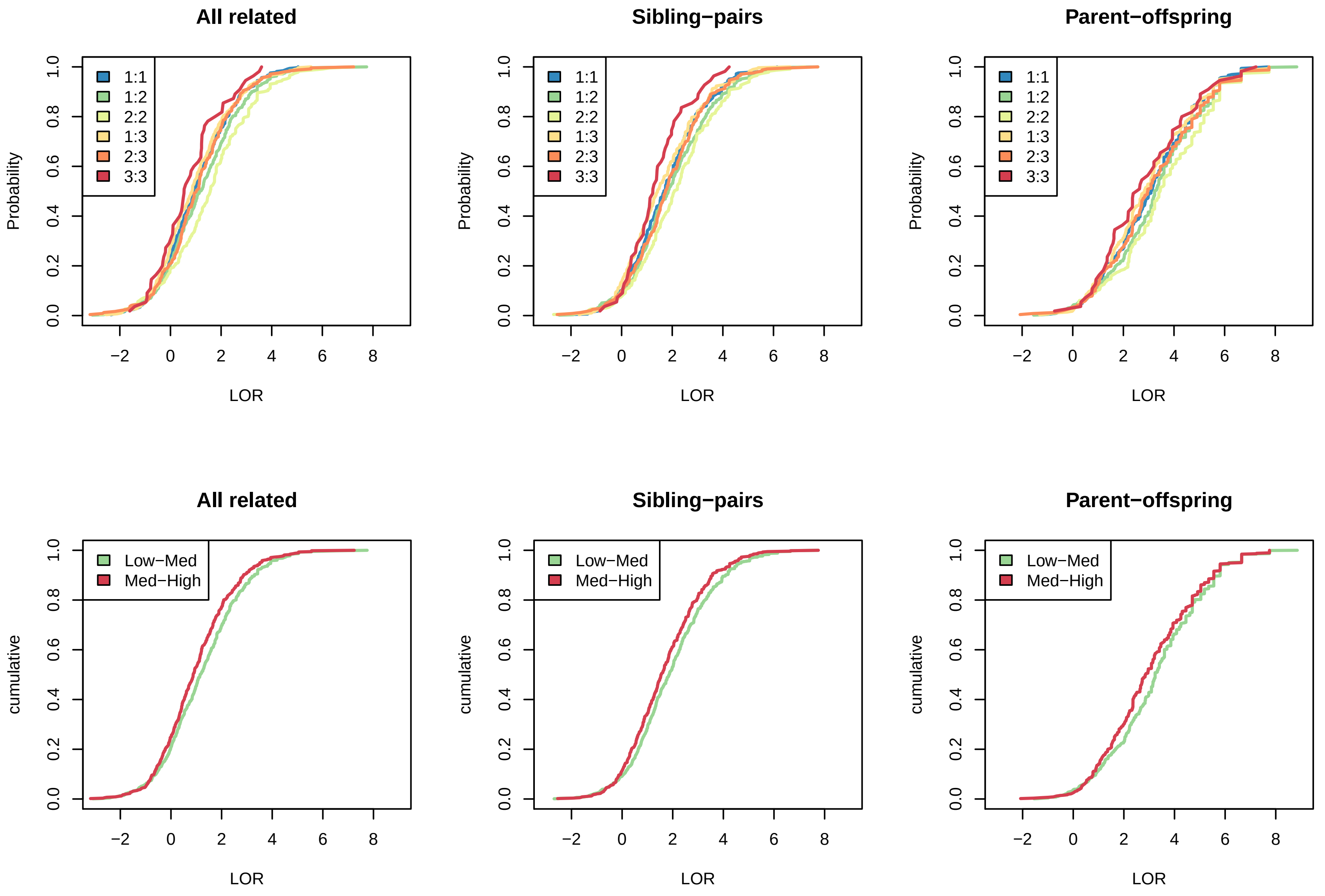

Supplemental Figure 6. Cumulative distribution of log-odds ratios. Upper panel: cumulative distribution of log-odds ratios calculated for six different groups of pairs, based on the severity of the malformations. Blue, green and pale yellow represent pairs with mild to intermediate severe malformations (combinations of score 1 and 2). Warm yellow, orange and red represent pairs with intermediate to severe malformations (combinations of score 2 and 3). Lower panel: cumulative distribution of log-odds ratios, calculated for two groups of phenotype-pairs based on the severity of the malformations. Red represent pairs where at least one of the two phenotypes is a severe malformation (combinations of severity scores with 3) while green curves represents pairs with mild to intermediate combinations of malformations (combinations of severity scores 1 and 2).

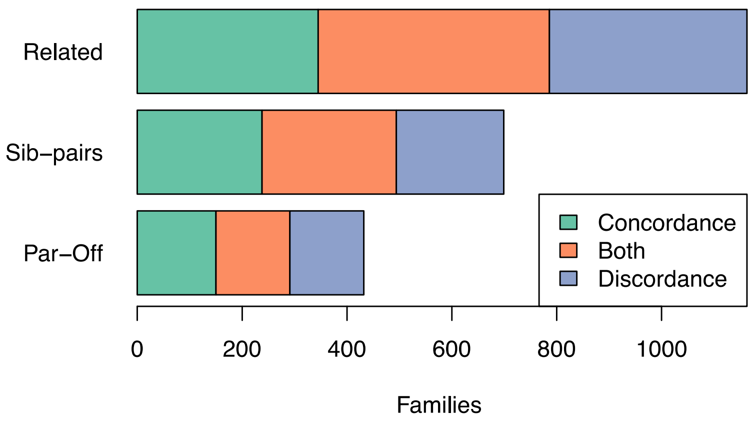

Supplemental Figure 7. Number or families with one or more concordant pairs, discordant pairs, or both for the 3 types of relationships. The total number of families (bar length) that exclusively show concordance (green), or exclusively discordance (blue), or both concordance and discordance (orange) can be compared for families with affected sibling-pairs or affected parent-offspring pairs. The proportions of concordance and discordance do not significantly differ by relationship type.

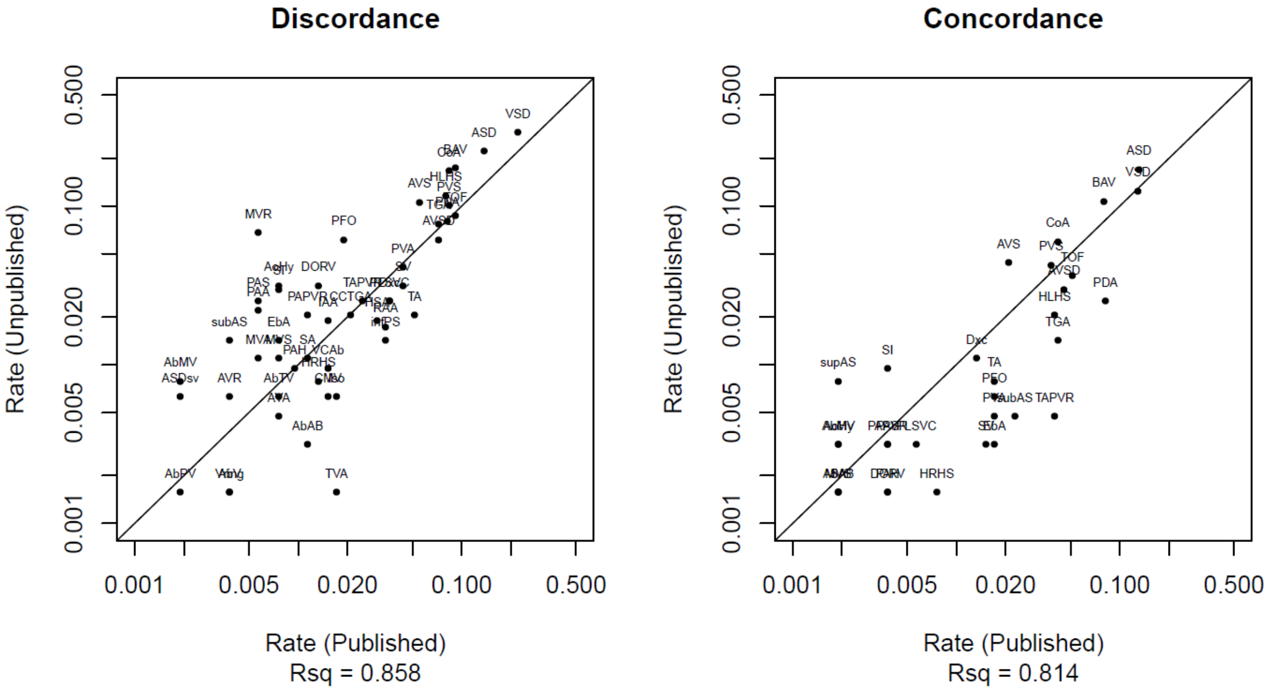

Supplemental Figure 8. Comparison of discordance and concordance rate between *Published* and *Unpublished* families. Abbreviations are listed in Supplemental Table 2.

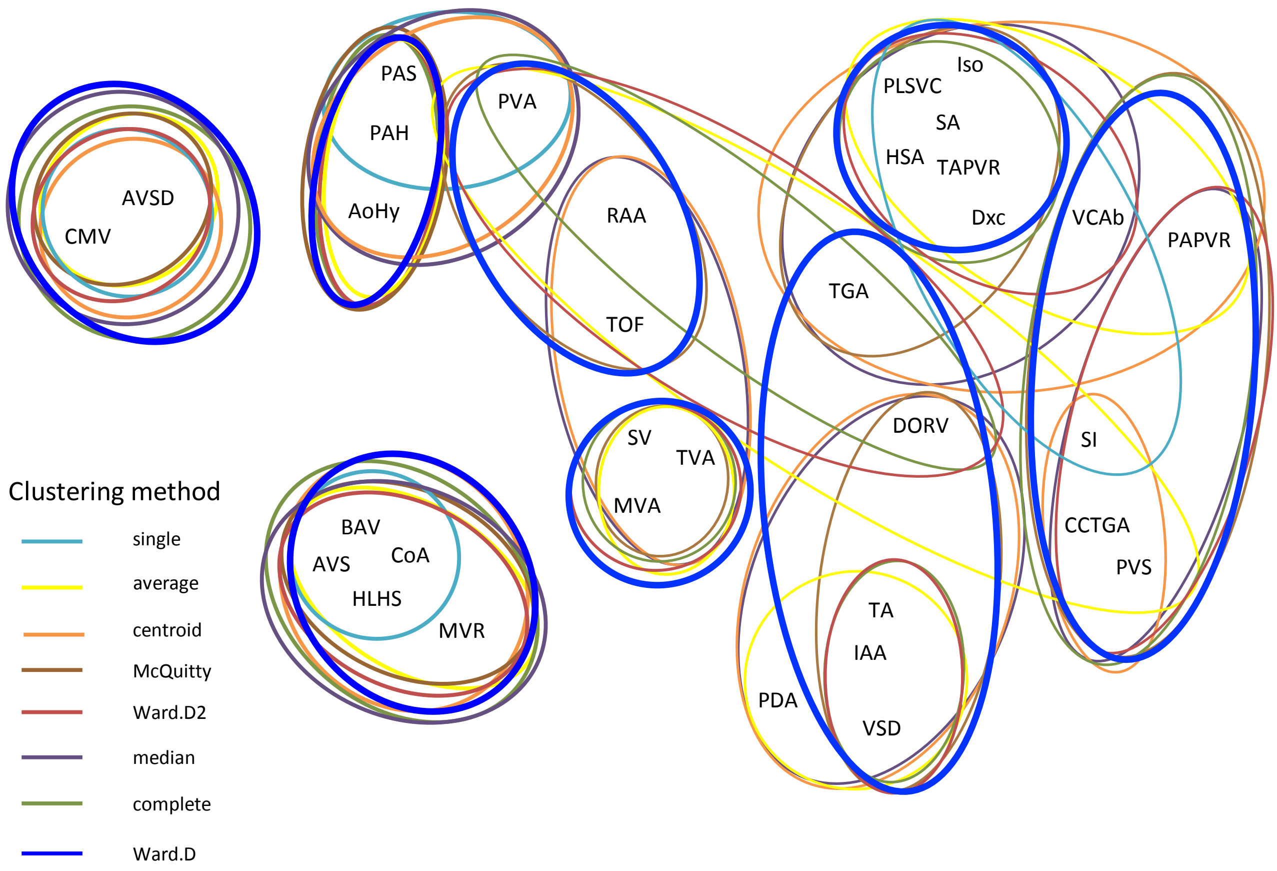

Supplemental Figure 9. Observed overlap of diagnoses between eight different clustering methods.

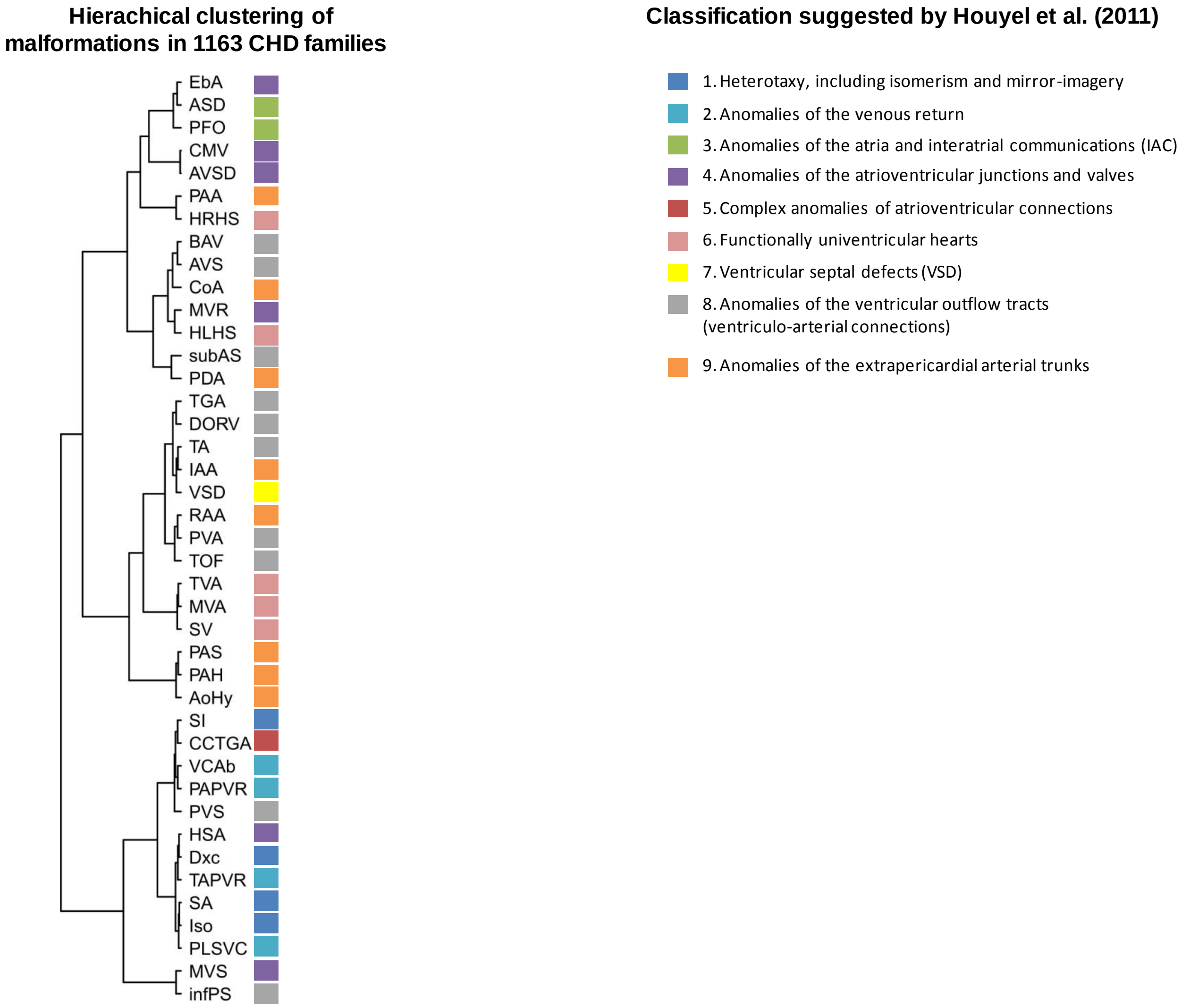

Supplemental Figure 10. Comparison between co-occurring groups of malformations in families and the classification of congenital heart defects proposed by Houyel et al. (2011). The dendrogram shown on the left side represent the grouping obtained using hierarchical clustering (Figure 4). The colour codes show the taxonomy group for each malformation as proposed by Houyel et al. (2011) [23].

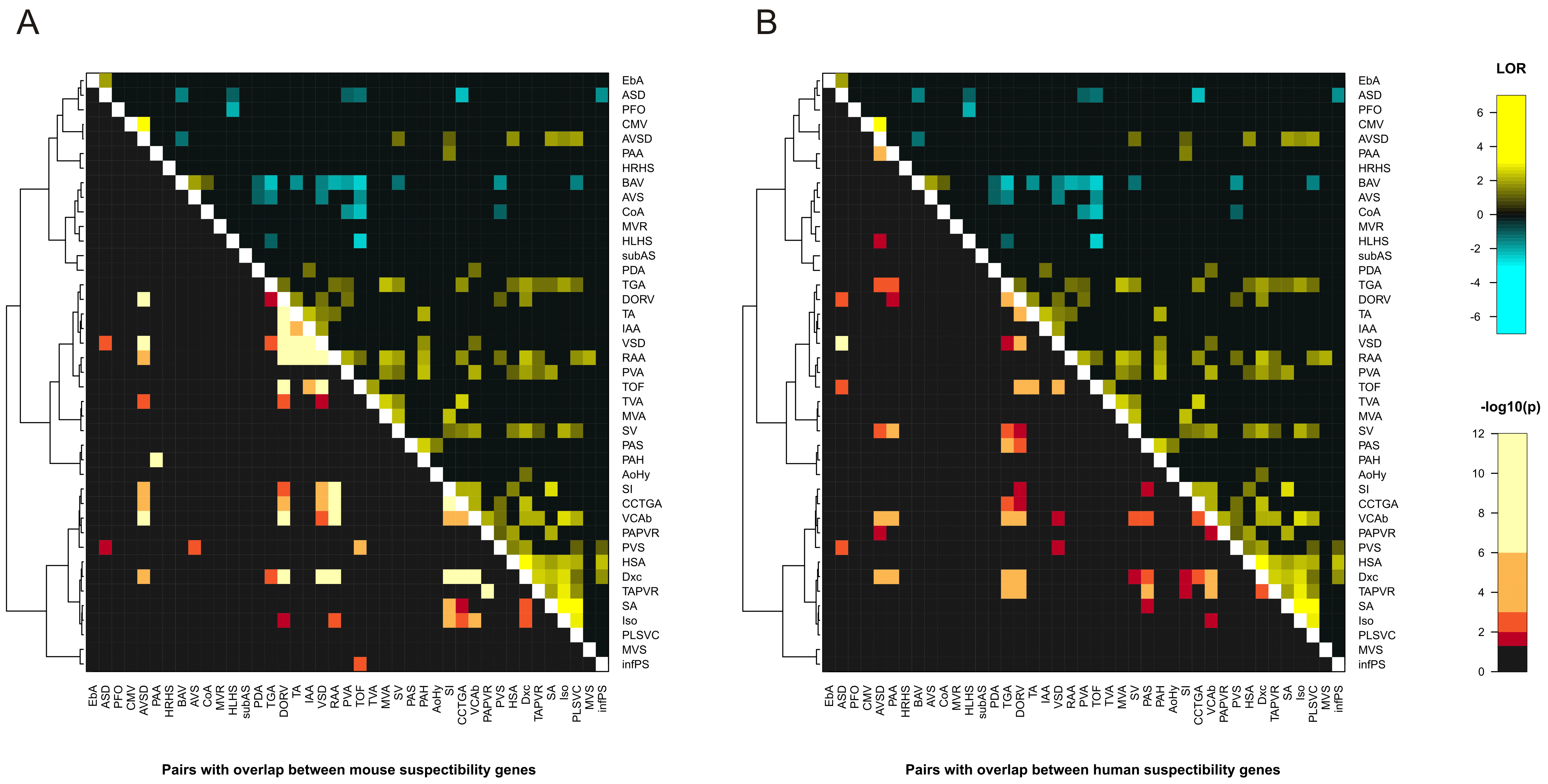

Supplemental Figure 11. LOR and significance of gene overlap in 1640 pairs of discordant cardiac malformations. The data was arranged by hierarchical cluster analysis according to LOR (LOR is shown in yellow-black-cyan at upper-right part of figure). The significance of overlapping mouse (A) and human (B) susceptibility genes between pairs of malformations was calculated using Fisher's exact test,with adjustment for multiple testing (values shown in black-red-yellow at lower-left part of figure).

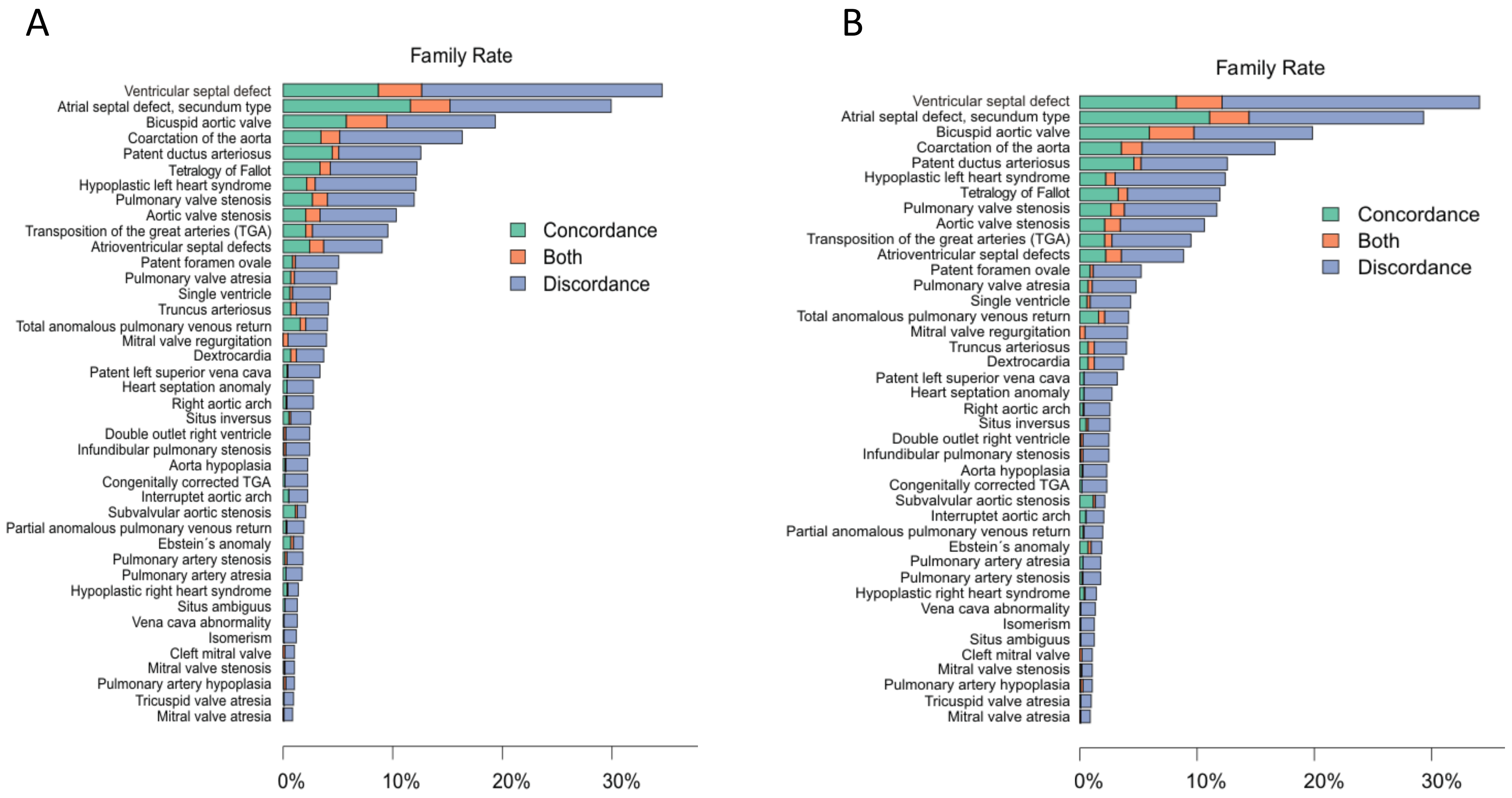

Supplemental Figure 12. Concordance and discordance in CHD families. A. Full dataset. B. Dataset without families with known monogenic cause

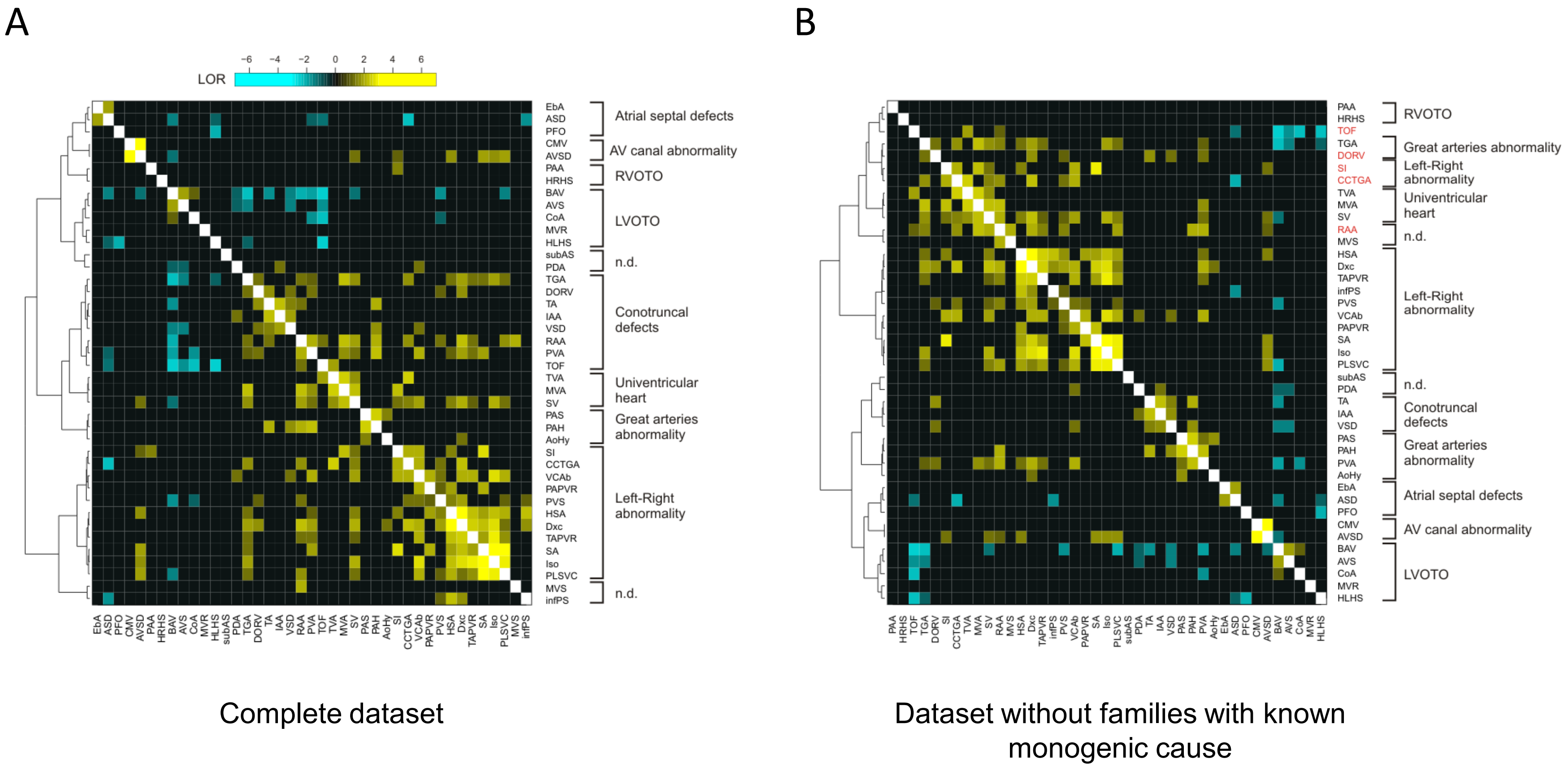

Supplemental Figure 13. Comparison of heatmaps. A. Clustering of complete dataset. B. Clustering of dataset without families with a known monogenic cause.

Supplemental references

1. Rickert-Sperling S, Kelly RG, Driscoll D. Congenital Heart Diseases: The Broken Heart. Wien: Springer-verlag; 2016

2. Chang SW, Mislankar M, Misra C, Huang N, Dajusta DG, Harrison SM, McBride KL, Baker LA, Garg V. Genetic abnormalities in FOXP1 are associated with congenital heart defects. Hum Mutat. 2013, 34(9):1226-30.

3. Maitra M, Koenig SN, Srivastava D, Garg V. Identification of GATA6 sequence variants in patients with congenital heart defects. Pediatr Res. 2010, 68(4):281-5.

4. Kosaki K, Bassi MT, Kosaki R, Lewin M, Belmont J, Schauer G, Casey B. Characterization and mutation analysis of human LEFTY A and LEFTY B, homologues of murine genes implicated in left-right axis development. Am J Hum Genet. 1999, 64(3):712-21.

5. Ma L, Selamet Tierney ES, Lee T, Lanzano P, Chung WK. Mutations in ZIC3 and ACVR2B are a common cause of heterotaxy and associated cardiovascular anomalies. Cardiol Young. 2012, 22(2):194-201.

6. Mohapatra B, Casey B, Li H, Ho-Dawson T, Smith L, Fernbach SD, Molinari L, Niesh SR, Jefferies JL, Craigen WJ, Towbin JA, Belmont JW, Ware SM. Identification and functional characterization of NODAL rare variants in heterotaxy and isolated cardiovascular malformations. Hum Mol Genet. 2009, 18(5):861-71.

7. Robinson SW, Morris CD, Goldmuntz E, Reller MD, Jones MA, Steiner RD, Maslen CL. Missense mutations in CRELD1 are associated with cardiac atrioventricular septal defects. Am J Hum Genet. 2003, 72(4):1047-52.

8. Posch MG, Perrot A, Schmitt K, Mittelhaus S, Esenwein EM, Stiller B, Geier C, Dietz R, Gessner R, Ozcelik C, Berger F. Mutations in GATA4, NKX2.5, CRELD1, and BMP4 are infrequently found in patients with congenital cardiac septal defects. Am J Med Genet A. 2008, 146A(2):251-3.

9. Dentici ML, Sarkozy A, Pantaleoni F, Carta C, Lepri F, Ferese R, Cordeddu V, Martinelli S, Briuglia S, Digilio MC, Zampino G, Tartaglia M, Dallapiccola B. Spectrum of MEK1 and MEK2 gene mutations in cardio-facio-cutaneous syndrome and genotype-phenotype correlations. Eur J Hum Genet. 2009, 17(6):733-40.

10. Kosaki R, Gebbia M, Kosaki K, Lewin M, Bowers P, Towbin JA, Casey B. Left-right axis malformations associated with mutations in ACVR2B, the gene for human activin receptor type IIB. Am J Med Genet. 1999, 82(1):70-6.

11. Kaasinen E, Aittomäki K, Eronen M, Vahteristo P, Karhu A, Mecklin JP, Kajantie E, Aaltonen LA, Lehtonen R. Recessively inherited right atrial isomerism caused by mutations in growth/differentiation factor 1 (GDF1). Hum Mol Genet. 2010, 19(14):2747-53.

12. Izumi K, Noon S, Wilkens A, Krantz ID. NKX2.5 mutation identification on exome sequencing in a patient with heterotaxy. Eur J Med Genet. 2014 Oct;57(10):558-61.

13. Ware SM, Peng J, Zhu L, Fernbach S, Colicos S, Casey B, Towbin J, Belmont JW. Identification and functional analysis of ZIC3 mutations in heterotaxy and related congenital heart defects. Am J Hum Genet. 2004, 74(1):93-105.

14. Bamford RN, Roessler E, Burdine RD, Saplakoğlu U, dela Cruz J, Splitt M, Goodship JA, Towbin J, Bowers P, Ferrero GB, Marino B, Schier AF, Shen MM, Muenke M, Casey B. Loss-of-function mutations in the EGF-CFC gene CFC1 are associated with human left-right laterality defects. Nat Genet. 2000, 26(3):365-9.

15. Sifrim A, Hitz MP, Wilsdon A, Breckpot J, Turki SH, Thienpont B, McRae J, Fitzgerald TW, Singh T, Swaminathan GJ, Prigmore E, Rajan D, Abdul-Khaliq H, Banka S, Bauer UM, Bentham J, Berger F, Bhattacharya S, Bu'Lock F, Canham N, Colgiu IG, Cosgrove C, Cox H, Daehnert I, Daly A, Danesh J, Fryer A, Gewillig M, Hobson E, Hoff K, Homfray T; INTERVAL Study, Kahlert AK, Ketley A, Kramer HH, Lachlan K, Lampe AK, Louw JJ, Manickara AK, Manase D, McCarthy KP, Metcalfe K, Moore C, Newbury-Ecob R, Omer SO, Ouwehand WH, Park SM, Parker MJ, Pickardt T, Pollard MO, Robert L, Roberts DJ, Sambrook J, Setchfield K, Stiller B, Thornborough C, Toka O, Watkins H, Williams D, Wright M, Mital S, Daubeney PE, Keavney B, Goodship J; UK10K Consortium, Abu-Sulaiman RM, Klaassen S, Wright CF, Firth HV, Barrett JC, Devriendt K, FitzPatrick DR, Brook JD; Deciphering Developmental Disorders Study, Hurles ME. Distinct genetic architectures for syndromic and nonsyndromic congenital heart defects identified by exome sequencing. Nat Genet. 2016 [Epub ahead of print].

16. Degenhardt K, Singh MK, Aghajanian H, Massera D, Wang Q, Li J, Li L, Choi C, Yzaguirre AD, Francey LJ, Gallant E, Krantz ID, Gruber PJ, Epstein JA. Semaphorin 3d signaling defects are associated with anomalous pulmonary venous connections. Nat Med. 2013, 19(6):760-5.

17. Bleyl SB, Saijoh Y, Bax NA, Gittenberger-de Groot AC, Wisse LJ, Chapman SC, Hunter J, Shiratori H, Hamada H, Yamada S, Shiota K, Klewer SE, Leppert MF, Schoenwolf GC. Dysregulation of the PDGFRA gene causes inflow tract anomalies including TAPVR: integrating evidence from human genetics and model organisms. Hum Mol Genet. 2010, 19(7):1286-301.

18. Cinquetti R, Badi I, Campione M, Bortoletto E, Chiesa G, Parolini C, Camesasca C, Russo A, Taramelli R, Acquati F. Transcriptional deregulation and a missense mutation define ANKRD1 as a candidate gene for total anomalous pulmonary venous return. Hum Mutat. 2008, 29(4):468-74.

19. Guichard C, Harricane MC, Lafitte JJ, Godard P, Zaegel M, Tack V, Lalau G, Bouvagnet P. Axonemal dynein intermediate-chain gene (DNAI1) mutations result in situs inversus and primary ciliary dyskinesia (Kartagener syndrome). Am J Hum Genet. 2001, 68(4):1030-5.

20. Tariq M, Belmont JW, Lalani S, Smolarek T, Ware SM. SHROOM3 is a novel candidate for heterotaxy identified by whole exome sequencing. Genome Biol. 2011, 12:R91.

21. Garg V, Muth AN, Ransom JF, Schluterman MK, Barnes R, King IN, Grossfeld PD, Srivastava D. Mutations in NOTCH1 cause aortic valve disease. Nature. 2005, 437(7056):270-4.

22. Mohamed SA, Aherrahrou Z, Liptau H, Erasmi AW, Hagemann C, Wrobel S, Borzym K, Schunkert H, Sievers HH, Erdmann J. Novel missense mutations (p.T596M and p.P1797H) in NOTCH1 in patients with bicuspid aortic valve. Biochem Biophys Res Commun. 2006, 345(4):1460-5.

23. McElhinney DB, Krantz ID, Bason L, Piccoli DA, Emerick KM, Spinner NB, Goldmuntz E. Analysis of cardiovascular phenotype and genotype-phenotype correlation in individuals with a JAG1 mutation and/or Alagille syndrome. Circulation. 2002, 106(20):2567-74.

24. Oda T, Elkahloun AG, Pike BL, Okajima K, Krantz ID, Genin A, Piccoli DA, Meltzer PS, Spinner NB, Collins FS, Chandrasekharappa SC. Mutations in the human Jagged1 gene are responsible for Alagille syndrome. Nat Genet. 1997, 16(3):235-42.

25. McDaniell R, Warthen DM, Sanchez-Lara PA, Pai A, Krantz ID, Piccoli DA, Spinner NB. NOTCH2 mutations cause Alagille syndrome, a heterogeneous disorder of the notch signaling pathway. Am J Hum Genet. 2006, 79(1):169-73.

26. Kamath BM, Bauer RC, Loomes KM, Chao G, Gerfen J, Hutchinson A, Hardikar W, Hirschfield G, Jara P, Krantz ID, Lapunzina P, Leonard L, Ling S, Ng VL, Hoang PL, Piccoli DA, Spinner NB. NOTCH2 mutations in Alagille syndrome. J Med Genet. 2012, 49(2):138-44.

27. Tartaglia M, Mehler EL, Goldberg R, Zampino G, Brunner HG, Kremer H, van der Burgt I, Crosby AH, Ion A, Jeffery S, Kalidas K, Patton MA, Kucherlapati RS, Gelb BD. Mutations in PTPN11, encoding the protein tyrosine phosphatase SHP-2, cause Noonan syndrome. Nat Genet. 2001, 29(4):465-8.

28. Ng SB, Bigham AW, Buckingham KJ, Hannibal MC, McMillin MJ, Gildersleeve HI, Beck AE, Tabor HK, Cooper GM, Mefford HC, Lee C, Turner EH, Smith JD, Rieder MJ, Yoshiura K, Matsumoto N, Ohta T, Niikawa N, Nickerson DA, Bamshad MJ, Shendure J. Exome sequencing identifies MLL2 mutations as a cause of Kabuki syndrome. Nat Genet. 2010, 42(9):790-3.

29. Tan HL, Glen E, Töpf A, Hall D, O'Sullivan JJ, Sneddon L, Wren C, Avery P, Lewis RJ, ten Dijke P, Arthur HM, Goodship JA, Keavney BD. Nonsynonymous variants in the SMAD6 gene predispose to congenital cardiovascular malformation. Hum Mutat. 2012, 33(4):720-7.

30. Corsten-Janssen N, Kerstjens-Frederikse WS, du Marchie Sarvaas GJ, Baardman ME, Bakker MK, Bergman JE, Hove HD, Heimdal KR, Rustad CF, Hennekam RC, Hofstra RM, Hoefsloot LH, Van Ravenswaaij-Arts CM, Kapusta L. The cardiac phenotype in patients with a CHD7 mutation. Circ Cardiovasc Genet. 2013, 6(3):248-54.

31. Kodo K, Nishizawa T, Furutani M, Arai S, Ishihara K, Oda M, Makino S, Fukuda K, Takahashi T, Matsuoka R, Nakanishi T, Yamagishi H. Genetic analysis of essential cardiac transcription factors in 256 patients with non-syndromic congenital heart defects. Circ J. 2012, 76(7):1703-11.

32. Abdul-Sater Z, Yehya A, Beresian J, Salem E, Kamar A, Baydoun S, Shibbani K, Soubra A, Bitar F, Nemer G. Two heterozygous mutations in NFATC1 in a patient with Tricuspid Atresia. PLoS One. 2012, 7(11):e49532.

33. Ellesøe SG, Johansen MM, Bjerre JV, Hjortdal VE, Brunak S, Larsen LA. Familial Atrial Septal Defect and Sudden Cardiac Death: Identification of a Novel NKX2-5 Mutation and a Review of the Literature. Congenit Heart Dis. 2016, 11(3):283-90.

34. Granados-Riveron JT, Ghosh TK, Pope M, Bu'Lock F, Thornborough C, Eason J, Kirk EP, Fatkin D, Feneley MP, Harvey RP, Armour JA, David Brook J. Alpha-cardiac myosin heavy chain (MYH6) mutations affecting myofibril formation are associated with congenital heart defects. Hum Mol Genet. 2010, 19(20):4007-16.

35. Stallmeyer B, Fenge H, Nowak-Göttl U, Schulze-Bahr E. Mutational spectrum in the cardiac transcription factor gene NKX2.5 (CSX) associated with congenital heart disease. Clin Genet. 2010, 78(6):533-40.

36. Benson DW, Silberbach GM, Kavanaugh-McHugh A, Cottrill C, Zhang Y, Riggs S, Smalls O, Johnson MC, Watson MS, Seidman JG, Seidman CE, Plowden J, Kugler JD. Mutations in the cardiac transcription factor NKX2.5 affect diverse cardiac developmental pathways. J Clin Invest. 1999, 104(11):1567-73.

37. Postma AV, van Engelen K, van de Meerakker J, Rahman T, Probst S, Baars MJ, Bauer U, Pickardt T, Sperling SR, Berger F, Moorman AF, Mulder BJ, Thierfelder L, Keavney B, Goodship J, Klaassen S. Mutations in the sarcomere gene MYH7 in Ebstein anomaly. Circ Cardiovasc Genet. 2011, 4(1):43-50.

38. McBride KL, Riley MF, Zender GA, Fitzgerald-Butt SM, Towbin JA, Belmont JW, Cole SE. NOTCH1 mutations in individuals with left ventricular outflow tract malformations reduce ligand-induced signaling. Hum Mol Genet. 2008, 17(18):2886-93.

39. Blue GM, Kirk EP, Giannoulatou E, Dunwoodie SL, Ho JW, Hilton DC, White SM, Sholler GF, Harvey RP, Winlaw DS. Targeted next-generation sequencing identifies pathogenic variants in familial congenital heart disease. J Am Coll Cardiol. 2014, 64(23):2498-506.

40. Zhu L, Vranckx R, Khau Van Kien P, Lalande A, Boisset N, Mathieu F, Wegman M, Glancy L, Gasc JM, Brunotte F, Bruneval P, Wolf JE, Michel JB, Jeunemaitre X. Mutations in

myosin heavy chain 11 cause a syndrome associating thoracic aortic aneurysm/aortic dissection and patent ductus arteriosus. Nat Genet. 2006, 38(3):343-9.

41. Pan Y, Wang ZG, Liu XY, Zhao H, Zhou N, Zheng GF, Qiu XB, Li RG, Yuan F, Shi HY, Hou XM, Yang YQ. A Novel TBX1 Loss-of-Function Mutation Associated with Congenital Heart Disease. Pediatr Cardiol. 2015, 36:1400-10.

42. Shaheen R, Al Hashem A, Alghamdi MH, Seidahmad MZ, Wakil SM, Dagriri K, Keavney B, Goodship J, Alyousif S, Al-Habshan FM, Alhussein K, Almoisheer A, Ibrahim N, Alkuraya FS. Positional mapping of PRKD1, NRP1 and PRDM1 as novel candidate disease genes in truncus arteriosus. J Med Genet. 2015, 52(5):322-9.

43. Burnicka-Turek O, Steimle JD, Huang W, Felker L, Kamp A, Kweon J, Peterson M, Reeves RH, Maslen CL, Gruber PJ, Yang XH, Shendure J, Moskowitz IP. Cilia gene mutations cause atrioventricular septal defects by multiple mechanisms. Hum Mol Genet. 2016 [Epub ahead of print].

44. Al Turki S, Manickaraj AK, Mercer CL, Gerety SS, Hitz MP, Lindsay S, D'Alessandro LC, Swaminathan GJ, Bentham J, Arndt AK, Louw J, Breckpot J, Gewillig M, Thienpont B, Abdul-Khaliq H, Harnack C, Hoff K, Kramer HH, Schubert S, Siebert R, Toka O, Cosgrove C, Watkins H, Lucassen AM, O'Kelly IM, Salmon AP, Bu'lock FA, Granados-Riveron J, Setchfield K, Thornborough C, Brook JD, Mulder B, Klaassen S, Bhattacharya S, Devriendt K, Fitzpatrick DF; UK10K Consortium, Wilson DI, Mital S, Hurles ME. Rare variants in NR2F2 cause congenital heart defects in humans. Am J Hum Genet. 2014, 94(4):574-85.

45. Chang SW, Mislankar M, Misra C, Huang N, Dajusta DG, Harrison SM, McBride KL, Baker LA, Garg V. Genetic abnormalities in FOXP1 are associated with congenital heart defects. Hum Mutat. 2013, 34(9):1226-30.

46. Priest JR, Osoegawa K, Mohammed N, Nanda V, Kundu R, Schultz K, Lammer EJ, Girirajan S, Scheetz T, Waggott D, Haddad F, Reddy S, Bernstein D, Burns T, Steimle JD, Yang XH, Moskowitz IP, Hurles M, Lifton RP, Nickerson D, Bamshad M, Eichler EE, Mital S, Sheffield V, Quertermous T, Gelb BD, Portman M, Ashley EA. De Novo and Rare Variants at Multiple Loci Support the Oligogenic Origins of Atrioventricular Septal Heart Defects. PLoS Genet. 2016, 12(4):e1005963.

47. Werner P, Latney B, Deardorff MA, Goldmuntz E. MESP1 Mutations in Patients with Congenital Heart Defects. Hum Mutat. 2016, 37(3):308-14.

48. Bonachea EM, Chang SW, Zender G, LaHaye S, Fitzgerald-Butt S, McBride KL, Garg V. Rare GATA5 sequence variants identified in individuals with bicuspid aortic valve. Pediatr Res. 2014, 76(2):211-6.

49. Guimier A, Gabriel GC, Bajolle F, Tsang M, Liu H, Noll A, Schwartz M, El Malti R, Smith LD, Klena NT, Jimenez G, Miller NA, Oufadem M, Moreau de Bellaing A, Yagi H, Saunders CJ, Baker CN, Di Filippo S, Peterson KA, Thiffault I, Bole-Feysot C, Cooley LD, Farrow

EG, Masson C, Schoen P, Deleuze JF, Nitschké P, Lyonnet S, de Pontual L, Murray SA, Bonnet D, Kingsmore SF, Amiel J, Bouvagnet P, Lo CW, Gordon CT. MMP21 is mutated in human heterotaxy and is required for normal left-right asymmetry in vertebrates. Nat Genet. 2015, 47(11):1260-3.

50. Peyvandi S, Ingall E, Woyciechowski S, Garbarini J, Mitchell LE, Goldmuntz E. Risk of congenital heart disease in relatives of probands with conotruncal cardiac defects: an evaluation of 1,620 families. Am J Med Genet A. 2014, 164A(6):1490-5.
